# Elastic‒plastic analysis of rock surrounding a circular roadway considering plastic hardening and dilatancy characteristics

**DOI:** 10.1038/s41598-025-04006-3

**Published:** 2025-06-04

**Authors:** Peng Li, Ze Zhou, Youlin Xu, Yongjian Zhu, Bo Zhou, Changlun Sun, Xianqing Wang

**Affiliations:** 1 School of Mining Engineering, Guizhou Institute of Technology, Guiyang, 550003 Guizhou China; 2 Work Safety Key Lab on Prevention and Control of Gas and Roof Disasters for Southern Goal Mines, Hunan University of Science and Technology, Xiangtan, 411201 Hunan China

**Keywords:** Plastic hardening, Expansion characteristics, UST criterion, Expansion coefficient, Elastic‒plastic, Petrology, Mineralogy

## Abstract

Excavation of deep roadways induces plastic failure of the surrounding rock, resulting in roof caving and uncoordinated large deformation, which seriously threatens support safety and production efficiency. A four-stage stress‒strain model of the elastic zone‒plastic zone‒softening zone‒fracture zone of the surrounding rock in a deep roadway is established to analyze the range of the roadway fracture zone. The model considers the unified strength criterion, the nonassociated flow rule, and the influence of the intermediate principal stress and dilatancy coefficient. Based on this model, closed solutions for the stress, strain and deformation of the surrounding rock are obtained. Results show that an increase in the value of the strength parameter *b* can reduce the tangential stress *σ*_*θ*_ on the surface of the roadway but increase the values of *σ*_*θ*_ and *σ*_*r*_ at the plastic-flow interface. The peak value of *σ*_*θ*_ increases with increasing *b* value. Both the tangential strain *ε*_*θ*_ and radial strain *ε*_*r*_ decrease with increasing strength parameter *b*, and the radial displacement *u*_*r*_ decreases with increasing *b*. With the increase of initial cohesion, the post-peak failure range of surrounding rock shows a nonlinear increase, With the increase of residual cohesion, the post-peak failure range of surrounding rock shows a nonlinear decrease. The dilatancy coefficient *η*_*i*_ is positively correlated with the *b* value and the dilatancy angle *ψ*_*i*_, revealing that the surrounding rock with high dilatancy is more prone to volume expansion instability. The research results provide a theoretical basis and quantitative parameter support for stability evaluation and differential support design of deep roadways.

**Supplementary Information:**

The online version contains supplementary material available at 10.1038/s41598-025-04006-3.

Shallow coal resources in China are being continuously reduced. To meet the needs of rapid economic development, coal mining has shifted from east to west and from shallow to deep. However, the surrounding rock of a deep roadway is affected by ‘three high and one disturbance’, which easily shows the failure characteristics of a wide fracture range, large deformation, and severe failure of the supporting structure. Especially for soft rock roadways, there is an obvious fracture zone after excavation, and the range of the fracture zone continues to increase due to mining disturbances^1–3^. The distribution characteristics of the surrounding rock state constitute the theoretical basis for evaluating the overall stability of deep roadways and the reliability of quantitative support design^4–7^. The mechanical behavior of postpeak failure rock is an important basis for determining the change in the surrounding rock state. Therefore, elastic‒plastic analysis of the surrounding rock of circular roadways considering plastic hardening and dilatancy characteristics is of great theoretical importance and practical value^8–13^.

After the excavation of the circular roadway, the stress in the surrounding rock is redistributed, producing tangential stress and radial stress. In the elastic stage, the tangential stress decreases with increasing distance from the roadway wall, and the radial stress increases with increasing distance from the roadway wall. When the surrounding rock enters the plastic stage, the stress distribution becomes more complex. The magnitude and distribution of the tangential stress and radial stress are closely related to the plastic hardening and dilatancy characteristics of the surrounding rock^14,15^. Thus, after the excavation of the roadway, the surrounding rock deforms, including radial displacement and tangential displacement. In the elastic stage, the deformation of the surrounding rock is small and decreases with increasing distance from the roadway wall. When the surrounding rock enters the plastic stage, the deformation increases rapidly. The plastic hardening and expansion characteristics have important impacts on the development of deformation. Plastic hardening is one of the important characteristics of surrounding rock in the process of stress. After the excavation of the roadway, the redistribution of stress in the surrounding rock causes some areas to enter the plastic state and show a hardening phenomenon with the development of deformation; that is, the yield stress of the material increases with increasing plastic strain. This characteristic significantly affects the stress‒strain relationship and deformation law of the surrounding rock so that the surrounding rock still has a certain bearing capacity in the plastic deformation stage and that its bearing characteristics change dynamically with deformation^16,17^. For example, in the roadways of deep metal mines, the plastic hardening of the surrounding rock under high stress plays a key role in maintaining the short-term stability of the roadway. If this characteristic is ignored in the analysis process, the bearing capacity of the surrounding rock will be underestimated, which will lead to unreasonable support design or deviation of engineering safety assessment. Moreover, the expansion characteristics of the surrounding rock cannot be ignored. The volume of rock expands through the process of plastic deformation. This phenomenon is called dilatancy. The dilatancy changes the stress state and pore structure inside the surrounding rock, which not only affects the mechanical response of the surrounding rock itself but also couples with multiple physical fields, such as the seepage of groundwater and damage evolution of the rock mass^18–24^. Expansion is more obvious under the action of stress in coal mine roadway because the rock surrounding the coal seam is mostly brittle rock. This leads to the expansion of cracks around the roadway and an increase in permeability. The migration of harmful gases such as gas becomes complicated, which poses a serious threat to safety in mines.

Fenner first proposed the partition theory of roadway surrounding rock in 1938^25^. Under the premise of assuming that the volume of surrounding rock does not change, the stress solution method of the surrounding rock in the plastic zone of a circular roadway is given, that is, the Fenner formula. Kastner modified the Fenner formula considering the self-weight of the surrounding rock in 1951 and obtained the classical Kastner formula^26^, but the existence of cohesion in the surrounding rock in the plastic zone was not considered. Until the 1980s, Zheng et al. regarded the cohesion of the surrounding rock in the plastic zone as a constant and then revised the Fenner formula^27^. However, with increasing mining depth, many researchers have reported that the cohesion of the surrounding rock in the plastic zone is not a constant. In contrast, it changes with the change in circumferential strain. Brown et al.^28^ studied this phenomenon and proposed that the cohesion of the surrounding rock in the plastic zone changes linearly. Ma et al.^29^ summarized previous research results, fully considered the reduction in rock strength in the plastic stage, combined the characteristics of the total stress‒strain curve of rock, and proposed a two-stage strain softening model in 1995, which promoted the study of elastic‒plastic surrounding rock partition. A large step has laid a theoretical foundation for the subsequent three-stage model and four-stage model. Yuan Wenbo proposed the concept of the softening modulus according to the strain softening characteristics of the surrounding rock in 1986. Through the ideal elastic‒plastic softening model, a three-zone differentiation model of the elastic zone + plastic zone + broken zone of roadway surrounding rock was established, which ushered in the use of a three-stage model of roadway surrounding rock^30^. Compared with the three-zone differentiation model, relatively few studies have focused on the four-zone differentiation model. At present, there are differences in the evolution laws of the strength parameters and expansion parameters in the four-zone differentiation model, which mainly include the ‘*φ* weakening-*c* strengthening’ model^31^, the ‘*φ* strengthening-*c* weakening’ model^32^, the ‘*φ* weakening-*c* weakening’ model^33^ and the model whose strength parameters are consistent with the change trend of rock strength^34^. In fact, the strength and dilatancy parameters of the surrounding rock depend on the molecular structure and force chain state of the rock mass. Therefore, carrying out elastic‒plastic analysis of the rock surrounding a circular roadway through plastic hardening and dilatancy characteristics is highly theoretically and practically important. Through accurate theoretical modeling and analysis, the mechanical behavior and deformation mechanism of the surrounding rock in a complex stress environment can be understood more deeply, providing a solid theoretical basis for reasonable roadway support design, engineering disaster prediction and prevention. This study focuses on these two characteristics and systematically carries out elastic‒plastic analysis of the rock surrounding a circular roadway by means of elastic‒plastic mechanics theory, numerical simulation methods and other methods to provide strong technical support and scientific guidance for the stability and safety of underground engineering.

## Mechanics model of roadway surrounding rock

### Four-zone differentiation mechanical model of roadway surrounding rock

**Fig. 1:**
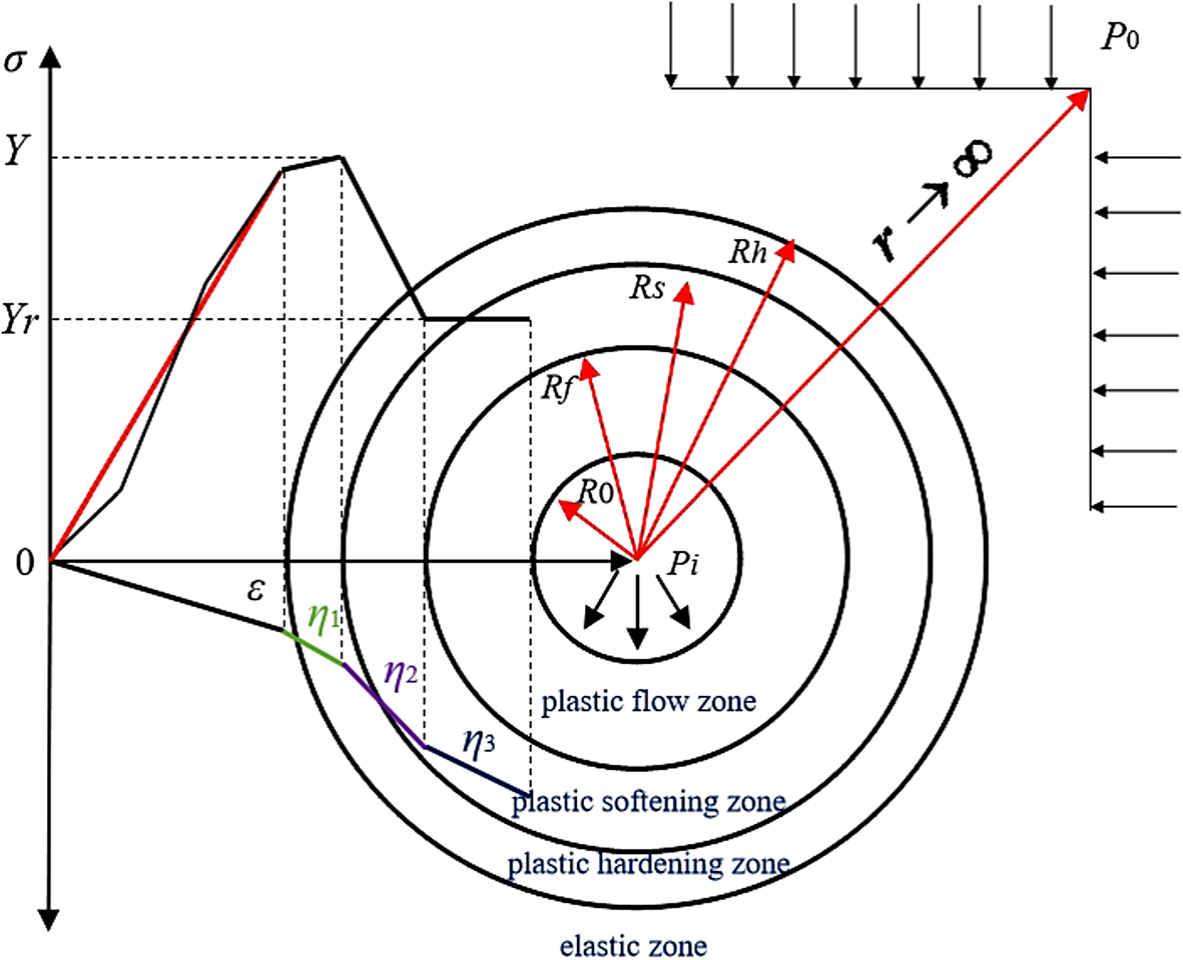
A four-zone differentiation mechanical model of roadway surrounding rock considering plastic hardening and dilatancy characteristics.

The excavation of deep high-stress roadways often causes the redistribution of surrounding rock stress. Before the excavation of a roadway, the surrounding rock is in the initial equilibrium state of three-dimensional stress, and the excavation disturbance breaks the original balance of stress. The surrounding rock away from the roadway is less affected and is still in a stable state of three-way stress. The closer to the surface of the roadway, the greater the impact, and the surrounding rock is in a two-way or even one-way stress state^14^. Therefore, a certain stress gradient field forms in the radial direction of the roadway, resulting in zonal deformation of the rock surrounding the roadway. When the stress state of the surrounding rock around the roadway exceeds the elastic limit of the rock mass and enters the plastic stress state, the surrounding rock around the roadway is divided into four zones from the free surface to the outside, namely, the plastic flow zone, the plastic softening zone, the plastic hardening zone and the elastic zone. With the expansion of the surrounding rock as the boundary, the mechanical behavior of the four zones of the surrounding rock around the roadway corresponds to the nonexpansion elastic stage (including the pore compaction, linear elasticity, and stable crack expansion stages), unstable crack development stage, rapid crack expansion stage and residual stage in the relationship between the total stress‒strain curve of the rock, as shown in Fig. 1. In the early stage of roadway excavation, the surrounding rock is essentially in an elastic state. When the maximum and minimum principal stresses satisfy the yield surface function and the strain increment in the principal stress direction satisfies the plastic flow rule, the surrounding rock experiences volume expansion and enters the plastic hardening state. The state is not infinite. The expansion range is constrained by the softening zone and the flow zone. When the increase in shear strain in the plastic zone satisfies a certain relationship, the surrounding rock begins to enter the strain softening state from the plastic state until the surrounding rock parameters reach a certain residual value. The rock begins to enter the fracture state and finally reaches a stable equilibrium state under the action of support resistance. The characteristics of roadway excavation are as follows: the excavation radius of the roadway is *R*_0_, the hydrostatic stress at infinity is *P*_0_, the support resistance is *P*_i_, and the radii of the plastic hardening zone, plastic softening zone and plastic flow zone are *R*_*h*_, *R*_*s*_ and *R*_*f*_, respectively. In addition, *Y* and *Y*_*r*_ represent the equivalent peak strength and residual strength, respectively. *η*_*i*_ represents the expansion coefficient of the surrounding rock in each zone, which can be determined by the plastic potential function. *η*_1_ represents the expansion coefficient of the plastic hardening zone, *η*_2_ represents the expansion coefficient of the plastic softening zone, and *η*_3_ represents the expansion coefficient of the plastic flow zone. The circumferential and radial stresses and the circumferential and radial strains of the rock surrounding the roadway are expressed by *σ*_*θi*_, *σ*_*ri*_, *ε*_*θi*_, and *ε*_*ri*_, respectively. Among them, the subscript’*i*'represents the symbols ‘*e'*, ‘*h'*, ‘*s'* and ‘*f'*, deliminating four different areas of surrounding rock.

### Unified strength criterion

The unified strength theory (UST) is based on the twin shear yield criterion, considering the influence of all stress components and intermediate principal stress on the yield failure of the material^35,36^. This theory is applicable to a variety of material media, and its expression forms are not the same. In geotechnical engineering, the cohesion (*c*) and internal friction angle (*φ*) of commonly used materials are expressed, and it is generally stipulated that the compressive stress is positive and the tensile stress is negative^27^, then the UST yield function can be expressed as :

1 $$\left\{ {\begin{array}{*{20}{c}} {{\sigma _2} \leqslant \frac{{{\sigma _1}+{\sigma _3}}}{2} - \frac{{{\sigma _1}+{\sigma _3}}}{2}\sin \varphi } \\ {\frac{{1 - \sin \varphi }}{{1+\sin \varphi }}{\sigma _1} - \frac{{b{\sigma _2}+{\sigma _3}}}{{1+b}}=\frac{{2c\cos \varphi }}{{1+\sin \varphi }}} \\ {{\sigma _2} \geqslant \frac{{{\sigma _1}+{\sigma _3}}}{2} - \frac{{{\sigma _1}+{\sigma _3}}}{2}\sin \varphi } \\ {\frac{{1 - \sin \varphi }}{{(1+b)(1+\sin \varphi )}}({\sigma _1}+b{\sigma _2}) - {\sigma _3}=\frac{{2c\cos \varphi }}{{1+\sin \varphi }}} \end{array}} \right.$$

where *σ*_1_, *σ*_2_, and *σ*_3_ represent the maximum, intermediate, and minimum principal stresses, respectively, in MPa. *φ* is the internal friction angle of the rock, °. *b* is the strength parameter in the unified strength criterion. When *b* = 0, the unified strength criterion (UST) is transformed into the Mohr‒Coulomb strength criterion. When *b* = 1, it is transformed into the twin shear strength criterion.

Research shows that the following relationships exist among the three principal stresses^10^:

2 $${\sigma _2}=\frac{{n({\sigma _1}+{\sigma _3})}}{2}$$

where *n* generally satisfies the condition of 2ν ≤ *n* ≤ 1. When *n* → 1, *σ*_2_ is closer to the average of the maximum and minimum principal stresses. *ν* denotes the Poisson’s ratio of the rock material. To simplify the calculation process, *n* = 1 is used in this paper. At this time, UST can be rewritten as:

3 $${\sigma _1}={N_p}{\sigma _3}+Y$$

Equation (3) shows that when *n* = 1, the UST strength criterion is the same as the Mohr‒Coulomb strength criterion:

4 $$\left\{ {\begin{array}{*{20}{c}} {{N_p}=\frac{{2+b+(2+3b)\sin \varphi }}{{(2+b)(1 - \sin \varphi )}}} \\ {Y=\frac{{4(1+b)c\sin \varphi }}{{(2+b)(1 - \sin \varphi )}}} \end{array}} \right.$$

where *N*_*p*_ and *Y* are the parameters considering the progressive damage of rock, which are related to the cohesion *c*, internal friction angle *φ*, and strength parameter *b*. *c* is rock cohesion, MPa.

For axisymmetric plane strain problems:

5 $${\sigma _\theta }={N_p}{\sigma _r}+{Y_i}$$

where ${Y_i}=\frac{{4(1+b){c_i}\sin \varphi }}{{(2+b)(1 - \sin \varphi )}}$ and *c*_*i*_ represents the cohesion of the’*h* ‘,'*s ‘*, and’*f'* regions.

### Definition of the expansion coefficient

In the process of plastic deformation of roadway surrounding rock, the phenomenon of volume expansion is called expansion. The plastic deformation of rock is nonlinear and generally satisfies the nonassociated flow rule, which can be expressed by the plastic potential function:

6 $$g\left( {{\sigma _\theta },{\sigma _r}} \right)={\sigma _\theta } - {n_i}{\sigma _r}$$

where ${n_i}=\frac{{2+b+(2+3b)\sin {\psi _i}}}{{(2+b)(1 - \sin {\psi _i})}}$ and *ψ*_*i*_ represents the dilatancy angle in the’*i*'interval.

According to the plastic potential theory, the relationship between the stress increment and strain increment is satisfied by^8^:

7 $$d\varepsilon _{{ij}}^{p}=d\lambda \frac{{\partial g}}{{\partial {\sigma _{ij}}}}$$

where $d\varepsilon _{{ij}}^{p}$ and *σ*_*ij*_ are the plastic strain increment and stress tensor, respectively; and *dλ* is a nonnegative proportional constant.

By substituting Eq. (1) into Eq. (2), the tangential plastic strain increment $d\varepsilon _{\theta }^{p}$ and radial plastic strain increment $d\varepsilon _{r}^{p}$ of the rock surrounding the roadway are obtained as follows:

8 $$\left\{ {\begin{array}{*{20}{c}} {d\varepsilon _{\theta }^{p}=d\lambda \frac{{\partial g}}{{\partial {\sigma _\theta }}}=d\lambda } \\ {d\varepsilon _{r}^{p}=d\lambda \frac{{\partial g}}{{\partial {\sigma _r}}}= - {\eta _i}d\lambda } \end{array}} \right.$$

where *η*_*i*_ is the parameter introduced considering the expansion characteristics of the rock surrounding the roadway. When the volume of the rock mass is expanded, *η*_*i*_ > 1; otherwise, *η*_*i*_ = 1.

On the basis of the nonassociated plastic flow rule, the increase in the tangential and radial plastic strains of the surrounding rock can be obtained as follows:

9 $$\Delta \varepsilon _{r}^{p}+{\eta _i}\Delta \varepsilon _{\theta }^{p}=0$$

According to Eq. (4), the expansion coefficient expression of the plastic hardening zone is obtained as follows:

10 $${\eta _1}= - \frac{{\Delta \varepsilon _{{rh}}^{p}}}{{\Delta \varepsilon _{{\theta h}}^{p}}}$$

Similarly, the expansion coefficient of the plastic softening zone is expressed as:

11 $${\eta _2}= - \frac{{\Delta \varepsilon _{{rs}}^{p}}}{{\Delta \varepsilon _{{\theta s}}^{p}}}$$

The expression of the expansion coefficient of the plastic flow zone is:

12 $${\eta _3}= - \frac{{\Delta \varepsilon _{{rf}}^{p}}}{{\Delta \varepsilon _{{\theta f}}^{p}}}$$

## Model solution

### Basic equation

For axisymmetric plane strain problems, the stresses in each region satisfy the equilibrium differential equations:

13 $$\frac{{d{\sigma _r}}}{{dr}}+\frac{{{\sigma _r} - {\sigma _\theta }}}{r}=0$$

The geometric equation is as follows:

14 $$\left\{ {\begin{array}{*{20}{c}} {{\varepsilon _r}=\frac{{du}}{{dr}}} \\ {{\varepsilon _\theta }=\frac{u}{r}} \end{array}} \right.$$

where *u* is the radial displacement and *r* is the polar diameter.

The stress boundary conditions satisfied by the model can be summarized as follows:

15 $$\left\{ {\begin{array}{*{20}{c}} {{\sigma _{re}}={P_0},\left( {r \to \infty } \right)} \\ {{\sigma _{re}}={\sigma _{rh}}={\sigma _{reh}},\left( {r={R_h}} \right)} \\ {{\sigma _{rh}}={\sigma _{rs}}={\sigma _{rhs}},\left( {r={R_s}} \right)} \\ {{\sigma _{rs}}={\sigma _{rf}}={\sigma _{rsf}},\left( {r={R_f}} \right)} \\ {{\sigma _{rf}}={P_i},\left( {r={R_0}} \right)} \end{array}} \right.$$

### Analysis of the stress, strain, and displacement in the elastic zone

Combined with the theory of elastic mechanics, the expressions of stress, strain and displacement in the elastic zone are as follows:

16 $$\left\{ {\begin{array}{*{20}{ll}} {{\sigma _{re}}={P_0}+\left( {{\sigma _{reh}} - {P_0}} \right){{\left( {\frac{{{R_h}}}{r}} \right)}^2}} \\ {{\sigma _{\theta e}}={A_0}{{\left( {\frac{{{R_h}}}{r}} \right)}^2}} \\ {{u_e}={A_0}\frac{{{R_h}^{2}}}{r}} \\ {{\varepsilon _{re}}= - {A_0}{{\left( {\frac{{{R_h}}}{r}} \right)}^2}} \\ {{\varepsilon _{r\theta }}={A_0}{{\left( {\frac{{{R_h}}}{r}} \right)}^2}} \end{array}} \right.$$

where ${A_0}=\frac{{1+\mu }}{E}\left( {{P_0} - {\sigma _{reh}}} \right)$ and *E* is the elastic modulus of the rock, MPa.

At the interface between the elastic zone and the initial plastic hardening zone, that is, *r* = *R*_*h*_, the surrounding rock is in the elastic‒plastic critical state, so its tangential and radial stresses meet the UST criterion. Therefore, substituting Eq. (16) into Eq. (5), it is easy to obtain the following expression:

17 $${\sigma _{reh}}=\frac{{2{P_0} - Y}}{{1+{N_p}}}$$

### Analysis of stress, strain, and displacement in the plastic hardening zone

The surrounding rock in the plastic zone should meet the stress equilibrium Eq. (13) and the yield criterion Eq. (5). Combined with the boundary condition ${({\sigma _{rh}})_{r={R_h}}}={\sigma _{reh}}=\frac{{2{P_0} - Y}}{{1+{N_p}}}$, the stress expression of the plastic zone is as follows:

18 $$\left\{ {\begin{array}{*{20}{ll}} {{\sigma _{rh}}={A_1}{{(\frac{{{R_h}}}{r})}^{1 - {N_p}}}+A{}_{2}} \\ {{\sigma _{\theta h}}={N_p}{A_1}{{(\frac{{{R_h}}}{r})}^{1 - {N_p}}}+A{}_{2}} \end{array}} \right.$$

where $${A_1}=\frac{{2{P_0}(1 - {N_p}) - 2Y}}{{1 - {N_p}^{2}}},{A_2}=\frac{Y}{{1 - {N_p}}}$$.

According to Eq. (18), when *r* = *R*_*s*_, the radial stress at the interface between the plastic hardening zone and the plastic softening zone is:

19 $${\sigma _{rhs}}={A_1}{(\frac{{{R_h}}}{{{R_s}}})^{1 - {N_p}}}+A{}_{2}$$

Assuming that the strain in the plastic zone is only composed of plastic strain, the geometric Eq. (14) is substituted into Eq. (9). Combined with the displacement boundary conditions, the displacement and strain expressions of the plastic hardening zone can be obtained as follows:

20 $$\left\{ {\begin{array}{*{20}{ll}} {{u_{rh}}={A_0}{R_h}^{{1+{\eta _1}}}{r^{ - {\eta _1}}}} \\ {{\varepsilon _{rh}}= - {\eta _1}{A_0}{{(\frac{{{R_h}}}{r})}^{^{{1+{\eta _1}}}}}} \\ {{\varepsilon _{\theta h}}={A_0}{{(\frac{{{R_h}}}{r})}^{^{{1+{\eta _1}}}}}} \end{array}} \right.$$

### Analysis of the stress, strain, and displacement in the plastic softening zone

In the softening zone, assuming that the attenuation of rock strength is related only to cohesion *c*, the parameter *Y*_*s*_ (*r*) in the softening zone should satisfy the following relationship [106]:

21 $${Y_s}\left( r \right)=Y - \lambda \left[ {{\varepsilon _{\theta s}}\left( r \right) - {{\left( {{\varepsilon _{\theta s}}} \right)}_{r={R_s}}}} \right]$$

where *λ* is the softening coefficient and where ${\varepsilon _{\theta s}}\left( r \right)$ is the tangential strain at any point in the softening zone.

According to the plastic zone strain solution method, Eq. (14) is substituted into Eq. (9). Combined with the radial displacement continuity condition ${({u_{rs}})_{r={R_s}}}={({u_{rh}})_{r={R_s}}}={A_0}{R_h}^{{1+{\eta _1}}}{R_s}^{{ - {\eta _1}}}$, the strain and radial displacement expressions of the softening zone can be obtained as follows:

22 $$\left\{ {\begin{array}{*{20}{ll}} {{u_{rs}}={A_0}{R_h}^{{1+{\eta _1}}}{R_s}^{{{\eta _2} - {\eta _1}}}{r^{ - {\eta _2}}}} \\ {{\varepsilon _{rs}}= - {\eta _2}{A_0}{{(\frac{{{R_h}}}{{{R_s}}})}^{^{{1+{\eta _1}}}}}{{(\frac{{{R_s}}}{r})}^{^{{1+{\eta _2}}}}}} \\ {{\varepsilon _{\theta s}}={A_0}{{(\frac{{{R_h}}}{{{R_s}}})}^{^{{1+{\eta _1}}}}}{{(\frac{{{R_s}}}{r})}^{^{{1+{\eta _2}}}}}} \end{array}} \right.$$

According to Eq. (22), the tangential strain ${\left( {{\varepsilon _{\theta s}}} \right)_{r={R_s}}}$ at the junction of the plastic hardening zone and the softening zone can be expressed as:

23 $${\left( {{\varepsilon _{\theta s}}} \right)_{r={R_s}}}={A_0}{(\frac{{{R_h}}}{{{R_s}}})^{^{{1+{\eta _1}}}}}$$

Substituting Eq. (23) and Eq. (22) into Eq. (21), we obtain:

24 $${Y_s}\left( r \right)=Y - \lambda \left[ {{A_0}{{(\frac{{{R_h}}}{{{R_s}}})}^{^{{1+{\eta _1}}}}}{{(\frac{{{R_s}}}{r})}^{^{{1+{\eta _2}}}}} - {A_0}{{(\frac{{{R_h}}}{{{R_s}}})}^{^{{1+{\eta _1}}}}}} \right]$$

From Eqs. (21) and (5), the constitutive relation of the surrounding rock in the softening zone is as follows:

25 $${\sigma _{\theta s}}={N_p}{\sigma _{rs}}+Y - \lambda \left[ {{A_0}{{(\frac{{{R_h}}}{{{R_s}}})}^{^{{1+{\eta _1}}}}}{{(\frac{{{R_s}}}{r})}^{^{{1+{\eta _2}}}}} - {A_0}{{(\frac{{{R_h}}}{{{R_s}}})}^{^{{1+{\eta _1}}}}}} \right]$$

Substituting Eq. (25) into equilibrium Eq. (13), combined with the stress contact condition ${({\sigma _{rs}})_{r={R_s}}}={\sigma _{rhs}}={A_1}{(\frac{{{R_h}}}{{{R_s}}})^{1 - {N_p}}}+A{}_{2}$ at *r* = *R*_*s*_, the stress expression in the softening zone can be obtained as follows:

26 $$\left\{ {\begin{array}{*{20}{ll}} \begin{gathered} {\sigma _{rs}}={A_1}{\left( {\frac{{{R_h}}}{r}} \right)^{1 - {N_p}}}+{A_2}+{A_3}{\left( {\frac{{{R_h}}}{{{R_s}}}} \right)^{1+{\eta _1}}}\left[ {{{\left( {\frac{{{R_s}}}{r}} \right)}^{1+{\eta _2}}} - {{\left( {\frac{{{R_s}}}{r}} \right)}^{1 - {N_p}}}} \right] \\ +{A_4}{\left( {\frac{{{R_h}}}{{{R_s}}}} \right)^{1+{\eta _1}}}\left[ {1 - {{\left( {\frac{{{R_s}}}{r}} \right)}^{1 - {N_p}}}} \right] \\ \end{gathered} \\ \begin{gathered} {\sigma _{\theta s}}={N_p}\left\{ {{A_1}{{\left( {\frac{{{R_h}}}{r}} \right)}^{1 - {N_p}}}+{A_2}+{A_3}{{\left( {\frac{{{R_h}}}{{{R_s}}}} \right)}^{1+{\eta _1}}}\left[ {{{\left( {\frac{{{R_s}}}{r}} \right)}^{1+{\eta _2}}} - {{\left( {\frac{{{R_s}}}{r}} \right)}^{1 - {N_p}}}} \right]} \right. \\ \left. {+{A_4}{{\left( {\frac{{{R_h}}}{{{R_s}}}} \right)}^{1+{\eta _1}}}\left[ {1 - {{\left( {\frac{{{R_s}}}{r}} \right)}^{1 - {N_p}}}} \right]} \right\}Y - \lambda \left[ {{A_0}{{(\frac{{{R_h}}}{{{R_s}}})}^{^{{1+{\eta _1}}}}}{{(\frac{{{R_s}}}{r})}^{^{{1+{\eta _2}}}}} - {A_0}{{(\frac{{{R_h}}}{{{R_s}}})}^{^{{1+{\eta _1}}}}}} \right] \\ \end{gathered} \end{array}} \right.$$

where $${A_3}=\frac{{\lambda {A_0}}}{{{\eta _2}+{N_p}}},{A_4}=\frac{{\lambda {A_0}}}{{1 - {N_p}}}$$.

### Analysis of stress, strain, and displacement in the plastic flow zone

Combined with the boundary condition ${({\sigma _{rf}})_{r={R_0}}}={P_i}$, the stress expression of the plastic flow zone is as follows:

27 $$\left\{ {\begin{array}{*{20}{c}} {{\sigma _{rf}}={A_5}{{\left( {\frac{r}{a}} \right)}^{{N_p} - 1}}+{A_6}} \\ {{\sigma _{\theta f}}={N_p}{A_5}{{\left( {\frac{r}{a}} \right)}^{{N_p} - 1}}+{A_6}} \end{array}} \right.$$

where ${A_5}=\frac{{(1 - {N_p}){P_i} - {Y_r}}}{{1 - {N_p}}},{A_6}=\frac{{{Y_r}}}{{1 - {N_p}}}$, ${Y_r}=\frac{{4(1+b){c_r}\sin \varphi }}{{(2+b)(1 - \sin \varphi )}}$, and *c*_*r*_ is the residual cohesion, MPa.

Combined with the displacement boundary condition ${({u_{rf}})_{r={R_f}}}={({u_{rs}})_{r={R_f}}}={A_0}{R_h}^{{1+{\eta _1}}}{R_s}^{{{\eta _2} - {\eta _1}}}{R_f}^{{ - {\eta _2}}}$, the expressions of strain and radial displacement in the plastic flow zone can be obtained as follows:

28 $$\left\{ {\begin{array}{*{20}{c}} {{u_{rf}}={A_0}{R_h}^{{1+{\eta _1}}}{R_s}^{{{\eta _2} - {\eta _1}}}{R_f}^{{{\eta _3} - {\eta _2}}}{r^{ - {\eta _3}}}} \\ {{\varepsilon _{rf}}= - {\eta _3}{A_0}{{(\frac{{{R_h}}}{{{R_s}}})}^{^{{1+{\eta _1}}}}}{{(\frac{{{R_s}}}{{{R_f}}})}^{^{{1+{\eta _2}}}}}{{(\frac{{{R_f}}}{r})}^{^{{1+{\eta _3}}}}}} \\ {{\varepsilon _{\theta f}}={A_0}{{(\frac{{{R_h}}}{{{R_s}}})}^{^{{1+{\eta _1}}}}}{{(\frac{{{R_s}}}{{{R_f}}})}^{^{{1+{\eta _2}}}}}{{(\frac{{{R_f}}}{r})}^{^{{1+{\eta _3}}}}}} \end{array}} \right.$$

### Radius analysis of each zone

By combining Eqs. (18) and (26), one of the relationships between *R*_*h*_, *R*_*s*_, *R*_*f*_, and the roadway radius *R*_0_ can be obtained as follows:

29 $$\begin{gathered} {A_1}{\left( {\frac{{{R_h}}}{{{R_f}}}} \right)^{1 - {N_p}}}+{A_2}+{A_3}{\left( {\frac{{{R_h}}}{{{R_s}}}} \right)^{1+{\eta _1}}}\left[ {{{\left( {\frac{{{R_s}}}{{{R_f}}}} \right)}^{1+{\eta _2}}} - {{\left( {\frac{{{R_s}}}{{{R_f}}}} \right)}^{1 - {N_p}}}} \right] \hfill \\ +{A_4}{\left( {\frac{{{R_h}}}{{{R_s}}}} \right)^{1+{\eta _1}}}\left[ {1 - {{\left( {\frac{{{R_s}}}{{{R_f}}}} \right)}^{1 - {N_p}}}} \right]={A_5}{\left( {\frac{{{R_f}}}{a}} \right)^{{N_p} - 1}}+{A_6} \hfill \\ \end{gathered}$$

In general, the expansion of the plastic zone is limited under high confining pressure conditions, which are limited and constrained by the softening zone and the fracture zone. The shear strain increment $\Delta \overline {\gamma }$ of the plastic zone can be used to represent the expansion of the plastic zone. When $\Delta \overline {\gamma }$ reaches a certain value, the surrounding rock begins to enter the softening stage from the plastic stage. At this time, $\Delta \overline {\gamma }$ can be expressed as:

30 $$\Delta \overline {\gamma } =\Delta \overline {{{\gamma _s}}} - \Delta \overline {{{\gamma _h}}} ={({\varepsilon _{\theta h}} - {\varepsilon _{rh}})_{r={R_s}}} - {({\varepsilon _{\theta h}} - {\varepsilon _{rh}})_{r={R_h}}}$$

The relationship between the radius of the plastic hardening zone and the plastic softening zone can be obtained by substituting Eq. (20) into Eq. (30) as follows:

31 $${R_h}={\left[ {\frac{{\Delta \overline {\gamma } }}{{{A_0}(1+{\eta _1})}}} \right]^{\frac{1}{{1+{\eta _1}}}}}{R_s}=T{R_s}$$

where $$T={\left[ {\frac{{\Delta \overline {\gamma } }}{{{A_0}(1+{\eta _1})}}} \right]^{\frac{1}{{1+{\eta _1}}}}}$$.

Equation (21) shows that in the plastic softening zone, the softening coefficient *λ* can be determined by the slope (softening modulus) of the plastic softening straight line segment in the simplified mechanical model of the rock surrounding the roadway (Fig. 1). The expression is as follows:

32 $$\lambda =\frac{{Y - {Y_r}}}{{{{\left( {{\varepsilon _{\theta s}}} \right)}_{r={R_f}}} - {{\left( {{\varepsilon _{\theta s}}} \right)}_{r={R_s}}}}}=\frac{{Y - {Y_r}}}{{{A_0}{{(\frac{{{R_h}}}{{{R_s}}})}^{^{{1+{\eta _1}}}}}{{(\frac{{{R_s}}}{{{R_f}}})}^{^{{1+{\eta _2}}}}} - {A_0}{{(\frac{{{R_h}}}{{{R_s}}})}^{^{{1+{\eta _1}}}}}}}$$

Substituting Eq. (31) into Eq. (32), the relationship between the radius of the plastic softening zone and the plastic flow zone can be obtained after finishing:

33 $${R_s}={\left( {\frac{{Y - {Y_r}}}{{\lambda {A_0}{T^{1+{\eta _1}}}}}+1} \right)^{\frac{1}{{1+{\eta _2}}}}}{R_f}=t{R_f}$$

where $$t={\left( {\frac{{Y - {Y_r}}}{{\lambda {A_0}{T^{1+{\eta _1}}}}}+1} \right)^{\frac{1}{{1+{\eta _2}}}}}$$.

By substituting Eq. (32) and Eq. (33) into Eq. (29), the relationship between the plastic flow zone and the radius of the roadway can be obtained after sorting:

34 $${R_f}={\left( {\frac{{\left[ {{A_1}{T^{1 - {N_p}}} - ({A_3}+{A_4}){T^{1+{\eta _1}}}} \right]{t^{1+{\eta _2}}}+\left( {{A_3}{t^{1+{\eta _2}}}+{A_4}} \right){T^{1+{\eta _1}}}+{A_2} - {A_6}}}{{{A_5}}}} \right)^{\frac{1}{{{N_p} - 1}}}}{R_0}$$

By substituting Eq. (34) into Eq. (31) and Eq. (32), the specific expressions of the plastic softening zone radius *R*_*s*_, plastic hardening zone *R*_*h*_ and roadway radius *R*_0_ can be obtained.

## Case analysis

### Engineering conditions

According to the theoretical analysis results of the previous section, the radius, stress, strain and displacement state of each zone of rock surrounding the roadway are related to the values of initial in situ stress *P*_0_, support resistance *P*_*i*_, elastic modulus *E*, Poisson’s ratio *µ*, initial internal friction angle *φ*_0_, initial cohesion *c*_0_, residual cohesion *c*_*r*_, dilatancy angle *ψ* and strength parameter *b*. The values of each parameter are as follows: roadway radius *R*_0_ = 3 m, initial ground stress *P*_0_ = 23.4 MPa, support resistance *P*_*i*_ = 0 MPa, elastic modulus *E* = 30 GPa, Poisson’s ratio *µ* = 0.25, initial internal friction angle *φ*_0_ = 30°, initial cohesion *c*_0_ = 5.85 MPa, residual cohesion *c*_*r*_ = 2 MPa, dilatancy angle *ψ*_*h*_ = *ψ*_*s*_ = *ψ*_*f*_ = *ψ*_0_ = 15°, and strength parameter *b* = 0. To effectively reflect the influence of the strength parameters, expansion coefficient and model selection on the change in the surrounding rock state, the control variable method is used to analyze them one by one.

### Influence of the strength parameter B on the stress state of the surrounding rock

**Fig. 2:**
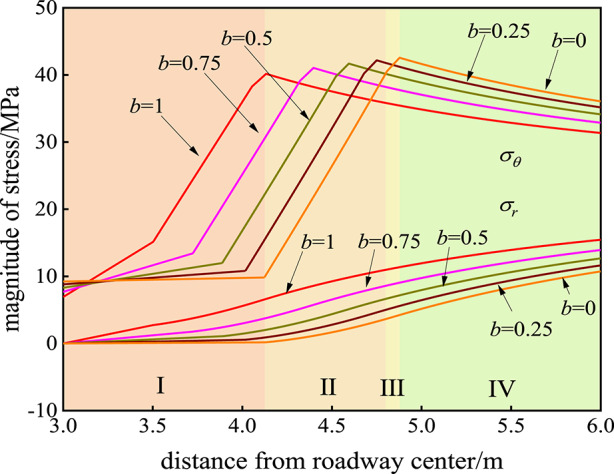
Influence of the strength parameter *b* on the stress field of the rock surrounding the roadway. I is the plastic flow zone, II the plastic softening zone, III the plastic hardening zone, and IV the elastic zone.

The influence of the strength parameter *b* on the stress field of the rock surrounding the roadway is shown in Fig. 2. When *b* = 0 (which is the Mohr‒Coulomb strength criterion), the tangential stress *σ*_*θ*_ at the interface between the plastic softening zone and the plastic flow zone is 9.838 MPa, and the radial stress *σ*_*r*_ is 0.162 MPa, which is obtained at 4.123 m from the center of the roadway. The maximum tangential stress *σ*_*θ*_ is 42.638 MPa, which is obtained at 4.881 m from the center of the roadway. When *b* = 0.25, the tangential stress *σ*_*θ*_ at the interface between the plastic softening zone and the plastic flow zone is 10.815 MPa, and the radial stress *σ*_*r*_ is 0.562 MPa, which is obtained at 4.017 m from the center of the roadway. The maximum tangential stress *σ*_*θ*_ is 42.296 MPa, which is obtained at 4.753 m from the center of the roadway. When *b* = 0.5, the tangential stress *σ*_*θ*_ at the interface between the plastic softening zone and the plastic flow zone is 11.999 MPa, and the radial stress *σ*_*r*_ is 1.08 MPa, which is obtained 3.888 m from the center of the roadway. The maximum tangential stress *σ*_*θ*_ is 41.689 MPa, which is obtained at 4.597 m from the center of the roadway. When *b* = 0.75, the tangential stress *σ*_*θ*_ at the interface between the plastic softening zone and the plastic flow zone is 13.429 MPa, and the radial stress *σ*_*r*_ is 1.774 MPa, which is obtained 3.724 m from the center of the roadway. The maximum tangential stress *σ*_*θ*_ is 41.082 MPa, which is obtained at 4.4 m from the center of the roadway. When *b* = 1 (the twin shear strength criterion), the tangential stress *σ*_*θ*_ at the interface between the plastic softening zone and the plastic flow zone is 15.171 MPa, and the radial stress *σ*_*r*_ is 2.745 MPa, which is obtained 3.503 m from the center of the roadway. The maximum tangential stress *σ*_*θ*_ is 40.133 MPa, which is obtained at 4.132 m from the center of the roadway. The influence of the strength parameter *b* on the stress field of the rock surrounding the roadway is as follows: with increasing strength parameter *b*, the tangential stress *σ*_*θ*_ of the rock surrounding the surface of the roadway decreases, but both the tangential stress *σ*_*θ*_ and the radial stress *σ*_*r*_ at the interface between the plastic softening zone and plastic flow zone increase. The peak value of the tangential stress *σ*_*θ*_ decreases with increasing strength parameter *b*, and the peak point shifts to the surface of the roadway, which is located at the elastic‒plastic interface.

### Influence of the strength parameter B on the strain state of the surrounding rock

**Fig. 3:**
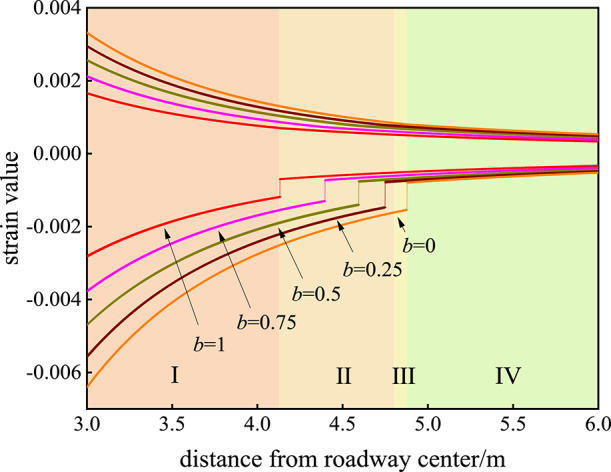
Influence of the strength parameter *b* on the strain field of the rock surrounding the roadway. I is the plastic flow zone, II the plastic softening zone, III the plastic hardening zone, and IV the elastic zone.

The influence of the strength parameter b on the strain field of the rock surrounding the roadway is shown in Fig. 3. When *b* = 0, the tangential strain of the surrounding rock on the surface of the roadway is 0.00332, and the radial strain is − 0.00641. When *b* = 0.25, the tangential strain of the surrounding rock on the surface of the roadway is 0.00295, and the radial strain is − 0.00557. When *b* = 0.5, the tangential strain of the surrounding rock on the surface of the roadway is 0.00255, and the radial strain is − 0.0047. When *b* = 0.75, the tangential strain *ε*_*θ*_ of the surrounding rock on the roadway surface is 0.00213, and the radial strain *ε*_*r*_ is − 0.00377. When *b* = 1, the tangential strain of the surrounding rock on the surface of the roadway is 0.00166, and the radial strain is − 0.00282. The influence of the strength parameter *b* on the strain field of the rock surrounding the roadway is as follows: with increasing strength parameter *b*, the tangential strain *ε*_*θ*_ and radial strain *ε*_*r*_ of the surrounding rock on the surface of the roadway both decrease, and the tangential strain *ε*_*θ*_ and radial strain *ε*_*r*_ of the surrounding rock decrease as a whole.

### Influence of the strength parameter *b* on the displacement state of the surrounding rock

**Fig. 4:**
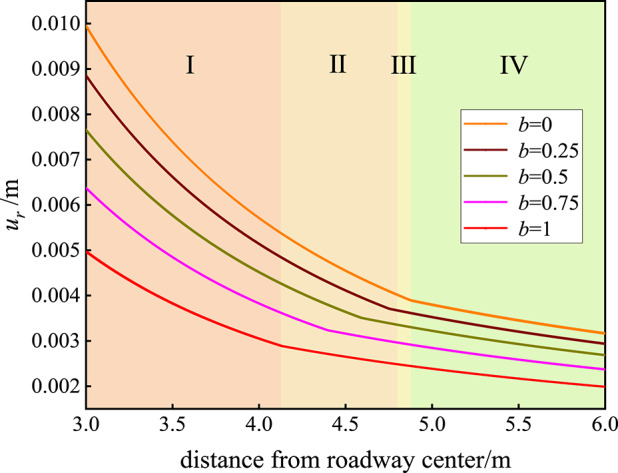
Influence of the strength parameter *b* on the displacement field of the rock surrounding the roadway. I is the plastic flow zone, II the plastic softening zone, III the plastic hardening zone, and IV the elastic zone.

The influence of the strength parameter *b* on the displacement field of the rock surrounding the roadway is shown in Fig. 4. When *b* = 0, the radial displacement *u*_*r*_ of the surrounding rock on the surface of the roadway is 0.00995 m; when *b* = 0.25, the radial displacement *u*_*r*_ of the surrounding rock on the surface of the roadway is 0.00885 m; when *b* = 0.5, the radial displacement *u*_*r*_ of the surrounding rock on the surface of the roadway is 0.00766 m; when *b* = 0.75, the radial displacement *u*_*r*_ of the surrounding rock on the surface of the roadway is 0.00638 m; and when *b* = 1, the radial displacement *u*_*r*_ of the surrounding rock on the roadway surface is 0.00497 m. The influence of strength parameter *b* on the displacement field of the rock surrounding the roadway is as follows: with increasing strength parameter *b*, the radial displacement of the surrounding rock on the surface of the roadway decreases, and the radial displacement of the surrounding rock on the surface of the roadway decreases as a whole.

### Effects of the strength parameter *b* on the postpeak failure range of the surrounding rock

**Fig. 5:**
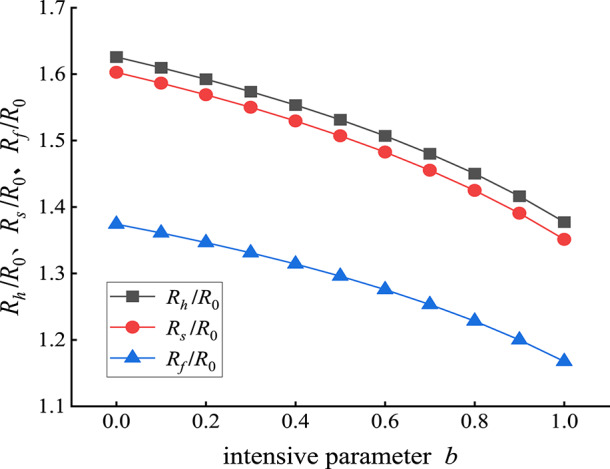
Changes in *R*_*h*_/*R*_0,_ *R*_*s*_/*R*_0,_ and *R*_*f*_/*R*_0_ with increasing intensity parameter *b*.

With the other parameters fixed, the strength parameter *b* is changed. The evolution laws of the plastic hardening zone *R*_*h*_/*R*_0_, plastic softening zone *R*_*s*_/*R*_0_ and plastic flow zone *R*_*f*_/*R*_0_ with the change in the strength parameter *b* are shown in Fig. 5. The radius of each partition of the roadway surrounding rock decreases to different degrees with increasing strength parameter *b*, and the rate of decrease gradually decreases, as shown in Fig. 5. For example, when the strength parameter *b* increases from 0 to 0.5, *R*_*h*_/*R*_0_, *R*_*s*_/*R*_0_ and *R*_*f*_/*R*_0_ decrease by 0.094, 0.096 and 0.078, respectively, and decrease by 5.81%, 5.96% and 5.7%, respectively. When the strength parameter *b* increases from 0.5 to 1, *R*_*h*_/*R*_0_, *R*_*s*_/*R*_0_ and *R*_*f*_/*R*_0_ decrease by 0.154, 0.156 and 0.128, respectively, and decrease by 10.1%, 10.3% and 9.9%, respectively.

These data show that when *b* changes from 0 to 1.0, the *R*_*h*_/*R*_0_ in the plastic hardening zone, the *R*_*s*_/*R*_0_ in the plastic softening zone and the *R*_*f*_/*R*_0_ in the plastic flow zone all gradually decrease. This finding shows that the Mohr‒Coulomb strength criterion easily estimates the plastic zone of the surrounding rock and improves the safety of support parameter design, but it also easily results in high support costs and waste of support strength.

### Effects of initial cohesion and residual cohesion on the postpeak failure range of the surrounding rock

Effects of initial cohesion and residual cohesion on the postpeak failure range of the surrounding rock

**Fig. 6:**
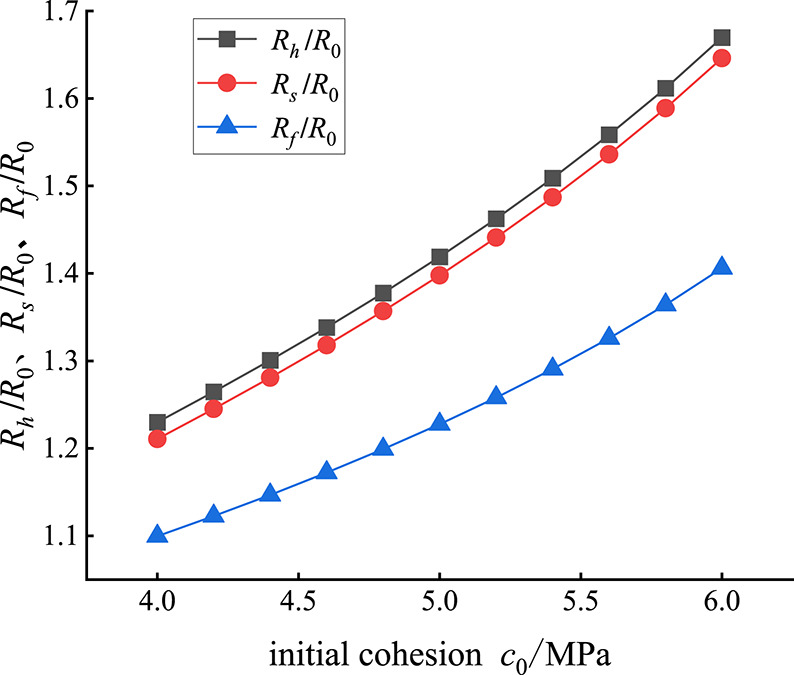
*R*_*h*_/*R*_0_, *R*_*s*_/*R*_0_, *R*_*f*_/*R*_0_ changes with *c*_0_.

When the other parameters are fixed, the initial cohesion *c*_0_ of the rock mass is changed, and the evolution of *R*_*h*_/*R*_0_ in the plastic hardening zone, *R*_*s*_/*R*_0_ in the plastic softening zone and *R*_*f*_/*R*_0_ in the plastic flow zone with initial cohesion *c*_0_ is obtained, as shown in Fig. 6. Figure 6 shows that with increasing initial cohesion *c*_0_, the radius of each partition of the rock surrounding the roadway increases to varying degrees, and the rate of increase gradually increases. The rates of increase in the plastic flow hardening radius *R*_*h*_/*R*_0_ and plastic softening zone radius *R*_*s*_/*R*_0_ are essentially the same, and the rate of increase in the plastic flow zone radius *R*_*h*_/*R*_0_ is the slowest. For example, when the initial cohesion *c*_0_ increases from 4 MPa to 5 MPa, *R*_*h*_/*R*_0_, *R*_*s*_/*R*_0_ and *R*_*f*_/*R*_0_ increase by 0.189, 0.187 and 0.128, respectively, by 15.4%, 15.5% and 11.6%, respectively. When the initial cohesion *c*_0_ increases from 5 MPa to 6 MPa, *R*_*h*_/*R*_0_, *R*_*s*_/*R*_0_ and *R*_*f*_/*R*_0_ increase by 0.251, 0.248 and 0.178, respectively, increasing by 17.7%, 17.8% and 14.5%, respectively.

**Fig. 7:**
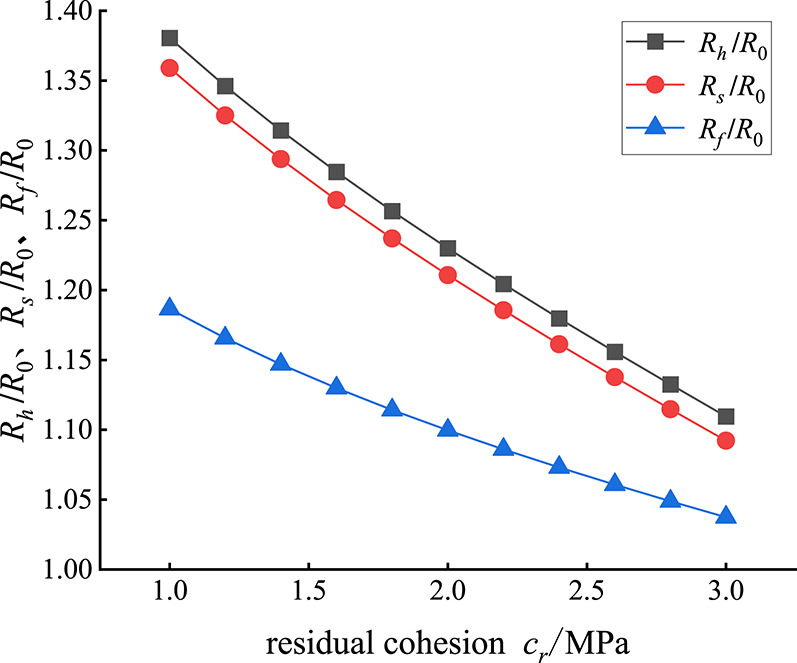
*R*_*h*_/*R*_0_, *R*_*s*_/*R*_0_, *R*_*f*_/*R*_0_ changes with *c*_*r*_.

When the other parameters are fixed, the residual cohesion *c*_*r*_ of the rock mass is changed, and the evolution laws of *R*_*h*_/*R*_0_ in the plastic hardening zone, *R*_*s*_/*R*_0_ in the plastic softening zone and *R*_*f*_/*R*_0_ in the plastic flow zone with the change in residual cohesion *c*_*r*_ are obtained, as shown in Fig. 7. Figure 7 shows that, unlike the initial cohesion, the radius of each zone of rock surrounding the roadway decreases with increasing residual cohesion *c*_*r*_, and the rate of decrease decreases gradually. The decreasing rate of the radius *R*_*h*_/*R*_0_ of the plastic hardening zone is equivalent to that of the radius *R*_*s*_/*R*_0_ of the plastic softening zone, and the decreasing rate of the radius *R*_*f*_/*R*_0_ of the plastic flow zone is the slowest. For example, when the residual cohesion cr increases from 1 MPa to 2 MPa, *R*_*h*_/*R*_0_, *R*_*s*_/*R*_0_ and *R*_*f*_/*R*_0_ decrease by 0.151, 0.148 and 0.087, respectively, by 10.9%, 10.9% and 7.3%, respectively. When the residual cohesion *c*_*r*_ increased from 2 MPa to 3 MPa, *R*_*h*_/*R*_0_, *R*_*s*_/*R*_0_ and *R*_*f*_/*R*_0_ decreased by 0.12, 0.118 and 0.062, respectively, by 9.8%, 9.8% and 5.7%, respectively.

The initial internal friction angle *φ*_0_ and initial cohesion *c*_0_ of the rock mass depend on the nature of the rock mass itself. Changing the initial internal friction angle *φ*_0_ and the initial cohesion *c*_0_ of the rock mass by artificial means is difficult. However, in roadway support, the size of the residual cohesion *c*_*r*_ can be fully considered, and the residual cohesion *c*_*r*_ of the broken rock mass in the plastic flow zone can be improved by means of grouting and support coupling, which can effectively limit the malignant expansion of the plastic zone of the surrounding rock.

### Effect of the strength parameter B on the expansion coefficient

**Fig. 8:**
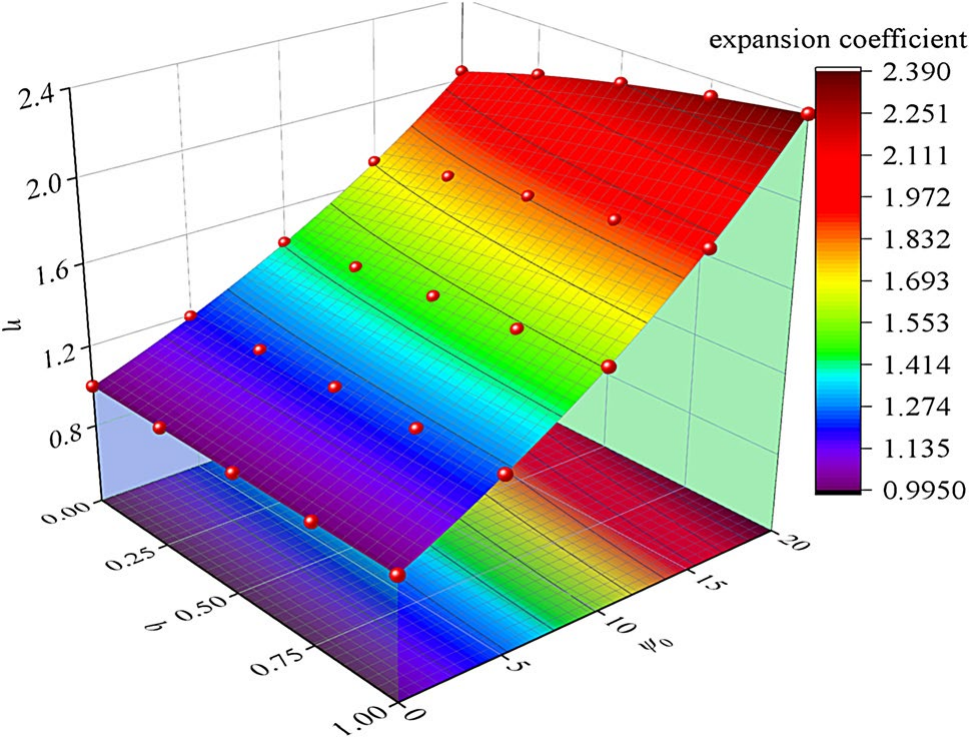
Three-dimensional curves of the relationships between the dilatancy coefficient and the dilatancy angle and strength parameters.

Figure 8 shows the influence of the dilatancy angle *ψ*_*i*_ and strength parameter b on the dilatancy coefficient *η*_*i*_. Figure 8 shows that the dilatancy coefficient *η*_*i*_ is positively correlated with the strength parameter *b* and the dilatancy angle *ψ*_*i*_. The data in Fig. 8 show the following. (1) Rocks with strong dilatancy are more likely to aggravate the instability of the surrounding rock. (2) When the transition process from the Mohr‒Coulomb strength criterion to the twin shear strength criterion (*b* from 0 to 1) occurs, the rock dilatancy effect is more significant, and the instability tendency of the surrounding rock is relatively intensified.

## Discussion

**Fig. 9:**
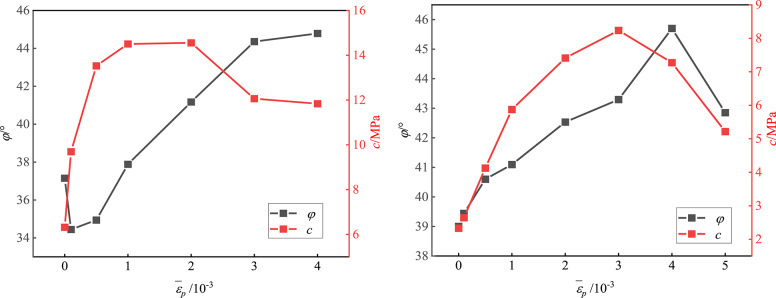
Strength parameter‒equivalent plastic strain curves of sandstone and sandy mudstone.

In fact, in the process of rock damage, the internal friction angle *φ* and cohesion *c* of rock exhibit nonlinear evolution. At present, there are still differences in the evolution of the internal friction angle *φ* and cohesion *c* in the four-zone differentiation model. There are mainly “*φ* weakening-*c* strengthening"models^31^,"*φ* strengthening-*c* weakening"models^32^,"*φ* weakening-*c* weakening"models^33^ and strength parameters that are consistent with the change trend of the rock strength model^34^. In this work, the equivalent plastic strain is introduced as a unified independent variable for different lithologies (sandstone and sandy mudstone) and different confining pressures. The nonlinear evolution of rock cohesion *c* and internal friction angle *φ* with equivalent plastic strain is analyzed, as shown in Fig. 9. The cohesion *c* and internal friction angle *φ* of different test samples evolve with increasing equivalent plastic strain ${\overline {\varepsilon } _p}$. In the early stage of deformation and failure of rock samples, *c* is the main factor influencing rock mass strength; with the expansion and connection of microcracks, the friction between crack surfaces gradually increases. Finally, the values of *c* and *φ* tend to be stable. In general, the cohesion *c* and internal friction angle *φ* of the test sandstone, sandy mudstone, and mudstone samples with an equivalent plastic strain ${\overline {\varepsilon } _p}$ conform to the constitutive model law of ‘*φ* weakening-*c* strengthening’(CWFS constitutive model)^31^.

**Fig. 10:**
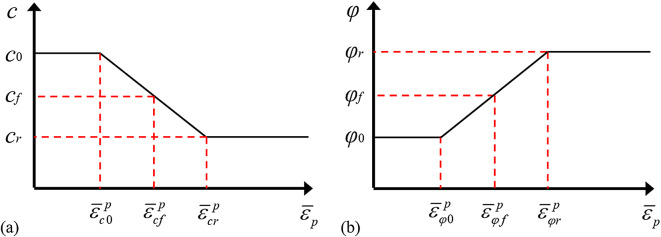
Evolution law of (**a**) rock strength parameters and (**b**) equivalent plastic strain.

To facilitate the calculation, a linear simplification method can be used to describe the evolution of the internal friction angle *φ* and the cohesion *c* with the equivalent plastic strain. As shown in Fig. 10, there is a piecewise linear function between *c*, *φ* and ${\overline {\varepsilon } _p}$. In the figure, *c*_0_ and *φ*_0_ are the initial cohesion and initial internal friction angle; *c*_*f*_ and *φ*_*f*_ are the cohesion and internal friction angle under peak stress; *c*_*r*_ and *φ*_*r*_ are the cohesion and internal friction angle under residual stress; and $\overline {\varepsilon } _{{c0}}^{p}$, $\overline {\varepsilon } _{{cf}}^{p}$, $\overline {\varepsilon } _{{cr}}^{p}$, $\overline {\varepsilon } _{{\varphi 0}}^{p}$, $\overline {\varepsilon } _{{\varphi f}}^{p}$ and $\overline {\varepsilon } _{{\varphi r}}^{p}$ are the corresponding critical plastic strain parameters.

By linearly simplifying the evolution of the internal friction angle *φ* and cohesion *c* with the equivalent plastic strain, the following conclusions can be drawn:

35 $$c=\left\{ {\begin{array}{*{20}{ll}} {{c_0},{{\bar {\varepsilon }}_p} \leqslant \bar {\varepsilon }_{{c0}}^{p}} \\ {{c_0} - \frac{{{c_0} - {c_r}}}{{\bar {\varepsilon }_{{cr}}^{p} - \bar {\varepsilon }_{{c0}}^{p}}}\left( {{{\bar {\varepsilon }}_p} - \bar {\varepsilon }_{{c0}}^{p}} \right),\bar {\varepsilon }_{{c0}}^{p}<{{\bar {\varepsilon }}_p}<\bar {\varepsilon }_{{cr}}^{p}} \\ {{c_r},{{\bar {\varepsilon }}_p} \geqslant \bar {\varepsilon }_{{cr}}^{p}} \end{array}} \right.$$

36 $$\varphi =\left\{ {\begin{array}{*{20}{ll}} {{\varphi _0},{{\bar {\varepsilon }}_p} \leqslant \bar {\varepsilon }_{{\varphi 0}}^{p}} \\ {{\varphi _0}+\frac{{{\varphi _r} - {\varphi _0}}}{{\bar {\varepsilon }_{{\varphi r}}^{p} - \bar {\varepsilon }_{{\varphi 0}}^{p}}}\left( {{{\bar {\varepsilon }}_p} - \bar {\varepsilon }_{{\varphi 0}}^{p}} \right),\bar {\varepsilon }_{{\varphi 0}}^{p}<{{\bar {\varepsilon }}_p}<\bar {\varepsilon }_{{\varphi r}}^{p}} \\ {{\varphi _r},{{\bar {\varepsilon }}_p} \geqslant \bar {\varepsilon }_{{\varphi r}}^{p}} \end{array}} \right.$$

In this work, elastoplastic analysis is based on the assumption that the attenuation of rock strength is related only to the cohesion *c*, ignoring the influence of the internal friction angle *φ* on the attenuation of rock strength. The next step is to study the influence of the rock internal friction angle *φ* and cohesion *c* on the elastoplastic analysis of roadway surrounding rock.

## Conclusion

(1) Taking the critical dilatancy point of rock as the boundary, a four-zone differentiation mechanical model of the elastic zone–plastic zone–softening zone–flow zone of the surrounding rock in a deep circular roadway is established. On the basis of the UST criterion and nonassociated flow rule and considering the influence of the intermediate principal stress and dilatancy coefficient, analytical solutions are found for the stress field, displacement field and plastic zone radius of the surrounding rock in a bidirectional isobaric circular roadway. The analytical solution can be used to invert the strength parameters of surrounding rock by inverting the displacement of on-site monitoring, which provides a quantitative basis for the design of bolt support length (through the flow zone) and grouting reinforcement range (covering the softening zone).

(2) Strength parameters have an important influence on changes in the surrounding rock state. With increasing strength parameter *b*, the tangential stress *σ*_*θ*_ of the rock surrounding the surface of the roadway decreases, but both the tangential stress *σ*_*θ*_ and the radial stress *σ*_*r*_ at the interface between the plastic softening zone and plastic flow zone increase, and the peak value of the tangential stress *σ*_*θ*_ decreases with increasing strength parameter *b*. The tangential strain *ε*_*θ*_ and radial strain *ε*_*r*_ of the surrounding rock decrease with increasing strength parameter *b*. The radial displacement *u*_*r*_ of the surrounding rock decreases with increasing strength parameter *b*. It provides a quantitative basis for the optimization of support parameters.

(3) With the increase of initial cohesion, the post-peak failure range of surrounding rock shows a nonlinear increase, and its increase rate is increasing. With the increase of residual cohesion, the post-peak failure range of surrounding rock shows a nonlinear decrease, and its reduction rate decreases continuously. In addition, based on the plastic potential theory, the influence of dilatancy angle *ψ*_*i*_ and strength parameter *b* on the dilatancy coefficient *η*_*i*_ is discussed. The results show that the dilatancy coefficient *η*_*i*_ is positively correlated with the strength parameter b and the dilatancy angle *ψ*_*i*_.

(4) Based on the linear simplification method, the evolution of rock strength parameters (cohesion *c* and internal friction angle *φ*) with the equivalent plastic strain is described. However, the current elastic-plastic analysis only considers the influence of cohesion *c* on the strength attenuation of rock, while ignoring the effect of internal friction angle *φ*. The subsequent research needs to comprehensively consider the coupling influence mechanism of c and *φ* on the elastoplastic response of roadway surrounding rock.

## Electronic supplementary material

Below is the link to the electronic supplementary material.

Supplementary Material 1

## Data Availability

All data generated or analyzed during this study are included in this published article [and its supplementary information files].

## References

[1] Chen, L. et al. Elastoplastic analysis of cracked surrounding rock in deep roadway based on Drucker–Prager criterion[J]. *J. China Coal Soc.*, (2). (2017).

[2] Zheng, W., Bu, Q. & Hu, Y. Plastic failure analysis of roadway floor surrounding rocks based on unified strength theory[J]. *Adv. Civil Eng.* **2018** (1), 7475698 (2018).

[3] Jiang, B. et al. Elastoplastic analysis of cracked surrounding rocks in deep circular openings[J]. *Chin. J. Rock Mechan. Eng.* **26** (5), 982–986 (2007).

[4] Guo, X. et al. Analytical solutions for characteristic radii of circular roadway surrounding rock plastic zone and their application[J]. *Int. J. Min. Sci. Technol.* **29** (2), 263–272 (2019).

[5] Li, J. et al. Study on shape characteristics of plastic zone in heterogeneous Roadway-Surrounding Rock[J]. *Sustainability* **14** (15), 9480 (2022).

[6] Wang, W. Dong E, Yuan, C. Boundary equation of plastic zone of circular roadway in nonaxisymmetric stress and its application[J]. Journal of China Coal Society, (1). (2019).

[7] Liu, H. et al. Approximate solution of plastic zone boundary of surrounding rock of circular roadway considering axial stress[J]. *Coal Sci. Technol.* **51** (10), 12–23 (2023).

[8] Zhang, Q. et al. A finite strain solution for strain-softening rock mass around circular roadways[J]. *Tunn. Undergr. Space Technol.* **111**, 103873 (2021).

[9] Zhang, X. B., Zhao, G. M., Meng, X. R. Elastoplastic analysis of surrounding rock on circular roadway based on Drucker Prager yield criterion[J]. *J. China Coal Soc.* **38** (1), 30–37 (2013).

[10] Huang, X. et al. Elasto-plastic analysis of the surrounding rock mass in circular tunnel based on the generalized nonlinear unified strength theory[J]. *Int. J. Min. Sci. Technol.* **26** (5), 819–823 (2016).

[11] Wang, X. et al. *Elastic–plastic Criterion Solution of Deep Roadway Surrounding Rock Based on Intermediate Principal Stress and Drucker–Prager Criterion[J]* (Energy Science & Engineering, 2024).

[12] Wang, R. et al. An innovative elastoplastic analysis for soft surrounding rock considering supporting opportunity based on Drucker-Prager strength Criterion[J]. *Adv. Civil Eng.* **2021** (1), 5555839 (2021).

[13] Wang, R. et al. The elastoplastic solutions of deep buried roadway based on the generalized 3D Hoek-Brown strength criterion considering Strain‐Softening Properties[J]. *Geofluids* **2021** (1), 5575376 (2021).

[14] Li, P. et al. Radial depth damage properties of coal tunnels surrounded by rock under excavation and unloading. *KSCE J. Civ. Eng.* **27**, 2287–2296 (2023).

[15] Ren, H. et al. Experimental study on mechanical characteristics of unloaded damaged white sandstone before peak. *Arab. J. Geosci.* **13**, 878 (2020).

[16] Cheng, L. et al. Characterization of crack evolution and hardening-softening in rock elastoplastic constitutive models[J]. *Comput. Geotech.* **176**, 106711 (2024).

[17] Zhou, J. et al. Research on nonlinear damage hardening creep model of soft surrounding rock under the stress of deep coal resources mining[J]. *Energy Rep.* **8**, 1493–1507 (2022).

[18] Xiao-bo, Z., Guang-ming, Z. & Xiang-rui, M. Elastoplastic solution for surrounding rock of circular roadway based on DP criterion by considering post-peak strain softening and dilatancy[J]. *J. Min. Saf. Eng.* **30** (6), 903 (2013).

[19] Yuan, Z. et al. A unified solution for surrounding rock of roadway considering seepage, dilatancy, strain-softening and intermediate principal stress[J]. *Sustainability* **14** (13), 8099 (2022).

[20] Cai, W. et al. A post-peak dilatancy model for soft rock and its application in deep tunnel excavation[J]. *J. Rock Mech. Geotech. Eng.* **15** (3), 683–701 (2023).

[21] Huang, X. et al. Experimental study on the dilatancy and fracturing behavior of soft rock under unloading conditions[J]. *Int. J. Civil Eng.* **15**, 921–948 (2017).

[22] Kang, H. & Yi, K. Simulation study on dilatant and rheologic properties of soft rocks surrounding deep roadway and its application[J]. *J. China Coal Soc.* **48** (1), 15–33 (2023).

[23] Zheng, Z. Q. et al. Dilatancy and Multi-Scale failure characteristics of a foliated rock under triaxial confinement unloading conditions[J]. *Eng. Fail. Anal.* **160**, 108168 (2024).

[24] Liu, X. et al. Mechanical response and dilatancy characteristics of deep marble under different stress paths: A sight from energy dissipation[J]. *J. Cent. South. Univ.* **31** (6), 2070–2086 (2024).

[25] Huo, T. et al. Non-uniform failure mechanism and stability control of mining roadway under deviatoric stress field[J]. *Sci. Rep.* **15** (1), 306–306 (2025). 39747297 10.1038/s41598-024-83355-x PMC11696908

[26] Liang, Y. et al. Study on the evolution law of the ‘Butterfly-type’ plastic zone in the surrounding rock of deep dynamic pressure roadway[J]. *Sci. Rep.* **15** (1), 2731–2731 (2025). 39837958 10.1038/s41598-025-85634-7 PMC11751150

[27] He, X. et al. Random phase field model for simulating mixed fracture modes in spatially variable rocks under impact loading[J]. *Int. J. Impact Eng.*, 196105174–196105174. (2025).

[28] Brown, E. T. et al. Ground response curves for rock Tunnels[J]. *J. Geotech. Eng.* **109** (1), 15–39 (1983).

[29] Nianjie, M. A., Ji, L. I. & Zhiqiang, Z. H. A. O. Distribution of the deviatoric stress field and plastic zone in the cir-cular roadway surrounding rock[Jl. *J. China Univ. Min. Technol.* **44** (2), 206–213 (2015).

[30] Ren, X. et al. Development of similar materials for fluid-solid coupling model testing and application in damage constitutive models[J]. *Sci. Rep.* **14** (1), 14786 (2024). 38926465 10.1038/s41598-024-65242-7 PMC11210313

[31] Meng, Q. et al. Experimental study on formation mechanism and mechanical properties of regenerated structure of very weak cemented rock mass[J]. *Rock. Soil. Mech.* **41** (03), 799–812 (2020).

[32] Hajiabdolmajid, V., Kaiser, P-K. & Martin, C-D. Modelling brittle failure of rock[J]. *Int. J. Rock Mech. Min. Sci.* **39** (6), 731–741 (2002).

[33] Lu, Y. et al. Post-peak strain softening mechanical properties of weak rock[J]. *Chin. J. Rock Mechan. Eng.* **29** (03), 640–648 (2010).

[34] Yuan, C. et al. Theoretical analysis on roadway surrounding rock deformation based on the properties of rock plastic hardening and softening[J]. *Journal·of·china·coal·society* **40** (S2), 311–319 (2015).

[35] Wang, C. et al. Elasto-plastic analysis of the surrounding rock mass in circular tunnel using a new numerical model based on generalized nonlinear unified strength theory[J]. *Comput. Geotech.* **154**, 105163 (2023).

[36] HAN, Z. Elastic-plastic analysis of surrounding rock in deep roadway based on four-stage stress-strain model [J]. *Coal Sci. Technol.* **50** (05), 84–91 (2022).

