# Supplementary material for: Elastic‒plastic analysis of rock surrounding a circular roadway considering plastic hardening and dilatancy characteristics
Source: Sci Rep. 2025 Jun 4;15:19540. doi: 10.1038/s41598-025-04006-3 (PMC12137801; doi:10.1038/s41598-025-04006-3)
Supplement: Supplementary file 1 — Supplementary Material 1 [file 41598_2025_4006_MOESM1_ESM.pdf]

b=1			b=0.75			b=0.5			b=0.25			b=0		
distance from roadway center/m	σ_r	σ_θ	distance from roadway center/m	σ_r	σ_θ	distance from roadway center/m	σ_r	σ_θ	distance from roadway center/m	σ_r	σ_θ	distance from roadway center/m	σ_r	σ_θ
3	0	6.9282	3	0	7.698	3	0	8.31384	3	0	8.81771	3	0	9.2376
3.001	0.00545	6.9446	3.001	0.00245	7.7059	3.001	0.00121	8.31799	3.001	0.00055	8.81967	3.001	0.000144	9.2381
3.002	0.0109	6.961	3.002	0.0049	7.7138	3.002	0.00242	8.32214	3.002	0.0011	8.82163	3.002	0.000288	9.2386
3.003	0.01635	6.9774	3.003	0.00735	7.7217	3.003	0.00363	8.32629	3.003	0.00165	8.82359	3.003	0.000432	9.2391
3.004	0.0218	6.9938	3.004	0.0098	7.7296	3.004	0.00484	8.33044	3.004	0.0022	8.82555	3.004	0.000576	9.2396
3.005	0.02725	7.0102	3.005	0.01225	7.7375	3.005	0.00605	8.33459	3.005	0.00275	8.82751	3.005	0.00072	9.2401
3.006	0.0327	7.0266	3.006	0.0147	7.7454	3.006	0.00726	8.33874	3.006	0.0033	8.82947	3.006	0.000864	9.2406
3.007	0.03815	7.043	3.007	0.01715	7.7533	3.007	0.00847	8.34289	3.007	0.00385	8.83143	3.007	0.001008	9.2411
3.008	0.0436	7.0594	3.008	0.0196	7.7612	3.008	0.00968	8.34704	3.008	0.0044	8.83339	3.008	0.001152	9.2416
3.009	0.04905	7.0758	3.009	0.02205	7.7691	3.009	0.01089	8.35119	3.009	0.00495	8.83535	3.009	0.001296	9.2421
3.01	0.0545	7.0922	3.01	0.0245	7.777	3.01	0.0121	8.35534	3.01	0.0055	8.83731	3.01	0.00144	9.2426
3.011	0.05995	7.1086	3.011	0.02695	7.7849	3.011	0.01331	8.35949	3.011	0.00605	8.83927	3.011	0.001584	9.2431
3.012	0.0654	7.125	3.012	0.0294	7.7928	3.012	0.01452	8.36364	3.012	0.0066	8.84123	3.012	0.001728	9.2436
3.013	0.07085	7.1414	3.013	0.03185	7.8007	3.013	0.01573	8.36779	3.013	0.00715	8.84319	3.013	0.001872	9.2441
3.014	0.0763	7.1578	3.014	0.0343	7.8086	3.014	0.01694	8.37194	3.014	0.0077	8.84515	3.014	0.002016	9.2446
3.015	0.08175	7.1742	3.015	0.03675	7.8165	3.015	0.01815	8.37609	3.015	0.00825	8.84711	3.015	0.00216	9.2451
3.016	0.0872	7.1906	3.016	0.0392	7.8244	3.016	0.01936	8.38024	3.016	0.0088	8.84907	3.016	0.002304	9.2456
3.017	0.09265	7.207	3.017	0.04165	7.8323	3.017	0.02057	8.38439	3.017	0.00935	8.85103	3.017	0.002448	9.2461
3.018	0.0981	7.2234	3.018	0.0441	7.8402	3.018	0.02178	8.38854	3.018	0.0099	8.85299	3.018	0.002592	9.2466
3.019	0.10355	7.2398	3.019	0.04655	7.8481	3.019	0.02299	8.39269	3.019	0.01045	8.85495	3.019	0.002736	9.2471
3.02	0.109	7.2562	3.02	0.049	7.856	3.02	0.0242	8.39684	3.02	0.011	8.85691	3.02	0.00288	9.2476
3.021	0.11445	7.2726	3.021	0.05145	7.8639	3.021	0.02541	8.40099	3.021	0.01155	8.85887	3.021	0.003024	9.2481
3.022	0.1199	7.289	3.022	0.0539	7.8718	3.022	0.02662	8.40514	3.022	0.0121	8.86083	3.022	0.003168	9.2486
3.023	0.12535	7.3054	3.023	0.05635	7.8797	3.023	0.02783	8.40929	3.023	0.01265	8.86279	3.023	0.003312	9.2491
3.024	0.1308	7.3218	3.024	0.0588	7.8876	3.024	0.02904	8.41344	3.024	0.0132	8.86475	3.024	0.003456	9.2496
3.025	0.13625	7.3382	3.025	0.06125	7.8955	3.025	0.03025	8.41759	3.025	0.01375	8.86671	3.025	0.0036	9.2501
3.026	0.1417	7.3546	3.026	0.0637	7.9034	3.026	0.03146	8.42174	3.026	0.0143	8.86867	3.026	0.003744	9.2506
3.027	0.14715	7.371	3.027	0.06615	7.9113	3.027	0.03267	8.42589	3.027	0.01485	8.87063	3.027	0.003888	9.2511
3.028	0.1526	7.3874	3.028	0.0686	7.9192	3.028	0.03388	8.43004	3.028	0.0154	8.87259	3.028	0.004032	9.2516
3.029	0.15805	7.4038	3.029	0.07105	7.9271	3.029	0.03509	8.43419	3.029	0.01595	8.87455	3.029	0.004176	9.2521
3.03	0.1635	7.4202	3.03	0.0735	7.935	3.03	0.0363	8.43834	3.03	0.0165	8.87651	3.03	0.00432	9.2526
3.031	0.16895	7.4366	3.031	0.07595	7.9429	3.031	0.03751	8.44249	3.031	0.01705	8.87847	3.031	0.004464	9.2531
3.032	0.1744	7.453	3.032	0.0784	7.9508	3.032	0.03872	8.44664	3.032	0.0176	8.88043	3.032	0.004608	9.2536
3.033	0.17985	7.4694	3.033	0.08085	7.9587	3.033	0.03993	8.45079	3.033	0.01815	8.88239	3.033	0.004752	9.2541
3.034	0.1853	7.4858	3.034	0.0833	7.9666	3.034	0.04114	8.45494	3.034	0.0187	8.88435	3.034	0.004896	9.2546
3.035	0.19075	7.5022	3.035	0.08575	7.9745	3.035	0.04235	8.45909	3.035	0.01925	8.88631	3.035	0.00504	9.2551
3.036	0.1962	7.5186	3.036	0.0882	7.9824	3.036	0.04356	8.46324	3.036	0.0198	8.88827	3.036	0.005184	9.2556
3.037	0.20165	7.535	3.037	0.09065	7.9903	3.037	0.04477	8.46739	3.037	0.02035	8.89023	3.037	0.005328	9.2561
3.038	0.2071	7.5514	3.038	0.0931	7.9982	3.038	0.04598	8.47154	3.038	0.0209	8.89219	3.038	0.005472	9.2566
3.039	0.21255	7.5678	3.039	0.09555	8.0061	3.039	0.04719	8.47569	3.039	0.02145	8.89415	3.039	0.005616	9.2571
3.04	0.218	7.5842	3.04	0.098	8.014	3.04	0.0484	8.47984	3.04	0.022	8.89611	3.04	0.00576	9.2576
3.041	0.22345	7.6006	3.041	0.10045	8.0219	3.041	0.04961	8.48399	3.041	0.02255	8.89807	3.041	0.005904	9.2581
3.042	0.2289	7.617	3.042	0.1029	8.0298	3.042	0.05082	8.48814	3.042	0.0231	8.90003	3.042	0.006048	9.2586
3.043	0.23435	7.6334	3.043	0.10535	8.0377	3.043	0.05203	8.49229	3.043	0.02365	8.90199	3.043	0.006192	9.2591
3.044	0.2398	7.6498	3.044	0.1078	8.0456	3.044	0.05324	8.49644	3.044	0.0242	8.90395	3.044	0.006336	9.2596
3.045	0.24525	7.6662	3.045	0.11025	8.0535	3.045	0.05445	8.50059	3.045	0.02475	8.90591	3.045	0.00648	9.2601
3.046	0.2507	7.6826	3.046	0.1127	8.0614	3.046	0.05566	8.50474	3.046	0.0253	8.90787	3.046	0.006624	9.2606
3.047	0.25615	7.699	3.047	0.11515	8.0693	3.047	0.05687	8.50889	3.047	0.02585	8.90983	3.047	0.006768	9.2611
3.048	0.2616	7.7154	3.048	0.1176	8.0772	3.048	0.05808	8.51304	3.048	0.0264	8.91179	3.048	0.006912	9.2616
3.049	0.26705	7.7318	3.049	0.12005	8.0851	3.049	0.05929	8.51719	3.049	0.02695	8.91375	3.049	0.007056	9.2621
3.05	0.2725	7.7482	3.05	0.1225	8.093	3.05	0.0605	8.52134	3.05	0.0275	8.91571	3.05	0.0072	9.2626
3.051	0.27795	7.7646	3.051	0.12495	8.1009	3.051	0.06171	8.52549	3.051	0.02805	8.91767	3.051	0.007344	9.2631
3.052	0.2834	7.781	3.052	0.1274	8.1088	3.052	0.06292	8.52964	3.052	0.0286	8.91963	3.052	0.007488	9.2636
3.053	0.28885	7.7974	3.053	0.12985	8.1167	3.053	0.06413	8.53379	3.053	0.02915	8.92159	3.053	0.007632	9.2641
3.054	0.2943	7.8138	3.054	0.1323	8.1246	3.054	0.06534	8.53794	3.054	0.0297	8.92355	3.054	0.007776	9.2646
3.055	0.29975	7.8302	3.055	0.13475	8.1325	3.055	0.06655	8.54209	3.055	0.03025	8.92551	3.055	0.00792	9.2651
3.056	0.3052	7.8466	3.056	0.1372	8.1404	3.056	0.06776	8.54624	3.056	0.0308	8.92747	3.056	0.008064	9.2656
3.057	0.31065	7.863	3.057	0.13965	8.1483	3.057	0.06897	8.55039	3.057	0.03135	8.92943	3.057	0.008208	9.2661
3.058	0.3161	7.8794	3.058	0.1421	8.1562	3.058	0.07018	8.55454	3.058	0.0319	8.93139	3.058	0.008352	9.2666
3.059	0.32155	7.8958	3.059	0.14455	8.1641	3.059	0.07139	8.55869	3.059	0.03245	8.93335	3.059	0.008496	9.2671
3.06	0.327	7.9122	3.06	0.147	8.172	3.06	0.0726	8.56284	3.06	0.033	8.93531	3.06	0.00864	9.2676
3.061	0.33245	7.9286	3.061	0.14945	8.1799	3.061	0.07381	8.56699	3.061	0.03355	8.93727	3.061	0.008784	9.2681
3.062	0.3379	7.945	3.062	0.1519	8.1878	3.062	0.07502	8.57114	3.062	0.0341	8.93923	3.062	0.008928	9.2686
3.063	0.34335	7.9614	3.063	0.15435	8.1957	3.063	0.07623	8.57529	3.063	0.03465	8.94119	3.063	0.009072	9.2691
3.064	0.3488	7.9778	3.064	0.1568	8.2036	3.064	0.07744	8.57944	3.064	0.0352	8.94315	3.064	0.009216	9.2696
3.065	0.35425	7.9942	3.065	0.15925	8.2115	3.065	0.07865	8.58359	3.065	0.03575	8.94511	3.065	0.00936	9.2701
3.066	0.3597	8.0106	3.066	0.1617	8.2194	3.066	0.07986	8.58774	3.066	0.0363	8.94707	3.066	0.009504	9.2706
3.067	0.36515	8.027	3.067	0.16415	8.2273	3.067	0.08107	8.59189	3.067	0.03685	8.94903	3.067	0.009648	9.2711
3.068	0.3706	8.0434	3.068	0.1666	8.2352	3.068	0.08228	8.59604	3.068	0.0374	8.95099	3.068	0.009792	9.2716
3.069	0.37605	8.0598	3.069	0.16905	8.2431	3.069	0.08349	8.60019	3.069	0.03795	8.95295	3.069	0.009936	9.2721
3.07	0.3815	8.0762	3.07	0.1715	8.251	3.07	0.0847	8.60434	3.07	0.0385	8.95491	3.07	0.01008	9.2726
3.071	0.38695	8.0926	3.071	0.17395	8.2589	3.071	0.08591	8.60849	3.071	0.03905	8.95687	3.071	0.010224	9.2731
3.072	0.3924	8.109	3.072	0.1764	8.2668	3.072	0.08712	8.61264	3.072	0.0396	8.95883	3.072	0.010368	9.2736
3.073	0.39785	8.1254	3.073	0.17885	8.2747	3.073	0.08833	8.61679	3.073	0.04015	8.96079	3.073	0.010512	9.2741
3.074	0.4033	8.1418	3.074	0.1813	8.2826	3.074	0.08954	8.62094	3.074	0.0407	8.96275	3.074	0.010656	9.2746
3.075	0.40875	8.1582	3.075	0.18375	8.2905	3.075	0.09075	8.62509	3.075	0.04125	8.96471	3.075	0.0108	9.2751
3.076	0.4142	8.1746	3.076	0.1862	8.2984	3.076	0.09196	8.62924	3.076	0.0418	8.96667	3.076	0.010944	9.2756
3.077	0.41965	8.191	3.077	0.18865	8.3063	3.077	0.09317	8.63339	3.077	0.04235	8.96863	3.077	0.011088	9.2761
3.078	0.4251	8.2074	3.078	0.1911	8.3142	3.078	0.09438	8.63754	3.078	0.0429	8.97059	3.078	0.011232	9.2766
3.079	0.43055	8.2238	3.079	0.19355	8.3221	3.079	0.09559	8.64169	3.079	0.04345	8.97255	3.079	0.011376	9.2771
3.08	0.436	8.2402	3.08	0.196	8.33	3.08	0.0968	8.64584	3.08	0.044	8.97451	3.08	0.01152	9.2776
3.081	0.44145	8.2566	3.081	0.19845	8.3379	3.081	0.09801	8.64999	3.081	0.04455	8.97647	3.081	0.011664	9.2781
3.082	0.4469	8.273	3.082	0.2009	8.3458	3.082	0.09922	8.65414	3.082	0.0451	8.97843	3.082	0.011808	9.2786
3.083	0.45235	8.2894	3.083	0.20335	8.3537	3.083	0.10043	8.65829	3.083	0.04565	8.98039	3.083	0.011952	9.2791
3.084	0.4578	8.3058	3.084	0.2058	8.3616	3.084	0.10164	8.66244	3.084	0.0462	8.98235	3.084	0.012096	9.2796
3.085	0.46325	8.3222	3.085	0.20825	8.3695	3.085	0.10285	8.66659	3.085	0.04675	8.98431	3.085	0.01224	9.2801
3.086	0.4687	8.3386	3.086	0.2107	8.3774	3.086	0.10406	8.67074	3.086	0.0473	8.98627	3.086	0.012384	9.2806
3.087	0.47415	8.355	3.087	0.21315	8.3853	3.087	0.10527	8.67489	3.087	0.04785	8.98823	3.087	0.012528	9.2811
3.088	0.4796	8.3714	3.088	0.2156	8.3932	3.088	0.10648	8.67904	3.088	0.0484	8.99019	3.088	0.012672	9.2816
3.089	0.48505	8.3878	3.089	0.21805	8.4011	3.089	0.10769	8.68319	3.089	0.04895	8.99215	3.089	0.012816	9.2821
3.09	0.4905	8.4042	3.09	0.2205	8.409	3.09	0.1089	8.68734	3.09	0.0495	8.99411	3.09	0.01296	9.2826
3.091	0.49595	8.4206	3.091	0.22295	8.4169	3.091	0.11011	8.69149	3.091	0.05005	8.99607	3.091	0.013104	9.2831
3.092	0.5014	8.437	3.092	0.2254	8.4248	3.092	0.11132	8.69564	3.092	0.0506	8.99803	3.092	0.013248	9.2836
3.093	0.50685	8.4534	3.093	0.22785	8.4327	3.093	0.11253	8.69979	3.093	0.05115	8.99999	3.093	0.013392	9.2841
3.094	0.5123	8.4698	3.094	0.2303	8.4406	3.094	0.11374	8.70394	3.094	0.0517	9.00195	3.094	0.013536	9.2846
3.095	0.51775	8.4862	3.095	0.23275	8.4485	3.095	0.11495	8.70809	3.095	0.05225	9.00391	3.095	0.01368	9.2851
3.096	0.5232	8.5026	3.096	0.2352	8.4564	3.096	0.11616	8.71224	3.096	0.0528	9.00587	3.096	0.013824	9.2856
3.097	0.52865	8.519	3.097	0.23765	8.4643	3.097	0.11737	8.71639	3.097	0.05335	9.00783	3.097	0.013968	9.2861
3.098	0.5341	8.5354	3.098	0.2401	8.4722	3.098	0.11858	8.72054	3.098	0.0539	9.00979	3.098	0.014112	9.2866
3.099	0.53955	8.5518	3.099	0.24255	8.4801	3.099	0.11979	8.72469	3.099	0.05445	9.01175	3.099	0.014256	9.2871
3.1	0.545	8.5682	3.1	0.245	8.488	3.1	0.121	8.72884	3.1	0.055	9.01371	3.1	0.0144	9.2876
3.101	0.55045	8.5846	3.101	0.24745	8.4959	3.101	0.12221	8.73299	3.101	0.05555	9.01567	3.101	0.014544	9.2881
3.102	0.5559	8.601	3.102	0.2499	8.5038	3.102	0.12342	8.73714	3.102	0.0561	9.01763	3.102	0.014688	9.2886
3.103	0.56135	8.6174	3.103	0.25235	8.5117	3.103	0.12463	8.74129	3.103	0.05665	9.01959	3.103	0.014832	9.2891
3.104	0.5668	8.6338	3.104	0.2548	8.5196	3.104	0.12584	8.74544	3.104	0.0572	9.02155	3.104	0.014976	9.2896
3.105	0.57225	8.6502	3.105	0.25725	8.5275	3.105	0.12705	8.74959	3.105	0.05775	9.02351	3.105	0.01512	9.2901
3.106	0.5777	8.6666	3.106	0.2597	8.5354	3.106	0.12826	8.75374	3.106	0.0583	9.02547	3.106	0.015264	9.2906
3.107	0.58315	8.683	3.107	0.26215	8.5433	3.107	0.12947	8.75789	3.107	0.05885	9.02743	3.107	0.015408	9.2911
3.108	0.5886	8.6994	3.108	0.2646	8.5512	3.108	0.13068	8.76204	3.108	0.0594	9.02939	3.108	0.015552	9.2916
3.109	0.59405	8.7158	3.109	0.26705	8.5591	3.109	0.13189	8.76619	3.109	0.05995	9.03135	3.109	0.015696	9.2921
3.11	0.5995	8.7322	3.11	0.2695	8.567	3.11	0.1331	8.77034	3.11	0.0605	9.03331	3.11	0.01584	9.2926
3.111	0.60495	8.7486	3.111	0.27195	8.5749	3.111	0.13431	8.77449	3.111	0.06105	9.03527	3.111	0.015984	9.2931
3.112	0.6104	8.765	3.112	0.2744	8.5828	3.112	0.13552	8.77864	3.112	0.0616	9.03723	3.112	0.016128	9.2936
3.113	0.61585	8.7814	3.113	0.27685	8.5907	3.113	0.13673	8.78279	3.113	0.06215	9.03919	3.113	0.016272	9.2941
3.114	0.6213	8.7978	3.114	0.2793	8.5986	3.114	0.13794	8.78694	3.114	0.0627	9.04115	3.114	0.016416	9.2946
3.115	0.62675	8.8142	3.115	0.28175	8.6065	3.115	0.13915	8.79109	3.115	0.06325	9.04311	3.115	0.01656	9.2951
3.116	0.6322	8.8306	3.116	0.2842	8.6144	3.116	0.14036	8.79524	3.116	0.0638	9.04507	3.116	0.016704	9.2956
3.117	0.63765	8.847	3.117	0.28665	8.6223	3.117	0.14157	8.79939	3.117	0.06435	9.04703	3.117	0.016848	9.2961
3.118	0.6431	8.8634	3.118	0.2891	8.6302	3.118	0.14278	8.80354	3.118	0.0649	9.04899	3.118	0.016992	9.2966
3.119	0.64855	8.8798	3.119	0.29155	8.6381	3.119	0.14399	8.80769	3.119	0.06545	9.05095	3.119	0.017136	9.2971
3.12	0.654	8.8962	3.12	0.294	8.646	3.12	0.1452	8.81184	3.12	0.066	9.05291	3.12	0.01728	9.2976
3.121	0.65945	8.9126	3.121	0.29645	8.6539	3.121	0.14641	8.81599	3.121	0.06655	9.05487	3.121	0.017424	9.2981
3.122	0.6649	8.929	3.122	0.2989	8.6618	3.122	0.14762	8.82014	3.122	0.0671	9.05683	3.122	0.017568	9.2986
3.123	0.67035	8.9454	3.123	0.30135	8.6697	3.123	0.14883	8.82429	3.123	0.06765	9.05879	3.123	0.017712	9.2991
3.124	0.6758	8.9618	3.124	0.3038	8.6776	3.124	0.15004	8.82844	3.124	0.0682	9.06075	3.124	0.017856	9.2996
3.125	0.68125	8.9782	3.125	0.30625	8.6855	3.125	0.15125	8.83259	3.125	0.06875	9.06271	3.125	0.018	9.3001
3.126	0.6867	8.9946	3.126	0.3087	8.6934	3.126	0.15246	8.83674	3.126	0.0693	9.06467	3.126	0.018144	9.3006
3.127	0.69215	9.011	3.127	0.31115	8.7013	3.127	0.15367	8.84089	3.127	0.06985	9.06663	3.127	0.018288	9.3011
3.128	0.6976	9.0274	3.128	0.3136	8.7092	3.128	0.15488	8.84504	3.128	0.0704	9.06859	3.128	0.018432	9.3016
3.129	0.70305	9.0438	3.129	0.31605	8.7171	3.129	0.15609	8.84919	3.129	0.07095	9.07055	3.129	0.018576	9.3021
3.13	0.7085	9.0602	3.13	0.3185	8.725	3.13	0.1573	8.85334	3.13	0.0715	9.07251	3.13	0.01872	9.3026
3.131	0.71395	9.0766	3.131	0.32095	8.7329	3.131	0.15851	8.85749	3.131	0.07205	9.07447	3.131	0.018864	9.3031
3.132	0.7194	9.093	3.132	0.3234	8.7408	3.132	0.15972	8.86164	3.132	0.0726	9.07643	3.132	0.019008	9.3036
3.133	0.72485	9.1094	3.133	0.32585	8.7487	3.133	0.16093	8.86579	3.133	0.07315	9.07839	3.133	0.019152	9.3041
3.134	0.7303	9.1258	3.134	0.3283	8.7566	3.134	0.16214	8.86994	3.134	0.0737	9.08035	3.134	0.019296	9.3046
3.135	0.73575	9.1422	3.135	0.33075	8.7645	3.135	0.16335	8.87409	3.135	0.07425	9.08231	3.135	0.01944	9.3051
3.136	0.7412	9.1586	3.136	0.3332	8.7724	3.136	0.16456	8.87824	3.136	0.0748	9.08427	3.136	0.019584	9.3056
3.137	0.74665	9.175	3.137	0.33565	8.7803	3.137	0.16577	8.88239	3.137	0.07535	9.08623	3.137	0.019728	9.3061
3.138	0.7521	9.1914	3.138	0.3381	8.7882	3.138	0.16698	8.88654	3.138	0.0759	9.08819	3.138	0.019872	9.3066
3.139	0.75755	9.2078	3.139	0.34055	8.7961	3.139	0.16819	8.89069	3.139	0.07645	9.09015	3.139	0.020016	9.3071
3.14	0.763	9.2242	3.14	0.343	8.804	3.14	0.1694	8.89484	3.14	0.077	9.09211	3.14	0.02016	9.3076
3.141	0.76845	9.2406	3.141	0.34545	8.8119	3.141	0.17061	8.89899	3.141	0.07755	9.09407	3.141	0.020304	9.3081
3.142	0.7739	9.257	3.142	0.3479	8.8198	3.142	0.17182	8.90314	3.142	0.0781	9.09603	3.142	0.020448	9.3086
3.143	0.77935	9.2734	3.143	0.35035	8.8277	3.143	0.17303	8.90729	3.143	0.07865	9.09799	3.143	0.020592	9.3091
3.144	0.7848	9.2898	3.144	0.3528	8.8356	3.144	0.17424	8.91144	3.144	0.0792	9.09995	3.144	0.020736	9.3096
3.145	0.79025	9.3062	3.145	0.35525	8.8435	3.145	0.17545	8.91559	3.145	0.07975	9.10191	3.145	0.02088	9.3101
3.146	0.7957	9.3226	3.146	0.3577	8.8514	3.146	0.17666	8.91974	3.146	0.0803	9.10387	3.146	0.021024	9.3106
3.147	0.80115	9.339	3.147	0.36015	8.8593	3.147	0.17787	8.92389	3.147	0.08085	9.10583	3.147	0.021168	9.3111
3.148	0.8066	9.3554	3.148	0.3626	8.8672	3.148	0.17908	8.92804	3.148	0.0814	9.10779	3.148	0.021312	9.3116
3.149	0.81205	9.3718	3.149	0.36505	8.8751	3.149	0.18029	8.93219	3.149	0.08195	9.10975	3.149	0.021456	9.3121
3.15	0.8175	9.3882	3.15	0.3675	8.883	3.15	0.1815	8.93634	3.15	0.0825	9.11171	3.15	0.0216	9.3126
3.151	0.82295	9.4046	3.151	0.36995	8.8909	3.151	0.18271	8.94049	3.151	0.08305	9.11367	3.151	0.021744	9.3131
3.152	0.8284	9.421	3.152	0.3724	8.8988	3.152	0.18392	8.94464	3.152	0.0836	9.11563	3.152	0.021888	9.3136
3.153	0.83385	9.4374	3.153	0.37485	8.9067	3.153	0.18513	8.94879	3.153	0.08415	9.11759	3.153	0.022032	9.3141
3.154	0.8393	9.4538	3.154	0.3773	8.9146	3.154	0.18634	8.95294	3.154	0.0847	9.11955	3.154	0.022176	9.3146
3.155	0.84475	9.4702	3.155	0.37975	8.9225	3.155	0.18755	8.95709	3.155	0.08525	9.12151	3.155	0.02232	9.3151
3.156	0.8502	9.4866	3.156	0.3822	8.9304	3.156	0.18876	8.96124	3.156	0.0858	9.12347	3.156	0.022464	9.3156
3.157	0.85565	9.503	3.157	0.38465	8.9383	3.157	0.18997	8.96539	3.157	0.08635	9.12543	3.157	0.022608	9.3161

3.158	0.8611	9.5194	3.158	0.3871	8.9462	3.158	0.19118	8.96954	3.158	0.0869	9.12739	3.158	0.022752	9.3166
3.159	0.86655	9.5358	3.159	0.38955	8.9541	3.159	0.19239	8.97369	3.159	0.08745	9.12935	3.159	0.022896	9.3171
3.16	0.872	9.5522	3.16	0.392	8.962	3.16	0.1936	8.97784	3.16	0.088	9.13131	3.16	0.02304	9.3176
3.161	0.87745	9.5686	3.161	0.39445	8.9699	3.161	0.19481	8.98199	3.161	0.08855	9.13327	3.161	0.023184	9.3181
3.162	0.8829	9.585	3.162	0.3969	8.9778	3.162	0.19602	8.98614	3.162	0.0891	9.13523	3.162	0.023328	9.3186
3.163	0.88835	9.6014	3.163	0.39935	8.9857	3.163	0.19723	8.99029	3.163	0.08965	9.13719	3.163	0.023472	9.3191
3.164	0.8938	9.6178	3.164	0.4018	8.9936	3.164	0.19844	8.99444	3.164	0.0902	9.13915	3.164	0.023616	9.3196
3.165	0.89925	9.6342	3.165	0.40425	9.0015	3.165	0.19965	8.99859	3.165	0.09075	9.14111	3.165	0.02376	9.3201
3.166	0.9047	9.6506	3.166	0.4067	9.0094	3.166	0.20086	9.00274	3.166	0.0913	9.14307	3.166	0.023904	9.3206
3.167	0.91015	9.667	3.167	0.40915	9.0173	3.167	0.20207	9.00689	3.167	0.09185	9.14503	3.167	0.024048	9.3211
3.168	0.9156	9.6834	3.168	0.4116	9.0252	3.168	0.20328	9.01104	3.168	0.0924	9.14699	3.168	0.024192	9.3216
3.169	0.92105	9.6998	3.169	0.41405	9.0331	3.169	0.20449	9.01519	3.169	0.09295	9.14895	3.169	0.024336	9.3221
3.17	0.9265	9.7162	3.17	0.4165	9.041	3.17	0.2057	9.01934	3.17	0.0935	9.15091	3.17	0.02448	9.3226
3.171	0.93195	9.7326	3.171	0.41895	9.0489	3.171	0.20691	9.02349	3.171	0.09405	9.15287	3.171	0.024624	9.3231
3.172	0.9374	9.749	3.172	0.4214	9.0568	3.172	0.20812	9.02764	3.172	0.0946	9.15483	3.172	0.024768	9.3236
3.173	0.94285	9.7654	3.173	0.42385	9.0647	3.173	0.20933	9.03179	3.173	0.09515	9.15679	3.173	0.024912	9.3241
3.174	0.9483	9.7818	3.174	0.4263	9.0726	3.174	0.21054	9.03594	3.174	0.0957	9.15875	3.174	0.025056	9.3246
3.175	0.95375	9.7982	3.175	0.42875	9.0805	3.175	0.21175	9.04009	3.175	0.09625	9.16071	3.175	0.0252	9.3251
3.176	0.9592	9.8146	3.176	0.4312	9.0884	3.176	0.21296	9.04424	3.176	0.0968	9.16267	3.176	0.025344	9.3256
3.177	0.96465	9.831	3.177	0.43365	9.0963	3.177	0.21417	9.04839	3.177	0.09735	9.16463	3.177	0.025488	9.3261
3.178	0.9701	9.8474	3.178	0.4361	9.1042	3.178	0.21538	9.05254	3.178	0.0979	9.16659	3.178	0.025632	9.3266
3.179	0.97555	9.8638	3.179	0.43855	9.1121	3.179	0.21659	9.05669	3.179	0.09845	9.16855	3.179	0.025776	9.3271
3.18	0.981	9.8802	3.18	0.441	9.12	3.18	0.2178	9.06084	3.18	0.099	9.17051	3.18	0.02592	9.3276
3.181	0.98645	9.8966	3.181	0.44345	9.1279	3.181	0.21901	9.06499	3.181	0.09955	9.17247	3.181	0.026064	9.3281
3.182	0.9919	9.913	3.182	0.4459	9.1358	3.182	0.22022	9.06914	3.182	0.1001	9.17443	3.182	0.026208	9.3286
3.183	0.99735	9.9294	3.183	0.44835	9.1437	3.183	0.22143	9.07329	3.183	0.10065	9.17639	3.183	0.026352	9.3291
3.184	1.0028	9.9458	3.184	0.4508	9.1516	3.184	0.22264	9.07744	3.184	0.1012	9.17835	3.184	0.026496	9.3296
3.185	1.00825	9.9622	3.185	0.45325	9.1595	3.185	0.22385	9.08159	3.185	0.10175	9.18031	3.185	0.02664	9.3301
3.186	1.0137	9.9786	3.186	0.4557	9.1674	3.186	0.22506	9.08574	3.186	0.1023	9.18227	3.186	0.026784	9.3306
3.187	1.01915	9.995	3.187	0.45815	9.1753	3.187	0.22627	9.08989	3.187	0.10285	9.18423	3.187	0.026928	9.3311
3.188	1.0246	10.0114	3.188	0.4606	9.1832	3.188	0.22748	9.09404	3.188	0.1034	9.18619	3.188	0.027072	9.3316
3.189	1.03005	10.0278	3.189	0.46305	9.1911	3.189	0.22869	9.09819	3.189	0.10395	9.18815	3.189	0.027216	9.3321
3.19	1.0355	10.0442	3.19	0.4655	9.199	3.19	0.2299	9.10234	3.19	0.1045	9.19011	3.19	0.02736	9.3326
3.191	1.04095	10.0606	3.191	0.46795	9.2069	3.191	0.23111	9.10649	3.191	0.10505	9.19207	3.191	0.027504	9.3331
3.192	1.0464	10.077	3.192	0.4704	9.2148	3.192	0.23232	9.11064	3.192	0.1056	9.19403	3.192	0.027648	9.3336
3.193	1.05185	10.0934	3.193	0.47285	9.2227	3.193	0.23353	9.11479	3.193	0.10615	9.19599	3.193	0.027792	9.3341
3.194	1.0573	10.1098	3.194	0.4753	9.2306	3.194	0.23474	9.11894	3.194	0.1067	9.19795	3.194	0.027936	9.3346
3.195	1.06275	10.1262	3.195	0.47775	9.2385	3.195	0.23595	9.12309	3.195	0.10725	9.19991	3.195	0.02808	9.3351
3.196	1.0682	10.1426	3.196	0.4802	9.2464	3.196	0.23716	9.12724	3.196	0.1078	9.20187	3.196	0.028224	9.3356
3.197	1.07365	10.159	3.197	0.48265	9.2543	3.197	0.23837	9.13139	3.197	0.10835	9.20383	3.197	0.028368	9.3361
3.198	1.0791	10.1754	3.198	0.4851	9.2622	3.198	0.23958	9.13554	3.198	0.1089	9.20579	3.198	0.028512	9.3366
3.199	1.08455	10.1918	3.199	0.48755	9.2701	3.199	0.24079	9.13969	3.199	0.10945	9.20775	3.199	0.028656	9.3371
3.2	1.09	10.2082	3.2	0.49	9.278	3.2	0.242	9.14384	3.2	0.11	9.20971	3.2	0.0288	9.3376
3.201	1.09545	10.2246	3.201	0.49245	9.2859	3.201	0.24321	9.14799	3.201	0.11055	9.21167	3.201	0.028944	9.3381
3.202	1.1009	10.241	3.202	0.4949	9.2938	3.202	0.24442	9.15214	3.202	0.1111	9.21363	3.202	0.029088	9.3386
3.203	1.10635	10.2574	3.203	0.49735	9.3017	3.203	0.24563	9.15629	3.203	0.11165	9.21559	3.203	0.029232	9.3391
3.204	1.1118	10.2738	3.204	0.4998	9.3096	3.204	0.24684	9.16044	3.204	0.1122	9.21755	3.204	0.029376	9.3396
3.205	1.11725	10.2902	3.205	0.50225	9.3175	3.205	0.24805	9.16459	3.205	0.11275	9.21951	3.205	0.02952	9.3401
3.206	1.1227	10.3066	3.206	0.5047	9.3254	3.206	0.24926	9.16874	3.206	0.1133	9.22147	3.206	0.029664	9.3406
3.207	1.12815	10.323	3.207	0.50715	9.3333	3.207	0.25047	9.17289	3.207	0.11385	9.22343	3.207	0.029808	9.3411
3.208	1.1336	10.3394	3.208	0.5096	9.3412	3.208	0.25168	9.17704	3.208	0.1144	9.22539	3.208	0.029952	9.3416
3.209	1.13905	10.3558	3.209	0.51205	9.3491	3.209	0.25289	9.18119	3.209	0.11495	9.22735	3.209	0.030096	9.3421
3.21	1.1445	10.3722	3.21	0.5145	9.357	3.21	0.2541	9.18534	3.21	0.1155	9.22931	3.21	0.03024	9.3426
3.211	1.14995	10.3886	3.211	0.51695	9.3649	3.211	0.25531	9.18949	3.211	0.11605	9.23127	3.211	0.030384	9.3431
3.212	1.1554	10.405	3.212	0.5194	9.3728	3.212	0.25652	9.19364	3.212	0.1166	9.23323	3.212	0.030528	9.3436
3.213	1.16085	10.4214	3.213	0.52185	9.3807	3.213	0.25773	9.19779	3.213	0.11715	9.23519	3.213	0.030672	9.3441
3.214	1.1663	10.4378	3.214	0.5243	9.3886	3.214	0.25894	9.20194	3.214	0.1177	9.23715	3.214	0.030816	9.3446
3.215	1.17175	10.4542	3.215	0.52675	9.3965	3.215	0.26015	9.20609	3.215	0.11825	9.23911	3.215	0.03096	9.3451
3.216	1.1772	10.4706	3.216	0.5292	9.4044	3.216	0.26136	9.21024	3.216	0.1188	9.24107	3.216	0.031104	9.3456
3.217	1.18265	10.487	3.217	0.53165	9.4123	3.217	0.26257	9.21439	3.217	0.11935	9.24303	3.217	0.031248	9.3461
3.218	1.1881	10.5034	3.218	0.5341	9.4202	3.218	0.26378	9.21854	3.218	0.1199	9.24499	3.218	0.031392	9.3466
3.219	1.19355	10.5198	3.219	0.53655	9.4281	3.219	0.26499	9.22269	3.219	0.12045	9.24695	3.219	0.031536	9.3471
3.22	1.199	10.5362	3.22	0.539	9.436	3.22	0.2662	9.22684	3.22	0.121	9.24891	3.22	0.03168	9.3476
3.221	1.20445	10.5526	3.221	0.54145	9.4439	3.221	0.26741	9.23099	3.221	0.12155	9.25087	3.221	0.031824	9.3481
3.222	1.2099	10.569	3.222	0.5439	9.4518	3.222	0.26862	9.23514	3.222	0.1221	9.25283	3.222	0.031968	9.3486
3.223	1.21535	10.5854	3.223	0.54635	9.4597	3.223	0.26983	9.23929	3.223	0.12265	9.25479	3.223	0.032112	9.3491
3.224	1.2208	10.6018	3.224	0.5488	9.4676	3.224	0.27104	9.24344	3.224	0.1232	9.25675	3.224	0.032256	9.3496
3.225	1.22625	10.6182	3.225	0.55125	9.4755	3.225	0.27225	9.24759	3.225	0.12375	9.25871	3.225	0.0324	9.3501
3.226	1.2317	10.6346	3.226	0.5537	9.4834	3.226	0.27346	9.25174	3.226	0.1243	9.26067	3.226	0.032544	9.3506
3.227	1.23715	10.651	3.227	0.55615	9.4913	3.227	0.27467	9.25589	3.227	0.12485	9.26263	3.227	0.032688	9.3511
3.228	1.2426	10.6674	3.228	0.5586	9.4992	3.228	0.27588	9.26004	3.228	0.1254	9.26459	3.228	0.032832	9.3516
3.229	1.24805	10.6838	3.229	0.56105	9.5071	3.229	0.27709	9.26419	3.229	0.12595	9.26655	3.229	0.032976	9.3521
3.23	1.2535	10.7002	3.23	0.5635	9.515	3.23	0.2783	9.26834	3.23	0.1265	9.26851	3.23	0.03312	9.3526
3.231	1.25895	10.7166	3.231	0.56595	9.5229	3.231	0.27951	9.27249	3.231	0.12705	9.27047	3.231	0.033264	9.3531
3.232	1.2644	10.733	3.232	0.5684	9.5308	3.232	0.28072	9.27664	3.232	0.1276	9.27243	3.232	0.033408	9.3536
3.233	1.26985	10.7494	3.233	0.57085	9.5387	3.233	0.28193	9.28079	3.233	0.12815	9.27439	3.233	0.033552	9.3541
3.234	1.2753	10.7658	3.234	0.5733	9.5466	3.234	0.28314	9.28494	3.234	0.1287	9.27635	3.234	0.033696	9.3546
3.235	1.28075	10.7822	3.235	0.57575	9.5545	3.235	0.28435	9.28909	3.235	0.12925	9.27831	3.235	0.03384	9.3551
3.236	1.2862	10.7986	3.236	0.5782	9.5624	3.236	0.28556	9.29324	3.236	0.1298	9.28027	3.236	0.033984	9.3556
3.237	1.29165	10.815	3.237	0.58065	9.5703	3.237	0.28677	9.29739	3.237	0.13035	9.28223	3.237	0.034128	9.3561
3.238	1.2971	10.8314	3.238	0.5831	9.5782	3.238	0.28798	9.30154	3.238	0.1309	9.28419	3.238	0.034272	9.3566
3.239	1.30255	10.8478	3.239	0.58555	9.5861	3.239	0.28919	9.30569	3.239	0.13145	9.28615	3.239	0.034416	9.3571
3.24	1.308	10.8642	3.24	0.588	9.594	3.24	0.2904	9.30984	3.24	0.132	9.28811	3.24	0.03456	9.3576
3.241	1.31345	10.8806	3.241	0.59045	9.6019	3.241	0.29161	9.31399	3.241	0.13255	9.29007	3.241	0.034704	9.3581
3.242	1.3189	10.897	3.242	0.5929	9.6098	3.242	0.29282	9.31814	3.242	0.1331	9.29203	3.242	0.034848	9.3586
3.243	1.32435	10.9134	3.243	0.59535	9.6177	3.243	0.29403	9.32229	3.243	0.13365	9.29399	3.243	0.034992	9.3591
3.244	1.3298	10.9298	3.244	0.5978	9.6256	3.244	0.29524	9.32644	3.244	0.1342	9.29595	3.244	0.035136	9.3596
3.245	1.33525	10.9462	3.245	0.60025	9.6335	3.245	0.29645	9.33059	3.245	0.13475	9.29791	3.245	0.03528	9.3601
3.246	1.3407	10.9626	3.246	0.6027	9.6414	3.246	0.29766	9.33474	3.246	0.1353	9.29987	3.246	0.035424	9.3606
3.247	1.34615	10.979	3.247	0.60515	9.6493	3.247	0.29887	9.33889	3.247	0.13585	9.30183	3.247	0.035568	9.3611
3.248	1.3516	10.9954	3.248	0.6076	9.6572	3.248	0.30008	9.34304	3.248	0.1364	9.30379	3.248	0.035712	9.3616
3.249	1.35705	11.0118	3.249	0.61005	9.6651	3.249	0.30129	9.34719	3.249	0.13695	9.30575	3.249	0.035856	9.3621
3.25	1.3625	11.0282	3.25	0.6125	9.673	3.25	0.3025	9.35134	3.25	0.1375	9.30771	3.25	0.036	9.3626
3.251	1.36795	11.0446	3.251	0.61495	9.6809	3.251	0.30371	9.35549	3.251	0.13805	9.30967	3.251	0.036144	9.3631
3.252	1.3734	11.061	3.252	0.6174	9.6888	3.252	0.30492	9.35964	3.252	0.1386	9.31163	3.252	0.036288	9.3636
3.253	1.37885	11.0774	3.253	0.61985	9.6967	3.253	0.30613	9.36379	3.253	0.13915	9.31359	3.253	0.036432	9.3641
3.254	1.3843	11.0938	3.254	0.6223	9.7046	3.254	0.30734	9.36794	3.254	0.1397	9.31555	3.254	0.036576	9.3646
3.255	1.38975	11.1102	3.255	0.62475	9.7125	3.255	0.30855	9.37209	3.255	0.14025	9.31751	3.255	0.03672	9.3651
3.256	1.3952	11.1266	3.256	0.6272	9.7204	3.256	0.30976	9.37624	3.256	0.1408	9.31947	3.256	0.036864	9.3656
3.257	1.40065	11.143	3.257	0.62965	9.7283	3.257	0.31097	9.38039	3.257	0.14135	9.32143	3.257	0.037008	9.3661
3.258	1.4061	11.1594	3.258	0.6321	9.7362	3.258	0.31218	9.38454	3.258	0.1419	9.32339	3.258	0.037152	9.3666
3.259	1.41155	11.1758	3.259	0.63455	9.7441	3.259	0.31339	9.38869	3.259	0.14245	9.32535	3.259	0.037296	9.3671
3.26	1.417	11.1922	3.26	0.637	9.752	3.26	0.3146	9.39284	3.26	0.143	9.32731	3.26	0.03744	9.3676
3.261	1.42245	11.2086	3.261	0.63945	9.7599	3.261	0.31581	9.39699	3.261	0.14355	9.32927	3.261	0.037584	9.3681
3.262	1.4279	11.225	3.262	0.6419	9.7678	3.262	0.31702	9.40114	3.262	0.1441	9.33123	3.262	0.037728	9.3686
3.263	1.43335	11.2414	3.263	0.64435	9.7757	3.263	0.31823	9.40529	3.263	0.14465	9.33319	3.263	0.037872	9.3691
3.264	1.4388	11.2578	3.264	0.6468	9.7836	3.264	0.31944	9.40944	3.264	0.1452	9.33515	3.264	0.038016	9.3696
3.265	1.44425	11.2742	3.265	0.64925	9.7915	3.265	0.32065	9.41359	3.265	0.14575	9.33711	3.265	0.03816	9.3701
3.266	1.4497	11.2906	3.266	0.6517	9.7994	3.266	0.32186	9.41774	3.266	0.1463	9.33907	3.266	0.038304	9.3706
3.267	1.45515	11.307	3.267	0.65415	9.8073	3.267	0.32307	9.42189	3.267	0.14685	9.34103	3.267	0.038448	9.3711
3.268	1.4606	11.3234	3.268	0.6566	9.8152	3.268	0.32428	9.42604	3.268	0.1474	9.34299	3.268	0.038592	9.3716
3.269	1.46605	11.3398	3.269	0.65905	9.8231	3.269	0.32549	9.43019	3.269	0.14795	9.34495	3.269	0.038736	9.3721
3.27	1.4715	11.3562	3.27	0.6615	9.831	3.27	0.3267	9.43434	3.27	0.1485	9.34691	3.27	0.03888	9.3726
3.271	1.47695	11.3726	3.271	0.66395	9.8389	3.271	0.32791	9.43849	3.271	0.14905	9.34887	3.271	0.039024	9.3731
3.272	1.4824	11.389	3.272	0.6664	9.8468	3.272	0.32912	9.44264	3.272	0.1496	9.35083	3.272	0.039168	9.3736
3.273	1.48785	11.4054	3.273	0.66885	9.8547	3.273	0.33033	9.44679	3.273	0.15015	9.35279	3.273	0.039312	9.3741
3.274	1.4933	11.4218	3.274	0.6713	9.8626	3.274	0.33154	9.45094	3.274	0.1507	9.35475	3.274	0.039456	9.3746
3.275	1.49875	11.4382	3.275	0.67375	9.8705	3.275	0.33275	9.45509	3.275	0.15125	9.35671	3.275	0.0396	9.3751
3.276	1.5042	11.4546	3.276	0.6762	9.8784	3.276	0.33396	9.45924	3.276	0.1518	9.35867	3.276	0.039744	9.3756
3.277	1.50965	11.471	3.277	0.67865	9.8863	3.277	0.33517	9.46339	3.277	0.15235	9.36063	3.277	0.039888	9.3761
3.278	1.5151	11.4874	3.278	0.6811	9.8942	3.278	0.33638	9.46754	3.278	0.1529	9.36259	3.278	0.040032	9.3766
3.279	1.52055	11.5038	3.279	0.68355	9.9021	3.279	0.33759	9.47169	3.279	0.15345	9.36455	3.279	0.040176	9.3771
3.28	1.526	11.5202	3.28	0.686	9.91	3.28	0.3388	9.47584	3.28	0.154	9.36651	3.28	0.04032	9.3776
3.281	1.53145	11.5366	3.281	0.68845	9.9179	3.281	0.34001	9.47999	3.281	0.15455	9.36847	3.281	0.040464	9.3781
3.282	1.5369	11.553	3.282	0.6909	9.9258	3.282	0.34122	9.48414	3.282	0.1551	9.37043	3.282	0.040608	9.3786
3.283	1.54235	11.5694	3.283	0.69335	9.9337	3.283	0.34243	9.48829	3.283	0.15565	9.37239	3.283	0.040752	9.3791
3.284	1.5478	11.5858	3.284	0.6958	9.9416	3.284	0.34364	9.49244	3.284	0.1562	9.37435	3.284	0.040896	9.3796
3.285	1.55325	11.6022	3.285	0.69825	9.9495	3.285	0.34485	9.49659	3.285	0.15675	9.37631	3.285	0.04104	9.3801
3.286	1.5587	11.6186	3.286	0.7007	9.9574	3.286	0.34606	9.50074	3.286	0.1573	9.37827	3.286	0.041184	9.3806
3.287	1.56415	11.635	3.287	0.70315	9.9653	3.287	0.34727	9.50489	3.287	0.15785	9.38023	3.287	0.041328	9.3811
3.288	1.5696	11.6514	3.288	0.7056	9.9732	3.288	0.34848	9.50904	3.288	0.1584	9.38219	3.288	0.041472	9.3816
3.289	1.57505	11.6678	3.289	0.70805	9.9811	3.289	0.34969	9.51319	3.289	0.15895	9.38415	3.289	0.041616	9.3821
3.29	1.5805	11.6842	3.29	0.7105	9.989	3.29	0.3509	9.51734	3.29	0.1595	9.38611	3.29	0.04176	9.3826
3.291	1.58595	11.7006	3.291	0.71295	9.9969	3.291	0.35211	9.52149	3.291	0.16005	9.38807	3.291	0.041904	9.3831
3.292	1.5914	11.717	3.292	0.7154	10.0048	3.292	0.35332	9.52564	3.292	0.1606	9.39003	3.292	0.042048	9.3836
3.293	1.59685	11.7334	3.293	0.71785	10.0127	3.293	0.35453	9.52979	3.293	0.16115	9.39199	3.293	0.042192	9.3841
3.294	1.6023	11.7498	3.294	0.7203	10.0206	3.294	0.35574	9.53394	3.294	0.1617	9.39395	3.294	0.042336	9.3846
3.295	1.60775	11.7662	3.295	0.72275	10.0285	3.295	0.35695	9.53809	3.295	0.16225	9.39591	3.295	0.04248	9.3851
3.296	1.6132	11.7826	3.296	0.7252	10.0364	3.296	0.35816	9.54224	3.296	0.1628	9.39787	3.296	0.042624	9.3856
3.297	1.61865	11.799	3.297	0.72765	10.0443	3.297	0.35937	9.54639	3.297	0.16335	9.39983	3.297	0.042768	9.3861
3.298	1.6241	11.8154	3.298	0.7301	10.0522	3.298	0.36058	9.55054	3.298	0.1639	9.40179	3.298	0.042912	9.3866
3.299	1.62955	11.8318	3.299	0.73255	10.0601	3.299	0.36179	9.55469	3.299	0.16445	9.40375	3.299	0.043056	9.3871
3.3	1.635	11.8482	3.3	0.735	10.068	3.3	0.363	9.55884	3.3	0.165	9.40571	3.3	0.0432	9.3876
3.301	1.64045	11.8646	3.301	0.73745	10.0759	3.301	0.36421	9.56299	3.301	0.16555	9.40767	3.301	0.043344	9.3881
3.302	1.6459	11.881	3.302	0.7399	10.0838	3.302	0.36542	9.56714	3.302	0.1661	9.40963	3.302	0.043488	9.3886
3.303	1.65135	11.8974	3.303	0.74235	10.0917	3.303	0.36663	9.57129	3.303	0.16665	9.41159	3.303	0.043632	9.3891
3.304	1.6568	11.9138	3.304	0.7448	10.0996	3.304	0.36784	9.57544	3.304	0.1672	9.41355	3.304	0.043776	9.3896
3.305	1.66225	11.9302	3.305	0.74725	10.1075	3.305	0.36905	9.57959	3.305	0.16775	9.41551	3.305	0.04392	9.3901
3.306	1.6677	11.9466	3.306	0.7497	10.1154	3.306	0.37026	9.58374	3.306	0.1683	9.41747	3.306	0.044064	9.3906
3.307	1.67315	11.963	3.307	0.75215	10.1233	3.307	0.37147	9.58789	3.307	0.16885	9.41943	3.307	0.044208	9.3911
3.308	1.6786	11.9794	3.308	0.7546	10.1312	3.308	0.37268	9.59204	3.308	0.1694	9.42139	3.308	0.044352	9.3916
3.309	1.68405	11.9958	3.309	0.75705	10.1391	3.309	0.37389	9.59619	3.309	0.16995	9.42335	3.309	0.044496	9.3921
3.31	1.6895	12.0122	3.31	0.7595	10.147	3.31	0.3751	9.60034	3.31	0.1705	9.42531	3.31	0.04464	9.3926
3.311	1.69495	12.0286	3.311	0.76195	10.1549	3.311	0.37631	9.60449	3.311	0.17105	9.42727	3.311	0.044784	9.3931
3.312	1.7004	12.045	3.312	0.7644	10.1628	3.312	0.37752	9.60864	3.312	0.1716	9.42923	3.312	0.044928	9.3936
3.313	1.70585	12.0614	3.313	0.76685	10.1707	3.313	0.37873	9.61279	3.313	0.17215	9.43119	3.313	0.045072	9.3941
3.314	1.7113	12.0778	3.314	0.7693	10.1786	3.314	0.37994	9.61694	3.314	0.1727	9.43315	3.314	0.045216	9.3946
3.315	1.71675	12.0942	3.315	0.77175	10.1865	3.315	0.38115	9.62109	3.315	0.17325	9.43511	3.315	0.04536	9.3951
3.316	1.7222	12.1106	3.316	0.7742	10.1944	3.316	0.38236	9.62524	3.316	0.1738	9.43707	3.316	0.045504	9.3956
3.317	1.72765	12.127	3.317	0.77665	10.2023	3.317	0.38357	9.62939	3.317	0.17435	9.43903	3.317	0.045648	9.3961

3.318	1.7331	12.1434	3.318	0.7791	10.2102	3.318	0.38478	9.63354	3.318	0.1749	9.44099	3.318	0.045792	9.3966
3.319	1.73855	12.1598	3.319	0.78155	10.2181	3.319	0.38599	9.63769	3.319	0.17545	9.44295	3.319	0.045936	9.3971
3.32	1.744	12.1762	3.32	0.784	10.226	3.32	0.3872	9.64184	3.32	0.176	9.44491	3.32	0.04608	9.3976
3.321	1.74945	12.1926	3.321	0.78645	10.2339	3.321	0.38841	9.64599	3.321	0.17655	9.44687	3.321	0.046224	9.3981
3.322	1.7549	12.209	3.322	0.7889	10.2418	3.322	0.38962	9.65014	3.322	0.1771	9.44883	3.322	0.046368	9.3986
3.323	1.76035	12.2254	3.323	0.79135	10.2497	3.323	0.39083	9.65429	3.323	0.17765	9.45079	3.323	0.046512	9.3991
3.324	1.7658	12.2418	3.324	0.7938	10.2576	3.324	0.39204	9.65844	3.324	0.1782	9.45275	3.324	0.046656	9.3996
3.325	1.77125	12.2582	3.325	0.79625	10.2655	3.325	0.39325	9.66259	3.325	0.17875	9.45471	3.325	0.0468	9.4001
3.326	1.7767	12.2746	3.326	0.7987	10.2734	3.326	0.39446	9.66674	3.326	0.1793	9.45667	3.326	0.046944	9.4006
3.327	1.78215	12.291	3.327	0.80115	10.2813	3.327	0.39567	9.67089	3.327	0.17985	9.45863	3.327	0.047088	9.4011
3.328	1.7876	12.3074	3.328	0.8036	10.2892	3.328	0.39688	9.67504	3.328	0.1804	9.46059	3.328	0.047232	9.4016
3.329	1.79305	12.3238	3.329	0.80605	10.2971	3.329	0.39809	9.67919	3.329	0.18095	9.46255	3.329	0.047376	9.4021
3.33	1.7985	12.3402	3.33	0.8085	10.305	3.33	0.3993	9.68334	3.33	0.1815	9.46451	3.33	0.04752	9.4026
3.331	1.80395	12.3566	3.331	0.81095	10.3129	3.331	0.40051	9.68749	3.331	0.18205	9.46647	3.331	0.047664	9.4031
3.332	1.8094	12.373	3.332	0.8134	10.3208	3.332	0.40172	9.69164	3.332	0.1826	9.46843	3.332	0.047808	9.4036
3.333	1.81485	12.3894	3.333	0.81585	10.3287	3.333	0.40293	9.69579	3.333	0.18315	9.47039	3.333	0.047952	9.4041
3.334	1.8203	12.4058	3.334	0.8183	10.3366	3.334	0.40414	9.69994	3.334	0.1837	9.47235	3.334	0.048096	9.4046
3.335	1.82575	12.4222	3.335	0.82075	10.3445	3.335	0.40535	9.70409	3.335	0.18425	9.47431	3.335	0.04824	9.4051
3.336	1.8312	12.4386	3.336	0.8232	10.3524	3.336	0.40656	9.70824	3.336	0.1848	9.47627	3.336	0.048384	9.4056
3.337	1.83665	12.455	3.337	0.82565	10.3603	3.337	0.40777	9.71239	3.337	0.18535	9.47823	3.337	0.048528	9.4061
3.338	1.8421	12.4714	3.338	0.8281	10.3682	3.338	0.40898	9.71654	3.338	0.1859	9.48019	3.338	0.048672	9.4066
3.339	1.84755	12.4878	3.339	0.83055	10.3761	3.339	0.41019	9.72069	3.339	0.18645	9.48215	3.339	0.048816	9.4071
3.34	1.853	12.5042	3.34	0.833	10.384	3.34	0.4114	9.72484	3.34	0.187	9.48411	3.34	0.04896	9.4076
3.341	1.85845	12.5206	3.341	0.83545	10.3919	3.341	0.41261	9.72899	3.341	0.18755	9.48607	3.341	0.049104	9.4081
3.342	1.8639	12.537	3.342	0.8379	10.3998	3.342	0.41382	9.73314	3.342	0.1881	9.48803	3.342	0.049248	9.4086
3.343	1.86935	12.5534	3.343	0.84035	10.4077	3.343	0.41503	9.73729	3.343	0.18865	9.48999	3.343	0.049392	9.4091
3.344	1.8748	12.5698	3.344	0.8428	10.4156	3.344	0.41624	9.74144	3.344	0.1892	9.49195	3.344	0.049536	9.4096
3.345	1.88025	12.5862	3.345	0.84525	10.4235	3.345	0.41745	9.74559	3.345	0.18975	9.49391	3.345	0.04968	9.4101
3.346	1.8857	12.6026	3.346	0.8477	10.4314	3.346	0.41866	9.74974	3.346	0.1903	9.49587	3.346	0.049824	9.4106
3.347	1.89115	12.619	3.347	0.85015	10.4393	3.347	0.41987	9.75389	3.347	0.19085	9.49783	3.347	0.049968	9.4111
3.348	1.8966	12.6354	3.348	0.8526	10.4472	3.348	0.42108	9.75804	3.348	0.1914	9.49979	3.348	0.050112	9.4116
3.349	1.90205	12.6518	3.349	0.85505	10.4551	3.349	0.42229	9.76219	3.349	0.19195	9.50175	3.349	0.050256	9.4121
3.35	1.9075	12.6682	3.35	0.8575	10.463	3.35	0.4235	9.76634	3.35	0.1925	9.50371	3.35	0.0504	9.4126
3.351	1.91295	12.6846	3.351	0.85995	10.4709	3.351	0.42471	9.77049	3.351	0.19305	9.50567	3.351	0.050544	9.4131
3.352	1.9184	12.701	3.352	0.8624	10.4788	3.352	0.42592	9.77464	3.352	0.1936	9.50763	3.352	0.050688	9.4136
3.353	1.92385	12.7174	3.353	0.86485	10.4867	3.353	0.42713	9.77879	3.353	0.19415	9.50959	3.353	0.050832	9.4141
3.354	1.9293	12.7338	3.354	0.8673	10.4946	3.354	0.42834	9.78294	3.354	0.1947	9.51155	3.354	0.050976	9.4146
3.355	1.93475	12.7502	3.355	0.86975	10.5025	3.355	0.42955	9.78709	3.355	0.19525	9.51351	3.355	0.05112	9.4151
3.356	1.9402	12.7666	3.356	0.8722	10.5104	3.356	0.43076	9.79124	3.356	0.1958	9.51547	3.356	0.051264	9.4156
3.357	1.94565	12.783	3.357	0.87465	10.5183	3.357	0.43197	9.79539	3.357	0.19635	9.51743	3.357	0.051408	9.4161
3.358	1.9511	12.7994	3.358	0.8771	10.5262	3.358	0.43318	9.79954	3.358	0.1969	9.51939	3.358	0.051552	9.4166
3.359	1.95655	12.8158	3.359	0.87955	10.5341	3.359	0.43439	9.80369	3.359	0.19745	9.52135	3.359	0.051696	9.4171
3.36	1.962	12.8322	3.36	0.882	10.542	3.36	0.4356	9.80784	3.36	0.198	9.52331	3.36	0.05184	9.4176
3.361	1.96745	12.8486	3.361	0.88445	10.5499	3.361	0.43681	9.81199	3.361	0.19855	9.52527	3.361	0.051984	9.4181
3.362	1.9729	12.865	3.362	0.8869	10.5578	3.362	0.43802	9.81614	3.362	0.1991	9.52723	3.362	0.052128	9.4186
3.363	1.97835	12.8814	3.363	0.88935	10.5657	3.363	0.43923	9.82029	3.363	0.19965	9.52919	3.363	0.052272	9.4191
3.364	1.9838	12.8978	3.364	0.8918	10.5736	3.364	0.44044	9.82444	3.364	0.2002	9.53115	3.364	0.052416	9.4196
3.365	1.98925	12.9142	3.365	0.89425	10.5815	3.365	0.44165	9.82859	3.365	0.20075	9.53311	3.365	0.05256	9.4201
3.366	1.9947	12.9306	3.366	0.8967	10.5894	3.366	0.44286	9.83274	3.366	0.2013	9.53507	3.366	0.052704	9.4206
3.367	2.00015	12.947	3.367	0.89915	10.5973	3.367	0.44407	9.83689	3.367	0.20185	9.53703	3.367	0.052848	9.4211
3.368	2.0056	12.9634	3.368	0.9016	10.6052	3.368	0.44528	9.84104	3.368	0.2024	9.53899	3.368	0.052992	9.4216
3.369	2.01105	12.9798	3.369	0.90405	10.6131	3.369	0.44649	9.84519	3.369	0.20295	9.54095	3.369	0.053136	9.4221
3.37	2.0165	12.9962	3.37	0.9065	10.621	3.37	0.4477	9.84934	3.37	0.2035	9.54291	3.37	0.05328	9.4226
3.371	2.02195	13.0126	3.371	0.90895	10.6289	3.371	0.44891	9.85349	3.371	0.20405	9.54487	3.371	0.053424	9.4231
3.372	2.0274	13.029	3.372	0.9114	10.6368	3.372	0.45012	9.85764	3.372	0.2046	9.54683	3.372	0.053568	9.4236
3.373	2.03285	13.0454	3.373	0.91385	10.6447	3.373	0.45133	9.86179	3.373	0.20515	9.54879	3.373	0.053712	9.4241
3.374	2.0383	13.0618	3.374	0.9163	10.6526	3.374	0.45254	9.86594	3.374	0.2057	9.55075	3.374	0.053856	9.4246
3.375	2.04375	13.0782	3.375	0.91875	10.6605	3.375	0.45375	9.87009	3.375	0.20625	9.55271	3.375	0.054	9.4251
3.376	2.0492	13.0946	3.376	0.9212	10.6684	3.376	0.45496	9.87424	3.376	0.2068	9.55467	3.376	0.054144	9.4256
3.377	2.05465	13.111	3.377	0.92365	10.6763	3.377	0.45617	9.87839	3.377	0.20735	9.55663	3.377	0.054288	9.4261
3.378	2.0601	13.1274	3.378	0.9261	10.6842	3.378	0.45738	9.88254	3.378	0.2079	9.55859	3.378	0.054432	9.4266
3.379	2.06555	13.1438	3.379	0.92855	10.6921	3.379	0.45859	9.88669	3.379	0.20845	9.56055	3.379	0.054576	9.4271
3.38	2.071	13.1602	3.38	0.931	10.7	3.38	0.4598	9.89084	3.38	0.209	9.56251	3.38	0.05472	9.4276
3.381	2.07645	13.1766	3.381	0.93345	10.7079	3.381	0.46101	9.89499	3.381	0.20955	9.56447	3.381	0.054864	9.4281
3.382	2.0819	13.193	3.382	0.9359	10.7158	3.382	0.46222	9.89914	3.382	0.2101	9.56643	3.382	0.055008	9.4286
3.383	2.08735	13.2094	3.383	0.93835	10.7237	3.383	0.46343	9.90329	3.383	0.21065	9.56839	3.383	0.055152	9.4291
3.384	2.0928	13.2258	3.384	0.9408	10.7316	3.384	0.46464	9.90744	3.384	0.2112	9.57035	3.384	0.055296	9.4296
3.385	2.09825	13.2422	3.385	0.94325	10.7395	3.385	0.46585	9.91159	3.385	0.21175	9.57231	3.385	0.05544	9.4301
3.386	2.1037	13.2586	3.386	0.9457	10.7474	3.386	0.46706	9.91574	3.386	0.2123	9.57427	3.386	0.055584	9.4306
3.387	2.10915	13.275	3.387	0.94815	10.7553	3.387	0.46827	9.91989	3.387	0.21285	9.57623	3.387	0.055728	9.4311
3.388	2.1146	13.2914	3.388	0.9506	10.7632	3.388	0.46948	9.92404	3.388	0.2134	9.57819	3.388	0.055872	9.4316
3.389	2.12005	13.3078	3.389	0.95305	10.7711	3.389	0.47069	9.92819	3.389	0.21395	9.58015	3.389	0.056016	9.4321
3.39	2.1255	13.3242	3.39	0.9555	10.779	3.39	0.4719	9.93234	3.39	0.2145	9.58211	3.39	0.05616	9.4326
3.391	2.13095	13.3406	3.391	0.95795	10.7869	3.391	0.47311	9.93649	3.391	0.21505	9.58407	3.391	0.056304	9.4331
3.392	2.1364	13.357	3.392	0.9604	10.7948	3.392	0.47432	9.94064	3.392	0.2156	9.58603	3.392	0.056448	9.4336
3.393	2.14185	13.3734	3.393	0.96285	10.8027	3.393	0.47553	9.94479	3.393	0.21615	9.58799	3.393	0.056592	9.4341
3.394	2.1473	13.3898	3.394	0.9653	10.8106	3.394	0.47674	9.94894	3.394	0.2167	9.58995	3.394	0.056736	9.4346
3.395	2.15275	13.4062	3.395	0.96775	10.8185	3.395	0.47795	9.95309	3.395	0.21725	9.59191	3.395	0.05688	9.4351
3.396	2.1582	13.4226	3.396	0.9702	10.8264	3.396	0.47916	9.95724	3.396	0.2178	9.59387	3.396	0.057024	9.4356
3.397	2.16365	13.439	3.397	0.97265	10.8343	3.397	0.48037	9.96139	3.397	0.21835	9.59583	3.397	0.057168	9.4361
3.398	2.1691	13.4554	3.398	0.9751	10.8422	3.398	0.48158	9.96554	3.398	0.2189	9.59779	3.398	0.057312	9.4366
3.399	2.17455	13.4718	3.399	0.97755	10.8501	3.399	0.48279	9.96969	3.399	0.21945	9.59975	3.399	0.057456	9.4371
3.4	2.18	13.4882	3.4	0.98	10.858	3.4	0.484	9.97384	3.4	0.22	9.60171	3.4	0.0576	9.4376
3.401	2.18545	13.5046	3.401	0.98245	10.8659	3.401	0.48521	9.97799	3.401	0.22055	9.60367	3.401	0.057744	9.4381
3.402	2.1909	13.521	3.402	0.9849	10.8738	3.402	0.48642	9.98214	3.402	0.2211	9.60563	3.402	0.057888	9.4386
3.403	2.19635	13.5374	3.403	0.98735	10.8817	3.403	0.48763	9.98629	3.403	0.22165	9.60759	3.403	0.058032	9.4391
3.404	2.2018	13.5538	3.404	0.9898	10.8896	3.404	0.48884	9.99044	3.404	0.2222	9.60955	3.404	0.058176	9.4396
3.405	2.20725	13.5702	3.405	0.99225	10.8975	3.405	0.49005	9.99459	3.405	0.22275	9.61151	3.405	0.05832	9.4401
3.406	2.2127	13.5866	3.406	0.9947	10.9054	3.406	0.49126	9.99874	3.406	0.2233	9.61347	3.406	0.058464	9.4406
3.407	2.21815	13.603	3.407	0.99715	10.9133	3.407	0.49247	10.00289	3.407	0.22385	9.61543	3.407	0.058608	9.4411
3.408	2.2236	13.6194	3.408	0.9996	10.9212	3.408	0.49368	10.00704	3.408	0.2244	9.61739	3.408	0.058752	9.4416
3.409	2.22905	13.6358	3.409	1.00205	10.9291	3.409	0.49489	10.01119	3.409	0.22495	9.61935	3.409	0.058896	9.4421
3.41	2.2345	13.6522	3.41	1.0045	10.937	3.41	0.4961	10.01534	3.41	0.2255	9.62131	3.41	0.05904	9.4426
3.411	2.23995	13.6686	3.411	1.00695	10.9449	3.411	0.49731	10.01949	3.411	0.22605	9.62327	3.411	0.059184	9.4431
3.412	2.2454	13.685	3.412	1.0094	10.9528	3.412	0.49852	10.02364	3.412	0.2266	9.62523	3.412	0.059328	9.4436
3.413	2.25085	13.7014	3.413	1.01185	10.9607	3.413	0.49973	10.02779	3.413	0.22715	9.62719	3.413	0.059472	9.4441
3.414	2.2563	13.7178	3.414	1.0143	10.9686	3.414	0.50094	10.03194	3.414	0.2277	9.62915	3.414	0.059616	9.4446
3.415	2.26175	13.7342	3.415	1.01675	10.9765	3.415	0.50215	10.03609	3.415	0.22825	9.63111	3.415	0.05976	9.4451
3.416	2.2672	13.7506	3.416	1.0192	10.9844	3.416	0.50336	10.04024	3.416	0.2288	9.63307	3.416	0.059904	9.4456
3.417	2.27265	13.767	3.417	1.02165	10.9923	3.417	0.50457	10.04439	3.417	0.22935	9.63503	3.417	0.060048	9.4461
3.418	2.2781	13.7834	3.418	1.0241	11.0002	3.418	0.50578	10.04854	3.418	0.2299	9.63699	3.418	0.060192	9.4466
3.419	2.28355	13.7998	3.419	1.02655	11.0081	3.419	0.50699	10.05269	3.419	0.23045	9.63895	3.419	0.060336	9.4471
3.42	2.289	13.8162	3.42	1.029	11.016	3.42	0.5082	10.05684	3.42	0.231	9.64091	3.42	0.06048	9.4476
3.421	2.29445	13.8326	3.421	1.03145	11.0239	3.421	0.50941	10.06099	3.421	0.23155	9.64287	3.421	0.060624	9.4481
3.422	2.2999	13.849	3.422	1.0339	11.0318	3.422	0.51062	10.06514	3.422	0.2321	9.64483	3.422	0.060768	9.4486
3.423	2.30535	13.8654	3.423	1.03635	11.0397	3.423	0.51183	10.06929	3.423	0.23265	9.64679	3.423	0.060912	9.4491
3.424	2.3108	13.8818	3.424	1.0388	11.0476	3.424	0.51304	10.07344	3.424	0.2332	9.64875	3.424	0.061056	9.4496
3.425	2.31625	13.8982	3.425	1.04125	11.0555	3.425	0.51425	10.07759	3.425	0.23375	9.65071	3.425	0.0612	9.4501
3.426	2.3217	13.9146	3.426	1.0437	11.0634	3.426	0.51546	10.08174	3.426	0.2343	9.65267	3.426	0.061344	9.4506
3.427	2.32715	13.931	3.427	1.04615	11.0713	3.427	0.51667	10.08589	3.427	0.23485	9.65463	3.427	0.061488	9.4511
3.428	2.3326	13.9474	3.428	1.0486	11.0792	3.428	0.51788	10.09004	3.428	0.2354	9.65659	3.428	0.061632	9.4516
3.429	2.33805	13.9638	3.429	1.05105	11.0871	3.429	0.51909	10.09419	3.429	0.23595	9.65855	3.429	0.061776	9.4521
3.43	2.3435	13.9802	3.43	1.0535	11.095	3.43	0.5203	10.09834	3.43	0.2365	9.66051	3.43	0.06192	9.4526
3.431	2.34895	13.9966	3.431	1.05595	11.1029	3.431	0.52151	10.10249	3.431	0.23705	9.66247	3.431	0.062064	9.4531
3.432	2.3544	14.013	3.432	1.0584	11.1108	3.432	0.52272	10.10664	3.432	0.2376	9.66443	3.432	0.062208	9.4536
3.433	2.35985	14.0294	3.433	1.06085	11.1187	3.433	0.52393	10.11079	3.433	0.23815	9.66639	3.433	0.062352	9.4541
3.434	2.3653	14.0458	3.434	1.0633	11.1266	3.434	0.52514	10.11494	3.434	0.2387	9.66835	3.434	0.062496	9.4546
3.435	2.37075	14.0622	3.435	1.06575	11.1345	3.435	0.52635	10.11909	3.435	0.23925	9.67031	3.435	0.06264	9.4551
3.436	2.3762	14.0786	3.436	1.0682	11.1424	3.436	0.52756	10.12324	3.436	0.2398	9.67227	3.436	0.062784	9.4556
3.437	2.38165	14.095	3.437	1.07065	11.1503	3.437	0.52877	10.12739	3.437	0.24035	9.67423	3.437	0.062928	9.4561
3.438	2.3871	14.1114	3.438	1.0731	11.1582	3.438	0.52998	10.13154	3.438	0.2409	9.67619	3.438	0.063072	9.4566
3.439	2.39255	14.1278	3.439	1.07555	11.1661	3.439	0.53119	10.13569	3.439	0.24145	9.67815	3.439	0.063216	9.4571
3.44	2.398	14.1442	3.44	1.078	11.174	3.44	0.5324	10.13984	3.44	0.242	9.68011	3.44	0.06336	9.4576
3.441	2.40345	14.1606	3.441	1.08045	11.1819	3.441	0.53361	10.14399	3.441	0.24255	9.68207	3.441	0.063504	9.4581
3.442	2.4089	14.177	3.442	1.0829	11.1898	3.442	0.53482	10.14814	3.442	0.2431	9.68403	3.442	0.063648	9.4586
3.443	2.41435	14.1934	3.443	1.08535	11.1977	3.443	0.53603	10.15229	3.443	0.24365	9.68599	3.443	0.063792	9.4591
3.444	2.4198	14.2098	3.444	1.0878	11.2056	3.444	0.53724	10.15644	3.444	0.2442	9.68795	3.444	0.063936	9.4596
3.445	2.42525	14.2262	3.445	1.09025	11.2135	3.445	0.53845	10.16059	3.445	0.24475	9.68991	3.445	0.06408	9.4601
3.446	2.4307	14.2426	3.446	1.0927	11.2214	3.446	0.53966	10.16474	3.446	0.2453	9.69187	3.446	0.064224	9.4606
3.447	2.43615	14.259	3.447	1.09515	11.2293	3.447	0.54087	10.16889	3.447	0.24585	9.69383	3.447	0.064368	9.4611
3.448	2.4416	14.2754	3.448	1.0976	11.2372	3.448	0.54208	10.17304	3.448	0.2464	9.69579	3.448	0.064512	9.4616
3.449	2.44705	14.2918	3.449	1.10005	11.2451	3.449	0.54329	10.17719	3.449	0.24695	9.69775	3.449	0.064656	9.4621
3.45	2.4525	14.3082	3.45	1.1025	11.253	3.45	0.5445	10.18134	3.45	0.2475	9.69971	3.45	0.0648	9.4626
3.451	2.45795	14.3246	3.451	1.10495	11.2609	3.451	0.54571	10.18549	3.451	0.24805	9.70167	3.451	0.064944	9.4631
3.452	2.4634	14.341	3.452	1.1074	11.2688	3.452	0.54692	10.18964	3.452	0.2486	9.70363	3.452	0.065088	9.4636
3.453	2.46885	14.3574	3.453	1.10985	11.2767	3.453	0.54813	10.19379	3.453	0.24915	9.70559	3.453	0.065232	9.4641
3.454	2.4743	14.3738	3.454	1.1123	11.2846	3.454	0.54934	10.19794	3.454	0.2497	9.70755	3.454	0.065376	9.4646
3.455	2.47975	14.3902	3.455	1.11475	11.2925	3.455	0.55055	10.20209	3.455	0.25025	9.70951	3.455	0.06552	9.4651
3.456	2.4852	14.4066	3.456	1.1172	11.3004	3.456	0.55176	10.20624	3.456	0.2508	9.71147	3.456	0.065664	9.4656
3.457	2.49065	14.423	3.457	1.11965	11.3083	3.457	0.55297	10.21039	3.457	0.25135	9.71343	3.457	0.065808	9.4661
3.458	2.4961	14.4394	3.458	1.1221	11.3162	3.458	0.55418	10.21454	3.458	0.2519	9.71539	3.458	0.065952	9.4666
3.459	2.50155	14.4558	3.459	1.12455	11.3241	3.459	0.55539	10.21869	3.459	0.25245	9.71735	3.459	0.066096	9.4671
3.46	2.507	14.4722	3.46	1.127	11.332	3.46	0.5566	10.22284	3.46	0.253	9.71931	3.46	0.06624	9.4676
3.461	2.51245	14.4886	3.461	1.12945	11.3399	3.461	0.55781	10.22699	3.461	0.25355	9.72127	3.461	0.066384	9.4681
3.462	2.5179	14.505	3.462	1.1319	11.3478	3.462	0.55902	10.23114	3.462	0.2541	9.72323	3.462	0.066528	9.4686
3.463	2.52335	14.5214	3.463	1.13435	11.3557	3.463	0.56023	10.23529	3.463	0.25465	9.72519	3.463	0.066672	9.4691
3.464	2.5288	14.5378	3.464	1.1368	11.3636	3.464	0.56144	10.23944	3.464	0.2552	9.72715	3.464	0.066816	9.4696
3.465	2.53425	14.5542	3.465	1.13925	11.3715	3.465	0.56265	10.24359	3.465	0.25575	9.72911	3.465	0.06696	9.4701
3.466	2.5397	14.5706	3.466	1.1417	11.3794	3.466	0.56386	10.24774	3.466	0.2563	9.73107	3.466	0.067104	9.4706
3.467	2.54515	14.587	3.467	1.14415	11.3873	3.467	0.56507	10.25189	3.467	0.25685	9.73303	3.467	0.067248	9.4711
3.468	2.5506	14.6034	3.468	1.1466	11.3952	3.468	0.56628	10.25604	3.468	0.2574	9.73499	3.468	0.067392	9.4716
3.469	2.55605	14.6198	3.469	1.14905	11.4031	3.469	0.56749	10.26019	3.469	0.25795	9.73695	3.469	0.067536	9.4721
3.47	2.5615	14.6362	3.47	1.1515	11.411	3.47	0.5687	10.26434	3.47	0.2585	9.73891	3.47	0.06768	9.4726
3.471	2.56695	14.6526	3.471	1.15395	11.4189	3.471	0.56991	10.26849	3.471	0.25905	9.74087	3.471	0.067824	9.4731
3.472	2.5724	14.669	3.472	1.1564	11.4268	3.472	0.57112	10.27264	3.472	0.2596	9.74283	3.472	0.067968	9.4736
3.473	2.57785	14.6854	3.473	1.15885	11.4347	3.473	0.57233	10.27679	3.473	0.26015	9.74479	3.473	0.068112	9.4741
3.474	2.5833	14.7018	3.474	1.1613	11.4426	3.474	0.57354	10.28094	3.474	0.2607	9.74675	3.474	0.068256	9.4746
3.475	2.58875	14.7182	3.475	1.16375	11.4505	3.475	0.57475	10.28509	3.475	0.26125	9.74871	3.475	0.0684	9.4751
3.476	2.5942	14.7346	3.476	1.1662	11.4584	3.476	0.57596	10.28924	3.476	0.2618	9.75067	3.476	0.068544	9.4756
3.477	2.59965	14.751	3.477	1.16865	11.4663	3.477	0.57717	10.29339	3.477	0.26235	9.75263	3.477	0.068688	9.4761

3.478	2.6051	14.7674	3.478	1.1711	11.4742	3.478	0.57838	10.29754	3.478	0.2629	9.75459	3.478	0.068832	9.4766
3.479	2.61055	14.7838	3.479	1.17355	11.4821	3.479	0.57959	10.30169	3.479	0.26345	9.75655	3.479	0.068976	9.4771
3.48	2.616	14.8002	3.48	1.176	11.49	3.48	0.5808	10.30584	3.48	0.264	9.75851	3.48	0.06912	9.4776
3.481	2.62145	14.8166	3.481	1.17845	11.4979	3.481	0.58201	10.30999	3.481	0.26455	9.76047	3.481	0.069264	9.4781
3.482	2.6269	14.833	3.482	1.1809	11.5058	3.482	0.58322	10.31414	3.482	0.2651	9.76243	3.482	0.069408	9.4786
3.483	2.63235	14.8494	3.483	1.18335	11.5137	3.483	0.58443	10.31829	3.483	0.26565	9.76439	3.483	0.069552	9.4791
3.484	2.6378	14.8658	3.484	1.1858	11.5216	3.484	0.58564	10.32244	3.484	0.2662	9.76635	3.484	0.069696	9.4796
3.485	2.64325	14.8822	3.485	1.18825	11.5295	3.485	0.58685	10.32659	3.485	0.26675	9.76831	3.485	0.06984	9.4801
3.486	2.6487	14.8986	3.486	1.1907	11.5374	3.486	0.58806	10.33074	3.486	0.2673	9.77027	3.486	0.069984	9.4806
3.487	2.65415	14.915	3.487	1.19315	11.5453	3.487	0.58927	10.33489	3.487	0.26785	9.77223	3.487	0.070128	9.4811
3.488	2.6596	14.9314	3.488	1.1956	11.5532	3.488	0.59048	10.33904	3.488	0.2684	9.77419	3.488	0.070272	9.4816
3.489	2.66505	14.9478	3.489	1.19805	11.5611	3.489	0.59169	10.34319	3.489	0.26895	9.77615	3.489	0.070416	9.4821
3.49	2.6705	14.9642	3.49	1.2005	11.569	3.49	0.5929	10.34734	3.49	0.2695	9.77811	3.49	0.07056	9.4826
3.491	2.67595	14.9806	3.491	1.20295	11.5769	3.491	0.59411	10.35149	3.491	0.27005	9.78007	3.491	0.070704	9.4831
3.492	2.6814	14.997	3.492	1.2054	11.5848	3.492	0.59532	10.35564	3.492	0.2706	9.78203	3.492	0.070848	9.4836
3.493	2.68685	15.0134	3.493	1.20785	11.5927	3.493	0.59653	10.35979	3.493	0.27115	9.78399	3.493	0.070992	9.4841
3.494	2.6923	15.0298	3.494	1.2103	11.6006	3.494	0.59774	10.36394	3.494	0.2717	9.78595	3.494	0.071136	9.4846
3.495	2.69775	15.0462	3.495	1.21275	11.6085	3.495	0.59895	10.36809	3.495	0.27225	9.78791	3.495	0.07128	9.4851
3.496	2.7032	15.0626	3.496	1.2152	11.6164	3.496	0.60016	10.37224	3.496	0.2728	9.78987	3.496	0.071424	9.4856
3.497	2.70865	15.079	3.497	1.21765	11.6243	3.497	0.60137	10.37639	3.497	0.27335	9.79183	3.497	0.071568	9.4861
3.498	2.7141	15.0954	3.498	1.2201	11.6322	3.498	0.60258	10.38054	3.498	0.2739	9.79379	3.498	0.071712	9.4866
3.499	2.71955	15.1118	3.499	1.22255	11.6401	3.499	0.60379	10.38469	3.499	0.27445	9.79575	3.499	0.071856	9.4871
3.5	2.725	15.1282	3.5	1.225	11.648	3.5	0.605	10.38884	3.5	0.275	9.79771	3.5	0.072	9.4876
3.501	2.73045	15.1446	3.501	1.22745	11.6559	3.501	0.60621	10.39299	3.501	0.27555	9.79967	3.501	0.072144	9.4881
3.502	2.7359	15.161	3.502	1.2299	11.6638	3.502	0.60742	10.39714	3.502	0.2761	9.80163	3.502	0.072288	9.4886
3.503	2.74537	15.17141	3.503	1.23235	11.6717	3.503	0.60863	10.40129	3.503	0.27665	9.80359	3.503	0.072432	9.4891
3.504	2.74892	15.21357	3.504	1.2348	11.6796	3.504	0.60984	10.40544	3.504	0.2772	9.80555	3.504	0.072576	9.4896
3.505	2.75249	15.25572	3.505	1.23725	11.6875	3.505	0.61105	10.40959	3.505	0.27775	9.80751	3.505	0.07272	9.4901
3.506	2.75606	15.29788	3.506	1.2397	11.6954	3.506	0.61226	10.41374	3.506	0.2783	9.80947	3.506	0.072864	9.4906
3.507	2.75964	15.34002	3.507	1.24215	11.7033	3.507	0.61347	10.41789	3.507	0.27885	9.81143	3.507	0.073008	9.4911
3.508	2.76323	15.38217	3.508	1.2446	11.7112	3.508	0.61468	10.42204	3.508	0.2794	9.81339	3.508	0.073152	9.4916
3.509	2.76683	15.42431	3.509	1.24705	11.7191	3.509	0.61589	10.42619	3.509	0.27995	9.81535	3.509	0.073296	9.4921
3.51	2.77045	15.46645	3.51	1.2495	11.727	3.51	0.6171	10.43034	3.51	0.2805	9.81731	3.51	0.07344	9.4926
3.511	2.77407	15.50859	3.511	1.25195	11.7349	3.511	0.61831	10.43449	3.511	0.28105	9.81927	3.511	0.073584	9.4931
3.512	2.7777	15.55072	3.512	1.2544	11.7428	3.512	0.61952	10.43864	3.512	0.2816	9.82123	3.512	0.073728	9.4936
3.513	2.78134	15.59285	3.513	1.25685	11.7507	3.513	0.62073	10.44279	3.513	0.28215	9.82319	3.513	0.073872	9.4941
3.514	2.78499	15.63498	3.514	1.2593	11.7586	3.514	0.62194	10.44694	3.514	0.2827	9.82515	3.514	0.074016	9.4946
3.515	2.78866	15.6771	3.515	1.26175	11.7665	3.515	0.62315	10.45109	3.515	0.28325	9.82711	3.515	0.07416	9.4951
3.516	2.79233	15.71922	3.516	1.2642	11.7744	3.516	0.62436	10.45524	3.516	0.2838	9.82907	3.516	0.074304	9.4956
3.517	2.79601	15.76134	3.517	1.26665	11.7823	3.517	0.62557	10.45939	3.517	0.28435	9.83103	3.517	0.074448	9.4961
3.518	2.7997	15.80346	3.518	1.2691	11.7902	3.518	0.62678	10.46354	3.518	0.2849	9.83299	3.518	0.074592	9.4966
3.519	2.8034	15.84557	3.519	1.27155	11.7981	3.519	0.62799	10.46769	3.519	0.28545	9.83495	3.519	0.074736	9.4971
3.52	2.80711	15.88768	3.52	1.274	11.806	3.52	0.6292	10.47184	3.52	0.286	9.83691	3.52	0.07488	9.4976
3.521	2.81083	15.92978	3.521	1.27645	11.8139	3.521	0.63041	10.47599	3.521	0.28655	9.83887	3.521	0.075024	9.4981
3.522	2.81456	15.97189	3.522	1.2789	11.8218	3.522	0.63162	10.48014	3.522	0.2871	9.84083	3.522	0.075168	9.4986
3.523	2.81831	16.01399	3.523	1.28135	11.8297	3.523	0.63283	10.48429	3.523	0.28765	9.84279	3.523	0.075312	9.4991
3.524	2.82206	16.05608	3.524	1.2838	11.8376	3.524	0.63404	10.48844	3.524	0.2882	9.84475	3.524	0.075456	9.4996
3.525	2.82582	16.09818	3.525	1.28625	11.8455	3.525	0.63525	10.49259	3.525	0.28875	9.84671	3.525	0.0756	9.5001
3.526	2.82959	16.14027	3.526	1.2887	11.8534	3.526	0.63646	10.49674	3.526	0.2893	9.84867	3.526	0.075744	9.5006
3.527	2.83337	16.18236	3.527	1.29115	11.8613	3.527	0.63767	10.50089	3.527	0.28985	9.85063	3.527	0.075888	9.5011
3.528	2.83716	16.22444	3.528	1.2936	11.8692	3.528	0.63888	10.50504	3.528	0.2904	9.85259	3.528	0.076032	9.5016
3.529	2.84096	16.26653	3.529	1.29605	11.8771	3.529	0.64009	10.50919	3.529	0.29095	9.85455	3.529	0.076176	9.5021
3.53	2.84476	16.30861	3.53	1.2985	11.885	3.53	0.6413	10.51334	3.53	0.2915	9.85651	3.53	0.07632	9.5026
3.531	2.84858	16.35068	3.531	1.30095	11.8929	3.531	0.64251	10.51749	3.531	0.29205	9.85847	3.531	0.076464	9.5031
3.532	2.85241	16.39276	3.532	1.3034	11.9008	3.532	0.64372	10.52164	3.532	0.2926	9.86043	3.532	0.076608	9.5036
3.533	2.85625	16.43483	3.533	1.30585	11.9087	3.533	0.64493	10.52579	3.533	0.29315	9.86239	3.533	0.076752	9.5041
3.534	2.8601	16.4769	3.534	1.3083	11.9166	3.534	0.64614	10.52994	3.534	0.2937	9.86435	3.534	0.076896	9.5046
3.535	2.86396	16.51896	3.535	1.31075	11.9245	3.535	0.64735	10.53409	3.535	0.29425	9.86631	3.535	0.07704	9.5051
3.536	2.86782	16.56103	3.536	1.3132	11.9324	3.536	0.64856	10.53824	3.536	0.2948	9.86827	3.536	0.077184	9.5056
3.537	2.8717	16.60309	3.537	1.31565	11.9403	3.537	0.64977	10.54239	3.537	0.29535	9.87023	3.537	0.077328	9.5061
3.538	2.87559	16.64515	3.538	1.3181	11.9482	3.538	0.65098	10.54654	3.538	0.2959	9.87219	3.538	0.077472	9.5066
3.539	2.87949	16.6872	3.539	1.32055	11.9561	3.539	0.65219	10.55069	3.539	0.29645	9.87415	3.539	0.077616	9.5071
3.54	2.88339	16.72925	3.54	1.323	11.964	3.54	0.6534	10.55484	3.54	0.297	9.87611	3.54	0.07776	9.5076
3.541	2.88731	16.7713	3.541	1.32545	11.9719	3.541	0.65461	10.55899	3.541	0.29755	9.87807	3.541	0.077904	9.5081
3.542	2.89123	16.81335	3.542	1.3279	11.9798	3.542	0.65582	10.56314	3.542	0.2981	9.88003	3.542	0.078048	9.5086
3.543	2.89517	16.85539	3.543	1.33035	11.9877	3.543	0.65703	10.56729	3.543	0.29865	9.88199	3.543	0.078192	9.5091
3.544	2.89911	16.89744	3.544	1.3328	11.9956	3.544	0.65824	10.57144	3.544	0.2992	9.88395	3.544	0.078336	9.5096
3.545	2.90307	16.93947	3.545	1.33525	12.0035	3.545	0.65945	10.57559	3.545	0.29975	9.88591	3.545	0.07848	9.5101
3.546	2.90703	16.98151	3.546	1.3377	12.0114	3.546	0.66066	10.57974	3.546	0.3003	9.88787	3.546	0.078624	9.5106
3.547	2.91101	17.02355	3.547	1.34015	12.0193	3.547	0.66187	10.58389	3.547	0.30085	9.88983	3.547	0.078768	9.5111
3.548	2.91499	17.06558	3.548	1.3426	12.0272	3.548	0.66308	10.58804	3.548	0.3014	9.89179	3.548	0.078912	9.5116
3.549	2.91898	17.10761	3.549	1.34505	12.0351	3.549	0.66429	10.59219	3.549	0.30195	9.89375	3.549	0.079056	9.5121
3.55	2.92299	17.14963	3.55	1.3475	12.043	3.55	0.6655	10.59634	3.55	0.3025	9.89571	3.55	0.0792	9.5126
3.551	2.927	17.19166	3.551	1.34995	12.0509	3.551	0.66671	10.60049	3.551	0.30305	9.89767	3.551	0.079344	9.5131
3.552	2.93102	17.23368	3.552	1.3524	12.0588	3.552	0.66792	10.60464	3.552	0.3036	9.89963	3.552	0.079488	9.5136
3.553	2.93505	17.2757	3.553	1.35485	12.0667	3.553	0.66913	10.60879	3.553	0.30415	9.90159	3.553	0.079632	9.5141
3.554	2.93909	17.31771	3.554	1.3573	12.0746	3.554	0.67034	10.61294	3.554	0.3047	9.90355	3.554	0.079776	9.5146
3.555	2.94314	17.35973	3.555	1.35975	12.0825	3.555	0.67155	10.61709	3.555	0.30525	9.90551	3.555	0.07992	9.5151
3.556	2.9472	17.40174	3.556	1.3622	12.0904	3.556	0.67276	10.62124	3.556	0.3058	9.90747	3.556	0.080064	9.5156
3.557	2.95127	17.44375	3.557	1.36465	12.0983	3.557	0.67397	10.62539	3.557	0.30635	9.90943	3.557	0.080208	9.5161
3.558	2.95535	17.48575	3.558	1.3671	12.1062	3.558	0.67518	10.62954	3.558	0.3069	9.91139	3.558	0.080352	9.5166
3.559	2.95944	17.52776	3.559	1.36955	12.1141	3.559	0.67639	10.63369	3.559	0.30745	9.91335	3.559	0.080496	9.5171
3.56	2.96354	17.56976	3.56	1.372	12.122	3.56	0.6776	10.63784	3.56	0.308	9.91531	3.56	0.08064	9.5176
3.561	2.96765	17.61176	3.561	1.37445	12.1299	3.561	0.67881	10.64199	3.561	0.30855	9.91727	3.561	0.080784	9.5181
3.562	2.97176	17.65376	3.562	1.3769	12.1378	3.562	0.68002	10.64614	3.562	0.3091	9.91923	3.562	0.080928	9.5186
3.563	2.97589	17.69575	3.563	1.37935	12.1457	3.563	0.68123	10.65029	3.563	0.30965	9.92119	3.563	0.081072	9.5191
3.564	2.98003	17.73774	3.564	1.3818	12.1536	3.564	0.68244	10.65444	3.564	0.3102	9.92315	3.564	0.081216	9.5196
3.565	2.98417	17.77973	3.565	1.38425	12.1615	3.565	0.68365	10.65859	3.565	0.31075	9.92511	3.565	0.08136	9.5201
3.566	2.98833	17.82172	3.566	1.3867	12.1694	3.566	0.68486	10.66274	3.566	0.3113	9.92707	3.566	0.081504	9.5206
3.567	2.99249	17.86371	3.567	1.38915	12.1773	3.567	0.68607	10.66689	3.567	0.31185	9.92903	3.567	0.081648	9.5211
3.568	2.99667	17.90569	3.568	1.3916	12.1852	3.568	0.68728	10.67104	3.568	0.3124	9.93099	3.568	0.081792	9.5216
3.569	3.00085	17.94767	3.569	1.39405	12.1931	3.569	0.68849	10.67519	3.569	0.31295	9.93295	3.569	0.081936	9.5221
3.57	3.00504	17.98965	3.57	1.3965	12.201	3.57	0.6897	10.67934	3.57	0.3135	9.93491	3.57	0.08208	9.5226
3.571	3.00924	18.03163	3.571	1.39895	12.2089	3.571	0.69091	10.68349	3.571	0.31405	9.93687	3.571	0.082224	9.5231
3.572	3.01346	18.0736	3.572	1.4014	12.2168	3.572	0.69212	10.68764	3.572	0.3146	9.93883	3.572	0.082368	9.5236
3.573	3.01768	18.11557	3.573	1.40385	12.2247	3.573	0.69333	10.69179	3.573	0.31515	9.94079	3.573	0.082512	9.5241
3.574	3.02191	18.15754	3.574	1.4063	12.2326	3.574	0.69454	10.69594	3.574	0.3157	9.94275	3.574	0.082656	9.5246
3.575	3.02615	18.19951	3.575	1.40875	12.2405	3.575	0.69575	10.70009	3.575	0.31625	9.94471	3.575	0.0828	9.5251
3.576	3.03039	18.24148	3.576	1.4112	12.2484	3.576	0.69696	10.70424	3.576	0.3168	9.94667	3.576	0.082944	9.5256
3.577	3.03465	18.28344	3.577	1.41365	12.2563	3.577	0.69817	10.70839	3.577	0.31735	9.94863	3.577	0.083088	9.5261
3.578	3.03892	18.3254	3.578	1.4161	12.2642	3.578	0.69938	10.71254	3.578	0.3179	9.95059	3.578	0.083232	9.5266
3.579	3.0432	18.36736	3.579	1.41855	12.2721	3.579	0.70059	10.71669	3.579	0.31845	9.95255	3.579	0.083376	9.5271
3.58	3.04748	18.40932	3.58	1.421	12.28	3.58	0.7018	10.72084	3.58	0.319	9.95451	3.58	0.08352	9.5276
3.581	3.05178	18.45127	3.581	1.42345	12.2879	3.581	0.70301	10.72499	3.581	0.31955	9.95647	3.581	0.083664	9.5281
3.582	3.05608	18.49323	3.582	1.4259	12.2958	3.582	0.70422	10.72914	3.582	0.3201	9.95843	3.582	0.083808	9.5286
3.583	3.0604	18.53518	3.583	1.42835	12.3037	3.583	0.70543	10.73329	3.583	0.32065	9.96039	3.583	0.083952	9.5291
3.584	3.06472	18.57713	3.584	1.4308	12.3116	3.584	0.70664	10.73744	3.584	0.3212	9.96235	3.584	0.084096	9.5296
3.585	3.06906	18.61908	3.585	1.43325	12.3195	3.585	0.70785	10.74159	3.585	0.32175	9.96431	3.585	0.08424	9.5301
3.586	3.0734	18.66102	3.586	1.4357	12.3274	3.586	0.70906	10.74574	3.586	0.3223	9.96627	3.586	0.084384	9.5306
3.587	3.07775	18.70296	3.587	1.43815	12.3353	3.587	0.71027	10.74989	3.587	0.32285	9.96823	3.587	0.084528	9.5311
3.588	3.08211	18.7449	3.588	1.4406	12.3432	3.588	0.71148	10.75404	3.588	0.3234	9.97019	3.588	0.084672	9.5316
3.589	3.08648	18.78684	3.589	1.44305	12.3511	3.589	0.71269	10.75819	3.589	0.32395	9.97215	3.589	0.084816	9.5321
3.59	3.09086	18.82878	3.59	1.4455	12.359	3.59	0.7139	10.76234	3.59	0.3245	9.97411	3.59	0.08496	9.5326
3.591	3.09525	18.87072	3.591	1.44795	12.3669	3.591	0.71511	10.76649	3.591	0.32505	9.97607	3.591	0.085104	9.5331
3.592	3.09965	18.91265	3.592	1.4504	12.3748	3.592	0.71632	10.77064	3.592	0.3256	9.97803	3.592	0.085248	9.5336
3.593	3.10405	18.95458	3.593	1.45285	12.3827	3.593	0.71753	10.77479	3.593	0.32615	9.97999	3.593	0.085392	9.5341
3.594	3.10847	18.99651	3.594	1.4553	12.3906	3.594	0.71874	10.77894	3.594	0.3267	9.98195	3.594	0.085536	9.5346
3.595	3.11289	19.03844	3.595	1.45775	12.3985	3.595	0.71995	10.78309	3.595	0.32725	9.98391	3.595	0.08568	9.5351
3.596	3.11733	19.08036	3.596	1.4602	12.4064	3.596	0.72116	10.78724	3.596	0.3278	9.98587	3.596	0.085824	9.5356
3.597	3.12177	19.12229	3.597	1.46265	12.4143	3.597	0.72237	10.79139	3.597	0.32835	9.98783	3.597	0.085968	9.5361
3.598	3.12622	19.16421	3.598	1.4651	12.4222	3.598	0.72358	10.79554	3.598	0.3289	9.98979	3.598	0.086112	9.5366
3.599	3.13069	19.20613	3.599	1.46755	12.4301	3.599	0.72479	10.79969	3.599	0.32945	9.99175	3.599	0.086256	9.5371
3.6	3.13516	19.24805	3.6	1.47	12.438	3.6	0.726	10.80384	3.6	0.33	9.99371	3.6	0.0864	9.5376
3.601	3.13964	19.28996	3.601	1.47245	12.4459	3.601	0.72721	10.80799	3.601	0.33055	9.99567	3.601	0.086544	9.5381
3.602	3.14413	19.33188	3.602	1.4749	12.4538	3.602	0.72842	10.81214	3.602	0.3311	9.99763	3.602	0.086688	9.5386
3.603	3.14863	19.37379	3.603	1.47735	12.4617	3.603	0.72963	10.81629	3.603	0.33165	9.99959	3.603	0.086832	9.5391
3.604	3.15313	19.4157	3.604	1.4798	12.4696	3.604	0.73084	10.82044	3.604	0.3322	10.00155	3.604	0.086976	9.5396
3.605	3.15765	19.45761	3.605	1.48225	12.4775	3.605	0.73205	10.82459	3.605	0.33275	10.00351	3.605	0.08712	9.5401
3.606	3.16218	19.49952	3.606	1.4847	12.4854	3.606	0.73326	10.82874	3.606	0.3333	10.00547	3.606	0.087264	9.5406
3.607	3.16671	19.54143	3.607	1.48715	12.4933	3.607	0.73447	10.83289	3.607	0.33385	10.00743	3.607	0.087408	9.5411
3.608	3.17126	19.58333	3.608	1.4896	12.5012	3.608	0.73568	10.83704	3.608	0.3344	10.00939	3.608	0.087552	9.5416
3.609	3.17581	19.62523	3.609	1.49205	12.5091	3.609	0.73689	10.84119	3.609	0.33495	10.01135	3.609	0.087696	9.5421
3.61	3.18037	19.66714	3.61	1.4945	12.517	3.61	0.7381	10.84534	3.61	0.3355	10.01331	3.61	0.08784	9.5426
3.611	3.18494	19.70904	3.611	1.49695	12.5249	3.611	0.73931	10.84949	3.611	0.33605	10.01527	3.611	0.087984	9.5431
3.612	3.18952	19.75093	3.612	1.4994	12.5328	3.612	0.74052	10.85364	3.612	0.3366	10.01723	3.612	0.088128	9.5436
3.613	3.19411	19.79283	3.613	1.50185	12.5407	3.613	0.74173	10.85779	3.613	0.33715	10.01919	3.613	0.088272	9.5441
3.614	3.19871	19.83472	3.614	1.5043	12.5486	3.614	0.74294	10.86194	3.614	0.3377	10.02115	3.614	0.088416	9.5446
3.615	3.20332	19.87662	3.615	1.50675	12.5565	3.615	0.74415	10.86609	3.615	0.33825	10.02311	3.615	0.08856	9.5451
3.616	3.20794	19.91851	3.616	1.5092	12.5644	3.616	0.74536	10.87024	3.616	0.3388	10.02507	3.616	0.088704	9.5456
3.617	3.21256	19.9604	3.617	1.51165	12.5723	3.617	0.74657	10.87439	3.617	0.33935	10.02703	3.617	0.088848	9.5461
3.618	3.2172	20.00229	3.618	1.5141	12.5802	3.618	0.74778	10.87854	3.618	0.3399	10.02899	3.618	0.088992	9.5466
3.619	3.22184	20.04417	3.619	1.51655	12.5881	3.619	0.74899	10.88269	3.619	0.34045	10.03095	3.619	0.089136	9.5471
3.62	3.22649	20.08606	3.62	1.519	12.596	3.62	0.7502	10.88684	3.62	0.341	10.03291	3.62	0.08928	9.5476
3.621	3.23116	20.12794	3.621	1.52145	12.6039	3.621	0.75141	10.89099	3.621	0.34155	10.03487	3.621	0.089424	9.5481
3.622	3.23583	20.16982	3.622	1.5239	12.6118	3.622	0.75262	10.89514	3.622	0.3421	10.03683	3.622	0.089568	9.5486
3.623	3.24051	20.2117	3.623	1.52635	12.6197	3.623	0.75383	10.89929	3.623	0.34265	10.03879	3.623	0.089712	9.5491
3.624	3.2452	20.25358	3.624	1.5288	12.6276	3.624	0.75504	10.90344	3.624	0.3432	10.04075	3.624	0.089856	9.5496
3.625	3.24989	20.29546	3.625	1.53125	12.6355	3.625	0.75625	10.90759	3.625	0.34375	10.04271	3.625	0.09	9.5501
3.626	3.2546	20.33734	3.626	1.5337	12.6434	3.626	0.75746	10.91174	3.626	0.3443	10.04467	3.626	0.090144	9.5506
3.627	3.25932	20.37921	3.627	1.53615	12.6513	3.627	0.75867	10.91589	3.627	0.34485	10.04663	3.627	0.090288	9.5511
3.628	3.26404	20.42108	3.628	1.5386	12.6592	3.628	0.75988	10.92004	3.628	0.3454	10.04859	3.628	0.090432	9.5516
3.629	3.26877	20.46296	3.629	1.54105	12.6671	3.629	0.76109	10.92419	3.629	0.34595	10.05055	3.629	0.090576	9.5521
3.63	3.27352	20.50483	3.63	1.5435	12.675	3.63	0.7623	10.92834	3.63	0.3465	10.05251	3.63	0.09072	9.5526
3.631	3.27827	20.5467	3.631	1.54595	12.6829	3.631	0.76351	10.93249	3.631	0.34705	10.05447	3.631	0.090864	9.5531
3.632	3.28303	20.58856	3.632	1.5484	12.6908	3.632	0.76472	10.93664	3.632	0.3476	10.05643	3.632	0.091008	9.5536
3.633	3.2878	20.63043	3.633	1.55085	12.6987	3.633	0.76593	10.94079	3.633	0.34815	10.05839	3.633	0.091152	9.5541
3.634	3.29258	20.67229	3.634	1.5533	12.7066	3.634	0.76714	10.94494	3.634	0.3487	10.06035	3.634	0.091296	9.5546
3.635	3.29736	20.71416	3.635	1.55575	12.7145	3.635	0.76835	10.94909	3.635	0.34925	10.06231	3.635	0.09144	9.5551
3.636	3.30216	20.75602	3.636	1.5582	12.7224	3.636	0.76956	10.95324	3.636	0.3498	10.06427	3.636	0.091584	9.5556
3.637	3.30696	20.79788	3.637	1.56065	12.7303	3.637	0.77077	10.95739	3.637	0.35035	10.06623	3.637	0.091728	9.5561

3.638	3.31178	20.83974	3.638	1.5631	12.7382	3.638	0.77198	10.96154	3.638	0.3509	10.06819	3.638	0.091872	9.5566
3.639	3.3166	20.8816	3.639	1.56555	12.7461	3.639	0.77319	10.96569	3.639	0.35145	10.07015	3.639	0.092016	9.5571
3.64	3.32143	20.92346	3.64	1.568	12.754	3.64	0.7744	10.96984	3.64	0.352	10.07211	3.64	0.09216	9.5576
3.641	3.32627	20.96531	3.641	1.57045	12.7619	3.641	0.77561	10.97399	3.641	0.35255	10.07407	3.641	0.092304	9.5581
3.642	3.33112	21.00717	3.642	1.5729	12.7698	3.642	0.77682	10.97814	3.642	0.3531	10.07603	3.642	0.092448	9.5586
3.643	3.33598	21.04902	3.643	1.57535	12.7777	3.643	0.77803	10.98229	3.643	0.35365	10.07799	3.643	0.092592	9.5591
3.644	3.34084	21.09087	3.644	1.5778	12.7856	3.644	0.77924	10.98644	3.644	0.3542	10.07995	3.644	0.092736	9.5596
3.645	3.34572	21.13272	3.645	1.58025	12.7935	3.645	0.78045	10.99059	3.645	0.35475	10.08191	3.645	0.09288	9.5601
3.646	3.3506	21.17457	3.646	1.5827	12.8014	3.646	0.78166	10.99474	3.646	0.3553	10.08387	3.646	0.093024	9.5606
3.647	3.3555	21.21642	3.647	1.58515	12.8093	3.647	0.78287	10.99889	3.647	0.35585	10.08583	3.647	0.093168	9.5611
3.648	3.3604	21.25827	3.648	1.5876	12.8172	3.648	0.78408	11.00304	3.648	0.3564	10.08779	3.648	0.093312	9.5616
3.649	3.36531	21.30011	3.649	1.59005	12.8251	3.649	0.78529	11.00719	3.649	0.35695	10.08975	3.649	0.093456	9.5621
3.65	3.37023	21.34196	3.65	1.5925	12.833	3.65	0.7865	11.01134	3.65	0.3575	10.09171	3.65	0.0936	9.5626
3.651	3.37516	21.3838	3.651	1.59495	12.8409	3.651	0.78771	11.01549	3.651	0.35805	10.09367	3.651	0.093744	9.5631
3.652	3.38009	21.42565	3.652	1.5974	12.8488	3.652	0.78892	11.01964	3.652	0.3586	10.09563	3.652	0.093888	9.5636
3.653	3.38504	21.46749	3.653	1.59985	12.8567	3.653	0.79013	11.02379	3.653	0.35915	10.09759	3.653	0.094032	9.5641
3.654	3.38999	21.50933	3.654	1.6023	12.8646	3.654	0.79134	11.02794	3.654	0.3597	10.09955	3.654	0.094176	9.5646
3.655	3.39496	21.55117	3.655	1.60475	12.8725	3.655	0.79255	11.03209	3.655	0.36025	10.10151	3.655	0.09432	9.5651
3.656	3.39993	21.59301	3.656	1.6072	12.8804	3.656	0.79376	11.03624	3.656	0.3608	10.10347	3.656	0.094464	9.5656
3.657	3.40491	21.63484	3.657	1.60965	12.8883	3.657	0.79497	11.04039	3.657	0.36135	10.10543	3.657	0.094608	9.5661
3.658	3.4099	21.67668	3.658	1.6121	12.8962	3.658	0.79618	11.04454	3.658	0.3619	10.10739	3.658	0.094752	9.5666
3.659	3.4149	21.71852	3.659	1.61455	12.9041	3.659	0.79739	11.04869	3.659	0.36245	10.10935	3.659	0.094896	9.5671
3.66	3.4199	21.76035	3.66	1.617	12.912	3.66	0.7986	11.05284	3.66	0.363	10.11131	3.66	0.09504	9.5676
3.661	3.42492	21.80218	3.661	1.61945	12.9199	3.661	0.79981	11.05699	3.661	0.36355	10.11327	3.661	0.095184	9.5681
3.662	3.42994	21.84402	3.662	1.6219	12.9278	3.662	0.80102	11.06114	3.662	0.3641	10.11523	3.662	0.095328	9.5686
3.663	3.43498	21.88585	3.663	1.62435	12.9357	3.663	0.80223	11.06529	3.663	0.36465	10.11719	3.663	0.095472	9.5691
3.664	3.44002	21.92768	3.664	1.6268	12.9436	3.664	0.80344	11.06944	3.664	0.3652	10.11915	3.664	0.095616	9.5696
3.665	3.44507	21.96951	3.665	1.62925	12.9515	3.665	0.80465	11.07359	3.665	0.36575	10.12111	3.665	0.09576	9.5701
3.666	3.45013	22.01134	3.666	1.6317	12.9594	3.666	0.80586	11.07774	3.666	0.3663	10.12307	3.666	0.095904	9.5706
3.667	3.45519	22.05316	3.667	1.63415	12.9673	3.667	0.80707	11.08189	3.667	0.36685	10.12503	3.667	0.096048	9.5711
3.668	3.46027	22.09499	3.668	1.6366	12.9752	3.668	0.80828	11.08604	3.668	0.3674	10.12699	3.668	0.096192	9.5716
3.669	3.46535	22.13681	3.669	1.63905	12.9831	3.669	0.80949	11.09019	3.669	0.36795	10.12895	3.669	0.096336	9.5721
3.67	3.47045	22.17864	3.67	1.6415	12.991	3.67	0.8107	11.09434	3.67	0.3685	10.13091	3.67	0.09648	9.5726
3.671	3.47555	22.22046	3.671	1.64395	12.9989	3.671	0.81191	11.09849	3.671	0.36905	10.13287	3.671	0.096624	9.5731
3.672	3.48066	22.26229	3.672	1.6464	13.0068	3.672	0.81312	11.10264	3.672	0.3696	10.13483	3.672	0.096768	9.5736
3.673	3.48578	22.30411	3.673	1.64885	13.0147	3.673	0.81433	11.10679	3.673	0.37015	10.13679	3.673	0.096912	9.5741
3.674	3.49091	22.34593	3.674	1.6513	13.0226	3.674	0.81554	11.11094	3.674	0.3707	10.13875	3.674	0.097056	9.5746
3.675	3.49604	22.38775	3.675	1.65375	13.0305	3.675	0.81675	11.11509	3.675	0.37125	10.14071	3.675	0.0972	9.5751
3.676	3.50119	22.42957	3.676	1.6562	13.0384	3.676	0.81796	11.11924	3.676	0.3718	10.14267	3.676	0.097344	9.5756
3.677	3.50634	22.47139	3.677	1.65865	13.0463	3.677	0.81917	11.12339	3.677	0.37235	10.14463	3.677	0.097488	9.5761
3.678	3.5115	22.51321	3.678	1.6611	13.0542	3.678	0.82038	11.12754	3.678	0.3729	10.14659	3.678	0.097632	9.5766
3.679	3.51667	22.55502	3.679	1.66355	13.0621	3.679	0.82159	11.13169	3.679	0.37345	10.14855	3.679	0.097776	9.5771
3.68	3.52185	22.59684	3.68	1.666	13.07	3.68	0.8228	11.13584	3.68	0.374	10.15051	3.68	0.09792	9.5776
3.681	3.52704	22.63866	3.681	1.66845	13.0779	3.681	0.82401	11.13999	3.681	0.37455	10.15247	3.681	0.098064	9.5781
3.682	3.53224	22.68047	3.682	1.6709	13.0858	3.682	0.82522	11.14414	3.682	0.3751	10.15443	3.682	0.098208	9.5786
3.683	3.53744	22.72229	3.683	1.67335	13.0937	3.683	0.82643	11.14829	3.683	0.37565	10.15639	3.683	0.098352	9.5791
3.684	3.54265	22.7641	3.684	1.6758	13.1016	3.684	0.82764	11.15244	3.684	0.3762	10.15835	3.684	0.098496	9.5796
3.685	3.54788	22.80591	3.685	1.67825	13.1095	3.685	0.82885	11.15659	3.685	0.37675	10.16031	3.685	0.09864	9.5801
3.686	3.55311	22.84772	3.686	1.6807	13.1174	3.686	0.83006	11.16074	3.686	0.3773	10.16227	3.686	0.098784	9.5806
3.687	3.55835	22.88954	3.687	1.68315	13.1253	3.687	0.83127	11.16489	3.687	0.37785	10.16423	3.687	0.098928	9.5811
3.688	3.56359	22.93135	3.688	1.6856	13.1332	3.688	0.83248	11.16904	3.688	0.3784	10.16619	3.688	0.099072	9.5816
3.689	3.56885	22.97316	3.689	1.68805	13.1411	3.689	0.83369	11.17319	3.689	0.37895	10.16815	3.689	0.099216	9.5821
3.69	3.57411	23.01497	3.69	1.6905	13.149	3.69	0.8349	11.17734	3.69	0.3795	10.17011	3.69	0.09936	9.5826
3.691	3.57939	23.05678	3.691	1.69295	13.1569	3.691	0.83611	11.18149	3.691	0.38005	10.17207	3.691	0.099504	9.5831
3.692	3.58467	23.09858	3.692	1.6954	13.1648	3.692	0.83732	11.18564	3.692	0.3806	10.17403	3.692	0.099648	9.5836
3.693	3.58996	23.14039	3.693	1.69785	13.1727	3.693	0.83853	11.18979	3.693	0.38115	10.17599	3.693	0.099792	9.5841
3.694	3.59525	23.1822	3.694	1.7003	13.1806	3.694	0.83974	11.19394	3.694	0.3817	10.17795	3.694	0.099936	9.5846
3.695	3.60056	23.224	3.695	1.70275	13.1885	3.695	0.84095	11.19809	3.695	0.38225	10.17991	3.695	0.10008	9.5851
3.696	3.60588	23.26581	3.696	1.7052	13.1964	3.696	0.84216	11.20224	3.696	0.3828	10.18187	3.696	0.100224	9.5856
3.697	3.6112	23.30762	3.697	1.70765	13.2043	3.697	0.84337	11.20639	3.697	0.38335	10.18383	3.697	0.100368	9.5861
3.698	3.61653	23.34942	3.698	1.7101	13.2122	3.698	0.84458	11.21054	3.698	0.3839	10.18579	3.698	0.100512	9.5866
3.699	3.62187	23.39122	3.699	1.71255	13.2201	3.699	0.84579	11.21469	3.699	0.38445	10.18775	3.699	0.100656	9.5871
3.7	3.62722	23.43303	3.7	1.715	13.228	3.7	0.847	11.21884	3.7	0.385	10.18971	3.7	0.1008	9.5876
3.701	3.63258	23.47483	3.701	1.71745	13.2359	3.701	0.84821	11.22299	3.701	0.38555	10.19167	3.701	0.100944	9.5881
3.702	3.63794	23.51663	3.702	1.7199	13.2438	3.702	0.84942	11.22714	3.702	0.3861	10.19363	3.702	0.101088	9.5886
3.703	3.64332	23.55844	3.703	1.72235	13.2517	3.703	0.85063	11.23129	3.703	0.38665	10.19559	3.703	0.101232	9.5891
3.704	3.6487	23.60024	3.704	1.7248	13.2596	3.704	0.85184	11.23544	3.704	0.3872	10.19755	3.704	0.101376	9.5896
3.705	3.65409	23.64204	3.705	1.72725	13.2675	3.705	0.85305	11.23959	3.705	0.38775	10.19951	3.705	0.10152	9.5901
3.706	3.65949	23.68384	3.706	1.7297	13.2754	3.706	0.85426	11.24374	3.706	0.3883	10.20147	3.706	0.101664	9.5906
3.707	3.6649	23.72564	3.707	1.73215	13.2833	3.707	0.85547	11.24789	3.707	0.38885	10.20343	3.707	0.101808	9.5911
3.708	3.67031	23.76744	3.708	1.7346	13.2912	3.708	0.85668	11.25204	3.708	0.3894	10.20539	3.708	0.101952	9.5916
3.709	3.67574	23.80924	3.709	1.73705	13.2991	3.709	0.85789	11.25619	3.709	0.38995	10.20735	3.709	0.102096	9.5921
3.71	3.68117	23.85104	3.71	1.7395	13.307	3.71	0.8591	11.26034	3.71	0.3905	10.20931	3.71	0.10224	9.5926
3.711	3.68661	23.89284	3.711	1.74195	13.3149	3.711	0.86031	11.26449	3.711	0.39105	10.21127	3.711	0.102384	9.5931
3.712	3.69206	23.93463	3.712	1.7444	13.3228	3.712	0.86152	11.26864	3.712	0.3916	10.21323	3.712	0.102528	9.5936
3.713	3.69752	23.97643	3.713	1.74685	13.3307	3.713	0.86273	11.27279	3.713	0.39215	10.21519	3.713	0.102672	9.5941
3.714	3.70298	24.01823	3.714	1.7493	13.3386	3.714	0.86394	11.27694	3.714	0.3927	10.21715	3.714	0.102816	9.5946
3.715	3.70846	24.06003	3.715	1.75175	13.3465	3.715	0.86515	11.28109	3.715	0.39325	10.21911	3.715	0.10296	9.5951
3.716	3.71394	24.10182	3.716	1.7542	13.3544	3.716	0.86636	11.28524	3.716	0.3938	10.22107	3.716	0.103104	9.5956
3.717	3.71943	24.14362	3.717	1.75665	13.3623	3.717	0.86757	11.28939	3.717	0.39435	10.22303	3.717	0.103248	9.5961
3.718	3.72493	24.18542	3.718	1.7591	13.3702	3.718	0.86878	11.29354	3.718	0.3949	10.22499	3.718	0.103392	9.5966
3.719	3.73044	24.22721	3.719	1.76155	13.3781	3.719	0.86999	11.29769	3.719	0.39545	10.22695	3.719	0.103536	9.5971
3.72	3.73595	24.26901	3.72	1.764	13.386	3.72	0.8712	11.30184	3.72	0.396	10.22891	3.72	0.10368	9.5976
3.721	3.74147	24.3108	3.721	1.76645	13.3939	3.721	0.87241	11.30599	3.721	0.39655	10.23087	3.721	0.103824	9.5981
3.722	3.74701	24.3526	3.722	1.7689	13.4018	3.722	0.87362	11.31014	3.722	0.3971	10.23283	3.722	0.103968	9.5986
3.723	3.75255	24.39439	3.723	1.77135	13.4097	3.723	0.87483	11.31429	3.723	0.39765	10.23479	3.723	0.104112	9.5991
3.724	3.7581	24.43619	3.724	1.77409	13.42969	3.724	0.87604	11.31844	3.724	0.3982	10.23675	3.724	0.104256	9.5996
3.725	3.76365	24.47798	3.725	1.77722	13.47237	3.725	0.87725	11.32259	3.725	0.39875	10.23871	3.725	0.1044	9.6001
3.726	3.76922	24.51977	3.726	1.78037	13.51505	3.726	0.87846	11.32674	3.726	0.3993	10.24067	3.726	0.104544	9.6006
3.727	3.77479	24.56157	3.727	1.78352	13.55773	3.727	0.87967	11.33089	3.727	0.39985	10.24263	3.727	0.104688	9.6011
3.728	3.78037	24.60336	3.728	1.78669	13.60041	3.728	0.88088	11.33504	3.728	0.4004	10.24459	3.728	0.104832	9.6016
3.729	3.78596	24.64515	3.729	1.78986	13.64309	3.729	0.88209	11.33919	3.729	0.40095	10.24655	3.729	0.104976	9.6021
3.73	3.79156	24.68695	3.73	1.79304	13.68576	3.73	0.8833	11.34334	3.73	0.4015	10.24851	3.73	0.10512	9.6026
3.731	3.79717	24.72874	3.731	1.79624	13.72844	3.731	0.88451	11.34749	3.731	0.40205	10.25047	3.731	0.105264	9.6031
3.732	3.80278	24.77053	3.732	1.79944	13.77111	3.732	0.88572	11.35164	3.732	0.4026	10.25243	3.732	0.105408	9.6036
3.733	3.8084	24.81233	3.733	1.80265	13.81378	3.733	0.88693	11.35579	3.733	0.40315	10.25439	3.733	0.105552	9.6041
3.734	3.81403	24.85412	3.734	1.80588	13.85645	3.734	0.88814	11.35994	3.734	0.4037	10.25635	3.734	0.105696	9.6046
3.735	3.81967	24.89591	3.735	1.80911	13.89912	3.735	0.88935	11.36409	3.735	0.40425	10.25831	3.735	0.10584	9.6051
3.736	3.82532	24.9377	3.736	1.81235	13.94178	3.736	0.89056	11.36824	3.736	0.4048	10.26027	3.736	0.105984	9.6056
3.737	3.83097	24.9795	3.737	1.8156	13.98445	3.737	0.89177	11.37239	3.737	0.40535	10.26223	3.737	0.106128	9.6061
3.738	3.83664	25.02129	3.738	1.81886	14.02711	3.738	0.89298	11.37654	3.738	0.4059	10.26419	3.738	0.106272	9.6066
3.739	3.84231	25.06308	3.739	1.82213	14.06978	3.739	0.89419	11.38069	3.739	0.40645	10.26615	3.739	0.106416	9.6071
3.74	3.84799	25.10487	3.74	1.82541	14.11244	3.74	0.8954	11.38484	3.74	0.407	10.26811	3.74	0.10656	9.6076
3.741	3.85368	25.14666	3.741	1.8287	14.1551	3.741	0.89661	11.38899	3.741	0.40755	10.27007	3.741	0.106704	9.6081
3.742	3.85937	25.18846	3.742	1.832	14.19776	3.742	0.89782	11.39314	3.742	0.4081	10.27203	3.742	0.106848	9.6086
3.743	3.86508	25.23025	3.743	1.83531	14.24041	3.743	0.89903	11.39729	3.743	0.40865	10.27399	3.743	0.106992	9.6091
3.744	3.87079	25.27204	3.744	1.83863	14.28307	3.744	0.90024	11.40144	3.744	0.4092	10.27595	3.744	0.107136	9.6096
3.745	3.87651	25.31383	3.745	1.84196	14.32572	3.745	0.90145	11.40559	3.745	0.40975	10.27791	3.745	0.10728	9.6101
3.746	3.88224	25.35562	3.746	1.8453	14.36838	3.746	0.90266	11.40974	3.746	0.4103	10.27987	3.746	0.107424	9.6106
3.747	3.88797	25.39741	3.747	1.84865	14.41103	3.747	0.90387	11.41389	3.747	0.41085	10.28183	3.747	0.107568	9.6111
3.748	3.89372	25.43921	3.748	1.852	14.45368	3.748	0.90508	11.41804	3.748	0.4114	10.28379	3.748	0.107712	9.6116
3.749	3.89947	25.481	3.749	1.85537	14.49633	3.749	0.90629	11.42219	3.749	0.41195	10.28575	3.749	0.107856	9.6121
3.75	3.90523	25.52279	3.75	1.85875	14.53898	3.75	0.9075	11.42634	3.75	0.4125	10.28771	3.75	0.108	9.6126
3.751	3.911	25.56458	3.751	1.86213	14.58163	3.751	0.90871	11.43049	3.751	0.41305	10.28967	3.751	0.108144	9.6131
3.752	3.91678	25.60637	3.752	1.86553	14.62428	3.752	0.90992	11.43464	3.752	0.4136	10.29163	3.752	0.108288	9.6136
3.753	3.92256	25.64816	3.753	1.86893	14.66692	3.753	0.91113	11.43879	3.753	0.41415	10.29359	3.753	0.108432	9.6141
3.754	3.92835	25.68996	3.754	1.87235	14.70957	3.754	0.91234	11.44294	3.754	0.4147	10.29555	3.754	0.108576	9.6146
3.755	3.93415	25.73175	3.755	1.87577	14.75221	3.755	0.91355	11.44709	3.755	0.41525	10.29751	3.755	0.10872	9.6151
3.756	3.93996	25.77354	3.756	1.87921	14.79485	3.756	0.91476	11.45124	3.756	0.4158	10.29947	3.756	0.108864	9.6156
3.757	3.94578	25.81533	3.757	1.88265	14.83749	3.757	0.91597	11.45539	3.757	0.41635	10.30143	3.757	0.109008	9.6161
3.758	3.95161	25.85713	3.758	1.8861	14.88013	3.758	0.91718	11.45954	3.758	0.4169	10.30339	3.758	0.109152	9.6166
3.759	3.95744	25.89892	3.759	1.88957	14.92277	3.759	0.91839	11.46369	3.759	0.41745	10.30535	3.759	0.109296	9.6171
3.76	3.96328	25.94071	3.76	1.89304	14.96541	3.76	0.9196	11.46784	3.76	0.418	10.30731	3.76	0.10944	9.6176
3.761	3.96913	25.9825	3.761	1.89652	15.00805	3.761	0.92081	11.47199	3.761	0.41855	10.30927	3.761	0.109584	9.6181
3.762	3.97499	26.0243	3.762	1.90001	15.05069	3.762	0.92202	11.47614	3.762	0.4191	10.31123	3.762	0.109728	9.6186
3.763	3.98085	26.06609	3.763	1.90351	15.09332	3.763	0.92323	11.48029	3.763	0.41965	10.31319	3.763	0.109872	9.6191
3.764	3.98672	26.10788	3.764	1.90702	15.13596	3.764	0.92444	11.48444	3.764	0.4202	10.31515	3.764	0.110016	9.6196
3.765	3.99261	26.14968	3.765	1.91054	15.17859	3.765	0.92565	11.48859	3.765	0.42075	10.31711	3.765	0.11016	9.6201
3.766	3.99849	26.19147	3.766	1.91407	15.22122	3.766	0.92686	11.49274	3.766	0.4213	10.31907	3.766	0.110304	9.6206
3.767	4.00439	26.23327	3.767	1.91761	15.26385	3.767	0.92807	11.49689	3.767	0.42185	10.32103	3.767	0.110448	9.6211
3.768	4.0103	26.27506	3.768	1.92116	15.30648	3.768	0.92928	11.50104	3.768	0.4224	10.32299	3.768	0.110592	9.6216
3.769	4.01621	26.31685	3.769	1.92471	15.34911	3.769	0.93049	11.50519	3.769	0.42295	10.32495	3.769	0.110736	9.6221
3.77	4.02213	26.35865	3.77	1.92828	15.39174	3.77	0.9317	11.50934	3.77	0.4235	10.32691	3.77	0.11088	9.6226
3.771	4.02806	26.40044	3.771	1.93185	15.43437	3.771	0.93291	11.51349	3.771	0.42405	10.32887	3.771	0.111024	9.6231
3.772	4.034	26.44224	3.772	1.93544	15.477	3.772	0.93412	11.51764	3.772	0.4246	10.33083	3.772	0.111168	9.6236
3.773	4.03994	26.48404	3.773	1.93903	15.51963	3.773	0.93533	11.52179	3.773	0.42515	10.33279	3.773	0.111312	9.6241
3.774	4.04589	26.52583	3.774	1.94264	15.56225	3.774	0.93654	11.52594	3.774	0.4257	10.33475	3.774	0.111456	9.6246
3.775	4.05185	26.56763	3.775	1.94625	15.60488	3.775	0.93775	11.53009	3.775	0.42625	10.33671	3.775	0.1116	9.6251
3.776	4.05782	26.60942	3.776	1.94988	15.6475	3.776	0.93896	11.53424	3.776	0.4268	10.33867	3.776	0.111744	9.6256
3.777	4.0638	26.65122	3.777	1.95351	15.69013	3.777	0.94017	11.53839	3.777	0.42735	10.34063	3.777	0.111888	9.6261
3.778	4.06978	26.69302	3.778	1.95715	15.73275	3.778	0.94138	11.54254	3.778	0.4279	10.34259	3.778	0.112032	9.6266
3.779	4.07577	26.73482	3.779	1.9608	15.77537	3.779	0.94259	11.54669	3.779	0.42845	10.34455	3.779	0.112176	9.6271
3.78	4.08177	26.77661	3.78	1.96446	15.81799	3.78	0.9438	11.55084	3.78	0.429	10.34651	3.78	0.11232	9.6276
3.781	4.08778	26.81841	3.781	1.96813	15.86061	3.781	0.94501	11.55499	3.781	0.42955	10.34847	3.781	0.112464	9.6281
3.782	4.0938	26.86021	3.782	1.97181	15.90323	3.782	0.94622	11.55914	3.782	0.4301	10.35043	3.782	0.112608	9.6286
3.783	4.09982	26.90201	3.783	1.9755	15.94585	3.783	0.94743	11.56329	3.783	0.43065	10.35239	3.783	0.112752	9.6291
3.784	4.10585	26.94381	3.784	1.97919	15.98847	3.784	0.94864	11.56744	3.784	0.4312	10.35435	3.784	0.112896	9.6296
3.785	4.11189	26.98561	3.785	1.9829	16.03109	3.785	0.94985	11.57159	3.785	0.43175	10.35631	3.785	0.11304	9.6301
3.786	4.11794	27.02741	3.786	1.98662	16.07371	3.786	0.95106	11.57574	3.786	0.4323	10.35827	3.786	0.113184	9.6306
3.787	4.12399	27.06921	3.787	1.99034	16.11632	3.787	0.95227	11.57989	3.787	0.43285	10.36023	3.787	0.113328	9.6311
3.788	4.13006	27.11101	3.788	1.99408	16.15894	3.788	0.95348	11.58404	3.788	0.4334	10.36219	3.788	0.113472	9.6316
3.789	4.13613	27.15281	3.789	1.99782	16.20155	3.789	0.95469	11.58819	3.789	0.43395	10.36415	3.789	0.113616	9.6321
3.79	4.14221	27.19462	3.79	2.00158	16.24417	3.79	0.9559	11.59234	3.79	0.4345	10.36611	3.79	0.11376	9.6326
3.791	4.14829	27.23642	3.791	2.00534	16.28678	3.791	0.95711	11.59649	3.791	0.43505	10.36807	3.791	0.113904	9.6331
3.792	4.15439	27.27822	3.792	2.00911	16.3294	3.792	0.95832	11.60064	3.792	0.4356	10.37003	3.792	0.114048	9.6336
3.793	4.16049	27.32002	3.793	2.01289	16.37201	3.793	0.95953	11.60479	3.793	0.43615	10.37199	3.793	0.114192	9.6341
3.794	4.1666	27.36183	3.794	2.01668	16.41462	3.794	0.96074	11.60894	3.794	0.4367	10.37395	3.794	0.114336	9.6346
3.795	4.17272	27.40363	3.795	2.02048	16.45724	3.795	0.96195	11.61309	3.795	0.43725	10.37591	3.795	0.11448	9.6351
3.796	4.17884	27.44544	3.796	2.02429	16.49985	3.796	0.96316	11.61724	3.796	0.4378	10.37787	3.796	0.114624	9.6356
3.797	4.18497	27.48724	3.797	2.02811	16.54246	3.797	0.96437	11.62139	3.797	0.43835	10.37983	3.797	0.114768	9.6361

3.798	4.19112	27.52905	3.798	2.03193	16.58507	3.798	0.96558	11.62554	3.798	0.4389	10.38179	3.798	0.114912	9.6366
3.799	4.19726	27.57086	3.799	2.03577	16.62768	3.799	0.96679	11.62969	3.799	0.43945	10.38375	3.799	0.115056	9.6371
3.8	4.20342	27.61266	3.8	2.03962	16.67029	3.8	0.968	11.63384	3.8	0.44	10.38571	3.8	0.1152	9.6376
3.801	4.20958	27.65447	3.801	2.04347	16.7129	3.801	0.96921	11.63799	3.801	0.44055	10.38767	3.801	0.115344	9.6381
3.802	4.21576	27.69628	3.802	2.04734	16.75551	3.802	0.97042	11.64214	3.802	0.4411	10.38963	3.802	0.115488	9.6386
3.803	4.22194	27.73809	3.803	2.05121	16.79812	3.803	0.97163	11.64629	3.803	0.44165	10.39159	3.803	0.115632	9.6391
3.804	4.22812	27.7799	3.804	2.05509	16.84073	3.804	0.97284	11.65044	3.804	0.4422	10.39355	3.804	0.115776	9.6396
3.805	4.23432	27.82171	3.805	2.05898	16.88334	3.805	0.97405	11.65459	3.805	0.44275	10.39551	3.805	0.11592	9.6401
3.806	4.24052	27.86352	3.806	2.06288	16.92595	3.806	0.97526	11.65874	3.806	0.4433	10.39747	3.806	0.116064	9.6406
3.807	4.24673	27.90533	3.807	2.06679	16.96855	3.807	0.97647	11.66289	3.807	0.44385	10.39943	3.807	0.116208	9.6411
3.808	4.25295	27.94714	3.808	2.07071	17.01116	3.808	0.97768	11.66704	3.808	0.4444	10.40139	3.808	0.116352	9.6416
3.809	4.25918	27.98896	3.809	2.07464	17.05377	3.809	0.97889	11.67119	3.809	0.44495	10.40335	3.809	0.116496	9.6421
3.81	4.26541	28.03077	3.81	2.07858	17.09637	3.81	0.9801	11.67534	3.81	0.4455	10.40531	3.81	0.11664	9.6426
3.811	4.27165	28.07258	3.811	2.08252	17.13898	3.811	0.98131	11.67949	3.811	0.44605	10.40727	3.811	0.116784	9.6431
3.812	4.2779	28.1144	3.812	2.08648	17.18159	3.812	0.98252	11.68364	3.812	0.4466	10.40923	3.812	0.116928	9.6436
3.813	4.28416	28.15622	3.813	2.09044	17.22419	3.813	0.98373	11.68779	3.813	0.44715	10.41119	3.813	0.117072	9.6441
3.814	4.29042	28.19803	3.814	2.09442	17.2668	3.814	0.98494	11.69194	3.814	0.4477	10.41315	3.814	0.117216	9.6446
3.815	4.29669	28.23985	3.815	2.0984	17.3094	3.815	0.98615	11.69609	3.815	0.44825	10.41511	3.815	0.11736	9.6451
3.816	4.30297	28.28167	3.816	2.10239	17.35201	3.816	0.98736	11.70024	3.816	0.4488	10.41707	3.816	0.117504	9.6456
3.817	4.30926	28.32348	3.817	2.10639	17.39461	3.817	0.98857	11.70439	3.817	0.44935	10.41903	3.817	0.117648	9.6461
3.818	4.31556	28.3653	3.818	2.1104	17.43722	3.818	0.98978	11.70854	3.818	0.4499	10.42099	3.818	0.117792	9.6466
3.819	4.32186	28.40712	3.819	2.11442	17.47982	3.819	0.99099	11.71269	3.819	0.45045	10.42295	3.819	0.117936	9.6471
3.82	4.32817	28.44894	3.82	2.11845	17.52243	3.82	0.9922	11.71684	3.82	0.451	10.42491	3.82	0.11808	9.6476
3.821	4.33449	28.49077	3.821	2.12248	17.56503	3.821	0.99341	11.72099	3.821	0.45155	10.42687	3.821	0.118224	9.6481
3.822	4.34081	28.53259	3.822	2.12653	17.60764	3.822	0.99462	11.72514	3.822	0.4521	10.42883	3.822	0.118368	9.6486
3.823	4.34715	28.57441	3.823	2.13059	17.65024	3.823	0.99583	11.72929	3.823	0.45265	10.43079	3.823	0.118512	9.6491
3.824	4.35349	28.61624	3.824	2.13465	17.69285	3.824	0.99704	11.73344	3.824	0.4532	10.43275	3.824	0.118656	9.6496
3.825	4.35984	28.65806	3.825	2.13872	17.73545	3.825	0.99825	11.73759	3.825	0.45375	10.43471	3.825	0.1188	9.6501
3.826	4.36619	28.69989	3.826	2.1428	17.77805	3.826	0.99946	11.74174	3.826	0.4543	10.43667	3.826	0.118944	9.6506
3.827	4.37256	28.74171	3.827	2.1469	17.82066	3.827	1.00067	11.74589	3.827	0.45485	10.43863	3.827	0.119088	9.6511
3.828	4.37893	28.78354	3.828	2.151	17.86326	3.828	1.00188	11.75004	3.828	0.4554	10.44059	3.828	0.119232	9.6516
3.829	4.38531	28.82537	3.829	2.15511	17.90587	3.829	1.00309	11.75419	3.829	0.45595	10.44255	3.829	0.119376	9.6521
3.83	4.3917	28.8672	3.83	2.15922	17.94847	3.83	1.0043	11.75834	3.83	0.4565	10.44451	3.83	0.11952	9.6526
3.831	4.39809	28.90903	3.831	2.16335	17.99107	3.831	1.00551	11.76249	3.831	0.45705	10.44647	3.831	0.119664	9.6531
3.832	4.40449	28.95086	3.832	2.16749	18.03368	3.832	1.00672	11.76664	3.832	0.4576	10.44843	3.832	0.119808	9.6536
3.833	4.4109	28.99269	3.833	2.17163	18.07628	3.833	1.00793	11.77079	3.833	0.45815	10.45039	3.833	0.119952	9.6541
3.834	4.41732	29.03452	3.834	2.17578	18.11888	3.834	1.00914	11.77494	3.834	0.4587	10.45235	3.834	0.120096	9.6546
3.835	4.42374	29.07635	3.835	2.17995	18.16149	3.835	1.01035	11.77909	3.835	0.45925	10.45431	3.835	0.12024	9.6551
3.836	4.43017	29.11819	3.836	2.18412	18.20409	3.836	1.01156	11.78324	3.836	0.4598	10.45627	3.836	0.120384	9.6556
3.837	4.43661	29.16002	3.837	2.1883	18.2467	3.837	1.01277	11.78739	3.837	0.46035	10.45823	3.837	0.120528	9.6561
3.838	4.44306	29.20186	3.838	2.19249	18.2893	3.838	1.01398	11.79154	3.838	0.4609	10.46019	3.838	0.120672	9.6566
3.839	4.44952	29.2437	3.839	2.19669	18.33191	3.839	1.01519	11.79569	3.839	0.46145	10.46215	3.839	0.120816	9.6571
3.84	4.45598	29.28554	3.84	2.2009	18.37451	3.84	1.0164	11.79984	3.84	0.462	10.46411	3.84	0.12096	9.6576
3.841	4.46245	29.32737	3.841	2.20511	18.41711	3.841	1.01761	11.80399	3.841	0.46255	10.46607	3.841	0.121104	9.6581
3.842	4.46893	29.36921	3.842	2.20934	18.45972	3.842	1.01882	11.80814	3.842	0.4631	10.46803	3.842	0.121248	9.6586
3.843	4.47541	29.41106	3.843	2.21357	18.50232	3.843	1.02003	11.81229	3.843	0.46365	10.46999	3.843	0.121392	9.6591
3.844	4.4819	29.4529	3.844	2.21781	18.54493	3.844	1.02124	11.81644	3.844	0.4642	10.47195	3.844	0.121536	9.6596
3.845	4.4884	29.49474	3.845	2.22207	18.58753	3.845	1.02245	11.82059	3.845	0.46475	10.47391	3.845	0.12168	9.6601
3.846	4.49491	29.53659	3.846	2.22633	18.63014	3.846	1.02366	11.82474	3.846	0.4653	10.47587	3.846	0.121824	9.6606
3.847	4.50142	29.57843	3.847	2.2306	18.67275	3.847	1.02487	11.82889	3.847	0.46585	10.47783	3.847	0.121968	9.6611
3.848	4.50795	29.62028	3.848	2.23487	18.71535	3.848	1.02608	11.83304	3.848	0.4664	10.47979	3.848	0.122112	9.6616
3.849	4.51448	29.66212	3.849	2.23916	18.75796	3.849	1.02729	11.83719	3.849	0.46695	10.48175	3.849	0.122256	9.6621
3.85	4.52101	29.70397	3.85	2.24346	18.80056	3.85	1.0285	11.84134	3.85	0.4675	10.48371	3.85	0.1224	9.6626
3.851	4.52756	29.74582	3.851	2.24776	18.84317	3.851	1.02971	11.84549	3.851	0.46805	10.48567	3.851	0.122544	9.6631
3.852	4.53411	29.78767	3.852	2.25208	18.88578	3.852	1.03092	11.84964	3.852	0.4686	10.48763	3.852	0.122688	9.6636
3.853	4.54067	29.82952	3.853	2.2564	18.92838	3.853	1.03213	11.85379	3.853	0.46915	10.48959	3.853	0.122832	9.6641
3.854	4.54724	29.87138	3.854	2.26073	18.97099	3.854	1.03334	11.85794	3.854	0.4697	10.49155	3.854	0.122976	9.6646
3.855	4.55381	29.91323	3.855	2.26507	19.0136	3.855	1.03455	11.86209	3.855	0.47025	10.49351	3.855	0.12312	9.6651
3.856	4.56039	29.95508	3.856	2.26942	19.05621	3.856	1.03576	11.86624	3.856	0.4708	10.49547	3.856	0.123264	9.6656
3.857	4.56698	29.99694	3.857	2.27378	19.09882	3.857	1.03697	11.87039	3.857	0.47135	10.49743	3.857	0.123408	9.6661
3.858	4.57358	30.0388	3.858	2.27814	19.14143	3.858	1.03818	11.87454	3.858	0.4719	10.49939	3.858	0.123552	9.6666
3.859	4.58019	30.08066	3.859	2.28252	19.18403	3.859	1.03939	11.87869	3.859	0.47245	10.50135	3.859	0.123696	9.6671
3.86	4.5868	30.12251	3.86	2.2869	19.22664	3.86	1.0406	11.88284	3.86	0.473	10.50331	3.86	0.12384	9.6676
3.861	4.59342	30.16438	3.861	2.2913	19.26925	3.861	1.04181	11.88699	3.861	0.47355	10.50527	3.861	0.123984	9.6681
3.862	4.60004	30.20624	3.862	2.2957	19.31186	3.862	1.04302	11.89114	3.862	0.4741	10.50723	3.862	0.124128	9.6686
3.863	4.60668	30.2481	3.863	2.30011	19.35448	3.863	1.04423	11.89529	3.863	0.47465	10.50919	3.863	0.124272	9.6691
3.864	4.61332	30.28996	3.864	2.30453	19.39709	3.864	1.04544	11.89944	3.864	0.4752	10.51115	3.864	0.124416	9.6696
3.865	4.61997	30.33183	3.865	2.30896	19.4397	3.865	1.04665	11.90359	3.865	0.47575	10.51311	3.865	0.12456	9.6701
3.866	4.62662	30.3737	3.866	2.31339	19.48231	3.866	1.04786	11.90774	3.866	0.4763	10.51507	3.866	0.124704	9.6706
3.867	4.63329	30.41556	3.867	2.31784	19.52492	3.867	1.04907	11.91189	3.867	0.47685	10.51703	3.867	0.124848	9.6711
3.868	4.63996	30.45743	3.868	2.32229	19.56754	3.868	1.05028	11.91604	3.868	0.4774	10.51899	3.868	0.124992	9.6716
3.869	4.64664	30.4993	3.869	2.32676	19.61015	3.869	1.05149	11.92019	3.869	0.47795	10.52095	3.869	0.125136	9.6721
3.87	4.65332	30.54117	3.87	2.33123	19.65276	3.87	1.0527	11.92434	3.87	0.4785	10.52291	3.87	0.12528	9.6726
3.871	4.66001	30.58305	3.871	2.33571	19.69538	3.871	1.05391	11.92849	3.871	0.47905	10.52487	3.871	0.125424	9.6731
3.872	4.66672	30.62492	3.872	2.3402	19.73799	3.872	1.05512	11.93264	3.872	0.4796	10.52683	3.872	0.125568	9.6736
3.873	4.67342	30.66679	3.873	2.34469	19.78061	3.873	1.05633	11.93679	3.873	0.48015	10.52879	3.873	0.125712	9.6741
3.874	4.68014	30.70867	3.874	2.3492	19.82323	3.874	1.05754	11.94094	3.874	0.4807	10.53075	3.874	0.125856	9.6746
3.875	4.68686	30.75055	3.875	2.35371	19.86584	3.875	1.05875	11.94509	3.875	0.48125	10.53271	3.875	0.126	9.6751
3.876	4.69359	30.79243	3.876	2.35824	19.90846	3.876	1.05996	11.94924	3.876	0.4818	10.53467	3.876	0.126144	9.6756
3.877	4.70033	30.83431	3.877	2.36277	19.95108	3.877	1.06117	11.95339	3.877	0.48235	10.53663	3.877	0.126288	9.6761
3.878	4.70707	30.87619	3.878	2.36731	19.9937	3.878	1.06238	11.95754	3.878	0.4829	10.53859	3.878	0.126432	9.6766
3.879	4.71382	30.91807	3.879	2.37186	20.03632	3.879	1.06359	11.96169	3.879	0.48345	10.54055	3.879	0.126576	9.6771
3.88	4.72058	30.95996	3.88	2.37642	20.07894	3.88	1.0648	11.96584	3.88	0.484	10.54251	3.88	0.12672	9.6776
3.881	4.72735	31.00184	3.881	2.38099	20.12156	3.881	1.06601	11.96999	3.881	0.48455	10.54447	3.881	0.126864	9.6781
3.882	4.73412	31.04373	3.882	2.38556	20.16418	3.882	1.06722	11.97414	3.882	0.4851	10.54643	3.882	0.127008	9.6786
3.883	4.7409	31.08562	3.883	2.39015	20.2068	3.883	1.06843	11.97829	3.883	0.48565	10.54839	3.883	0.127152	9.6791
3.884	4.74769	31.12751	3.884	2.39474	20.24942	3.884	1.06964	11.98244	3.884	0.4862	10.55035	3.884	0.127296	9.6796
3.885	4.75449	31.1694	3.885	2.39934	20.29205	3.885	1.07085	11.98659	3.885	0.48675	10.55231	3.885	0.12744	9.6801
3.886	4.76129	31.21129	3.886	2.40395	20.33467	3.886	1.07206	11.99074	3.886	0.4873	10.55427	3.886	0.127584	9.6806
3.887	4.7681	31.25318	3.887	2.40857	20.37729	3.887	1.07327	11.99489	3.887	0.48785	10.55623	3.887	0.127728	9.6811
3.888	4.77492	31.29508	3.888	2.41319	20.41992	3.888	1.08064	11.99931	3.888	0.4884	10.55819	3.888	0.127872	9.6816
3.889	4.78174	31.33698	3.889	2.41783	20.46255	3.889	1.08346	12.04236	3.889	0.48895	10.56015	3.889	0.128016	9.6821
3.89	4.78857	31.37887	3.89	2.42247	20.50517	3.89	1.08628	12.0854	3.89	0.4895	10.56211	3.89	0.12816	9.6826
3.891	4.79541	31.42077	3.891	2.42713	20.5478	3.891	1.08911	12.12845	3.891	0.49005	10.56407	3.891	0.128304	9.6831
3.892	4.80226	31.46268	3.892	2.43179	20.59043	3.892	1.09195	12.1715	3.892	0.4906	10.56603	3.892	0.128448	9.6836
3.893	4.80911	31.50458	3.893	2.43646	20.63306	3.893	1.0948	12.21455	3.893	0.49115	10.56799	3.893	0.128592	9.6841
3.894	4.81597	31.54648	3.894	2.44114	20.67569	3.894	1.09767	12.25759	3.894	0.4917	10.56995	3.894	0.128736	9.6846
3.895	4.82284	31.58839	3.895	2.44582	20.71832	3.895	1.10054	12.30064	3.895	0.49225	10.57191	3.895	0.12888	9.6851
3.896	4.82972	31.63029	3.896	2.45052	20.76095	3.896	1.10342	12.34368	3.896	0.4928	10.57387	3.896	0.129024	9.6856
3.897	4.8366	31.6722	3.897	2.45522	20.80358	3.897	1.10631	12.38673	3.897	0.49335	10.57583	3.897	0.129168	9.6861
3.898	4.84349	31.71411	3.898	2.45994	20.84622	3.898	1.10921	12.42978	3.898	0.4939	10.57779	3.898	0.129312	9.6866
3.899	4.85039	31.75602	3.899	2.46466	20.88885	3.899	1.11211	12.47282	3.899	0.49445	10.57975	3.899	0.129456	9.6871
3.9	4.85729	31.79794	3.9	2.46939	20.93149	3.9	1.11503	12.51587	3.9	0.495	10.58171	3.9	0.1296	9.6876
3.901	4.8642	31.83985	3.901	2.47413	20.97412	3.901	1.11796	12.55891	3.901	0.49555	10.58367	3.901	0.129744	9.6881
3.902	4.87112	31.88177	3.902	2.47887	21.01676	3.902	1.1209	12.60196	3.902	0.4961	10.58563	3.902	0.129888	9.6886
3.903	4.87805	31.92368	3.903	2.48363	21.0594	3.903	1.12385	12.645	3.903	0.49665	10.58759	3.903	0.130032	9.6891
3.904	4.88498	31.9656	3.904	2.48839	21.10204	3.904	1.1268	12.68805	3.904	0.4972	10.58955	3.904	0.130176	9.6896
3.905	4.89192	32.00752	3.905	2.49316	21.14467	3.905	1.12977	12.73109	3.905	0.49775	10.59151	3.905	0.13032	9.6901
3.906	4.89887	32.04944	3.906	2.49794	21.18732	3.906	1.13274	12.77414	3.906	0.4983	10.59347	3.906	0.130464	9.6906
3.907	4.90582	32.09137	3.907	2.50273	21.22996	3.907	1.13573	12.81718	3.907	0.49885	10.59543	3.907	0.130608	9.6911
3.908	4.91279	32.13329	3.908	2.50753	21.2726	3.908	1.13872	12.86023	3.908	0.4994	10.59739	3.908	0.130752	9.6916
3.909	4.91975	32.17522	3.909	2.51234	21.31524	3.909	1.14173	12.90327	3.909	0.49995	10.59935	3.909	0.130896	9.6921
3.91	4.92673	32.21715	3.91	2.51715	21.35789	3.91	1.14474	12.94631	3.91	0.5005	10.60131	3.91	0.13104	9.6926
3.911	4.93371	32.25908	3.911	2.52197	21.40053	3.911	1.14776	12.98936	3.911	0.50105	10.60327	3.911	0.131184	9.6931
3.912	4.9407	32.30101	3.912	2.5268	21.44318	3.912	1.1508	13.0324	3.912	0.5016	10.60523	3.912	0.131328	9.6936
3.913	4.9477	32.34294	3.913	2.53164	21.48583	3.913	1.15384	13.07545	3.913	0.50215	10.60719	3.913	0.131472	9.6941
3.914	4.95471	32.38488	3.914	2.53649	21.52848	3.914	1.15689	13.11849	3.914	0.5027	10.60915	3.914	0.131616	9.6946
3.915	4.96172	32.42681	3.915	2.54135	21.57113	3.915	1.15995	13.16154	3.915	0.50325	10.61111	3.915	0.13176	9.6951
3.916	4.96874	32.46875	3.916	2.54621	21.61378	3.916	1.16302	13.20458	3.916	0.5038	10.61307	3.916	0.131904	9.6956
3.917	4.97576	32.51069	3.917	2.55109	21.65643	3.917	1.1661	13.24763	3.917	0.50435	10.61503	3.917	0.132048	9.6961
3.918	4.9828	32.55263	3.918	2.55597	21.69908	3.918	1.16919	13.29067	3.918	0.5049	10.61699	3.918	0.132192	9.6966
3.919	4.98984	32.59458	3.919	2.56086	21.74174	3.919	1.17229	13.33372	3.919	0.50545	10.61895	3.919	0.132336	9.6971
3.92	4.99688	32.63652	3.92	2.56576	21.78439	3.92	1.1754	13.37676	3.92	0.506	10.62091	3.92	0.13248	9.6976
3.921	5.00394	32.67847	3.921	2.57066	21.82705	3.921	1.17851	13.41981	3.921	0.50655	10.62287	3.921	0.132624	9.6981
3.922	5.011	32.72042	3.922	2.57558	21.86971	3.922	1.18164	13.46285	3.922	0.5071	10.62483	3.922	0.132768	9.6986
3.923	5.01807	32.76237	3.923	2.5805	21.91236	3.923	1.18478	13.5059	3.923	0.50765	10.62679	3.923	0.132912	9.6991
3.924	5.02514	32.80432	3.924	2.58543	21.95502	3.924	1.18792	13.54894	3.924	0.5082	10.62875	3.924	0.133056	9.6996
3.925	5.03223	32.84627	3.925	2.59038	21.99769	3.925	1.19108	13.59199	3.925	0.50875	10.63071	3.925	0.1332	9.7001
3.926	5.03932	32.88823	3.926	2.59532	22.04035	3.926	1.19424	13.63504	3.926	0.5093	10.63267	3.926	0.133344	9.7006
3.927	5.04641	32.93018	3.927	2.60028	22.08301	3.927	1.19741	13.67808	3.927	0.50985	10.63463	3.927	0.133488	9.7011
3.928	5.05352	32.97214	3.928	2.60525	22.12568	3.928	1.2006	13.72113	3.928	0.5104	10.63659	3.928	0.133632	9.7016
3.929	5.06063	33.0141	3.929	2.61022	22.16834	3.929	1.20379	13.76418	3.929	0.51095	10.63855	3.929	0.133776	9.7021
3.93	5.06775	33.05606	3.93	2.6152	22.21101	3.93	1.20699	13.80723	3.93	0.5115	10.64051	3.93	0.13392	9.7026
3.931	5.07487	33.09803	3.931	2.62019	22.25368	3.931	1.2102	13.85027	3.931	0.51205	10.64247	3.931	0.134064	9.7031
3.932	5.082	33.13999	3.932	2.62519	22.29635	3.932	1.21342	13.89332	3.932	0.5126	10.64443	3.932	0.134208	9.7036
3.933	5.08914	33.18196	3.933	2.6302	22.33902	3.933	1.21665	13.93637	3.933	0.51315	10.64639	3.933	0.134352	9.7041
3.934	5.09629	33.22393	3.934	2.63521	22.38169	3.934	1.21989	13.97942	3.934	0.5137	10.64835	3.934	0.134496	9.7046
3.935	5.10344	33.2659	3.935	2.64024	22.42436	3.935	1.22314	14.02247	3.935	0.51425	10.65031	3.935	0.13464	9.7051
3.936	5.1106	33.30787	3.936	2.64527	22.46704	3.936	1.2264	14.06552	3.936	0.5148	10.65227	3.936	0.134784	9.7056
3.937	5.11777	33.34985	3.937	2.65031	22.50971	3.937	1.22966	14.10857	3.937	0.51535	10.65423	3.937	0.134928	9.7061
3.938	5.12495	33.39183	3.938	2.65536	22.55239	3.938	1.23294	14.15162	3.938	0.5159	10.65619	3.938	0.135072	9.7066
3.939	5.13213	33.4338	3.939	2.66041	22.59507	3.939	1.23622	14.19467	3.939	0.51645	10.65815	3.939	0.135216	9.7071
3.94	5.13932	33.47578	3.94	2.66548	22.63775	3.94	1.23952	14.23772	3.94	0.517	10.66011	3.94	0.13536	9.7076
3.941	5.14651	33.51777	3.941	2.67055	22.68043	3.941	1.24282	14.28077	3.941	0.51755	10.66207	3.941	0.135504	9.7081
3.942	5.15371	33.55975	3.942	2.67563	22.72312	3.942	1.24613	14.32382	3.942	0.5181	10.66403	3.942	0.135648	9.7086
3.943	5.16092	33.60174	3.943	2.68072	22.7658	3.943	1.24946	14.36687	3.943	0.51865	10.66599	3.943	0.135792	9.7091
3.944	5.16814	33.64372	3.944	2.68582	22.80849	3.944	1.25279	14.40993	3.944	0.5192	10.66795	3.944	0.135936	9.7096
3.945	5.17536	33.68571	3.945	2.69093	22.85117	3.945	1.25613	14.45298	3.945	0.51975	10.66991	3.945	0.13608	9.7101
3.946	5.18259	33.7277	3.946	2.69604	22.89386	3.946	1.25948	14.49603	3.946	0.5203	10.67187	3.946	0.136224	9.7106
3.947	5.18983	33.7697	3.947	2.70117	22.93655	3.947	1.26284	14.53909	3.947	0.52085	10.67383	3.947	0.136368	9.7111
3.948	5.19708	33.81169	3.948	2.7063	22.97924	3.948	1.26621	14.58214	3.948	0.5214	10.67579	3.948	0.136512	9.7116
3.949	5.20433	33.85369	3.949	2.71144	23.02193	3.949	1.26958	14.6252	3.949	0.52195	10.67775	3.949	0.136656	9.7121
3.95	5.21159	33.89569	3.95	2.71658	23.06463	3.95	1.27297	14.66826	3.95	0.5225	10.67971	3.95	0.1368	9.7126
3.951	5.21885	33.93769	3.951	2.72174	23.10732	3.951	1.27637	14.71131	3.951	0.52305	10.68167	3.951	0.136944	9.7131
3.952	5.22612	33.97969	3.952	2.7269	23.15002	3.952	1.27977	14.75437	3.952	0.5236	10.68363	3.952	0.137088	9.7136
3.953	5.2334	34.0217	3.953	2.73207	23.19272	3.953	1.28318	14.79743	3.953	0.52415	10.68559	3.953	0.137232	9.7141
3.954	5.24069	34.06371	3.954	2.73725	23.23542	3.954	1.28661	14.84049	3.954	0.5247	10.68755	3.954	0.137376	9.7146
3.955	5.24798	34.10572	3.955	2.74244	23.27812	3.955	1.29004	14.88355	3.955	0.52525	10.68951	3.955	0.13752	9.7151
3.956	5.25528	34.14773	3.956	2.74764	23.32083	3.956	1.29348	14.92661	3.956	0.5258	10.69147	3.956	0.137664	9.7156
3.957	5.26259	34.18974	3.957	2.75284	23.36353	3.957	1.29693	14.96967	3.957	0.52635	10.69343	3.957	0.137808	9.7161

3.958	5.2699	34.23175	3.958	2.75806	23.40624	3.958	1.30039	15.01273	3.958	0.5269	10.69539	3.958	0.137952	9.7166
3.959	5.27722	34.27377	3.959	2.76328	23.44895	3.959	1.30386	15.05579	3.959	0.52745	10.69735	3.959	0.138096	9.7171
3.96	5.28455	34.31579	3.96	2.76851	23.49165	3.96	1.30734	15.09885	3.96	0.528	10.69931	3.96	0.13824	9.7176
3.961	5.29188	34.35781	3.961	2.77374	23.53437	3.961	1.31083	15.14192	3.961	0.52855	10.70127	3.961	0.138384	9.7181
3.962	5.29923	34.39983	3.962	2.77899	23.57708	3.962	1.31432	15.18498	3.962	0.5291	10.70323	3.962	0.138528	9.7186
3.963	5.30657	34.44186	3.963	2.78424	23.61979	3.963	1.31783	15.22804	3.963	0.52965	10.70519	3.963	0.138672	9.7191
3.964	5.31393	34.48389	3.964	2.7895	23.66251	3.964	1.32134	15.27111	3.964	0.5302	10.70715	3.964	0.138816	9.7196
3.965	5.32129	34.52592	3.965	2.79477	23.70523	3.965	1.32487	15.31418	3.965	0.53075	10.70911	3.965	0.13896	9.7201
3.966	5.32866	34.56795	3.966	2.80005	23.74795	3.966	1.3284	15.35724	3.966	0.5313	10.71107	3.966	0.139104	9.7206
3.967	5.33604	34.60998	3.967	2.80534	23.79067	3.967	1.33194	15.40031	3.967	0.53185	10.71303	3.967	0.139248	9.7211
3.968	5.34342	34.65202	3.968	2.81063	23.83339	3.968	1.33549	15.44338	3.968	0.5324	10.71499	3.968	0.139392	9.7216
3.969	5.35081	34.69405	3.969	2.81593	23.87611	3.969	1.33905	15.48645	3.969	0.53295	10.71695	3.969	0.139536	9.7221
3.97	5.35821	34.73609	3.97	2.82124	23.91884	3.97	1.34262	15.52952	3.97	0.5335	10.71891	3.97	0.13968	9.7226
3.971	5.36561	34.77813	3.971	2.82656	23.96157	3.971	1.3462	15.57259	3.971	0.53405	10.72087	3.971	0.139824	9.7231
3.972	5.37302	34.82018	3.972	2.83189	24.0043	3.972	1.34979	15.61567	3.972	0.5346	10.72283	3.972	0.139968	9.7236
3.973	5.38044	34.86222	3.973	2.83722	24.04703	3.973	1.35338	15.65874	3.973	0.53515	10.72479	3.973	0.140112	9.7241
3.974	5.38786	34.90427	3.974	2.84256	24.08976	3.974	1.35699	15.70181	3.974	0.5357	10.72675	3.974	0.140256	9.7246
3.975	5.39529	34.94632	3.975	2.84792	24.1325	3.975	1.3606	15.74489	3.975	0.53625	10.72871	3.975	0.1404	9.7251
3.976	5.40273	34.98837	3.976	2.85327	24.17523	3.976	1.36423	15.78796	3.976	0.5368	10.73067	3.976	0.140544	9.7256
3.977	5.41017	35.03043	3.977	2.85864	24.21797	3.977	1.36786	15.83104	3.977	0.53735	10.73263	3.977	0.140688	9.7261
3.978	5.41763	35.07249	3.978	2.86402	24.26071	3.978	1.3715	15.87412	3.978	0.5379	10.73459	3.978	0.140832	9.7266
3.979	5.42508	35.11454	3.979	2.8694	24.30345	3.979	1.37515	15.9172	3.979	0.53845	10.73655	3.979	0.140976	9.7271
3.98	5.43255	35.1566	3.98	2.87479	24.34619	3.98	1.37881	15.96028	3.98	0.539	10.73851	3.98	0.14112	9.7276
3.981	5.44002	35.19867	3.981	2.88019	24.38894	3.981	1.38248	16.00336	3.981	0.53955	10.74047	3.981	0.141264	9.7281
3.982	5.4475	35.24073	3.982	2.88559	24.43169	3.982	1.38615	16.04644	3.982	0.5401	10.74243	3.982	0.141408	9.7286
3.983	5.45498	35.2828	3.983	2.89101	24.47444	3.983	1.38984	16.08952	3.983	0.54065	10.74439	3.983	0.141552	9.7291
3.984	5.46248	35.32487	3.984	2.89643	24.51719	3.984	1.39353	16.13261	3.984	0.5412	10.74635	3.984	0.141696	9.7296
3.985	5.46997	35.36694	3.985	2.90186	24.55994	3.985	1.39724	16.17569	3.985	0.54175	10.74831	3.985	0.14184	9.7301
3.986	5.47748	35.40902	3.986	2.9073	24.60269	3.986	1.40095	16.21878	3.986	0.5423	10.75027	3.986	0.141984	9.7306
3.987	5.48499	35.45109	3.987	2.91275	24.64545	3.987	1.40467	16.26187	3.987	0.54285	10.75223	3.987	0.142128	9.7311
3.988	5.49251	35.49317	3.988	2.9182	24.68821	3.988	1.4084	16.30496	3.988	0.5434	10.75419	3.988	0.142272	9.7316
3.989	5.50004	35.53525	3.989	2.92367	24.73097	3.989	1.41214	16.34805	3.989	0.54395	10.75615	3.989	0.142416	9.7321
3.99	5.50757	35.57734	3.99	2.92914	24.77373	3.99	1.41589	16.39114	3.99	0.5445	10.75811	3.99	0.14256	9.7326
3.991	5.51511	35.61942	3.991	2.93462	24.81649	3.991	1.41965	16.43423	3.991	0.54505	10.76007	3.991	0.142704	9.7331
3.992	5.52266	35.66151	3.992	2.9401	24.85926	3.992	1.42342	16.47732	3.992	0.5456	10.76203	3.992	0.142848	9.7336
3.993	5.53021	35.7036	3.993	2.9456	24.90203	3.993	1.42719	16.52042	3.993	0.54615	10.76399	3.993	0.142992	9.7341
3.994	5.53777	35.74569	3.994	2.9511	24.9448	3.994	1.43098	16.56351	3.994	0.5467	10.76595	3.994	0.143136	9.7346
3.995	5.54534	35.78778	3.995	2.95661	24.98757	3.995	1.43477	16.60661	3.995	0.54725	10.76791	3.995	0.14328	9.7351
3.996	5.55291	35.82988	3.996	2.96213	25.03034	3.996	1.43857	16.64971	3.996	0.5478	10.76987	3.996	0.143424	9.7356
3.997	5.56049	35.87198	3.997	2.96766	25.07312	3.997	1.44238	16.6928	3.997	0.54835	10.77183	3.997	0.143568	9.7361
3.998	5.56808	35.91408	3.998	2.97319	25.1159	3.998	1.4462	16.73591	3.998	0.5489	10.77379	3.998	0.143712	9.7366
3.999	5.57567	35.95619	3.999	2.97873	25.15868	3.999	1.45003	16.77901	3.999	0.54945	10.77575	3.999	0.143856	9.7371
4	5.58327	35.99829	4	2.98428	25.20146	4	1.45387	16.82211	4	0.55	10.77771	4	0.144	9.7376
4.001	5.59088	36.0404	4.001	2.98984	25.24424	4.001	1.45772	16.86521	4.001	0.55055	10.77967	4.001	0.144144	9.7381
4.002	5.59849	36.08251	4.002	2.99541	25.28703	4.002	1.46157	16.90832	4.002	0.5511	10.78163	4.002	0.144288	9.7386
4.003	5.60611	36.12462	4.003	3.00098	25.32982	4.003	1.46544	16.95143	4.003	0.55165	10.78359	4.003	0.144432	9.7391
4.004	5.61374	36.16674	4.004	3.00656	25.37261	4.004	1.46931	16.99453	4.004	0.5522	10.78555	4.004	0.144576	9.7396
4.005	5.62137	36.20886	4.005	3.01215	25.4154	4.005	1.47319	17.03764	4.005	0.55275	10.78751	4.005	0.14472	9.7401
4.006	5.62902	36.25098	4.006	3.01775	25.45819	4.006	1.47708	17.08075	4.006	0.5533	10.78947	4.006	0.144864	9.7406
4.007	5.63666	36.2931	4.007	3.02336	25.50099	4.007	1.48098	17.12387	4.007	0.55385	10.79143	4.007	0.145008	9.7411
4.008	5.64432	36.33522	4.008	3.02897	25.54379	4.008	1.48489	17.16698	4.008	0.5544	10.79339	4.008	0.145152	9.7416
4.009	5.65198	36.37735	4.009	3.03459	25.58659	4.009	1.48881	17.21009	4.009	0.55495	10.79535	4.009	0.145296	9.7421
4.01	5.65965	36.41948	4.01	3.04022	25.62939	4.01	1.49273	17.25321	4.01	0.5555	10.79731	4.01	0.14544	9.7426
4.011	5.66732	36.46161	4.011	3.04586	25.67219	4.011	1.49667	17.29633	4.011	0.55605	10.79927	4.011	0.145584	9.7431
4.012	5.675	36.50375	4.012	3.0515	25.715	4.012	1.50061	17.33945	4.012	0.5566	10.80123	4.012	0.145728	9.7436
4.013	5.68269	36.54588	4.013	3.05716	25.75781	4.013	1.50456	17.38257	4.013	0.55715	10.80319	4.013	0.145872	9.7441
4.014	5.69038	36.58802	4.014	3.06282	25.80062	4.014	1.50852	17.42569	4.014	0.5577	10.80515	4.014	0.146016	9.7446
4.015	5.69808	36.63017	4.015	3.06849	25.84343	4.015	1.51249	17.46881	4.015	0.55825	10.80711	4.015	0.14616	9.7451
4.016	5.70579	36.67231	4.016	3.07416	25.88625	4.016	1.51647	17.51194	4.016	0.5588	10.80907	4.016	0.146304	9.7456
4.017	5.7135	36.71446	4.017	3.07985	25.92906	4.017	1.52046	17.55506	4.017	0.56243	10.8158	4.017	0.146448	9.7461
4.018	5.72122	36.75661	4.018	3.08554	25.97188	4.018	1.52446	17.59819	4.018	0.56499	10.85908	4.018	0.146592	9.7466
4.019	5.72895	36.79876	4.019	3.09124	26.01471	4.019	1.52846	17.64132	4.019	0.56755	10.90237	4.019	0.146736	9.7471
4.02	5.73669	36.84091	4.02	3.09695	26.05753	4.02	1.53247	17.68445	4.02	0.57013	10.94566	4.02	0.14688	9.7476
4.021	5.74443	36.88307	4.021	3.10266	26.10036	4.021	1.5365	17.72759	4.021	0.57272	10.98895	4.021	0.147024	9.7481
4.022	5.75217	36.92523	4.022	3.10838	26.14319	4.022	1.54053	17.77072	4.022	0.57531	11.03224	4.022	0.147168	9.7486
4.023	5.75993	36.96739	4.023	3.11412	26.18602	4.023	1.54457	17.81386	4.023	0.57792	11.07554	4.023	0.147312	9.7491
4.024	5.76769	37.00955	4.024	3.11985	26.22885	4.024	1.54861	17.85699	4.024	0.58053	11.11883	4.024	0.147456	9.7496
4.025	5.77546	37.05172	4.025	3.1256	26.27168	4.025	1.55267	17.90013	4.025	0.58315	11.16212	4.025	0.1476	9.7501
4.026	5.78323	37.09389	4.026	3.13136	26.31452	4.026	1.55674	17.94327	4.026	0.58579	11.20541	4.026	0.147744	9.7506
4.027	5.79101	37.13606	4.027	3.13712	26.35736	4.027	1.56081	17.98641	4.027	0.58843	11.24871	4.027	0.147888	9.7511
4.028	5.7988	37.17823	4.028	3.14289	26.4002	4.028	1.5649	18.02956	4.028	0.59108	11.292	4.028	0.148032	9.7516
4.029	5.80659	37.22041	4.029	3.14867	26.44305	4.029	1.56899	18.0727	4.029	0.59374	11.3353	4.029	0.148176	9.7521
4.03	5.81439	37.26259	4.03	3.15445	26.4859	4.03	1.57309	18.11585	4.03	0.59641	11.3786	4.03	0.14832	9.7526
4.031	5.8222	37.30477	4.031	3.16024	26.52875	4.031	1.5772	18.159	4.031	0.59909	11.42189	4.031	0.148464	9.7531
4.032	5.83001	37.34696	4.032	3.16605	26.5716	4.032	1.58132	18.20215	4.032	0.60178	11.46519	4.032	0.148608	9.7536
4.033	5.83783	37.38914	4.033	3.17185	26.61445	4.033	1.58544	18.2453	4.033	0.60448	11.50849	4.033	0.148752	9.7541
4.034	5.84566	37.43133	4.034	3.17767	26.65731	4.034	1.58958	18.28845	4.034	0.60719	11.55179	4.034	0.148896	9.7546
4.035	5.85349	37.47352	4.035	3.1835	26.70017	4.035	1.59372	18.33161	4.035	0.60991	11.59509	4.035	0.14904	9.7551
4.036	5.86133	37.51572	4.036	3.18933	26.74303	4.036	1.59787	18.37477	4.036	0.61264	11.63839	4.036	0.149184	9.7556
4.037	5.86918	37.55792	4.037	3.19517	26.78589	4.037	1.60203	18.41793	4.037	0.61537	11.68169	4.037	0.149328	9.7561
4.038	5.87703	37.60012	4.038	3.20101	26.82876	4.038	1.6062	18.46109	4.038	0.61812	11.725	4.038	0.149472	9.7566
4.039	5.88489	37.64232	4.039	3.20687	26.87162	4.039	1.61038	18.50425	4.039	0.62087	11.7683	4.039	0.149616	9.7571
4.04	5.89276	37.68452	4.04	3.21273	26.9145	4.04	1.61457	18.54741	4.04	0.62364	11.81161	4.04	0.14976	9.7576
4.041	5.90063	37.72673	4.041	3.2186	26.95737	4.041	1.61877	18.59058	4.041	0.62641	11.85491	4.041	0.149904	9.7581
4.042	5.90851	37.76894	4.042	3.22448	27.00024	4.042	1.62297	18.63375	4.042	0.6292	11.89822	4.042	0.150048	9.7586
4.043	5.91639	37.81116	4.043	3.23037	27.04312	4.043	1.62718	18.67692	4.043	0.63199	11.94153	4.043	0.150192	9.7591
4.044	5.92428	37.85337	4.044	3.23626	27.086	4.044	1.6314	18.72009	4.044	0.63479	11.98483	4.044	0.150336	9.7596
4.045	5.93218	37.89559	4.045	3.24216	27.12889	4.045	1.63563	18.76326	4.045	0.6376	12.02814	4.045	0.15048	9.7601
4.046	5.94009	37.93781	4.046	3.24807	27.17177	4.046	1.63987	18.80644	4.046	0.64042	12.07145	4.046	0.150624	9.7606
4.047	5.948	37.98003	4.047	3.25399	27.21466	4.047	1.64412	18.84962	4.047	0.64325	12.11476	4.047	0.150768	9.7611
4.048	5.95592	38.02226	4.048	3.25991	27.25755	4.048	1.64838	18.8928	4.048	0.64609	12.15808	4.048	0.150912	9.7616
4.049	5.96384	38.06449	4.049	3.26585	27.30044	4.049	1.65264	18.93598	4.049	0.64894	12.20139	4.049	0.151056	9.7621
4.05	5.97177	38.10672	4.05	3.27179	27.34334	4.05	1.65691	18.97916	4.05	0.6518	12.24471	4.05	0.1512	9.7626
4.051	5.97971	38.14896	4.051	3.27773	27.38624	4.051	1.66119	19.02234	4.051	0.65466	12.28802	4.051	0.151344	9.7631
4.052	5.98766	38.19119	4.052	3.28369	27.42914	4.052	1.66548	19.06553	4.052	0.65754	12.33134	4.052	0.151488	9.7636
4.053	5.99561	38.23343	4.053	3.28965	27.47204	4.053	1.66978	19.10872	4.053	0.66043	12.37466	4.053	0.151632	9.7641
4.054	6.00357	38.27568	4.054	3.29562	27.51495	4.054	1.67409	19.15191	4.054	0.66332	12.41797	4.054	0.151776	9.7646
4.055	6.01153	38.29957	4.055	3.3016	27.55785	4.055	1.6784	19.1951	4.055	0.66623	12.46129	4.055	0.15192	9.7651
4.056	6.01949	38.32346	4.056	3.30758	27.60076	4.056	1.68273	19.2383	4.056	0.66914	12.50462	4.056	0.152064	9.7656
4.057	6.02746	38.34736	4.057	3.31358	27.64368	4.057	1.68706	19.28149	4.057	0.67206	12.54794	4.057	0.152208	9.7661
4.058	6.03542	38.37126	4.058	3.31958	27.68659	4.058	1.6914	19.32469	4.058	0.67499	12.59126	4.058	0.152352	9.7666
4.059	6.04339	38.39517	4.059	3.32559	27.72951	4.059	1.69575	19.36789	4.059	0.67793	12.63459	4.059	0.152496	9.7671
4.06	6.05136	38.41909	4.06	3.3316	27.77243	4.06	1.70011	19.4111	4.06	0.68088	12.67791	4.06	0.15264	9.7676
4.061	6.05934	38.44301	4.061	3.33763	27.81536	4.061	1.70448	19.4543	4.061	0.68384	12.72124	4.061	0.152784	9.7681
4.062	6.06731	38.46693	4.062	3.34366	27.85828	4.062	1.70885	19.49751	4.062	0.68681	12.76457	4.062	0.152928	9.7686
4.063	6.07529	38.49086	4.063	3.3497	27.90121	4.063	1.71323	19.54072	4.063	0.68979	12.8079	4.063	0.153072	9.7691
4.064	6.08327	38.5148	4.064	3.35574	27.94415	4.064	1.71763	19.58393	4.064	0.69278	12.85123	4.064	0.153216	9.7696
4.065	6.09125	38.53874	4.065	3.3618	27.98708	4.065	1.72203	19.62714	4.065	0.69577	12.89456	4.065	0.15336	9.7701
4.066	6.09923	38.56269	4.066	3.36786	28.03002	4.066	1.72644	19.67036	4.066	0.69878	12.93789	4.066	0.153504	9.7706
4.067	6.10722	38.58665	4.067	3.37393	28.07296	4.067	1.73085	19.71357	4.067	0.70179	12.98123	4.067	0.153648	9.7711
4.068	6.11521	38.61061	4.068	3.38001	28.1159	4.068	1.73528	19.75679	4.068	0.70482	13.02456	4.068	0.153792	9.7716
4.069	6.12319	38.63458	4.069	3.38609	28.15884	4.069	1.73971	19.80002	4.069	0.70785	13.0679	4.069	0.153936	9.7721
4.07	6.13119	38.65855	4.07	3.39218	28.20179	4.07	1.74416	19.84324	4.07	0.71089	13.11124	4.07	0.15408	9.7726
4.071	6.13918	38.68253	4.071	3.39828	28.24474	4.071	1.74861	19.88647	4.071	0.71395	13.15458	4.071	0.154224	9.7731
4.072	6.14717	38.70651	4.072	3.40439	28.2877	4.072	1.75307	19.92969	4.072	0.71701	13.19792	4.072	0.154368	9.7736
4.073	6.15517	38.73051	4.073	3.4105	28.33065	4.073	1.75754	19.97292	4.073	0.72008	13.24126	4.073	0.154512	9.7741
4.074	6.16317	38.7545	4.074	3.41663	28.37361	4.074	1.76201	20.01616	4.074	0.72315	13.28461	4.074	0.154656	9.7746
4.075	6.17117	38.7785	4.075	3.42275	28.41657	4.075	1.7665	20.05939	4.075	0.72624	13.32795	4.075	0.1548	9.7751
4.076	6.17917	38.80251	4.076	3.42889	28.45954	4.076	1.77099	20.10263	4.076	0.72934	13.3713	4.076	0.154944	9.7756
4.077	6.18718	38.82653	4.077	3.43504	28.50251	4.077	1.77549	20.14587	4.077	0.73244	13.41465	4.077	0.155088	9.7761
4.078	6.19518	38.85055	4.078	3.44119	28.54548	4.078	1.78	20.18911	4.078	0.73556	13.458	4.078	0.155232	9.7766
4.079	6.20319	38.87457	4.079	3.44735	28.58845	4.079	1.78452	20.23235	4.079	0.73868	13.50135	4.079	0.155376	9.7771
4.08	6.2112	38.8986	4.08	3.45352	28.63142	4.08	1.78905	20.2756	4.08	0.74182	13.5447	4.08	0.15552	9.7776
4.081	6.21922	38.92264	4.081	3.45969	28.6744	4.081	1.79358	20.31885	4.081	0.74496	13.58806	4.081	0.155664	9.7781
4.082	6.22723	38.94669	4.082	3.46587	28.71738	4.082	1.79813	20.3621	4.082	0.74811	13.63141	4.082	0.155808	9.7786
4.083	6.23525	38.97074	4.083	3.47206	28.76037	4.083	1.80268	20.40535	4.083	0.75127	13.67477	4.083	0.155952	9.7791
4.084	6.24327	38.99479	4.084	3.47826	28.80336	4.084	1.80724	20.44861	4.084	0.75444	13.71813	4.084	0.156096	9.7796
4.085	6.25129	39.01885	4.085	3.48447	28.84634	4.085	1.81181	20.49187	4.085	0.75762	13.76149	4.085	0.15624	9.7801
4.086	6.25931	39.04292	4.086	3.49068	28.88934	4.086	1.81638	20.53513	4.086	0.76081	13.80485	4.086	0.156384	9.7806
4.087	6.26733	39.06699	4.087	3.4969	28.93233	4.087	1.82097	20.57839	4.087	0.76401	13.84821	4.087	0.156528	9.7811
4.088	6.27536	39.09107	4.088	3.50312	28.97533	4.088	1.82556	20.62165	4.088	0.76721	13.89158	4.088	0.156672	9.7816
4.089	6.28339	39.11516	4.089	3.50936	29.01833	4.089	1.83016	20.66492	4.089	0.77043	13.93495	4.089	0.156816	9.7821
4.09	6.29142	39.13925	4.09	3.5156	29.06134	4.09	1.83477	20.70819	4.09	0.77365	13.97831	4.09	0.15696	9.7826
4.091	6.29945	39.16334	4.091	3.52185	29.10434	4.091	1.83939	20.75146	4.091	0.77688	14.02168	4.091	0.157104	9.7831
4.092	6.30748	39.18745	4.092	3.52811	29.14736	4.092	1.84402	20.79474	4.092	0.78013	14.06506	4.092	0.157248	9.7836
4.093	6.31552	39.21156	4.093	3.53437	29.19037	4.093	1.84866	20.83801	4.093	0.78338	14.10843	4.093	0.157392	9.7841
4.094	6.32356	39.23567	4.094	3.54064	29.23338	4.094	1.8533	20.88129	4.094	0.78664	14.1518	4.094	0.157536	9.7846
4.095	6.3316	39.25979	4.095	3.54692	29.2764	4.095	1.85795	20.92458	4.095	0.78991	14.19518	4.095	0.15768	9.7851
4.096	6.33964	39.28392	4.096	3.55321	29.31943	4.096	1.86261	20.96786	4.096	0.79318	14.23856	4.096	0.157824	9.7856
4.097	6.34768	39.30805	4.097	3.55951	29.36245	4.097	1.86728	21.01115	4.097	0.79647	14.28194	4.097	0.157968	9.7861
4.098	6.35573	39.33219	4.098	3.56581	29.40548	4.098	1.87196	21.05444	4.098	0.79977	14.32532	4.098	0.158112	9.7866
4.099	6.36378	39.35633	4.099	3.57212	29.44851	4.099	1.87664	21.09773	4.099	0.80307	14.36871	4.099	0.158256	9.7871
4.1	6.37183	39.38048	4.1	3.57843	29.49154	4.1	1.88134	21.14102	4.1	0.80639	14.41209	4.1	0.1584	9.7876
4.101	6.37988	39.40464	4.101	3.58476	29.53458	4.101	1.88604	21.18432	4.101	0.80971	14.45548	4.101	0.158544	9.7881
4.102	6.38793	39.4288	4.102	3.59109	29.57762	4.102	1.89075	21.22762	4.102	0.81304	14.49887	4.102	0.158688	9.7886
4.103	6.39599	39.45296	4.103	3.59743	29.62066	4.103	1.89547	21.27092	4.103	0.81638	14.54226	4.103	0.158832	9.7891
4.104	6.40405	39.47714	4.104	3.60377	29.66371	4.104	1.90019	21.31423	4.104	0.81973	14.58565	4.104	0.158976	9.7896
4.105	6.41211	39.50132	4.105	3.61013	29.70676	4.105	1.90493	21.35753	4.105	0.82309	14.62905	4.105	0.15912	9.7901
4.106	6.42017	39.5255	4.106	3.61649	29.74981	4.106	1.90967	21.40084	4.106	0.82646	14.67244	4.106	0.159264	9.7906
4.107	6.42823	39.54969	4.107	3.62286	29.79287	4.107	1.91442	21.44415	4.107	0.82983	14.71584	4.107	0.159408	9.7911
4.108	6.4363	39.57389	4.108	3.62923	29.83592	4.108	1.91918	21.48747	4.108	0.83322	14.75924	4.108	0.159552	9.7916
4.109	6.44437	39.59809	4.109	3.63561	29.87898	4.109	1.92395	21.53079	4.109	0.83661	14.80264	4.109	0.159696	9.7921
4.11	6.45244	39.6223	4.11	3.64201	29.92205	4.11	1.92872	21.57411	4.11	0.84002	14.84605	4.11	0.15984	9.7926
4.111	6.46051	39.64652	4.111	3.6484	29.96512	4.111	1.93351	21.61743	4.111	0.84343	14.88945	4.111	0.159984	9.7931
4.112	6.46858	39.67074	4.112	3.65481	30.00819	4.112	1.9383	21.66076	4.112	0.84685	14.93286	4.112	0.160128	9.7936
4.113	6.47666	39.69496	4.113	3.66122	30.05126	4.113	1.9431	21.70408	4.113	0.85028	14.97627	4.113	0.160272	9.7941
4.114	6.48473	39.71919	4.114	3.66764	30.09434	4.114	1.94791	21.74741	4.114	0.85372	15.01968	4.114	0.160416	9.7946
4.115	6.49281	39.74343	4.115	3.67407	30.13742	4.115	1.95272	21.79075	4.115	0.85717	15.0631	4.115	0.16056	9.7951
4.116	6.50089	39.76768	4.116	3.6805	30.1805	4.116	1.95755	21.83408	4.116	0.86062	15.10651	4.116	0.160704	9.7956
4.117	6.50898	39.79193	4.117	3.68694	30.22359	4.117	1.96238	21.87742	4.117	0.86409	15.14993	4.117	0.160848	9.7961

4.118	6.51706	39.81618	4.118	3.69339	30.26668	4.118	1.96722	21.92077	4.118	0.86756	15.19335	4.118	0.160992	9.7966
4.119	6.52515	39.84044	4.119	3.69985	30.30977	4.119	1.97207	21.96411	4.119	0.87105	15.23677	4.119	0.161136	9.7971
4.12	6.53324	39.86471	4.12	3.70631	30.35287	4.12	1.97693	22.00746	4.12	0.87454	15.2802	4.12	0.16128	9.7976
4.121	6.54133	39.88899	4.121	3.71279	30.39596	4.121	1.9818	22.05081	4.121	0.87804	15.32362	4.121	0.161424	9.7981
4.122	6.54942	39.91326	4.122	3.71926	30.43907	4.122	1.98667	22.09416	4.122	0.88155	15.36705	4.122	0.161568	9.7986
4.123	6.55752	39.93755	4.123	3.72575	30.48217	4.123	1.99155	22.13752	4.123	0.88507	15.41048	4.123	0.16204	9.83853
4.124	6.56562	39.96184	4.124	3.73224	30.52528	4.124	1.99644	22.18087	4.124	0.8886	15.45391	4.124	0.16439	9.88197
4.125	6.57371	39.98614	4.125	3.73874	30.56839	4.125	2.00134	22.22424	4.125	0.89213	15.49735	4.125	0.16675	9.92541
4.126	6.58182	40.01044	4.126	3.74525	30.61151	4.126	2.00625	22.2676	4.126	0.89568	15.54078	4.126	0.16912	9.96885
4.127	6.58992	40.03475	4.127	3.75177	30.65463	4.127	2.01116	22.31097	4.127	0.89923	15.58422	4.127	0.1715	10.01229
4.128	6.59802	40.05906	4.128	3.75829	30.69775	4.128	2.01609	22.35434	4.128	0.90279	15.62766	4.128	0.17389	10.05574
4.129	6.60613	40.08338	4.129	3.76482	30.74087	4.129	2.02102	22.39771	4.129	0.90637	15.6711	4.129	0.17629	10.09918
4.13	6.61424	40.10771	4.13	3.77136	30.784	4.13	2.02596	22.44108	4.13	0.90995	15.71455	4.13	0.1787	10.14263
4.131	6.62235	40.13204	4.131	3.7779	30.82713	4.131	2.0309	22.48446	4.131	0.91354	15.758	4.131	0.18112	10.18608
4.132	6.63046	40.15638	4.132	3.78445	30.87027	4.132	2.03586	22.52784	4.132	0.91713	15.80145	4.132	0.18354	10.22952
4.133	6.63858	40.16142	4.133	3.79101	30.9134	4.133	2.04082	22.57123	4.133	0.92074	15.8449	4.133	0.18598	10.27297
4.134	6.64668	40.15332	4.134	3.79758	30.95655	4.134	2.04579	22.61461	4.134	0.92436	15.88835	4.134	0.18842	10.31643
4.135	6.65479	40.14521	4.135	3.80415	30.99969	4.135	2.05077	22.658	4.135	0.92798	15.93181	4.135	0.19088	10.35988
4.136	6.66288	40.13712	4.136	3.81073	31.04284	4.136	2.05576	22.7014	4.136	0.93161	15.97527	4.136	0.19334	10.40333
4.137	6.67097	40.12903	4.137	3.81732	31.08599	4.137	2.06076	22.74479	4.137	0.93525	16.01873	4.137	0.19582	10.44679
4.138	6.67906	40.12094	4.138	3.82391	31.12914	4.138	2.06576	22.78819	4.138	0.9389	16.06219	4.138	0.1983	10.49025
4.139	6.68714	40.11286	4.139	3.83052	31.1723	4.139	2.07077	22.83159	4.139	0.94256	16.10566	4.139	0.20079	10.53371
4.14	6.69521	40.10479	4.14	3.83712	31.21546	4.14	2.07579	22.875	4.14	0.94623	16.14912	4.14	0.20329	10.57717
4.141	6.70328	40.09672	4.141	3.84374	31.25863	4.141	2.08082	22.91841	4.141	0.94991	16.19259	4.141	0.2058	10.62063
4.142	6.71134	40.08866	4.142	3.85037	31.3018	4.142	2.08586	22.96182	4.142	0.95359	16.23607	4.142	0.20832	10.66409
4.143	6.71939	40.08061	4.143	3.857	31.34497	4.143	2.0909	23.00523	4.143	0.95729	16.27954	4.143	0.21085	10.70756
4.144	6.72744	40.07256	4.144	3.86364	31.38814	4.144	2.09595	23.04865	4.144	0.96099	16.32302	4.144	0.21339	10.75102
4.145	6.73549	40.06451	4.145	3.87028	31.43132	4.145	2.10101	23.09207	4.145	0.9647	16.3665	4.145	0.21594	10.79449
4.146	6.74352	40.05648	4.146	3.87693	31.4745	4.146	2.10608	23.13549	4.146	0.96842	16.40998	4.146	0.21849	10.83796
4.147	6.75156	40.04844	4.147	3.88359	31.51769	4.147	2.11116	23.17892	4.147	0.97215	16.45346	4.147	0.22106	10.88143
4.148	6.75958	40.04042	4.148	3.89026	31.56088	4.148	2.11624	23.22235	4.148	0.97589	16.49695	4.148	0.22363	10.9249
4.149	6.7676	40.0324	4.149	3.89694	31.60407	4.149	2.12133	23.26578	4.149	0.97963	16.54044	4.149	0.22622	10.96838
4.15	6.77562	40.02438	4.15	3.90362	31.64726	4.15	2.12643	23.30921	4.15	0.98339	16.58393	4.15	0.22881	11.01185
4.151	6.78363	40.01637	4.151	3.91031	31.69046	4.151	2.13154	23.35265	4.151	0.98715	16.62743	4.151	0.23141	11.05533
4.152	6.79163	40.00837	4.152	3.917	31.73367	4.152	2.13666	23.39609	4.152	0.99092	16.67092	4.152	0.23403	11.09881
4.153	6.79963	40.00037	4.153	3.9237	31.77687	4.153	2.14178	23.43954	4.153	0.9947	16.71442	4.153	0.23665	11.14229
4.154	6.80762	39.99238	4.154	3.93042	31.82008	4.154	2.14692	23.48298	4.154	0.99849	16.75792	4.154	0.23928	11.18578
4.155	6.8156	39.9844	4.155	3.93713	31.86329	4.155	2.15206	23.52644	4.155	1.00229	16.80143	4.155	0.24192	11.22926
4.156	6.82358	39.97642	4.156	3.94386	31.90651	4.156	2.1572	23.56989	4.156	1.0061	16.84494	4.156	0.24457	11.27275
4.157	6.83156	39.96844	4.157	3.95059	31.94973	4.157	2.16236	23.61335	4.157	1.00991	16.88844	4.157	0.24723	11.31623
4.158	6.83953	39.96047	4.158	3.95733	31.99295	4.158	2.16752	23.65681	4.158	1.01374	16.93196	4.158	0.24989	11.35972
4.159	6.84749	39.95251	4.159	3.96407	32.03618	4.159	2.1727	23.70027	4.159	1.01757	16.97547	4.159	0.25257	11.40322
4.16	6.85545	39.94455	4.16	3.97083	32.07941	4.16	2.17788	23.74374	4.16	1.02141	17.01899	4.16	0.25525	11.44671
4.161	6.8634	39.9366	4.161	3.97759	32.12264	4.161	2.18306	23.78721	4.161	1.02526	17.06251	4.161	0.25795	11.4902
4.162	6.87134	39.92866	4.162	3.98436	32.16588	4.162	2.18826	23.83068	4.162	1.02912	17.10603	4.162	0.26065	11.5337
4.163	6.87928	39.92072	4.163	3.99113	32.20912	4.163	2.19346	23.87416	4.163	1.03299	17.14955	4.163	0.26337	11.5772
4.164	6.88722	39.91278	4.164	3.99791	32.25237	4.164	2.19868	23.91764	4.164	1.03686	17.19308	4.164	0.26609	11.6207
4.165	6.89514	39.90486	4.165	4.0047	32.29561	4.165	2.2039	23.96112	4.165	1.04075	17.23661	4.165	0.26882	11.6642
4.166	6.90307	39.89693	4.166	4.0115	32.33887	4.166	2.20912	24.0046	4.166	1.04464	17.28014	4.166	0.27156	11.70771
4.167	6.91098	39.88902	4.167	4.0183	32.38212	4.167	2.21436	24.04809	4.167	1.04854	17.32368	4.167	0.27431	11.75121
4.168	6.9189	39.8811	4.168	4.02511	32.42538	4.168	2.2196	24.09159	4.168	1.05245	17.36722	4.168	0.27707	11.79472
4.169	6.9268	39.8732	4.169	4.03193	32.46864	4.169	2.22486	24.13508	4.169	1.05637	17.41076	4.169	0.27984	11.83823
4.17	6.9347	39.8653	4.17	4.03875	32.51191	4.17	2.23011	24.17858	4.17	1.0603	17.4543	4.17	0.28261	11.88175
4.171	6.9426	39.8574	4.171	4.04558	32.55518	4.171	2.23538	24.22208	4.171	1.06423	17.49785	4.171	0.2854	11.92526
4.172	6.95048	39.84952	4.172	4.05242	32.59845	4.172	2.24066	24.26559	4.172	1.06818	17.5414	4.172	0.2882	11.96878
4.173	6.95837	39.84163	4.173	4.05927	32.64173	4.173	2.24594	24.3091	4.173	1.07213	17.58495	4.173	0.291	12.01229
4.174	6.96624	39.83376	4.174	4.06612	32.68501	4.174	2.25123	24.35261	4.174	1.07609	17.6285	4.174	0.29381	12.05581
4.175	6.97412	39.82588	4.175	4.07298	32.72829	4.175	2.25653	24.39613	4.175	1.08006	17.67206	4.175	0.29664	12.09934
4.176	6.98198	39.81802	4.176	4.07985	32.77158	4.176	2.26184	24.43965	4.176	1.08404	17.71562	4.176	0.29947	12.14286
4.177	6.98984	39.81016	4.177	4.08672	32.81487	4.177	2.26715	24.48317	4.177	1.08803	17.75918	4.177	0.30231	12.18639
4.178	6.9977	39.8023	4.178	4.0936	32.85817	4.178	2.27247	24.5267	4.178	1.09202	17.80275	4.178	0.30516	12.22992
4.179	7.00554	39.79446	4.179	4.10049	32.90147	4.179	2.2778	24.57022	4.179	1.09603	17.84632	4.179	0.30802	12.27345
4.18	7.01339	39.78661	4.18	4.10739	32.94477	4.18	2.28314	24.61376	4.18	1.10004	17.88989	4.18	0.31088	12.31698
4.181	7.02123	39.77877	4.181	4.11429	32.98808	4.181	2.28849	24.65729	4.181	1.10406	17.93346	4.181	0.31376	12.36051
4.182	7.02906	39.77094	4.182	4.1212	33.03139	4.182	2.29384	24.70083	4.182	1.10809	17.97704	4.182	0.31665	12.40405
4.183	7.03688	39.76312	4.183	4.12812	33.0747	4.183	2.29921	24.74438	4.183	1.11213	18.02062	4.183	0.31954	12.44759
4.184	7.04471	39.75529	4.184	4.13504	33.11802	4.184	2.30458	24.78792	4.184	1.11617	18.0642	4.184	0.32245	12.49113
4.185	7.05252	39.74748	4.185	4.14197	33.16134	4.185	2.30995	24.83147	4.185	1.12023	18.10779	4.185	0.32536	12.53467
4.186	7.06033	39.73967	4.186	4.14891	33.20467	4.186	2.31534	24.87503	4.186	1.12429	18.15138	4.186	0.32828	12.57822
4.187	7.06813	39.73187	4.187	4.15585	33.24799	4.187	2.32073	24.91858	4.187	1.12836	18.19497	4.187	0.33121	12.62177
4.188	7.07593	39.72407	4.188	4.1628	33.29133	4.188	2.32613	24.96215	4.188	1.13244	18.23856	4.188	0.33415	12.66532
4.189	7.08373	39.71627	4.189	4.16976	33.33466	4.189	2.33154	25.00571	4.189	1.13653	18.28216	4.189	0.3371	12.70887
4.19	7.09151	39.70849	4.19	4.17673	33.378	4.19	2.33696	25.04928	4.19	1.14063	18.32576	4.19	0.34006	12.75242
4.191	7.09929	39.70071	4.191	4.1837	33.42135	4.191	2.34238	25.09285	4.191	1.14474	18.36937	4.191	0.34302	12.79598
4.192	7.10707	39.69293	4.192	4.19068	33.4647	4.192	2.34781	25.13642	4.192	1.14885	18.41297	4.192	0.346	12.83954
4.193	7.11484	39.68516	4.193	4.19767	33.50805	4.193	2.35325	25.18	4.193	1.15297	18.45658	4.193	0.34899	12.8831
4.194	7.12261	39.67739	4.194	4.20466	33.5514	4.194	2.3587	25.22358	4.194	1.1571	18.50019	4.194	0.35198	12.92666
4.195	7.13037	39.66963	4.195	4.21166	33.59476	4.195	2.36416	25.26717	4.195	1.16124	18.54381	4.195	0.35498	12.97023
4.196	7.13812	39.66188	4.196	4.21867	33.63812	4.196	2.36962	25.31076	4.196	1.16539	18.58743	4.196	0.35799	13.01379
4.197	7.14587	39.65413	4.197	4.22569	33.68149	4.197	2.37509	25.35435	4.197	1.16955	18.63105	4.197	0.36101	13.05737
4.198	7.15361	39.64639	4.198	4.23271	33.72486	4.198	2.38057	25.39795	4.198	1.17371	18.67468	4.198	0.36404	13.10094
4.199	7.16135	39.63865	4.199	4.23974	33.76824	4.199	2.38606	25.44155	4.199	1.17789	18.7183	4.199	0.36708	13.14451
4.2	7.16908	39.63092	4.2	4.24677	33.81162	4.2	2.39155	25.48515	4.2	1.18207	18.76193	4.2	0.37013	13.18809
4.201	7.17681	39.62319	4.201	4.25382	33.855	4.201	2.39706	25.52876	4.201	1.18626	18.80557	4.201	0.37319	13.23167
4.202	7.18453	39.61547	4.202	4.26086	33.89838	4.202	2.40257	25.57237	4.202	1.19045	18.8492	4.202	0.37625	13.27525
4.203	7.19224	39.60776	4.203	4.26792	33.94177	4.203	2.40808	25.61598	4.203	1.19466	18.89285	4.203	0.37933	13.31884
4.204	7.19995	39.60005	4.204	4.27499	33.98517	4.204	2.41361	25.6596	4.204	1.19888	18.93649	4.204	0.38241	13.36242
4.205	7.20766	39.59234	4.205	4.28206	34.02857	4.205	2.41914	25.70322	4.205	1.2031	18.98014	4.205	0.3855	13.40601
4.206	7.21535	39.58465	4.206	4.28913	34.07197	4.206	2.42468	25.74685	4.206	1.20733	19.02378	4.206	0.3886	13.44961
4.207	7.22305	39.57695	4.207	4.29622	34.11537	4.207	2.43023	25.79048	4.207	1.21157	19.06744	4.207	0.39171	13.4932
4.208	7.23074	39.56926	4.208	4.30331	34.15878	4.208	2.43579	25.83411	4.208	1.21582	19.11109	4.208	0.39483	13.5368
4.209	7.23842	39.56158	4.209	4.31041	34.2022	4.209	2.44135	25.87774	4.209	1.22008	19.15475	4.209	0.39796	13.5804
4.21	7.24609	39.55391	4.21	4.31751	34.24561	4.21	2.44693	25.92138	4.21	1.22434	19.19842	4.21	0.40109	13.624
4.211	7.25377	39.54623	4.211	4.32463	34.28904	4.211	2.45251	25.96503	4.211	1.22862	19.24208	4.211	0.40424	13.6676
4.212	7.26143	39.53857	4.212	4.33174	34.33246	4.212	2.45809	26.00868	4.212	1.2329	19.28575	4.212	0.40739	13.71121
4.213	7.26909	39.53091	4.213	4.33887	34.37589	4.213	2.46369	26.05233	4.213	1.23719	19.32942	4.213	0.41056	13.75482
4.214	7.27675	39.52325	4.214	4.346	34.41932	4.214	2.46929	26.09598	4.214	1.24149	19.3731	4.214	0.41373	13.79843
4.215	7.2844	39.5156	4.215	4.35314	34.46276	4.215	2.4749	26.13964	4.215	1.24579	19.41678	4.215	0.41691	13.84205
4.216	7.29204	39.50796	4.216	4.36029	34.5062	4.216	2.48052	26.1833	4.216	1.25011	19.46046	4.216	0.4201	13.88567
4.217	7.29968	39.50032	4.217	4.36744	34.54965	4.217	2.48615	26.22697	4.217	1.25443	19.50414	4.217	0.4233	13.92929
4.218	7.30731	39.49269	4.218	4.37461	34.5931	4.218	2.49178	26.27064	4.218	1.25876	19.54783	4.218	0.4265	13.97291
4.219	7.31494	39.48506	4.219	4.38177	34.63655	4.219	2.49742	26.31432	4.219	1.2631	19.59152	4.219	0.42972	14.01654
4.22	7.32256	39.47744	4.22	4.38895	34.68001	4.22	2.50307	26.35799	4.22	1.26745	19.63522	4.22	0.43294	14.06016
4.221	7.33018	39.46982	4.221	4.39613	34.72347	4.221	2.50873	26.40168	4.221	1.27181	19.67892	4.221	0.43618	14.10379
4.222	7.33779	39.46221	4.222	4.40332	34.76694	4.222	2.51439	26.44536	4.222	1.27617	19.72262	4.222	0.43942	14.14743
4.223	7.3454	39.4546	4.223	4.41051	34.8104	4.223	2.52006	26.48905	4.223	1.28055	19.76633	4.223	0.44267	14.19106
4.224	7.353	39.447	4.224	4.41771	34.85388	4.224	2.52574	26.53274	4.224	1.28493	19.81004	4.224	0.44593	14.2347
4.225	7.36059	39.43941	4.225	4.42492	34.89736	4.225	2.53143	26.57644	4.225	1.28932	19.85375	4.225	0.4492	14.27834
4.226	7.36818	39.43182	4.226	4.43214	34.94084	4.226	2.53712	26.62014	4.226	1.29372	19.89746	4.226	0.45248	14.32199
4.227	7.37577	39.42423	4.227	4.43936	34.98432	4.227	2.54283	26.66385	4.227	1.29812	19.94118	4.227	0.45576	14.36564
4.228	7.38335	39.41665	4.228	4.44659	35.02781	4.228	2.54854	26.70756	4.228	1.30254	19.9849	4.228	0.45906	14.40929
4.229	7.39092	39.40908	4.229	4.45383	35.07131	4.229	2.55426	26.75127	4.229	1.30696	20.02863	4.229	0.46236	14.45294
4.23	7.39849	39.40151	4.23	4.46107	35.11481	4.23	2.55998	26.79499	4.23	1.31139	20.07236	4.23	0.46568	14.4966
4.231	7.40605	39.39395	4.231	4.46832	35.15831	4.231	2.56571	26.83871	4.231	1.31583	20.11609	4.231	0.469	14.54025
4.232	7.41361	39.38639	4.232	4.47558	35.20181	4.232	2.57145	26.88243	4.232	1.32028	20.15983	4.232	0.47233	14.58391
4.233	7.42116	39.37884	4.233	4.48284	35.24532	4.233	2.5772	26.92616	4.233	1.32473	20.20357	4.233	0.47567	14.62758
4.234	7.42871	39.37129	4.234	4.49011	35.28884	4.234	2.58296	26.96989	4.234	1.3292	20.24731	4.234	0.47901	14.67125
4.235	7.43625	39.36375	4.235	4.49739	35.33236	4.235	2.58872	27.01363	4.235	1.33367	20.29106	4.235	0.48237	14.71492
4.236	7.44379	39.35621	4.236	4.50467	35.37588	4.236	2.59449	27.05737	4.236	1.33815	20.33481	4.236	0.48574	14.75859
4.237	7.45132	39.34868	4.237	4.51197	35.41941	4.237	2.60027	27.10111	4.237	1.34264	20.37856	4.237	0.48911	14.80226
4.238	7.45884	39.34116	4.238	4.51926	35.46294	4.238	2.60606	27.14486	4.238	1.34714	20.42232	4.238	0.49249	14.84594
4.239	7.46636	39.33364	4.239	4.52657	35.50647	4.239	2.61185	27.18862	4.239	1.35164	20.46608	4.239	0.49588	14.88962
4.24	7.47388	39.32612	4.24	4.53388	35.55001	4.24	2.61765	27.23237	4.24	1.35616	20.50985	4.24	0.49928	14.93331
4.241	7.48139	39.31861	4.241	4.5412	35.59356	4.241	2.62346	27.27613	4.241	1.36068	20.55362	4.241	0.50269	14.977
4.242	7.48889	39.31111	4.242	4.54852	35.6371	4.242	2.62928	27.3199	4.242	1.36521	20.59739	4.242	0.50611	15.02069
4.243	7.49639	39.30361	4.243	4.55586	35.68066	4.243	2.6351	27.36367	4.243	1.36974	20.64116	4.243	0.50953	15.06438
4.244	7.50389	39.29611	4.244	4.5632	35.72421	4.244	2.64094	27.40744	4.244	1.37429	20.68494	4.244	0.51297	15.10808
4.245	7.51137	39.28863	4.245	4.57054	35.76777	4.245	2.64678	27.45122	4.245	1.37884	20.72872	4.245	0.51641	15.15178
4.246	7.51886	39.28114	4.246	4.57789	35.81134	4.246	2.65262	27.495	4.246	1.38341	20.77251	4.246	0.51986	15.19548
4.247	7.52634	39.27366	4.247	4.58525	35.85491	4.247	2.65848	27.53878	4.247	1.38798	20.8163	4.247	0.52333	15.23918
4.248	7.53381	39.26619	4.248	4.59262	35.89848	4.248	2.66434	27.58257	4.248	1.39256	20.86009	4.248	0.52679	15.28289
4.249	7.54128	39.25872	4.249	4.59999	35.94206	4.249	2.67021	27.62636	4.249	1.39714	20.90389	4.249	0.53027	15.3266
4.25	7.54874	39.25126	4.25	4.60737	35.98564	4.25	2.67609	27.67016	4.25	1.40174	20.94769	4.25	0.53376	15.37032
4.251	7.55619	39.24381	4.251	4.61476	36.02923	4.251	2.68197	27.71396	4.251	1.40634	20.9915	4.251	0.53725	15.41404
4.252	7.56365	39.23635	4.252	4.62215	36.07282	4.252	2.68786	27.75777	4.252	1.41095	21.03531	4.252	0.54076	15.45776
4.253	7.57109	39.22891	4.253	4.62955	36.11641	4.253	2.69376	27.80158	4.253	1.41557	21.07912	4.253	0.54427	15.50148
4.254	7.57853	39.22147	4.254	4.63696	36.16001	4.254	2.69967	27.84539	4.254	1.4202	21.12293	4.254	0.54779	15.54521
4.255	7.58597	39.21403	4.255	4.64437	36.20361	4.255	2.70559	27.88921	4.255	1.42483	21.16675	4.255	0.55132	15.58894
4.256	7.5934	39.2066	4.256	4.65179	36.24722	4.256	2.71151	27.93303	4.256	1.42948	21.21058	4.256	0.55486	15.63267
4.257	7.60082	39.19918	4.257	4.65922	36.29083	4.257	2.71744	27.97686	4.257	1.43413	21.25441	4.257	0.55841	15.67641
4.258	7.60824	39.19176	4.258	4.66665	36.33445	4.258	2.72338	28.02069	4.258	1.43879	21.29824	4.258	0.56196	15.72015
4.259	7.61566	39.18434	4.259	4.67409	36.37807	4.259	2.72932	28.06453	4.259	1.44346	21.34207	4.259	0.56553	15.76389
4.26	7.62307	39.17693	4.26	4.68154	36.42169	4.26	2.73527	28.10837	4.26	1.44813	21.38591	4.26	0.5691	15.80764
4.261	7.63047	39.16953	4.261	4.689	36.46532	4.261	2.74123	28.15221	4.261	1.45282	21.42975	4.261	0.57268	15.85139
4.262	7.63787	39.16213	4.262	4.69646	36.50896	4.262	2.7472	28.19606	4.262	1.45751	21.4736	4.262	0.57627	15.89514
4.263	7.64527	39.15473	4.263	4.70392	36.55259	4.263	2.75317	28.23991	4.263	1.46221	21.51745	4.263	0.57987	15.9389
4.264	7.65266	39.14734	4.264	4.7114	36.59623	4.264	2.75916	28.28376	4.264	1.46692	21.5613	4.264	0.58348	15.98266
4.265	7.66004	39.13996	4.265	4.71888	36.63988	4.265	2.76515	28.32762	4.265	1.47164	21.60516	4.265	0.58709	16.02642
4.266	7.66742	39.13258	4.266	4.72637	36.68353	4.266	2.77114	28.37149	4.266	1.47636	21.64902	4.266	0.59072	16.07018
4.267	7.67479	39.12521	4.267	4.73386	36.72719	4.267	2.77715	28.41536	4.267	1.48109	21.69289	4.267	0.59435	16.11395
4.268	7.68216	39.11784	4.268	4.74136	36.77085	4.268	2.78316	28.45923	4.268	1.48583	21.73676	4.268	0.59799	16.15773
4.269	7.68952	39.11048	4.269	4.74887	36.81451	4.269	2.78918	28.50311	4.269	1.49058	21.78063	4.269	0.60164	16.2015
4.27	7.69688	39.10312	4.27	4.75639	36.85818	4.27	2.79521	28.54699	4.27	1.49534	21.82451	4.27	0.6053	16.24528
4.271	7.70423	39.09577	4.271	4.76391	36.90185	4.271	2.80124	28.59088	4.271	1.50011	21.86839	4.271	0.60897	16.28906
4.272	7.71158	39.08842	4.272	4.77144	36.94553	4.272	2.80728	28.63477	4.272	1.50488	21.91228	4.272	0.61264	16.33285
4.273	7.71892	39.08108	4.273	4.77897	36.98921	4.273	2.81333	28.67866	4.273	1.50966	21.95616	4.273	0.61633	16.37664
4.274	7.72626	39.07374	4.274	4.78651	37.0329	4.274	2.81939	28.72256	4.274	1.51445	22.00006	4.274	0.62002	16.42043
4.275	7.73359	39.06641	4.275	4.79406	37.07659	4.275	2.82546	28.76647	4.275	1.51925	22.04396	4.275	0.62372	16.46423
4.276	7.74092	39.05908	4.276	4.80161	37.12028	4.276	2.83153	28.81037	4.276	1.52405	22.08786	4.276	0.62743	16.50803
4.277	7.74824	39.05176	4.277	4.80918	37.16398	4.277	2.83761	28.85429	4.277	1.52886	22.13176	4.277	0.63115	16.55183

4.278	7.75555	39.04445	4.278	4.81674	37.20769	4.278	2.84369	28.8982	4.278	1.53369	22.17567	4.278	0.63487	16.59564
4.279	7.76287	39.03713	4.279	4.82432	37.25139	4.279	2.84979	28.94212	4.279	1.53851	22.21958	4.279	0.63861	16.63945
4.28	7.77017	39.02983	4.28	4.8319	37.29511	4.28	2.85589	28.98605	4.28	1.54335	22.2635	4.28	0.64235	16.68326
4.281	7.77747	39.02253	4.281	4.83949	37.33882	4.281	2.862	29.02998	4.281	1.5482	22.30742	4.281	0.64611	16.72708
4.282	7.78477	39.01523	4.282	4.84708	37.38255	4.282	2.86811	29.07391	4.282	1.55305	22.35135	4.282	0.64987	16.7709
4.283	7.79206	39.00794	4.283	4.85469	37.42627	4.283	2.87424	29.11785	4.283	1.55791	22.39528	4.283	0.65364	16.81472
4.284	7.79935	39.00065	4.284	4.86229	37.47	4.284	2.88037	29.1618	4.284	1.56278	22.43921	4.284	0.65741	16.85855
4.285	7.80663	38.99337	4.285	4.86991	37.51374	4.285	2.88651	29.20574	4.285	1.56766	22.48315	4.285	0.6612	16.90238
4.286	7.8139	38.9861	4.286	4.87753	37.55748	4.286	2.89265	29.2497	4.286	1.57254	22.52709	4.286	0.66499	16.94621
4.287	7.82117	38.97883	4.287	4.88516	37.60122	4.287	2.89881	29.29365	4.287	1.57744	22.57104	4.287	0.6688	16.99005
4.288	7.82844	38.97156	4.288	4.89279	37.64497	4.288	2.90497	29.33761	4.288	1.58234	22.61499	4.288	0.67261	17.03389
4.289	7.8357	38.9643	4.289	4.90043	37.68873	4.289	2.91113	29.38158	4.289	1.58725	22.65894	4.289	0.67643	17.07774
4.29	7.84295	38.95705	4.29	4.90808	37.73248	4.29	2.91731	29.42555	4.29	1.59216	22.7029	4.29	0.68026	17.12159
4.291	7.8502	38.9498	4.291	4.91574	37.77625	4.291	2.92349	29.46952	4.291	1.59709	22.74686	4.291	0.68409	17.16544
4.292	7.85745	38.94255	4.292	4.9234	37.82001	4.292	2.92968	29.5135	4.292	1.60202	22.79083	4.292	0.68794	17.2093
4.293	7.86469	38.93531	4.293	4.93107	37.86378	4.293	2.93588	29.55749	4.293	1.60696	22.8348	4.293	0.69179	17.25316
4.294	7.87192	38.92808	4.294	4.93874	37.90756	4.294	2.94209	29.60148	4.294	1.61191	22.87877	4.294	0.69565	17.29702
4.295	7.87915	38.92085	4.295	4.94642	37.95134	4.295	2.9483	29.64547	4.295	1.61687	22.92275	4.295	0.69952	17.34089
4.296	7.88638	38.91362	4.296	4.95411	37.99513	4.296	2.95452	29.68947	4.296	1.62183	22.96674	4.296	0.7034	17.38476
4.297	7.8936	38.9064	4.297	4.9618	38.03892	4.297	2.96074	29.73347	4.297	1.6268	23.01072	4.297	0.70729	17.42863
4.298	7.90081	38.89919	4.298	4.9695	38.08271	4.298	2.96698	29.77747	4.298	1.63178	23.05472	4.298	0.71119	17.47251
4.299	7.90802	38.89198	4.299	4.97721	38.12651	4.299	2.97322	29.82149	4.299	1.63677	23.09871	4.299	0.71509	17.51639
4.3	7.91523	38.88477	4.3	4.98493	38.17032	4.3	2.97947	29.8655	4.3	1.64177	23.14271	4.3	0.719	17.56028
4.301	7.92243	38.87757	4.301	4.99265	38.21412	4.301	2.98572	29.90952	4.301	1.64677	23.18672	4.301	0.72292	17.60417
4.302	7.92962	38.87038	4.302	5.00038	38.25794	4.302	2.99199	29.95355	4.302	1.65178	23.23073	4.302	0.72685	17.64806
4.303	7.93681	38.86319	4.303	5.00811	38.30176	4.303	2.99826	29.99758	4.303	1.6568	23.27474	4.303	0.73079	17.69196
4.304	7.944	38.856	4.304	5.01585	38.34558	4.304	3.00454	30.04161	4.304	1.66183	23.31876	4.304	0.73473	17.73586
4.305	7.95118	38.84882	4.305	5.0236	38.3894	4.305	3.01082	30.08565	4.305	1.66687	23.36278	4.305	0.73869	17.77976
4.306	7.95835	38.84165	4.306	5.03135	38.43324	4.306	3.01712	30.12969	4.306	1.67191	23.40681	4.306	0.74265	17.82367
4.307	7.96552	38.83448	4.307	5.03911	38.47707	4.307	3.02342	30.17374	4.307	1.67696	23.45084	4.307	0.74662	17.86758
4.308	7.97269	38.82731	4.308	5.04688	38.52091	4.308	3.02972	30.21779	4.308	1.68202	23.49487	4.308	0.7506	17.9115
4.309	7.97984	38.82016	4.309	5.05465	38.56476	4.309	3.03604	30.26185	4.309	1.68709	23.53891	4.309	0.75459	17.95542
4.31	7.987	38.813	4.31	5.06243	38.60861	4.31	3.04236	30.30591	4.31	1.69217	23.58296	4.31	0.75859	17.99934
4.311	7.99415	38.80585	4.311	5.07022	38.65247	4.311	3.04869	30.34998	4.311	1.69725	23.627	4.311	0.76259	18.04327
4.312	8.00129	38.79871	4.312	5.07801	38.69633	4.312	3.05503	30.39405	4.312	1.70234	23.67106	4.312	0.7666	18.0872
4.313	8.00843	38.79157	4.313	5.08581	38.74019	4.313	3.06137	30.43813	4.313	1.70744	23.71511	4.313	0.77062	18.13114
4.314	8.01557	38.78443	4.314	5.09362	38.78406	4.314	3.06772	30.48221	4.314	1.71254	23.75917	4.314	0.77465	18.17508
4.315	8.0227	38.7773	4.315	5.10143	38.82793	4.315	3.07408	30.52629	4.315	1.71766	23.80324	4.315	0.77869	18.21902
4.316	8.02982	38.77018	4.316	5.10925	38.87181	4.316	3.08044	30.57038	4.316	1.72278	23.84731	4.316	0.78274	18.26297
4.317	8.03694	38.76306	4.317	5.11708	38.9157	4.317	3.08682	30.61448	4.317	1.72791	23.89138	4.317	0.78679	18.30692
4.318	8.04406	38.75594	4.318	5.12491	38.95958	4.318	3.0932	30.65858	4.318	1.73305	23.93546	4.318	0.79085	18.35087
4.319	8.05117	38.74883	4.319	5.13275	39.00348	4.319	3.09959	30.70268	4.319	1.73819	23.97955	4.319	0.79492	18.39483
4.32	8.05827	38.74173	4.32	5.14059	39.04738	4.32	3.10598	30.74679	4.32	1.74335	24.02363	4.32	0.799	18.4388
4.321	8.06537	38.73463	4.321	5.14844	39.09128	4.321	3.11238	30.79091	4.321	1.74851	24.06773	4.321	0.80309	18.48276
4.322	8.07247	38.72753	4.322	5.1563	39.13141	4.322	3.11879	30.83503	4.322	1.75368	24.11182	4.322	0.80719	18.52673
4.323	8.07956	38.72044	4.323	5.16417	39.15675	4.323	3.12521	30.87915	4.323	1.75886	24.15593	4.323	0.81129	18.57071
4.324	8.08664	38.71336	4.324	5.17203	39.18209	4.324	3.13163	30.92328	4.324	1.76404	24.20003	4.324	0.8154	18.61469
4.325	8.09373	38.70627	4.325	5.1799	39.20744	4.325	3.13806	30.96741	4.325	1.76923	24.24414	4.325	0.81952	18.65867
4.326	8.1008	38.6992	4.326	5.18776	39.23279	4.326	3.1445	31.01155	4.326	1.77443	24.28826	4.326	0.82365	18.70266
4.327	8.10787	38.69213	4.327	5.19564	39.25815	4.327	3.15095	31.05569	4.327	1.77964	24.33238	4.327	0.82779	18.74665
4.328	8.11494	38.68506	4.328	5.20351	39.28352	4.328	3.1574	31.09984	4.328	1.78486	24.3765	4.328	0.83193	18.79064
4.329	8.122	38.678	4.329	5.21138	39.3089	4.329	3.16386	31.144	4.329	1.79008	24.42063	4.329	0.83609	18.83464
4.33	8.12905	38.67095	4.33	5.21926	39.33428	4.33	3.17033	31.18815	4.33	1.79531	24.46476	4.33	0.84025	18.87865
4.331	8.13611	38.66389	4.331	5.22714	39.35967	4.331	3.1768	31.23232	4.331	1.80055	24.5089	4.331	0.84442	18.92266
4.332	8.14315	38.65685	4.332	5.23502	39.38507	4.332	3.18328	31.27648	4.332	1.8058	24.55304	4.332	0.8486	18.96667
4.333	8.15019	38.64981	4.333	5.24291	39.41048	4.333	3.18977	31.32066	4.333	1.81105	24.59719	4.333	0.85278	19.01068
4.334	8.15723	38.64277	4.334	5.25079	39.43589	4.334	3.19627	31.36483	4.334	1.81632	24.64134	4.334	0.85698	19.0547
4.335	8.16426	38.63574	4.335	5.25868	39.46131	4.335	3.20277	31.40902	4.335	1.82159	24.6855	4.335	0.86118	19.09873
4.336	8.17129	38.62871	4.336	5.26657	39.48673	4.336	3.20928	31.4532	4.336	1.82687	24.72966	4.336	0.86539	19.14276
4.337	8.17831	38.62169	4.337	5.27447	39.51217	4.337	3.2158	31.49739	4.337	1.83215	24.77383	4.337	0.86961	19.18679
4.338	8.18533	38.61467	4.338	5.28236	39.53761	4.338	3.22232	31.54159	4.338	1.83744	24.818	4.338	0.87384	19.23083
4.339	8.19234	38.60766	4.339	5.29026	39.56306	4.339	3.22885	31.58579	4.339	1.84275	24.86217	4.339	0.87808	19.27487
4.34	8.19935	38.60065	4.34	5.29816	39.58851	4.34	3.23539	31.63	4.34	1.84806	24.90635	4.34	0.88232	19.31891
4.341	8.20635	38.59365	4.341	5.30606	39.61397	4.341	3.24194	31.67421	4.341	1.85337	24.95054	4.341	0.88657	19.36296
4.342	8.21335	38.58665	4.342	5.31397	39.63944	4.342	3.24849	31.71843	4.342	1.8587	24.99473	4.342	0.89083	19.40702
4.343	8.22034	38.57966	4.343	5.32187	39.66492	4.343	3.25505	31.76265	4.343	1.86403	25.03892	4.343	0.8951	19.45107
4.344	8.22733	38.57267	4.344	5.32978	39.6904	4.344	3.26162	31.80688	4.344	1.86937	25.08312	4.344	0.89938	19.49514
4.345	8.23431	38.56569	4.345	5.33769	39.7159	4.345	3.26819	31.85111	4.345	1.87472	25.12732	4.345	0.90366	19.5392
4.346	8.24129	38.55871	4.346	5.34561	39.74139	4.346	3.27478	31.89535	4.346	1.88007	25.17153	4.346	0.90796	19.58328
4.347	8.24826	38.55174	4.347	5.35352	39.7669	4.347	3.28136	31.93959	4.347	1.88544	25.21574	4.347	0.91226	19.62735
4.348	8.25523	38.54477	4.348	5.36144	39.79241	4.348	3.28796	31.98383	4.348	1.89081	25.25996	4.348	0.91657	19.67143
4.349	8.26219	38.53781	4.349	5.36936	39.81793	4.349	3.29456	32.02809	4.349	1.89618	25.30418	4.349	0.92088	19.71551
4.35	8.26915	38.53085	4.35	5.37728	39.84346	4.35	3.30117	32.07234	4.35	1.90157	25.34841	4.35	0.92521	19.7596
4.351	8.27611	38.52389	4.351	5.38521	39.86899	4.351	3.30779	32.1166	4.351	1.90696	25.39264	4.351	0.92954	19.8037
4.352	8.28306	38.51694	4.352	5.39313	39.89453	4.352	3.31442	32.16087	4.352	1.91237	25.43688	4.352	0.93389	19.84779
4.353	8.29	38.51	4.353	5.40106	39.92008	4.353	3.32105	32.20514	4.353	1.91778	25.48112	4.353	0.93824	19.89189
4.354	8.29694	38.50306	4.354	5.40899	39.94564	4.354	3.32769	32.24942	4.354	1.92319	25.52537	4.354	0.94259	19.936
4.355	8.30388	38.49612	4.355	5.41693	39.9712	4.355	3.33433	32.2937	4.355	1.92862	25.56962	4.355	0.94696	19.98011
4.356	8.31081	38.48919	4.356	5.42486	39.99677	4.356	3.34099	32.33799	4.356	1.93405	25.61388	4.356	0.95133	20.02423
4.357	8.31773	38.48227	4.357	5.4328	40.02235	4.357	3.34765	32.38228	4.357	1.93949	25.65814	4.357	0.95572	20.06834
4.358	8.32465	38.47535	4.358	5.44074	40.04793	4.358	3.35432	32.42658	4.358	1.94494	25.7024	4.358	0.96011	20.11247
4.359	8.33157	38.46843	4.359	5.44868	40.07352	4.359	3.36099	32.47088	4.359	1.95039	25.74667	4.359	0.96451	20.1566
4.36	8.33848	38.46152	4.36	5.45663	40.09912	4.36	3.36767	32.51519	4.36	1.95586	25.79095	4.36	0.96891	20.20073
4.361	8.34539	38.45461	4.361	5.46457	40.12473	4.361	3.37436	32.5595	4.361	1.96133	25.83523	4.361	0.97333	20.24487
4.362	8.35229	38.44771	4.362	5.47252	40.15034	4.362	3.38106	32.60382	4.362	1.9668	25.87952	4.362	0.97775	20.28901
4.363	8.35919	38.44081	4.363	5.48047	40.17596	4.363	3.38776	32.64814	4.363	1.97229	25.92381	4.363	0.98218	20.33315
4.364	8.36608	38.43392	4.364	5.48843	40.20159	4.364	3.39447	32.69247	4.364	1.97778	25.9681	4.364	0.98662	20.3773
4.365	8.37297	38.42703	4.365	5.49638	40.22722	4.365	3.40119	32.7368	4.365	1.98328	26.0124	4.365	0.99107	20.42146
4.366	8.37985	38.42015	4.366	5.50434	40.25286	4.366	3.40791	32.78114	4.366	1.98879	26.05671	4.366	0.99552	20.46562
4.367	8.38673	38.41327	4.367	5.5123	40.27851	4.367	3.41464	32.82549	4.367	1.99431	26.10102	4.367	0.99999	20.50978
4.368	8.3936	38.4064	4.368	5.52026	40.30417	4.368	3.42138	32.86984	4.368	1.99983	26.14533	4.368	1.00446	20.55395
4.369	8.40047	38.39953	4.369	5.52823	40.32983	4.369	3.42813	32.91419	4.369	2.00537	26.18965	4.369	1.00894	20.59813
4.37	8.40733	38.39267	4.37	5.53619	40.3555	4.37	3.43488	32.95855	4.37	2.0109	26.23398	4.37	1.01343	20.6423
4.371	8.41419	38.38581	4.371	5.54416	40.38118	4.371	3.44164	33.00291	4.371	2.01645	26.27831	4.371	1.01792	20.68649
4.372	8.42105	38.37895	4.372	5.55213	40.40687	4.372	3.4484	33.04728	4.372	2.02201	26.32265	4.372	1.02243	20.73067
4.373	8.4279	38.3721	4.373	5.56011	40.43256	4.373	3.45518	33.09166	4.373	2.02757	26.36699	4.373	1.02694	20.77487
4.374	8.43474	38.36526	4.374	5.56808	40.45826	4.374	3.46196	33.13604	4.374	2.03314	26.41133	4.374	1.03146	20.81906
4.375	8.44158	38.35842	4.375	5.57606	40.48396	4.375	3.46875	33.18042	4.375	2.03872	26.45568	4.375	1.03599	20.86326
4.376	8.44842	38.35158	4.376	5.58404	40.50968	4.376	3.47554	33.22481	4.376	2.0443	26.50004	4.376	1.04052	20.90747
4.377	8.45525	38.34475	4.377	5.59202	40.5354	4.377	3.48234	33.26921	4.377	2.04989	26.5444	4.377	1.04507	20.95168
4.378	8.46208	38.33792	4.378	5.60001	40.56113	4.378	3.48915	33.31361	4.378	2.05549	26.58876	4.378	1.04962	20.9959
4.379	8.4689	38.3311	4.379	5.60799	40.58686	4.379	3.49597	33.35802	4.379	2.0611	26.63314	4.379	1.05418	21.04011
4.38	8.47572	38.32428	4.38	5.61598	40.6126	4.38	3.50279	33.40243	4.38	2.06672	26.67751	4.38	1.05875	21.08434
4.381	8.48253	38.31747	4.381	5.62397	40.63835	4.381	3.50962	33.44685	4.381	2.07234	26.72189	4.381	1.06332	21.12857
4.382	8.48934	38.31066	4.382	5.63197	40.66411	4.382	3.51646	33.49127	4.382	2.07797	26.76628	4.382	1.06791	21.1728
4.383	8.49614	38.30386	4.383	5.63996	40.68987	4.383	3.5233	33.5357	4.383	2.08361	26.81067	4.383	1.0725	21.21704
4.384	8.50294	38.29706	4.384	5.64796	40.71564	4.384	3.53015	33.58013	4.384	2.08925	26.85507	4.384	1.0771	21.26129
4.385	8.50973	38.29027	4.385	5.65596	40.74142	4.385	3.53701	33.62457	4.385	2.0949	26.89947	4.385	1.08171	21.30553
4.386	8.51652	38.28348	4.386	5.66396	40.76721	4.386	3.54387	33.66901	4.386	2.10056	26.94387	4.386	1.08632	21.34979
4.387	8.5233	38.2767	4.387	5.67197	40.793	4.387	3.55075	33.71346	4.387	2.10623	26.98829	4.387	1.09095	21.39404
4.388	8.53008	38.26992	4.388	5.67997	40.8188	4.388	3.55763	33.75792	4.388	2.11191	27.0327	4.388	1.09558	21.43831
4.389	8.53686	38.26314	4.389	5.68798	40.84461	4.389	3.56451	33.80238	4.389	2.11759	27.07713	4.389	1.10022	21.48257
4.39	8.54363	38.25637	4.39	5.69599	40.87042	4.39	3.5714	33.84684	4.39	2.12328	27.12155	4.39	1.10487	21.52685
4.391	8.5504	38.2496	4.391	5.70401	40.89624	4.391	3.5783	33.89131	4.391	2.12898	27.16599	4.391	1.10952	21.57112
4.392	8.55716	38.24284	4.392	5.71202	40.92207	4.392	3.58521	33.93579	4.392	2.13469	27.21042	4.392	1.11419	21.61541
4.393	8.56391	38.23609	4.393	5.72004	40.94791	4.393	3.59213	33.98027	4.393	2.1404	27.25487	4.393	1.11886	21.65969
4.394	8.57067	38.22933	4.394	5.72806	40.97375	4.394	3.59905	34.02476	4.394	2.14612	27.29932	4.394	1.12354	21.70398
4.395	8.57741	38.22259	4.395	5.73608	40.9996	4.395	3.60597	34.06925	4.395	2.15185	27.34377	4.395	1.12823	21.74828
4.396	8.58416	38.21584	4.396	5.74411	41.02546	4.396	3.61291	34.11374	4.396	2.15758	27.38823	4.396	1.13292	21.79258
4.397	8.5909	38.2091	4.397	5.75214	41.04786	4.397	3.61985	34.15825	4.397	2.16333	27.43269	4.397	1.13763	21.83689
4.398	8.59763	38.20237	4.398	5.76016	41.03984	4.398	3.6268	34.20276	4.398	2.16908	27.47716	4.398	1.14234	21.8812
4.399	8.60436	38.19564	4.399	5.76818	41.03182	4.399	3.63376	34.24727	4.399	2.17484	27.52164	4.399	1.14706	21.92552
4.4	8.61108	38.18892	4.4	5.77619	41.02381	4.4	3.64072	34.29179	4.4	2.1806	27.56612	4.4	1.15178	21.96984
4.401	8.6178	38.1822	4.401	5.7842	41.0158	4.401	3.64769	34.33631	4.401	2.18637	27.6106	4.401	1.15652	22.01416
4.402	8.62452	38.17548	4.402	5.7922	41.0078	4.402	3.65466	34.38084	4.402	2.19215	27.65509	4.402	1.16126	22.0585
4.403	8.63123	38.16877	4.403	5.8002	40.9998	4.403	3.66165	34.42538	4.403	2.19794	27.69959	4.403	1.16601	22.10283
4.404	8.63794	38.16206	4.404	5.80819	40.99181	4.404	3.66864	34.46992	4.404	2.20374	27.74409	4.404	1.17077	22.14717
4.405	8.64464	38.15536	4.405	5.81618	40.98382	4.405	3.67564	34.51447	4.405	2.20954	27.7886	4.405	1.17554	22.19152
4.406	8.65133	38.14867	4.406	5.82416	40.97584	4.406	3.68264	34.55902	4.406	2.21535	27.83311	4.406	1.18031	22.23587
4.407	8.65803	38.14197	4.407	5.83214	40.96786	4.407	3.68965	34.60358	4.407	2.22117	27.87763	4.407	1.1851	22.28023
4.408	8.66471	38.13529	4.408	5.84011	40.95989	4.408	3.69667	34.64814	4.408	2.227	27.92215	4.408	1.18989	22.32459
4.409	8.6714	38.1286	4.409	5.84807	40.95193	4.409	3.70369	34.69271	4.409	2.23283	27.96668	4.409	1.19469	22.36895
4.41	8.67808	38.12192	4.41	5.85603	40.94397	4.41	3.71073	34.73728	4.41	2.23867	28.01121	4.41	1.19949	22.41332
4.411	8.68475	38.11525	4.411	5.86398	40.93602	4.411	3.71777	34.78186	4.411	2.24452	28.05575	4.411	1.20431	22.4577
4.412	8.69142	38.10858	4.412	5.87193	40.92807	4.412	3.72481	34.82645	4.412	2.25037	28.10029	4.412	1.20913	22.50208
4.413	8.69809	38.10191	4.413	5.87987	40.92013	4.413	3.73186	34.87104	4.413	2.25623	28.14484	4.413	1.21396	22.54647
4.414	8.70475	38.09525	4.414	5.88781	40.91219	4.414	3.73892	34.91564	4.414	2.26211	28.1894	4.414	1.2188	22.59086
4.415	8.7114	38.0886	4.415	5.89574	40.90426	4.415	3.74599	34.96024	4.415	2.26798	28.23396	4.415	1.22364	22.63526
4.416	8.71805	38.08195	4.416	5.90367	40.89633	4.416	3.75306	35.00485	4.416	2.27387	28.27853	4.416	1.2285	22.67966
4.417	8.7247	38.0753	4.417	5.91159	40.88841	4.417	3.76014	35.04946	4.417	2.27976	28.3231	4.417	1.23336	22.72406
4.418	8.73134	38.06866	4.418	5.91951	40.88049	4.418	3.76723	35.09408	4.418	2.28566	28.36768	4.418	1.23823	22.76848
4.419	8.73798	38.06202	4.419	5.92742	40.87258	4.419	3.77433	35.1387	4.419	2.29157	28.41226	4.419	1.24311	22.81289
4.42	8.74462	38.05538	4.42	5.93532	40.86468	4.42	3.78143	35.18333	4.42	2.29748	28.45685	4.42	1.24799	22.85732
4.421	8.75125	38.04875	4.421	5.94322	40.85678	4.421	3.78853	35.22797	4.421	2.3034	28.50144	4.421	1.25288	22.90174
4.422	8.75787	38.04213	4.422	5.95112	40.84888	4.422	3.79565	35.27261	4.422	2.30933	28.54604	4.422	1.25778	22.94618
4.423	8.76449	38.03551	4.423	5.95901	40.84099	4.423	3.80277	35.31726	4.423	2.31527	28.59064	4.423	1.26269	22.99061
4.424	8.77111	38.02889	4.424	5.96689	40.83311	4.424	3.8099	35.36191	4.424	2.32121	28.63525	4.424	1.26761	23.03506
4.425	8.77772	38.02228	4.425	5.97477	40.82523	4.425	3.81704	35.40657	4.425	2.32717	28.67987	4.425	1.27253	23.07951
4.426	8.78432	38.01568	4.426	5.98264	40.81736	4.426	3.82418	35.45123	4.426	2.33313	28.72449	4.426	1.27747	23.12396
4.427	8.79093	38.00907	4.427	5.99051	40.80949	4.427	3.83133	35.4959	4.427	2.33909	28.76912	4.427	1.2824	23.16842
4.428	8.79752	38.00248	4.428	5.99837	40.80163	4.428	3.83848	35.54058	4.428	2.34507	28.81375	4.428	1.28735	23.21288
4.429	8.80412	37.99588	4.429	6.00623	40.79377	4.429	3.84565	35.58526	4.429	2.35105	28.85839	4.429	1.29231	23.25735
4.43	8.81071	37.98929	4.43	6.01408	40.78592	4.43	3.85282	35.62995	4.43	2.35704	28.90303	4.43	1.29727	23.30183
4.431	8.81729	37.98271	4.431	6.02193	40.77807	4.431	3.85999	35.67464	4.431	2.36303	28.94768	4.431	1.30224	23.34631
4.432	8.82387	37.97613	4.432	6.02977	40.77023	4.432	3.86718	35.71934	4.432	2.36904	28.99234	4.432	1.30722	23.39079
4.433	8.83045	37.96955	4.433	6.03761	40.76239	4.433	3.87437	35.76404	4.433	2.37505	29.037	4.433	1.31221	23.43528
4.434	8.83702	37.96298	4.434	6.04544	40.75456	4.434	3.88156	35.80875	4.434	2.38106	29.08166	4.434	1.3172	23.47978
4.435	8.84358	37.95642	4.435	6.05326	40.74674	4.435	3.88877	35.85346	4.435	2.38709	29.12633	4.435	1.3222	23.52428
4.436	8.85015	37.94985	4.436	6.06108	40.73892	4.436	3.89598	35.89819	4.436	2.39312	29.17101	4.436	1.32721	23.56878
4.437	8.8567	37.9433	4.437	6.0689	40.7311	4.437	3.9032	35.94291	4.437	2.39916	29.21569	4.437	1.33223	23.6133

4.438	8.86326	37.93674	4.438	6.07671	40.72329	4.438	3.91042	35.98764	4.438	2.40521	29.26038	4.438	1.33726	23.65781
4.439	8.86981	37.93019	4.439	6.08451	40.71549	4.439	3.91765	36.03238	4.439	2.41127	29.30508	4.439	1.34229	23.70234
4.44	8.87635	37.92365	4.44	6.09231	40.70769	4.44	3.92489	36.07713	4.44	2.41733	29.34978	4.44	1.34733	23.74686
4.441	8.88289	37.91711	4.441	6.1001	40.6999	4.441	3.93213	36.12188	4.441	2.4234	29.39448	4.441	1.35238	23.7914
4.442	8.88943	37.91057	4.442	6.10789	40.69211	4.442	3.93939	36.16663	4.442	2.42947	29.43919	4.442	1.35744	23.83594
4.443	8.89596	37.90404	4.443	6.11567	40.68433	4.443	3.94664	36.21139	4.443	2.43556	29.48391	4.443	1.3625	23.88048
4.444	8.90248	37.89752	4.444	6.12345	40.67655	4.444	3.95391	36.25616	4.444	2.44165	29.52863	4.444	1.36757	23.92503
4.445	8.90901	37.89099	4.445	6.13122	40.66878	4.445	3.96118	36.30093	4.445	2.44775	29.57336	4.445	1.37265	23.96958
4.446	8.91552	37.88448	4.446	6.13899	40.66101	4.446	3.96846	36.34571	4.446	2.45385	29.6181	4.446	1.37774	24.01414
4.447	8.92204	37.87796	4.447	6.14675	40.65325	4.447	3.97575	36.3905	4.447	2.45997	29.66284	4.447	1.38284	24.05871
4.448	8.92855	37.87145	4.448	6.15451	40.64549	4.448	3.98304	36.43529	4.448	2.46609	29.70758	4.448	1.38794	24.10328
4.449	8.93505	37.86495	4.449	6.16226	40.63774	4.449	3.99034	36.48008	4.449	2.47222	29.75233	4.449	1.39305	24.14786
4.45	8.94155	37.85845	4.45	6.17001	40.62999	4.45	3.99764	36.52489	4.45	2.47835	29.79709	4.45	1.39817	24.19244
4.451	8.94805	37.85195	4.451	6.17775	40.62225	4.451	4.00496	36.56969	4.451	2.48449	29.84185	4.451	1.40329	24.23703
4.452	8.95454	37.84546	4.452	6.18549	40.61451	4.452	4.01228	36.61451	4.452	2.49064	29.88662	4.452	1.40843	24.28162
4.453	8.96103	37.83897	4.453	6.19322	40.60678	4.453	4.0196	36.65933	4.453	2.4968	29.9314	4.453	1.41357	24.32622
4.454	8.96751	37.83249	4.454	6.20094	40.59906	4.454	4.02694	36.70415	4.454	2.50297	29.97618	4.454	1.41872	24.37082
4.455	8.97399	37.82601	4.455	6.20866	40.59134	4.455	4.03428	36.74898	4.455	2.50914	30.02096	4.455	1.42388	24.41543
4.456	8.98046	37.81954	4.456	6.21638	40.58362	4.456	4.04162	36.79382	4.456	2.51532	30.06576	4.456	1.42904	24.46005
4.457	8.98693	37.81307	4.457	6.22409	40.57591	4.457	4.04898	36.83867	4.457	2.5215	30.11055	4.457	1.43421	24.50467
4.458	8.9934	37.8066	4.458	6.23179	40.56821	4.458	4.05634	36.88351	4.458	2.5277	30.15536	4.458	1.43939	24.5493
4.459	8.99986	37.80014	4.459	6.23949	40.56051	4.459	4.0637	36.92837	4.459	2.5339	30.20017	4.459	1.44458	24.59393
4.46	9.00631	37.79369	4.46	6.24719	40.55281	4.46	4.07108	36.97323	4.46	2.54011	30.24498	4.46	1.44978	24.63857
4.461	9.01277	37.78723	4.461	6.25488	40.54512	4.461	4.07846	37.0181	4.461	2.54632	30.28981	4.461	1.45498	24.68321
4.462	9.01921	37.78079	4.462	6.26256	40.53744	4.462	4.08585	37.06297	4.462	2.55255	30.33463	4.462	1.46019	24.72786
4.463	9.02566	37.77434	4.463	6.27024	40.52976	4.463	4.09324	37.10785	4.463	2.55878	30.37947	4.463	1.46541	24.77251
4.464	9.0321	37.7679	4.464	6.27791	40.52209	4.464	4.10064	37.15273	4.464	2.56501	30.42431	4.464	1.47064	24.81717
4.465	9.03853	37.76147	4.465	6.28558	40.51442	4.465	4.10805	37.19762	4.465	2.57126	30.46915	4.465	1.47587	24.86184
4.466	9.04496	37.75504	4.466	6.29324	40.50676	4.466	4.11546	37.24252	4.466	2.57751	30.514	4.466	1.48111	24.90651
4.467	9.05139	37.74861	4.467	6.3009	40.4991	4.467	4.12288	37.28742	4.467	2.58377	30.55886	4.467	1.48636	24.95119
4.468	9.05781	37.74219	4.468	6.30856	40.49144	4.468	4.13031	37.33233	4.468	2.59003	30.60372	4.468	1.49162	24.99587
4.469	9.06423	37.73577	4.469	6.3162	40.4838	4.469	4.13775	37.37725	4.469	2.59631	30.64859	4.469	1.49688	25.04056
4.47	9.07064	37.72936	4.47	6.32385	40.47615	4.47	4.14519	37.42217	4.47	2.60259	30.69346	4.47	1.50215	25.08526
4.471	9.07705	37.72295	4.471	6.33148	40.46852	4.471	4.15263	37.46709	4.471	2.60888	30.73834	4.471	1.50743	25.12996
4.472	9.08346	37.71654	4.472	6.33912	40.46088	4.472	4.16009	37.51203	4.472	2.61517	30.78323	4.472	1.51272	25.17466
4.473	9.08986	37.71014	4.473	6.34674	40.45326	4.473	4.16755	37.55697	4.473	2.62147	30.82812	4.473	1.51801	25.21937
4.474	9.09625	37.70375	4.474	6.35437	40.44563	4.474	4.17502	37.60191	4.474	2.62778	30.87302	4.474	1.52332	25.26409
4.475	9.10265	37.69735	4.475	6.36198	40.43802	4.475	4.18249	37.64686	4.475	2.6341	30.91793	4.475	1.52863	25.30881
4.476	9.10903	37.69097	4.476	6.3696	40.4304	4.476	4.18997	37.69182	4.476	2.64042	30.96284	4.476	1.53395	25.35354
4.477	9.11542	37.68458	4.477	6.3772	40.4228	4.477	4.19746	37.73678	4.477	2.64676	31.00776	4.477	1.53927	25.39828
4.478	9.1218	37.6782	4.478	6.38481	40.41519	4.478	4.20496	37.78175	4.478	2.65309	31.05268	4.478	1.5446	25.44302
4.479	9.12817	37.67183	4.479	6.3924	40.4076	4.479	4.21246	37.82673	4.479	2.65944	31.09761	4.479	1.54994	25.48776
4.48	9.13454	37.66546	4.48	6.39999	40.40001	4.48	4.21997	37.87171	4.48	2.66579	31.14254	4.48	1.55529	25.53251
4.481	9.14091	37.65909	4.481	6.40758	40.39242	4.481	4.22748	37.91669	4.481	2.67215	31.18748	4.481	1.56065	25.57727
4.482	9.14727	37.65273	4.482	6.41516	40.38484	4.482	4.235	37.96169	4.482	2.67852	31.23243	4.482	1.56601	25.62204
4.483	9.15363	37.64637	4.483	6.42274	40.37726	4.483	4.24253	38.00669	4.483	2.68489	31.27738	4.483	1.57138	25.66681
4.484	9.15998	37.64002	4.484	6.43031	40.36969	4.484	4.25007	38.05169	4.484	2.69128	31.32234	4.484	1.57676	25.71158
4.485	9.16633	37.63367	4.485	6.43788	40.36212	4.485	4.25761	38.0967	4.485	2.69766	31.36731	4.485	1.58215	25.75636
4.486	9.17268	37.62732	4.486	6.44544	40.35456	4.486	4.26516	38.14172	4.486	2.70406	31.41228	4.486	1.58754	25.80115
4.487	9.17902	37.62098	4.487	6.453	40.347	4.487	4.27271	38.18674	4.487	2.71046	31.45726	4.487	1.59294	25.84594
4.488	9.18535	37.61465	4.488	6.46055	40.33945	4.488	4.28027	38.23177	4.488	2.71687	31.50224	4.488	1.59835	25.89074
4.489	9.19169	37.60831	4.489	6.46809	40.33191	4.489	4.28784	38.27681	4.489	2.72329	31.54723	4.489	1.60377	25.93554
4.49	9.19802	37.60198	4.49	6.47563	40.32437	4.49	4.29542	38.32185	4.49	2.72972	31.59222	4.49	1.60919	25.98036
4.491	9.20434	37.59566	4.491	6.48317	40.31683	4.491	4.303	38.3669	4.491	2.73615	31.63723	4.491	1.61462	26.02517
4.492	9.21066	37.58934	4.492	6.4907	40.3093	4.492	4.31059	38.41196	4.492	2.74259	31.68223	4.492	1.62006	26.06999
4.493	9.21697	37.58303	4.493	6.49823	40.30177	4.493	4.31818	38.45702	4.493	2.74903	31.72725	4.493	1.62551	26.11482
4.494	9.22329	37.57671	4.494	6.50575	40.29425	4.494	4.32578	38.50208	4.494	2.75549	31.77227	4.494	1.63096	26.15966
4.495	9.22959	37.57041	4.495	6.51326	40.28674	4.495	4.33339	38.54716	4.495	2.76195	31.8173	4.495	1.63643	26.2045
4.496	9.2359	37.5641	4.496	6.52078	40.27922	4.496	4.34101	38.59223	4.496	2.76841	31.86233	4.496	1.6419	26.24934
4.497	9.24219	37.55781	4.497	6.52828	40.27172	4.497	4.34863	38.63732	4.497	2.77489	31.90737	4.497	1.64737	26.2942
4.498	9.24849	37.55151	4.498	6.53578	40.26422	4.498	4.35626	38.68241	4.498	2.78137	31.95241	4.498	1.65286	26.33905
4.499	9.25478	37.54522	4.499	6.54328	40.25672	4.499	4.36389	38.72751	4.499	2.78786	31.99746	4.499	1.65835	26.38392
4.5	9.26106	37.53894	4.5	6.55077	40.24923	4.5	4.37153	38.77261	4.5	2.79435	32.04252	4.5	1.66385	26.42879
4.501	9.26735	37.53265	4.501	6.55826	40.24174	4.501	4.37918	38.81772	4.501	2.80086	32.08759	4.501	1.66936	26.47366
4.502	9.27362	37.52638	4.502	6.56574	40.23426	4.502	4.38683	38.86284	4.502	2.80737	32.13266	4.502	1.67487	26.51855
4.503	9.2799	37.5201	4.503	6.57321	40.22679	4.503	4.3945	38.90796	4.503	2.81389	32.17773	4.503	1.68039	26.56344
4.504	9.28617	37.51383	4.504	6.58068	40.21932	4.504	4.40216	38.95309	4.504	2.82041	32.22281	4.504	1.68592	26.60833
4.505	9.29243	37.50757	4.505	6.58815	40.21185	4.505	4.40984	38.99823	4.505	2.82694	32.2679	4.505	1.69146	26.65323
4.506	9.29869	37.50131	4.506	6.59561	40.20439	4.506	4.41752	39.04337	4.506	2.83348	32.313	4.506	1.697	26.69814
4.507	9.30495	37.49505	4.507	6.60307	40.19693	4.507	4.42521	39.08852	4.507	2.84003	32.3581	4.507	1.70256	26.74305
4.508	9.3112	37.4888	4.508	6.61052	40.18948	4.508	4.4329	39.13367	4.508	2.84658	32.40321	4.508	1.70812	26.78797
4.509	9.31745	37.48255	4.509	6.61797	40.18203	4.509	4.4406	39.17883	4.509	2.85314	32.44832	4.509	1.71368	26.83289
4.51	9.3237	37.4763	4.51	6.62541	40.17459	4.51	4.44831	39.224	4.51	2.85971	32.49344	4.51	1.71926	26.87782
4.511	9.32994	37.47006	4.511	6.63284	40.16716	4.511	4.45602	39.26917	4.511	2.86628	32.53857	4.511	1.72484	26.92276
4.512	9.33617	37.46383	4.512	6.64027	40.15973	4.512	4.46374	39.31435	4.512	2.87286	32.5837	4.512	1.73043	26.9677
4.513	9.3424	37.4576	4.513	6.6477	40.1523	4.513	4.47147	39.35954	4.513	2.87945	32.62884	4.513	1.73603	27.01265
4.514	9.34863	37.45137	4.514	6.65512	40.14488	4.514	4.4792	39.40473	4.514	2.88605	32.67399	4.514	1.74163	27.05761
4.515	9.35486	37.44514	4.515	6.66254	40.13746	4.515	4.48695	39.44993	4.515	2.89265	32.71914	4.515	1.74724	27.10257
4.516	9.36107	37.43893	4.516	6.66995	40.13005	4.516	4.49469	39.49513	4.516	2.89926	32.7643	4.516	1.75286	27.14754
4.517	9.36729	37.43271	4.517	6.67736	40.12264	4.517	4.50245	39.54034	4.517	2.90587	32.80946	4.517	1.75849	27.19251
4.518	9.3735	37.4265	4.518	6.68476	40.11524	4.518	4.51021	39.58556	4.518	2.9125	32.85463	4.518	1.76412	27.23749
4.519	9.37971	37.42029	4.519	6.69216	40.10784	4.519	4.51797	39.63078	4.519	2.91913	32.89981	4.519	1.76977	27.28248
4.52	9.38591	37.41409	4.52	6.69955	40.10045	4.52	4.52575	39.67601	4.52	2.92577	32.945	4.52	1.77542	27.32747
4.521	9.39211	37.40789	4.521	6.70694	40.09306	4.521	4.53353	39.72125	4.521	2.93241	32.99019	4.521	1.78107	27.37247
4.522	9.3983	37.4017	4.522	6.71432	40.08568	4.522	4.54131	39.75845	4.522	2.93906	33.03538	4.522	1.78674	27.41747
4.523	9.4045	37.3955	4.523	6.7217	40.0783	4.523	4.5491	39.78494	4.523	2.94572	33.08059	4.523	1.79241	27.46249
4.524	9.41068	37.38932	4.524	6.72907	40.07093	4.524	4.55689	39.81143	4.524	2.95239	33.1258	4.524	1.79809	27.5075
4.525	9.41686	37.38314	4.525	6.73644	40.06356	4.525	4.56469	39.83793	4.525	2.95906	33.17101	4.525	1.80377	27.55253
4.526	9.42304	37.37696	4.526	6.7438	40.0562	4.526	4.57248	39.86444	4.526	2.96574	33.21623	4.526	1.80947	27.59756
4.527	9.42922	37.37078	4.527	6.75116	40.04884	4.527	4.58028	39.89095	4.527	2.97243	33.26146	4.527	1.81517	27.64259
4.528	9.43539	37.36461	4.528	6.75851	40.04149	4.528	4.58808	39.91748	4.528	2.97912	33.3067	4.528	1.82088	27.68764
4.529	9.44155	37.35845	4.529	6.76586	40.03414	4.529	4.59589	39.94401	4.529	2.98582	33.35194	4.529	1.8266	27.73268
4.53	9.44772	37.35228	4.53	6.7732	40.0268	4.53	4.60369	39.97055	4.53	2.99253	33.39719	4.53	1.83232	27.77774
4.531	9.45387	37.34613	4.531	6.78054	40.01946	4.531	4.6115	39.9971	4.531	2.99925	33.44244	4.531	1.83805	27.8228
4.532	9.46003	37.33997	4.532	6.78787	40.01213	4.532	4.61931	40.02366	4.532	3.00597	33.4877	4.532	1.84379	27.86787
4.533	9.46618	37.33382	4.533	6.7952	40.0048	4.533	4.62713	40.05022	4.533	3.0127	33.53297	4.533	1.84953	27.91294
4.534	9.47232	37.32768	4.534	6.80252	39.99748	4.534	4.63494	40.07679	4.534	3.01944	33.57825	4.534	1.85529	27.95802
4.535	9.47846	37.32154	4.535	6.80984	39.99016	4.535	4.64276	40.10338	4.535	3.02618	33.62353	4.535	1.86105	28.00311
4.536	9.4846	37.3154	4.536	6.81716	39.98284	4.536	4.65058	40.12997	4.536	3.03293	33.66882	4.536	1.86682	28.0482
4.537	9.49074	37.30926	4.537	6.82447	39.97553	4.537	4.6584	40.15656	4.537	3.03969	33.71411	4.537	1.87259	28.0933
4.538	9.49686	37.30314	4.538	6.83177	39.96823	4.538	4.66623	40.18317	4.538	3.04645	33.75941	4.538	1.87838	28.13841
4.539	9.50299	37.29701	4.539	6.83907	39.96093	4.539	4.67406	40.20978	4.539	3.05322	33.80472	4.539	1.88417	28.18352
4.54	9.50911	37.29089	4.54	6.84636	39.95364	4.54	4.68189	40.23641	4.54	3.06	33.85003	4.54	1.88996	28.22864
4.541	9.51523	37.28477	4.541	6.85365	39.94635	4.541	4.68972	40.26304	4.541	3.06679	33.89535	4.541	1.89577	28.27377
4.542	9.52134	37.27866	4.542	6.86094	39.93906	4.542	4.69755	40.28968	4.542	3.07358	33.94068	4.542	1.90158	28.3189
4.543	9.52745	37.27255	4.543	6.86822	39.93178	4.543	4.70539	40.31632	4.543	3.08038	33.98601	4.543	1.9074	28.36404
4.544	9.53356	37.26644	4.544	6.8755	39.9245	4.544	4.71323	40.34298	4.544	3.08719	34.03135	4.544	1.91323	28.40918
4.545	9.53966	37.26034	4.545	6.88277	39.91723	4.545	4.72107	40.36964	4.545	3.094	34.0767	4.545	1.91906	28.45433
4.546	9.54575	37.25425	4.546	6.89003	39.90997	4.546	4.72892	40.39631	4.546	3.10082	34.12205	4.546	1.92491	28.49949
4.547	9.55185	37.24815	4.547	6.89729	39.90271	4.547	4.73677	40.42299	4.547	3.10765	34.16741	4.547	1.93076	28.54465
4.548	9.55794	37.24206	4.548	6.90455	39.89545	4.548	4.74461	40.44968	4.548	3.11448	34.21278	4.548	1.93661	28.58982
4.549	9.56402	37.23598	4.549	6.9118	39.8882	4.549	4.75247	40.47638	4.549	3.12132	34.25815	4.549	1.94248	28.635
4.55	9.5701	37.2299	4.55	6.91905	39.88095	4.55	4.76032	40.50308	4.55	3.12817	34.30353	4.55	1.94835	28.68019
4.551	9.57618	37.22382	4.551	6.92629	39.87371	4.551	4.76818	40.5298	4.551	3.13503	34.34892	4.551	1.95423	28.72538
4.552	9.58225	37.21775	4.552	6.93353	39.86647	4.552	4.77604	40.55652	4.552	3.14189	34.39431	4.552	1.96011	28.77057
4.553	9.58832	37.21168	4.553	6.94076	39.85924	4.553	4.7839	40.58325	4.553	3.14876	34.43971	4.553	1.96601	28.81578
4.554	9.59439	37.20561	4.554	6.94799	39.85201	4.554	4.79176	40.60999	4.554	3.15563	34.48512	4.554	1.97191	28.86098
4.555	9.60045	37.19955	4.555	6.95521	39.84479	4.555	4.79963	40.63673	4.555	3.16252	34.53053	4.555	1.97781	28.9062
4.556	9.60651	37.19349	4.556	6.96243	39.83757	4.556	4.8075	40.66349	4.556	3.16941	34.57595	4.556	1.98373	28.95142
4.557	9.61256	37.18744	4.557	6.96964	39.83036	4.557	4.81537	40.69025	4.557	3.1763	34.62138	4.557	1.98965	28.99665
4.558	9.61861	37.18139	4.558	6.97685	39.82315	4.558	4.82324	40.71702	4.558	3.18321	34.66681	4.558	1.99558	29.04189
4.559	9.62465	37.17535	4.559	6.98405	39.81595	4.559	4.83112	40.7438	4.559	3.19012	34.71225	4.559	2.00152	29.08713
4.56	9.63069	37.16931	4.56	6.99125	39.80875	4.56	4.839	40.77058	4.56	3.19704	34.7577	4.56	2.00747	29.13238
4.561	9.63673	37.16327	4.561	6.99845	39.80155	4.561	4.84688	40.79738	4.561	3.20396	34.80315	4.561	2.01342	29.17764
4.562	9.64276	37.15724	4.562	7.00564	39.79436	4.562	4.85476	40.82418	4.562	3.21089	34.84861	4.562	2.01938	29.2229
4.563	9.64879	37.15121	4.563	7.01282	39.78718	4.563	4.86265	40.85099	4.563	3.21783	34.89408	4.563	2.02534	29.26817
4.564	9.65482	37.14518	4.564	7.02	39.78	4.564	4.87054	40.87781	4.564	3.22478	34.93955	4.564	2.03132	29.31344
4.565	9.66084	37.13916	4.565	7.02718	39.77282	4.565	4.87843	40.90464	4.565	3.23173	34.98503	4.565	2.0373	29.35873
4.566	9.66686	37.13314	4.566	7.03435	39.76565	4.566	4.88632	40.93148	4.566	3.23869	35.03052	4.566	2.04329	29.40401
4.567	9.67287	37.12713	4.567	7.04152	39.75848	4.567	4.89421	40.95832	4.567	3.24565	35.07601	4.567	2.04928	29.44931
4.568	9.67888	37.12112	4.568	7.04868	39.75132	4.568	4.90211	40.98518	4.568	3.25263	35.12152	4.568	2.05529	29.49461
4.569	9.68489	37.11511	4.569	7.05583	39.74417	4.569	4.91001	41.01204	4.569	3.25961	35.16702	4.569	2.0613	29.53992
4.57	9.69089	37.10911	4.57	7.06299	39.73701	4.57	4.91792	41.03891	4.57	3.26659	35.21254	4.57	2.06732	29.58524
4.571	9.69689	37.10311	4.571	7.07013	39.72987	4.571	4.92582	41.06578	4.571	3.27359	35.25806	4.571	2.07334	29.63056
4.572	9.70288	37.09712	4.572	7.07728	39.72272	4.572	4.93373	41.09267	4.572	3.28059	35.30359	4.572	2.07937	29.67589
4.573	9.70887	37.09113	4.573	7.08441	39.71559	4.573	4.94164	41.11956	4.573	3.2876	35.34912	4.573	2.08541	29.72122
4.574	9.71485	37.08515	4.574	7.09155	39.70845	4.574	4.94955	41.14647	4.574	3.29461	35.39466	4.574	2.09146	29.76657
4.575	9.72084	37.07916	4.575	7.09868	39.70132	4.575	4.95747	41.17338	4.575	3.30163	35.44021	4.575	2.09751	29.81192
4.576	9.72681	37.07319	4.576	7.1058	39.6942	4.576	4.96538	41.2003	4.576	3.30866	35.48577	4.576	2.10357	29.85727
4.577	9.73279	37.06721	4.577	7.11292	39.68708	4.577	4.9733	41.22722	4.577	3.3157	35.53133	4.577	2.10964	29.90263
4.578	9.73876	37.06124	4.578	7.12003	39.67997	4.578	4.98122	41.25416	4.578	3.32274	35.5769	4.578	2.11572	29.948
4.579	9.74473	37.05527	4.579	7.12714	39.67286	4.579	4.98915	41.2811	4.579	3.32979	35.62248	4.579	2.1218	29.99338
4.58	9.75069	37.04931	4.58	7.13425	39.66575	4.58	4.99708	41.30805	4.58	3.33684	35.66806	4.58	2.12789	30.03876
4.581	9.75665	37.04335	4.581	7.14135	39.65865	4.581	5.00501	41.33501	4.581	3.3439	35.71365	4.581	2.13399	30.08415
4.582	9.7626	37.0374	4.582	7.14845	39.65155	4.582	5.01294	41.36198	4.582	3.35097	35.75925	4.582	2.1401	30.12955
4.583	9.76855	37.03145	4.583	7.15554	39.64446	4.583	5.02087	41.38896	4.583	3.35805	35.80485	4.583	2.14621	30.17495
4.584	9.7745	37.0255	4.584	7.16262	39.63738	4.584	5.02881	41.41594	4.584	3.36513	35.85046	4.584	2.15233	30.22036
4.585	9.78044	37.01956	4.585	7.16971	39.63029	4.585	5.03675	41.44293	4.585	3.37222	35.89608	4.585	2.15845	30.26578
4.586	9.78638	37.01362	4.586	7.17678	39.62322	4.586	5.04469	41.46994	4.586	3.37932	35.94171	4.586	2.16459	30.31121
4.587	9.79231	37.00769	4.587	7.18386	39.61614	4.587	5.05263	41.49694	4.587	3.38642	35.98734	4.587	2.17073	30.35664
4.588	9.79825	37.00175	4.588	7.19092	39.60908	4.588	5.06058	41.52396	4.588	3.39354	36.03298	4.588	2.17688	30.40207
4.589	9.80417	36.99583	4.589	7.19799	39.60201	4.589	5.06853	41.55099	4.589	3.40065	36.07862	4.589	2.18303	30.44752
4.59	9.8101	36.9899	4.59	7.20505	39.59495	4.59	5.07648	41.57802	4.59	3.40778	36.12427	4.59	2.1892	30.49297
4.591	9.81602	36.98398	4.591	7.2121	39.5879	4.591	5.08443	41.60506	4.591	3.41491	36.16993	4.591	2.19537	30.53843
4.592	9.82193	36.97807	4.592	7.21915	39.58085	4.592	5.09239	41.63211	4.592	3.42205	36.2156	4.592	2.20154	30.5839
4.593	9.82784	36.97216	4.593	7.2262	39.5738	4.593	5.10035	41.65917	4.593	3.42919	36.26127	4.593	2.20773	30.62937
4.594	9.83375	36.96625	4.594	7.23324	39.56676	4.594	5.10831	41.68624	4.594	3.43634	36.30695	4.594	2.21392	30.67485
4.595	9.83966	36.96034	4.595	7.24027	39.55973	4.595	5.11627	41.68373	4.595	3.4435	36.35264	4.595	2.22012	30.72033
4.596	9.84556	36.95444	4.596	7.2473	39.5527	4.596	5.12422	41.67578	4.596	3.45067	36.39834	4.596	2.22632	30.76583
4.597	9.85145	36.94855	4.597	7.25433	39.54567	4.597	5.13217	41.66783	4.597	3.45784	36.44404	4.597	2.23254	30.81133

4.598	9.85735	36.94265	4.598	7.26135	39.53865	4.598	5.14012	41.65988	4.598	3.46502	36.48975	4.598	2.23876	30.85683
4.599	9.86323	36.93677	4.599	7.26837	39.53163	4.599	5.14806	41.65194	4.599	3.4722	36.53546	4.599	2.24499	30.90235
4.6	9.86912	36.93088	4.6	7.27538	39.52462	4.6	5.15599	41.64401	4.6	3.4794	36.58119	4.6	2.25122	30.94787
4.601	9.875	36.925	4.601	7.28239	39.51761	4.601	5.16392	41.63608	4.601	3.4866	36.62692	4.601	2.25746	30.9934
4.602	9.88088	36.91912	4.602	7.28939	39.51061	4.602	5.17185	41.62815	4.602	3.4938	36.67265	4.602	2.26371	31.03893
4.603	9.88675	36.91325	4.603	7.29639	39.50361	4.603	5.17977	41.62023	4.603	3.50102	36.7184	4.603	2.26997	31.08447
4.604	9.89262	36.90738	4.604	7.30339	39.49661	4.604	5.18768	41.61232	4.604	3.50824	36.76415	4.604	2.27623	31.13002
4.605	9.89849	36.90151	4.605	7.31038	39.48962	4.605	5.19559	41.60441	4.605	3.51546	36.80991	4.605	2.2825	31.17558
4.606	9.90435	36.89565	4.606	7.31736	39.48264	4.606	5.20349	41.59651	4.606	3.5227	36.85567	4.606	2.28878	31.22114
4.607	9.91021	36.88979	4.607	7.32435	39.47565	4.607	5.21139	41.58861	4.607	3.52994	36.90144	4.607	2.29506	31.26671
4.608	9.91606	36.88394	4.608	7.33132	39.46868	4.608	5.21929	41.58071	4.608	3.53718	36.94722	4.608	2.30136	31.31229
4.609	9.92191	36.87809	4.609	7.33829	39.46171	4.609	5.22717	41.57283	4.609	3.54444	36.99301	4.609	2.30766	31.35787
4.61	9.92776	36.87224	4.61	7.34526	39.45474	4.61	5.23506	41.56494	4.61	3.5517	37.0388	4.61	2.31396	31.40346
4.611	9.9336	36.8664	4.611	7.35222	39.44778	4.611	5.24294	41.55706	4.611	3.55896	37.0846	4.611	2.32028	31.44906
4.612	9.93944	36.86056	4.612	7.35918	39.44082	4.612	5.25081	41.54919	4.612	3.56624	37.13041	4.612	2.3266	31.49467
4.613	9.94528	36.85472	4.613	7.36614	39.43386	4.613	5.25868	41.54132	4.613	3.57352	37.17623	4.613	2.33293	31.54028
4.614	9.95111	36.84889	4.614	7.37309	39.42691	4.614	5.26654	41.53346	4.614	3.58081	37.22205	4.614	2.33926	31.5859
4.615	9.95693	36.84307	4.615	7.38003	39.41997	4.615	5.2744	41.5256	4.615	3.5881	37.26788	4.615	2.3456	31.63152
4.616	9.96276	36.83724	4.616	7.38697	39.41303	4.616	5.28225	41.51775	4.616	3.5954	37.31372	4.616	2.35195	31.67716
4.617	9.96858	36.83142	4.617	7.39391	39.40609	4.617	5.2901	41.5099	4.617	3.60271	37.35956	4.617	2.35831	31.7228
4.618	9.97439	36.82561	4.618	7.40084	39.39916	4.618	5.29794	41.50206	4.618	3.61003	37.40541	4.618	2.36467	31.76845
4.619	9.98021	36.81979	4.619	7.40777	39.39223	4.619	5.30578	41.49422	4.619	3.61735	37.45127	4.619	2.37104	31.8141
4.62	9.98602	36.81398	4.62	7.41469	39.38531	4.62	5.31361	41.48639	4.62	3.62468	37.49713	4.62	2.37742	31.85977
4.621	9.99182	36.80818	4.621	7.42161	39.37839	4.621	5.32144	41.47856	4.621	3.63201	37.54301	4.621	2.38381	31.90543
4.622	9.99762	36.80238	4.622	7.42852	39.37148	4.622	5.32926	41.47074	4.622	3.63935	37.58889	4.622	2.3902	31.95111
4.623	10.00342	36.79658	4.623	7.43543	39.36457	4.623	5.33708	41.46292	4.623	3.6467	37.63477	4.623	2.3966	31.9968
4.624	10.00921	36.79079	4.624	7.44233	39.35767	4.624	5.34489	41.45511	4.624	3.65406	37.68067	4.624	2.403	32.04249
4.625	10.015	36.785	4.625	7.44923	39.35077	4.625	5.35269	41.44731	4.625	3.66142	37.72657	4.625	2.40942	32.08818
4.626	10.02079	36.77921	4.626	7.45613	39.34387	4.626	5.3605	41.4395	4.626	3.66879	37.77248	4.626	2.41584	32.13389
4.627	10.02657	36.77343	4.627	7.46302	39.33698	4.627	5.36829	41.43171	4.627	3.67616	37.8184	4.627	2.42227	32.1796
4.628	10.03235	36.76765	4.628	7.4699	39.3301	4.628	5.37608	41.42392	4.628	3.68355	37.86432	4.628	2.4287	32.22532
4.629	10.03813	36.76187	4.629	7.47679	39.32321	4.629	5.38387	41.41613	4.629	3.69093	37.91025	4.629	2.43514	32.27105
4.63	10.0439	36.7561	4.63	7.48366	39.31634	4.63	5.39165	41.40835	4.63	3.69833	37.95619	4.63	2.44159	32.31678
4.631	10.04966	36.75034	4.631	7.49054	39.30946	4.631	5.39943	41.40057	4.631	3.70573	38.00213	4.631	2.44805	32.36253
4.632	10.05543	36.74457	4.632	7.49741	39.30259	4.632	5.4072	41.3928	4.632	3.71314	38.04809	4.632	2.45451	32.40828
4.633	10.06119	36.73881	4.633	7.50427	39.29573	4.633	5.41497	41.38503	4.633	3.72056	38.09405	4.633	2.46098	32.45403
4.634	10.06694	36.73306	4.634	7.51113	39.28887	4.634	5.42273	41.37727	4.634	3.72798	38.14001	4.634	2.46746	32.49979
4.635	10.0727	36.7273	4.635	7.51798	39.28202	4.635	5.43048	41.36952	4.635	3.73541	38.18599	4.635	2.47394	32.54557
4.636	10.07845	36.72155	4.636	7.52484	39.27516	4.636	5.43824	41.36176	4.636	3.74285	38.23197	4.636	2.48043	32.59134
4.637	10.08419	36.71581	4.637	7.53168	39.26832	4.637	5.44598	41.35402	4.637	3.75029	38.27796	4.637	2.48693	32.63713
4.638	10.08993	36.71007	4.638	7.53852	39.26148	4.638	5.45372	41.34628	4.638	3.75774	38.32396	4.638	2.49344	32.68292
4.639	10.09567	36.70433	4.639	7.54536	39.25464	4.639	5.46146	41.33854	4.639	3.76519	38.36996	4.639	2.49995	32.72872
4.64	10.1014	36.6986	4.64	7.55219	39.24781	4.64	5.46919	41.33081	4.64	3.77266	38.41597	4.64	2.50647	32.77453
4.641	10.10714	36.69286	4.641	7.55902	39.24098	4.641	5.47692	41.32308	4.641	3.78013	38.46199	4.641	2.513	32.82034
4.642	10.11286	36.68714	4.642	7.56585	39.23415	4.642	5.48464	41.31536	4.642	3.7876	38.50802	4.642	2.51953	32.86617
4.643	10.11858	36.68142	4.643	7.57267	39.22733	4.643	5.49235	41.30765	4.643	3.79509	38.55405	4.643	2.52607	32.912
4.644	10.1243	36.6757	4.644	7.57948	39.22052	4.644	5.50007	41.29993	4.644	3.80258	38.60009	4.644	2.53262	32.95783
4.645	10.13002	36.66998	4.645	7.58629	39.21371	4.645	5.50777	41.29223	4.645	3.81007	38.64614	4.645	2.53917	33.00368
4.646	10.13573	36.66427	4.646	7.5931	39.2069	4.646	5.51547	41.28453	4.646	3.81758	38.69219	4.646	2.54574	33.04953
4.647	10.14144	36.65856	4.647	7.5999	39.2001	4.647	5.52317	41.27683	4.647	3.82508	38.73826	4.647	2.55231	33.09539
4.648	10.14714	36.65286	4.648	7.6067	39.1933	4.648	5.53086	41.26914	4.648	3.8326	38.78433	4.648	2.55888	33.14125
4.649	10.15284	36.64716	4.649	7.6135	39.1865	4.649	5.53855	41.26145	4.649	3.84012	38.83041	4.649	2.56547	33.18713
4.65	10.15854	36.64146	4.65	7.62028	39.17972	4.65	5.54623	41.25377	4.65	3.84765	38.87649	4.65	2.57206	33.23301
4.651	10.16423	36.63577	4.651	7.62707	39.17293	4.651	5.55391	41.24609	4.651	3.85519	38.92258	4.651	2.57865	33.2789
4.652	10.16992	36.63008	4.652	7.63385	39.16615	4.652	5.56158	41.23842	4.652	3.86273	38.96868	4.652	2.58526	33.32479
4.653	10.17561	36.62439	4.653	7.64063	39.15937	4.653	5.56924	41.23076	4.653	3.87028	39.01479	4.653	2.59187	33.3707
4.654	10.18129	36.61871	4.654	7.6474	39.1526	4.654	5.57691	41.22309	4.654	3.87784	39.06091	4.654	2.59849	33.41661
4.655	10.18697	36.61303	4.655	7.65416	39.14584	4.655	5.58456	41.21544	4.655	3.8854	39.10703	4.655	2.60511	33.46253
4.656	10.19265	36.60735	4.656	7.66093	39.13907	4.656	5.59221	41.20779	4.656	3.89297	39.15316	4.656	2.61174	33.50845
4.657	10.19832	36.60168	4.657	7.66769	39.13231	4.657	5.59986	41.20014	4.657	3.90055	39.1993	4.657	2.61838	33.55439
4.658	10.20399	36.59601	4.658	7.67444	39.12556	4.658	5.6075	41.1925	4.658	3.90813	39.24544	4.658	2.62503	33.60033
4.659	10.20965	36.59035	4.659	7.68119	39.11881	4.659	5.61514	41.18486	4.659	3.91572	39.2916	4.659	2.63168	33.64627
4.66	10.21531	36.58469	4.66	7.68794	39.11206	4.66	5.62277	41.17723	4.66	3.92332	39.33776	4.66	2.63834	33.69223
4.661	10.22097	36.57903	4.661	7.69468	39.10532	4.661	5.6304	41.1696	4.661	3.93092	39.38392	4.661	2.64501	33.73819
4.662	10.22662	36.57338	4.662	7.70141	39.09859	4.662	5.63802	41.16198	4.662	3.93853	39.4301	4.662	2.65169	33.78416
4.663	10.23227	36.56773	4.663	7.70815	39.09185	4.663	5.64564	41.15436	4.663	3.94615	39.47628	4.663	2.65837	33.83014
4.664	10.23792	36.56208	4.664	7.71487	39.08513	4.664	5.65325	41.14675	4.664	3.95377	39.52247	4.664	2.66506	33.87613
4.665	10.24356	36.55644	4.665	7.7216	39.0784	4.665	5.66086	41.13914	4.665	3.9614	39.56867	4.665	2.67175	33.92212
4.666	10.2492	36.5508	4.666	7.72832	39.07168	4.666	5.66846	41.13154	4.666	3.96904	39.61488	4.666	2.67845	33.96812
4.667	10.25483	36.54517	4.667	7.73503	39.06497	4.667	5.67606	41.12394	4.667	3.97668	39.66109	4.667	2.68516	34.01413
4.668	10.26046	36.53954	4.668	7.74174	39.05826	4.668	5.68365	41.11635	4.668	3.98433	39.70731	4.668	2.69188	34.06015
4.669	10.26609	36.53391	4.669	7.74845	39.05155	4.669	5.69124	41.10876	4.669	3.99198	39.75354	4.669	2.6986	34.10617
4.67	10.27172	36.52828	4.67	7.75515	39.04485	4.67	5.69882	41.10118	4.67	3.99965	39.79977	4.67	2.70533	34.1522
4.671	10.27734	36.52266	4.671	7.76185	39.03815	4.671	5.7064	41.0936	4.671	4.00732	39.84602	4.671	2.71207	34.19824
4.672	10.28295	36.51705	4.672	7.76854	39.03146	4.672	5.71398	41.08602	4.672	4.01499	39.89227	4.672	2.71881	34.24429
4.673	10.28857	36.51143	4.673	7.77523	39.02477	4.673	5.72155	41.07845	4.673	4.02267	39.93853	4.673	2.72557	34.29034
4.674	10.29418	36.50582	4.674	7.78192	39.01808	4.674	5.72911	41.07089	4.674	4.03036	39.98479	4.674	2.73232	34.33641
4.675	10.29978	36.50022	4.675	7.7886	39.0114	4.675	5.73667	41.06333	4.675	4.03806	40.03107	4.675	2.73909	34.38248
4.676	10.30539	36.49461	4.676	7.79528	39.00472	4.676	5.74422	41.05578	4.676	4.04576	40.07735	4.676	2.74586	34.42855
4.677	10.31098	36.48902	4.677	7.80195	38.99805	4.677	5.75177	41.04823	4.677	4.05347	40.12364	4.677	2.75264	34.47464
4.678	10.31658	36.48342	4.678	7.80862	38.99138	4.678	5.75932	41.04068	4.678	4.06119	40.16993	4.678	2.75943	34.52073
4.679	10.32217	36.47783	4.679	7.81528	38.98472	4.679	5.76686	41.03314	4.679	4.06891	40.21624	4.679	2.76622	34.56683
4.68	10.32776	36.47224	4.68	7.82194	38.97806	4.68	5.77439	41.02561	4.68	4.07664	40.24534	4.68	2.77302	34.61294
4.681	10.33334	36.46666	4.681	7.8286	38.9714	4.681	5.78192	41.01808	4.681	4.08436	40.27274	4.681	2.77983	34.65905
4.682	10.33893	36.46107	4.682	7.83525	38.96475	4.682	5.78944	41.01056	4.682	4.0921	40.30015	4.682	2.78664	34.70518
4.683	10.3445	36.4555	4.683	7.84189	38.95811	4.683	5.79697	41.00303	4.683	4.09983	40.32758	4.683	2.79346	34.75131
4.684	10.35008	36.44992	4.684	7.84853	38.95147	4.684	5.80448	40.99552	4.684	4.10757	40.35501	4.684	2.80029	34.79745
4.685	10.35565	36.44435	4.685	7.85517	38.94483	4.685	5.81199	40.98801	4.685	4.11531	40.38245	4.685	2.80712	34.84359
4.686	10.36121	36.43879	4.686	7.86181	38.93819	4.686	5.8195	40.9805	4.686	4.12305	40.4099	4.686	2.81396	34.88975
4.687	10.36678	36.43322	4.687	7.86844	38.93156	4.687	5.827	40.973	4.687	4.1308	40.43736	4.687	2.82081	34.93591
4.688	10.37234	36.42766	4.688	7.87506	38.92494	4.688	5.83449	40.96551	4.688	4.13854	40.46483	4.688	2.82767	34.98208
4.689	10.37789	36.42211	4.689	7.88168	38.91832	4.689	5.84199	40.95801	4.689	4.14629	40.49231	4.689	2.83453	35.02826
4.69	10.38345	36.41655	4.69	7.8883	38.9117	4.69	5.84947	40.95053	4.69	4.15405	40.51979	4.69	2.8414	35.07444
4.691	10.38899	36.41101	4.691	7.89491	38.90509	4.691	5.85695	40.94305	4.691	4.1618	40.54729	4.691	2.84828	35.12063
4.692	10.39454	36.40546	4.692	7.90152	38.89848	4.692	5.86443	40.93557	4.692	4.16956	40.57479	4.692	2.85516	35.16683
4.693	10.40008	36.39992	4.693	7.90813	38.89187	4.693	5.8719	40.9281	4.693	4.17732	40.60231	4.693	2.86205	35.21304
4.694	10.40562	36.39438	4.694	7.91473	38.88527	4.694	5.87937	40.92063	4.694	4.18508	40.62983	4.694	2.86895	35.25926
4.695	10.41115	36.38885	4.695	7.92132	38.87868	4.695	5.88683	40.91317	4.695	4.19285	40.65736	4.695	2.87585	35.30548
4.696	10.41669	36.38331	4.696	7.92791	38.87209	4.696	5.89429	40.90571	4.696	4.20062	40.6849	4.696	2.88276	35.35172
4.697	10.42221	36.37779	4.697	7.9345	38.8655	4.697	5.90174	40.89826	4.697	4.20839	40.71245	4.697	2.88968	35.39795
4.698	10.42774	36.37226	4.698	7.94108	38.85892	4.698	5.90919	40.89081	4.698	4.21616	40.74001	4.698	2.8966	35.4442
4.699	10.43326	36.36674	4.699	7.94766	38.85234	4.699	5.91664	40.88336	4.699	4.22393	40.76758	4.699	2.90353	35.49046
4.7	10.43878	36.36122	4.7	7.95424	38.84576	4.7	5.92408	40.87592	4.7	4.23171	40.79516	4.7	2.91047	35.53672
4.701	10.44429	36.35571	4.701	7.96081	38.83919	4.701	5.93151	40.86849	4.701	4.23949	40.82274	4.701	2.91742	35.58299
4.702	10.4498	36.3502	4.702	7.96737	38.83263	4.702	5.93894	40.86106	4.702	4.24728	40.85034	4.702	2.92437	35.62927
4.703	10.45531	36.34469	4.703	7.97394	38.82606	4.703	5.94636	40.85364	4.703	4.25506	40.87794	4.703	2.93133	35.67556
4.704	10.46081	36.33919	4.704	7.98049	38.81951	4.704	5.95378	40.84622	4.704	4.26285	40.90556	4.704	2.93829	35.72185
4.705	10.46631	36.33369	4.705	7.98705	38.81295	4.705	5.9612	40.8388	4.705	4.27064	40.93318	4.705	2.94527	35.76816
4.706	10.47181	36.32819	4.706	7.9936	38.8064	4.706	5.96861	40.83139	4.706	4.27844	40.96081	4.706	2.95225	35.81447
4.707	10.4773	36.3227	4.707	8.00014	38.79986	4.707	5.97602	40.82398	4.707	4.28623	40.98845	4.707	2.95923	35.86078
4.708	10.48279	36.31721	4.708	8.00668	38.79332	4.708	5.98342	40.81658	4.708	4.29403	41.0161	4.708	2.96623	35.90711
4.709	10.48827	36.31173	4.709	8.01322	38.78678	4.709	5.99081	40.80919	4.709	4.30183	41.04376	4.709	2.97323	35.95344
4.71	10.49375	36.30625	4.71	8.01975	38.78025	4.71	5.9982	40.8018	4.71	4.30964	41.07143	4.71	2.98023	35.99979
4.711	10.49923	36.30077	4.711	8.02628	38.77372	4.711	6.00559	40.79441	4.711	4.31744	41.09911	4.711	2.98725	36.04614
4.712	10.50471	36.29529	4.712	8.03281	38.76719	4.712	6.01297	40.78703	4.712	4.32525	41.12679	4.712	2.99427	36.09249
4.713	10.51018	36.28982	4.713	8.03933	38.76067	4.713	6.02035	40.77965	4.713	4.33306	41.15449	4.713	3.0013	36.13886
4.714	10.51565	36.28435	4.714	8.04584	38.75416	4.714	6.02772	40.77228	4.714	4.34088	41.18219	4.714	3.00833	36.18523
4.715	10.52111	36.27889	4.715	8.05236	38.74764	4.715	6.03509	40.76491	4.715	4.34869	41.2099	4.715	3.01537	36.23162
4.716	10.52657	36.27343	4.716	8.05887	38.74113	4.716	6.04246	40.75754	4.716	4.35651	41.23763	4.716	3.02242	36.27801
4.717	10.53203	36.26797	4.717	8.06537	38.73463	4.717	6.04981	40.75019	4.717	4.36433	41.26536	4.717	3.02948	36.3244
4.718	10.53749	36.26251	4.718	8.07187	38.72813	4.718	6.05717	40.74283	4.718	4.37216	41.2931	4.718	3.03654	36.37081
4.719	10.54294	36.25706	4.719	8.07836	38.72164	4.719	6.06452	40.73548	4.719	4.37998	41.32085	4.719	3.04361	36.41722
4.72	10.54838	36.25162	4.72	8.08486	38.71514	4.72	6.07186	40.72814	4.72	4.38781	41.34861	4.72	3.05068	36.46365
4.721	10.55383	36.24617	4.721	8.09134	38.70866	4.721	6.0792	40.7208	4.721	4.39565	41.37637	4.721	3.05776	36.51008
4.722	10.55927	36.24073	4.722	8.09783	38.70217	4.722	6.08654	40.71346	4.722	4.40348	41.40415	4.722	3.06485	36.55651
4.723	10.56471	36.23529	4.723	8.10431	38.69569	4.723	6.09387	40.70613	4.723	4.41132	41.43194	4.723	3.07195	36.60296
4.724	10.57014	36.22986	4.724	8.11078	38.68922	4.724	6.1012	40.6988	4.724	4.41916	41.45973	4.724	3.07905	36.64941
4.725	10.57557	36.22443	4.725	8.11725	38.68275	4.725	6.10852	40.69148	4.725	4.427	41.48754	4.725	3.08616	36.69588
4.726	10.581	36.219	4.726	8.12372	38.67628	4.726	6.11583	40.68417	4.726	4.43484	41.51535	4.726	3.09328	36.74235
4.727	10.58642	36.21358	4.727	8.13018	38.66982	4.727	6.12315	40.67685	4.727	4.44269	41.54317	4.727	3.1004	36.78882
4.728	10.59184	36.20816	4.728	8.13664	38.66336	4.728	6.13045	40.66955	4.728	4.45054	41.571	4.728	3.10753	36.83531
4.729	10.59725	36.20275	4.729	8.14309	38.65691	4.729	6.13776	40.66224	4.729	4.45839	41.59884	4.729	3.11467	36.8818
4.73	10.60267	36.19733	4.73	8.14955	38.65045	4.73	6.14505	40.65495	4.73	4.46625	41.62669	4.73	3.12181	36.92831
4.731	10.60808	36.19192	4.731	8.15599	38.64401	4.731	6.15235	40.64765	4.731	4.47411	41.65455	4.731	3.12896	36.97482
4.732	10.61348	36.18652	4.732	8.16243	38.63757	4.732	6.15964	40.64036	4.732	4.48197	41.68242	4.732	3.13612	37.02134
4.733	10.61889	36.18111	4.733	8.16887	38.63113	4.733	6.16692	40.63308	4.733	4.48983	41.7103	4.733	3.14329	37.06786
4.734	10.62428	36.17572	4.734	8.17531	38.62469	4.734	6.1742	40.6258	4.734	4.4977	41.73818	4.734	3.15046	37.1144
4.735	10.62968	36.17032	4.735	8.18174	38.61826	4.735	6.18148	40.61852	4.735	4.50556	41.76608	4.735	3.15764	37.16094
4.736	10.63507	36.16493	4.736	8.18816	38.61184	4.736	6.18875	40.61125	4.736	4.51343	41.79398	4.736	3.16482	37.20749
4.737	10.64046	36.15954	4.737	8.19458	38.60542	4.737	6.19601	40.60399	4.737	4.52131	41.8219	4.737	3.17201	37.25405
4.738	10.64585	36.15415	4.738	8.201	38.599	4.738	6.20327	40.59673	4.738	4.52918	41.84982	4.738	3.17921	37.30062
4.739	10.65123	36.14877	4.739	8.20742	38.59258	4.739	6.21053	40.58947	4.739	4.53706	41.87775	4.739	3.18641	37.34719
4.74	10.65661	36.14339	4.74	8.21383	38.58617	4.74	6.21778	40.58222	4.74	4.54494	41.90569	4.74	3.19363	37.39378
4.741	10.66198	36.13802	4.741	8.22023	38.57977	4.741	6.22503	40.57497	4.741	4.55282	41.93364	4.741	3.20084	37.44037
4.742	10.66736	36.13264	4.742	8.22663	38.57337	4.742	6.23227	40.56773	4.742	4.56071	41.9616	4.742	3.20807	37.48697
4.743	10.67272	36.12728	4.743	8.23303	38.56697	4.743	6.23951	40.56049	4.743	4.5686	41.98957	4.743	3.2153	37.53358
4.744	10.67809	36.12191	4.744	8.23942	38.56058	4.744	6.24675	40.55325	4.744	4.57649	42.01755	4.744	3.22254	37.58019
4.745	10.68345	36.11655	4.745	8.24581	38.55419	4.745	6.25398	40.54602	4.745	4.58438	42.04553	4.745	3.22979	37.62682
4.746	10.68881	36.11119	4.746	8.2522	38.5478	4.746	6.2612	40.5388	4.746	4.59228	42.07353	4.746	3.23704	37.67345
4.747	10.69416	36.10584	4.747	8.25858	38.54142	4.747	6.26842	40.53158	4.747	4.60018	42.10153	4.747	3.2443	37.72009
4.748	10.69951	36.10049	4.748	8.26496	38.53504	4.748	6.27564	40.52436	4.748	4.60808	42.12955	4.748	3.25157	37.76674
4.749	10.70486	36.09514	4.749	8.27133	38.52867	4.749	6.28285	40.51715	4.749	4.61598	42.15757	4.749	3.25884	37.8134
4.75	10.71021	36.08979	4.75	8.2777	38.5223	4.75	6.29005	40.50995	4.75	4.62389	42.17611	4.75	3.26612	37.86007
4.751	10.71555	36.08445	4.751	8.28406	38.51594	4.751	6.29726	40.50274	4.751	4.63179	42.16821	4.751	3.2734	37.90674
4.752	10.72089	36.07911	4.752	8.29043	38.50957	4.752	6.30445	40.49555	4.752	4.63969	42.16031	4.752	3.2807	37.95342
4.753	10.72622	36.07378	4.753	8.29678	38.50322	4.753	6.31165	40.48835	4.753	4.64758	42.15242	4.753	3.288	38.00011
4.754	10.73155	36.06845	4.754	8.30314	38.49686	4.754	6.31883	40.48117	4.754	4.65547	42.14453	4.754	3.2953	38.04681
4.755	10.73688	36.06312	4.755	8.30949	38.49051	4.755	6.32602	40.47398	4.755	4.66336	42.13664	4.755	3.30262	38.09352
4.756	10.74221	36.05779	4.756	8.31583	38.48417	4.756	6.3332	40.4668	4.756	4.67123	42.12877	4.756	3.30994	38.14023
4.757	10.74753	36.05247	4.757	8.32217	38.47783	4.757	6.34037	40.45963	4.757	4.67911	42.12089	4.757	3.31726	38.18696

4.758	10.75284	36.04716	4.758	8.32851	38.47149	4.758	6.34754	40.45246	4.758	4.68698	42.11302	4.758	3.3246	38.23369
4.759	10.75816	36.04184	4.759	8.33484	38.46516	4.759	6.35471	40.44529	4.759	4.69484	42.10516	4.759	3.33194	38.28043
4.76	10.76347	36.03653	4.76	8.34117	38.45883	4.76	6.36187	40.43813	4.76	4.7027	42.0973	4.76	3.33928	38.32718
4.761	10.76878	36.03122	4.761	8.3475	38.4525	4.761	6.36903	40.43097	4.761	4.71055	42.08945	4.761	3.34664	38.37394
4.762	10.77408	36.02592	4.762	8.35382	38.44618	4.762	6.37618	40.42382	4.762	4.7184	42.0816	4.762	3.354	38.4207
4.763	10.77938	36.02062	4.763	8.36014	38.43986	4.763	6.38333	40.41667	4.763	4.72624	42.07376	4.763	3.36137	38.46748
4.764	10.78468	36.01532	4.764	8.36645	38.43355	4.764	6.39047	40.40953	4.764	4.73408	42.06592	4.764	3.36874	38.51426
4.765	10.78998	36.01002	4.765	8.37276	38.42724	4.765	6.39761	40.40239	4.765	4.74192	42.05808	4.765	3.37612	38.56105
4.766	10.79527	36.00473	4.766	8.37906	38.42094	4.766	6.40474	40.39526	4.766	4.74974	42.05026	4.766	3.38351	38.60785
4.767	10.80055	35.99945	4.767	8.38537	38.41463	4.767	6.41187	40.38813	4.767	4.75757	42.04243	4.767	3.3909	38.65466
4.768	10.80584	35.99416	4.768	8.39166	38.40834	4.768	6.419	40.381	4.768	4.76539	42.03461	4.768	3.3983	38.70147
4.769	10.81112	35.98888	4.769	8.39796	38.40204	4.769	6.42612	40.37388	4.769	4.7732	42.0268	4.769	3.40571	38.7483
4.77	10.8164	35.9836	4.77	8.40425	38.39575	4.77	6.43323	40.36677	4.77	4.78101	42.01899	4.77	3.41312	38.79513
4.771	10.82167	35.97833	4.771	8.41053	38.38947	4.771	6.44034	40.35966	4.771	4.78882	42.01118	4.771	3.42055	38.84197
4.772	10.82694	35.97306	4.772	8.41681	38.38319	4.772	6.44745	40.35255	4.772	4.79661	42.00339	4.772	3.42797	38.88882
4.773	10.83221	35.96779	4.773	8.42309	38.37691	4.773	6.45455	40.34545	4.773	4.80441	41.99559	4.773	3.43541	38.93568
4.774	10.83748	35.96252	4.774	8.42936	38.37064	4.774	6.46165	40.33835	4.774	4.8122	41.9878	4.774	3.44285	38.98254
4.775	10.84274	35.95726	4.775	8.43563	38.36437	4.775	6.46875	40.33125	4.775	4.81998	41.98002	4.775	3.4503	39.02942
4.776	10.848	35.952	4.776	8.4419	38.3581	4.776	6.47584	40.32416	4.776	4.82776	41.97224	4.776	3.45775	39.0763
4.777	10.85325	35.94675	4.777	8.44816	38.35184	4.777	6.48292	40.31708	4.777	4.83554	41.96446	4.777	3.46521	39.12319
4.778	10.8585	35.9415	4.778	8.45442	38.34558	4.778	6.49	40.31	4.778	4.84331	41.95669	4.778	3.47268	39.17009
4.779	10.86375	35.93625	4.779	8.46067	38.33933	4.779	6.49708	40.30292	4.779	4.85107	41.94893	4.779	3.48015	39.217
4.78	10.86899	35.93101	4.78	8.46692	38.33308	4.78	6.50415	40.29585	4.78	4.85883	41.94117	4.78	3.48764	39.26392
4.781	10.87424	35.92576	4.781	8.47317	38.32683	4.781	6.51122	40.28878	4.781	4.86659	41.93341	4.781	3.49512	39.31084
4.782	10.87947	35.92053	4.782	8.47941	38.32059	4.782	6.51828	40.28172	4.782	4.87434	41.92566	4.782	3.50262	39.35778
4.783	10.88471	35.91529	4.783	8.48565	38.31435	4.783	6.52534	40.27466	4.783	4.88208	41.91792	4.783	3.51012	39.40472
4.784	10.88994	35.91006	4.784	8.49188	38.30812	4.784	6.53239	40.26761	4.784	4.88983	41.91017	4.784	3.51763	39.45167
4.785	10.89517	35.90483	4.785	8.49812	38.30188	4.785	6.53944	40.26056	4.785	4.89756	41.90244	4.785	3.52514	39.49863
4.786	10.90039	35.89961	4.786	8.50434	38.29566	4.786	6.54649	40.25351	4.786	4.90529	41.89471	4.786	3.53266	39.5456
4.787	10.90562	35.89438	4.787	8.51056	38.28944	4.787	6.55353	40.24647	4.787	4.91302	41.88698	4.787	3.54019	39.59257
4.788	10.91083	35.88917	4.788	8.51678	38.28322	4.788	6.56056	40.23944	4.788	4.92074	41.87926	4.788	3.54773	39.63956
4.789	10.91605	35.88395	4.789	8.523	38.277	4.789	6.56759	40.23241	4.789	4.92846	41.87154	4.789	3.55527	39.68655
4.79	10.92126	35.87874	4.79	8.52921	38.27079	4.79	6.57462	40.22538	4.79	4.93617	41.86383	4.79	3.56282	39.73356
4.791	10.92647	35.87353	4.791	8.53542	38.26458	4.791	6.58164	40.21836	4.791	4.94388	41.85612	4.791	3.57037	39.78057
4.792	10.93168	35.86832	4.792	8.54162	38.25838	4.792	6.58866	40.21134	4.792	4.95158	41.84842	4.792	3.57793	39.82759
4.793	10.93688	35.86312	4.793	8.54782	38.25218	4.793	6.59568	40.20432	4.793	4.95927	41.84073	4.793	3.5855	39.87461
4.794	10.94208	35.85792	4.794	8.55401	38.24599	4.794	6.60269	40.19731	4.794	4.96697	41.83303	4.794	3.59307	39.92165
4.795	10.94727	35.85273	4.795	8.56021	38.23979	4.795	6.60969	40.19031	4.795	4.97465	41.82535	4.795	3.60065	39.9687
4.796	10.95246	35.84754	4.796	8.56639	38.23361	4.796	6.61669	40.18331	4.796	4.98234	41.81766	4.796	3.60824	40.01575
4.797	10.95765	35.84235	4.797	8.57258	38.22742	4.797	6.62369	40.17631	4.797	4.99002	41.80998	4.797	3.61584	40.06281
4.798	10.96284	35.83716	4.798	8.57876	38.22124	4.798	6.63068	40.16932	4.798	4.99769	41.80231	4.798	3.62344	40.10988
4.799	10.96802	35.83198	4.799	8.58493	38.21507	4.799	6.63767	40.16233	4.799	5.00536	41.79464	4.799	3.63105	40.15696
4.8	10.9732	35.8268	4.8	8.59111	38.20889	4.8	6.64465	40.15535	4.8	5.01302	41.78698	4.8	3.63866	40.20405
4.801	10.97838	35.82162	4.801	8.59728	38.20272	4.801	6.65163	40.14837	4.801	5.02068	41.77932	4.801	3.64628	40.25115
4.802	10.98355	35.81645	4.802	8.60344	38.19656	4.802	6.65861	40.14139	4.802	5.02833	41.77167	4.802	3.65391	40.29825
4.803	10.98872	35.81128	4.803	8.6096	38.1904	4.803	6.66558	40.13442	4.803	5.03598	41.76402	4.803	3.66154	40.34537
4.804	10.99389	35.80611	4.804	8.61576	38.18424	4.804	6.67255	40.12745	4.804	5.04363	41.75637	4.804	3.66919	40.39249
4.805	10.99905	35.80095	4.805	8.62191	38.17809	4.805	6.67951	40.12049	4.805	5.05127	41.74873	4.805	3.67683	40.43962
4.806	11.00421	35.79579	4.806	8.62806	38.17194	4.806	6.68646	40.11354	4.806	5.0589	41.7411	4.806	3.68449	40.48676
4.807	11.00937	35.79063	4.807	8.6342	38.1658	4.807	6.69342	40.10658	4.807	5.06653	41.73347	4.807	3.69215	40.53391
4.808	11.01452	35.78548	4.808	8.64035	38.15965	4.808	6.70037	40.09963	4.808	5.07416	41.72584	4.808	3.69982	40.58107
4.809	11.01967	35.78033	4.809	8.64648	38.15352	4.809	6.70731	40.09269	4.809	5.08178	41.71822	4.809	3.70749	40.61412
4.81	11.02482	35.77518	4.81	8.65262	38.14738	4.81	6.71425	40.08575	4.81	5.08939	41.71061	4.81	3.71516	40.64226
4.811	11.02996	35.77004	4.811	8.65875	38.14125	4.811	6.72119	40.07881	4.811	5.09701	41.70299	4.811	3.72284	40.67042
4.812	11.0351	35.7649	4.812	8.66487	38.13513	4.812	6.72812	40.07188	4.812	5.10461	41.69539	4.812	3.73052	40.69858
4.813	11.04024	35.75976	4.813	8.671	38.129	4.813	6.73505	40.06495	4.813	5.11221	41.68779	4.813	3.73821	40.72675
4.814	11.04538	35.75462	4.814	8.67712	38.12288	4.814	6.74197	40.05803	4.814	5.11981	41.68019	4.814	3.74589	40.75494
4.815	11.05051	35.74949	4.815	8.68323	38.11677	4.815	6.74889	40.05111	4.815	5.1274	41.6726	4.815	3.75358	40.78313
4.816	11.05563	35.74437	4.816	8.68934	38.11066	4.816	6.7558	40.0442	4.816	5.13499	41.66501	4.816	3.76128	40.81133
4.817	11.06076	35.73924	4.817	8.69545	38.10455	4.817	6.76271	40.03729	4.817	5.14257	41.65743	4.817	3.76897	40.83955
4.818	11.06588	35.73412	4.818	8.70155	38.09845	4.818	6.76962	40.03038	4.818	5.15015	41.64985	4.818	3.77667	40.86777
4.819	11.071	35.729	4.819	8.70765	38.09235	4.819	6.77652	40.02348	4.819	5.15772	41.64228	4.819	3.78437	40.896
4.82	11.07611	35.72389	4.82	8.71375	38.08625	4.82	6.78341	40.01659	4.82	5.16529	41.63471	4.82	3.79207	40.92425
4.821	11.08123	35.71877	4.821	8.71984	38.08016	4.821	6.79031	40.00969	4.821	5.17286	41.62714	4.821	3.79977	40.9525
4.822	11.08634	35.71366	4.822	8.72593	38.07407	4.822	6.7972	40.0028	4.822	5.18042	41.61958	4.822	3.80748	40.98076
4.823	11.09144	35.70856	4.823	8.73201	38.06799	4.823	6.80408	39.99592	4.823	5.18797	41.61203	4.823	3.81519	41.00903
4.824	11.09654	35.70346	4.824	8.73809	38.06191	4.824	6.81096	39.98904	4.824	5.19552	41.60448	4.824	3.82291	41.03731
4.825	11.10164	35.69836	4.825	8.74417	38.05583	4.825	6.81784	39.98216	4.825	5.20307	41.59693	4.825	3.83062	41.0656
4.826	11.10674	35.69326	4.826	8.75024	38.04976	4.826	6.82471	39.97529	4.826	5.21061	41.58939	4.826	3.83834	41.0939
4.827	11.11183	35.68817	4.827	8.75631	38.04369	4.827	6.83157	39.96843	4.827	5.21814	41.58186	4.827	3.84606	41.12221
4.828	11.11692	35.68308	4.828	8.76238	38.03762	4.828	6.83844	39.96156	4.828	5.22567	41.57433	4.828	3.85378	41.15054
4.829	11.12201	35.67799	4.829	8.76844	38.03156	4.829	6.84529	39.95471	4.829	5.2332	41.5668	4.829	3.86151	41.17887
4.83	11.12709	35.67291	4.83	8.7745	38.0255	4.83	6.85215	39.94785	4.83	5.24072	41.55928	4.83	3.86924	41.20721
4.831	11.13217	35.66783	4.831	8.78055	38.01945	4.831	6.859	39.941	4.831	5.24824	41.55176	4.831	3.87697	41.23556
4.832	11.13725	35.66275	4.832	8.7866	38.0134	4.832	6.86584	39.93416	4.832	5.25575	41.54425	4.832	3.88471	41.26391
4.833	11.14232	35.65768	4.833	8.79265	38.00735	4.833	6.87269	39.92731	4.833	5.26326	41.53674	4.833	3.89244	41.29228
4.834	11.14739	35.65261	4.834	8.79869	38.00131	4.834	6.87952	39.92048	4.834	5.27076	41.52924	4.834	3.90018	41.32066
4.835	11.15246	35.64754	4.835	8.80473	37.99527	4.835	6.88636	39.91364	4.835	5.27826	41.52174	4.835	3.90793	41.34905
4.836	11.15753	35.64247	4.836	8.81077	37.98923	4.836	6.89319	39.90681	4.836	5.28575	41.51425	4.836	3.91567	41.37745
4.837	11.16259	35.63741	4.837	8.8168	37.9832	4.837	6.90001	39.89999	4.837	5.29324	41.50676	4.837	3.92342	41.40586
4.838	11.16765	35.63235	4.838	8.82283	37.97717	4.838	6.90683	39.89317	4.838	5.30073	41.49927	4.838	3.93117	41.43428
4.839	11.1727	35.6273	4.839	8.82885	37.97115	4.839	6.91365	39.88635	4.839	5.30821	41.49179	4.839	3.93892	41.46271
4.84	11.17775	35.62225	4.84	8.83487	37.96513	4.84	6.92046	39.87954	4.84	5.31568	41.48432	4.84	3.94668	41.49114
4.841	11.1828	35.6172	4.841	8.84089	37.95911	4.841	6.92727	39.87273	4.841	5.32315	41.47685	4.841	3.95444	41.51959
4.842	11.18785	35.61215	4.842	8.8469	37.9531	4.842	6.93407	39.86593	4.842	5.33062	41.46938	4.842	3.9622	41.54805
4.843	11.19289	35.60711	4.843	8.85291	37.94709	4.843	6.94087	39.85913	4.843	5.33808	41.46192	4.843	3.96996	41.57652
4.844	11.19793	35.60207	4.844	8.85891	37.94109	4.844	6.94766	39.85234	4.844	5.34554	41.45446	4.844	3.97773	41.60499
4.845	11.20297	35.59703	4.845	8.86492	37.93508	4.845	6.95445	39.84555	4.845	5.35299	41.44701	4.845	3.9855	41.63348
4.846	11.208	35.592	4.846	8.87092	37.92908	4.846	6.96124	39.83876	4.846	5.36044	41.43956	4.846	3.99327	41.66198
4.847	11.21303	35.58697	4.847	8.87691	37.92309	4.847	6.96802	39.83198	4.847	5.36788	41.43212	4.847	4.00104	41.69048
4.848	11.21806	35.58194	4.848	8.8829	37.9171	4.848	6.9748	39.8252	4.848	5.37532	41.42468	4.848	4.00882	41.719
4.849	11.22308	35.57692	4.849	8.88889	37.91111	4.849	6.98157	39.81843	4.849	5.38275	41.41725	4.849	4.0166	41.74753
4.85	11.2281	35.5719	4.85	8.89487	37.90513	4.85	6.98834	39.81166	4.85	5.39018	41.40982	4.85	4.02438	41.77606
4.851	11.23312	35.56688	4.851	8.90085	37.89915	4.851	6.99511	39.80489	4.851	5.3976	41.4024	4.851	4.03217	41.80461
4.852	11.23814	35.56186	4.852	8.90683	37.89317	4.852	7.00187	39.79813	4.852	5.40502	41.39498	4.852	4.03996	41.83316
4.853	11.24315	35.55685	4.853	8.9128	37.8872	4.853	7.00863	39.79137	4.853	5.41244	41.38756	4.853	4.04775	41.86173
4.854	11.24816	35.55184	4.854	8.91877	37.88123	4.854	7.01538	39.78462	4.854	5.41985	41.38015	4.854	4.05554	41.8903
4.855	11.25316	35.54684	4.855	8.92473	37.87527	4.855	7.02213	39.77787	4.855	5.42726	41.37274	4.855	4.06334	41.91889
4.856	11.25816	35.54184	4.856	8.93069	37.86931	4.856	7.02888	39.77112	4.856	5.43466	41.36534	4.856	4.07113	41.94748
4.857	11.26316	35.53684	4.857	8.93665	37.86335	4.857	7.03562	39.76438	4.857	5.44205	41.35795	4.857	4.07893	41.97609
4.858	11.26816	35.53184	4.858	8.9426	37.8574	4.858	7.04235	39.75765	4.858	5.44945	41.35055	4.858	4.08674	42.0047
4.859	11.27315	35.52685	4.859	8.94855	37.85145	4.859	7.04908	39.75092	4.859	5.45683	41.34317	4.859	4.09455	42.03333
4.86	11.27814	35.52186	4.86	8.9545	37.8455	4.86	7.05581	39.74419	4.86	5.46422	41.33578	4.86	4.10235	42.06196
4.861	11.28313	35.51687	4.861	8.96044	37.83956	4.861	7.06254	39.73746	4.861	5.4716	41.3284	4.861	4.11017	42.0906
4.862	11.28811	35.51189	4.862	8.96638	37.83362	4.862	7.06926	39.73074	4.862	5.47897	41.32103	4.862	4.11798	42.11926
4.863	11.29309	35.50691	4.863	8.97232	37.82768	4.863	7.07597	39.72403	4.863	5.48634	41.31366	4.863	4.1258	42.14792
4.864	11.29807	35.50193	4.864	8.97825	37.82175	4.864	7.08268	39.71732	4.864	5.49371	41.30629	4.864	4.13362	42.17659
4.865	11.30305	35.49695	4.865	8.98418	37.81582	4.865	7.08939	39.71061	4.865	5.50107	41.29893	4.865	4.14144	42.20528
4.866	11.30802	35.49198	4.866	8.9901	37.8099	4.866	7.09609	39.70391	4.866	5.50842	41.29158	4.866	4.14927	42.23397
4.867	11.31299	35.48701	4.867	8.99602	37.80398	4.867	7.10279	39.69721	4.867	5.51577	41.28423	4.867	4.15709	42.26267
4.868	11.31795	35.48205	4.868	9.00194	37.79806	4.868	7.10949	39.69051	4.868	5.52312	41.27688	4.868	4.16493	42.29139
4.869	11.32291	35.47709	4.869	9.00785	37.79215	4.869	7.11618	39.68382	4.869	5.53046	41.26954	4.869	4.17276	42.32011
4.87	11.32787	35.47213	4.87	9.01376	37.78624	4.87	7.12287	39.67713	4.87	5.5378	41.2622	4.87	4.18059	42.34884
4.871	11.33283	35.46717	4.871	9.01967	37.78033	4.871	7.12955	39.67045	4.871	5.54513	41.25487	4.871	4.18843	42.37758
4.872	11.33778	35.46222	4.872	9.02557	37.77443	4.872	7.13623	39.66377	4.872	5.55246	41.24754	4.872	4.19628	42.40633
4.873	11.34273	35.45727	4.873	9.03147	37.76853	4.873	7.1429	39.6571	4.873	5.55979	41.24021	4.873	4.20412	42.4351
4.874	11.34768	35.45232	4.874	9.03737	37.76263	4.874	7.14957	39.65043	4.874	5.56711	41.23289	4.874	4.21197	42.46387
4.875	11.35262	35.44738	4.875	9.04326	37.75674	4.875	7.15624	39.64376	4.875	5.57442	41.22558	4.875	4.21982	42.49265
4.876	11.35756	35.44244	4.876	9.04915	37.75085	4.876	7.1629	39.6371	4.876	5.58173	41.21827	4.876	4.22767	42.52144
4.877	11.3625	35.4375	4.877	9.05503	37.74497	4.877	7.16956	39.63044	4.877	5.58904	41.21096	4.877	4.23552	42.55024
4.878	11.36744	35.43256	4.878	9.06091	37.73909	4.878	7.17621	39.62379	4.878	5.59634	41.20366	4.878	4.24338	42.55662
4.879	11.37237	35.42763	4.879	9.06679	37.73321	4.879	7.18286	39.61714	4.879	5.60364	41.19636	4.879	4.25123	42.54877
4.88	11.3773	35.4227	4.88	9.07266	37.72734	4.88	7.18951	39.61049	4.88	5.61093	41.18907	4.88	4.25908	42.54092
4.881	11.38222	35.41778	4.881	9.07853	37.72147	4.881	7.19615	39.60385	4.881	5.61822	41.18178	4.881	4.26692	42.53308
4.882	11.38715	35.41285	4.882	9.0844	37.7156	4.882	7.20279	39.59721	4.882	5.6255	41.1745	4.882	4.27476	42.52524
4.883	11.39207	35.40793	4.883	9.09026	37.70974	4.883	7.20942	39.59058	4.883	5.63278	41.16722	4.883	4.28259	42.51741
4.884	11.39698	35.40302	4.884	9.09612	37.70388	4.884	7.21605	39.58395	4.884	5.64006	41.15994	4.884	4.29042	42.50958
4.885	11.4019	35.3981	4.885	9.10198	37.69802	4.885	7.22267	39.57733	4.885	5.64733	41.15267	4.885	4.29824	42.50176
4.886	11.40681	35.39319	4.886	9.10783	37.69217	4.886	7.2293	39.5707	4.886	5.65459	41.14541	4.886	4.30606	42.49394
4.887	11.41172	35.38828	4.887	9.11368	37.68632	4.887	7.23591	39.56409	4.887	5.66186	41.13814	4.887	4.31387	42.48613
4.888	11.41662	35.38338	4.888	9.11952	37.68048	4.888	7.24253	39.55747	4.888	5.66911	41.13089	4.888	4.32168	42.47832
4.889	11.42152	35.37848	4.889	9.12537	37.67463	4.889	7.24913	39.55087	4.889	5.67637	41.12363	4.889	4.32948	42.47052
4.89	11.42642	35.37358	4.89	9.1312	37.6688	4.89	7.25574	39.54426	4.89	5.68361	41.11639	4.89	4.33728	42.46272
4.891	11.43132	35.36868	4.891	9.13704	37.66296	4.891	7.26234	39.53766	4.891	5.69086	41.10914	4.891	4.34508	42.45492
4.892	11.43621	35.36379	4.892	9.14287	37.65713	4.892	7.26894	39.53106	4.892	5.6981	41.1019	4.892	4.35287	42.44713
4.893	11.4411	35.3589	4.893	9.14869	37.65131	4.893	7.27553	39.52447	4.893	5.70533	41.09467	4.893	4.36065	42.43935
4.894	11.44598	35.35402	4.894	9.15452	37.64548	4.894	7.28212	39.51788	4.894	5.71256	41.08744	4.894	4.36843	42.43157
4.895	11.45087	35.34913	4.895	9.16034	37.63966	4.895	7.2887	39.5113	4.895	5.71979	41.08021	4.895	4.37621	42.42379
4.896	11.45575	35.34425	4.896	9.16615	37.63385	4.896	7.29528	39.50472	4.896	5.72701	41.07299	4.896	4.38398	42.41602
4.897	11.46063	35.33937	4.897	9.17197	37.62803	4.897	7.30186	39.49814	4.897	5.73423	41.06577	4.897	4.39174	42.40826
4.898	11.4655	35.3345	4.898	9.17778	37.62222	4.898	7.30843	39.49157	4.898	5.74144	41.05856	4.898	4.3995	42.4005
4.899	11.47037	35.32963	4.899	9.18358	37.61642	4.899	7.315	39.485	4.899	5.74865	41.05135	4.899	4.40726	42.39274
4.9	11.47524	35.32476	4.9	9.18938	37.61062	4.9	7.32157	39.47843	4.9	5.75585	41.04415	4.9	4.41501	42.38499
4.901	11.48011	35.31989	4.901	9.19518	37.60482	4.901	7.32813	39.47187	4.901	5.76305	41.03695	4.901	4.42276	42.37724
4.902	11.48497	35.31503	4.902	9.20098	37.59902	4.902	7.33468	39.46532	4.902	5.77025	41.02975	4.902	4.4305	42.3695
4.903	11.48983	35.31017	4.903	9.20677	37.59323	4.903	7.34124	39.45876	4.903	5.77744	41.02256	4.903	4.43824	42.36176
4.904	11.49469	35.30531	4.904	9.21256	37.58744	4.904	7.34779	39.45221	4.904	5.78462	41.01538	4.904	4.44597	42.35403
4.905	11.49954	35.30046	4.905	9.21834	37.58166	4.905	7.35433	39.44567	4.905	5.79181	41.00819	4.905	4.4537	42.3463
4.906	11.50439	35.29561	4.906	9.22412	37.57588	4.906	7.36087	39.43913	4.906	5.79898	41.00102	4.906	4.46142	42.33858
4.907	11.50924	35.29076	4.907	9.2299	37.5701	4.907	7.36741	39.43259	4.907	5.80616	40.99384	4.907	4.46914	42.33086
4.908	11.51408	35.28592	4.908	9.23567	37.56433	4.908	7.37394	39.42606	4.908	5.81332	40.98668	4.908	4.47685	42.32315
4.909	11.51893	35.28107	4.909	9.24144	37.55856	4.909	7.38047	39.41953	4.909	5.82049	40.97951	4.909	4.48456	42.31544
4.91	11.52377	35.27623	4.91	9.24721	37.55279	4.91	7.38699	39.41301	4.91	5.82765	40.97235	4.91	4.49226	42.30774
4.911	11.5286	35.2714	4.911	9.25297	37.54703	4.911	7.39351	39.40649	4.911	5.8348	40.9652	4.911	4.49996	42.30004
4.912	11.53344	35.26656	4.912	9.25873	37.54127	4.912	7.40003	39.39997	4.912	5.84196	40.95804	4.912	4.50766	42.29234
4.913	11.53827	35.26173	4.913	9.26449	37.53551	4.913	7.40654	39.39346	4.913	5.8491	40.9509	4.913	4.51535	42.28465
4.914	11.54309	35.25691	4.914	9.27024	37.52976	4.914	7.41305	39.38695	4.914	5.85625	40.94375	4.914	4.52303	42.27697
4.915	11.54792	35.25208	4.915	9.27599	37.52401	4.915	7.41956	39.38044	4.915	5.86338	40.93662	4.915	4.53071	42.26929
4.916	11.55274	35.24726	4.916	9.28173	37.51827	4.916	7.42606	39.37394	4.916	5.87052	40.92948	4.916	4.53839	42.26161
4.917	11.55756	35.24244	4.917	9.28748	37.51252	4.917	7.43255	39.36745	4.917	5.87765	40.92235	4.917	4.54606	42.25394

4.918	11.56237	35.23763	4.918	9.29322	37.50678	4.918	7.43905	39.36095	4.918	5.88477	40.91523	4.918	4.55373	42.24627
4.919	11.56718	35.23282	4.919	9.29895	37.50105	4.919	7.44554	39.35446	4.919	5.89189	40.90811	4.919	4.56139	42.23861
4.92	11.57199	35.22801	4.92	9.30468	37.49532	4.92	7.45202	39.34798	4.92	5.89901	40.90099	4.92	4.56905	42.23095
4.921	11.5768	35.2232	4.921	9.31041	37.48959	4.921	7.4585	39.3415	4.921	5.90612	40.89388	4.921	4.5767	42.2233
4.922	11.5816	35.2184	4.922	9.31613	37.48387	4.922	7.46498	39.33502	4.922	5.91323	40.88677	4.922	4.58435	42.21565
4.923	11.58641	35.21359	4.923	9.32186	37.47814	4.923	7.47145	39.32855	4.923	5.92033	40.87967	4.923	4.59199	42.20801
4.924	11.5912	35.2088	4.924	9.32757	37.47243	4.924	7.47792	39.32208	4.924	5.92743	40.87257	4.924	4.59963	42.20037
4.925	11.596	35.204	4.925	9.33329	37.46671	4.925	7.48439	39.31561	4.925	5.93453	40.86547	4.925	4.60726	42.19274
4.926	11.60079	35.19921	4.926	9.339	37.461	4.926	7.49085	39.30915	4.926	5.94162	40.85838	4.926	4.61489	42.18511
4.927	11.60558	35.19442	4.927	9.34471	37.45529	4.927	7.4973	39.3027	4.927	5.9487	40.8513	4.927	4.62252	42.17748
4.928	11.61037	35.18963	4.928	9.35041	37.44959	4.928	7.50376	39.29624	4.928	5.95578	40.84422	4.928	4.63014	42.16986
4.929	11.61515	35.18485	4.929	9.35611	37.44389	4.929	7.51021	39.28979	4.929	5.96286	40.83714	4.929	4.63775	42.16225
4.93	11.61993	35.18007	4.93	9.36181	37.43819	4.93	7.51665	39.28335	4.93	5.96993	40.83007	4.93	4.64536	42.15464
4.931	11.62471	35.17529	4.931	9.3675	37.4325	4.931	7.52309	39.27691	4.931	5.977	40.823	4.931	4.65297	42.14703
4.932	11.62948	35.17052	4.932	9.37319	37.42681	4.932	7.52953	39.27047	4.932	5.98407	40.81593	4.932	4.66057	42.13943
4.933	11.63425	35.16575	4.933	9.37888	37.42112	4.933	7.53597	39.26403	4.933	5.99113	40.80887	4.933	4.66817	42.13183
4.934	11.63902	35.16098	4.934	9.38456	37.41544	4.934	7.5424	39.2576	4.934	5.99818	40.80182	4.934	4.67576	42.12424
4.935	11.64379	35.15621	4.935	9.39024	37.40976	4.935	7.54882	39.25118	4.935	6.00524	40.79476	4.935	4.68335	42.11665
4.936	11.64855	35.15145	4.936	9.39591	37.40409	4.936	7.55524	39.24476	4.936	6.01228	40.78772	4.936	4.69093	42.10907
4.937	11.65331	35.14669	4.937	9.40159	37.39841	4.937	7.56166	39.23834	4.937	6.01933	40.78067	4.937	4.69851	42.10149
4.938	11.65807	35.14193	4.938	9.40726	37.39274	4.938	7.56808	39.23192	4.938	6.02637	40.77363	4.938	4.70608	42.09392
4.939	11.66282	35.13718	4.939	9.41292	37.38708	4.939	7.57449	39.22551	4.939	6.0334	40.7666	4.939	4.71365	42.08635
4.94	11.66757	35.13243	4.94	9.41858	37.38142	4.94	7.58089	39.21911	4.94	6.04043	40.75957	4.94	4.72122	42.07878
4.941	11.67232	35.12768	4.941	9.42424	37.37576	4.941	7.5873	39.2127	4.941	6.04746	40.75254	4.941	4.72878	42.07122
4.942	11.67707	35.12293	4.942	9.4299	37.3701	4.942	7.59369	39.20631	4.942	6.05448	40.74552	4.942	4.73633	42.06367
4.943	11.68181	35.11819	4.943	9.43555	37.36445	4.943	7.60009	39.19991	4.943	6.0615	40.7385	4.943	4.74388	42.05612
4.944	11.68655	35.11345	4.944	9.4412	37.3588	4.944	7.60648	39.19352	4.944	6.06851	40.73149	4.944	4.75143	42.04857
4.945	11.69129	35.10871	4.945	9.44684	37.35316	4.945	7.61287	39.18713	4.945	6.07552	40.72448	4.945	4.75897	42.04103
4.946	11.69602	35.10398	4.946	9.45248	37.34752	4.946	7.61925	39.18075	4.946	6.08252	40.71748	4.946	4.76651	42.03349
4.947	11.70075	35.09925	4.947	9.45812	37.34188	4.947	7.62563	39.17437	4.947	6.08952	40.71048	4.947	4.77404	42.02596
4.948	11.70548	35.09452	4.948	9.46376	37.33624	4.948	7.632	39.168	4.948	6.09652	40.70348	4.948	4.78157	42.01843
4.949	11.71021	35.08979	4.949	9.46939	37.33061	4.949	7.63838	39.16162	4.949	6.10351	40.69649	4.949	4.78909	42.01091
4.95	11.71493	35.08507	4.95	9.47502	37.32498	4.95	7.64474	39.15526	4.95	6.1105	40.6895	4.95	4.79661	42.00339
4.951	11.71965	35.08035	4.951	9.48064	37.31936	4.951	7.65111	39.14889	4.951	6.11748	40.68252	4.951	4.80412	41.99588
4.952	11.72437	35.07563	4.952	9.48626	37.31374	4.952	7.65747	39.14253	4.952	6.12446	40.67554	4.952	4.81163	41.98837
4.953	11.72908	35.07092	4.953	9.49188	37.30812	4.953	7.66382	39.13618	4.953	6.13144	40.66856	4.953	4.81914	41.98086
4.954	11.73379	35.06621	4.954	9.49749	37.30251	4.954	7.67018	39.12982	4.954	6.13841	40.66159	4.954	4.82664	41.97336
4.955	11.7385	35.0615	4.955	9.50311	37.29689	4.955	7.67652	39.12348	4.955	6.14537	40.65463	4.955	4.83413	41.96587
4.956	11.74321	35.05679	4.956	9.50871	37.29129	4.956	7.68287	39.11713	4.956	6.15234	40.64766	4.956	4.84163	41.95837
4.957	11.74791	35.05209	4.957	9.51432	37.28568	4.957	7.68921	39.11079	4.957	6.1593	40.6407	4.957	4.84911	41.95089
4.958	11.75261	35.04739	4.958	9.51992	37.28008	4.958	7.69555	39.10445	4.958	6.16625	40.63375	4.958	4.8566	41.9434
4.959	11.75731	35.04269	4.959	9.52552	37.27448	4.959	7.70188	39.09812	4.959	6.1732	40.6268	4.959	4.86407	41.93593
4.96	11.762	35.038	4.96	9.53111	37.26889	4.96	7.70821	39.09179	4.96	6.18014	40.61986	4.96	4.87155	41.92845
4.961	11.76669	35.03331	4.961	9.5367	37.2633	4.961	7.71453	39.08547	4.961	6.18709	40.61291	4.961	4.87902	41.92098
4.962	11.77138	35.02862	4.962	9.54229	37.25771	4.962	7.72086	39.07914	4.962	6.19402	40.60598	4.962	4.88648	41.91352
4.963	11.77606	35.02394	4.963	9.54787	37.25213	4.963	7.72717	39.07283	4.963	6.20096	40.59904	4.963	4.89394	41.90606
4.964	11.78075	35.01925	4.964	9.55345	37.24655	4.964	7.73349	39.06651	4.964	6.20788	40.59212	4.964	4.9014	41.8986
4.965	11.78543	35.01457	4.965	9.55903	37.24097	4.965	7.7398	39.0602	4.965	6.21481	40.58519	4.965	4.90885	41.89115
4.966	11.7901	35.0099	4.966	9.5646	37.2354	4.966	7.7461	39.0539	4.966	6.22173	40.57827	4.966	4.91629	41.88371
4.967	11.79478	35.00522	4.967	9.57017	37.22983	4.967	7.75241	39.04759	4.967	6.22865	40.57135	4.967	4.92373	41.87627
4.968	11.79945	35.00055	4.968	9.57574	37.22426	4.968	7.75871	39.04129	4.968	6.23556	40.56444	4.968	4.93117	41.86883
4.969	11.80412	34.99588	4.969	9.5813	37.2187	4.969	7.765	39.035	4.969	6.24247	40.55753	4.969	4.93861	41.86139
4.97	11.80879	34.99121	4.97	9.58686	37.21314	4.97	7.77129	39.02871	4.97	6.24937	40.55063	4.97	4.94603	41.85397
4.971	11.81345	34.98655	4.971	9.59242	37.20758	4.971	7.77758	39.02242	4.971	6.25627	40.54373	4.971	4.95346	41.84654
4.972	11.81811	34.98189	4.972	9.59797	37.20203	4.972	7.78386	39.01614	4.972	6.26316	40.53684	4.972	4.96088	41.83912
4.973	11.82277	34.97723	4.973	9.60352	37.19648	4.973	7.79014	39.00986	4.973	6.27006	40.52994	4.973	4.96829	41.83171
4.974	11.82742	34.97258	4.974	9.60907	37.19093	4.974	7.79642	39.00358	4.974	6.27694	40.52306	4.974	4.9757	41.8243
4.975	11.83207	34.96793	4.975	9.61461	37.18539	4.975	7.80269	38.99731	4.975	6.28383	40.51617	4.975	4.98311	41.81689
4.976	11.83672	34.96328	4.976	9.62015	37.17985	4.976	7.80896	38.99104	4.976	6.29071	40.50929	4.976	4.99051	41.80949
4.977	11.84137	34.95863	4.977	9.62569	37.17431	4.977	7.81522	38.98478	4.977	6.29758	40.50242	4.977	4.99791	41.80209
4.978	11.84601	34.95399	4.978	9.63123	37.16877	4.978	7.82148	38.97852	4.978	6.30445	40.49555	4.978	5.0053	41.7947
4.979	11.85065	34.94935	4.979	9.63676	37.16324	4.979	7.82774	38.97226	4.979	6.31132	40.48868	4.979	5.01269	41.78731
4.98	11.85529	34.94471	4.98	9.64228	37.15772	4.98	7.83399	38.96601	4.98	6.31818	40.48182	4.98	5.02007	41.77993
4.981	11.85992	34.94008	4.981	9.64781	37.15219	4.981	7.84024	38.95976	4.981	6.32504	40.47496	4.981	5.02745	41.77255
4.982	11.86456	34.93544	4.982	9.65333	37.14667	4.982	7.84649	38.95351	4.982	6.33189	40.46811	4.982	5.03483	41.76517
4.983	11.86919	34.93081	4.983	9.65884	37.14116	4.983	7.85273	38.94727	4.983	6.33874	40.46126	4.983	5.0422	41.7578
4.984	11.87381	34.92619	4.984	9.66436	37.13564	4.984	7.85897	38.94103	4.984	6.34559	40.45441	4.984	5.04956	41.75044
4.985	11.87844	34.92156	4.985	9.66987	37.13013	4.985	7.8652	38.9348	4.985	6.35243	40.44757	4.985	5.05692	41.74308
4.986	11.88306	34.91694	4.986	9.67537	37.12463	4.986	7.87144	38.92856	4.986	6.35927	40.44073	4.986	5.06428	41.73572
4.987	11.88768	34.91232	4.987	9.68088	37.11912	4.987	7.87766	38.92234	4.987	6.3661	40.4339	4.987	5.07163	41.72837
4.988	11.89229	34.90771	4.988	9.68638	37.11362	4.988	7.88389	38.91611	4.988	6.37293	40.42707	4.988	5.07898	41.72102
4.989	11.8969	34.9031	4.989	9.69187	37.10813	4.989	7.89011	38.90989	4.989	6.37975	40.42025	4.989	5.08633	41.71367
4.99	11.90151	34.89849	4.99	9.69737	37.10263	4.99	7.89632	38.90368	4.99	6.38657	40.41343	4.99	5.09366	41.70634
4.991	11.90612	34.89388	4.991	9.70286	37.09714	4.991	7.90253	38.89747	4.991	6.39339	40.40661	4.991	5.101	41.699
4.992	11.91073	34.88927	4.992	9.70835	37.09165	4.992	7.90874	38.89126	4.992	6.4002	40.3998	4.992	5.10833	41.69167
4.993	11.91533	34.88467	4.993	9.71383	37.08617	4.993	7.91495	38.88505	4.993	6.40701	40.39299	4.993	5.11566	41.68434
4.994	11.91993	34.88007	4.994	9.71931	37.08069	4.994	7.92115	38.87885	4.994	6.41382	40.38618	4.994	5.12298	41.67702
4.995	11.92452	34.87548	4.995	9.72479	37.07521	4.995	7.92734	38.87266	4.995	6.42062	40.37938	4.995	5.1303	41.6697
4.996	11.92912	34.87088	4.996	9.73026	37.06974	4.996	7.93354	38.86646	4.996	6.42741	40.37259	4.996	5.13761	41.66239
4.997	11.93371	34.86629	4.997	9.73573	37.06427	4.997	7.93973	38.86027	4.997	6.43421	40.36579	4.997	5.14492	41.65508
4.998	11.9383	34.8617	4.998	9.7412	37.0588	4.998	7.94591	38.85409	4.998	6.441	40.359	4.998	5.15222	41.64778
4.999	11.94288	34.85712	4.999	9.74666	37.05334	4.999	7.95209	38.84791	4.999	6.44778	40.35222	4.999	5.15952	41.64048
5	11.94746	34.85254	5	9.75212	37.04788	5	7.95827	38.84173	5	6.45456	40.34544	5	5.16682	41.63318
5.001	11.95204	34.84796	5.001	9.75758	37.04242	5.001	7.96445	38.83555	5.001	6.46134	40.33866	5.001	5.17411	41.62589
5.002	11.95662	34.84338	5.002	9.76304	37.03696	5.002	7.97062	38.82938	5.002	6.46811	40.33189	5.002	5.18139	41.61861
5.003	11.96119	34.83881	5.003	9.76849	37.03151	5.003	7.97679	38.82321	5.003	6.47488	40.32512	5.003	5.18868	41.61132
5.004	11.96576	34.83424	5.004	9.77393	37.02607	5.004	7.98295	38.81705	5.004	6.48164	40.31836	5.004	5.19595	41.60405
5.005	11.97033	34.82967	5.005	9.77938	37.02062	5.005	7.98911	38.81089	5.005	6.4884	40.3116	5.005	5.20323	41.59677
5.006	11.9749	34.8251	5.006	9.78482	37.01518	5.006	7.99527	38.80473	5.006	6.49516	40.30484	5.006	5.2105	41.5895
5.007	11.97946	34.82054	5.007	9.79026	37.00974	5.007	8.00142	38.79858	5.007	6.50191	40.29809	5.007	5.21776	41.58224
5.008	11.98402	34.81598	5.008	9.79569	37.00431	5.008	8.00757	38.79243	5.008	6.50866	40.29134	5.008	5.22502	41.57498
5.009	11.98858	34.81142	5.009	9.80112	36.99888	5.009	8.01371	38.78629	5.009	6.5154	40.2846	5.009	5.23228	41.56772
5.01	11.99314	34.80686	5.01	9.80655	36.99345	5.01	8.01986	38.78014	5.01	6.52214	40.27786	5.01	5.23953	41.56047
5.011	11.99769	34.80231	5.011	9.81198	36.98802	5.011	8.02599	38.77401	5.011	6.52887	40.27113	5.011	5.24678	41.55322
5.012	12.00224	34.79776	5.012	9.8174	36.9826	5.012	8.03213	38.76787	5.012	6.53561	40.26439	5.012	5.25402	41.54598
5.013	12.00678	34.79322	5.013	9.82282	36.97718	5.013	8.03826	38.76174	5.013	6.54233	40.25767	5.013	5.26126	41.53874
5.014	12.01133	34.78867	5.014	9.82823	36.97177	5.014	8.04439	38.75561	5.014	6.54906	40.25094	5.014	5.2685	41.5315
5.015	12.01587	34.78413	5.015	9.83364	36.96636	5.015	8.05051	38.74949	5.015	6.55578	40.24422	5.015	5.27573	41.52427
5.016	12.02041	34.77959	5.016	9.83905	36.96095	5.016	8.05663	38.74337	5.016	6.56249	40.23751	5.016	5.28295	41.51705
5.017	12.02494	34.77506	5.017	9.84446	36.95554	5.017	8.06274	38.73726	5.017	6.5692	40.2308	5.017	5.29017	41.50983
5.018	12.02948	34.77052	5.018	9.84986	36.95014	5.018	8.06886	38.73114	5.018	6.57591	40.22409	5.018	5.29739	41.50261
5.019	12.03401	34.76599	5.019	9.85526	36.94474	5.019	8.07497	38.72503	5.019	6.58262	40.21738	5.019	5.3046	41.4954
5.02	12.03854	34.76146	5.02	9.86066	36.93934	5.02	8.08107	38.71893	5.02	6.58931	40.21069	5.02	5.31181	41.48819
5.021	12.04306	34.75694	5.021	9.86605	36.93395	5.021	8.08717	38.71283	5.021	6.59601	40.20399	5.021	5.31902	41.48098
5.022	12.04758	34.75242	5.022	9.87144	36.92856	5.022	8.09327	38.70673	5.022	6.6027	40.1973	5.022	5.32622	41.47378
5.023	12.0521	34.7479	5.023	9.87682	36.92318	5.023	8.09936	38.70064	5.023	6.60939	40.19061	5.023	5.33341	41.46659
5.024	12.05662	34.74338	5.024	9.88221	36.91779	5.024	8.10545	38.69455	5.024	6.61607	40.18393	5.024	5.3406	41.4594
5.025	12.06113	34.73887	5.025	9.88759	36.91241	5.025	8.11154	38.68846	5.025	6.62275	40.17725	5.025	5.34779	41.45221
5.026	12.06565	34.73435	5.026	9.89296	36.90704	5.026	8.11762	38.68238	5.026	6.62943	40.17057	5.026	5.35497	41.44503
5.027	12.07016	34.72984	5.027	9.89834	36.90166	5.027	8.1237	38.6763	5.027	6.6361	40.1639	5.027	5.36215	41.43785
5.028	12.07466	34.72534	5.028	9.90371	36.89629	5.028	8.12978	38.67022	5.028	6.64277	40.15723	5.028	5.36933	41.43067
5.029	12.07917	34.72083	5.029	9.90907	36.89093	5.029	8.13585	38.66415	5.029	6.64943	40.15057	5.029	5.3765	41.4235
5.03	12.08367	34.71633	5.03	9.91444	36.88556	5.03	8.14192	38.65808	5.03	6.65609	40.14391	5.03	5.38366	41.41634
5.031	12.08816	34.71184	5.031	9.9198	36.8802	5.031	8.14798	38.65202	5.031	6.66275	40.13725	5.031	5.39082	41.40918
5.032	12.09266	34.70734	5.032	9.92515	36.87485	5.032	8.15405	38.64595	5.032	6.6694	40.1306	5.032	5.39798	41.40202
5.033	12.09715	34.70285	5.033	9.93051	36.86949	5.033	8.1601	38.6399	5.033	6.67604	40.12396	5.033	5.40513	41.39487
5.034	12.10164	34.69836	5.034	9.93586	36.86414	5.034	8.16616	38.63384	5.034	6.68269	40.11731	5.034	5.41228	41.38772
5.035	12.10613	34.69387	5.035	9.94121	36.85879	5.035	8.17221	38.62779	5.035	6.68933	40.11067	5.035	5.41943	41.38057
5.036	12.11061	34.68939	5.036	9.94655	36.85345	5.036	8.17826	38.62174	5.036	6.69596	40.10404	5.036	5.42657	41.37343
5.037	12.1151	34.6849	5.037	9.95189	36.84811	5.037	8.1843	38.6157	5.037	6.7026	40.0974	5.037	5.4337	41.3663
5.038	12.11958	34.68042	5.038	9.95723	36.84277	5.038	8.19034	38.60966	5.038	6.70922	40.09078	5.038	5.44083	41.35917
5.039	12.12405	34.67595	5.039	9.96257	36.83743	5.039	8.19638	38.60362	5.039	6.71585	40.08415	5.039	5.44796	41.35204
5.04	12.12853	34.67147	5.04	9.9679	36.8321	5.04	8.20241	38.59759	5.04	6.72247	40.07753	5.04	5.45508	41.34492
5.041	12.133	34.667	5.041	9.97323	36.82677	5.041	8.20844	38.59156	5.041	6.72908	40.07092	5.041	5.4622	41.3378
5.042	12.13747	34.66253	5.042	9.97855	36.82145	5.042	8.21446	38.58554	5.042	6.7357	40.0643	5.042	5.46932	41.33068
5.043	12.14193	34.65807	5.043	9.98387	36.81613	5.043	8.22048	38.57952	5.043	6.7423	40.0577	5.043	5.47643	41.32357
5.044	12.1464	34.6536	5.044	9.98919	36.81081	5.044	8.2265	38.5735	5.044	6.74891	40.05109	5.044	5.48353	41.31647
5.045	12.15086	34.64914	5.045	9.99451	36.80549	5.045	8.23252	38.56748	5.045	6.75551	40.04449	5.045	5.49064	41.30936
5.046	12.15532	34.64468	5.046	9.99982	36.80018	5.046	8.23853	38.56147	5.046	6.76211	40.03789	5.046	5.49773	41.30227
5.047	12.15977	34.64023	5.047	10.00513	36.79487	5.047	8.24454	38.55546	5.047	6.7687	40.0313	5.047	5.50483	41.29517
5.048	12.16422	34.63578	5.048	10.01044	36.78956	5.048	8.25054	38.54946	5.048	6.77529	40.02471	5.048	5.51192	41.28808
5.049	12.16868	34.63132	5.049	10.01574	36.78426	5.049	8.25654	38.54346	5.049	6.78187	40.01813	5.049	5.519	41.281
5.05	12.17312	34.62688	5.05	10.02104	36.77896	5.05	8.26254	38.53746	5.05	6.78845	40.01155	5.05	5.52608	41.27392
5.051	12.17757	34.62243	5.051	10.02634	36.77366	5.051	8.26853	38.53147	5.051	6.79503	40.00497	5.051	5.53316	41.26684
5.052	12.18201	34.61799	5.052	10.03163	36.76837	5.052	8.27452	38.52548	5.052	6.8016	39.9984	5.052	5.54023	41.25977
5.053	12.18645	34.61355	5.053	10.03692	36.76308	5.053	8.28051	38.51949	5.053	6.80817	39.99183	5.053	5.5473	41.2527
5.054	12.19089	34.60911	5.054	10.04221	36.75779	5.054	8.28649	38.51351	5.054	6.81474	39.98526	5.054	5.55436	41.24564
5.055	12.19532	34.60468	5.055	10.04749	36.75251	5.055	8.29247	38.50753	5.055	6.8213	39.9787	5.055	5.56142	41.23858
5.056	12.19975	34.60025	5.056	10.05278	36.74722	5.056	8.29844	38.50156	5.056	6.82785	39.97215	5.056	5.56848	41.23152
5.057	12.20418	34.59582	5.057	10.05805	36.74195	5.057	8.30441	38.49559	5.057	6.83441	39.96559	5.057	5.57553	41.22447
5.058	12.20861	34.59139	5.058	10.06333	36.73667	5.058	8.31038	38.48962	5.058	6.84096	39.95904	5.058	5.58258	41.21742
5.059	12.21303	34.58697	5.059	10.0686	36.7314	5.059	8.31635	38.48365	5.059	6.8475	39.9525	5.059	5.58962	41.21038
5.06	12.21745	34.58255	5.06	10.07387	36.72613	5.06	8.32231	38.47769	5.06	6.85405	39.94595	5.06	5.59666	41.20334
5.061	12.22187	34.57813	5.061	10.07914	36.72086	5.061	8.32827	38.47173	5.061	6.86058	39.93942	5.061	5.6037	41.1963
5.062	12.22629	34.57371	5.062	10.0844	36.7156	5.062	8.33422	38.46578	5.062	6.86712	39.93288	5.062	5.61073	41.18927
5.063	12.2307	34.5693	5.063	10.08966	36.71034	5.063	8.34017	38.45983	5.063	6.87365	39.92635	5.063	5.61775	41.18225
5.064	12.23511	34.56489	5.064	10.09491	36.70509	5.064	8.34612	38.45388	5.064	6.88017	39.91983	5.064	5.62477	41.17523
5.065	12.23952	34.56048	5.065	10.10017	36.69983	5.065	8.35206	38.44794	5.065	6.8867	39.9133	5.065	5.63179	41.16821
5.066	12.24393	34.55607	5.066	10.10542	36.69458	5.066	8.358	38.442	5.066	6.89322	39.90678	5.066	5.63881	41.16119
5.067	12.24833	34.55167	5.067	10.11066	36.68934	5.067	8.36394	38.43606	5.067	6.89973	39.90027	5.067	5.64582	41.15418
5.068	12.25273	34.54727	5.068	10.11591	36.68409	5.068	8.36987	38.43013	5.068	6.90624	39.89376	5.068	5.65282	41.14718
5.069	12.25713	34.54287	5.069	10.12115	36.67885	5.069	8.3758	38.4242	5.069	6.91275	39.88725	5.069	5.65982	41.14018
5.07	12.26152	34.53848	5.07	10.12639	36.67361	5.07	8.38173	38.41827	5.07	6.91925	39.88075	5.07	5.66682	41.13318
5.071	12.26592	34.53408	5.071	10.13162	36.66838	5.071	8.38765	38.41235	5.071	6.92575	39.87425	5.071	5.67381	41.12619
5.072	12.27031	34.52969	5.072	10.13685	36.66315	5.072	8.39357	38.40643	5.072	6.93225	39.86775	5.072	5.6808	41.1192
5.073	12.27469	34.52531	5.073	10.14208	36.65792	5.073	8.39949	38.40051	5.073	6.93874	39.86126	5.073	5.68779	41.11221
5.074	12.27908	34.52092	5.074	10.14731	36.65269	5.074	8.4054	38.3946	5.074	6.94523	39.85477	5.074	5.69477	41.10523
5.075	12.28346	34.51654	5.075	10.15253	36.64747	5.075	8.41131	38.38869	5.075	6.95171	39.84829	5.075	5.70175	41.09825
5.076	12.28784	34.51216	5.076	10.15775	36.64225	5.076	8.41721	38.38279	5.076	6.95819	39.84181	5.076	5.70872	41.09128
5.077	12.29222	34.50778	5.077	10.16296	36.63704	5.077	8.42311	38.37689	5.077	6.96467	39.83533	5.077	5.71569	41.08431

5.078	12.29659	34.50341	5.078	10.16818	36.63182	5.078	8.42901	38.37099	5.078	6.97114	39.82886	5.078	5.72265	41.07735
5.079	12.30096	34.49904	5.079	10.17339	36.62661	5.079	8.43491	38.36509	5.079	6.97761	39.82239	5.079	5.72961	41.07039
5.08	12.30533	34.49467	5.08	10.17859	36.62141	5.08	8.4408	38.3592	5.08	6.98407	39.81593	5.08	5.73657	41.06343
5.081	12.3097	34.4903	5.081	10.1838	36.6162	5.081	8.44669	38.35331	5.081	6.99053	39.80947	5.081	5.74352	41.05648
5.082	12.31406	34.48594	5.082	10.189	36.611	5.082	8.45257	38.34743	5.082	6.99699	39.80301	5.082	5.75047	41.04953
5.083	12.31842	34.48158	5.083	10.1942	36.6058	5.083	8.45845	38.34155	5.083	7.00344	39.79656	5.083	5.75741	41.04259
5.084	12.32278	34.47722	5.084	10.19939	36.60061	5.084	8.46433	38.33567	5.084	7.00989	39.79011	5.084	5.76435	41.03565
5.085	12.32714	34.47286	5.085	10.20458	36.59542	5.085	8.4702	38.3298	5.085	7.01634	39.78366	5.085	5.77129	41.02871
5.086	12.33149	34.46851	5.086	10.20977	36.59023	5.086	8.47607	38.32393	5.086	7.02278	39.77722	5.086	5.77822	41.02178
5.087	12.33584	34.46416	5.087	10.21496	36.58504	5.087	8.48194	38.31806	5.087	7.02922	39.77078	5.087	5.78515	41.01485
5.088	12.34019	34.45981	5.088	10.22014	36.57986	5.088	8.4878	38.3122	5.088	7.03565	39.76435	5.088	5.79207	41.00793
5.089	12.34454	34.45546	5.089	10.22532	36.57468	5.089	8.49366	38.30634	5.089	7.04208	39.75792	5.089	5.79899	41.00101
5.09	12.34888	34.45112	5.09	10.23049	36.56951	5.09	8.49952	38.30048	5.09	7.04851	39.75149	5.09	5.8059	40.9941
5.091	12.35322	34.44678	5.091	10.23567	36.56433	5.091	8.50537	38.29463	5.091	7.05493	39.74507	5.091	5.81282	40.98718
5.092	12.35756	34.44244	5.092	10.24084	36.55916	5.092	8.51122	38.28878	5.092	7.06135	39.73865	5.092	5.81972	40.98028
5.093	12.3619	34.4381	5.093	10.246	36.554	5.093	8.51707	38.28293	5.093	7.06777	39.73223	5.093	5.82663	40.97337
5.094	12.36623	34.43377	5.094	10.25117	36.54883	5.094	8.52291	38.27709	5.094	7.07418	39.72582	5.094	5.83352	40.96648
5.095	12.37056	34.42944	5.095	10.25633	36.54367	5.095	8.52875	38.27125	5.095	7.08059	39.71941	5.095	5.84042	40.95958
5.096	12.37489	34.42511	5.096	10.26149	36.53851	5.096	8.53459	38.26541	5.096	7.08699	39.71301	5.096	5.84731	40.95269
5.097	12.37922	34.42078	5.097	10.26664	36.53336	5.097	8.54042	38.25958	5.097	7.09339	39.70661	5.097	5.8542	40.9458
5.098	12.38354	34.41646	5.098	10.27179	36.52821	5.098	8.54625	38.25375	5.098	7.09979	39.70021	5.098	5.86108	40.93892
5.099	12.38786	34.41214	5.099	10.27694	36.52306	5.099	8.55207	38.24793	5.099	7.10618	39.69382	5.099	5.86796	40.93204
5.1	12.39218	34.40782	5.1	10.28209	36.51791	5.1	8.55789	38.24211	5.1	7.11257	39.68743	5.1	5.87483	40.92517
5.101	12.39649	34.40351	5.101	10.28723	36.51277	5.101	8.56371	38.23629	5.101	7.11896	39.68104	5.101	5.8817	40.9183
5.102	12.40081	34.39919	5.102	10.29237	36.50763	5.102	8.56953	38.23047	5.102	7.12534	39.67466	5.102	5.88857	40.91143
5.103	12.40512	34.39488	5.103	10.29751	36.50249	5.103	8.57534	38.22466	5.103	7.13172	39.66828	5.103	5.89543	40.90457
5.104	12.40943	34.39057	5.104	10.30264	36.49736	5.104	8.58115	38.21885	5.104	7.13809	39.66191	5.104	5.90229	40.89771
5.105	12.41373	34.38627	5.105	10.30777	36.49223	5.105	8.58695	38.21305	5.105	7.14446	39.65554	5.105	5.90915	40.89085
5.106	12.41803	34.38197	5.106	10.3129	36.4871	5.106	8.59276	38.20724	5.106	7.15083	39.64917	5.106	5.916	40.884
5.107	12.42233	34.37767	5.107	10.31802	36.48198	5.107	8.59855	38.20145	5.107	7.15719	39.64281	5.107	5.92284	40.87716
5.108	12.42663	34.37337	5.108	10.32315	36.47685	5.108	8.60435	38.19565	5.108	7.16355	39.63645	5.108	5.92969	40.87031
5.109	12.43093	34.36907	5.109	10.32826	36.47174	5.109	8.61014	38.18986	5.109	7.16991	39.63009	5.109	5.93652	40.86348
5.11	12.43522	34.36478	5.11	10.33338	36.46662	5.11	8.61593	38.18407	5.11	7.17626	39.62374	5.11	5.94336	40.85664
5.111	12.43951	34.36049	5.111	10.33849	36.46151	5.111	8.62171	38.17829	5.111	7.18261	39.61739	5.111	5.95019	40.84981
5.112	12.4438	34.3562	5.112	10.3436	36.4564	5.112	8.62749	38.17251	5.112	7.18895	39.61105	5.112	5.95701	40.84299
5.113	12.44808	34.35192	5.113	10.34871	36.45129	5.113	8.63327	38.16673	5.113	7.19529	39.60471	5.113	5.96384	40.83616
5.114	12.45237	34.34763	5.114	10.35381	36.44619	5.114	8.63905	38.16095	5.114	7.20163	39.59837	5.114	5.97066	40.82934
5.115	12.45665	34.34335	5.115	10.35891	36.44109	5.115	8.64482	38.15518	5.115	7.20796	39.59204	5.115	5.97747	40.82253
5.116	12.46092	34.33908	5.116	10.36401	36.43599	5.116	8.65059	38.14941	5.116	7.21429	39.58571	5.116	5.98428	40.81572
5.117	12.4652	34.3348	5.117	10.3691	36.4309	5.117	8.65635	38.14365	5.117	7.22061	39.57939	5.117	5.99109	40.80891
5.118	12.46947	34.33053	5.118	10.3742	36.4258	5.118	8.66211	38.13789	5.118	7.22694	39.57306	5.118	5.99789	40.80211
5.119	12.47374	34.32626	5.119	10.37929	36.42071	5.119	8.66787	38.13213	5.119	7.23325	39.56675	5.119	6.00469	40.79531
5.12	12.47801	34.32199	5.12	10.38437	36.41563	5.12	8.67362	38.12638	5.12	7.23957	39.56043	5.12	6.01148	40.78852
5.121	12.48227	34.31773	5.121	10.38945	36.41055	5.121	8.67937	38.12063	5.121	7.24588	39.55412	5.121	6.01827	40.78173
5.122	12.48654	34.31346	5.122	10.39453	36.40547	5.122	8.68512	38.11488	5.122	7.25219	39.54781	5.122	6.02506	40.77494
5.123	12.4908	34.3092	5.123	10.39961	36.40039	5.123	8.69086	38.10914	5.123	7.25849	39.54151	5.123	6.03184	40.76816
5.124	12.49505	34.30495	5.124	10.40468	36.39532	5.124	8.69661	38.10339	5.124	7.26479	39.53521	5.124	6.03862	40.76138
5.125	12.49931	34.30069	5.125	10.40975	36.39025	5.125	8.70234	38.09766	5.125	7.27109	39.52891	5.125	6.04539	40.75461
5.126	12.50356	34.29644	5.126	10.41482	36.38518	5.126	8.70808	38.09192	5.126	7.27738	39.52262	5.126	6.05216	40.74784
5.127	12.50781	34.29219	5.127	10.41989	36.38011	5.127	8.71381	38.08619	5.127	7.28367	39.51633	5.127	6.05893	40.74107
5.128	12.51206	34.28794	5.128	10.42495	36.37505	5.128	8.71953	38.08047	5.128	7.28995	39.51005	5.128	6.06569	40.73431
5.129	12.51631	34.28369	5.129	10.43001	36.36999	5.129	8.72526	38.07474	5.129	7.29623	39.50377	5.129	6.07245	40.72755
5.13	12.52055	34.27945	5.13	10.43506	36.36494	5.13	8.73098	38.06902	5.13	7.30251	39.49749	5.13	6.07921	40.72079
5.131	12.52479	34.27521	5.131	10.44012	36.35988	5.131	8.7367	38.0633	5.131	7.30879	39.49121	5.131	6.08596	40.71404
5.132	12.52903	34.27097	5.132	10.44517	36.35483	5.132	8.74241	38.05759	5.132	7.31506	39.48494	5.132	6.0927	40.7073
5.133	12.53326	34.26674	5.133	10.45022	36.34978	5.133	8.74812	38.05188	5.133	7.32132	39.47868	5.133	6.09945	40.70055
5.134	12.53749	34.26251	5.134	10.45526	36.34474	5.134	8.75383	38.04617	5.134	7.32759	39.47241	5.134	6.10619	40.69381
5.135	12.54173	34.25827	5.135	10.4603	36.3397	5.135	8.75953	38.04047	5.135	7.33384	39.46616	5.135	6.11292	40.68708
5.136	12.54595	34.25405	5.136	10.46534	36.33466	5.136	8.76523	38.03477	5.136	7.3401	39.4599	5.136	6.11965	40.68035
5.137	12.55018	34.24982	5.137	10.47037	36.32963	5.137	8.77093	38.02907	5.137	7.34635	39.45365	5.137	6.12638	40.67362
5.138	12.5544	34.2456	5.138	10.47541	36.32459	5.138	8.77662	38.02338	5.138	7.3526	39.4474	5.138	6.1331	40.6669
5.139	12.55862	34.24138	5.139	10.48044	36.31956	5.139	8.78231	38.01769	5.139	7.35885	39.44115	5.139	6.13982	40.66018
5.14	12.56284	34.23716	5.14	10.48546	36.31454	5.14	8.788	38.012	5.14	7.36509	39.43491	5.14	6.14654	40.65346
5.141	12.56706	34.23294	5.141	10.49049	36.30951	5.141	8.79369	38.00631	5.141	7.37132	39.42868	5.141	6.15325	40.64675
5.142	12.57127	34.22873	5.142	10.49551	36.30449	5.142	8.79937	38.00063	5.142	7.37756	39.42244	5.142	6.15996	40.64004
5.143	12.57548	34.22452	5.143	10.50053	36.29947	5.143	8.80504	37.99496	5.143	7.38379	39.41621	5.143	6.16666	40.63334
5.144	12.57969	34.22031	5.144	10.50554	36.29446	5.144	8.81072	37.98928	5.144	7.39001	39.40999	5.144	6.17336	40.62664
5.145	12.58389	34.21611	5.145	10.51055	36.28945	5.145	8.81639	37.98361	5.145	7.39624	39.40376	5.145	6.18006	40.61994
5.146	12.5881	34.2119	5.146	10.51556	36.28444	5.146	8.82206	37.97794	5.146	7.40246	39.39754	5.146	6.18675	40.61325
5.147	12.5923	34.2077	5.147	10.52057	36.27943	5.147	8.82772	37.97228	5.147	7.40867	39.39133	5.147	6.19344	40.60656
5.148	12.5965	34.2035	5.148	10.52557	36.27443	5.148	8.83338	37.96662	5.148	7.41488	39.38512	5.148	6.20012	40.59988
5.149	12.60069	34.19931	5.149	10.53057	36.26943	5.149	8.83904	37.96096	5.149	7.42109	39.37891	5.149	6.2068	40.5932
5.15	12.60489	34.19511	5.15	10.53557	36.26443	5.15	8.84469	37.95531	5.15	7.4273	39.3727	5.15	6.21348	40.58652
5.151	12.60908	34.19092	5.151	10.54056	36.25944	5.151	8.85034	37.94966	5.151	7.4335	39.3665	5.151	6.22015	40.57985
5.152	12.61326	34.18674	5.152	10.54555	36.25445	5.152	8.85599	37.94401	5.152	7.4397	39.3603	5.152	6.22682	40.57318
5.153	12.61745	34.18255	5.153	10.55054	36.24946	5.153	8.86163	37.93837	5.153	7.44589	39.35411	5.153	6.23348	40.56652
5.154	12.62163	34.17837	5.154	10.55553	36.24447	5.154	8.86728	37.93272	5.154	7.45208	39.34792	5.154	6.24014	40.55986
5.155	12.62582	34.17418	5.155	10.56051	36.23949	5.155	8.87291	37.92709	5.155	7.45827	39.34173	5.155	6.2468	40.5532
5.156	12.62999	34.17001	5.156	10.56549	36.23451	5.156	8.87855	37.92145	5.156	7.46445	39.33555	5.156	6.25345	40.54655
5.157	12.63417	34.16583	5.157	10.57047	36.22953	5.157	8.88418	37.91582	5.157	7.47063	39.32937	5.157	6.2601	40.5399
5.158	12.63835	34.16165	5.158	10.57544	36.22456	5.158	8.88981	37.91019	5.158	7.47681	39.32319	5.158	6.26675	40.53325
5.159	12.64252	34.15748	5.159	10.58041	36.21959	5.159	8.89543	37.90457	5.159	7.48298	39.31702	5.159	6.27339	40.52661
5.16	12.64669	34.15331	5.16	10.58538	36.21462	5.16	8.90105	37.89895	5.16	7.48915	39.31085	5.16	6.28003	40.51997
5.161	12.65085	34.14915	5.161	10.59035	36.20965	5.161	8.90667	37.89333	5.161	7.49531	39.30469	5.161	6.28666	40.51334
5.162	12.65502	34.14498	5.162	10.59531	36.20469	5.162	8.91229	37.88771	5.162	7.50147	39.29853	5.162	6.29329	40.50671
5.163	12.65918	34.14082	5.163	10.60027	36.19973	5.163	8.9179	37.8821	5.163	7.50763	39.29237	5.163	6.29992	40.50008
5.164	12.66334	34.13666	5.164	10.60523	36.19477	5.164	8.92351	37.87649	5.164	7.51379	39.28621	5.164	6.30654	40.49346
5.165	12.6675	34.1325	5.165	10.61018	36.18982	5.165	8.92911	37.87089	5.165	7.51994	39.28006	5.165	6.31316	40.48684
5.166	12.67165	34.12835	5.166	10.61513	36.18487	5.166	8.93471	37.86529	5.166	7.52608	39.27392	5.166	6.31977	40.48023
5.167	12.6758	34.1242	5.167	10.62008	36.17992	5.167	8.94031	37.85969	5.167	7.53223	39.26777	5.167	6.32638	40.47362
5.168	12.67995	34.12005	5.168	10.62502	36.17498	5.168	8.94591	37.85409	5.168	7.53837	39.26163	5.168	6.33299	40.46701
5.169	12.6841	34.1159	5.169	10.62997	36.17003	5.169	8.9515	37.8485	5.169	7.54451	39.25549	5.169	6.33959	40.46041
5.17	12.68824	34.11176	5.17	10.63491	36.16509	5.17	8.95709	37.84291	5.17	7.55064	39.24936	5.17	6.34619	40.45381
5.171	12.69239	34.10761	5.171	10.63984	36.16016	5.171	8.96267	37.83733	5.171	7.55677	39.24323	5.171	6.35279	40.44721
5.172	12.69653	34.10347	5.172	10.64478	36.15522	5.172	8.96826	37.83174	5.172	7.56289	39.23711	5.172	6.35938	40.44062
5.173	12.70067	34.09933	5.173	10.64971	36.15029	5.173	8.97383	37.82617	5.173	7.56902	39.23098	5.173	6.36596	40.43404
5.174	12.7048	34.0952	5.174	10.65464	36.14536	5.174	8.97941	37.82059	5.174	7.57514	39.22486	5.174	6.37255	40.42745
5.175	12.70893	34.09107	5.175	10.65956	36.14044	5.175	8.98498	37.81502	5.175	7.58125	39.21875	5.175	6.37913	40.42087
5.176	12.71306	34.08694	5.176	10.66448	36.13552	5.176	8.99055	37.80945	5.176	7.58736	39.21264	5.176	6.3857	40.4143
5.177	12.71719	34.08281	5.177	10.6694	36.1306	5.177	8.99612	37.80388	5.177	7.59347	39.20653	5.177	6.39228	40.40772
5.178	12.72132	34.07868	5.178	10.67432	36.12568	5.178	9.00168	37.79832	5.178	7.59958	39.20042	5.178	6.39885	40.40115
5.179	12.72544	34.07456	5.179	10.67923	36.12077	5.179	9.00724	37.79276	5.179	7.60568	39.19432	5.179	6.40541	40.39459
5.18	12.72956	34.07044	5.18	10.68415	36.11585	5.18	9.0128	37.7872	5.18	7.61177	39.18823	5.18	6.41197	40.38803
5.181	12.73368	34.06632	5.181	10.68905	36.11095	5.181	9.01835	37.78165	5.181	7.61787	39.18213	5.181	6.41853	40.38147
5.182	12.7378	34.0622	5.182	10.69396	36.10604	5.182	9.0239	37.7761	5.182	7.62396	39.17604	5.182	6.42508	40.37492
5.183	12.74191	34.05809	5.183	10.69886	36.10114	5.183	9.02945	37.77055	5.183	7.63005	39.16995	5.183	6.43163	40.36837
5.184	12.74602	34.05398	5.184	10.70376	36.09624	5.184	9.03499	37.76501	5.184	7.63613	39.16387	5.184	6.43818	40.36182
5.185	12.75013	34.04987	5.185	10.70866	36.09134	5.185	9.04053	37.75947	5.185	7.64221	39.15779	5.185	6.44472	40.35528
5.186	12.75424	34.04576	5.186	10.71355	36.08645	5.186	9.04607	37.75393	5.186	7.64829	39.15171	5.186	6.45126	40.34874
5.187	12.75834	34.04166	5.187	10.71844	36.08156	5.187	9.0516	37.7484	5.187	7.65436	39.14564	5.187	6.45779	40.34221
5.188	12.76245	34.03755	5.188	10.72333	36.07667	5.188	9.05713	37.74287	5.188	7.66043	39.13957	5.188	6.46432	40.33568
5.189	12.76655	34.03345	5.189	10.72822	36.07178	5.189	9.06266	37.73734	5.189	7.66649	39.13351	5.189	6.47085	40.32915
5.19	12.77064	34.02936	5.19	10.7331	36.0669	5.19	9.06819	37.73181	5.19	7.67256	39.12744	5.19	6.47737	40.32263
5.191	12.77474	34.02526	5.191	10.73798	36.06202	5.191	9.07371	37.72629	5.191	7.67862	39.12138	5.191	6.48389	40.31611
5.192	12.77883	34.02117	5.192	10.74286	36.05714	5.192	9.07923	37.72077	5.192	7.68467	39.11533	5.192	6.49041	40.30959
5.193	12.78292	34.01708	5.193	10.74773	36.05227	5.193	9.08474	37.71526	5.193	7.69072	39.10928	5.193	6.49692	40.30308
5.194	12.78701	34.01299	5.194	10.7526	36.0474	5.194	9.09025	37.70975	5.194	7.69677	39.10323	5.194	6.50343	40.29657
5.195	12.79109	34.00891	5.195	10.75747	36.04253	5.195	9.09576	37.70424	5.195	7.70282	39.09718	5.195	6.50993	40.29007
5.196	12.79518	34.00482	5.196	10.76234	36.03766	5.196	9.10127	37.69873	5.196	7.70886	39.09114	5.196	6.51643	40.28357
5.197	12.79926	34.00074	5.197	10.7672	36.0328	5.197	9.10677	37.69323	5.197	7.7149	39.0851	5.197	6.52293	40.27707
5.198	12.80334	33.99666	5.198	10.77206	36.02794	5.198	9.11227	37.68773	5.198	7.72093	39.07907	5.198	6.52942	40.27058
5.199	12.80741	33.99259	5.199	10.77692	36.02308	5.199	9.11776	37.68224	5.199	7.72696	39.07304	5.199	6.53591	40.26409
5.2	12.81149	33.98851	5.2	10.78177	36.01823	5.2	9.12326	37.67674	5.2	7.73299	39.06701	5.2	6.5424	40.2576
5.201	12.81556	33.98444	5.201	10.78662	36.01338	5.201	9.12875	37.67125	5.201	7.73901	39.06099	5.201	6.54888	40.25112
5.202	12.81963	33.98037	5.202	10.79147	36.00853	5.202	9.13423	37.66577	5.202	7.74503	39.05497	5.202	6.55536	40.24464
5.203	12.82369	33.97631	5.203	10.79632	36.00368	5.203	9.13971	37.66029	5.203	7.75105	39.04895	5.203	6.56183	40.23817
5.204	12.82776	33.97224	5.204	10.80116	35.99884	5.204	9.14519	37.65481	5.204	7.75706	39.04294	5.204	6.5683	40.2317
5.205	12.83182	33.96818	5.205	10.806	35.994	5.205	9.15067	37.64933	5.205	7.76307	39.03693	5.205	6.57477	40.22523
5.206	12.83588	33.96412	5.206	10.81084	35.98916	5.206	9.15615	37.64385	5.206	7.76908	39.03092	5.206	6.58123	40.21877
5.207	12.83994	33.96006	5.207	10.81567	35.98433	5.207	9.16162	37.63838	5.207	7.77508	39.02492	5.207	6.58769	40.21231
5.208	12.84399	33.95601	5.208	10.82051	35.97949	5.208	9.16708	37.63292	5.208	7.78108	39.01892	5.208	6.59415	40.20585
5.209	12.84804	33.95196	5.209	10.82534	35.97466	5.209	9.17255	37.62745	5.209	7.78708	39.01292	5.209	6.6006	40.1994
5.21	12.85209	33.94791	5.21	10.83016	35.96984	5.21	9.17801	37.62199	5.21	7.79307	39.00693	5.21	6.60705	40.19295
5.211	12.85614	33.94386	5.211	10.83499	35.96501	5.211	9.18347	37.61653	5.211	7.79906	39.00094	5.211	6.61349	40.18651
5.212	12.86019	33.93981	5.212	10.83981	35.96019	5.212	9.18892	37.61108	5.212	7.80505	38.99495	5.212	6.61993	40.18007
5.213	12.86423	33.93577	5.213	10.84463	35.95537	5.213	9.19437	37.60563	5.213	7.81103	38.98897	5.213	6.62637	40.17363
5.214	12.86827	33.93173	5.214	10.84944	35.95056	5.214	9.19982	37.60018	5.214	7.81701	38.98299	5.214	6.6328	40.1672
5.215	12.87231	33.92769	5.215	10.85425	35.94575	5.215	9.20527	37.59473	5.215	7.82299	38.97701	5.215	6.63923	40.16077
5.216	12.87635	33.92365	5.216	10.85906	35.94094	5.216	9.21071	37.58929	5.216	7.82896	38.97104	5.216	6.64566	40.15434
5.217	12.88038	33.91962	5.217	10.86387	35.93613	5.217	9.21615	37.58385	5.217	7.83493	38.96507	5.217	6.65208	40.14792
5.218	12.88441	33.91559	5.218	10.86868	35.93132	5.218	9.22158	37.57842	5.218	7.84089	38.95911	5.218	6.6585	40.1415
5.219	12.88844	33.91156	5.219	10.87348	35.92652	5.219	9.22702	37.57298	5.219	7.84685	38.95315	5.219	6.66492	40.13508
5.22	12.89247	33.90753	5.22	10.87828	35.92172	5.22	9.23245	37.56755	5.22	7.85281	38.94719	5.22	6.67133	40.12867
5.221	12.89649	33.90351	5.221	10.88307	35.91693	5.221	9.23787	37.56213	5.221	7.85877	38.94123	5.221	6.67773	40.12227
5.222	12.90052	33.89948	5.222	10.88787	35.91213	5.222	9.2433	37.5567	5.222	7.86472	38.93528	5.222	6.68414	40.11586
5.223	12.90454	33.89546	5.223	10.89266	35.90734	5.223	9.24872	37.55128	5.223	7.87067	38.92933	5.223	6.69054	40.10946
5.224	12.90855	33.89145	5.224	10.89745	35.90255	5.224	9.25413	37.54587	5.224	7.87661	38.92339	5.224	6.69694	40.10306
5.225	12.91257	33.88743	5.225	10.90223	35.89777	5.225	9.25955	37.54045	5.225	7.88255	38.91745	5.225	6.70333	40.09667
5.226	12.91658	33.88342	5.226	10.90701	35.89299	5.226	9.26496	37.53504	5.226	7.88849	38.91151	5.226	6.70972	40.09028
5.227	12.92059	33.87941	5.227	10.91179	35.88821	5.227	9.27037	37.52963	5.227	7.89443	38.90557	5.227	6.7161	40.0839
5.228	12.9246	33.8754	5.228	10.91657	35.88343	5.228	9.27577	37.52423	5.228	7.90036	38.89964	5.228	6.72248	40.07752
5.229	12.92861	33.87139	5.229	10.92134	35.87866	5.229	9.28117	37.51883	5.229	7.90628	38.89372	5.229	6.72886	40.07114
5.23	12.93261	33.86739	5.23	10.92612	35.87388	5.23	9.28657	37.51343	5.23	7.91221	38.88779	5.23	6.73524	40.06476
5.231	12.93661	33.86339	5.231	10.93088	35.86912	5.231	9.29197	37.50803	5.231	7.91813	38.88187	5.231	6.74161	40.05839
5.232	12.94061	33.85939	5.232	10.93565	35.86435	5.232	9.29736	37.50264	5.232	7.92405	38.87595	5.232	6.74798	40.05202
5.233	12.94461	33.85539	5.233	10.94041	35.85959	5.233	9.30275	37.49725	5.233	7.92996	38.87004	5.233	6.75434	40.04566
5.234	12.9486	33.8514	5.234	10.94517	35.85483	5.234	9.30814	37.49186	5.234	7.93587	38.86413	5.234	6.7607	40.0393
5.235	12.9526	33.8474	5.235	10.94993	35.85007	5.235	9.31352	37.48648	5.235	7.94178	38.85822	5.235	6.76706	40.03294
5.236	12.95659	33.84341	5.236	10.95469	35.84531	5.236	9.3189	37.4811	5.236	7.94768	38.85232	5.236	6.77341	40.02659
5.237	12.96057	33.83943	5.237	10.95944	35.84056	5.237	9.32428	37.47572	5.237	7.95358	38.84642	5.237	6.77976	40.02024

5.238	12.96456	33.83544	5.238	10.96419	35.83581	5.238	9.32965	37.47035	5.238	7.95948	38.84052	5.238	6.7861	40.0139
5.239	12.96854	33.83146	5.239	10.96894	35.83106	5.239	9.33502	37.46498	5.239	7.96538	38.83462	5.239	6.79244	40.00756
5.24	12.97253	33.82747	5.24	10.97368	35.82632	5.24	9.34039	37.45961	5.24	7.97127	38.82873	5.24	6.79878	40.00122
5.241	12.9765	33.8235	5.241	10.97842	35.82158	5.241	9.34575	37.45425	5.241	7.97715	38.82285	5.241	6.80512	39.99488
5.242	12.98048	33.81952	5.242	10.98316	35.81684	5.242	9.35112	37.44888	5.242	7.98304	38.81696	5.242	6.81145	39.98855
5.243	12.98445	33.81555	5.243	10.9879	35.8121	5.243	9.35647	37.44353	5.243	7.98892	38.81108	5.243	6.81778	39.98222
5.244	12.98843	33.81157	5.244	10.99263	35.80737	5.244	9.36183	37.43817	5.244	7.99479	38.80521	5.244	6.8241	39.9759
5.245	12.9924	33.8076	5.245	10.99736	35.80264	5.245	9.36718	37.43282	5.245	8.00067	38.79933	5.245	6.83042	39.96958
5.246	12.99636	33.80364	5.246	11.00209	35.79791	5.246	9.37253	37.42747	5.246	8.00654	38.79346	5.246	6.83674	39.96326
5.247	13.00033	33.79967	5.247	11.00681	35.79319	5.247	9.37788	37.42212	5.247	8.01241	38.78759	5.247	6.84305	39.95695
5.248	13.00429	33.79571	5.248	11.01154	35.78846	5.248	9.38322	37.41678	5.248	8.01827	38.78173	5.248	6.84936	39.95064
5.249	13.00825	33.79175	5.249	11.01626	35.78374	5.249	9.38856	37.41144	5.249	8.02413	38.77587	5.249	6.85566	39.94434
5.25	13.01221	33.78779	5.25	11.02097	35.77903	5.25	9.3939	37.4061	5.25	8.02999	38.77001	5.25	6.86197	39.93803
5.251	13.01617	33.78383	5.251	11.02569	35.77431	5.251	9.39923	37.40077	5.251	8.03584	38.76416	5.251	6.86826	39.93174
5.252	13.02012	33.77988	5.252	11.0304	35.7696	5.252	9.40456	37.39544	5.252	8.04169	38.75831	5.252	6.87456	39.92544
5.253	13.02407	33.77593	5.253	11.03511	35.76489	5.253	9.40989	37.39011	5.253	8.04754	38.75246	5.253	6.88085	39.91915
5.254	13.02802	33.77198	5.254	11.03982	35.76018	5.254	9.41522	37.38478	5.254	8.05338	38.74662	5.254	6.88714	39.91286
5.255	13.03197	33.76803	5.255	11.04452	35.75548	5.255	9.42054	37.37946	5.255	8.05922	38.74078	5.255	6.89342	39.90658
5.256	13.03591	33.76409	5.256	11.04922	35.75078	5.256	9.42586	37.37414	5.256	8.06506	38.73494	5.256	6.8997	39.9003
5.257	13.03986	33.76014	5.257	11.05392	35.74608	5.257	9.43117	37.36883	5.257	8.07089	38.72911	5.257	6.90598	39.89402
5.258	13.0438	33.7562	5.258	11.05861	35.74139	5.258	9.43649	37.36351	5.258	8.07672	38.72328	5.258	6.91225	39.88775
5.259	13.04773	33.75227	5.259	11.06331	35.73669	5.259	9.4418	37.3582	5.259	8.08255	38.71745	5.259	6.91852	39.88148
5.26	13.05167	33.74833	5.26	11.068	35.732	5.26	9.4471	37.3529	5.26	8.08837	38.71163	5.26	6.92479	39.87521
5.261	13.0556	33.7444	5.261	11.07269	35.72731	5.261	9.45241	37.34759	5.261	8.09419	38.70581	5.261	6.93105	39.86895
5.262	13.05954	33.74046	5.262	11.07737	35.72263	5.262	9.45771	37.34229	5.262	8.10001	38.69999	5.262	6.93731	39.86269
5.263	13.06346	33.73654	5.263	11.08205	35.71795	5.263	9.46301	37.33699	5.263	8.10582	38.69418	5.263	6.94356	39.85644
5.264	13.06739	33.73261	5.264	11.08673	35.71327	5.264	9.4683	37.3317	5.264	8.11163	38.68837	5.264	6.94982	39.85018
5.265	13.07132	33.72868	5.265	11.09141	35.70859	5.265	9.47359	37.32641	5.265	8.11744	38.68256	5.265	6.95606	39.84394
5.266	13.07524	33.72476	5.266	11.09608	35.70392	5.266	9.47888	37.32112	5.266	8.12324	38.67676	5.266	6.96231	39.83769
5.267	13.07916	33.72084	5.267	11.10076	35.69924	5.267	9.48417	37.31583	5.267	8.12904	38.67096	5.267	6.96855	39.83145
5.268	13.08308	33.71692	5.268	11.10542	35.69458	5.268	9.48945	37.31055	5.268	8.13484	38.66516	5.268	6.97479	39.82521
5.269	13.08699	33.71301	5.269	11.11009	35.68991	5.269	9.49473	37.30527	5.269	8.14064	38.65936	5.269	6.98102	39.81898
5.27	13.09091	33.70909	5.27	11.11475	35.68525	5.27	9.50001	37.29999	5.27	8.14643	38.65357	5.27	6.98725	39.81275
5.271	13.09482	33.70518	5.271	11.11942	35.68058	5.271	9.50528	37.29472	5.271	8.15221	38.64779	5.271	6.99348	39.80652
5.272	13.09873	33.70127	5.272	11.12407	35.67593	5.272	9.51055	37.28945	5.272	8.158	38.642	5.272	6.9997	39.8003
5.273	13.10263	33.69737	5.273	11.12873	35.67127	5.273	9.51582	37.28418	5.273	8.16378	38.63622	5.273	7.00592	39.79408
5.274	13.10654	33.69346	5.274	11.13338	35.66662	5.274	9.52108	37.27892	5.274	8.16955	38.63045	5.274	7.01214	39.78786
5.275	13.11044	33.68956	5.275	11.13803	35.66197	5.275	9.52634	37.27366	5.275	8.17533	38.62467	5.275	7.01835	39.78165
5.276	13.11434	33.68566	5.276	11.14268	35.65732	5.276	9.5316	37.2684	5.276	8.1811	38.6189	5.276	7.02456	39.77544
5.277	13.11824	33.68176	5.277	11.14733	35.65267	5.277	9.53686	37.26314	5.277	8.18687	38.61313	5.277	7.03077	39.76923
5.278	13.12213	33.67787	5.278	11.15197	35.64803	5.278	9.54211	37.25789	5.278	8.19263	38.60737	5.278	7.03697	39.76303
5.279	13.12603	33.67397	5.279	11.15661	35.64339	5.279	9.54736	37.25264	5.279	8.19839	38.60161	5.279	7.04317	39.75683
5.28	13.12992	33.67008	5.28	11.16125	35.63875	5.28	9.55261	37.24739	5.28	8.20415	38.59585	5.28	7.04936	39.75064
5.281	13.13381	33.66619	5.281	11.16588	35.63412	5.281	9.55785	37.24215	5.281	8.2099	38.5901	5.281	7.05556	39.74444
5.282	13.13769	33.66231	5.282	11.17051	35.62949	5.282	9.56309	37.23691	5.282	8.21566	38.58434	5.282	7.06174	39.73826
5.283	13.14158	33.65842	5.283	11.17514	35.62486	5.283	9.56833	37.23167	5.283	8.2214	38.5786	5.283	7.06793	39.73207
5.284	13.14546	33.65454	5.284	11.17977	35.62023	5.284	9.57356	37.22644	5.284	8.22715	38.57285	5.284	7.07411	39.72589
5.285	13.14934	33.65066	5.285	11.18439	35.61561	5.285	9.5788	37.2212	5.285	8.23289	38.56711	5.285	7.08029	39.71971
5.286	13.15322	33.64678	5.286	11.18901	35.61099	5.286	9.58402	37.21598	5.286	8.23863	38.56137	5.286	7.08646	39.71354
5.287	13.1571	33.6429	5.287	11.19363	35.60637	5.287	9.58925	37.21075	5.287	8.24436	38.55564	5.287	7.09263	39.70737
5.288	13.16097	33.63903	5.288	11.19825	35.60175	5.288	9.59447	37.20553	5.288	8.25009	38.54991	5.288	7.0988	39.7012
5.289	13.16484	33.63516	5.289	11.20286	35.59714	5.289	9.59969	37.20031	5.289	8.25582	38.54418	5.289	7.10496	39.69504
5.29	13.16871	33.63129	5.29	11.20747	35.59253	5.29	9.60491	37.19509	5.29	8.26155	38.53845	5.29	7.11112	39.68888
5.291	13.17258	33.62742	5.291	11.21208	35.58792	5.291	9.61012	37.18988	5.291	8.26727	38.53273	5.291	7.11728	39.68272
5.292	13.17644	33.62356	5.292	11.21669	35.58331	5.292	9.61534	37.18466	5.292	8.27299	38.52701	5.292	7.12343	39.67657
5.293	13.1803	33.6197	5.293	11.22129	35.57871	5.293	9.62054	37.17946	5.293	8.2787	38.5213	5.293	7.12958	39.67042
5.294	13.18417	33.61583	5.294	11.22589	35.57411	5.294	9.62575	37.17425	5.294	8.28441	38.51559	5.294	7.13573	39.66427
5.295	13.18802	33.61198	5.295	11.23049	35.56951	5.295	9.63095	37.16905	5.295	8.29012	38.50988	5.295	7.14187	39.65813
5.296	13.19188	33.60812	5.296	11.23508	35.56492	5.296	9.63615	37.16385	5.296	8.29583	38.50417	5.296	7.14801	39.65199
5.297	13.19573	33.60427	5.297	11.23968	35.56032	5.297	9.64135	37.15865	5.297	8.30153	38.49847	5.297	7.15415	39.64585
5.298	13.19959	33.60041	5.298	11.24427	35.55573	5.298	9.64654	37.15346	5.298	8.30723	38.49277	5.298	7.16028	39.63972
5.299	13.20344	33.59656	5.299	11.24885	35.55115	5.299	9.65173	37.14827	5.299	8.31293	38.48707	5.299	7.16641	39.63359
5.3	13.20728	33.59272	5.3	11.25344	35.54656	5.3	9.65692	37.14308	5.3	8.31862	38.48138	5.3	7.17253	39.62747
5.301	13.21113	33.58887	5.301	11.25802	35.54198	5.301	9.6621	37.1379	5.301	8.32431	38.47569	5.301	7.17865	39.62135
5.302	13.21497	33.58503	5.302	11.2626	35.5374	5.302	9.66728	37.13272	5.302	8.32999	38.47001	5.302	7.18477	39.61523
5.303	13.21881	33.58119	5.303	11.26718	35.53282	5.303	9.67246	37.12754	5.303	8.33568	38.46432	5.303	7.19089	39.60911
5.304	13.22265	33.57735	5.304	11.27175	35.52825	5.304	9.67764	37.12236	5.304	8.34136	38.45864	5.304	7.197	39.603
5.305	13.22649	33.57351	5.305	11.27632	35.52368	5.305	9.68281	37.11719	5.305	8.34703	38.45297	5.305	7.20311	39.59689
5.306	13.23032	33.56968	5.306	11.28089	35.51911	5.306	9.68798	37.11202	5.306	8.35271	38.44729	5.306	7.20921	39.59079
5.307	13.23415	33.56585	5.307	11.28546	35.51454	5.307	9.69315	37.10685	5.307	8.35838	38.44162	5.307	7.21531	39.58469
5.308	13.23798	33.56202	5.308	11.29002	35.50998	5.308	9.69831	37.10169	5.308	8.36404	38.43596	5.308	7.22141	39.57859
5.309	13.24181	33.55819	5.309	11.29459	35.50541	5.309	9.70347	37.09653	5.309	8.36971	38.43029	5.309	7.2275	39.5725
5.31	13.24564	33.55436	5.31	11.29915	35.50085	5.31	9.70863	37.09137	5.31	8.37537	38.42463	5.31	7.23359	39.56641
5.311	13.24946	33.55054	5.311	11.3037	35.4963	5.311	9.71379	37.08621	5.311	8.38103	38.41897	5.311	7.23968	39.56032
5.312	13.25328	33.54672	5.312	11.30826	35.49174	5.312	9.71894	37.08106	5.312	8.38668	38.41332	5.312	7.24577	39.55423
5.313	13.2571	33.5429	5.313	11.31281	35.48719	5.313	9.72409	37.07591	5.313	8.39233	38.40767	5.313	7.25185	39.54815
5.314	13.26092	33.53908	5.314	11.31736	35.48264	5.314	9.72924	37.07076	5.314	8.39798	38.40202	5.314	7.25792	39.54208
5.315	13.26473	33.53527	5.315	11.3219	35.4781	5.315	9.73438	37.06562	5.315	8.40362	38.39638	5.315	7.264	39.536
5.316	13.26855	33.53145	5.316	11.32645	35.47355	5.316	9.73952	37.06048	5.316	8.40927	38.39073	5.316	7.27007	39.52993
5.317	13.27236	33.52764	5.317	11.33099	35.46901	5.317	9.74466	37.05534	5.317	8.4149	38.3851	5.317	7.27613	39.52387
5.318	13.27616	33.52384	5.318	11.33553	35.46447	5.318	9.74979	37.05021	5.318	8.42054	38.37946	5.318	7.2822	39.5178
5.319	13.27997	33.52003	5.319	11.34006	35.45994	5.319	9.75493	37.04507	5.319	8.42617	38.37383	5.319	7.28826	39.51174
5.32	13.28378	33.51622	5.32	11.34459	35.45541	5.32	9.76006	37.03994	5.32	8.4318	38.3682	5.32	7.29431	39.50569
5.321	13.28758	33.51242	5.321	11.34913	35.45087	5.321	9.76518	37.03482	5.321	8.43743	38.36257	5.321	7.30037	39.49963
5.322	13.29138	33.50862	5.322	11.35365	35.44635	5.322	9.77031	37.02969	5.322	8.44305	38.35695	5.322	7.30642	39.49358
5.323	13.29517	33.50483	5.323	11.35818	35.44182	5.323	9.77543	37.02457	5.323	8.44867	38.35133	5.323	7.31246	39.48754
5.324	13.29897	33.50103	5.324	11.3627	35.4373	5.324	9.78054	37.01946	5.324	8.45428	38.34572	5.324	7.31851	39.48149
5.325	13.30276	33.49724	5.325	11.36722	35.43278	5.325	9.78566	37.01434	5.325	8.4599	38.3401	5.325	7.32454	39.47546
5.326	13.30656	33.49344	5.326	11.37174	35.42826	5.326	9.79077	37.00923	5.326	8.46551	38.33449	5.326	7.33058	39.46942
5.327	13.31034	33.48966	5.327	11.37626	35.42374	5.327	9.79588	37.00412	5.327	8.47111	38.32889	5.327	7.33661	39.46339
5.328	13.31413	33.48587	5.328	11.38077	35.41923	5.328	9.80099	36.99901	5.328	8.47672	38.32328	5.328	7.34264	39.45736
5.329	13.31792	33.48208	5.329	11.38528	35.41472	5.329	9.80609	36.99391	5.329	8.48232	38.31768	5.329	7.34867	39.45133
5.33	13.3217	33.4783	5.33	11.38979	35.41021	5.33	9.81119	36.98881	5.33	8.48791	38.31209	5.33	7.35469	39.44531
5.331	13.32548	33.47452	5.331	11.39429	35.40571	5.331	9.81629	36.98371	5.331	8.49351	38.30649	5.331	7.36071	39.43929
5.332	13.32926	33.47074	5.332	11.3988	35.4012	5.332	9.82138	36.97862	5.332	8.4991	38.3009	5.332	7.36673	39.43327
5.333	13.33303	33.46697	5.333	11.4033	35.3967	5.333	9.82647	36.97353	5.333	8.50469	38.29531	5.333	7.37274	39.42726
5.334	13.33681	33.46319	5.334	11.40779	35.39221	5.334	9.83156	36.96844	5.334	8.51027	38.28973	5.334	7.37875	39.42125
5.335	13.34058	33.45942	5.335	11.41229	35.38771	5.335	9.83665	36.96335	5.335	8.51585	38.28415	5.335	7.38475	39.41525
5.336	13.34435	33.45565	5.336	11.41678	35.38322	5.336	9.84173	36.95827	5.336	8.52143	38.27857	5.336	7.39075	39.40925
5.337	13.34812	33.45188	5.337	11.42127	35.37873	5.337	9.84681	36.95319	5.337	8.527	38.273	5.337	7.39675	39.40325
5.338	13.35189	33.44811	5.338	11.42576	35.37424	5.338	9.85189	36.94811	5.338	8.53258	38.26742	5.338	7.40275	39.39725
5.339	13.35565	33.44435	5.339	11.43025	35.36975	5.339	9.85696	36.94304	5.339	8.53815	38.26185	5.339	7.40874	39.39126
5.34	13.35941	33.44059	5.34	11.43473	35.36527	5.34	9.86204	36.93796	5.34	8.54371	38.25629	5.34	7.41473	39.38527
5.341	13.36317	33.43683	5.341	11.43921	35.36079	5.341	9.86711	36.93289	5.341	8.54927	38.25073	5.341	7.42071	39.37929
5.342	13.36693	33.43307	5.342	11.44369	35.35631	5.342	9.87217	36.92783	5.342	8.55483	38.24517	5.342	7.4267	39.3733
5.343	13.37068	33.42932	5.343	11.44816	35.35184	5.343	9.87723	36.92277	5.343	8.56039	38.23961	5.343	7.43268	39.36732
5.344	13.37444	33.42556	5.344	11.45263	35.34737	5.344	9.88229	36.91771	5.344	8.56594	38.23406	5.344	7.43865	39.36135
5.345	13.37819	33.42181	5.345	11.4571	35.3429	5.345	9.88735	36.91265	5.345	8.57149	38.22851	5.345	7.44462	39.35538
5.346	13.38194	33.41806	5.346	11.46157	35.33843	5.346	9.89241	36.90759	5.346	8.57704	38.22296	5.346	7.45059	39.34941
5.347	13.38568	33.41432	5.347	11.46604	35.33396	5.347	9.89746	36.90254	5.347	8.58258	38.21742	5.347	7.45656	39.34344
5.348	13.38943	33.41057	5.348	11.4705	35.3295	5.348	9.90251	36.89749	5.348	8.58812	38.21188	5.348	7.46252	39.33748
5.349	13.39317	33.40683	5.349	11.47496	35.32504	5.349	9.90755	36.89245	5.349	8.59366	38.20634	5.349	7.46848	39.33152
5.35	13.39691	33.40309	5.35	11.47942	35.32058	5.35	9.9126	36.8874	5.35	8.5992	38.2008	5.35	7.47443	39.32557
5.351	13.40065	33.39935	5.351	11.48387	35.31613	5.351	9.91764	36.88236	5.351	8.60473	38.19527	5.351	7.48038	39.31962
5.352	13.40438	33.39562	5.352	11.48832	35.31168	5.352	9.92268	36.87732	5.352	8.61026	38.18974	5.352	7.48633	39.31367
5.353	13.40812	33.39188	5.353	11.49277	35.30723	5.353	9.92771	36.87229	5.353	8.61578	38.18422	5.353	7.49228	39.30772
5.354	13.41185	33.38815	5.354	11.49722	35.30278	5.354	9.93274	36.86726	5.354	8.6213	38.1787	5.354	7.49822	39.30178
5.355	13.41558	33.38442	5.355	11.50167	35.29833	5.355	9.93777	36.86223	5.355	8.62682	38.17318	5.355	7.50416	39.29584
5.356	13.41931	33.38069	5.356	11.50611	35.29389	5.356	9.9428	36.8572	5.356	8.63234	38.16766	5.356	7.51009	39.28991
5.357	13.42304	33.37696	5.357	11.51055	35.28945	5.357	9.94782	36.85218	5.357	8.63785	38.16215	5.357	7.51602	39.28398
5.358	13.42676	33.37324	5.358	11.51499	35.28501	5.358	9.95284	36.84716	5.358	8.64336	38.15664	5.358	7.52195	39.27805
5.359	13.43048	33.36952	5.359	11.51942	35.28058	5.359	9.95786	36.84214	5.359	8.64887	38.15113	5.359	7.52788	39.27212
5.36	13.4342	33.3658	5.36	11.52385	35.27615	5.36	9.96288	36.83712	5.36	8.65437	38.14563	5.36	7.5338	39.2662
5.361	13.43792	33.36208	5.361	11.52828	35.27172	5.361	9.96789	36.83211	5.361	8.65987	38.14013	5.361	7.53972	39.26028
5.362	13.44163	33.35837	5.362	11.53271	35.26729	5.362	9.9729	36.8271	5.362	8.66537	38.13463	5.362	7.54563	39.25437
5.363	13.44535	33.35465	5.363	11.53714	35.26286	5.363	9.97791	36.82209	5.363	8.67086	38.12914	5.363	7.55155	39.24845
5.364	13.44906	33.35094	5.364	11.54156	35.25844	5.364	9.98291	36.81709	5.364	8.67636	38.12364	5.364	7.55745	39.24255
5.365	13.45277	33.34723	5.365	11.54598	35.25402	5.365	9.98791	36.81209	5.365	8.68184	38.11816	5.365	7.56336	39.23664
5.366	13.45647	33.34353	5.366	11.5504	35.2496	5.366	9.99291	36.80709	5.366	8.68733	38.11267	5.366	7.56926	39.23074
5.367	13.46018	33.33982	5.367	11.55481	35.24519	5.367	9.99791	36.80209	5.367	8.69281	38.10719	5.367	7.57516	39.22484
5.368	13.46388	33.33612	5.368	11.55923	35.24077	5.368	10.0029	36.7971	5.368	8.69829	38.10171	5.368	7.58106	39.21894
5.369	13.46758	33.33242	5.369	11.56364	35.23636	5.369	10.00789	36.79211	5.369	8.70377	38.09623	5.369	7.58695	39.21305
5.37	13.47128	33.32872	5.37	11.56804	35.23196	5.37	10.01288	36.78712	5.37	8.70924	38.09076	5.37	7.59284	39.20716
5.371	13.47498	33.32502	5.371	11.57245	35.22755	5.371	10.01786	36.78214	5.371	8.71471	38.08529	5.371	7.59872	39.20128
5.372	13.47867	33.32133	5.372	11.57685	35.22315	5.372	10.02284	36.77716	5.372	8.72018	38.07982	5.372	7.6046	39.1954
5.373	13.48237	33.31763	5.373	11.58125	35.21875	5.373	10.02782	36.77218	5.373	8.72564	38.07436	5.373	7.61048	39.18952
5.374	13.48606	33.31394	5.374	11.58565	35.21435	5.374	10.0328	36.7672	5.374	8.7311	38.0689	5.374	7.61636	39.18364
5.375	13.48975	33.31025	5.375	11.59005	35.20995	5.375	10.03777	36.76223	5.375	8.73656	38.06344	5.375	7.62223	39.17777
5.376	13.49343	33.30657	5.376	11.59444	35.20556	5.376	10.04274	36.75726	5.376	8.74201	38.05799	5.376	7.6281	39.1719
5.377	13.49712	33.30288	5.377	11.59883	35.20117	5.377	10.04771	36.75229	5.377	8.74746	38.05254	5.377	7.63397	39.16603
5.378	13.5008	33.2992	5.378	11.60322	35.19678	5.378	10.05267	36.74733	5.378	8.75291	38.04709	5.378	7.63983	39.16017
5.379	13.50448	33.29552	5.379	11.60761	35.19239	5.379	10.05764	36.74236	5.379	8.75836	38.04164	5.379	7.64569	39.15431
5.38	13.50816	33.29184	5.38	11.61199	35.18801	5.38	10.0626	36.7374	5.38	8.7638	38.0362	5.38	7.65154	39.14846
5.381	13.51183	33.28817	5.381	11.61637	35.18363	5.381	10.06755	36.73245	5.381	8.76924	38.03076	5.381	7.6574	39.1426
5.382	13.51551	33.28449	5.382	11.62075	35.17925	5.382	10.07251	36.72749	5.382	8.77468	38.02532	5.382	7.66325	39.13675
5.383	13.51918	33.28082	5.383	11.62512	35.17488	5.383	10.07746	36.72254	5.383	8.78011	38.01989	5.383	7.66909	39.13091
5.384	13.52285	33.27715	5.384	11.6295	35.1705	5.384	10.08241	36.71759	5.384	8.78554	38.01446	5.384	7.67494	39.12506
5.385	13.52652	33.27348	5.385	11.63387	35.16613	5.385	10.08735	36.71265	5.385	8.79097	38.00903	5.385	7.68078	39.11922
5.386	13.53018	33.26982	5.386	11.63824	35.16176	5.386	10.09229	36.70771	5.386	8.79639	38.00361	5.386	7.68661	39.11339
5.387	13.53385	33.26615	5.387	11.6426	35.1574	5.387	10.09724	36.70276	5.387	8.80181	37.99819	5.387	7.69245	39.10755
5.388	13.53751	33.26249	5.388	11.64697	35.15303	5.388	10.10217	36.69783	5.388	8.80723	37.99277	5.388	7.69828	39.10172
5.389	13.54117	33.25883	5.389	11.65133	35.14867	5.389	10.10711	36.69289	5.389	8.81265	37.98735	5.389	7.7041	39.0959
5.39	13.54483	33.25517	5.39	11.65569	35.14431	5.39	10.11204	36.68796	5.39	8.81806	37.98194	5.39	7.70993	39.09007
5.391	13.54848	33.25152	5.391	11.66005	35.13995	5.391	10.11697	36.68303	5.391	8.82347	37.97653	5.391	7.71575	39.08425
5.392	13.55214	33.24786	5.392	11.6644	35.1356	5.392	10.1219	36.6781	5.392	8.82887	37.97113	5.392	7.72156	39.07844
5.393	13.55579	33.24421	5.393	11.66875	35.13125	5.393	10.12682	36.67318	5.393	8.83428	37.96572	5.393	7.72738	39.07262
5.394	13.55944	33.24056	5.394	11.6731	35.1269	5.394	10.13174	36.66826	5.394	8.83968	37.96032	5.394	7.73319	39.06681
5.395	13.56309	33.23691	5.395	11.67745	35.12255	5.395	10.13666	36.66334	5.395	8.84508	37.95492	5.395	7.739	39.061
5.396	13.56673	33.23327	5.396	11.68179	35.11821	5.396	10.14157	36.65843	5.396	8.85047	37.94953	5.396	7.7448	39.0552
5.397	13.57038	33.22962	5.397	11.68613	35.11387	5.397	10.14649	36.65351	5.397	8.85586	37.94414	5.397	7.7506	39.0494

5.398	13.57402	33.22598	5.398	11.69047	35.10953	5.398	10.1514	36.6486	5.398	8.86125	37.93875	5.398	7.7564	39.0436
5.399	13.57766	33.22234	5.399	11.69481	35.10519	5.399	10.1563	36.6437	5.399	8.86663	37.93337	5.399	7.76219	39.03781
5.4	13.5813	33.2187	5.4	11.69915	35.10085	5.4	10.16121	36.63879	5.4	8.87202	37.92798	5.4	7.76798	39.03202
5.401	13.58493	33.21507	5.401	11.70348	35.09652	5.401	10.16611	36.63389	5.401	8.8774	37.9226	5.401	7.77377	39.02623
5.402	13.58856	33.21144	5.402	11.70781	35.09219	5.402	10.17101	36.62899	5.402	8.88277	37.91723	5.402	7.77956	39.02044
5.403	13.5922	33.2078	5.403	11.71214	35.08786	5.403	10.17591	36.62409	5.403	8.88815	37.91185	5.403	7.78534	39.01466
5.404	13.59583	33.20417	5.404	11.71646	35.08354	5.404	10.1808	36.6192	5.404	8.89352	37.90648	5.404	7.79112	39.00888
5.405	13.59945	33.20055	5.405	11.72078	35.07922	5.405	10.18569	36.61431	5.405	8.89888	37.90112	5.405	7.79689	39.00311
5.406	13.60308	33.19692	5.406	11.7251	35.0749	5.406	10.19058	36.60942	5.406	8.90425	37.89575	5.406	7.80266	38.99734
5.407	13.6067	33.1933	5.407	11.72942	35.07058	5.407	10.19546	36.60454	5.407	8.90961	37.89039	5.407	7.80843	38.99157
5.408	13.61032	33.18968	5.408	11.73374	35.06626	5.408	10.20035	36.59965	5.408	8.91497	37.88503	5.408	7.8142	38.9858
5.409	13.61394	33.18606	5.409	11.73805	35.06195	5.409	10.20523	36.59477	5.409	8.92032	37.87968	5.409	7.81996	38.98004
5.41	13.61756	33.18244	5.41	11.74236	35.05764	5.41	10.21011	36.58989	5.41	8.92567	37.87433	5.41	7.82572	38.97428
5.411	13.62118	33.17882	5.411	11.74667	35.05333	5.411	10.21498	36.58502	5.411	8.93102	37.86898	5.411	7.83148	38.96852
5.412	13.62479	33.17521	5.412	11.75098	35.04902	5.412	10.21985	36.58015	5.412	8.93637	37.86363	5.412	7.83723	38.96277
5.413	13.6284	33.1716	5.413	11.75528	35.04472	5.413	10.22472	36.57528	5.413	8.94171	37.85829	5.413	7.84298	38.95702
5.414	13.63201	33.16799	5.414	11.75958	35.04042	5.414	10.22959	36.57041	5.414	8.94705	37.85295	5.414	7.84872	38.95128
5.415	13.63562	33.16438	5.415	11.76388	35.03612	5.415	10.23445	36.56555	5.415	8.95239	37.84761	5.415	7.85447	38.94553
5.416	13.63922	33.16078	5.416	11.76818	35.03182	5.416	10.23931	36.56069	5.416	8.95773	37.84227	5.416	7.86021	38.93979
5.417	13.64283	33.15717	5.417	11.77247	35.02753	5.417	10.24417	36.55583	5.417	8.96306	37.83694	5.417	7.86595	38.93405
5.418	13.64643	33.15357	5.418	11.77676	35.02324	5.418	10.24903	36.55097	5.418	8.96839	37.83161	5.418	7.87168	38.92832
5.419	13.65003	33.14997	5.419	11.78105	35.01895	5.419	10.25388	36.54612	5.419	8.97371	37.82629	5.419	7.87741	38.92259
5.42	13.65362	33.14638	5.42	11.78534	35.01466	5.42	10.25873	36.54127	5.42	8.97904	37.82096	5.42	7.88314	38.91686
5.421	13.65722	33.14278	5.421	11.78962	35.01038	5.421	10.26358	36.53642	5.421	8.98436	37.81564	5.421	7.88886	38.91114
5.422	13.66081	33.13919	5.422	11.79391	35.00609	5.422	10.26842	36.53158	5.422	8.98967	37.81033	5.422	7.89458	38.90542
5.423	13.6644	33.1356	5.423	11.79819	35.00181	5.423	10.27327	36.52673	5.423	8.99499	37.80501	5.423	7.9003	38.8997
5.424	13.66799	33.13201	5.424	11.80246	34.99754	5.424	10.27811	36.52189	5.424	9.0003	37.7997	5.424	7.90601	38.89399
5.425	13.67158	33.12842	5.425	11.80674	34.99326	5.425	10.28294	36.51706	5.425	9.00561	37.79439	5.425	7.91173	38.88827
5.426	13.67517	33.12483	5.426	11.81101	34.98899	5.426	10.28778	36.51222	5.426	9.01091	37.78909	5.426	7.91743	38.88257
5.427	13.67875	33.12125	5.427	11.81528	34.98472	5.427	10.29261	36.50739	5.427	9.01621	37.78379	5.427	7.92314	38.87686
5.428	13.68233	33.11767	5.428	11.81955	34.98045	5.428	10.29744	36.50256	5.428	9.02151	37.77849	5.428	7.92884	38.87116
5.429	13.68591	33.11409	5.429	11.82382	34.97618	5.429	10.30227	36.49773	5.429	9.02681	37.77319	5.429	7.93454	38.86546
5.43	13.68949	33.11051	5.43	11.82808	34.97192	5.43	10.30709	36.49291	5.43	9.0321	37.7679	5.43	7.94024	38.85976
5.431	13.69306	33.10694	5.431	11.83234	34.96766	5.431	10.31191	36.48809	5.431	9.03739	37.76261	5.431	7.94593	38.85407
5.432	13.69664	33.10336	5.432	11.8366	34.9634	5.432	10.31673	36.48327	5.432	9.04268	37.75732	5.432	7.95162	38.84838
5.433	13.70021	33.09979	5.433	11.84086	34.95914	5.433	10.32154	36.47846	5.433	9.04797	37.75203	5.433	7.9573	38.8427
5.434	13.70378	33.09622	5.434	11.84511	34.95489	5.434	10.32636	36.47364	5.434	9.05325	37.74675	5.434	7.96299	38.83701
5.435	13.70735	33.09265	5.435	11.84936	34.95064	5.435	10.33117	36.46883	5.435	9.05853	37.74147	5.435	7.96867	38.83133
5.436	13.71091	33.08909	5.436	11.85361	34.94639	5.436	10.33598	36.46402	5.436	9.0638	37.7362	5.436	7.97435	38.82565
5.437	13.71448	33.08552	5.437	11.85786	34.94214	5.437	10.34078	36.45922	5.437	9.06908	37.73092	5.437	7.98002	38.81998
5.438	13.71804	33.08196	5.438	11.8621	34.9379	5.438	10.34558	36.45442	5.438	9.07435	37.72565	5.438	7.98569	38.81431
5.439	13.7216	33.0784	5.439	11.86634	34.93366	5.439	10.35038	36.44962	5.439	9.07961	37.72039	5.439	7.99136	38.80864
5.44	13.72516	33.07484	5.44	11.87058	34.92942	5.44	10.35518	36.44482	5.44	9.08488	37.71512	5.44	7.99702	38.80298
5.441	13.72871	33.07129	5.441	11.87482	34.92518	5.441	10.35998	36.44002	5.441	9.09014	37.70986	5.441	8.00268	38.79732
5.442	13.73227	33.06773	5.442	11.87906	34.92094	5.442	10.36477	36.43523	5.442	9.0954	37.7046	5.442	8.00834	38.79166
5.443	13.73582	33.06418	5.443	11.88329	34.91671	5.443	10.36956	36.43044	5.443	9.10065	37.69935	5.443	8.014	38.786
5.444	13.73937	33.06063	5.444	11.88752	34.91248	5.444	10.37434	36.42566	5.444	9.10591	37.69409	5.444	8.01965	38.78035
5.445	13.74292	33.05708	5.445	11.89175	34.90825	5.445	10.37913	36.42087	5.445	9.11116	37.68884	5.445	8.0253	38.7747
5.446	13.74646	33.05354	5.446	11.89598	34.90402	5.446	10.38391	36.41609	5.446	9.1164	37.6836	5.446	8.03094	38.76906
5.447	13.75001	33.04999	5.447	11.9002	34.8998	5.447	10.38869	36.41131	5.447	9.12165	37.67835	5.447	8.03659	38.76341
5.448	13.75355	33.04645	5.448	11.90442	34.89558	5.448	10.39346	36.40654	5.448	9.12689	37.67311	5.448	8.04222	38.75778
5.449	13.75709	33.04291	5.449	11.90864	34.89136	5.449	10.39824	36.40176	5.449	9.13213	37.66787	5.449	8.04786	38.75214
5.45	13.76063	33.03937	5.45	11.91286	34.88714	5.45	10.40301	36.39699	5.45	9.13736	37.66264	5.45	8.05349	38.74651
5.451	13.76416	33.03584	5.451	11.91707	34.88293	5.451	10.40778	36.39222	5.451	9.14259	37.65741	5.451	8.05912	38.74088
5.452	13.7677	33.0323	5.452	11.92128	34.87872	5.452	10.41254	36.38746	5.452	9.14782	37.65218	5.452	8.06475	38.73525
5.453	13.77123	33.02877	5.453	11.92549	34.87451	5.453	10.4173	36.3827	5.453	9.15305	37.64695	5.453	8.07038	38.72962
5.454	13.77476	33.02524	5.454	11.9297	34.8703	5.454	10.42206	36.37794	5.454	9.15828	37.64172	5.454	8.076	38.724
5.455	13.77829	33.02171	5.455	11.9339	34.8661	5.455	10.42682	36.37318	5.455	9.1635	37.6365	5.455	8.08161	38.71839
5.456	13.78182	33.01818	5.456	11.93811	34.86189	5.456	10.43158	36.36842	5.456	9.16871	37.63129	5.456	8.08723	38.71277
5.457	13.78534	33.01466	5.457	11.94231	34.85769	5.457	10.43633	36.36367	5.457	9.17393	37.62607	5.457	8.09284	38.70716
5.458	13.78887	33.01113	5.458	11.94651	34.85349	5.458	10.44108	36.35892	5.458	9.17914	37.62086	5.458	8.09845	38.70155
5.459	13.79239	33.00761	5.459	11.9507	34.8493	5.459	10.44583	36.35417	5.459	9.18435	37.61565	5.459	8.10405	38.69595
5.46	13.79591	33.00409	5.46	11.95489	34.84511	5.46	10.45057	36.34943	5.46	9.18956	37.61044	5.46	8.10966	38.69034
5.461	13.79942	33.00058	5.461	11.95909	34.84091	5.461	10.45531	36.34469	5.461	9.19476	37.60524	5.461	8.11526	38.68474
5.462	13.80294	32.99706	5.462	11.96327	34.83673	5.462	10.46005	36.33995	5.462	9.19996	37.60004	5.462	8.12085	38.67915
5.463	13.80645	32.99355	5.463	11.96746	34.83254	5.463	10.46479	36.33521	5.463	9.20516	37.59484	5.463	8.12645	38.67355
5.464	13.80996	32.99004	5.464	11.97165	34.82835	5.464	10.46952	36.33048	5.464	9.21036	37.58964	5.464	8.13204	38.66796
5.465	13.81347	32.98653	5.465	11.97583	34.82417	5.465	10.47426	36.32574	5.465	9.21555	37.58445	5.465	8.13762	38.66238
5.466	13.81698	32.98302	5.466	11.98001	34.81999	5.466	10.47899	36.32101	5.466	9.22074	37.57926	5.466	8.14321	38.65679
5.467	13.82048	32.97952	5.467	11.98418	34.81582	5.467	10.48371	36.31629	5.467	9.22593	37.57407	5.467	8.14879	38.65121
5.468	13.82399	32.97601	5.468	11.98836	34.81164	5.468	10.48844	36.31156	5.468	9.23111	37.56889	5.468	8.15437	38.64563
5.469	13.82749	32.97251	5.469	11.99253	34.80747	5.469	10.49316	36.30684	5.469	9.23629	37.56371	5.469	8.15994	38.64006
5.47	13.83099	32.96901	5.47	11.9967	34.8033	5.47	10.49788	36.30212	5.47	9.24147	37.55853	5.47	8.16551	38.63449
5.471	13.83449	32.96551	5.471	12.00087	34.79913	5.471	10.50259	36.29741	5.471	9.24664	37.55336	5.471	8.17108	38.62892
5.472	13.83798	32.96202	5.472	12.00504	34.79496	5.472	10.50731	36.29269	5.472	9.25182	37.54818	5.472	8.17665	38.62335
5.473	13.84148	32.95852	5.473	12.0092	34.7908	5.473	10.51202	36.28798	5.473	9.25699	37.54301	5.473	8.18221	38.61779
5.474	13.84497	32.95503	5.474	12.01336	34.78664	5.474	10.51672	36.28328	5.474	9.26215	37.53785	5.474	8.18777	38.61223
5.475	13.84846	32.95154	5.475	12.01752	34.78248	5.475	10.52143	36.27857	5.475	9.26732	37.53268	5.475	8.19333	38.60667
5.476	13.85195	32.94805	5.476	12.02168	34.77832	5.476	10.52613	36.27387	5.476	9.27248	37.52752	5.476	8.19888	38.60112
5.477	13.85543	32.94457	5.477	12.02583	34.77417	5.477	10.53083	36.26917	5.477	9.27764	37.52236	5.477	8.20443	38.59557
5.478	13.85892	32.94108	5.478	12.02999	34.77001	5.478	10.53553	36.26447	5.478	9.28279	37.51721	5.478	8.20998	38.59002
5.479	13.8624	32.9376	5.479	12.03414	34.76586	5.479	10.54023	36.25977	5.479	9.28795	37.51205	5.479	8.21552	38.58448
5.48	13.86588	32.93412	5.48	12.03828	34.76172	5.48	10.54492	36.25508	5.48	9.2931	37.5069	5.48	8.22106	38.57894
5.481	13.86936	32.93064	5.481	12.04243	34.75757	5.481	10.54961	36.25039	5.481	9.29824	37.50176	5.481	8.2266	38.5734
5.482	13.87284	32.92716	5.482	12.04657	34.75343	5.482	10.5543	36.2457	5.482	9.30339	37.49661	5.482	8.23214	38.56786
5.483	13.87631	32.92369	5.483	12.05071	34.74929	5.483	10.55898	36.24102	5.483	9.30853	37.49147	5.483	8.23767	38.56233
5.484	13.87978	32.92022	5.484	12.05485	34.74515	5.484	10.56367	36.23633	5.484	9.31367	37.48633	5.484	8.2432	38.5568
5.485	13.88325	32.91675	5.485	12.05899	34.74101	5.485	10.56835	36.23165	5.485	9.3188	37.4812	5.485	8.24872	38.55128
5.486	13.88672	32.91328	5.486	12.06312	34.73688	5.486	10.57302	36.22698	5.486	9.32394	37.47606	5.486	8.25425	38.54575
5.487	13.89019	32.90981	5.487	12.06725	34.73275	5.487	10.5777	36.2223	5.487	9.32907	37.47093	5.487	8.25977	38.54023
5.488	13.89366	32.90634	5.488	12.07138	34.72862	5.488	10.58237	36.21763	5.488	9.33419	37.46581	5.488	8.26528	38.53472
5.489	13.89712	32.90288	5.489	12.07551	34.72449	5.489	10.58704	36.21296	5.489	9.33932	37.46068	5.489	8.2708	38.5292
5.49	13.90058	32.89942	5.49	12.07964	34.72036	5.49	10.59171	36.20829	5.49	9.34444	37.45556	5.49	8.27631	38.52369
5.491	13.90404	32.89596	5.491	12.08376	34.71624	5.491	10.59637	36.20363	5.491	9.34956	37.45044	5.491	8.28182	38.51818
5.492	13.9075	32.8925	5.492	12.08788	34.71212	5.492	10.60104	36.19896	5.492	9.35467	37.44533	5.492	8.28732	38.51268
5.493	13.91095	32.88905	5.493	12.092	34.708	5.493	10.6057	36.1943	5.493	9.35979	37.44021	5.493	8.29282	38.50718
5.494	13.91441	32.88559	5.494	12.09611	34.70389	5.494	10.61035	36.18965	5.494	9.3649	37.4351	5.494	8.29832	38.50168
5.495	13.91786	32.88214	5.495	12.10023	34.69977	5.495	10.61501	36.18499	5.495	9.37001	37.42999	5.495	8.30382	38.49618
5.496	13.92131	32.87869	5.496	12.10434	34.69566	5.496	10.61966	36.18034	5.496	9.37511	37.42489	5.496	8.30931	38.49069
5.497	13.92476	32.87524	5.497	12.10845	34.69155	5.497	10.62431	36.17569	5.497	9.38021	37.41979	5.497	8.3148	38.4852
5.498	13.92821	32.87179	5.498	12.11256	34.68744	5.498	10.62896	36.17104	5.498	9.38531	37.41469	5.498	8.32029	38.47971
5.499	13.93165	32.86835	5.499	12.11666	34.68334	5.499	10.6336	36.1664	5.499	9.39041	37.40959	5.499	8.32577	38.47423
5.5	13.93509	32.86491	5.5	12.12076	34.67924	5.5	10.63824	36.16176	5.5	9.3955	37.4045	5.5	8.33125	38.46875
5.501	13.93853	32.86147	5.501	12.12486	34.67514	5.501	10.64288	36.15712	5.501	9.4006	37.3994	5.501	8.33673	38.46327
5.502	13.94197	32.85803	5.502	12.12896	34.67104	5.502	10.64752	36.15248	5.502	9.40568	37.39432	5.502	8.34221	38.45779
5.503	13.94541	32.85459	5.503	12.13306	34.66694	5.503	10.65215	36.14785	5.503	9.41077	37.38923	5.503	8.34768	38.45232
5.504	13.94885	32.85115	5.504	12.13715	34.66285	5.504	10.65678	36.14322	5.504	9.41585	37.38415	5.504	8.35315	38.44685
5.505	13.95228	32.84772	5.505	12.14124	34.65876	5.505	10.66141	36.13859	5.505	9.42093	37.37907	5.505	8.35861	38.44139
5.506	13.95571	32.84429	5.506	12.14533	34.65467	5.506	10.66604	36.13396	5.506	9.42601	37.37399	5.506	8.36408	38.43592
5.507	13.95914	32.84086	5.507	12.14942	34.65058	5.507	10.67067	36.12933	5.507	9.43108	37.36892	5.507	8.36954	38.43046
5.508	13.96257	32.83743	5.508	12.1535	34.6465	5.508	10.67529	36.12471	5.508	9.43616	37.36384	5.508	8.37499	38.42501
5.509	13.96599	32.83401	5.509	12.15759	34.64241	5.509	10.67991	36.12009	5.509	9.44122	37.35878	5.509	8.38045	38.41955
5.51	13.96942	32.83058	5.51	12.16167	34.63833	5.51	10.68452	36.11548	5.51	9.44629	37.35371	5.51	8.3859	38.4141
5.511	13.97284	32.82716	5.511	12.16575	34.63425	5.511	10.68914	36.11086	5.511	9.45135	37.34865	5.511	8.39135	38.40865
5.512	13.97626	32.82374	5.512	12.16982	34.63018	5.512	10.69375	36.10625	5.512	9.45642	37.34358	5.512	8.39679	38.40321
5.513	13.97968	32.82032	5.513	12.1739	34.6261	5.513	10.69836	36.10164	5.513	9.46147	37.33853	5.513	8.40224	38.39776
5.514	13.98309	32.81691	5.514	12.17797	34.62203	5.514	10.70296	36.09704	5.514	9.46653	37.33347	5.514	8.40768	38.39232
5.515	13.98651	32.81349	5.515	12.18204	34.61796	5.515	10.70757	36.09243	5.515	9.47158	37.32842	5.515	8.41311	38.38689
5.516	13.98992	32.81008	5.516	12.1861	34.6139	5.516	10.71217	36.08783	5.516	9.47663	37.32337	5.516	8.41855	38.38145
5.517	13.99333	32.80667	5.517	12.19017	34.60983	5.517	10.71677	36.08323	5.517	9.48168	37.31832	5.517	8.42398	38.37602
5.518	13.99674	32.80326	5.518	12.19423	34.60577	5.518	10.72137	36.07863	5.518	9.48672	37.31328	5.518	8.4294	38.3706
5.519	14.00015	32.79985	5.519	12.19829	34.60171	5.519	10.72596	36.07404	5.519	9.49176	37.30824	5.519	8.43483	38.36517
5.52	14.00356	32.79644	5.52	12.20235	34.59765	5.52	10.73055	36.06945	5.52	9.4968	37.3032	5.52	8.44025	38.35975
5.521	14.00696	32.79304	5.521	12.20641	34.59359	5.521	10.73514	36.06486	5.521	9.50184	37.29816	5.521	8.44567	38.35433
5.522	14.01036	32.78964	5.522	12.21046	34.58954	5.522	10.73973	36.06027	5.522	9.50687	37.29313	5.522	8.45108	38.34892
5.523	14.01376	32.78624	5.523	12.21451	34.58549	5.523	10.74431	36.05569	5.523	9.5119	37.2881	5.523	8.4565	38.3435
5.524	14.01716	32.78284	5.524	12.21856	34.58144	5.524	10.74889	36.05111	5.524	9.51693	37.28307	5.524	8.46191	38.33809
5.525	14.02055	32.77945	5.525	12.22261	34.57739	5.525	10.75347	36.04653	5.525	9.52195	37.27805	5.525	8.46731	38.33269
5.526	14.02395	32.77605	5.526	12.22665	34.57335	5.526	10.75805	36.04195	5.526	9.52698	37.27302	5.526	8.47272	38.32728
5.527	14.02734	32.77266	5.527	12.23069	34.56931	5.527	10.76262	36.03738	5.527	9.532	37.268	5.527	8.47812	38.32188
5.528	14.03073	32.76927	5.528	12.23474	34.56526	5.528	10.76719	36.03281	5.528	9.53701	37.26299	5.528	8.48352	38.31648
5.529	14.03412	32.76588	5.529	12.23877	34.56123	5.529	10.77176	36.02824	5.529	9.54203	37.25797	5.529	8.48891	38.31109
5.53	14.03751	32.76249	5.53	12.24281	34.55719	5.53	10.77633	36.02367	5.53	9.54704	37.25296	5.53	8.4943	38.3057
5.531	14.04089	32.75911	5.531	12.24684	34.55316	5.531	10.7809	36.0191	5.531	9.55205	37.24795	5.531	8.49969	38.30031
5.532	14.04428	32.75572	5.532	12.25088	34.54912	5.532	10.78546	36.01454	5.532	9.55705	37.24295	5.532	8.50508	38.29492
5.533	14.04766	32.75234	5.533	12.25491	34.54509	5.533	10.79002	36.00998	5.533	9.56206	37.23794	5.533	8.51046	38.28954
5.534	14.05104	32.74896	5.534	12.25893	34.54107	5.534	10.79457	36.00543	5.534	9.56706	37.23294	5.534	8.51584	38.28416
5.535	14.05442	32.74558	5.535	12.26296	34.53704	5.535	10.79913	36.00087	5.535	9.57206	37.22794	5.535	8.52122	38.27878
5.536	14.05779	32.74221	5.536	12.26698	34.53302	5.536	10.80368	35.99632	5.536	9.57705	37.22295	5.536	8.5266	38.2734
5.537	14.06117	32.73883	5.537	12.271	34.529	5.537	10.80823	35.99177	5.537	9.58204	37.21796	5.537	8.53197	38.26803
5.538	14.06454	32.73546	5.538	12.27502	34.52498	5.538	10.81278	35.98722	5.538	9.58703	37.21297	5.538	8.53734	38.26266
5.539	14.06791	32.73209	5.539	12.27904	34.52096	5.539	10.81732	35.98268	5.539	9.59202	37.20798	5.539	8.5427	38.2573
5.54	14.07128	32.72872	5.54	12.28305	34.51695	5.54	10.82186	35.97814	5.54	9.59701	37.20299	5.54	8.54807	38.25193
5.541	14.07464	32.72536	5.541	12.28706	34.51294	5.541	10.8264	35.9736	5.541	9.60199	37.19801	5.541	8.55343	38.24657
5.542	14.07801	32.72199	5.542	12.29107	34.50893	5.542	10.83094	35.96906	5.542	9.60697	37.19303	5.542	8.55878	38.24122
5.543	14.08137	32.71863	5.543	12.29508	34.50492	5.543	10.83547	35.96453	5.543	9.61194	37.18806	5.543	8.56414	38.23586
5.544	14.08473	32.71527	5.544	12.29909	34.50091	5.544	10.84001	35.95999	5.544	9.61692	37.18308	5.544	8.56949	38.23051
5.545	14.08809	32.71191	5.545	12.30309	34.49691	5.545	10.84454	35.95546	5.545	9.62189	37.17811	5.545	8.57484	38.22516
5.546	14.09145	32.70855	5.546	12.30709	34.49291	5.546	10.84906	35.95094	5.546	9.62685	37.17315	5.546	8.58019	38.21981
5.547	14.09481	32.70519	5.547	12.31109	34.48891	5.547	10.85359	35.94641	5.547	9.63182	37.16818	5.547	8.58553	38.21447
5.548	14.09816	32.70184	5.548	12.31509	34.48491	5.548	10.85811	35.94189	5.548	9.63678	37.16322	5.548	8.59087	38.20913
5.549	14.10151	32.69849	5.549	12.31908	34.48092	5.549	10.86263	35.93737	5.549	9.64174	37.15826	5.549	8.59621	38.20379
5.55	14.10486	32.69514	5.55	12.32308	34.47692	5.55	10.86715	35.93285	5.55	9.6467	37.1533	5.55	8.60154	38.19846
5.551	14.10821	32.69179	5.551	12.32707	34.47293	5.551	10.87166	35.92834	5.551	9.65166	37.14834	5.551	8.60687	38.19313
5.552	14.11156	32.68844	5.552	12.33106	34.46894	5.552	10.87618	35.92382	5.552	9.65661	37.14339	5.552	8.6122	38.1878
5.553	14.1149	32.6851	5.553	12.33504	34.46496	5.553	10.88069	35.91931	5.553	9.66156	37.13844	5.553	8.61752	38.18248
5.554	14.11825	32.68175	5.554	12.33903	34.46097	5.554	10.88519	35.91481	5.554	9.6665	37.1335	5.554	8.62285	38.17715
5.555	14.12159	32.67841	5.555	12.34301	34.45699	5.555	10.8897	35.9103	5.555	9.67145	37.12855	5.555	8.62817	38.17183
5.556	14.12493	32.67507	5.556	12.34699	34.45301	5.556	10.8942	35.9058	5.556	9.67639	37.12361	5.556	8.63348	38.16652
5.557	14.12827	32.67173	5.557	12.35097	34.44903	5.557	10.8987	35.9013	5.557	9.68133	37.11867	5.557	8.6388	38.1612

5.558	14.1316	32.6684	5.558	12.35494	34.44506	5.558	10.9032	35.8968	5.558	9.68626	37.11374	5.558	8.64411	38.15589
5.559	14.13494	32.66506	5.559	12.35892	34.44108	5.559	10.9077	35.8923	5.559	9.6912	37.1088	5.559	8.64942	38.15058
5.56	14.13827	32.66173	5.56	12.36289	34.43711	5.56	10.91219	35.88781	5.56	9.69613	37.10387	5.56	8.65472	38.14528
5.561	14.1416	32.6584	5.561	12.36686	34.43314	5.561	10.91668	35.88332	5.561	9.70106	37.09894	5.561	8.66003	38.13997
5.562	14.14493	32.65507	5.562	12.37082	34.42918	5.562	10.92117	35.87883	5.562	9.70598	37.09402	5.562	8.66533	38.13467
5.563	14.14826	32.65174	5.563	12.37479	34.42521	5.563	10.92566	35.87434	5.563	9.71091	37.08909	5.563	8.67062	38.12938
5.564	14.15158	32.64842	5.564	12.37875	34.42125	5.564	10.93014	35.86986	5.564	9.71583	37.08417	5.564	8.67592	38.12408
5.565	14.1549	32.6451	5.565	12.38271	34.41729	5.565	10.93462	35.86538	5.565	9.72074	37.07926	5.565	8.68121	38.11879
5.566	14.15823	32.64177	5.566	12.38667	34.41333	5.566	10.9391	35.8609	5.566	9.72566	37.07434	5.566	8.6865	38.1135
5.567	14.16155	32.63845	5.567	12.39063	34.40937	5.567	10.94357	35.85643	5.567	9.73057	37.06943	5.567	8.69178	38.10822
5.568	14.16486	32.63514	5.568	12.39458	34.40542	5.568	10.94805	35.85195	5.568	9.73548	37.06452	5.568	8.69706	38.10294
5.569	14.16818	32.63182	5.569	12.39853	34.40147	5.569	10.95252	35.84748	5.569	9.74039	37.05961	5.569	8.70234	38.09766
5.57	14.1715	32.6285	5.57	12.40248	34.39752	5.57	10.95699	35.84301	5.57	9.74529	37.05471	5.57	8.70762	38.09238
5.571	14.17481	32.62519	5.571	12.40643	34.39357	5.571	10.96146	35.83854	5.571	9.75019	37.04981	5.571	8.7129	38.0871
5.572	14.17812	32.62188	5.572	12.41038	34.38962	5.572	10.96592	35.83408	5.572	9.75509	37.04491	5.572	8.71817	38.08183
5.573	14.18143	32.61857	5.573	12.41432	34.38568	5.573	10.97038	35.82962	5.573	9.75999	37.04001	5.573	8.72344	38.07656
5.574	14.18474	32.61526	5.574	12.41826	34.38174	5.574	10.97484	35.82516	5.574	9.76488	37.03512	5.574	8.7287	38.0713
5.575	14.18804	32.61196	5.575	12.4222	34.3778	5.575	10.9793	35.8207	5.575	9.76977	37.03023	5.575	8.73396	38.06604
5.576	14.19135	32.60865	5.576	12.42614	34.37386	5.576	10.98375	35.81625	5.576	9.77466	37.02534	5.576	8.73922	38.06078
5.577	14.19465	32.60535	5.577	12.43007	34.36993	5.577	10.98821	35.81179	5.577	9.77955	37.02045	5.577	8.74448	38.05552
5.578	14.19795	32.60205	5.578	12.434	34.366	5.578	10.99266	35.80734	5.578	9.78443	37.01557	5.578	8.74973	38.05027
5.579	14.20125	32.59875	5.579	12.43794	34.36206	5.579	10.9971	35.8029	5.579	9.78931	37.01069	5.579	8.75499	38.04501
5.58	14.20454	32.59546	5.58	12.44186	34.35814	5.58	11.00155	35.79845	5.58	9.79419	37.00581	5.58	8.76023	38.03977
5.581	14.20784	32.59216	5.581	12.44579	34.35421	5.581	11.00599	35.79401	5.581	9.79906	37.00094	5.581	8.76548	38.03452
5.582	14.21113	32.58887	5.582	12.44972	34.35028	5.582	11.01043	35.78957	5.582	9.80394	36.99606	5.582	8.77072	38.02928
5.583	14.21442	32.58558	5.583	12.45364	34.34636	5.583	11.01487	35.78513	5.583	9.80881	36.99119	5.583	8.77596	38.02404
5.584	14.21771	32.58229	5.584	12.45756	34.34244	5.584	11.0193	35.7807	5.584	9.81367	36.98633	5.584	8.7812	38.0188
5.585	14.221	32.579	5.585	12.46148	34.33852	5.585	11.02374	35.77626	5.585	9.81854	36.98146	5.585	8.78644	38.01356
5.586	14.22429	32.57571	5.586	12.46539	34.33461	5.586	11.02817	35.77183	5.586	9.8234	36.9766	5.586	8.79167	38.00833
5.587	14.22757	32.57243	5.587	12.46931	34.33069	5.587	11.0326	35.7674	5.587	9.82826	36.97174	5.587	8.7969	38.0031
5.588	14.23085	32.56915	5.588	12.47322	34.32678	5.588	11.03702	35.76298	5.588	9.83312	36.96688	5.588	8.80212	37.99788
5.589	14.23413	32.56587	5.589	12.47713	34.32287	5.589	11.04145	35.75855	5.589	9.83797	36.96203	5.589	8.80735	37.99265
5.59	14.23741	32.56259	5.59	12.48104	34.31896	5.59	11.04587	35.75413	5.59	9.84282	36.95718	5.59	8.81257	37.98743
5.591	14.24069	32.55931	5.591	12.48494	34.31506	5.591	11.05029	35.74971	5.591	9.84767	36.95233	5.591	8.81778	37.98222
5.592	14.24397	32.55603	5.592	12.48884	34.31116	5.592	11.0547	35.7453	5.592	9.85252	36.94748	5.592	8.823	37.977
5.593	14.24724	32.55276	5.593	12.49275	34.30725	5.593	11.05912	35.74088	5.593	9.85736	36.94264	5.593	8.82821	37.97179
5.594	14.25051	32.54949	5.594	12.49664	34.30336	5.594	11.06353	35.73647	5.594	9.86221	36.93779	5.594	8.83342	37.96658
5.595	14.25378	32.54622	5.595	12.50054	34.29946	5.595	11.06794	35.73206	5.595	9.86704	36.93296	5.595	8.83863	37.96137
5.596	14.25705	32.54295	5.596	12.50444	34.29556	5.596	11.07235	35.72765	5.596	9.87188	36.92812	5.596	8.84383	37.95617
5.597	14.26032	32.53968	5.597	12.50833	34.29167	5.597	11.07675	35.72325	5.597	9.87671	36.92329	5.597	8.84903	37.95097
5.598	14.26358	32.53642	5.598	12.51222	34.28778	5.598	11.08115	35.71885	5.598	9.88154	36.91846	5.598	8.85423	37.94577
5.599	14.26685	32.53315	5.599	12.51611	34.28389	5.599	11.08555	35.71445	5.599	9.88637	36.91363	5.599	8.85943	37.94057
5.6	14.27011	32.52989	5.6	12.52	34.28	5.6	11.08995	35.71005	5.6	9.8912	36.9088	5.6	8.86462	37.93538
5.601	14.27337	32.52663	5.601	12.52388	34.27612	5.601	11.09435	35.70565	5.601	9.89602	36.90398	5.601	8.86981	37.93019
5.602	14.27663	32.52337	5.602	12.52776	34.27224	5.602	11.09874	35.70126	5.602	9.90084	36.89916	5.602	8.87499	37.92501
5.603	14.27988	32.52012	5.603	12.53164	34.26836	5.603	11.10313	35.69687	5.603	9.90566	36.89434	5.603	8.88018	37.91982
5.604	14.28314	32.51686	5.604	12.53552	34.26448	5.604	11.10752	35.69248	5.604	9.91048	36.88952	5.604	8.88536	37.91464
5.605	14.28639	32.51361	5.605	12.5394	34.2606	5.605	11.1119	35.6881	5.605	9.91529	36.88471	5.605	8.89054	37.90946
5.606	14.28964	32.51036	5.606	12.54327	34.25673	5.606	11.11629	35.68371	5.606	9.9201	36.8799	5.606	8.89572	37.90428
5.607	14.29289	32.50711	5.607	12.54715	34.25285	5.607	11.12067	35.67933	5.607	9.92491	36.87509	5.607	8.90089	37.89911
5.608	14.29614	32.50386	5.608	12.55102	34.24898	5.608	11.12505	35.67495	5.608	9.92971	36.87029	5.608	8.90606	37.89394
5.609	14.29938	32.50062	5.609	12.55488	34.24512	5.609	11.12942	35.67058	5.609	9.93452	36.86548	5.609	8.91123	37.88877
5.61	14.30263	32.49737	5.61	12.55875	34.24125	5.61	11.1338	35.6662	5.61	9.93932	36.86068	5.61	8.91639	37.88361
5.611	14.30587	32.49413	5.611	12.56261	34.23739	5.611	11.13817	35.66183	5.611	9.94411	36.85589	5.611	8.92155	37.87845
5.612	14.30911	32.49089	5.612	12.56648	34.23352	5.612	11.14254	35.65746	5.612	9.94891	36.85109	5.612	8.92671	37.87329
5.613	14.31235	32.48765	5.613	12.57034	34.22966	5.613	11.14691	35.65309	5.613	9.9537	36.8463	5.613	8.93187	37.86813
5.614	14.31559	32.48441	5.614	12.57419	34.22581	5.614	11.15127	35.64873	5.614	9.95849	36.84151	5.614	8.93702	37.86298
5.615	14.31882	32.48118	5.615	12.57805	34.22195	5.615	11.15563	35.64437	5.615	9.96328	36.83672	5.615	8.94217	37.85783
5.616	14.32206	32.47794	5.616	12.5819	34.2181	5.616	11.15999	35.64001	5.616	9.96806	36.83194	5.616	8.94732	37.85268
5.617	14.32529	32.47471	5.617	12.58575	34.21425	5.617	11.16435	35.63565	5.617	9.97284	36.82716	5.617	8.95247	37.84753
5.618	14.32852	32.47148	5.618	12.5896	34.2104	5.618	11.16871	35.63129	5.618	9.97762	36.82238	5.618	8.95761	37.84239
5.619	14.33175	32.46825	5.619	12.59345	34.20655	5.619	11.17306	35.62694	5.619	9.9824	36.8176	5.619	8.96275	37.83725
5.62	14.33497	32.46503	5.62	12.5973	34.2027	5.62	11.17741	35.62259	5.62	9.98718	36.81282	5.62	8.96789	37.83211
5.621	14.3382	32.4618	5.621	12.60114	34.19886	5.621	11.18176	35.61824	5.621	9.99195	36.80805	5.621	8.97302	37.82698
5.622	14.34142	32.45858	5.622	12.60498	34.19502	5.622	11.18611	35.61389	5.622	9.99672	36.80328	5.622	8.97816	37.82184
5.623	14.34464	32.45536	5.623	12.60882	34.19118	5.623	11.19045	35.60955	5.623	10.00148	36.79852	5.623	8.98328	37.81672
5.624	14.34786	32.45214	5.624	12.61266	34.18734	5.624	11.19479	35.60521	5.624	10.00625	36.79375	5.624	8.98841	37.81159
5.625	14.35108	32.44892	5.625	12.61649	34.18351	5.625	11.19913	35.60087	5.625	10.01101	36.78899	5.625	8.99353	37.80647
5.626	14.3543	32.4457	5.626	12.62033	34.17967	5.626	11.20347	35.59653	5.626	10.01577	36.78423	5.626	8.99866	37.80134
5.627	14.35751	32.44249	5.627	12.62416	34.17584	5.627	11.2078	35.5922	5.627	10.02053	36.77947	5.627	9.00377	37.79623
5.628	14.36073	32.43927	5.628	12.62799	34.17201	5.628	11.21213	35.58787	5.628	10.02528	36.77472	5.628	9.00889	37.79111
5.629	14.36394	32.43606	5.629	12.63181	34.16819	5.629	11.21646	35.58354	5.629	10.03003	36.76997	5.629	9.014	37.786
5.63	14.36715	32.43285	5.63	12.63564	34.16436	5.63	11.22079	35.57921	5.63	10.03478	36.76522	5.63	9.01911	37.78089
5.631	14.37036	32.42964	5.631	12.63946	34.16054	5.631	11.22512	35.57488	5.631	10.03953	36.76047	5.631	9.02422	37.77578
5.632	14.37356	32.42644	5.632	12.64328	34.15672	5.632	11.22944	35.57056	5.632	10.04427	36.75573	5.632	9.02932	37.77068
5.633	14.37677	32.42323	5.633	12.6471	34.1529	5.633	11.23376	35.56624	5.633	10.04901	36.75099	5.633	9.03443	37.76557
5.634	14.37997	32.42003	5.634	12.65092	34.14908	5.634	11.23808	35.56192	5.634	10.05375	36.74625	5.634	9.03952	37.76048
5.635	14.38317	32.41683	5.635	12.65473	34.14527	5.635	11.2424	35.5576	5.635	10.05849	36.74151	5.635	9.04462	37.75538
5.636	14.38637	32.41363	5.636	12.65854	34.14146	5.636	11.24671	35.55329	5.636	10.06322	36.73678	5.636	9.04971	37.75029
5.637	14.38957	32.41043	5.637	12.66236	34.13764	5.637	11.25102	35.54898	5.637	10.06795	36.73205	5.637	9.05481	37.74519
5.638	14.39276	32.40724	5.638	12.66616	34.13384	5.638	11.25533	35.54467	5.638	10.07268	36.72732	5.638	9.05989	37.74011
5.639	14.39596	32.40404	5.639	12.66997	34.13003	5.639	11.25964	35.54036	5.639	10.07741	36.72259	5.639	9.06498	37.73502
5.64	14.39915	32.40085	5.64	12.67378	34.12622	5.64	11.26394	35.53606	5.64	10.08213	36.71787	5.64	9.07006	37.72994
5.641	14.40234	32.39766	5.641	12.67758	34.12242	5.641	11.26824	35.53176	5.641	10.08686	36.71314	5.641	9.07514	37.72486
5.642	14.40553	32.39447	5.642	12.68138	34.11862	5.642	11.27254	35.52746	5.642	10.09157	36.70843	5.642	9.08022	37.71978
5.643	14.40872	32.39128	5.643	12.68518	34.11482	5.643	11.27684	35.52316	5.643	10.09629	36.70371	5.643	9.08529	37.71471
5.644	14.4119	32.3881	5.644	12.68897	34.11103	5.644	11.28114	35.51886	5.644	10.101	36.699	5.644	9.09037	37.70963
5.645	14.41509	32.38491	5.645	12.69277	34.10723	5.645	11.28543	35.51457	5.645	10.10572	36.69428	5.645	9.09544	37.70456
5.646	14.41827	32.38173	5.646	12.69656	34.10344	5.646	11.28972	35.51028	5.646	10.11042	36.68958	5.646	9.1005	37.6995
5.647	14.42145	32.37855	5.647	12.70035	34.09965	5.647	11.29401	35.50599	5.647	10.11513	36.68487	5.647	9.10557	37.69443
5.648	14.42463	32.37537	5.648	12.70414	34.09586	5.648	11.2983	35.5017	5.648	10.11983	36.68017	5.648	9.11063	37.68937
5.649	14.42781	32.37219	5.649	12.70793	34.09207	5.649	11.30258	35.49742	5.649	10.12454	36.67546	5.649	9.11569	37.68431
5.65	14.43098	32.36902	5.65	12.71171	34.08829	5.65	11.30686	35.49314	5.65	10.12923	36.67077	5.65	9.12074	37.67926
5.651	14.43416	32.36584	5.651	12.71549	34.08451	5.651	11.31114	35.48886	5.651	10.13393	36.66607	5.651	9.1258	37.6742
5.652	14.43733	32.36267	5.652	12.71927	34.08073	5.652	11.31542	35.48458	5.652	10.13863	36.66137	5.652	9.13085	37.66915
5.653	14.4405	32.3595	5.653	12.72305	34.07695	5.653	11.3197	35.4803	5.653	10.14332	36.65668	5.653	9.13589	37.66411
5.654	14.44367	32.35633	5.654	12.72683	34.07317	5.654	11.32397	35.47603	5.654	10.14801	36.65199	5.654	9.14094	37.65906
5.655	14.44684	32.35316	5.655	12.7306	34.0694	5.655	11.32824	35.47176	5.655	10.15269	36.64731	5.655	9.14598	37.65402
5.656	14.45	32.35	5.656	12.73438	34.06562	5.656	11.33251	35.46749	5.656	10.15738	36.64262	5.656	9.15102	37.64898
5.657	14.45317	32.34683	5.657	12.73815	34.06185	5.657	11.33677	35.46323	5.657	10.16206	36.63794	5.657	9.15606	37.64394
5.658	14.45633	32.34367	5.658	12.74191	34.05809	5.658	11.34104	35.45896	5.658	10.16674	36.63326	5.658	9.16109	37.63891
5.659	14.45949	32.34051	5.659	12.74568	34.05432	5.659	11.3453	35.4547	5.659	10.17141	36.62859	5.659	9.16613	37.63387
5.66	14.46265	32.33735	5.66	12.74945	34.05055	5.66	11.34956	35.45044	5.66	10.17609	36.62391	5.66	9.17116	37.62884
5.661	14.46581	32.33419	5.661	12.75321	34.04679	5.661	11.35381	35.44619	5.661	10.18076	36.61924	5.661	9.17618	37.62382
5.662	14.46896	32.33104	5.662	12.75697	34.04303	5.662	11.35807	35.44193	5.662	10.18543	36.61457	5.662	9.18121	37.61879
5.663	14.47211	32.32789	5.663	12.76073	34.03927	5.663	11.36232	35.43768	5.663	10.19009	36.60991	5.663	9.18623	37.61377
5.664	14.47527	32.32473	5.664	12.76448	34.03552	5.664	11.36657	35.43343	5.664	10.19476	36.60524	5.664	9.19125	37.60875
5.665	14.47842	32.32158	5.665	12.76824	34.03176	5.665	11.37082	35.42918	5.665	10.19942	36.60058	5.665	9.19626	37.60374
5.666	14.48157	32.31843	5.666	12.77199	34.02801	5.666	11.37507	35.42493	5.666	10.20408	36.59592	5.666	9.20127	37.59873
5.667	14.48471	32.31529	5.667	12.77574	34.02426	5.667	11.37931	35.42069	5.667	10.20874	36.59126	5.667	9.20628	37.59372
5.668	14.48786	32.31214	5.668	12.77949	34.02051	5.668	11.38355	35.41645	5.668	10.21339	36.58661	5.668	9.21129	37.58871
5.669	14.491	32.309	5.669	12.78324	34.01676	5.669	11.38779	35.41221	5.669	10.21804	36.58196	5.669	9.2163	37.5837
5.67	14.49415	32.30585	5.67	12.78698	34.01302	5.67	11.39203	35.40797	5.67	10.22269	36.57731	5.67	9.2213	37.5787
5.671	14.49729	32.30271	5.671	12.79072	34.00928	5.671	11.39626	35.40374	5.671	10.22734	36.57266	5.671	9.2263	37.5737
5.672	14.50042	32.29958	5.672	12.79446	34.00554	5.672	11.40049	35.39951	5.672	10.23198	36.56802	5.672	9.2313	37.5687
5.673	14.50356	32.29644	5.673	12.7982	34.0018	5.673	11.40472	35.39528	5.673	10.23662	36.56338	5.673	9.23629	37.56371
5.674	14.5067	32.2933	5.674	12.80194	33.99806	5.674	11.40895	35.39105	5.674	10.24126	36.55874	5.674	9.24128	37.55872
5.675	14.50983	32.29017	5.675	12.80567	33.99433	5.675	11.41318	35.38682	5.675	10.2459	36.5541	5.675	9.24627	37.55373
5.676	14.51296	32.28704	5.676	12.80941	33.99059	5.676	11.4174	35.3826	5.676	10.25054	36.54946	5.676	9.25126	37.54874
5.677	14.51609	32.28391	5.677	12.81314	33.98686	5.677	11.42162	35.37838	5.677	10.25517	36.54483	5.677	9.25625	37.54375
5.678	14.51922	32.28078	5.678	12.81687	33.98313	5.678	11.42584	35.37416	5.678	10.2598	36.5402	5.678	9.26123	37.53877
5.679	14.52235	32.27765	5.679	12.82059	33.97941	5.679	11.43006	35.36994	5.679	10.26442	36.53558	5.679	9.26621	37.53379
5.68	14.52548	32.27452	5.68	12.82432	33.97568	5.68	11.43427	35.36573	5.68	10.26905	36.53095	5.68	9.27118	37.52882
5.681	14.5286	32.2714	5.681	12.82804	33.97196	5.681	11.43848	35.36152	5.681	10.27367	36.52633	5.681	9.27616	37.52384
5.682	14.53172	32.26828	5.682	12.83176	33.96824	5.682	11.44269	35.35731	5.682	10.27829	36.52171	5.682	9.28113	37.51887
5.683	14.53484	32.26516	5.683	12.83548	33.96452	5.683	11.4469	35.3531	5.683	10.28291	36.51709	5.683	9.28609	37.51391
5.684	14.53796	32.26204	5.684	12.8392	33.9608	5.684	11.45111	35.34889	5.684	10.28752	36.51248	5.684	9.29106	37.50894
5.685	14.54108	32.25892	5.685	12.84291	33.95709	5.685	11.45531	35.34469	5.685	10.29214	36.50786	5.685	9.29602	37.50398
5.686	14.5442	32.2558	5.686	12.84662	33.95338	5.686	11.45951	35.34049	5.686	10.29675	36.50325	5.686	9.30098	37.49902
5.687	14.54731	32.25269	5.687	12.85034	33.94966	5.687	11.46371	35.33629	5.687	10.30135	36.49865	5.687	9.30594	37.49406
5.688	14.55042	32.24958	5.688	12.85405	33.94595	5.688	11.46791	35.33209	5.688	10.30596	36.49404	5.688	9.3109	37.4891
5.689	14.55353	32.24647	5.689	12.85775	33.94225	5.689	11.4721	35.3279	5.689	10.31056	36.48944	5.689	9.31585	37.48415
5.69	14.55664	32.24336	5.69	12.86146	33.93854	5.69	11.47629	35.32371	5.69	10.31516	36.48484	5.69	9.3208	37.4792
5.691	14.55975	32.24025	5.691	12.86516	33.93484	5.691	11.48048	35.31952	5.691	10.31976	36.48024	5.691	9.32575	37.47425
5.692	14.56286	32.23714	5.692	12.86886	33.93114	5.692	11.48467	35.31533	5.692	10.32436	36.47564	5.692	9.33069	37.46931
5.693	14.56596	32.23404	5.693	12.87256	33.92744	5.693	11.48886	35.31114	5.693	10.32895	36.47105	5.693	9.33563	37.46437
5.694	14.56906	32.23094	5.694	12.87626	33.92374	5.694	11.49304	35.30696	5.694	10.33354	36.46646	5.694	9.34057	37.45943
5.695	14.57216	32.22784	5.695	12.87995	33.92005	5.695	11.49722	35.30278	5.695	10.33813	36.46187	5.695	9.34551	37.45449
5.696	14.57526	32.22474	5.696	12.88365	33.91635	5.696	11.5014	35.2986	5.696	10.34271	36.45729	5.696	9.35045	37.44955
5.697	14.57836	32.22164	5.697	12.88734	33.91266	5.697	11.50558	35.29442	5.697	10.3473	36.4527	5.697	9.35538	37.44462
5.698	14.58146	32.21854	5.698	12.89103	33.90897	5.698	11.50975	35.29025	5.698	10.35188	36.44812	5.698	9.36031	37.43969
5.699	14.58455	32.21545	5.699	12.89472	33.90528	5.699	11.51392	35.28608	5.699	10.35646	36.44354	5.699	9.36523	37.43477
5.7	14.58764	32.21236	5.7	12.8984	33.9016	5.7	11.51809	35.28191	5.7	10.36103	36.43897	5.7	9.37016	37.42984
5.701	14.59074	32.20926	5.701	12.90209	33.89791	5.701	11.52226	35.27774	5.701	10.36561	36.43439	5.701	9.37508	37.42492
5.702	14.59383	32.20617	5.702	12.90577	33.89423	5.702	11.52643	35.27357	5.702	10.37018	36.42982	5.702	9.38	37.42
5.703	14.59691	32.20309	5.703	12.90945	33.89055	5.703	11.53059	35.26941	5.703	10.37475	36.42525	5.703	9.38491	37.41509
5.704	14.6	32.2	5.704	12.91313	33.88687	5.704	11.53475	35.26525	5.704	10.37932	36.42068	5.704	9.38983	37.41017
5.705	14.60308	32.19692	5.705	12.9168	33.8832	5.705	11.53891	35.26109	5.705	10.38388	36.41612	5.705	9.39474	37.40526
5.706	14.60617	32.19383	5.706	12.92048	33.87952	5.706	11.54307	35.25693	5.706	10.38844	36.41156	5.706	9.39965	37.40035
5.707	14.60925	32.19075	5.707	12.92415	33.87585	5.707	11.54722	35.25278	5.707	10.393	36.407	5.707	9.40455	37.39545
5.708	14.61233	32.18767	5.708	12.92782	33.87218	5.708	11.55138	35.24862	5.708	10.39756	36.40244	5.708	9.40946	37.39054
5.709	14.61541	32.18459	5.709	12.93149	33.86851	5.709	11.55553	35.24447	5.709	10.40211	36.39789	5.709	9.41436	37.38564
5.71	14.61848	32.18152	5.71	12.93515	33.86485	5.71	11.55967	35.24033	5.71	10.40666	36.39334	5.71	9.41926	37.38074
5.711	14.62156	32.17844	5.711	12.93882	33.86118	5.711	11.56382	35.23618	5.711	10.41121	36.38879	5.711	9.42415	37.37585
5.712	14.62463	32.17537	5.712	12.94248	33.85752	5.712	11.56796	35.23204	5.712	10.41576	36.38424	5.712	9.42904	37.37096
5.713	14.6277	32.1723	5.713	12.94614	33.85386	5.713	11.57211	35.22789	5.713	10.42031	36.37969	5.713	9.43394	37.36606
5.714	14.63077	32.16923	5.714	12.9498	33.8502	5.714	11.57625	35.22375	5.714	10.42485	36.37515	5.714	9.43882	37.36118
5.715	14.63384	32.16616	5.715	12.95346	33.84654	5.715	11.58038	35.21962	5.715	10.42939	36.37061	5.715	9.44371	37.35629
5.716	14.63691	32.16309	5.716	12.95711	33.84289	5.716	11.58452	35.21548	5.716	10.43393	36.36607	5.716	9.44859	37.35141
5.717	14.63998	32.16002	5.717	12.96076	33.83924	5.717	11.58865	35.21135	5.717	10.43846	36.36154	5.717	9.45347	37.34653

5.718	14.64304	32.15696	5.718	12.96442	33.83558	5.718	11.59278	35.20722	5.718	10.443	36.357	5.718	9.45835	37.34165
5.719	14.6461	32.1539	5.719	12.96806	33.83194	5.719	11.59691	35.20309	5.719	10.44753	36.35247	5.719	9.46322	37.33678
5.72	14.64916	32.15084	5.72	12.97171	33.82829	5.72	11.60104	35.19896	5.72	10.45206	36.34794	5.72	9.4681	37.3319
5.721	14.65222	32.14778	5.721	12.97536	33.82464	5.721	11.60516	35.19484	5.721	10.45658	36.34342	5.721	9.47297	37.32703
5.722	14.65528	32.14472	5.722	12.979	33.821	5.722	11.60928	35.19072	5.722	10.46111	36.33889	5.722	9.47783	37.32217
5.723	14.65833	32.14167	5.723	12.98264	33.81736	5.723	11.6134	35.1866	5.723	10.46563	36.33437	5.723	9.4827	37.3173
5.724	14.66139	32.13861	5.724	12.98628	33.81372	5.724	11.61752	35.18248	5.724	10.47015	36.32985	5.724	9.48756	37.31244
5.725	14.66444	32.13556	5.725	12.98992	33.81008	5.725	11.62164	35.17836	5.725	10.47466	36.32534	5.725	9.49242	37.30758
5.726	14.66749	32.13251	5.726	12.99355	33.80645	5.726	11.62575	35.17425	5.726	10.47918	36.32082	5.726	9.49728	37.30272
5.727	14.67054	32.12946	5.727	12.99719	33.80281	5.727	11.62986	35.17014	5.727	10.48369	36.31631	5.727	9.50213	37.29787
5.728	14.67359	32.12641	5.728	13.00082	33.79918	5.728	11.63397	35.16603	5.728	10.4882	36.3118	5.728	9.50699	37.29301
5.729	14.67663	32.12337	5.729	13.00445	33.79555	5.729	11.63808	35.16192	5.729	10.49271	36.30729	5.729	9.51184	37.28816
5.73	14.67968	32.12032	5.73	13.00808	33.79192	5.73	11.64219	35.15781	5.73	10.49721	36.30279	5.73	9.51668	37.28332
5.731	14.68272	32.11728	5.731	13.01171	33.78829	5.731	11.64629	35.15371	5.731	10.50171	36.29829	5.731	9.52153	37.27847
5.732	14.68576	32.11424	5.732	13.01533	33.78467	5.732	11.65039	35.14961	5.732	10.50621	36.29379	5.732	9.52637	37.27363
5.733	14.6888	32.1112	5.733	13.01895	33.78105	5.733	11.65449	35.14551	5.733	10.51071	36.28929	5.733	9.53121	37.26879
5.734	14.69184	32.10816	5.734	13.02257	33.77743	5.734	11.65858	35.14142	5.734	10.51521	36.28479	5.734	9.53605	37.26395
5.735	14.69488	32.10512	5.735	13.02619	33.77381	5.735	11.66268	35.13732	5.735	10.5197	36.2803	5.735	9.54088	37.25912
5.736	14.69791	32.10209	5.736	13.02981	33.77019	5.736	11.66677	35.13323	5.736	10.52419	36.27581	5.736	9.54571	37.25429
5.737	14.70095	32.09905	5.737	13.03342	33.76658	5.737	11.67086	35.12914	5.737	10.52868	36.27132	5.737	9.55054	37.24946
5.738	14.70398	32.09602	5.738	13.03704	33.76296	5.738	11.67495	35.12505	5.738	10.53316	36.26684	5.738	9.55537	37.24463
5.739	14.70701	32.09299	5.739	13.04065	33.75935	5.739	11.67903	35.12097	5.739	10.53765	36.26235	5.739	9.56019	37.23981
5.74	14.71004	32.08996	5.74	13.04426	33.75574	5.74	11.68312	35.11688	5.74	10.54213	36.25787	5.74	9.56501	37.23499
5.741	14.71306	32.08694	5.741	13.04786	33.75214	5.741	11.6872	35.1128	5.741	10.54661	36.25339	5.741	9.56983	37.23017
5.742	14.71609	32.08391	5.742	13.05147	33.74853	5.742	11.69128	35.10872	5.742	10.55108	36.24892	5.742	9.57465	37.22535
5.743	14.71911	32.08089	5.743	13.05507	33.74493	5.743	11.69536	35.10464	5.743	10.55556	36.24444	5.743	9.57946	37.22054
5.744	14.72214	32.07786	5.744	13.05867	33.74133	5.744	11.69943	35.10057	5.744	10.56003	36.23997	5.744	9.58428	37.21572
5.745	14.72516	32.07484	5.745	13.06227	33.73773	5.745	11.7035	35.0965	5.745	10.5645	36.2355	5.745	9.58909	37.21091
5.746	14.72818	32.07182	5.746	13.06587	33.73413	5.746	11.70757	35.09243	5.746	10.56897	36.23103	5.746	9.59389	37.20611
5.747	14.73119	32.06881	5.747	13.06947	33.73053	5.747	11.71164	35.08836	5.747	10.57343	36.22657	5.747	9.5987	37.2013
5.748	14.73421	32.06579	5.748	13.07306	33.72694	5.748	11.71571	35.08429	5.748	10.57789	36.22211	5.748	9.6035	37.1965
5.749	14.73722	32.06278	5.749	13.07665	33.72335	5.749	11.71977	35.08023	5.749	10.58236	36.21764	5.749	9.6083	37.1917
5.75	14.74024	32.05976	5.75	13.08024	33.71976	5.75	11.72384	35.07616	5.75	10.58681	36.21319	5.75	9.61309	37.18691
5.751	14.74325	32.05675	5.751	13.08383	33.71617	5.751	11.7279	35.0721	5.751	10.59127	36.20873	5.751	9.61789	37.18211
5.752	14.74626	32.05374	5.752	13.08742	33.71258	5.752	11.73195	35.06805	5.752	10.59572	36.20428	5.752	9.62268	37.17732
5.753	14.74927	32.05073	5.753	13.091	33.709	5.753	11.73601	35.06399	5.753	10.60017	36.19983	5.753	9.62747	37.17253
5.754	14.75227	32.04773	5.754	13.09459	33.70541	5.754	11.74006	35.05994	5.754	10.60462	36.19538	5.754	9.63226	37.16774
5.755	14.75528	32.04472	5.755	13.09817	33.70183	5.755	11.74412	35.05588	5.755	10.60907	36.19093	5.755	9.63704	37.16296
5.756	14.75828	32.04172	5.756	13.10175	33.69825	5.756	11.74817	35.05183	5.756	10.61351	36.18649	5.756	9.64182	37.15818
5.757	14.76128	32.03872	5.757	13.10533	33.69467	5.757	11.75221	35.04779	5.757	10.61795	36.18205	5.757	9.6466	37.1534
5.758	14.76428	32.03572	5.758	13.1089	33.6911	5.758	11.75626	35.04374	5.758	10.62239	36.17761	5.758	9.65138	37.14862
5.759	14.76728	32.03272	5.759	13.11247	33.68753	5.759	11.7603	35.0397	5.759	10.62683	36.17317	5.759	9.65615	37.14385
5.76	14.77028	32.02972	5.76	13.11605	33.68395	5.76	11.76434	35.03566	5.76	10.63126	36.16874	5.76	9.66092	37.13908
5.761	14.77327	32.02673	5.761	13.11962	33.68038	5.761	11.76838	35.03162	5.761	10.6357	36.1643	5.761	9.66569	37.13431
5.762	14.77627	32.02373	5.762	13.12318	33.67682	5.762	11.77242	35.02758	5.762	10.64013	36.15987	5.762	9.67046	37.12954
5.763	14.77926	32.02074	5.763	13.12675	33.67325	5.763	11.77645	35.02355	5.763	10.64455	36.15545	5.763	9.67522	37.12478
5.764	14.78225	32.01775	5.764	13.13031	33.66969	5.764	11.78049	35.01951	5.764	10.64898	36.15102	5.764	9.67999	37.12001
5.765	14.78524	32.01476	5.765	13.13388	33.66612	5.765	11.78452	35.01548	5.765	10.6534	36.1466	5.765	9.68475	37.11525
5.766	14.78823	32.01177	5.766	13.13744	33.66256	5.766	11.78855	35.01145	5.766	10.65782	36.14218	5.766	9.6895	37.1105
5.767	14.79122	32.00878	5.767	13.141	33.659	5.767	11.79257	35.00743	5.767	10.66224	36.13776	5.767	9.69426	37.10574
5.768	14.7942	32.0058	5.768	13.14455	33.65545	5.768	11.7966	35.0034	5.768	10.66666	36.13334	5.768	9.69901	37.10099
5.769	14.79718	32.00282	5.769	13.14811	33.65189	5.769	11.80062	34.99938	5.769	10.67107	36.12893	5.769	9.70376	37.09624
5.77	14.80017	31.99983	5.77	13.15166	33.64834	5.77	11.80464	34.99536	5.77	10.67549	36.12451	5.77	9.7085	37.0915
5.771	14.80315	31.99685	5.771	13.15521	33.64479	5.771	11.80866	34.99134	5.771	10.67989	36.12011	5.771	9.71325	37.08675
5.772	14.80612	31.99388	5.772	13.15876	33.64124	5.772	11.81267	34.98733	5.772	10.6843	36.1157	5.772	9.71799	37.08201
5.773	14.8091	31.9909	5.773	13.16231	33.63769	5.773	11.81669	34.98331	5.773	10.68871	36.11129	5.773	9.72273	37.07727
5.774	14.81208	31.98792	5.774	13.16586	33.63414	5.774	11.8207	34.9793	5.774	10.69311	36.10689	5.774	9.72747	37.07253
5.775	14.81505	31.98495	5.775	13.1694	33.6306	5.775	11.82471	34.97529	5.775	10.69751	36.10249	5.775	9.7322	37.0678
5.776	14.81802	31.98198	5.776	13.17294	33.62706	5.776	11.82872	34.97128	5.776	10.70191	36.09809	5.776	9.73693	37.06307
5.777	14.82099	31.97901	5.777	13.17648	33.62352	5.777	11.83272	34.96728	5.777	10.7063	36.0937	5.777	9.74166	37.05834
5.778	14.82396	31.97604	5.778	13.18002	33.61998	5.778	11.83673	34.96327	5.778	10.7107	36.0893	5.778	9.74639	37.05361
5.779	14.82693	31.97307	5.779	13.18356	33.61644	5.779	11.84073	34.95927	5.779	10.71509	36.08491	5.779	9.75112	37.04888
5.78	14.8299	31.9701	5.78	13.18709	33.61291	5.78	11.84473	34.95527	5.78	10.71948	36.08052	5.78	9.75584	37.04416
5.781	14.83286	31.96714	5.781	13.19063	33.60937	5.781	11.84872	34.95128	5.781	10.72386	36.07614	5.781	9.76056	37.03944
5.782	14.83582	31.96418	5.782	13.19416	33.60584	5.782	11.85272	34.94728	5.782	10.72825	36.07175	5.782	9.76528	37.03472
5.783	14.83879	31.96121	5.783	13.19769	33.60231	5.783	11.85671	34.94329	5.783	10.73263	36.06737	5.783	9.76999	37.03001
5.784	14.84175	31.95825	5.784	13.20121	33.59879	5.784	11.8607	34.9393	5.784	10.73701	36.06299	5.784	9.7747	37.0253
5.785	14.8447	31.9553	5.785	13.20474	33.59526	5.785	11.86469	34.93531	5.785	10.74139	36.05861	5.785	9.77941	37.02059
5.786	14.84766	31.95234	5.786	13.20826	33.59174	5.786	11.86868	34.93132	5.786	10.74576	36.05424	5.786	9.78412	37.01588
5.787	14.85062	31.94938	5.787	13.21178	33.58822	5.787	11.87267	34.92733	5.787	10.75013	36.04987	5.787	9.78883	37.01117
5.788	14.85357	31.94643	5.788	13.2153	33.5847	5.788	11.87665	34.92335	5.788	10.75451	36.04549	5.788	9.79353	37.00647
5.789	14.85652	31.94348	5.789	13.21882	33.58118	5.789	11.88063	34.91937	5.789	10.75887	36.04113	5.789	9.79823	37.00177
5.79	14.85947	31.94053	5.79	13.22234	33.57766	5.79	11.88461	34.91539	5.79	10.76324	36.03676	5.79	9.80293	36.99707
5.791	14.86242	31.93758	5.791	13.22585	33.57415	5.791	11.88858	34.91142	5.791	10.7676	36.0324	5.791	9.80762	36.99238
5.792	14.86537	31.93463	5.792	13.22937	33.57063	5.792	11.89256	34.90744	5.792	10.77197	36.02803	5.792	9.81232	36.98768
5.793	14.86832	31.93168	5.793	13.23288	33.56712	5.793	11.89653	34.90347	5.793	10.77633	36.02367	5.793	9.81701	36.98299
5.794	14.87126	31.92874	5.794	13.23639	33.56361	5.794	11.9005	34.8995	5.794	10.78068	36.01932	5.794	9.8217	36.9783
5.795	14.87421	31.92579	5.795	13.23989	33.56011	5.795	11.90447	34.89553	5.795	10.78504	36.01496	5.795	9.82638	36.97362
5.796	14.87715	31.92285	5.796	13.2434	33.5566	5.796	11.90844	34.89156	5.796	10.78939	36.01061	5.796	9.83107	36.96893
5.797	14.88009	31.91991	5.797	13.2469	33.5531	5.797	11.9124	34.8876	5.797	10.79374	36.00626	5.797	9.83575	36.96425
5.798	14.88303	31.91697	5.798	13.25041	33.54959	5.798	11.91636	34.88364	5.798	10.79809	36.00191	5.798	9.84042	36.95958
5.799	14.88596	31.91404	5.799	13.25391	33.54609	5.799	11.92032	34.87968	5.799	10.80243	35.99757	5.799	9.8451	36.9549
5.8	14.8889	31.9111	5.8	13.2574	33.5426	5.8	11.92428	34.87572	5.8	10.80678	35.99322	5.8	9.84977	36.95023
5.801	14.89183	31.90817	5.801	13.2609	33.5391	5.801	11.92824	34.87176	5.801	10.81112	35.98888	5.801	9.85445	36.94555
5.802	14.89477	31.90523	5.802	13.2644	33.5356	5.802	11.93219	34.86781	5.802	10.81546	35.98454	5.802	9.85911	36.94089
5.803	14.8977	31.9023	5.803	13.26789	33.53211	5.803	11.93614	34.86386	5.803	10.8198	35.9802	5.803	9.86378	36.93622
5.804	14.90063	31.89937	5.804	13.27138	33.52862	5.804	11.94009	34.85991	5.804	10.82413	35.97587	5.804	9.86845	36.93155
5.805	14.90355	31.89645	5.805	13.27487	33.52513	5.805	11.94404	34.85596	5.805	10.82846	35.97154	5.805	9.87311	36.92689
5.806	14.90648	31.89352	5.806	13.27836	33.52164	5.806	11.94799	34.85201	5.806	10.83279	35.96721	5.806	9.87777	36.92223
5.807	14.90941	31.89059	5.807	13.28184	33.51816	5.807	11.95193	34.84807	5.807	10.83712	35.96288	5.807	9.88242	36.91758
5.808	14.91233	31.88767	5.808	13.28533	33.51467	5.808	11.95587	34.84413	5.808	10.84145	35.95855	5.808	9.88708	36.91292
5.809	14.91525	31.88475	5.809	13.28881	33.51119	5.809	11.95981	34.84019	5.809	10.84577	35.95423	5.809	9.89173	36.90827
5.81	14.91817	31.88183	5.81	13.29229	33.50771	5.81	11.96375	34.83625	5.81	10.85009	35.94991	5.81	9.89638	36.90362
5.811	14.92109	31.87891	5.811	13.29577	33.50423	5.811	11.96769	34.83231	5.811	10.85441	35.94559	5.811	9.90103	36.89897
5.812	14.92401	31.87599	5.812	13.29924	33.50076	5.812	11.97162	34.82838	5.812	10.85873	35.94127	5.812	9.90567	36.89433
5.813	14.92692	31.87308	5.813	13.30272	33.49728	5.813	11.97555	34.82445	5.813	10.86304	35.93696	5.813	9.91031	36.88969
5.814	14.92984	31.87016	5.814	13.30619	33.49381	5.814	11.97948	34.82052	5.814	10.86735	35.93265	5.814	9.91495	36.88505
5.815	14.93275	31.86725	5.815	13.30966	33.49034	5.815	11.98341	34.81659	5.815	10.87166	35.92834	5.815	9.91959	36.88041
5.816	14.93566	31.86434	5.816	13.31313	33.48687	5.816	11.98734	34.81266	5.816	10.87597	35.92403	5.816	9.92423	36.87577
5.817	14.93857	31.86143	5.817	13.3166	33.4834	5.817	11.99126	34.80874	5.817	10.88028	35.91972	5.817	9.92886	36.87114
5.818	14.94148	31.85852	5.818	13.32007	33.47993	5.818	11.99518	34.80482	5.818	10.88458	35.91542	5.818	9.93349	36.86651
5.819	14.94439	31.85561	5.819	13.32353	33.47647	5.819	11.9991	34.8009	5.819	10.88888	35.91112	5.819	9.93812	36.86188
5.82	14.94729	31.85271	5.82	13.32699	33.47301	5.82	12.00302	34.79698	5.82	10.89318	35.90682	5.82	9.94274	36.85726
5.821	14.9502	31.8498	5.821	13.33045	33.46955	5.821	12.00693	34.79307	5.821	10.89748	35.90252	5.821	9.94737	36.85263
5.822	14.9531	31.8469	5.822	13.33391	33.46609	5.822	12.01085	34.78915	5.822	10.90177	35.89823	5.822	9.95199	36.84801
5.823	14.956	31.844	5.823	13.33737	33.46263	5.823	12.01476	34.78524	5.823	10.90606	35.89394	5.823	9.95661	36.84339
5.824	14.9589	31.8411	5.824	13.34083	33.45917	5.824	12.01867	34.78133	5.824	10.91035	35.88965	5.824	9.96122	36.83878
5.825	14.9618	31.8382	5.825	13.34428	33.45572	5.825	12.02257	34.77743	5.825	10.91464	35.88536	5.825	9.96584	36.83416
5.826	14.9647	31.8353	5.826	13.34773	33.45227	5.826	12.02648	34.77352	5.826	10.91893	35.88107	5.826	9.97045	36.82955
5.827	14.96759	31.83241	5.827	13.35118	33.44882	5.827	12.03038	34.76962	5.827	10.92321	35.87679	5.827	9.97506	36.82494
5.828	14.97048	31.82952	5.828	13.35463	33.44537	5.828	12.03428	34.76572	5.828	10.92749	35.87251	5.828	9.97966	36.82034
5.829	14.97338	31.82662	5.829	13.35808	33.44192	5.829	12.03818	34.76182	5.829	10.93177	35.86823	5.829	9.98427	36.81573
5.83	14.97627	31.82373	5.83	13.36152	33.43848	5.83	12.04208	34.75792	5.83	10.93605	35.86395	5.83	9.98887	36.81113
5.831	14.97916	31.82084	5.831	13.36496	33.43504	5.831	12.04598	34.75402	5.831	10.94032	35.85968	5.831	9.99347	36.80653
5.832	14.98204	31.81796	5.832	13.3684	33.4316	5.832	12.04987	34.75013	5.832	10.9446	35.8554	5.832	9.99807	36.80193
5.833	14.98493	31.81507	5.833	13.37184	33.42816	5.833	12.05376	34.74624	5.833	10.94887	35.85113	5.833	10.00266	36.79734
5.834	14.98781	31.81219	5.834	13.37528	33.42472	5.834	12.05765	34.74235	5.834	10.95313	35.84687	5.834	10.00725	36.79275
5.835	14.9907	31.8093	5.835	13.37872	33.42128	5.835	12.06154	34.73846	5.835	10.9574	35.8426	5.835	10.01184	36.78816
5.836	14.99358	31.80642	5.836	13.38215	33.41785	5.836	12.06542	34.73458	5.836	10.96166	35.83834	5.836	10.01643	36.78357
5.837	14.99646	31.80354	5.837	13.38558	33.41442	5.837	12.06931	34.73069	5.837	10.96593	35.83407	5.837	10.02102	36.77898
5.838	14.99934	31.80066	5.838	13.38901	33.41099	5.838	12.07319	34.72681	5.838	10.97018	35.82982	5.838	10.0256	36.7744
5.839	15.00221	31.79779	5.839	13.39244	33.40756	5.839	12.07707	34.72293	5.839	10.97444	35.82556	5.839	10.03018	36.76982
5.84	15.00509	31.79491	5.84	13.39587	33.40413	5.84	12.08094	34.71906	5.84	10.9787	35.8213	5.84	10.03476	36.76524
5.841	15.00796	31.79204	5.841	13.39929	33.40071	5.841	12.08482	34.71518	5.841	10.98295	35.81705	5.841	10.03933	36.76067
5.842	15.01084	31.78916	5.842	13.40272	33.39728	5.842	12.08869	34.71131	5.842	10.9872	35.8128	5.842	10.04391	36.75609
5.843	15.01371	31.78629	5.843	13.40614	33.39386	5.843	12.09257	34.70743	5.843	10.99145	35.80855	5.843	10.04848	36.75152
5.844	15.01658	31.78342	5.844	13.40956	33.39044	5.844	12.09643	34.70357	5.844	10.99569	35.80431	5.844	10.05305	36.74695
5.845	15.01945	31.78055	5.845	13.41298	33.38702	5.845	12.1003	34.6997	5.845	10.99994	35.80006	5.845	10.05761	36.74239
5.846	15.02231	31.77769	5.846	13.41639	33.38361	5.846	12.10417	34.69583	5.846	11.00418	35.79582	5.846	10.06218	36.73782
5.847	15.02518	31.77482	5.847	13.41981	33.38019	5.847	12.10803	34.69197	5.847	11.00842	35.79158	5.847	10.06674	36.73326
5.848	15.02804	31.77196	5.848	13.42322	33.37678	5.848	12.11189	34.68811	5.848	11.01266	35.78734	5.848	10.0713	36.7287
5.849	15.03091	31.76909	5.849	13.42663	33.37337	5.849	12.11575	34.68425	5.849	11.01689	35.78311	5.849	10.07586	36.72414
5.85	15.03377	31.76623	5.85	13.43004	33.36996	5.85	12.11961	34.68039	5.85	11.02113	35.77887	5.85	10.08041	36.71959
5.851	15.03663	31.76337	5.851	13.43345	33.36655	5.851	12.12347	34.67653	5.851	11.02536	35.77464	5.851	10.08497	36.71503
5.852	15.03948	31.76052	5.852	13.43686	33.36314	5.852	12.12732	34.67268	5.852	11.02959	35.77041	5.852	10.08952	36.71048
5.853	15.04234	31.75766	5.853	13.44026	33.35974	5.853	12.13117	34.66883	5.853	11.03381	35.76619	5.853	10.09406	36.70594
5.854	15.0452	31.7548	5.854	13.44366	33.35634	5.854	12.13502	34.66498	5.854	11.03804	35.76196	5.854	10.09861	36.70139
5.855	15.04805	31.75195	5.855	13.44706	33.35294	5.855	12.13887	34.66113	5.855	11.04226	35.75774	5.855	10.10315	36.69685
5.856	15.0509	31.7491	5.856	13.45046	33.34954	5.856	12.14271	34.65729	5.856	11.04648	35.75352	5.856	10.10769	36.69231
5.857	15.05375	31.74625	5.857	13.45386	33.34614	5.857	12.14656	34.65344	5.857	11.0507	35.7493	5.857	10.11223	36.68777
5.858	15.0566	31.7434	5.858	13.45725	33.34275	5.858	12.1504	34.6496	5.858	11.05491	35.74509	5.858	10.11677	36.68323
5.859	15.05945	31.74055	5.859	13.46065	33.33935	5.859	12.15424	34.64576	5.859	11.05913	35.74087	5.859	10.1213	36.6787
5.86	15.0623	31.7377	5.86	13.46404	33.33596	5.86	12.15808	34.64192	5.86	11.06334	35.73666	5.86	10.12583	36.67417
5.861	15.06514	31.73486	5.861	13.46743	33.33257	5.861	12.16191	34.63809	5.861	11.06755	35.73245	5.861	10.13036	36.66964
5.862	15.06798	31.73202	5.862	13.47082	33.32918	5.862	12.16575	34.63425	5.862	11.07176	35.72824	5.862	10.13489	36.66511
5.863	15.07083	31.72917	5.863	13.47421	33.32579	5.863	12.16958	34.63042	5.863	11.07596	35.72404	5.863	10.13941	36.66059
5.864	15.07367	31.72633	5.864	13.47759	33.32241	5.864	12.17341	34.62659	5.864	11.08016	35.71984	5.864	10.14394	36.65606
5.865	15.07651	31.72349	5.865	13.48097	33.31903	5.865	12.17724	34.62276	5.865	11.08436	35.71564	5.865	10.14846	36.65154
5.866	15.07934	31.72066	5.866	13.48436	33.31564	5.866	12.18106	34.61894	5.866	11.08856	35.71144	5.866	10.15297	36.64703
5.867	15.08218	31.71782	5.867	13.48773	33.31227	5.867	12.18489	34.61511	5.867	11.09276	35.70724	5.867	10.15749	36.64251
5.868	15.08501	31.71499	5.868	13.49111	33.30889	5.868	12.18871	34.61129	5.868	11.09695	35.70305	5.868	10.162	36.638
5.869	15.08785	31.71215	5.869	13.49449	33.30551	5.869	12.19253	34.60747	5.869	11.10115	35.69885	5.869	10.16651	36.63349
5.87	15.09068	31.70932	5.87	13.49786	33.30214	5.87	12.19635	34.60365	5.87	11.10534	35.69466	5.87	10.17102	36.62898
5.871	15.09351	31.70649	5.871	13.50124	33.29876	5.871	12.20016	34.59984	5.871	11.10952	35.69048	5.871	10.17553	36.62447
5.872	15.09634	31.70366	5.872	13.50461	33.29539	5.872	12.20398	34.59602	5.872	11.11371	35.68629	5.872	10.18003	36.61997
5.873	15.09917	31.70083	5.873	13.50798	33.29202	5.873	12.20779	34.59221	5.873	11.11789	35.68211	5.873	10.18453	36.61547
5.874	15.10199	31.69801	5.874	13.51135	33.28865	5.874	12.2116	34.5884	5.874	11.12208	35.67792	5.874	10.18903	36.61097
5.875	15.10482	31.69518	5.875	13.51471	33.28529	5.875	12.21541	34.58459	5.875	11.12625	35.67375	5.875	10.19353	36.60647
5.876	15.10764	31.69236	5.876	13.51808	33.28192	5.876	12.21922	34.58078	5.876	11.13043	35.66957	5.876	10.19802	36.60198
5.877	15.11046	31.68954	5.877	13.52144	33.27856	5.877	12.22302	34.57698	5.877	11.13461	35.66539	5.877	10.20252	36.59748

5.878	15.11328	31.68672	5.878	13.5248	33.2752	5.878	12.22682	34.57318	5.878	11.13878	35.66122	5.878	10.20701	36.59299
5.879	15.1161	31.6839	5.879	13.52816	33.27184	5.879	12.23062	34.56938	5.879	11.14295	35.65705	5.879	10.21149	36.58851
5.88	15.11892	31.68108	5.88	13.53152	33.26848	5.88	12.23442	34.56558	5.88	11.14712	35.65288	5.88	10.21598	36.58402
5.881	15.12173	31.67827	5.881	13.53487	33.26513	5.881	12.23822	34.56178	5.881	11.15129	35.64871	5.881	10.22046	36.57954
5.882	15.12455	31.67545	5.882	13.53823	33.26177	5.882	12.24201	34.55799	5.882	11.15545	35.64455	5.882	10.22494	36.57506
5.883	15.12736	31.67264	5.883	13.54158	33.25842	5.883	12.24581	34.55419	5.883	11.15961	35.64039	5.883	10.22942	36.57058
5.884	15.13017	31.66983	5.884	13.54493	33.25507	5.884	12.2496	34.5504	5.884	11.16377	35.63623	5.884	10.2339	36.5661
5.885	15.13298	31.66702	5.885	13.54828	33.25172	5.885	12.25339	34.54661	5.885	11.16793	35.63207	5.885	10.23837	36.56163
5.886	15.13579	31.66421	5.886	13.55163	33.24837	5.886	12.25717	34.54283	5.886	11.17209	35.62791	5.886	10.24285	36.55715
5.887	15.1386	31.6614	5.887	13.55497	33.24503	5.887	12.26096	34.53904	5.887	11.17624	35.62376	5.887	10.24731	36.55269
5.888	15.14141	31.65859	5.888	13.55831	33.24169	5.888	12.26474	34.53526	5.888	11.18039	35.61961	5.888	10.25178	36.54822
5.889	15.14421	31.65579	5.889	13.56166	33.23834	5.889	12.26852	34.53148	5.889	11.18454	35.61546	5.889	10.25625	36.54375
5.89	15.14701	31.65299	5.89	13.565	33.235	5.89	12.2723	34.5277	5.89	11.18869	35.61131	5.89	10.26071	36.53929
5.891	15.14982	31.65018	5.891	13.56834	33.23166	5.891	12.27608	34.52392	5.891	11.19284	35.60716	5.891	10.26517	36.53483
5.892	15.15262	31.64738	5.892	13.57167	33.22833	5.892	12.27986	34.52014	5.892	11.19698	35.60302	5.892	10.26963	36.53037
5.893	15.15541	31.64459	5.893	13.57501	33.22499	5.893	12.28363	34.51637	5.893	11.20112	35.59888	5.893	10.27408	36.52592
5.894	15.15821	31.64179	5.894	13.57834	33.22166	5.894	12.2874	34.5126	5.894	11.20526	35.59474	5.894	10.27854	36.52146
5.895	15.16101	31.63899	5.895	13.58167	33.21833	5.895	12.29117	34.50883	5.895	11.2094	35.5906	5.895	10.28299	36.51701
5.896	15.1638	31.6362	5.896	13.585	33.215	5.896	12.29494	34.50506	5.896	11.21353	35.58647	5.896	10.28744	36.51256
5.897	15.1666	31.6334	5.897	13.58833	33.21167	5.897	12.29871	34.50129	5.897	11.21766	35.58234	5.897	10.29188	36.50812
5.898	15.16939	31.63061	5.898	13.59166	33.20834	5.898	12.30247	34.49753	5.898	11.22179	35.57821	5.898	10.29633	36.50367
5.899	15.17218	31.62782	5.899	13.59498	33.20502	5.899	12.30623	34.49377	5.899	11.22592	35.57408	5.899	10.30077	36.49923
5.9	15.17497	31.62503	5.9	13.59831	33.20169	5.9	12.30999	34.49001	5.9	11.23005	35.56995	5.9	10.30521	36.49479
5.901	15.17775	31.62225	5.901	13.60163	33.19837	5.901	12.31375	34.48625	5.901	11.23417	35.56583	5.901	10.30965	36.49035
5.902	15.18054	31.61946	5.902	13.60495	33.19505	5.902	12.31751	34.48249	5.902	11.2383	35.5617	5.902	10.31408	36.48592
5.903	15.18332	31.61668	5.903	13.60827	33.19173	5.903	12.32126	34.47874	5.903	11.24242	35.55758	5.903	10.31852	36.48148
5.904	15.18611	31.61389	5.904	13.61158	33.18842	5.904	12.32501	34.47499	5.904	11.24653	35.55347	5.904	10.32295	36.47705
5.905	15.18889	31.61111	5.905	13.6149	33.1851	5.905	12.32877	34.47123	5.905	11.25065	35.54935	5.905	10.32738	36.47262
5.906	15.19167	31.60833	5.906	13.61821	33.18179	5.906	12.33251	34.46749	5.906	11.25476	35.54524	5.906	10.3318	36.4682
5.907	15.19445	31.60555	5.907	13.62152	33.17848	5.907	12.33626	34.46374	5.907	11.25888	35.54112	5.907	10.33623	36.46377
5.908	15.19723	31.60277	5.908	13.62483	33.17517	5.908	12.34001	34.45999	5.908	11.26299	35.53701	5.908	10.34065	36.45935
5.909	15.2	31.6	5.909	13.62814	33.17186	5.909	12.34375	34.45625	5.909	11.26709	35.53291	5.909	10.34507	36.45493
5.91	15.20278	31.59722	5.91	13.63145	33.16855	5.91	12.34749	34.45251	5.91	11.2712	35.5288	5.91	10.34949	36.45051
5.911	15.20555	31.59445	5.911	13.63475	33.16525	5.911	12.35123	34.44877	5.911	11.2753	35.5247	5.911	10.3539	36.4461
5.912	15.20832	31.59168	5.912	13.63806	33.16194	5.912	12.35497	34.44503	5.912	11.2794	35.5206	5.912	10.35832	36.44168
5.913	15.21109	31.58891	5.913	13.64136	33.15864	5.913	12.3587	34.4413	5.913	11.2835	35.5165	5.913	10.36273	36.43727
5.914	15.21386	31.58614	5.914	13.64466	33.15534	5.914	12.36244	34.43756	5.914	11.2876	35.5124	5.914	10.36714	36.43286
5.915	15.21663	31.58337	5.915	13.64796	33.15204	5.915	12.36617	34.43383	5.915	11.29169	35.50831	5.915	10.37154	36.42846
5.916	15.2194	31.5806	5.916	13.65125	33.14875	5.916	12.3699	34.4301	5.916	11.29579	35.50421	5.916	10.37595	36.42405
5.917	15.22216	31.57784	5.917	13.65455	33.14545	5.917	12.37363	34.42637	5.917	11.29988	35.50012	5.917	10.38035	36.41965
5.918	15.22492	31.57508	5.918	13.65784	33.14216	5.918	12.37735	34.42265	5.918	11.30397	35.49603	5.918	10.38475	36.41525
5.919	15.22769	31.57231	5.919	13.66113	33.13887	5.919	12.38108	34.41892	5.919	11.30805	35.49195	5.919	10.38915	36.41085
5.92	15.23045	31.56955	5.92	13.66442	33.13558	5.92	12.3848	34.4152	5.92	11.31214	35.48786	5.92	10.39354	36.40646
5.921	15.23321	31.56679	5.921	13.66771	33.13229	5.921	12.38852	34.41148	5.921	11.31622	35.48378	5.921	10.39793	36.40207
5.922	15.23596	31.56404	5.922	13.671	33.129	5.922	12.39224	34.40776	5.922	11.3203	35.4797	5.922	10.40232	36.39768
5.923	15.23872	31.56128	5.923	13.67428	33.12572	5.923	12.39595	34.40405	5.923	11.32438	35.47562	5.923	10.40671	36.39329
5.924	15.24148	31.55852	5.924	13.67757	33.12243	5.924	12.39967	34.40033	5.924	11.32846	35.47154	5.924	10.4111	36.3889
5.925	15.24423	31.55577	5.925	13.68085	33.11915	5.925	12.40338	34.39662	5.925	11.33253	35.46747	5.925	10.41548	36.38452
5.926	15.24698	31.55302	5.926	13.68413	33.11587	5.926	12.40709	34.39291	5.926	11.3366	35.4634	5.926	10.41986	36.38014
5.927	15.24973	31.55027	5.927	13.68741	33.11259	5.927	12.4108	34.3892	5.927	11.34067	35.45933	5.927	10.42424	36.37576
5.928	15.25248	31.54752	5.928	13.69068	33.10932	5.928	12.41451	34.38549	5.928	11.34474	35.45526	5.928	10.42862	36.37138
5.929	15.25523	31.54477	5.929	13.69396	33.10604	5.929	12.41821	34.38179	5.929	11.34881	35.45119	5.929	10.433	36.367
5.93	15.25798	31.54202	5.93	13.69723	33.10277	5.93	12.42192	34.37808	5.93	11.35287	35.44713	5.93	10.43737	36.36263
5.931	15.26072	31.53928	5.931	13.7005	33.0995	5.931	12.42562	34.37438	5.931	11.35694	35.44306	5.931	10.44174	36.35826
5.932	15.26347	31.53653	5.932	13.70377	33.09623	5.932	12.42932	34.37068	5.932	11.361	35.439	5.932	10.44611	36.35389
5.933	15.26621	31.53379	5.933	13.70704	33.09296	5.933	12.43302	34.36698	5.933	11.36505	35.43495	5.933	10.45048	36.34952
5.934	15.26895	31.53105	5.934	13.71031	33.08969	5.934	12.43671	34.36329	5.934	11.36911	35.43089	5.934	10.45484	36.34516
5.935	15.27169	31.52831	5.935	13.71357	33.08643	5.935	12.44041	34.35959	5.935	11.37316	35.42684	5.935	10.4592	36.3408
5.936	15.27443	31.52557	5.936	13.71684	33.08316	5.936	12.4441	34.3559	5.936	11.37722	35.42278	5.936	10.46356	36.33644
5.937	15.27716	31.52284	5.937	13.7201	33.0799	5.937	12.44779	34.35221	5.937	11.38127	35.41873	5.937	10.46792	36.33208
5.938	15.2799	31.5201	5.938	13.72336	33.07664	5.938	12.45148	34.34852	5.938	11.38531	35.41469	5.938	10.47227	36.32773
5.939	15.28263	31.51737	5.939	13.72662	33.07338	5.939	12.45517	34.34483	5.939	11.38936	35.41064	5.939	10.47663	36.32337
5.94	15.28537	31.51463	5.94	13.72987	33.07013	5.94	12.45885	34.34115	5.94	11.3934	35.4066	5.94	10.48098	36.31902
5.941	15.2881	31.5119	5.941	13.73313	33.06687	5.941	12.46253	34.33747	5.941	11.39744	35.40256	5.941	10.48533	36.31467
5.942	15.29083	31.50917	5.942	13.73638	33.06362	5.942	12.46621	34.33379	5.942	11.40148	35.39852	5.942	10.48967	36.31033
5.943	15.29356	31.50644	5.943	13.73963	33.06037	5.943	12.46989	34.33011	5.943	11.40552	35.39448	5.943	10.49402	36.30598
5.944	15.29629	31.50371	5.944	13.74288	33.05712	5.944	12.47357	34.32643	5.944	11.40956	35.39044	5.944	10.49836	36.30164
5.945	15.29901	31.50099	5.945	13.74613	33.05387	5.945	12.47725	34.32275	5.945	11.41359	35.38641	5.945	10.5027	36.2973
5.946	15.30174	31.49826	5.946	13.74938	33.05062	5.946	12.48092	34.31908	5.946	11.41762	35.38238	5.946	10.50704	36.29296
5.947	15.30446	31.49554	5.947	13.75262	33.04738	5.947	12.48459	34.31541	5.947	11.42165	35.37835	5.947	10.51137	36.28863
5.948	15.30718	31.49282	5.948	13.75587	33.04413	5.948	12.48826	34.31174	5.948	11.42568	35.37432	5.948	10.51571	36.28429
5.949	15.3099	31.4901	5.949	13.75911	33.04089	5.949	12.49193	34.30807	5.949	11.4297	35.3703	5.949	10.52004	36.27996
5.95	15.31262	31.48738	5.95	13.76235	33.03765	5.95	12.4956	34.3044	5.95	11.43373	35.36627	5.95	10.52437	36.27563
5.951	15.31534	31.48466	5.951	13.76559	33.03441	5.951	12.49926	34.30074	5.951	11.43775	35.36225	5.951	10.52869	36.27131
5.952	15.31806	31.48194	5.952	13.76883	33.03117	5.952	12.50292	34.29708	5.952	11.44177	35.35823	5.952	10.53302	36.26698
5.953	15.32077	31.47923	5.953	13.77206	33.02794	5.953	12.50658	34.29342	5.953	11.44578	35.35422	5.953	10.53734	36.26266
5.954	15.32348	31.47652	5.954	13.7753	33.0247	5.954	12.51024	34.28976	5.954	11.4498	35.3502	5.954	10.54166	36.25834
5.955	15.3262	31.4738	5.955	13.77853	33.02147	5.955	12.5139	34.2861	5.955	11.45381	35.34619	5.955	10.54598	36.25402
5.956	15.32891	31.47109	5.956	13.78176	33.01824	5.956	12.51755	34.28245	5.956	11.45782	35.34218	5.956	10.5503	36.2497
5.957	15.33162	31.46838	5.957	13.78499	33.01501	5.957	12.52121	34.27879	5.957	11.46183	35.33817	5.957	10.55461	36.24539
5.958	15.33432	31.46568	5.958	13.78821	33.01179	5.958	12.52486	34.27514	5.958	11.46584	35.33416	5.958	10.55892	36.24108
5.959	15.33703	31.46297	5.959	13.79144	33.00856	5.959	12.52851	34.27149	5.959	11.46985	35.33015	5.959	10.56323	36.23677
5.96	15.33974	31.46026	5.96	13.79466	33.00534	5.96	12.53216	34.26784	5.96	11.47385	35.32615	5.96	10.56754	36.23246
5.961	15.34244	31.45756	5.961	13.79789	33.00211	5.961	12.5358	34.2642	5.961	11.47785	35.32215	5.961	10.57184	36.22816
5.962	15.34514	31.45486	5.962	13.80111	32.99889	5.962	12.53945	34.26055	5.962	11.48185	35.31815	5.962	10.57615	36.22385
5.963	15.34785	31.45215	5.963	13.80433	32.99567	5.963	12.54309	34.25691	5.963	11.48585	35.31415	5.963	10.58045	36.21955
5.964	15.35055	31.44945	5.964	13.80754	32.99246	5.964	12.54673	34.25327	5.964	11.48984	35.31016	5.964	10.58475	36.21525
5.965	15.35324	31.44676	5.965	13.81076	32.98924	5.965	12.55037	34.24963	5.965	11.49383	35.30617	5.965	10.58904	36.21096
5.966	15.35594	31.44406	5.966	13.81397	32.98603	5.966	12.55401	34.24599	5.966	11.49782	35.30218	5.966	10.59334	36.20666
5.967	15.35864	31.44136	5.967	13.81719	32.98281	5.967	12.55764	34.24236	5.967	11.50181	35.29819	5.967	10.59763	36.20237
5.968	15.36133	31.43867	5.968	13.8204	32.9796	5.968	12.56127	34.23873	5.968	11.5058	35.2942	5.968	10.60192	36.19808
5.969	15.36403	31.43597	5.969	13.82361	32.97639	5.969	12.56491	34.23509	5.969	11.50979	35.29021	5.969	10.60621	36.19379
5.97	15.36672	31.43328	5.97	13.82682	32.97318	5.97	12.56853	34.23147	5.97	11.51377	35.28623	5.97	10.61049	36.18951
5.971	15.36941	31.43059	5.971	13.83002	32.96998	5.971	12.57216	34.22784	5.971	11.51775	35.28225	5.971	10.61478	36.18522
5.972	15.3721	31.4279	5.972	13.83323	32.96677	5.972	12.57579	34.22421	5.972	11.52173	35.27827	5.972	10.61906	36.18094
5.973	15.37478	31.42522	5.973	13.83643	32.96357	5.973	12.57941	34.22059	5.973	11.52571	35.27429	5.973	10.62334	36.17666
5.974	15.37747	31.42253	5.974	13.83963	32.96037	5.974	12.58303	34.21697	5.974	11.52968	35.27032	5.974	10.62761	36.17239
5.975	15.38016	31.41984	5.975	13.84283	32.95717	5.975	12.58666	34.21334	5.975	11.53365	35.26635	5.975	10.63189	36.16811
5.976	15.38284	31.41716	5.976	13.84603	32.95397	5.976	12.59027	34.20973	5.976	11.53762	35.26238	5.976	10.63616	36.16384
5.977	15.38552	31.41448	5.977	13.84923	32.95077	5.977	12.59389	34.20611	5.977	11.54159	35.25841	5.977	10.64043	36.15957
5.978	15.3882	31.4118	5.978	13.85242	32.94758	5.978	12.59751	34.20249	5.978	11.54556	35.25444	5.978	10.6447	36.1553
5.979	15.39088	31.40912	5.979	13.85561	32.94439	5.979	12.60112	34.19888	5.979	11.54953	35.25047	5.979	10.64897	36.15103
5.98	15.39356	31.40644	5.98	13.85881	32.94119	5.98	12.60473	34.19527	5.98	11.55349	35.24651	5.98	10.65323	36.14677
5.981	15.39624	31.40376	5.981	13.862	32.938	5.981	12.60834	34.19166	5.981	11.55745	35.24255	5.981	10.65749	36.14251
5.982	15.39891	31.40109	5.982	13.86519	32.93481	5.982	12.61195	34.18805	5.982	11.56141	35.23859	5.982	10.66175	36.13825
5.983	15.40159	31.39841	5.983	13.86837	32.93163	5.983	12.61555	34.18445	5.983	11.56537	35.23463	5.983	10.66601	36.13399
5.984	15.40426	31.39574	5.984	13.87156	32.92844	5.984	12.61916	34.18084	5.984	11.56932	35.23068	5.984	10.67027	36.12973
5.985	15.40693	31.39307	5.985	13.87474	32.92526	5.985	12.62276	34.17724	5.985	11.57327	35.22673	5.985	10.67452	36.12548
5.986	15.4096	31.3904	5.986	13.87792	32.92208	5.986	12.62636	34.17364	5.986	11.57722	35.22278	5.986	10.67877	36.12123
5.987	15.41227	31.38773	5.987	13.8811	32.9189	5.987	12.62996	34.17004	5.987	11.58117	35.21883	5.987	10.68302	36.11698
5.988	15.41494	31.38506	5.988	13.88428	32.91572	5.988	12.63356	34.16644	5.988	11.58512	35.21488	5.988	10.68727	36.11273
5.989	15.41761	31.38239	5.989	13.88746	32.91254	5.989	12.63715	34.16285	5.989	11.58907	35.21093	5.989	10.69151	36.10849
5.99	15.42027	31.37973	5.99	13.89064	32.90936	5.99	12.64074	34.15926	5.99	11.59301	35.20699	5.99	10.69575	36.10425
5.991	15.42294	31.37706	5.991	13.89381	32.90619	5.991	12.64434	34.15566	5.991	11.59695	35.20305	5.991	10.7	36.1
5.992	15.4256	31.3744	5.992	13.89698	32.90302	5.992	12.64793	34.15207	5.992	11.60089	35.19911	5.992	10.70423	36.09577
5.993	15.42826	31.37174	5.993	13.90015	32.89985	5.993	12.65151	34.14849	5.993	11.60483	35.19517	5.993	10.70847	36.09153
5.994	15.43092	31.36908	5.994	13.90332	32.89668	5.994	12.6551	34.1449	5.994	11.60876	35.19124	5.994	10.71271	36.08729
5.995	15.43358	31.36642	5.995	13.90649	32.89351	5.995	12.65868	34.14132	5.995	11.6127	35.1873	5.995	10.71694	36.08306
5.996	15.43623	31.36377	5.996	13.90966	32.89034	5.996	12.66227	34.13773	5.996	11.61663	35.18337	5.996	10.72117	36.07883
5.997	15.43889	31.36111	5.997	13.91282	32.88718	5.997	12.66585	34.13415	5.997	11.62056	35.17944	5.997	10.7254	36.0746
5.998	15.44154	31.35846	5.998	13.91599	32.88401	5.998	12.66943	34.13057	5.998	11.62448	35.17552	5.998	10.72962	36.07038
5.999	15.4442	31.3558	5.999	13.91915	32.88085	5.999	12.673	34.127	5.999	11.62841	35.17159	5.999	10.73385	36.06615
6	15.44685	31.35315	6	13.92231	32.87769	6	12.67658	34.12342	6	11.63233	35.16767	6	10.73807	36.06193

b=1			b=0.75			b=0.5			b=0.25			b=0		
distance from roadway center/m	ε_r	ε_θ	distance from roadway center/m	ε_r	ε_θ	distance from roadway center/m	ε_r	ε_θ	distance from roadway center/m	ε_r	ε_θ	distance from roadway center/m	ε_r	ε_θ
3	-0.00282	0.00166	3	-0.00377	0.00213	3	-0.0047	0.00255	3	-0.00557	0.00295	3	-0.00641	0.00332
3.001	-0.00281	0.00166	3.001	-0.00377	0.00212	3.001	-0.00469	0.00255	3.001	-0.00557	0.00295	3.001	-0.0064	0.00331
3.002	-0.00281	0.00165	3.002	-0.00377	0.00212	3.002	-0.00469	0.00255	3.002	-0.00556	0.00294	3.002	-0.0064	0.00331
3.003	-0.00281	0.00165	3.003	-0.00376	0.00212	3.003	-0.00468	0.00255	3.003	-0.00556	0.00294	3.003	-0.00639	0.00331
3.004	-0.00281	0.00165	3.004	-0.00376	0.00212	3.004	-0.00468	0.00254	3.004	-0.00555	0.00294	3.004	-0.00638	0.00331
3.005	-0.0028	0.00165	3.005	-0.00376	0.00212	3.005	-0.00467	0.00254	3.005	-0.00555	0.00294	3.005	-0.00638	0.0033
3.006	-0.0028	0.00165	3.006	-0.00375	0.00211	3.006	-0.00467	0.00254	3.006	-0.00554	0.00293	3.006	-0.00637	0.0033
3.007	-0.0028	0.00165	3.007	-0.00375	0.00211	3.007	-0.00466	0.00254	3.007	-0.00554	0.00293	3.007	-0.00636	0.0033
3.008	-0.0028	0.00165	3.008	-0.00375	0.00211	3.008	-0.00466	0.00254	3.008	-0.00553	0.00293	3.008	-0.00636	0.00329
3.009	-0.00279	0.00164	3.009	-0.00374	0.00211	3.009	-0.00466	0.00253	3.009	-0.00553	0.00293	3.009	-0.00635	0.00329
3.01	-0.00279	0.00164	3.01	-0.00374	0.00211	3.01	-0.00465	0.00253	3.01	-0.00552	0.00292	3.01	-0.00635	0.00329
3.011	-0.00279	0.00164	3.011	-0.00374	0.0021	3.011	-0.00465	0.00253	3.011	-0.00551	0.00292	3.011	-0.00634	0.00328
3.012	-0.00279	0.00164	3.012	-0.00373	0.0021	3.012	-0.00464	0.00253	3.012	-0.00551	0.00292	3.012	-0.00633	0.00328
3.013	-0.00278	0.00164	3.013	-0.00373	0.0021	3.013	-0.00464	0.00252	3.013	-0.0055	0.00291	3.013	-0.00633	0.00328
3.014	-0.00278	0.00164	3.014	-0.00373	0.0021	3.014	-0.00463	0.00252	3.014	-0.0055	0.00291	3.014	-0.00632	0.00327
3.015	-0.00278	0.00164	3.015	-0.00372	0.0021	3.015	-0.00463	0.00252	3.015	-0.00549	0.00291	3.015	-0.00631	0.00327
3.016	-0.00278	0.00163	3.016	-0.00372	0.00209	3.016	-0.00462	0.00252	3.016	-0.00549	0.00291	3.016	-0.00631	0.00327
3.017	-0.00277	0.00163	3.017	-0.00372	0.00209	3.017	-0.00462	0.00251	3.017	-0.00548	0.0029	3.017	-0.0063	0.00326
3.018	-0.00277	0.00163	3.018	-0.00371	0.00209	3.018	-0.00462	0.00251	3.018	-0.00548	0.0029	3.018	-0.0063	0.00326
3.019	-0.00277	0.00163	3.019	-0.00371	0.00209	3.019	-0.00461	0.00251	3.019	-0.00547	0.0029	3.019	-0.00629	0.00326
3.02	-0.00277	0.00163	3.02	-0.00371	0.00209	3.02	-0.00461	0.00251	3.02	-0.00547	0.00289	3.02	-0.00628	0.00325
3.021	-0.00276	0.00163	3.021	-0.0037	0.00208	3.021	-0.0046	0.0025	3.021	-0.00546	0.00289	3.021	-0.00628	0.00325
3.022	-0.00276	0.00163	3.022	-0.0037	0.00208	3.022	-0.0046	0.0025	3.022	-0.00546	0.00289	3.022	-0.00627	0.00325
3.023	-0.00276	0.00162	3.023	-0.0037	0.00208	3.023	-0.00459	0.0025	3.023	-0.00545	0.00289	3.023	-0.00627	0.00324
3.024	-0.00276	0.00162	3.024	-0.00369	0.00208	3.024	-0.00459	0.0025	3.024	-0.00545	0.00288	3.024	-0.00626	0.00324
3.025	-0.00275	0.00162	3.025	-0.00369	0.00208	3.025	-0.00459	0.00249	3.025	-0.00544	0.00288	3.025	-0.00625	0.00324
3.026	-0.00275	0.00162	3.026	-0.00368	0.00207	3.026	-0.00458	0.00249	3.026	-0.00544	0.00288	3.026	-0.00625	0.00324
3.027	-0.00275	0.00162	3.027	-0.00368	0.00207	3.027	-0.00458	0.00249	3.027	-0.00543	0.00288	3.027	-0.00624	0.00323
3.028	-0.00275	0.00162	3.028	-0.00368	0.00207	3.028	-0.00457	0.00249	3.028	-0.00543	0.00287	3.028	-0.00624	0.00323
3.029	-0.00274	0.00162	3.029	-0.00367	0.00207	3.029	-0.00457	0.00249	3.029	-0.00542	0.00287	3.029	-0.00623	0.00323
3.03	-0.00274	0.00161	3.03	-0.00367	0.00207	3.03	-0.00456	0.00248	3.03	-0.00542	0.00287	3.03	-0.00622	0.00322
3.031	-0.00274	0.00161	3.031	-0.00367	0.00207	3.031	-0.00456	0.00248	3.031	-0.00541	0.00286	3.031	-0.00622	0.00322
3.032	-0.00274	0.00161	3.032	-0.00366	0.00206	3.032	-0.00456	0.00248	3.032	-0.00541	0.00286	3.032	-0.00621	0.00322
3.033	-0.00273	0.00161	3.033	-0.00366	0.00206	3.033	-0.00455	0.00248	3.033	-0.0054	0.00286	3.033	-0.00621	0.00321
3.034	-0.00273	0.00161	3.034	-0.00366	0.00206	3.034	-0.00455	0.00247	3.034	-0.00539	0.00286	3.034	-0.0062	0.00321
3.035	-0.00273	0.00161	3.035	-0.00365	0.00206	3.035	-0.00454	0.00247	3.035	-0.00539	0.00285	3.035	-0.00619	0.00321
3.036	-0.00273	0.00161	3.036	-0.00365	0.00206	3.036	-0.00454	0.00247	3.036	-0.00538	0.00285	3.036	-0.00619	0.0032
3.037	-0.00272	0.0016	3.037	-0.00365	0.00205	3.037	-0.00453	0.00247	3.037	-0.00538	0.00285	3.037	-0.00618	0.0032
3.038	-0.00272	0.0016	3.038	-0.00364	0.00205	3.038	-0.00453	0.00246	3.038	-0.00537	0.00285	3.038	-0.00618	0.0032
3.039	-0.00272	0.0016	3.039	-0.00364	0.00205	3.039	-0.00453	0.00246	3.039	-0.00537	0.00284	3.039	-0.00617	0.00319
3.04	-0.00272	0.0016	3.04	-0.00364	0.00205	3.04	-0.00452	0.00246	3.04	-0.00536	0.00284	3.04	-0.00616	0.00319
3.041	-0.00271	0.0016	3.041	-0.00363	0.00205	3.041	-0.00452	0.00246	3.041	-0.00536	0.00284	3.041	-0.00616	0.00319
3.042	-0.00271	0.0016	3.042	-0.00363	0.00204	3.042	-0.00451	0.00246	3.042	-0.00535	0.00283	3.042	-0.00615	0.00319
3.043	-0.00271	0.0016	3.043	-0.00363	0.00204	3.043	-0.00451	0.00245	3.043	-0.00535	0.00283	3.043	-0.00615	0.00318
3.044	-0.00271	0.00159	3.044	-0.00362	0.00204	3.044	-0.00451	0.00245	3.044	-0.00534	0.00283	3.044	-0.00614	0.00318
3.045	-0.0027	0.00159	3.045	-0.00362	0.00204	3.045	-0.0045	0.00245	3.045	-0.00534	0.00283	3.045	-0.00613	0.00318
3.046	-0.0027	0.00159	3.046	-0.00362	0.00204	3.046	-0.0045	0.00245	3.046	-0.00533	0.00282	3.046	-0.00613	0.00317
3.047	-0.0027	0.00159	3.047	-0.00361	0.00204	3.047	-0.00449	0.00244	3.047	-0.00533	0.00282	3.047	-0.00612	0.00317
3.048	-0.0027	0.00159	3.048	-0.00361	0.00203	3.048	-0.00449	0.00244	3.048	-0.00532	0.00282	3.048	-0.00612	0.00317
3.049	-0.0027	0.00159	3.049	-0.00361	0.00203	3.049	-0.00448	0.00244	3.049	-0.00532	0.00282	3.049	-0.00611	0.00316
3.05	-0.00269	0.00159	3.05	-0.0036	0.00203	3.05	-0.00448	0.00244	3.05	-0.00531	0.00281	3.05	-0.0061	0.00316
3.051	-0.00269	0.00158	3.051	-0.0036	0.00203	3.051	-0.00448	0.00244	3.051	-0.00531	0.00281	3.051	-0.0061	0.00316
3.052	-0.00269	0.00158	3.052	-0.0036	0.00203	3.052	-0.00447	0.00243	3.052	-0.0053	0.00281	3.052	-0.00609	0.00316
3.053	-0.00269	0.00158	3.053	-0.0036	0.00202	3.053	-0.00447	0.00243	3.053	-0.0053	0.00281	3.053	-0.00609	0.00315
3.054	-0.00268	0.00158	3.054	-0.00359	0.00202	3.054	-0.00446	0.00243	3.054	-0.00529	0.0028	3.054	-0.00608	0.00315
3.055	-0.00268	0.00158	3.055	-0.00359	0.00202	3.055	-0.00446	0.00243	3.055	-0.00529	0.0028	3.055	-0.00608	0.00315
3.056	-0.00268	0.00158	3.056	-0.00359	0.00202	3.056	-0.00446	0.00242	3.056	-0.00528	0.0028	3.056	-0.00607	0.00314
3.057	-0.00268	0.00158	3.057	-0.00358	0.00202	3.057	-0.00445	0.00242	3.057	-0.00528	0.00279	3.057	-0.00606	0.00314
3.058	-0.00267	0.00157	3.058	-0.00358	0.00202	3.058	-0.00445	0.00242	3.058	-0.00527	0.00279	3.058	-0.00606	0.00314
3.059	-0.00267	0.00157	3.059	-0.00358	0.00201	3.059	-0.00444	0.00242	3.059	-0.00527	0.00279	3.059	-0.00605	0.00313
3.06	-0.00267	0.00157	3.06	-0.00357	0.00201	3.06	-0.00444	0.00241	3.06	-0.00526	0.00279	3.06	-0.00605	0.00313
3.061	-0.00267	0.00157	3.061	-0.00357	0.00201	3.061	-0.00443	0.00241	3.061	-0.00526	0.00278	3.061	-0.00604	0.00313
3.062	-0.00266	0.00157	3.062	-0.00357	0.00201	3.062	-0.00443	0.00241	3.062	-0.00525	0.00278	3.062	-0.00604	0.00313
3.063	-0.00266	0.00157	3.063	-0.00356	0.00201	3.063	-0.00443	0.00241	3.063	-0.00525	0.00278	3.063	-0.00603	0.00312
3.064	-0.00266	0.00157	3.064	-0.00356	0.002	3.064	-0.00442	0.00241	3.064	-0.00524	0.00278	3.064	-0.00602	0.00312
3.065	-0.00266	0.00156	3.065	-0.00356	0.002	3.065	-0.00442	0.0024	3.065	-0.00524	0.00277	3.065	-0.00602	0.00312
3.066	-0.00265	0.00156	3.066	-0.00355	0.002	3.066	-0.00441	0.0024	3.066	-0.00523	0.00277	3.066	-0.00601	0.00311
3.067	-0.00265	0.00156	3.067	-0.00355	0.002	3.067	-0.00441	0.0024	3.067	-0.00523	0.00277	3.067	-0.00601	0.00311
3.068	-0.00265	0.00156	3.068	-0.00355	0.002	3.068	-0.00441	0.0024	3.068	-0.00522	0.00277	3.068	-0.006	0.00311
3.069	-0.00265	0.00156	3.069	-0.00354	0.002	3.069	-0.0044	0.00239	3.069	-0.00522	0.00276	3.069	-0.00599	0.0031
3.07	-0.00265	0.00156	3.07	-0.00354	0.00199	3.07	-0.0044	0.00239	3.07	-0.00521	0.00276	3.07	-0.00599	0.0031
3.071	-0.00264	0.00156	3.071	-0.00354	0.00199	3.071	-0.00439	0.00239	3.071	-0.00521	0.00276	3.071	-0.00598	0.0031
3.072	-0.00264	0.00155	3.072	-0.00353	0.00199	3.072	-0.00439	0.00239	3.072	-0.0052	0.00276	3.072	-0.00598	0.0031
3.073	-0.00264	0.00155	3.073	-0.00353	0.00199	3.073	-0.00439	0.00239	3.073	-0.0052	0.00275	3.073	-0.00597	0.00309
3.074	-0.00264	0.00155	3.074	-0.00353	0.00199	3.074	-0.00438	0.00238	3.074	-0.00519	0.00275	3.074	-0.00597	0.00309
3.075	-0.00263	0.00155	3.075	-0.00352	0.00198	3.075	-0.00438	0.00238	3.075	-0.00519	0.00275	3.075	-0.00596	0.00309
3.076	-0.00263	0.00155	3.076	-0.00352	0.00198	3.076	-0.00437	0.00238	3.076	-0.00518	0.00274	3.076	-0.00595	0.00308
3.077	-0.00263	0.00155	3.077	-0.00352	0.00198	3.077	-0.00437	0.00238	3.077	-0.00518	0.00274	3.077	-0.00595	0.00308
3.078	-0.00263	0.00155	3.078	-0.00351	0.00198	3.078	-0.00437	0.00237	3.078	-0.00517	0.00274	3.078	-0.00594	0.00308
3.079	-0.00262	0.00155	3.079	-0.00351	0.00198	3.079	-0.00436	0.00237	3.079	-0.00517	0.00274	3.079	-0.00594	0.00307
3.08	-0.00262	0.00154	3.08	-0.00351	0.00198	3.08	-0.00436	0.00237	3.08	-0.00517	0.00273	3.08	-0.00593	0.00307
3.081	-0.00262	0.00154	3.081	-0.00351	0.00197	3.081	-0.00435	0.00237	3.081	-0.00516	0.00273	3.081	-0.00593	0.00307
3.082	-0.00262	0.00154	3.082	-0.0035	0.00197	3.082	-0.00435	0.00237	3.082	-0.00516	0.00273	3.082	-0.00592	0.00307
3.083	-0.00262	0.00154	3.083	-0.0035	0.00197	3.083	-0.00435	0.00236	3.083	-0.00515	0.00273	3.083	-0.00592	0.00306
3.084	-0.00261	0.00154	3.084	-0.0035	0.00197	3.084	-0.00434	0.00236	3.084	-0.00515	0.00272	3.084	-0.00591	0.00306
3.085	-0.00261	0.00154	3.085	-0.00349	0.00197	3.085	-0.00434	0.00236	3.085	-0.00514	0.00272	3.085	-0.0059	0.00306
3.086	-0.00261	0.00154	3.086	-0.00349	0.00196	3.086	-0.00433	0.00236	3.086	-0.00514	0.00272	3.086	-0.0059	0.00305
3.087	-0.00261	0.00153	3.087	-0.00349	0.00196	3.087	-0.00433	0.00236	3.087	-0.00513	0.00272	3.087	-0.00589	0.00305
3.088	-0.0026	0.00153	3.088	-0.00348	0.00196	3.088	-0.00433	0.00235	3.088	-0.00513	0.00271	3.088	-0.00589	0.00305
3.089	-0.0026	0.00153	3.089	-0.00348	0.00196	3.089	-0.00432	0.00235	3.089	-0.00512	0.00271	3.089	-0.00588	0.00305
3.09	-0.0026	0.00153	3.09	-0.00348	0.00196	3.09	-0.00432	0.00235	3.09	-0.00512	0.00271	3.09	-0.00588	0.00304
3.091	-0.0026	0.00153	3.091	-0.00347	0.00196	3.091	-0.00431	0.00235	3.091	-0.00511	0.00271	3.091	-0.00587	0.00304
3.092	-0.0026	0.00153	3.092	-0.00347	0.00195	3.092	-0.00431	0.00234	3.092	-0.00511	0.0027	3.092	-0.00586	0.00304
3.093	-0.00259	0.00153	3.093	-0.00347	0.00195	3.093	-0.00431	0.00234	3.093	-0.0051	0.0027	3.093	-0.00586	0.00303
3.094	-0.00259	0.00153	3.094	-0.00346	0.00195	3.094	-0.0043	0.00234	3.094	-0.0051	0.0027	3.094	-0.00585	0.00303
3.095	-0.00259	0.00152	3.095	-0.00346	0.00195	3.095	-0.0043	0.00234	3.095	-0.00509	0.0027	3.095	-0.00585	0.00303
3.096	-0.00259	0.00152	3.096	-0.00346	0.00195	3.096	-0.00429	0.00234	3.096	-0.00509	0.00269	3.096	-0.00584	0.00303
3.097	-0.00258	0.00152	3.097	-0.00346	0.00195	3.097	-0.00429	0.00233	3.097	-0.00508	0.00269	3.097	-0.00584	0.00302
3.098	-0.00258	0.00152	3.098	-0.00345	0.00194	3.098	-0.00429	0.00233	3.098	-0.00508	0.00269	3.098	-0.00583	0.00302
3.099	-0.00258	0.00152	3.099	-0.00345	0.00194	3.099	-0.00428	0.00233	3.099	-0.00507	0.00269	3.099	-0.00583	0.00302
3.1	-0.00258	0.00152	3.1	-0.00345	0.00194	3.1	-0.00428	0.00233	3.1	-0.00507	0.00268	3.1	-0.00582	0.00301
3.101	-0.00257	0.00152	3.101	-0.00344	0.00194	3.101	-0.00427	0.00233	3.101	-0.00506	0.00268	3.101	-0.00582	0.00301
3.102	-0.00257	0.00151	3.102	-0.00344	0.00194	3.102	-0.00427	0.00232	3.102	-0.00506	0.00268	3.102	-0.00581	0.00301
3.103	-0.00257	0.00151	3.103	-0.00344	0.00194	3.103	-0.00427	0.00232	3.103	-0.00506	0.00268	3.103	-0.0058	0.00301
3.104	-0.00257	0.00151	3.104	-0.00343	0.00193	3.104	-0.00426	0.00232	3.104	-0.00505	0.00267	3.104	-0.0058	0.003
3.105	-0.00257	0.00151	3.105	-0.00343	0.00193	3.105	-0.00426	0.00232	3.105	-0.00505	0.00267	3.105	-0.00579	0.003
3.106	-0.00256	0.00151	3.106	-0.00343	0.00193	3.106	-0.00425	0.00231	3.106	-0.00504	0.00267	3.106	-0.00579	0.003
3.107	-0.00256	0.00151	3.107	-0.00342	0.00193	3.107	-0.00425	0.00231	3.107	-0.00504	0.00267	3.107	-0.00578	0.00299
3.108	-0.00256	0.00151	3.108	-0.00342	0.00193	3.108	-0.00425	0.00231	3.108	-0.00503	0.00266	3.108	-0.00578	0.00299
3.109	-0.00256	0.00151	3.109	-0.00342	0.00192	3.109	-0.00424	0.00231	3.109	-0.00503	0.00266	3.109	-0.00577	0.00299
3.11	-0.00255	0.0015	3.11	-0.00342	0.00192	3.11	-0.00424	0.00231	3.11	-0.00502	0.00266	3.11	-0.00577	0.00299
3.111	-0.00255	0.0015	3.111	-0.00341	0.00192	3.111	-0.00424	0.0023	3.111	-0.00502	0.00266	3.111	-0.00576	0.00298
3.112	-0.00255	0.0015	3.112	-0.00341	0.00192	3.112	-0.00423	0.0023	3.112	-0.00501	0.00265	3.112	-0.00576	0.00298
3.113	-0.00255	0.0015	3.113	-0.00341	0.00192	3.113	-0.00423	0.0023	3.113	-0.00501	0.00265	3.113	-0.00575	0.00298
3.114	-0.00255	0.0015	3.114	-0.0034	0.00192	3.114	-0.00422	0.0023	3.114	-0.005	0.00265	3.114	-0.00574	0.00297
3.115	-0.00254	0.0015	3.115	-0.0034	0.00191	3.115	-0.00422	0.0023	3.115	-0.005	0.00265	3.115	-0.00574	0.00297
3.116	-0.00254	0.0015	3.116	-0.0034	0.00191	3.116	-0.00422	0.00229	3.116	-0.00499	0.00264	3.116	-0.00573	0.00297
3.117	-0.00254	0.0015	3.117	-0.00339	0.00191	3.117	-0.00421	0.00229	3.117	-0.00499	0.00264	3.117	-0.00573	0.00297
3.118	-0.00254	0.00149	3.118	-0.00339	0.00191	3.118	-0.00421	0.00229	3.118	-0.00499	0.00264	3.118	-0.00572	0.00296
3.119	-0.00253	0.00149	3.119	-0.00339	0.00191	3.119	-0.0042	0.00229	3.119	-0.00498	0.00264	3.119	-0.00572	0.00296
3.12	-0.00253	0.00149	3.12	-0.00338	0.00191	3.12	-0.0042	0.00229	3.12	-0.00498	0.00263	3.12	-0.00571	0.00296
3.121	-0.00253	0.00149	3.121	-0.00338	0.0019	3.121	-0.0042	0.00228	3.121	-0.00497	0.00263	3.121	-0.00571	0.00295
3.122	-0.00253	0.00149	3.122	-0.00338	0.0019	3.122	-0.00419	0.00228	3.122	-0.00497	0.00263	3.122	-0.0057	0.00295
3.123	-0.00253	0.00149	3.123	-0.00338	0.0019	3.123	-0.00419	0.00228	3.123	-0.00496	0.00263	3.123	-0.0057	0.00295
3.124	-0.00252	0.00149	3.124	-0.00337	0.0019	3.124	-0.00419	0.00228	3.124	-0.00496	0.00262	3.124	-0.00569	0.00295
3.125	-0.00252	0.00148	3.125	-0.00337	0.0019	3.125	-0.00418	0.00227	3.125	-0.00495	0.00262	3.125	-0.00569	0.00294
3.126	-0.00252	0.00148	3.126	-0.00337	0.0019	3.126	-0.00418	0.00227	3.126	-0.00495	0.00262	3.126	-0.00568	0.00294
3.127	-0.00252	0.00148	3.127	-0.00336	0.00189	3.127	-0.00417	0.00227	3.127	-0.00494	0.00262	3.127	-0.00567	0.00294
3.128	-0.00252	0.00148	3.128	-0.00336	0.00189	3.128	-0.00417	0.00227	3.128	-0.00494	0.00262	3.128	-0.00567	0.00294
3.129	-0.00251	0.00148	3.129	-0.00336	0.00189	3.129	-0.00417	0.00227	3.129	-0.00494	0.00261	3.129	-0.00566	0.00293
3.13	-0.00251	0.00148	3.13	-0.00335	0.00189	3.13	-0.00416	0.00226	3.13	-0.00493	0.00261	3.13	-0.00566	0.00293
3.131	-0.00251	0.00148	3.131	-0.00335	0.00189	3.131	-0.00416	0.00226	3.131	-0.00493	0.00261	3.131	-0.00565	0.00293
3.132	-0.00251	0.00148	3.132	-0.00335	0.00189	3.132	-0.00416	0.00226	3.132	-0.00492	0.00261	3.132	-0.00565	0.00292
3.133	-0.0025	0.00147	3.133	-0.00335	0.00188	3.133	-0.00415	0.00226	3.133	-0.00492	0.0026	3.133	-0.00564	0.00292
3.134	-0.0025	0.00147	3.134	-0.00334	0.00188	3.134	-0.00415	0.00226	3.134	-0.00491	0.0026	3.134	-0.00564	0.00292
3.135	-0.0025	0.00147	3.135	-0.00334	0.00188	3.135	-0.00414	0.00225	3.135	-0.00491	0.0026	3.135	-0.00563	0.00292
3.136	-0.0025	0.00147	3.136	-0.00334	0.00188	3.136	-0.00414	0.00225	3.136	-0.0049	0.0026	3.136	-0.00563	0.00291
3.137	-0.0025	0.00147	3.137	-0.00333	0.00188	3.137	-0.00414	0.00225	3.137	-0.0049	0.00259	3.137	-0.00562	0.00291
3.138	-0.00249	0.00147	3.138	-0.00333	0.00188	3.138	-0.00413	0.00225	3.138	-0.00489	0.00259	3.138	-0.00562	0.00291
3.139	-0.00249	0.00147	3.139	-0.00333	0.00187	3.139	-0.00413	0.00225	3.139	-0.00489	0.00259	3.139	-0.00561	0.00291
3.14	-0.00249	0.00147	3.14	-0.00333	0.00187	3.14	-0.00413	0.00224	3.14	-0.00489	0.00259	3.14	-0.00561	0.0029
3.141	-0.00249	0.00146	3.141	-0.00332	0.00187	3.141	-0.00412	0.00224	3.141	-0.00488	0.00258	3.141	-0.0056	0.0029
3.142	-0.00249	0.00146	3.142	-0.00332	0.00187	3.142	-0.00412	0.00224	3.142	-0.00488	0.00258	3.142	-0.0056	0.0029
3.143	-0.00248	0.00146	3.143	-0.00332	0.00187	3.143	-0.00411	0.00224	3.143	-0.00487	0.00258	3.143	-0.00559	0.00289
3.144	-0.00248	0.00146	3.144	-0.00331	0.00187	3.144	-0.00411	0.00224	3.144	-0.00487	0.00258	3.144	-0.00559	0.00289
3.145	-0.00248	0.00146	3.145	-0.00331	0.00186	3.145	-0.00411	0.00223	3.145	-0.00486	0.00257	3.145	-0.00558	0.00289
3.146	-0.00248	0.00146	3.146	-0.00331	0.00186	3.146	-0.0041	0.00223	3.146	-0.00486	0.00257	3.146	-0.00557	0.00289
3.147	-0.00247	0.00146	3.147	-0.0033	0.00186	3.147	-0.0041	0.00223	3.147	-0.00485	0.00257	3.147	-0.00557	0.00288
3.148	-0.00247	0.00146	3.148	-0.0033	0.00186	3.148	-0.0041	0.00223	3.148	-0.00485	0.00257	3.148	-0.00556	0.00288
3.149	-0.00247	0.00145	3.149	-0.0033	0.00186	3.149	-0.00409	0.00223	3.149	-0.00485	0.00257	3.149	-0.00556	0.00288
3.15	-0.00247	0.00145	3.15	-0.0033	0.00186	3.15	-0.00409	0.00222	3.15	-0.00484	0.00256	3.15	-0.00555	0.00288
3.151	-0.00247	0.00145	3.151	-0.00329	0.00185	3.151	-0.00408	0.00222	3.151	-0.00484	0.00256	3.151	-0.00555	0.00287
3.152	-0.00246	0.00145	3.152	-0.00329	0.00185	3.152	-0.00408	0.00222	3.152	-0.00483	0.00256	3.152	-0.00554	0.00287
3.153	-0.00246	0.00145	3.153	-0.00329	0.00185	3.153	-0.00408	0.00222	3.153	-0.00483	0.00256	3.153	-0.00554	0.00287
3.154	-0.00246	0.00145	3.154	-0.00328	0.00185	3.154	-0.00407	0.00222	3.154	-0.00482	0.00255	3.154	-0.00553	0.00287
3.155	-0.00246	0.00145	3.155	-0.00328	0.00185	3.155	-0.00407	0.00221	3.155	-0.00482	0.00255	3.155	-0.00553	0.00286
3.156	-0.00246	0.00145	3.156	-0.00328	0.00185	3.156	-0.00407	0.00221	3.156	-0.00481	0.00255	3.156	-0.00552	0.00286
3.157	-0.00245	0.00144	3.157	-0.00328	0.00184	3.157	-0.00406	0.00221	3.157	-0.00481	0.00255	3.157	-0.00552	0.00286
3.158	-0.00245	0.00144	3.158	-0.00327	0.00184	3.158	-0.00406	0.00221	3.158	-0.00481	0.00254	3.158	-0.00551	0.00285
3.159	-0.00245	0.00144	3.159	-0.00327	0.00184	3.159	-0.00406	0.00221	3.159	-0.0048	0.00254	3.159	-0.00551	0.00285
3.16	-0.00245	0.00144	3.16	-0.00327	0.00184	3.16	-0.00405	0.0022	3.16	-0.0048	0.00254	3.16	-0.0055	0.00285
3.161	-0.00245	0.00144	3.161	-0.00326	0.00184	3.161	-0.00405	0.0022	3.161	-0.00479	0.00254	3.161	-0.0055	0.00285
3.162	-0.00244	0.00144	3.162	-0.00326	0.00184	3.162	-0.00404	0.0022	3.162	-0.00479	0.00253	3.162	-0.00549	0.00284
3.163	-0.00244	0.00144	3.163	-0.00326	0.00183	3.163	-0.00404	0.0022	3.163	-0.00478	0.00253	3.163	-0.00549	0.00284
3.164	-0.00244	0.00144	3.164	-0.00326	0.00183	3.164	-0.00404	0.0022	3.164	-0.00478	0.00253	3.164	-0.00548	0.00284
3.165	-0.00244	0.00143	3.165	-0.00325	0.00183	3.165	-0.00403	0.00219	3.165	-0.00477	0.00253	3.165	-0.00548	0.00284
3.166	-0.00243	0.00143	3.166	-0.00325	0.00183	3.166	-0.00403	0.00219	3.166	-0.00477	0.00253	3.166	-0.00547	0.00283
3.167	-0.00243	0.00143	3.167	-0.00325	0.00183	3.167	-0.00403	0.00219	3.167	-0.00477	0.00252	3.167	-0.00547	0.00283
3.168	-0.00243	0.00143	3.168	-0.00324	0.00183	3.168	-0.00402	0.00219	3.168	-0.00476	0.00252	3.168	-0.00546	0.00283
3.169	-0.00243	0.00143	3.169	-0.00324	0.00183	3.169	-0.00402	0.00219	3.169	-0.00476	0.00252	3.169	-0.00546	0.00283
3.17	-0.00243	0.00143	3.17	-0.00324	0.00182	3.17	-0.00402	0.00218	3.17	-0.00475	0.00252	3.17	-0.00545	0.00282
3.171	-0.00242	0.00143	3.171	-0.00324	0.00182	3.171	-0.00401	0.00218	3.171	-0.00475	0.00251	3.171	-0.00545	0.00282
3.172	-0.00242	0.00143	3.172	-0.00323	0.00182	3.172	-0.00401	0.00218	3.172	-0.00474	0.00251	3.172	-0.00544	0.00282
3.173	-0.00242	0.00143	3.173	-0.00323	0.00182	3.173	-0.004	0.00218	3.173	-0.00474	0.00251	3.173	-0.00544	0.00282
3.174	-0.00242	0.00142	3.174	-0.00323	0.00182	3.174	-0.004	0.00218	3.174	-0.00474	0.00251	3.174	-0.00543	0.00281
3.175	-0.00242	0.00142	3.175	-0.00322	0.00182	3.175	-0.004	0.00217	3.175	-0.00473	0.0025	3.175	-0.00543	0.00281
3.176	-0.00241	0.00142	3.176	-0.00322	0.00181	3.176	-0.00399	0.00217	3.176	-0.00473	0.0025	3.176	-0.00542	0.00281
3.177	-0.00241	0.00142	3.177	-0.00322	0.00181	3.177	-0.00399	0.00217	3.177	-0.00472	0.0025	3.177	-0.00542	0.0028
3.178	-0.00241	0.00142	3.178	-0.00322	0.00181	3.178	-0.00399	0.00217	3.178	-0.00472	0.0025	3.178	-0.00541	0.0028
3.179	-0.00241	0.00142	3.179	-0.00321	0.00181	3.179	-0.00398	0.00217	3.179	-0.00471	0.0025	3.179	-0.00541	0.0028
3.18	-0.00241	0.00142	3.18	-0.00321	0.00181	3.18	-0.00398	0.00217	3.18	-0.00471	0.00249	3.18	-0.0054	0.0028
3.181	-0.0024	0.00142	3.181	-0.00321	0.00181	3.181	-0.00398	0.00216	3.181	-0.00471	0.00249	3.181	-0.0054	0.00279
3.182	-0.0024	0.00141	3.182	-0.0032	0.0018	3.182	-0.00397	0.00216	3.182	-0.0047	0.00249	3.182	-0.00539	0.00279

3.183	-0.0024	0.00141	3.183	-0.0032	0.0018	3.183	-0.00397	0.00216	3.183	-0.0047	0.00249	3.183	-0.00539	0.00279
3.184	-0.0024	0.00141	3.184	-0.0032	0.0018	3.184	-0.00397	0.00216	3.184	-0.00469	0.00248	3.184	-0.00538	0.00279
3.185	-0.0024	0.00141	3.185	-0.0032	0.0018	3.185	-0.00396	0.00216	3.185	-0.00469	0.00248	3.185	-0.00538	0.00278
3.186	-0.00239	0.00141	3.186	-0.00319	0.0018	3.186	-0.00396	0.00215	3.186	-0.00468	0.00248	3.186	-0.00537	0.00278
3.187	-0.00239	0.00141	3.187	-0.00319	0.0018	3.187	-0.00395	0.00215	3.187	-0.00468	0.00248	3.187	-0.00537	0.00278
3.188	-0.00239	0.00141	3.188	-0.00319	0.0018	3.188	-0.00395	0.00215	3.188	-0.00468	0.00248	3.188	-0.00536	0.00278
3.189	-0.00239	0.00141	3.189	-0.00319	0.00179	3.189	-0.00395	0.00215	3.189	-0.00467	0.00247	3.189	-0.00536	0.00277
3.19	-0.00239	0.0014	3.19	-0.00318	0.00179	3.19	-0.00394	0.00215	3.19	-0.00467	0.00247	3.19	-0.00535	0.00277
3.191	-0.00238	0.0014	3.191	-0.00318	0.00179	3.191	-0.00394	0.00214	3.191	-0.00466	0.00247	3.191	-0.00535	0.00277
3.192	-0.00238	0.0014	3.192	-0.00318	0.00179	3.192	-0.00394	0.00214	3.192	-0.00466	0.00247	3.192	-0.00534	0.00277
3.193	-0.00238	0.0014	3.193	-0.00317	0.00179	3.193	-0.00393	0.00214	3.193	-0.00465	0.00246	3.193	-0.00534	0.00276
3.194	-0.00238	0.0014	3.194	-0.00317	0.00179	3.194	-0.00393	0.00214	3.194	-0.00465	0.00246	3.194	-0.00533	0.00276
3.195	-0.00238	0.0014	3.195	-0.00317	0.00178	3.195	-0.00393	0.00214	3.195	-0.00465	0.00246	3.195	-0.00533	0.00276
3.196	-0.00237	0.0014	3.196	-0.00317	0.00178	3.196	-0.00392	0.00213	3.196	-0.00464	0.00246	3.196	-0.00532	0.00276
3.197	-0.00237	0.0014	3.197	-0.00316	0.00178	3.197	-0.00392	0.00213	3.197	-0.00464	0.00246	3.197	-0.00532	0.00275
3.198	-0.00237	0.0014	3.198	-0.00316	0.00178	3.198	-0.00392	0.00213	3.198	-0.00463	0.00245	3.198	-0.00531	0.00275
3.199	-0.00237	0.00139	3.199	-0.00316	0.00178	3.199	-0.00391	0.00213	3.199	-0.00463	0.00245	3.199	-0.00531	0.00275
3.2	-0.00237	0.00139	3.2	-0.00316	0.00178	3.2	-0.00391	0.00213	3.2	-0.00463	0.00245	3.2	-0.0053	0.00275
3.201	-0.00236	0.00139	3.201	-0.00315	0.00178	3.201	-0.00391	0.00213	3.201	-0.00462	0.00245	3.201	-0.0053	0.00274
3.202	-0.00236	0.00139	3.202	-0.00315	0.00177	3.202	-0.0039	0.00212	3.202	-0.00462	0.00244	3.202	-0.00529	0.00274
3.203	-0.00236	0.00139	3.203	-0.00315	0.00177	3.203	-0.0039	0.00212	3.203	-0.00461	0.00244	3.203	-0.00529	0.00274
3.204	-0.00236	0.00139	3.204	-0.00314	0.00177	3.204	-0.0039	0.00212	3.204	-0.00461	0.00244	3.204	-0.00528	0.00274
3.205	-0.00236	0.00139	3.205	-0.00314	0.00177	3.205	-0.00389	0.00212	3.205	-0.0046	0.00244	3.205	-0.00528	0.00273
3.206	-0.00235	0.00139	3.206	-0.00314	0.00177	3.206	-0.00389	0.00212	3.206	-0.0046	0.00244	3.206	-0.00527	0.00273
3.207	-0.00235	0.00138	3.207	-0.00314	0.00177	3.207	-0.00389	0.00211	3.207	-0.0046	0.00243	3.207	-0.00527	0.00273
3.208	-0.00235	0.00138	3.208	-0.00313	0.00176	3.208	-0.00388	0.00211	3.208	-0.00459	0.00243	3.208	-0.00526	0.00273
3.209	-0.00235	0.00138	3.209	-0.00313	0.00176	3.209	-0.00388	0.00211	3.209	-0.00459	0.00243	3.209	-0.00526	0.00272
3.21	-0.00235	0.00138	3.21	-0.00313	0.00176	3.21	-0.00387	0.00211	3.21	-0.00458	0.00243	3.21	-0.00526	0.00272
3.211	-0.00234	0.00138	3.211	-0.00313	0.00176	3.211	-0.00387	0.00211	3.211	-0.00458	0.00242	3.211	-0.00525	0.00272
3.212	-0.00234	0.00138	3.212	-0.00312	0.00176	3.212	-0.00387	0.0021	3.212	-0.00458	0.00242	3.212	-0.00525	0.00272
3.213	-0.00234	0.00138	3.213	-0.00312	0.00176	3.213	-0.00386	0.0021	3.213	-0.00457	0.00242	3.213	-0.00524	0.00271
3.214	-0.00234	0.00138	3.214	-0.00312	0.00176	3.214	-0.00386	0.0021	3.214	-0.00457	0.00242	3.214	-0.00524	0.00271
3.215	-0.00234	0.00138	3.215	-0.00311	0.00175	3.215	-0.00386	0.0021	3.215	-0.00456	0.00242	3.215	-0.00523	0.00271
3.216	-0.00233	0.00137	3.216	-0.00311	0.00175	3.216	-0.00385	0.0021	3.216	-0.00456	0.00241	3.216	-0.00523	0.00271
3.217	-0.00233	0.00137	3.217	-0.00311	0.00175	3.217	-0.00385	0.0021	3.217	-0.00456	0.00241	3.217	-0.00522	0.0027
3.218	-0.00233	0.00137	3.218	-0.00311	0.00175	3.218	-0.00385	0.00209	3.218	-0.00455	0.00241	3.218	-0.00522	0.0027
3.219	-0.00233	0.00137	3.219	-0.0031	0.00175	3.219	-0.00384	0.00209	3.219	-0.00455	0.00241	3.219	-0.00521	0.0027
3.22	-0.00233	0.00137	3.22	-0.0031	0.00175	3.22	-0.00384	0.00209	3.22	-0.00454	0.00241	3.22	-0.00521	0.0027
3.221	-0.00232	0.00137	3.221	-0.0031	0.00174	3.221	-0.00384	0.00209	3.221	-0.00454	0.0024	3.221	-0.0052	0.00269
3.222	-0.00232	0.00137	3.222	-0.0031	0.00174	3.222	-0.00383	0.00209	3.222	-0.00453	0.0024	3.222	-0.0052	0.00269
3.223	-0.00232	0.00137	3.223	-0.00309	0.00174	3.223	-0.00383	0.00208	3.223	-0.00453	0.0024	3.223	-0.00519	0.00269
3.224	-0.00232	0.00137	3.224	-0.00309	0.00174	3.224	-0.00383	0.00208	3.224	-0.00453	0.0024	3.224	-0.00519	0.00269
3.225	-0.00232	0.00136	3.225	-0.00309	0.00174	3.225	-0.00382	0.00208	3.225	-0.00452	0.00239	3.225	-0.00518	0.00268
3.226	-0.00231	0.00136	3.226	-0.00309	0.00174	3.226	-0.00382	0.00208	3.226	-0.00452	0.00239	3.226	-0.00518	0.00268
3.227	-0.00231	0.00136	3.227	-0.00308	0.00174	3.227	-0.00382	0.00208	3.227	-0.00451	0.00239	3.227	-0.00517	0.00268
3.228	-0.00231	0.00136	3.228	-0.00308	0.00173	3.228	-0.00381	0.00207	3.228	-0.00451	0.00239	3.228	-0.00517	0.00268
3.229	-0.00231	0.00136	3.229	-0.00308	0.00173	3.229	-0.00381	0.00207	3.229	-0.00451	0.00239	3.229	-0.00517	0.00267
3.23	-0.00231	0.00136	3.23	-0.00307	0.00173	3.23	-0.00381	0.00207	3.23	-0.0045	0.00238	3.23	-0.00516	0.00267
3.231	-0.0023	0.00136	3.231	-0.00307	0.00173	3.231	-0.0038	0.00207	3.231	-0.0045	0.00238	3.231	-0.00516	0.00267
3.232	-0.0023	0.00136	3.232	-0.00307	0.00173	3.232	-0.0038	0.00207	3.232	-0.00449	0.00238	3.232	-0.00515	0.00267
3.233	-0.0023	0.00135	3.233	-0.00307	0.00173	3.233	-0.0038	0.00207	3.233	-0.00449	0.00238	3.233	-0.00515	0.00266
3.234	-0.0023	0.00135	3.234	-0.00306	0.00173	3.234	-0.00379	0.00206	3.234	-0.00449	0.00238	3.234	-0.00514	0.00266
3.235	-0.0023	0.00135	3.235	-0.00306	0.00172	3.235	-0.00379	0.00206	3.235	-0.00448	0.00237	3.235	-0.00514	0.00266
3.236	-0.0023	0.00135	3.236	-0.00306	0.00172	3.236	-0.00379	0.00206	3.236	-0.00448	0.00237	3.236	-0.00513	0.00266
3.237	-0.00229	0.00135	3.237	-0.00306	0.00172	3.237	-0.00378	0.00206	3.237	-0.00447	0.00237	3.237	-0.00513	0.00266
3.238	-0.00229	0.00135	3.238	-0.00305	0.00172	3.238	-0.00378	0.00206	3.238	-0.00447	0.00237	3.238	-0.00512	0.00265
3.239	-0.00229	0.00135	3.239	-0.00305	0.00172	3.239	-0.00378	0.00206	3.239	-0.00447	0.00236	3.239	-0.00512	0.00265
3.24	-0.00229	0.00135	3.24	-0.00305	0.00172	3.24	-0.00377	0.00205	3.24	-0.00446	0.00236	3.24	-0.00511	0.00265
3.241	-0.00229	0.00135	3.241	-0.00305	0.00171	3.241	-0.00377	0.00205	3.241	-0.00446	0.00236	3.241	-0.00511	0.00265
3.242	-0.00228	0.00134	3.242	-0.00304	0.00171	3.242	-0.00377	0.00205	3.242	-0.00445	0.00236	3.242	-0.0051	0.00264
3.243	-0.00228	0.00134	3.243	-0.00304	0.00171	3.243	-0.00376	0.00205	3.243	-0.00445	0.00236	3.243	-0.0051	0.00264
3.244	-0.00228	0.00134	3.244	-0.00304	0.00171	3.244	-0.00376	0.00205	3.244	-0.00445	0.00235	3.244	-0.0051	0.00264
3.245	-0.00228	0.00134	3.245	-0.00304	0.00171	3.245	-0.00376	0.00204	3.245	-0.00444	0.00235	3.245	-0.00509	0.00264
3.246	-0.00228	0.00134	3.246	-0.00303	0.00171	3.246	-0.00375	0.00204	3.246	-0.00444	0.00235	3.246	-0.00509	0.00263
3.247	-0.00227	0.00134	3.247	-0.00303	0.00171	3.247	-0.00375	0.00204	3.247	-0.00443	0.00235	3.247	-0.00508	0.00263
3.248	-0.00227	0.00134	3.248	-0.00303	0.0017	3.248	-0.00375	0.00204	3.248	-0.00443	0.00235	3.248	-0.00508	0.00263
3.249	-0.00227	0.00134	3.249	-0.00302	0.0017	3.249	-0.00374	0.00204	3.249	-0.00443	0.00234	3.249	-0.00507	0.00263
3.25	-0.00227	0.00134	3.25	-0.00302	0.0017	3.25	-0.00374	0.00204	3.25	-0.00442	0.00234	3.25	-0.00507	0.00262
3.251	-0.00227	0.00133	3.251	-0.00302	0.0017	3.251	-0.00374	0.00203	3.251	-0.00442	0.00234	3.251	-0.00506	0.00262
3.252	-0.00226	0.00133	3.252	-0.00302	0.0017	3.252	-0.00373	0.00203	3.252	-0.00441	0.00234	3.252	-0.00506	0.00262
3.253	-0.00226	0.00133	3.253	-0.00301	0.0017	3.253	-0.00373	0.00203	3.253	-0.00441	0.00234	3.253	-0.00505	0.00262
3.254	-0.00226	0.00133	3.254	-0.00301	0.0017	3.254	-0.00373	0.00203	3.254	-0.00441	0.00233	3.254	-0.00505	0.00261
3.255	-0.00226	0.00133	3.255	-0.00301	0.00169	3.255	-0.00372	0.00203	3.255	-0.0044	0.00233	3.255	-0.00505	0.00261
3.256	-0.00226	0.00133	3.256	-0.00301	0.00169	3.256	-0.00372	0.00202	3.256	-0.0044	0.00233	3.256	-0.00504	0.00261
3.257	-0.00226	0.00133	3.257	-0.003	0.00169	3.257	-0.00372	0.00202	3.257	-0.0044	0.00233	3.257	-0.00504	0.00261
3.258	-0.00225	0.00133	3.258	-0.003	0.00169	3.258	-0.00372	0.00202	3.258	-0.00439	0.00232	3.258	-0.00503	0.00261
3.259	-0.00225	0.00133	3.259	-0.003	0.00169	3.259	-0.00371	0.00202	3.259	-0.00439	0.00232	3.259	-0.00503	0.0026
3.26	-0.00225	0.00132	3.26	-0.003	0.00169	3.26	-0.00371	0.00202	3.26	-0.00438	0.00232	3.26	-0.00502	0.0026
3.261	-0.00225	0.00132	3.261	-0.00299	0.00169	3.261	-0.00371	0.00202	3.261	-0.00438	0.00232	3.261	-0.00502	0.0026
3.262	-0.00225	0.00132	3.262	-0.00299	0.00168	3.262	-0.0037	0.00201	3.262	-0.00438	0.00232	3.262	-0.00501	0.0026
3.263	-0.00224	0.00132	3.263	-0.00299	0.00168	3.263	-0.0037	0.00201	3.263	-0.00437	0.00231	3.263	-0.00501	0.00259
3.264	-0.00224	0.00132	3.264	-0.00299	0.00168	3.264	-0.0037	0.00201	3.264	-0.00437	0.00231	3.264	-0.005	0.00259
3.265	-0.00224	0.00132	3.265	-0.00298	0.00168	3.265	-0.00369	0.00201	3.265	-0.00436	0.00231	3.265	-0.005	0.00259
3.266	-0.00224	0.00132	3.266	-0.00298	0.00168	3.266	-0.00369	0.00201	3.266	-0.00436	0.00231	3.266	-0.005	0.00259
3.267	-0.00224	0.00132	3.267	-0.00298	0.00168	3.267	-0.00369	0.00201	3.267	-0.00436	0.00231	3.267	-0.00499	0.00258
3.268	-0.00224	0.00132	3.268	-0.00298	0.00168	3.268	-0.00368	0.002	3.268	-0.00435	0.0023	3.268	-0.00499	0.00258
3.269	-0.00223	0.00131	3.269	-0.00297	0.00167	3.269	-0.00368	0.002	3.269	-0.00435	0.0023	3.269	-0.00498	0.00258
3.27	-0.00223	0.00131	3.27	-0.00297	0.00167	3.27	-0.00368	0.002	3.27	-0.00435	0.0023	3.27	-0.00498	0.00258
3.271	-0.00223	0.00131	3.271	-0.00297	0.00167	3.271	-0.00367	0.002	3.271	-0.00434	0.0023	3.271	-0.00497	0.00258
3.272	-0.00223	0.00131	3.272	-0.00297	0.00167	3.272	-0.00367	0.002	3.272	-0.00434	0.0023	3.272	-0.00497	0.00257
3.273	-0.00223	0.00131	3.273	-0.00296	0.00167	3.273	-0.00367	0.002	3.273	-0.00433	0.00229	3.273	-0.00496	0.00257
3.274	-0.00222	0.00131	3.274	-0.00296	0.00167	3.274	-0.00366	0.00199	3.274	-0.00433	0.00229	3.274	-0.00496	0.00257
3.275	-0.00222	0.00131	3.275	-0.00296	0.00167	3.275	-0.00366	0.00199	3.275	-0.00433	0.00229	3.275	-0.00496	0.00257
3.276	-0.00222	0.00131	3.276	-0.00296	0.00166	3.276	-0.00366	0.00199	3.276	-0.00432	0.00229	3.276	-0.00495	0.00256
3.277	-0.00222	0.00131	3.277	-0.00295	0.00166	3.277	-0.00365	0.00199	3.277	-0.00432	0.00229	3.277	-0.00495	0.00256
3.278	-0.00222	0.00131	3.278	-0.00295	0.00166	3.278	-0.00365	0.00199	3.278	-0.00431	0.00228	3.278	-0.00494	0.00256
3.279	-0.00221	0.0013	3.279	-0.00295	0.00166	3.279	-0.00365	0.00198	3.279	-0.00431	0.00228	3.279	-0.00494	0.00256
3.28	-0.00221	0.0013	3.28	-0.00295	0.00166	3.28	-0.00364	0.00198	3.28	-0.00431	0.00228	3.28	-0.00493	0.00255
3.281	-0.00221	0.0013	3.281	-0.00294	0.00166	3.281	-0.00364	0.00198	3.281	-0.0043	0.00228	3.281	-0.00493	0.00255
3.282	-0.00221	0.0013	3.282	-0.00294	0.00166	3.282	-0.00364	0.00198	3.282	-0.0043	0.00228	3.282	-0.00492	0.00255
3.283	-0.00221	0.0013	3.283	-0.00294	0.00165	3.283	-0.00364	0.00198	3.283	-0.0043	0.00227	3.283	-0.00492	0.00255
3.284	-0.00221	0.0013	3.284	-0.00294	0.00165	3.284	-0.00363	0.00198	3.284	-0.00429	0.00227	3.284	-0.00492	0.00255
3.285	-0.0022	0.0013	3.285	-0.00293	0.00165	3.285	-0.00363	0.00197	3.285	-0.00429	0.00227	3.285	-0.00491	0.00254
3.286	-0.0022	0.0013	3.286	-0.00293	0.00165	3.286	-0.00363	0.00197	3.286	-0.00428	0.00227	3.286	-0.00491	0.00254
3.287	-0.0022	0.0013	3.287	-0.00293	0.00165	3.287	-0.00362	0.00197	3.287	-0.00428	0.00227	3.287	-0.0049	0.00254
3.288	-0.0022	0.00129	3.288	-0.00293	0.00165	3.288	-0.00362	0.00197	3.288	-0.00428	0.00226	3.288	-0.0049	0.00254
3.289	-0.0022	0.00129	3.289	-0.00292	0.00165	3.289	-0.00362	0.00197	3.289	-0.00427	0.00226	3.289	-0.00489	0.00253
3.29	-0.00219	0.00129	3.29	-0.00292	0.00164	3.29	-0.00361	0.00197	3.29	-0.00427	0.00226	3.29	-0.00489	0.00253
3.291	-0.00219	0.00129	3.291	-0.00292	0.00164	3.291	-0.00361	0.00196	3.291	-0.00427	0.00226	3.291	-0.00489	0.00253
3.292	-0.00219	0.00129	3.292	-0.00292	0.00164	3.292	-0.00361	0.00196	3.292	-0.00426	0.00226	3.292	-0.00488	0.00253
3.293	-0.00219	0.00129	3.293	-0.00291	0.00164	3.293	-0.0036	0.00196	3.293	-0.00426	0.00225	3.293	-0.00488	0.00253
3.294	-0.00219	0.00129	3.294	-0.00291	0.00164	3.294	-0.0036	0.00196	3.294	-0.00425	0.00225	3.294	-0.00487	0.00252
3.295	-0.00219	0.00129	3.295	-0.00291	0.00164	3.295	-0.0036	0.00196	3.295	-0.00425	0.00225	3.295	-0.00487	0.00252
3.296	-0.00218	0.00129	3.296	-0.00291	0.00164	3.296	-0.00359	0.00196	3.296	-0.00425	0.00225	3.296	-0.00486	0.00252
3.297	-0.00218	0.00128	3.297	-0.0029	0.00164	3.297	-0.00359	0.00195	3.297	-0.00424	0.00225	3.297	-0.00486	0.00252
3.298	-0.00218	0.00128	3.298	-0.0029	0.00163	3.298	-0.00359	0.00195	3.298	-0.00424	0.00224	3.298	-0.00485	0.00251
3.299	-0.00218	0.00128	3.299	-0.0029	0.00163	3.299	-0.00359	0.00195	3.299	-0.00424	0.00224	3.299	-0.00485	0.00251
3.3	-0.00218	0.00128	3.3	-0.0029	0.00163	3.3	-0.00358	0.00195	3.3	-0.00423	0.00224	3.3	-0.00485	0.00251
3.301	-0.00218	0.00128	3.301	-0.00289	0.00163	3.301	-0.00358	0.00195	3.301	-0.00423	0.00224	3.301	-0.00484	0.00251
3.302	-0.00217	0.00128	3.302	-0.00289	0.00163	3.302	-0.00358	0.00195	3.302	-0.00422	0.00224	3.302	-0.00484	0.0025
3.303	-0.00217	0.00128	3.303	-0.00289	0.00163	3.303	-0.00357	0.00194	3.303	-0.00422	0.00223	3.303	-0.00483	0.0025
3.304	-0.00217	0.00128	3.304	-0.00289	0.00163	3.304	-0.00357	0.00194	3.304	-0.00422	0.00223	3.304	-0.00483	0.0025
3.305	-0.00217	0.00128	3.305	-0.00288	0.00162	3.305	-0.00357	0.00194	3.305	-0.00421	0.00223	3.305	-0.00482	0.0025
3.306	-0.00217	0.00128	3.306	-0.00288	0.00162	3.306	-0.00356	0.00194	3.306	-0.00421	0.00223	3.306	-0.00482	0.0025
3.307	-0.00216	0.00127	3.307	-0.00288	0.00162	3.307	-0.00356	0.00194	3.307	-0.00421	0.00223	3.307	-0.00482	0.00249
3.308	-0.00216	0.00127	3.308	-0.00288	0.00162	3.308	-0.00356	0.00194	3.308	-0.0042	0.00222	3.308	-0.00481	0.00249
3.309	-0.00216	0.00127	3.309	-0.00288	0.00162	3.309	-0.00355	0.00193	3.309	-0.0042	0.00222	3.309	-0.00481	0.00249
3.31	-0.00216	0.00127	3.31	-0.00287	0.00162	3.31	-0.00355	0.00193	3.31	-0.0042	0.00222	3.31	-0.0048	0.00249
3.311	-0.00216	0.00127	3.311	-0.00287	0.00162	3.311	-0.00355	0.00193	3.311	-0.00419	0.00222	3.311	-0.0048	0.00248
3.312	-0.00216	0.00127	3.312	-0.00287	0.00161	3.312	-0.00355	0.00193	3.312	-0.00419	0.00222	3.312	-0.00479	0.00248
3.313	-0.00215	0.00127	3.313	-0.00287	0.00161	3.313	-0.00354	0.00193	3.313	-0.00418	0.00222	3.313	-0.00479	0.00248
3.314	-0.00215	0.00127	3.314	-0.00286	0.00161	3.314	-0.00354	0.00193	3.314	-0.00418	0.00221	3.314	-0.00479	0.00248
3.315	-0.00215	0.00127	3.315	-0.00286	0.00161	3.315	-0.00354	0.00192	3.315	-0.00418	0.00221	3.315	-0.00478	0.00248
3.316	-0.00215	0.00127	3.316	-0.00286	0.00161	3.316	-0.00353	0.00192	3.316	-0.00417	0.00221	3.316	-0.00478	0.00247
3.317	-0.00215	0.00126	3.317	-0.00286	0.00161	3.317	-0.00353	0.00192	3.317	-0.00417	0.00221	3.317	-0.00477	0.00247
3.318	-0.00215	0.00126	3.318	-0.00285	0.00161	3.318	-0.00353	0.00192	3.318	-0.00417	0.00221	3.318	-0.00477	0.00247
3.319	-0.00214	0.00126	3.319	-0.00285	0.00161	3.319	-0.00352	0.00192	3.319	-0.00416	0.0022	3.319	-0.00477	0.00247
3.32	-0.00214	0.00126	3.32	-0.00285	0.0016	3.32	-0.00352	0.00192	3.32	-0.00416	0.0022	3.32	-0.00476	0.00247
3.321	-0.00214	0.00126	3.321	-0.00285	0.0016	3.321	-0.00352	0.00191	3.321	-0.00416	0.0022	3.321	-0.00476	0.00246
3.322	-0.00214	0.00126	3.322	-0.00284	0.0016	3.322	-0.00352	0.00191	3.322	-0.00415	0.0022	3.322	-0.00475	0.00246
3.323	-0.00214	0.00126	3.323	-0.00284	0.0016	3.323	-0.00351	0.00191	3.323	-0.00415	0.0022	3.323	-0.00475	0.00246
3.324	-0.00213	0.00126	3.324	-0.00284	0.0016	3.324	-0.00351	0.00191	3.324	-0.00414	0.00219	3.324	-0.00474	0.00246
3.325	-0.00213	0.00126	3.325	-0.00284	0.0016	3.325	-0.00351	0.00191	3.325	-0.00414	0.00219	3.325	-0.00474	0.00245
3.326	-0.00213	0.00125	3.326	-0.00283	0.0016	3.326	-0.0035	0.00191	3.326	-0.00414	0.00219	3.326	-0.00474	0.00245
3.327	-0.00213	0.00125	3.327	-0.00283	0.00159	3.327	-0.0035	0.0019	3.327	-0.00413	0.00219	3.327	-0.00473	0.00245
3.328	-0.00213	0.00125	3.328	-0.00283	0.00159	3.328	-0.0035	0.0019	3.328	-0.00413	0.00219	3.328	-0.00473	0.00245
3.329	-0.00213	0.00125	3.329	-0.00283	0.00159	3.329	-0.00349	0.0019	3.329	-0.00413	0.00218	3.329	-0.00472	0.00245
3.33	-0.00212	0.00125	3.33	-0.00282	0.00159	3.33	-0.00349	0.0019	3.33	-0.00412	0.00218	3.33	-0.00472	0.00244
3.331	-0.00212	0.00125	3.331	-0.00282	0.00159	3.331	-0.00349	0.0019	3.331	-0.00412	0.00218	3.331	-0.00472	0.00244
3.332	-0.00212	0.00125	3.332	-0.00282	0.00159	3.332	-0.00349	0.0019	3.332	-0.00412	0.00218	3.332	-0.00471	0.00244
3.333	-0.00212	0.00125	3.333	-0.00282	0.00159	3.333	-0.00348	0.00189	3.333	-0.00411	0.00218	3.333	-0.00471	0.00244
3.334	-0.00212	0.00125	3.334	-0.00282	0.00159	3.334	-0.00348	0.00189	3.334	-0.00411	0.00218	3.334	-0.0047	0.00244
3.335	-0.00212	0.00125	3.335	-0.00281	0.00158	3.335	-0.00348	0.00189	3.335	-0.0041	0.00217	3.335	-0.0047	0.00243
3.336	-0.00211	0.00124	3.336	-0.00281	0.00158	3.336	-0.00347	0.00189	3.336	-0.0041	0.00217	3.336	-0.00469	0.00243
3.337	-0.00211	0.00124	3.337	-0.00281	0.00158	3.337	-0.00347	0.00189	3.337	-0.0041	0.00217	3.337	-0.00469	0.00243
3.338	-0.00211	0.00124	3.338	-0.00281	0.00158	3.338	-0.00347	0.00189	3.338	-0.00409	0.00217	3.338	-0.00469	0.00243
3.339	-0.00211	0.00124	3.339	-0.0028	0.00158	3.339	-0.00347	0.00189	3.339	-0.00409	0.00217	3.339	-0.00468	0.00242
3.34	-0.00211	0.00124	3.34	-0.0028	0.00158	3.34	-0.00346	0.00188	3.34	-0.00409	0.00216	3.34	-0.00468	0.00242
3.341	-0.00211	0.00124	3.341	-0.0028	0.00158	3.341	-0.00346	0.00188	3.341	-0.00408	0.00216	3.341	-0.00467	0.00242
3.342	-0.0021	0.00124	3.342	-0.0028	0.00157	3.342	-0.00346	0.00188	3.342	-0.00408	0.00216	3.342	-0.00467	0.00242
3.343	-0.0021	0.00124	3.343	-0.00279	0.00157	3.343	-0.00345	0.00188	3.343	-0.00408	0.00216	3.343	-0.00467	0.00242
3.344	-0.0021	0.00124	3.344	-0.00279	0.00157	3.344	-0.00345	0.00188	3.344	-0.00407	0.00216	3.344	-0.00466	0.00241
3.345	-0.0021	0.00124	3.345	-0.00279	0.00157	3.345	-0.00345	0.00188	3.345	-0.00407	0.00215	3.345	-0.00466	0.00241
3.346	-0.0021	0.00123	3.346	-0.00279	0.00157	3.346	-0.00344	0.00187	3.346	-0.00407	0.00215	3.346	-0.00465	0.00241
3.347	-0.0021	0.00123	3.347	-0.00279	0.00157	3.347	-0.00344	0.00187	3.347	-0.00406	0.00215	3.347	-0.00465	0.00241
3.348	-0.00209	0.00123	3.348	-0.00278	0.00157	3.348	-0.00344	0.00187	3.348	-0.00406	0.00215	3.348	-0.00465	0.00241
3.349	-0.00209	0.00123	3.349	-0.00278	0.00157	3.349	-0.00344	0.00187	3.349	-0.00406	0.00215	3.349	-0.00464	0.0024
3.35	-0.00209	0.00123	3.35	-0.00278	0.00156	3.35	-0.00343	0.00187	3.35	-0.00405	0.00215	3.35	-0.00464	0.0024
3.351	-0.00209	0.00123	3.351	-0.00278	0.00156	3.351	-0.00343	0.00187	3.351	-0.00405	0.00214	3.351	-0.00463	0.0024
3.352	-0.00209	0.00123	3.352	-0.00277	0.00156	3.352	-0.00343	0.00186	3.352	-0.00405	0.00214	3.352	-0.00463	0.0024
3.353	-0.00209	0.00123	3.353	-0.00277	0.00156	3.353	-0.00342	0.00186	3.353	-0.00404	0.00214	3.353	-0.00462	0.00239
3.354	-0.00208	0.00123	3.354	-0.00277	0.00156	3.354	-0.00342	0.00186	3.354	-0.00404	0.00214	3.354	-0.00462	0.00239
3.355	-0.00208	0.00123	3.355	-0.00277	0.00156	3.355	-0.00342	0.00186	3.355	-0.00403	0.00214	3.355	-0.00462	0.00239
3.356	-0.00208	0.00122	3.356	-0.00276	0.00156	3.356	-0.00342	0.00186	3.356	-0.00403	0.00213	3.356	-0.00461	0.00239
3.357	-0.00208	0.00122	3.357	-0.00276	0.00156	3.357	-0.00341	0.00186	3.357	-0.00403	0.00213	3.357	-0.00461	0.00239
3.358	-0.00208	0.00122	3.358	-0.00276	0.00155	3.358	-0.00341	0.00186	3.358	-0.00402	0.00213	3.358	-0.0046	0.00238
3.359	-0.00208	0.00122	3.359	-0.00276	0.00155	3.359	-0.00341	0.00185	3.359	-0.00402	0.00213	3.359	-0.0046	0.00238
3.36	-0.00207	0.00122	3.36	-0.00276	0.00155	3.36	-0.0034	0.00185	3.36	-0.00402	0.00213	3.36	-0.0046	0.00238
3.361	-0.00207	0.00122	3.361	-0.00275	0.00155	3.361	-0.0034	0.00185	3.361	-0.00401	0.00212	3.361	-0.00459	0.00238
3.362	-0.00207	0.00122	3.362	-0.00275	0.00155	3.362	-0.0034	0.00185	3.362	-0.00401	0.00212	3.362	-0.00459	0.00238
3.363	-0.00207	0.00122	3.363	-0.00275	0.00155	3.363	-0.0034	0.00185	3.363	-0.00401	0.00212	3.363	-0.00458	0.00237
3.364	-0.00207	0.00122	3.364	-0.00275	0.00155	3.364	-0.00339	0.00185	3.364	-0.004	0.00212	3.364	-0.00458	0.00237
3.365	-0.00207	0.00122	3.365	-0.00274	0.00155	3.365	-0.00339	0.00184	3.365	-0.004	0.00212	3.365	-0.00458	0.00237
3.366	-0.00206	0.00122	3.366	-0.00274	0.00154	3.366	-0.00339	0.00184	3.366	-0.004	0.00212	3.366	-0.00457	0.00237
3.367	-0.00206	0.00121	3.367	-0.00274	0.00154	3.367	-0.00338	0.00184	3.367	-0.00399	0.00211	3.367	-0.00457	0.00237

3.368	-0.00206	0.00121	3.368	-0.00274	0.00154	3.368	-0.00338	0.00184	3.368	-0.00399	0.00211	3.368	-0.00456	0.00236
3.369	-0.00206	0.00121	3.369	-0.00274	0.00154	3.369	-0.00338	0.00184	3.369	-0.00399	0.00211	3.369	-0.00456	0.00236
3.37	-0.00206	0.00121	3.37	-0.00273	0.00154	3.37	-0.00338	0.00184	3.37	-0.00398	0.00211	3.37	-0.00456	0.00236
3.371	-0.00206	0.00121	3.371	-0.00273	0.00154	3.371	-0.00337	0.00183	3.371	-0.00398	0.00211	3.371	-0.00455	0.00236
3.372	-0.00205	0.00121	3.372	-0.00273	0.00154	3.372	-0.00337	0.00183	3.372	-0.00398	0.00211	3.372	-0.00455	0.00236
3.373	-0.00205	0.00121	3.373	-0.00273	0.00153	3.373	-0.00337	0.00183	3.373	-0.00397	0.0021	3.373	-0.00455	0.00235
3.374	-0.00205	0.00121	3.374	-0.00272	0.00153	3.374	-0.00336	0.00183	3.374	-0.00397	0.0021	3.374	-0.00454	0.00235
3.375	-0.00205	0.00121	3.375	-0.00272	0.00153	3.375	-0.00336	0.00183	3.375	-0.00397	0.0021	3.375	-0.00454	0.00235
3.376	-0.00205	0.00121	3.376	-0.00272	0.00153	3.376	-0.00336	0.00183	3.376	-0.00396	0.0021	3.376	-0.00453	0.00235
3.377	-0.00205	0.0012	3.377	-0.00272	0.00153	3.377	-0.00336	0.00183	3.377	-0.00396	0.0021	3.377	-0.00453	0.00235
3.378	-0.00204	0.0012	3.378	-0.00271	0.00153	3.378	-0.00335	0.00182	3.378	-0.00396	0.00209	3.378	-0.00453	0.00234
3.379	-0.00204	0.0012	3.379	-0.00271	0.00153	3.379	-0.00335	0.00182	3.379	-0.00395	0.00209	3.379	-0.00452	0.00234
3.38	-0.00204	0.0012	3.38	-0.00271	0.00153	3.38	-0.00335	0.00182	3.38	-0.00395	0.00209	3.38	-0.00452	0.00234
3.381	-0.00204	0.0012	3.381	-0.00271	0.00152	3.381	-0.00334	0.00182	3.381	-0.00395	0.00209	3.381	-0.00451	0.00234
3.382	-0.00204	0.0012	3.382	-0.00271	0.00152	3.382	-0.00334	0.00182	3.382	-0.00394	0.00209	3.382	-0.00451	0.00234
3.383	-0.00204	0.0012	3.383	-0.0027	0.00152	3.383	-0.00334	0.00182	3.383	-0.00394	0.00209	3.383	-0.00451	0.00233
3.384	-0.00203	0.0012	3.384	-0.0027	0.00152	3.384	-0.00334	0.00181	3.384	-0.00394	0.00208	3.384	-0.0045	0.00233
3.385	-0.00203	0.0012	3.385	-0.0027	0.00152	3.385	-0.00333	0.00181	3.385	-0.00393	0.00208	3.385	-0.0045	0.00233
3.386	-0.00203	0.0012	3.386	-0.0027	0.00152	3.386	-0.00333	0.00181	3.386	-0.00393	0.00208	3.386	-0.00449	0.00233
3.387	-0.00203	0.00119	3.387	-0.00269	0.00152	3.387	-0.00333	0.00181	3.387	-0.00393	0.00208	3.387	-0.00449	0.00233
3.388	-0.00203	0.00119	3.388	-0.00269	0.00152	3.388	-0.00332	0.00181	3.388	-0.00392	0.00208	3.388	-0.00449	0.00232
3.389	-0.00203	0.00119	3.389	-0.00269	0.00151	3.389	-0.00332	0.00181	3.389	-0.00392	0.00207	3.389	-0.00448	0.00232
3.39	-0.00202	0.00119	3.39	-0.00269	0.00151	3.39	-0.00332	0.00181	3.39	-0.00392	0.00207	3.39	-0.00448	0.00232
3.391	-0.00202	0.00119	3.391	-0.00269	0.00151	3.391	-0.00332	0.0018	3.391	-0.00391	0.00207	3.391	-0.00447	0.00232
3.392	-0.00202	0.00119	3.392	-0.00268	0.00151	3.392	-0.00331	0.0018	3.392	-0.00391	0.00207	3.392	-0.00447	0.00232
3.393	-0.00202	0.00119	3.393	-0.00268	0.00151	3.393	-0.00331	0.0018	3.393	-0.00391	0.00207	3.393	-0.00447	0.00231
3.394	-0.00202	0.00119	3.394	-0.00268	0.00151	3.394	-0.00331	0.0018	3.394	-0.0039	0.00207	3.394	-0.00446	0.00231
3.395	-0.00202	0.00119	3.395	-0.00268	0.00151	3.395	-0.00331	0.0018	3.395	-0.0039	0.00206	3.395	-0.00446	0.00231
3.396	-0.00201	0.00119	3.396	-0.00268	0.00151	3.396	-0.0033	0.0018	3.396	-0.0039	0.00206	3.396	-0.00446	0.00231
3.397	-0.00201	0.00119	3.397	-0.00267	0.00151	3.397	-0.0033	0.0018	3.397	-0.00389	0.00206	3.397	-0.00445	0.00231
3.398	-0.00201	0.00118	3.398	-0.00267	0.0015	3.398	-0.0033	0.00179	3.398	-0.00389	0.00206	3.398	-0.00445	0.0023
3.399	-0.00201	0.00118	3.399	-0.00267	0.0015	3.399	-0.00329	0.00179	3.399	-0.00389	0.00206	3.399	-0.00444	0.0023
3.4	-0.00201	0.00118	3.4	-0.00267	0.0015	3.4	-0.00329	0.00179	3.4	-0.00388	0.00206	3.4	-0.00444	0.0023
3.401	-0.00201	0.00118	3.401	-0.00266	0.0015	3.401	-0.00329	0.00179	3.401	-0.00388	0.00205	3.401	-0.00444	0.0023
3.402	-0.00201	0.00118	3.402	-0.00266	0.0015	3.402	-0.00329	0.00179	3.402	-0.00388	0.00205	3.402	-0.00443	0.0023
3.403	-0.002	0.00118	3.403	-0.00266	0.0015	3.403	-0.00328	0.00179	3.403	-0.00387	0.00205	3.403	-0.00443	0.00229
3.404	-0.002	0.00118	3.404	-0.00266	0.0015	3.404	-0.00328	0.00178	3.404	-0.00387	0.00205	3.404	-0.00442	0.00229
3.405	-0.002	0.00118	3.405	-0.00266	0.0015	3.405	-0.00328	0.00178	3.405	-0.00387	0.00205	3.405	-0.00442	0.00229
3.406	-0.002	0.00118	3.406	-0.00265	0.00149	3.406	-0.00328	0.00178	3.406	-0.00386	0.00204	3.406	-0.00442	0.00229
3.407	-0.002	0.00118	3.407	-0.00265	0.00149	3.407	-0.00327	0.00178	3.407	-0.00386	0.00204	3.407	-0.00441	0.00229
3.408	-0.002	0.00118	3.408	-0.00265	0.00149	3.408	-0.00327	0.00178	3.408	-0.00386	0.00204	3.408	-0.00441	0.00228
3.409	-0.00199	0.00117	3.409	-0.00265	0.00149	3.409	-0.00327	0.00178	3.409	-0.00385	0.00204	3.409	-0.00441	0.00228
3.41	-0.00199	0.00117	3.41	-0.00264	0.00149	3.41	-0.00326	0.00178	3.41	-0.00385	0.00204	3.41	-0.0044	0.00228
3.411	-0.00199	0.00117	3.411	-0.00264	0.00149	3.411	-0.00326	0.00177	3.411	-0.00385	0.00204	3.411	-0.0044	0.00228
3.412	-0.00199	0.00117	3.412	-0.00264	0.00149	3.412	-0.00326	0.00177	3.412	-0.00384	0.00203	3.412	-0.00439	0.00228
3.413	-0.00199	0.00117	3.413	-0.00264	0.00149	3.413	-0.00326	0.00177	3.413	-0.00384	0.00203	3.413	-0.00439	0.00227
3.414	-0.00199	0.00117	3.414	-0.00264	0.00148	3.414	-0.00325	0.00177	3.414	-0.00384	0.00203	3.414	-0.00439	0.00227
3.415	-0.00198	0.00117	3.415	-0.00263	0.00148	3.415	-0.00325	0.00177	3.415	-0.00383	0.00203	3.415	-0.00438	0.00227
3.416	-0.00198	0.00117	3.416	-0.00263	0.00148	3.416	-0.00325	0.00177	3.416	-0.00383	0.00203	3.416	-0.00438	0.00227
3.417	-0.00198	0.00117	3.417	-0.00263	0.00148	3.417	-0.00325	0.00177	3.417	-0.00383	0.00203	3.417	-0.00438	0.00227
3.418	-0.00198	0.00117	3.418	-0.00263	0.00148	3.418	-0.00324	0.00176	3.418	-0.00382	0.00202	3.418	-0.00437	0.00226
3.419	-0.00198	0.00116	3.419	-0.00263	0.00148	3.419	-0.00324	0.00176	3.419	-0.00382	0.00202	3.419	-0.00437	0.00226
3.42	-0.00198	0.00116	3.42	-0.00262	0.00148	3.42	-0.00324	0.00176	3.42	-0.00382	0.00202	3.42	-0.00436	0.00226
3.421	-0.00198	0.00116	3.421	-0.00262	0.00148	3.421	-0.00323	0.00176	3.421	-0.00381	0.00202	3.421	-0.00436	0.00226
3.422	-0.00197	0.00116	3.422	-0.00262	0.00147	3.422	-0.00323	0.00176	3.422	-0.00381	0.00202	3.422	-0.00436	0.00226
3.423	-0.00197	0.00116	3.423	-0.00262	0.00147	3.423	-0.00323	0.00176	3.423	-0.00381	0.00202	3.423	-0.00435	0.00225
3.424	-0.00197	0.00116	3.424	-0.00261	0.00147	3.424	-0.00323	0.00176	3.424	-0.0038	0.00201	3.424	-0.00435	0.00225
3.425	-0.00197	0.00116	3.425	-0.00261	0.00147	3.425	-0.00322	0.00175	3.425	-0.0038	0.00201	3.425	-0.00435	0.00225
3.426	-0.00197	0.00116	3.426	-0.00261	0.00147	3.426	-0.00322	0.00175	3.426	-0.0038	0.00201	3.426	-0.00434	0.00225
3.427	-0.00197	0.00116	3.427	-0.00261	0.00147	3.427	-0.00322	0.00175	3.427	-0.00379	0.00201	3.427	-0.00434	0.00225
3.428	-0.00196	0.00116	3.428	-0.00261	0.00147	3.428	-0.00322	0.00175	3.428	-0.00379	0.00201	3.428	-0.00433	0.00224
3.429	-0.00196	0.00116	3.429	-0.0026	0.00147	3.429	-0.00321	0.00175	3.429	-0.00379	0.00201	3.429	-0.00433	0.00224
3.43	-0.00196	0.00115	3.43	-0.0026	0.00147	3.43	-0.00321	0.00175	3.43	-0.00378	0.002	3.43	-0.00433	0.00224
3.431	-0.00196	0.00115	3.431	-0.0026	0.00146	3.431	-0.00321	0.00175	3.431	-0.00378	0.002	3.431	-0.00432	0.00224
3.432	-0.00196	0.00115	3.432	-0.0026	0.00146	3.432	-0.00321	0.00174	3.432	-0.00378	0.002	3.432	-0.00432	0.00224
3.433	-0.00196	0.00115	3.433	-0.0026	0.00146	3.433	-0.0032	0.00174	3.433	-0.00378	0.002	3.433	-0.00432	0.00223
3.434	-0.00196	0.00115	3.434	-0.00259	0.00146	3.434	-0.0032	0.00174	3.434	-0.00377	0.002	3.434	-0.00431	0.00223
3.435	-0.00195	0.00115	3.435	-0.00259	0.00146	3.435	-0.0032	0.00174	3.435	-0.00377	0.002	3.435	-0.00431	0.00223
3.436	-0.00195	0.00115	3.436	-0.00259	0.00146	3.436	-0.00319	0.00174	3.436	-0.00377	0.00199	3.436	-0.00431	0.00223
3.437	-0.00195	0.00115	3.437	-0.00259	0.00146	3.437	-0.00319	0.00174	3.437	-0.00376	0.00199	3.437	-0.0043	0.00223
3.438	-0.00195	0.00115	3.438	-0.00259	0.00146	3.438	-0.00319	0.00174	3.438	-0.00376	0.00199	3.438	-0.0043	0.00223
3.439	-0.00195	0.00115	3.439	-0.00258	0.00145	3.439	-0.00319	0.00173	3.439	-0.00376	0.00199	3.439	-0.00429	0.00222
3.44	-0.00195	0.00115	3.44	-0.00258	0.00145	3.44	-0.00318	0.00173	3.44	-0.00375	0.00199	3.44	-0.00429	0.00222
3.441	-0.00194	0.00114	3.441	-0.00258	0.00145	3.441	-0.00318	0.00173	3.441	-0.00375	0.00199	3.441	-0.00429	0.00222
3.442	-0.00194	0.00114	3.442	-0.00258	0.00145	3.442	-0.00318	0.00173	3.442	-0.00375	0.00198	3.442	-0.00428	0.00222
3.443	-0.00194	0.00114	3.443	-0.00258	0.00145	3.443	-0.00318	0.00173	3.443	-0.00374	0.00198	3.443	-0.00428	0.00222
3.444	-0.00194	0.00114	3.444	-0.00257	0.00145	3.444	-0.00317	0.00173	3.444	-0.00374	0.00198	3.444	-0.00428	0.00221
3.445	-0.00194	0.00114	3.445	-0.00257	0.00145	3.445	-0.00317	0.00173	3.445	-0.00374	0.00198	3.445	-0.00427	0.00221
3.446	-0.00194	0.00114	3.446	-0.00257	0.00145	3.446	-0.00317	0.00172	3.446	-0.00373	0.00198	3.446	-0.00427	0.00221
3.447	-0.00194	0.00114	3.447	-0.00257	0.00145	3.447	-0.00317	0.00172	3.447	-0.00373	0.00198	3.447	-0.00426	0.00221
3.448	-0.00193	0.00114	3.448	-0.00256	0.00144	3.448	-0.00316	0.00172	3.448	-0.00373	0.00197	3.448	-0.00426	0.00221
3.449	-0.00193	0.00114	3.449	-0.00256	0.00144	3.449	-0.00316	0.00172	3.449	-0.00373	0.00197	3.449	-0.00426	0.0022
3.45	-0.00193	0.00114	3.45	-0.00256	0.00144	3.45	-0.00316	0.00172	3.45	-0.00372	0.00197	3.45	-0.00425	0.0022
3.451	-0.00193	0.00114	3.451	-0.00256	0.00144	3.451	-0.00316	0.00172	3.451	-0.00372	0.00197	3.451	-0.00425	0.0022
3.452	-0.00193	0.00114	3.452	-0.00256	0.00144	3.452	-0.00315	0.00172	3.452	-0.00372	0.00197	3.452	-0.00425	0.0022
3.453	-0.00193	0.00113	3.453	-0.00255	0.00144	3.453	-0.00315	0.00171	3.453	-0.00371	0.00197	3.453	-0.00424	0.0022
3.454	-0.00192	0.00113	3.454	-0.00255	0.00144	3.454	-0.00315	0.00171	3.454	-0.00371	0.00196	3.454	-0.00424	0.0022
3.455	-0.00192	0.00113	3.455	-0.00255	0.00144	3.455	-0.00314	0.00171	3.455	-0.00371	0.00196	3.455	-0.00424	0.00219
3.456	-0.00192	0.00113	3.456	-0.00255	0.00143	3.456	-0.00314	0.00171	3.456	-0.0037	0.00196	3.456	-0.00423	0.00219
3.457	-0.00192	0.00113	3.457	-0.00255	0.00143	3.457	-0.00314	0.00171	3.457	-0.0037	0.00196	3.457	-0.00423	0.00219
3.458	-0.00192	0.00113	3.458	-0.00254	0.00143	3.458	-0.00314	0.00171	3.458	-0.0037	0.00196	3.458	-0.00423	0.00219
3.459	-0.00192	0.00113	3.459	-0.00254	0.00143	3.459	-0.00313	0.00171	3.459	-0.00369	0.00196	3.459	-0.00422	0.00219
3.46	-0.00192	0.00113	3.46	-0.00254	0.00143	3.46	-0.00313	0.0017	3.46	-0.00369	0.00195	3.46	-0.00422	0.00218
3.461	-0.00191	0.00113	3.461	-0.00254	0.00143	3.461	-0.00313	0.0017	3.461	-0.00369	0.00195	3.461	-0.00421	0.00218
3.462	-0.00191	0.00113	3.462	-0.00254	0.00143	3.462	-0.00313	0.0017	3.462	-0.00368	0.00195	3.462	-0.00421	0.00218
3.463	-0.00191	0.00113	3.463	-0.00253	0.00143	3.463	-0.00312	0.0017	3.463	-0.00368	0.00195	3.463	-0.00421	0.00218
3.464	-0.00191	0.00112	3.464	-0.00253	0.00143	3.464	-0.00312	0.0017	3.464	-0.00368	0.00195	3.464	-0.0042	0.00218
3.465	-0.00191	0.00112	3.465	-0.00253	0.00142	3.465	-0.00312	0.0017	3.465	-0.00368	0.00195	3.465	-0.0042	0.00217
3.466	-0.00191	0.00112	3.466	-0.00253	0.00142	3.466	-0.00312	0.0017	3.466	-0.00367	0.00194	3.466	-0.0042	0.00217
3.467	-0.00191	0.00112	3.467	-0.00253	0.00142	3.467	-0.00311	0.00169	3.467	-0.00367	0.00194	3.467	-0.00419	0.00217
3.468	-0.0019	0.00112	3.468	-0.00252	0.00142	3.468	-0.00311	0.00169	3.468	-0.00367	0.00194	3.468	-0.00419	0.00217
3.469	-0.0019	0.00112	3.469	-0.00252	0.00142	3.469	-0.00311	0.00169	3.469	-0.00366	0.00194	3.469	-0.00419	0.00217
3.47	-0.0019	0.00112	3.47	-0.00252	0.00142	3.47	-0.00311	0.00169	3.47	-0.00366	0.00194	3.47	-0.00418	0.00217
3.471	-0.0019	0.00112	3.471	-0.00252	0.00142	3.471	-0.0031	0.00169	3.471	-0.00366	0.00194	3.471	-0.00418	0.00216
3.472	-0.0019	0.00112	3.472	-0.00252	0.00142	3.472	-0.0031	0.00169	3.472	-0.00365	0.00193	3.472	-0.00418	0.00216
3.473	-0.0019	0.00112	3.473	-0.00251	0.00142	3.473	-0.0031	0.00169	3.473	-0.00365	0.00193	3.473	-0.00417	0.00216
3.474	-0.0019	0.00112	3.474	-0.00251	0.00141	3.474	-0.0031	0.00168	3.474	-0.00365	0.00193	3.474	-0.00417	0.00216
3.475	-0.00189	0.00111	3.475	-0.00251	0.00141	3.475	-0.00309	0.00168	3.475	-0.00365	0.00193	3.475	-0.00416	0.00216
3.476	-0.00189	0.00111	3.476	-0.00251	0.00141	3.476	-0.00309	0.00168	3.476	-0.00364	0.00193	3.476	-0.00416	0.00215
3.477	-0.00189	0.00111	3.477	-0.00251	0.00141	3.477	-0.00309	0.00168	3.477	-0.00364	0.00193	3.477	-0.00416	0.00215
3.478	-0.00189	0.00111	3.478	-0.0025	0.00141	3.478	-0.00309	0.00168	3.478	-0.00364	0.00192	3.478	-0.00415	0.00215
3.479	-0.00189	0.00111	3.479	-0.0025	0.00141	3.479	-0.00308	0.00168	3.479	-0.00363	0.00192	3.479	-0.00415	0.00215
3.48	-0.00189	0.00111	3.48	-0.0025	0.00141	3.48	-0.00308	0.00168	3.48	-0.00363	0.00192	3.48	-0.00415	0.00215
3.481	-0.00188	0.00111	3.481	-0.0025	0.00141	3.481	-0.00308	0.00167	3.481	-0.00363	0.00192	3.481	-0.00414	0.00215
3.482	-0.00188	0.00111	3.482	-0.0025	0.00141	3.482	-0.00308	0.00167	3.482	-0.00362	0.00192	3.482	-0.00414	0.00214
3.483	-0.00188	0.00111	3.483	-0.00249	0.0014	3.483	-0.00307	0.00167	3.483	-0.00362	0.00192	3.483	-0.00414	0.00214
3.484	-0.00188	0.00111	3.484	-0.00249	0.0014	3.484	-0.00307	0.00167	3.484	-0.00362	0.00192	3.484	-0.00413	0.00214
3.485	-0.00188	0.00111	3.485	-0.00249	0.0014	3.485	-0.00307	0.00167	3.485	-0.00361	0.00191	3.485	-0.00413	0.00214
3.486	-0.00188	0.00111	3.486	-0.00249	0.0014	3.486	-0.00307	0.00167	3.486	-0.00361	0.00191	3.486	-0.00413	0.00214
3.487	-0.00188	0.0011	3.487	-0.00249	0.0014	3.487	-0.00306	0.00167	3.487	-0.00361	0.00191	3.487	-0.00412	0.00213
3.488	-0.00187	0.0011	3.488	-0.00248	0.0014	3.488	-0.00306	0.00167	3.488	-0.00361	0.00191	3.488	-0.00412	0.00213
3.489	-0.00187	0.0011	3.489	-0.00248	0.0014	3.489	-0.00306	0.00166	3.489	-0.0036	0.00191	3.489	-0.00412	0.00213
3.49	-0.00187	0.0011	3.49	-0.00248	0.0014	3.49	-0.00306	0.00166	3.49	-0.0036	0.00191	3.49	-0.00411	0.00213
3.491	-0.00187	0.0011	3.491	-0.00248	0.0014	3.491	-0.00305	0.00166	3.491	-0.0036	0.0019	3.491	-0.00411	0.00213
3.492	-0.00187	0.0011	3.492	-0.00248	0.00139	3.492	-0.00305	0.00166	3.492	-0.00359	0.0019	3.492	-0.00411	0.00213
3.493	-0.00187	0.0011	3.493	-0.00247	0.00139	3.493	-0.00305	0.00166	3.493	-0.00359	0.0019	3.493	-0.0041	0.00212
3.494	-0.00187	0.0011	3.494	-0.00247	0.00139	3.494	-0.00305	0.00166	3.494	-0.00359	0.0019	3.494	-0.0041	0.00212
3.495	-0.00186	0.0011	3.495	-0.00247	0.00139	3.495	-0.00304	0.00166	3.495	-0.00359	0.0019	3.495	-0.0041	0.00212
3.496	-0.00186	0.0011	3.496	-0.00247	0.00139	3.496	-0.00304	0.00165	3.496	-0.00358	0.0019	3.496	-0.00409	0.00212
3.497	-0.00186	0.0011	3.497	-0.00247	0.00139	3.497	-0.00304	0.00165	3.497	-0.00358	0.00189	3.497	-0.00409	0.00212
3.498	-0.00186	0.0011	3.498	-0.00246	0.00139	3.498	-0.00304	0.00165	3.498	-0.00358	0.00189	3.498	-0.00409	0.00212
3.499	-0.00186	0.00109	3.499	-0.00246	0.00139	3.499	-0.00303	0.00165	3.499	-0.00357	0.00189	3.499	-0.00408	0.00211
3.5	-0.00186	0.00109	3.5	-0.00246	0.00139	3.5	-0.00303	0.00165	3.5	-0.00357	0.00189	3.5	-0.00408	0.00211
3.501	-0.00186	0.00109	3.501	-0.00246	0.00138	3.501	-0.00303	0.00165	3.501	-0.00357	0.00189	3.501	-0.00407	0.00211
3.502	-0.00185	0.00109	3.502	-0.00246	0.00138	3.502	-0.00303	0.00165	3.502	-0.00356	0.00189	3.502	-0.00407	0.00211
3.503	-0.00185	0.00109	3.503	-0.00245	0.00138	3.503	-0.00302	0.00165	3.503	-0.00356	0.00189	3.503	-0.00407	0.00211
3.504	-0.00185	0.00109	3.504	-0.00245	0.00138	3.504	-0.00302	0.00164	3.504	-0.00356	0.00188	3.504	-0.00406	0.0021
3.505	-0.00185	0.00109	3.505	-0.00245	0.00138	3.505	-0.00302	0.00164	3.505	-0.00356	0.00188	3.505	-0.00406	0.0021
3.506	-0.00185	0.00109	3.506	-0.00245	0.00138	3.506	-0.00302	0.00164	3.506	-0.00355	0.00188	3.506	-0.00406	0.0021
3.507	-0.00185	0.00109	3.507	-0.00245	0.00138	3.507	-0.00301	0.00164	3.507	-0.00355	0.00188	3.507	-0.00405	0.0021
3.508	-0.00185	0.00109	3.508	-0.00244	0.00138	3.508	-0.00301	0.00164	3.508	-0.00355	0.00188	3.508	-0.00405	0.0021
3.509	-0.00184	0.00109	3.509	-0.00244	0.00138	3.509	-0.00301	0.00164	3.509	-0.00354	0.00188	3.509	-0.00405	0.0021
3.51	-0.00184	0.00109	3.51	-0.00244	0.00137	3.51	-0.00301	0.00164	3.51	-0.00354	0.00187	3.51	-0.00404	0.00209
3.511	-0.00184	0.00108	3.511	-0.00244	0.00137	3.511	-0.003	0.00163	3.511	-0.00354	0.00187	3.511	-0.00404	0.00209
3.512	-0.00184	0.00108	3.512	-0.00244	0.00137	3.512	-0.003	0.00163	3.512	-0.00354	0.00187	3.512	-0.00404	0.00209
3.513	-0.00184	0.00108	3.513	-0.00244	0.00137	3.513	-0.003	0.00163	3.513	-0.00353	0.00187	3.513	-0.00403	0.00209
3.514	-0.00184	0.00108	3.514	-0.00243	0.00137	3.514	-0.003	0.00163	3.514	-0.00353	0.00187	3.514	-0.00403	0.00209
3.515	-0.00184	0.00108	3.515	-0.00243	0.00137	3.515	-0.00299	0.00163	3.515	-0.00353	0.00187	3.515	-0.00403	0.00209
3.516	-0.00183	0.00108	3.516	-0.00243	0.00137	3.516	-0.00299	0.00163	3.516	-0.00352	0.00187	3.516	-0.00402	0.00208
3.517	-0.00183	0.00108	3.517	-0.00243	0.00137	3.517	-0.00299	0.00163	3.517	-0.00352	0.00186	3.517	-0.00402	0.00208
3.518	-0.00183	0.00108	3.518	-0.00243	0.00137	3.518	-0.00299	0.00163	3.518	-0.00352	0.00186	3.518	-0.00402	0.00208
3.519	-0.00183	0.00108	3.519	-0.00242	0.00136	3.519	-0.00299	0.00162	3.519	-0.00351	0.00186	3.519	-0.00401	0.00208
3.52	-0.00183	0.00108	3.52	-0.00242	0.00136	3.52	-0.00298	0.00162	3.52	-0.00351	0.00186	3.52	-0.00401	0.00208
3.521	-0.00183	0.00108	3.521	-0.00242	0.00136	3.521	-0.00298	0.00162	3.521	-0.00351	0.00186	3.521	-0.00401	0.00208
3.522	-0.00183	0.00108	3.522	-0.00242	0.00136	3.522	-0.00298	0.00162	3.522	-0.00351	0.00186	3.522	-0.004	0.00207
3.523	-0.00182	0.00107	3.523	-0.00242	0.00136	3.523	-0.00298	0.00162	3.523	-0.0035	0.00185	3.523	-0.004	0.00207
3.524	-0.00182	0.00107	3.524	-0.00241	0.00136	3.524	-0.00297	0.00162	3.524	-0.0035	0.00185	3.524	-0.004	0.00207
3.525	-0.00182	0.00107	3.525	-0.00241	0.00136	3.525	-0.00297	0.00162	3.525	-0.0035	0.00185	3.525	-0.00399	0.00207
3.526	-0.00182	0.00107	3.526	-0.00241	0.00136	3.526	-0.00297	0.00162	3.526	-0.00349	0.00185	3.526	-0.00399	0.00207
3.527	-0.00182	0.00107	3.527	-0.00241	0.00136	3.527	-0.00297	0.00161	3.527	-0.00349	0.00185	3.527	-0.00399	0.00206
3.528	-0.00182	0.00107	3.528	-0.00241	0.00135	3.528	-0.00296	0.00161	3.528	-0.00349	0.00185	3.528	-0.00398	0.00206
3.529	-0.00182	0.00107	3.529	-0.0024	0.00135	3.529	-0.00296	0.00161	3.529	-0.00349	0.00185	3.529	-0.00398	0.00206
3.53	-0.00182	0.00107	3.53	-0.0024	0.00135	3.53	-0.00296	0.00161	3.53	-0.00348	0.00184	3.53	-0.00398	0.00206
3.531	-0.00181	0.00107	3.531	-0.0024	0.00135	3.531	-0.00296	0.00161	3.531	-0.00348	0.00184	3.531	-0.00397	0.00206
3.532	-0.00181	0.00107	3.532	-0.0024	0.00135	3.532	-0.00295	0.00161	3.532	-0.00348	0.00184	3.532	-0.00397	0.00206
3.533	-0.00181	0.00107	3.533	-0.0024	0.00135	3.533	-0.00295	0.00161	3.533	-0.00347	0.00184	3.533	-0.00397	0.00205
3.534	-0.00181	0.00107	3.534	-0.0024	0.00135	3.534	-0.00295	0.0016	3.534	-0.00347	0.00184	3.534	-0.00396	0.00205
3.535	-0.00181	0.00106	3.535	-0.00239	0.00135	3.535	-0.00295	0.0016	3.535	-0.00347	0.00184	3.535	-0.00396	0.00205
3.536	-0.00181	0.00106	3.536	-0.00239	0.00135	3.536	-0.00294	0.0016	3.536	-0.00347	0.00184	3.536	-0.00396	0.00205
3.537	-0.00181	0.00106	3.537	-0.00239	0.00135	3.537	-0.00294	0.0016	3.537	-0.00346	0.00183	3.537	-0.00395	0.00205
3.538	-0.0018	0.00106	3.538	-0.00239	0.00134	3.538	-0.00294	0.0016	3.538	-0.00346	0.00183	3.538	-0.00395	0.00205
3.539	-0.0018	0.00106	3.539	-0.00239	0.00134	3.539	-0.00294	0.0016	3.539	-0.00346	0.00183	3.539	-0.00395	0.00204
3.54	-0.0018	0.00106	3.54	-0.00238	0.00134	3.54	-0.00294	0.0016	3.54	-0.00346	0.00183	3.54	-0.00394	0.00204
3.541	-0.0018	0.00106	3.541	-0.00238	0.00134	3.541	-0.00293	0.0016	3.541	-0.00345	0.00183	3.541	-0.00394	0.00204
3.542	-0.0018	0.00106	3.542	-0.00238	0.00134	3.542	-0.00293	0.00159	3.542	-0.00345	0.00183	3.542	-0.00394	0.00204
3.543	-0.0018	0.00106	3.543	-0.00238	0.00134	3.543	-0.00293	0.00159	3.543	-0.00345	0.00182	3.543	-0.00393	0.00204
3.544	-0.0018	0.00106	3.544	-0.00238	0.00134	3.544	-0.00293	0.00159	3.544	-0.00344	0.00182	3.544	-0.00393	0.00204
3.545	-0.00179	0.00106	3.545	-0.00237	0.00134	3.545	-0.00292	0.00159	3.545	-0.00344	0.00182	3.545	-0.00393	0.00203
3.546	-0.00179	0.00106	3.546	-0.00237	0.00134	3.546	-0.00292	0.00159	3.546	-0.00344	0.00182	3.546	-0.00393	0.00203
3.547	-0.00179	0.00105	3.547	-0.00237	0.00133	3.547	-0.00292	0.00159	3.547	-0.00344	0.00182	3.547	-0.00392	0.00203
3.548	-0.00179	0.00105	3.548	-0.00237	0.00133	3.548	-0.00292	0.00159	3.548	-0.00343	0.00182	3.548	-0.00392	0.00203
3.549	-0.00179	0.00105	3.549	-0.00237	0.00133	3.549	-0.00291	0.00159	3.549	-0.00343	0.00182	3.549	-0.00392	0.00203
3.55	-0.00179	0.00105	3.55	-0.00237	0.00133	3.55	-0.00291	0.00158	3.55	-0.00343	0.00181	3.55	-0.00391	0.00203
3.551	-0.00179	0.00105	3.551	-0.00236	0.00133	3.551	-0.00291	0.00158	3.551	-0.00342	0.00181	3.551	-0.00391	0.00202
3.552	-0.00178	0.00105	3.552	-0.00236	0.00133	3.552	-0.00291	0.00158	3.552	-0.00342	0.00181	3.552	-0.00391	0.00202

3.553	-0.00178	0.00105	3.553	-0.00236	0.00133	3.553	-0.0029	0.00158	3.553	-0.00342	0.00181	3.553	-0.0039	0.00202
3.554	-0.00178	0.00105	3.554	-0.00236	0.00133	3.554	-0.0029	0.00158	3.554	-0.00342	0.00181	3.554	-0.0039	0.00202
3.555	-0.00178	0.00105	3.555	-0.00236	0.00133	3.555	-0.0029	0.00158	3.555	-0.00341	0.00181	3.555	-0.0039	0.00202
3.556	-0.00178	0.00105	3.556	-0.00235	0.00133	3.556	-0.0029	0.00158	3.556	-0.00341	0.00181	3.556	-0.00389	0.00202
3.557	-0.00178	0.00105	3.557	-0.00235	0.00132	3.557	-0.0029	0.00158	3.557	-0.00341	0.0018	3.557	-0.00389	0.00201
3.558	-0.00178	0.00105	3.558	-0.00235	0.00132	3.558	-0.00289	0.00157	3.558	-0.0034	0.0018	3.558	-0.00389	0.00201
3.559	-0.00178	0.00105	3.559	-0.00235	0.00132	3.559	-0.00289	0.00157	3.559	-0.0034	0.0018	3.559	-0.00388	0.00201
3.56	-0.00177	0.00104	3.56	-0.00235	0.00132	3.56	-0.00289	0.00157	3.56	-0.0034	0.0018	3.56	-0.00388	0.00201
3.561	-0.00177	0.00104	3.561	-0.00235	0.00132	3.561	-0.00289	0.00157	3.561	-0.0034	0.0018	3.561	-0.00388	0.00201
3.562	-0.00177	0.00104	3.562	-0.00234	0.00132	3.562	-0.00288	0.00157	3.562	-0.00339	0.0018	3.562	-0.00387	0.00201
3.563	-0.00177	0.00104	3.563	-0.00234	0.00132	3.563	-0.00288	0.00157	3.563	-0.00339	0.0018	3.563	-0.00387	0.002
3.564	-0.00177	0.00104	3.564	-0.00234	0.00132	3.564	-0.00288	0.00157	3.564	-0.00339	0.00179	3.564	-0.00387	0.002
3.565	-0.00177	0.00104	3.565	-0.00234	0.00132	3.565	-0.00288	0.00157	3.565	-0.00339	0.00179	3.565	-0.00386	0.002
3.566	-0.00177	0.00104	3.566	-0.00234	0.00132	3.566	-0.00287	0.00156	3.566	-0.00338	0.00179	3.566	-0.00386	0.002
3.567	-0.00176	0.00104	3.567	-0.00233	0.00131	3.567	-0.00287	0.00156	3.567	-0.00338	0.00179	3.567	-0.00386	0.002
3.568	-0.00176	0.00104	3.568	-0.00233	0.00131	3.568	-0.00287	0.00156	3.568	-0.00338	0.00179	3.568	-0.00385	0.002
3.569	-0.00176	0.00104	3.569	-0.00233	0.00131	3.569	-0.00287	0.00156	3.569	-0.00337	0.00179	3.569	-0.00385	0.00199
3.57	-0.00176	0.00104	3.57	-0.00233	0.00131	3.57	-0.00287	0.00156	3.57	-0.00337	0.00179	3.57	-0.00385	0.00199
3.571	-0.00176	0.00104	3.571	-0.00233	0.00131	3.571	-0.00286	0.00156	3.571	-0.00337	0.00178	3.571	-0.00385	0.00199
3.572	-0.00176	0.00104	3.572	-0.00233	0.00131	3.572	-0.00286	0.00156	3.572	-0.00337	0.00178	3.572	-0.00384	0.00199
3.573	-0.00176	0.00103	3.573	-0.00232	0.00131	3.573	-0.00286	0.00156	3.573	-0.00336	0.00178	3.573	-0.00384	0.00199
3.574	-0.00176	0.00103	3.574	-0.00232	0.00131	3.574	-0.00286	0.00155	3.574	-0.00336	0.00178	3.574	-0.00384	0.00199
3.575	-0.00175	0.00103	3.575	-0.00232	0.00131	3.575	-0.00285	0.00155	3.575	-0.00336	0.00178	3.575	-0.00383	0.00198
3.576	-0.00175	0.00103	3.576	-0.00232	0.00131	3.576	-0.00285	0.00155	3.576	-0.00336	0.00178	3.576	-0.00383	0.00198
3.577	-0.00175	0.00103	3.577	-0.00232	0.0013	3.577	-0.00285	0.00155	3.577	-0.00335	0.00178	3.577	-0.00383	0.00198
3.578	-0.00175	0.00103	3.578	-0.00231	0.0013	3.578	-0.00285	0.00155	3.578	-0.00335	0.00177	3.578	-0.00382	0.00198
3.579	-0.00175	0.00103	3.579	-0.00231	0.0013	3.579	-0.00285	0.00155	3.579	-0.00335	0.00177	3.579	-0.00382	0.00198
3.58	-0.00175	0.00103	3.58	-0.00231	0.0013	3.58	-0.00284	0.00155	3.58	-0.00334	0.00177	3.58	-0.00382	0.00198
3.581	-0.00175	0.00103	3.581	-0.00231	0.0013	3.581	-0.00284	0.00155	3.581	-0.00334	0.00177	3.581	-0.00381	0.00197
3.582	-0.00174	0.00103	3.582	-0.00231	0.0013	3.582	-0.00284	0.00154	3.582	-0.00334	0.00177	3.582	-0.00381	0.00197
3.583	-0.00174	0.00103	3.583	-0.00231	0.0013	3.583	-0.00284	0.00154	3.583	-0.00334	0.00177	3.583	-0.00381	0.00197
3.584	-0.00174	0.00103	3.584	-0.0023	0.0013	3.584	-0.00283	0.00154	3.584	-0.00333	0.00177	3.584	-0.0038	0.00197
3.585	-0.00174	0.00103	3.585	-0.0023	0.0013	3.585	-0.00283	0.00154	3.585	-0.00333	0.00176	3.585	-0.0038	0.00197
3.586	-0.00174	0.00102	3.586	-0.0023	0.0013	3.586	-0.00283	0.00154	3.586	-0.00333	0.00176	3.586	-0.0038	0.00197
3.587	-0.00174	0.00102	3.587	-0.0023	0.00129	3.587	-0.00283	0.00154	3.587	-0.00333	0.00176	3.587	-0.0038	0.00197
3.588	-0.00174	0.00102	3.588	-0.0023	0.00129	3.588	-0.00283	0.00154	3.588	-0.00332	0.00176	3.588	-0.00379	0.00196
3.589	-0.00174	0.00102	3.589	-0.00229	0.00129	3.589	-0.00282	0.00154	3.589	-0.00332	0.00176	3.589	-0.00379	0.00196
3.59	-0.00173	0.00102	3.59	-0.00229	0.00129	3.59	-0.00282	0.00153	3.59	-0.00332	0.00176	3.59	-0.00379	0.00196
3.591	-0.00173	0.00102	3.591	-0.00229	0.00129	3.591	-0.00282	0.00153	3.591	-0.00332	0.00176	3.591	-0.00378	0.00196
3.592	-0.00173	0.00102	3.592	-0.00229	0.00129	3.592	-0.00282	0.00153	3.592	-0.00331	0.00175	3.592	-0.00378	0.00196
3.593	-0.00173	0.00102	3.593	-0.00229	0.00129	3.593	-0.00281	0.00153	3.593	-0.00331	0.00175	3.593	-0.00378	0.00196
3.594	-0.00173	0.00102	3.594	-0.00229	0.00129	3.594	-0.00281	0.00153	3.594	-0.00331	0.00175	3.594	-0.00377	0.00195
3.595	-0.00173	0.00102	3.595	-0.00228	0.00129	3.595	-0.00281	0.00153	3.595	-0.0033	0.00175	3.595	-0.00377	0.00195
3.596	-0.00173	0.00102	3.596	-0.00228	0.00129	3.596	-0.00281	0.00153	3.596	-0.0033	0.00175	3.596	-0.00377	0.00195
3.597	-0.00173	0.00102	3.597	-0.00228	0.00128	3.597	-0.00281	0.00153	3.597	-0.0033	0.00175	3.597	-0.00376	0.00195
3.598	-0.00172	0.00102	3.598	-0.00228	0.00128	3.598	-0.0028	0.00152	3.598	-0.0033	0.00175	3.598	-0.00376	0.00195
3.599	-0.00172	0.00101	3.599	-0.00228	0.00128	3.599	-0.0028	0.00152	3.599	-0.00329	0.00174	3.599	-0.00376	0.00195
3.6	-0.00172	0.00101	3.6	-0.00228	0.00128	3.6	-0.0028	0.00152	3.6	-0.00329	0.00174	3.6	-0.00376	0.00194
3.601	-0.00172	0.00101	3.601	-0.00227	0.00128	3.601	-0.0028	0.00152	3.601	-0.00329	0.00174	3.601	-0.00375	0.00194
3.602	-0.00172	0.00101	3.602	-0.00227	0.00128	3.602	-0.00279	0.00152	3.602	-0.00329	0.00174	3.602	-0.00375	0.00194
3.603	-0.00172	0.00101	3.603	-0.00227	0.00128	3.603	-0.00279	0.00152	3.603	-0.00328	0.00174	3.603	-0.00375	0.00194
3.604	-0.00172	0.00101	3.604	-0.00227	0.00128	3.604	-0.00279	0.00152	3.604	-0.00328	0.00174	3.604	-0.00374	0.00194
3.605	-0.00172	0.00101	3.605	-0.00227	0.00128	3.605	-0.00279	0.00152	3.605	-0.00328	0.00174	3.605	-0.00374	0.00194
3.606	-0.00171	0.00101	3.606	-0.00226	0.00128	3.606	-0.00279	0.00152	3.606	-0.00328	0.00173	3.606	-0.00374	0.00193
3.607	-0.00171	0.00101	3.607	-0.00226	0.00127	3.607	-0.00278	0.00151	3.607	-0.00327	0.00173	3.607	-0.00373	0.00193
3.608	-0.00171	0.00101	3.608	-0.00226	0.00127	3.608	-0.00278	0.00151	3.608	-0.00327	0.00173	3.608	-0.00373	0.00193
3.609	-0.00171	0.00101	3.609	-0.00226	0.00127	3.609	-0.00278	0.00151	3.609	-0.00327	0.00173	3.609	-0.00373	0.00193
3.61	-0.00171	0.00101	3.61	-0.00226	0.00127	3.61	-0.00278	0.00151	3.61	-0.00327	0.00173	3.61	-0.00372	0.00193
3.611	-0.00171	0.00101	3.611	-0.00226	0.00127	3.611	-0.00277	0.00151	3.611	-0.00326	0.00173	3.611	-0.00372	0.00193
3.612	-0.00171	0.001	3.612	-0.00225	0.00127	3.612	-0.00277	0.00151	3.612	-0.00326	0.00173	3.612	-0.00372	0.00193
3.613	-0.0017	0.001	3.613	-0.00225	0.00127	3.613	-0.00277	0.00151	3.613	-0.00326	0.00172	3.613	-0.00372	0.00192
3.614	-0.0017	0.001	3.614	-0.00225	0.00127	3.614	-0.00277	0.00151	3.614	-0.00325	0.00172	3.614	-0.00371	0.00192
3.615	-0.0017	0.001	3.615	-0.00225	0.00127	3.615	-0.00277	0.0015	3.615	-0.00325	0.00172	3.615	-0.00371	0.00192
3.616	-0.0017	0.001	3.616	-0.00225	0.00127	3.616	-0.00276	0.0015	3.616	-0.00325	0.00172	3.616	-0.00371	0.00192
3.617	-0.0017	0.001	3.617	-0.00225	0.00126	3.617	-0.00276	0.0015	3.617	-0.00325	0.00172	3.617	-0.0037	0.00192
3.618	-0.0017	0.001	3.618	-0.00224	0.00126	3.618	-0.00276	0.0015	3.618	-0.00324	0.00172	3.618	-0.0037	0.00192
3.619	-0.0017	9.99E-04	3.619	-0.00224	0.00126	3.619	-0.00276	0.0015	3.619	-0.00324	0.00172	3.619	-0.0037	0.00191
3.62	-0.0017	9.99E-04	3.62	-0.00224	0.00126	3.62	-0.00275	0.0015	3.62	-0.00324	0.00171	3.62	-0.00369	0.00191
3.621	-0.00169	9.98E-04	3.621	-0.00224	0.00126	3.621	-0.00275	0.0015	3.621	-0.00324	0.00171	3.621	-0.00369	0.00191
3.622	-0.00169	9.97E-04	3.622	-0.00224	0.00126	3.622	-0.00275	0.0015	3.622	-0.00323	0.00171	3.622	-0.00369	0.00191
3.623	-0.00169	9.96E-04	3.623	-0.00224	0.00126	3.623	-0.00275	0.0015	3.623	-0.00323	0.00171	3.623	-0.00369	0.00191
3.624	-0.00169	9.96E-04	3.624	-0.00223	0.00126	3.624	-0.00275	0.00149	3.624	-0.00323	0.00171	3.624	-0.00368	0.00191
3.625	-0.00169	9.95E-04	3.625	-0.00223	0.00126	3.625	-0.00274	0.00149	3.625	-0.00323	0.00171	3.625	-0.00368	0.00191
3.626	-0.00169	9.94E-04	3.626	-0.00223	0.00126	3.626	-0.00274	0.00149	3.626	-0.00322	0.00171	3.626	-0.00368	0.0019
3.627	-0.00169	9.93E-04	3.627	-0.00223	0.00125	3.627	-0.00274	0.00149	3.627	-0.00322	0.00171	3.627	-0.00367	0.0019
3.628	-0.00169	9.93E-04	3.628	-0.00223	0.00125	3.628	-0.00274	0.00149	3.628	-0.00322	0.0017	3.628	-0.00367	0.0019
3.629	-0.00168	9.92E-04	3.629	-0.00223	0.00125	3.629	-0.00274	0.00149	3.629	-0.00322	0.0017	3.629	-0.00367	0.0019
3.63	-0.00168	9.91E-04	3.63	-0.00222	0.00125	3.63	-0.00273	0.00149	3.63	-0.00321	0.0017	3.63	-0.00366	0.0019
3.631	-0.00168	9.90E-04	3.631	-0.00222	0.00125	3.631	-0.00273	0.00149	3.631	-0.00321	0.0017	3.631	-0.00366	0.0019
3.632	-0.00168	9.90E-04	3.632	-0.00222	0.00125	3.632	-0.00273	0.00148	3.632	-0.00321	0.0017	3.632	-0.00366	0.00189
3.633	-0.00168	9.89E-04	3.633	-0.00222	0.00125	3.633	-0.00273	0.00148	3.633	-0.00321	0.0017	3.633	-0.00366	0.00189
3.634	-0.00168	9.88E-04	3.634	-0.00222	0.00125	3.634	-0.00272	0.00148	3.634	-0.0032	0.0017	3.634	-0.00365	0.00189
3.635	-0.00168	9.87E-04	3.635	-0.00221	0.00125	3.635	-0.00272	0.00148	3.635	-0.0032	0.00169	3.635	-0.00365	0.00189
3.636	-0.00168	9.87E-04	3.636	-0.00221	0.00125	3.636	-0.00272	0.00148	3.636	-0.0032	0.00169	3.636	-0.00365	0.00189
3.637	-0.00167	9.86E-04	3.637	-0.00221	0.00125	3.637	-0.00272	0.00148	3.637	-0.0032	0.00169	3.637	-0.00364	0.00189
3.638	-0.00167	9.85E-04	3.638	-0.00221	0.00124	3.638	-0.00272	0.00148	3.638	-0.00319	0.00169	3.638	-0.00364	0.00189
3.639	-0.00167	9.85E-04	3.639	-0.00221	0.00124	3.639	-0.00271	0.00148	3.639	-0.00319	0.00169	3.639	-0.00364	0.00188
3.64	-0.00167	9.84E-04	3.64	-0.00221	0.00124	3.64	-0.00271	0.00148	3.64	-0.00319	0.00169	3.64	-0.00364	0.00188
3.641	-0.00167	9.83E-04	3.641	-0.0022	0.00124	3.641	-0.00271	0.00147	3.641	-0.00319	0.00169	3.641	-0.00363	0.00188
3.642	-0.00167	9.82E-04	3.642	-0.0022	0.00124	3.642	-0.00271	0.00147	3.642	-0.00318	0.00169	3.642	-0.00363	0.00188
3.643	-0.00167	9.82E-04	3.643	-0.0022	0.00124	3.643	-0.00271	0.00147	3.643	-0.00318	0.00168	3.643	-0.00363	0.00188
3.644	-0.00167	9.81E-04	3.644	-0.0022	0.00124	3.644	-0.0027	0.00147	3.644	-0.00318	0.00168	3.644	-0.00362	0.00188
3.645	-0.00166	9.80E-04	3.645	-0.0022	0.00124	3.645	-0.0027	0.00147	3.645	-0.00318	0.00168	3.645	-0.00362	0.00187
3.646	-0.00166	9.79E-04	3.646	-0.0022	0.00124	3.646	-0.0027	0.00147	3.646	-0.00317	0.00168	3.646	-0.00362	0.00187
3.647	-0.00166	9.79E-04	3.647	-0.00219	0.00124	3.647	-0.0027	0.00147	3.647	-0.00317	0.00168	3.647	-0.00362	0.00187
3.648	-0.00166	9.78E-04	3.648	-0.00219	0.00123	3.648	-0.0027	0.00147	3.648	-0.00317	0.00168	3.648	-0.00361	0.00187
3.649	-0.00166	9.77E-04	3.649	-0.00219	0.00123	3.649	-0.00269	0.00147	3.649	-0.00317	0.00168	3.649	-0.00361	0.00187
3.65	-0.00166	9.77E-04	3.65	-0.00219	0.00123	3.65	-0.00269	0.00146	3.65	-0.00316	0.00167	3.65	-0.00361	0.00187
3.651	-0.00166	9.76E-04	3.651	-0.00219	0.00123	3.651	-0.00269	0.00146	3.651	-0.00316	0.00167	3.651	-0.0036	0.00187
3.652	-0.00166	9.75E-04	3.652	-0.00219	0.00123	3.652	-0.00269	0.00146	3.652	-0.00316	0.00167	3.652	-0.0036	0.00186
3.653	-0.00165	9.74E-04	3.653	-0.00218	0.00123	3.653	-0.00268	0.00146	3.653	-0.00316	0.00167	3.653	-0.0036	0.00186
3.654	-0.00165	9.74E-04	3.654	-0.00218	0.00123	3.654	-0.00268	0.00146	3.654	-0.00315	0.00167	3.654	-0.00359	0.00186
3.655	-0.00165	9.73E-04	3.655	-0.00218	0.00123	3.655	-0.00268	0.00146	3.655	-0.00315	0.00167	3.655	-0.00359	0.00186
3.656	-0.00165	9.72E-04	3.656	-0.00218	0.00123	3.656	-0.00268	0.00146	3.656	-0.00315	0.00167	3.656	-0.00359	0.00186
3.657	-0.00165	9.72E-04	3.657	-0.00218	0.00123	3.657	-0.00268	0.00146	3.657	-0.00315	0.00167	3.657	-0.00359	0.00186
3.658	-0.00165	9.71E-04	3.658	-0.00218	0.00123	3.658	-0.00267	0.00146	3.658	-0.00314	0.00166	3.658	-0.00358	0.00186
3.659	-0.00165	9.70E-04	3.659	-0.00217	0.00122	3.659	-0.00267	0.00145	3.659	-0.00314	0.00166	3.659	-0.00358	0.00185
3.66	-0.00165	9.69E-04	3.66	-0.00217	0.00122	3.66	-0.00267	0.00145	3.66	-0.00314	0.00166	3.66	-0.00358	0.00185
3.661	-0.00165	9.69E-04	3.661	-0.00217	0.00122	3.661	-0.00267	0.00145	3.661	-0.00314	0.00166	3.661	-0.00357	0.00185
3.662	-0.00164	9.68E-04	3.662	-0.00217	0.00122	3.662	-0.00267	0.00145	3.662	-0.00313	0.00166	3.662	-0.00357	0.00185
3.663	-0.00164	9.67E-04	3.663	-0.00217	0.00122	3.663	-0.00266	0.00145	3.663	-0.00313	0.00166	3.663	-0.00357	0.00185
3.664	-0.00164	9.67E-04	3.664	-0.00217	0.00122	3.664	-0.00266	0.00145	3.664	-0.00313	0.00166	3.664	-0.00357	0.00185
3.665	-0.00164	9.66E-04	3.665	-0.00216	0.00122	3.665	-0.00266	0.00145	3.665	-0.00313	0.00165	3.665	-0.00356	0.00185
3.666	-0.00164	9.65E-04	3.666	-0.00216	0.00122	3.666	-0.00266	0.00145	3.666	-0.00312	0.00165	3.666	-0.00356	0.00184
3.667	-0.00164	9.64E-04	3.667	-0.00216	0.00122	3.667	-0.00266	0.00144	3.667	-0.00312	0.00165	3.667	-0.00356	0.00184
3.668	-0.00164	9.64E-04	3.668	-0.00216	0.00122	3.668	-0.00265	0.00144	3.668	-0.00312	0.00165	3.668	-0.00355	0.00184
3.669	-0.00164	9.63E-04	3.669	-0.00216	0.00122	3.669	-0.00265	0.00144	3.669	-0.00312	0.00165	3.669	-0.00355	0.00184
3.67	-0.00163	9.62E-04	3.67	-0.00216	0.00121	3.67	-0.00265	0.00144	3.67	-0.00311	0.00165	3.67	-0.00355	0.00184
3.671	-0.00163	9.62E-04	3.671	-0.00216	0.00121	3.671	-0.00265	0.00144	3.671	-0.00311	0.00165	3.671	-0.00355	0.00184
3.672	-0.00163	9.61E-04	3.672	-0.00215	0.00121	3.672	-0.00265	0.00144	3.672	-0.00311	0.00165	3.672	-0.00354	0.00183
3.673	-0.00163	9.60E-04	3.673	-0.00215	0.00121	3.673	-0.00264	0.00144	3.673	-0.00311	0.00164	3.673	-0.00354	0.00183
3.674	-0.00163	9.59E-04	3.674	-0.00215	0.00121	3.674	-0.00264	0.00144	3.674	-0.0031	0.00164	3.674	-0.00354	0.00183
3.675	-0.00163	9.59E-04	3.675	-0.00215	0.00121	3.675	-0.00264	0.00144	3.675	-0.0031	0.00164	3.675	-0.00353	0.00183
3.676	-0.00163	9.58E-04	3.676	-0.00215	0.00121	3.676	-0.00264	0.00143	3.676	-0.0031	0.00164	3.676	-0.00353	0.00183
3.677	-0.00163	9.57E-04	3.677	-0.00215	0.00121	3.677	-0.00264	0.00143	3.677	-0.0031	0.00164	3.677	-0.00353	0.00183
3.678	-0.00162	9.57E-04	3.678	-0.00214	0.00121	3.678	-0.00263	0.00143	3.678	-0.00309	0.00164	3.678	-0.00353	0.00183
3.679	-0.00162	9.56E-04	3.679	-0.00214	0.00121	3.679	-0.00263	0.00143	3.679	-0.00309	0.00164	3.679	-0.00352	0.00182
3.68	-0.00162	9.55E-04	3.68	-0.00214	0.00121	3.68	-0.00263	0.00143	3.68	-0.00309	0.00164	3.68	-0.00352	0.00182
3.681	-0.00162	9.55E-04	3.681	-0.00214	0.0012	3.681	-0.00263	0.00143	3.681	-0.00309	0.00163	3.681	-0.00352	0.00182
3.682	-0.00162	9.54E-04	3.682	-0.00214	0.0012	3.682	-0.00263	0.00143	3.682	-0.00308	0.00163	3.682	-0.00352	0.00182
3.683	-0.00162	9.53E-04	3.683	-0.00214	0.0012	3.683	-0.00262	0.00143	3.683	-0.00308	0.00163	3.683	-0.00351	0.00182
3.684	-0.00162	9.52E-04	3.684	-0.00213	0.0012	3.684	-0.00262	0.00143	3.684	-0.00308	0.00163	3.684	-0.00351	0.00182
3.685	-0.00162	9.52E-04	3.685	-0.00213	0.0012	3.685	-0.00262	0.00142	3.685	-0.00308	0.00163	3.685	-0.00351	0.00182
3.686	-0.00162	9.51E-04	3.686	-0.00213	0.0012	3.686	-0.00262	0.00142	3.686	-0.00307	0.00163	3.686	-0.0035	0.00181
3.687	-0.00161	9.50E-04	3.687	-0.00213	0.0012	3.687	-0.00262	0.00142	3.687	-0.00307	0.00163	3.687	-0.0035	0.00181
3.688	-0.00161	9.50E-04	3.688	-0.00213	0.0012	3.688	-0.00261	0.00142	3.688	-0.00307	0.00163	3.688	-0.0035	0.00181
3.689	-0.00161	9.49E-04	3.689	-0.00213	0.0012	3.689	-0.00261	0.00142	3.689	-0.00307	0.00162	3.689	-0.0035	0.00181
3.69	-0.00161	9.48E-04	3.69	-0.00212	0.0012	3.69	-0.00261	0.00142	3.69	-0.00306	0.00162	3.69	-0.00349	0.00181
3.691	-0.00161	9.48E-04	3.691	-0.00212	0.0012	3.691	-0.00261	0.00142	3.691	-0.00306	0.00162	3.691	-0.00349	0.00181
3.692	-0.00161	9.47E-04	3.692	-0.00212	0.00119	3.692	-0.00261	0.00142	3.692	-0.00306	0.00162	3.692	-0.00349	0.00181
3.693	-0.00161	9.46E-04	3.693	-0.00212	0.00119	3.693	-0.0026	0.00142	3.693	-0.00306	0.00162	3.693	-0.00348	0.0018
3.694	-0.00161	9.45E-04	3.694	-0.00212	0.00119	3.694	-0.0026	0.00142	3.694	-0.00306	0.00162	3.694	-0.00348	0.0018
3.695	-0.0016	9.45E-04	3.695	-0.00212	0.00119	3.695	-0.0026	0.00141	3.695	-0.00305	0.00162	3.695	-0.00348	0.0018
3.696	-0.0016	9.44E-04	3.696	-0.00211	0.00119	3.696	-0.0026	0.00141	3.696	-0.00305	0.00161	3.696	-0.00348	0.0018
3.697	-0.0016	9.43E-04	3.697	-0.00211	0.00119	3.697	-0.0026	0.00141	3.697	-0.00305	0.00161	3.697	-0.00347	0.0018
3.698	-0.0016	9.43E-04	3.698	-0.00211	0.00119	3.698	-0.00259	0.00141	3.698	-0.00305	0.00161	3.698	-0.00347	0.0018
3.699	-0.0016	9.42E-04	3.699	-0.00211	0.00119	3.699	-0.00259	0.00141	3.699	-0.00304	0.00161	3.699	-0.00347	0.0018
3.7	-0.0016	9.41E-04	3.7	-0.00211	0.00119	3.7	-0.00259	0.00141	3.7	-0.00304	0.00161	3.7	-0.00347	0.00179
3.701	-0.0016	9.41E-04	3.701	-0.00211	0.00119	3.701	-0.00259	0.00141	3.701	-0.00304	0.00161	3.701	-0.00346	0.00179
3.702	-0.0016	9.40E-04	3.702	-0.00211	0.00119	3.702	-0.00259	0.00141	3.702	-0.00304	0.00161	3.702	-0.00346	0.00179
3.703	-0.0016	9.39E-04	3.703	-0.0021	0.00118	3.703	-0.00258	0.00141	3.703	-0.00303	0.00161	3.703	-0.00346	0.00179
3.704	-0.00159	9.39E-04	3.704	-0.0021	0.00118	3.704	-0.00258	0.0014	3.704	-0.00303	0.0016	3.704	-0.00345	0.00179
3.705	-0.00159	9.38E-04	3.705	-0.0021	0.00118	3.705	-0.00258	0.0014	3.705	-0.00303	0.0016	3.705	-0.00345	0.00179
3.706	-0.00159	9.37E-04	3.706	-0.0021	0.00118	3.706	-0.00258	0.0014	3.706	-0.00303	0.0016	3.706	-0.00345	0.00179
3.707	-0.00159	9.37E-04	3.707	-0.0021	0.00118	3.707	-0.00258	0.0014	3.707	-0.00302	0.0016	3.707	-0.00345	0.00178
3.708	-0.00159	9.36E-04	3.708	-0.0021	0.00118	3.708	-0.00257	0.0014	3.708	-0.00302	0.0016	3.708	-0.00344	0.00178
3.709	-0.00159	9.35E-04	3.709	-0.00209	0.00118	3.709	-0.00257	0.0014	3.709	-0.00302	0.0016	3.709	-0.00344	0.00178
3.71	-0.00159	9.35E-04	3.71	-0.00209	0.00118	3.71	-0.00257	0.0014	3.71	-0.00302	0.0016	3.71	-0.00344	0.00178
3.711	-0.00159	9.34E-04	3.711	-0.00209	0.00118	3.711	-0.00257	0.0014	3.711	-0.00301	0.0016	3.711	-0.00344	0.00178
3.712	-0.00158	9.33E-04	3.712	-0.00209	0.00118	3.712	-0.00257	0.0014	3.712	-0.00301	0.00159	3.712	-0.00343	0.00178
3.713	-0.00158	9.32E-04	3.713	-0.00209	0.00118	3.713	-0.00256	0.00139	3.713	-0.00301	0.00159	3.713	-0.00343	0.00178
3.714	-0.00158	9.32E-04	3.714	-0.00209	0.00117	3.714	-0.00256	0.00139	3.714	-0.00301	0.00159	3.714	-0.00343	0.00177
3.715	-0.00158	9.31E-04	3.715	-0.00209	0.00117	3.715	-0.00256	0.00139	3.715	-0.00301	0.00159	3.715	-0.00342	0.00177
3.716	-0.00158	9.30E-04	3.716	-0.00208	0.00117	3.716	-0.00256	0.00139	3.716	-0.003	0.00159	3.716	-0.00342	0.00177
3.717	-0.00158	9.30E-04	3.717	-0.00208	0.00117	3.717	-0.00256	0.00139	3.717	-0.003	0.00159	3.717	-0.00342	0.00177
3.718	-0.00158	9.29E-04	3.718	-0.00208	0.00117	3.718	-0.00255	0.00139	3.718	-0.003	0.00159	3.718	-0.00342	0.00177
3.719	-0.00158	9.28E-04	3.719	-0.00208	0.00117	3.719	-0.00255	0.00139	3.719	-0.003	0.00159	3.719	-0.00341	0.00177
3.72	-0.00158	9.28E-04	3.72	-0.00208	0.00117	3.72	-0.00255	0.00139	3.72	-0.00299	0.00158	3.72	-0.00341	0.00177
3.721	-0.00157	9.27E-04	3.721	-0.00208	0.00117	3.721	-0.00255	0.00139	3.721	-0.00299	0.00158	3.721	-0.00341	0.00176
3.722	-0.00157	9.26E-04	3.722	-0.00207	0.00117	3.722	-0.00255	0.00139	3.722	-0.00299	0.00158	3.722	-0.00341	0.00176
3.723	-0.00157	9.26E-04	3.723	-0.00207	0.00117	3.723	-0.00254	0.00138	3.723	-0.00299	0.00158	3.723	-0.0034	0.00176
3.724	-0.00157	9.25E-04	3.724	-0.00207	0.00117	3.724	-0.00254	0.00138	3.724	-0.00298	0.00158	3.724	-0.0034	0.00176
3.725	-0.00157	9.24E-04	3.725	-0.00207	0.00117	3.725	-0.00254	0.00138	3.725	-0.00298	0.00158	3.725	-0.0034	0.00176
3.726	-0.00157	9.24E-04	3.726	-0.00207	0.00116	3.726	-0.00254	0.00138	3.726	-0.00298	0.00158	3.726	-0.00339	0.00176
3.727	-0.00157	9.23E-04	3.727	-0.00207	0.00116	3.727	-0.00254	0.00138	3.727	-0.00298	0.00158	3.727	-0.00339	0.00176
3.728	-0.00157	9.22E-04	3.728	-0.00206	0.00116	3.728	-0.00253	0.00138	3.728	-0.00298	0.00158	3.728	-0.00339	0.00176
3.729	-0.00157	9.22E-04	3.729	-0.00206	0.00116	3.729	-0.00253	0.00138	3.729	-0.00297	0.00157	3.729	-0.00339	0.00175
3.73	-0.00156	9.21E-04	3.73	-0.00206	0.00116	3.73	-0.00253	0.00138	3.73	-0.00297	0.00157	3.73	-0.00338	0.00175
3.731	-0.00156	9.20E-04	3.731	-0.00206	0.00116	3.731	-0.00253	0.00138	3.731	-0.00297	0.00157	3.731	-0.00338	0.00175
3.732	-0.00156	9.20E-04	3.732	-0.00206	0.00116	3.732	-0.00253	0.00137	3.732	-0.00297	0.00157	3.732	-0.00338	0.00175
3.733	-0.00156	9.19E-04	3.733	-0.00206	0.00116	3.733	-0.00252	0.00137	3.733	-0.00296	0.00157	3.733	-0.00338	0.00175
3.734	-0.00156	9.18E-04	3.734	-0.00206	0.00116	3.734	-0.00252	0.00137	3.734	-0.00296	0.00157	3.734	-0.00337	0.00175
3.735	-0.00156	9.18E-04	3.735	-0.00205	0.00116	3.735	-0.00252	0.00137	3.735	-0.00296	0.00157	3.735	-0.00337	0.00175
3.736	-0.00156	9.17E-04	3.736	-0.00205	0.00116	3.736	-0.00252	0.00137	3.736	-0.00296	0.00157	3.736	-0.00337	0.00174
3.737	-0.00156	9.16E-04	3.737	-0.00205	0.00115	3.737	-0.00252	0.00137	3.737	-0.00295	0.00156	3.737	-0.00337	0.00174

3.738	-0.00156	9.16E-04	3.738	-0.00205	0.00115	3.738	-0.00252	0.00137	3.738	-0.00295	0.00156	3.738	-0.00336	0.00174
3.739	-0.00155	9.15E-04	3.739	-0.00205	0.00115	3.739	-0.00251	0.00137	3.739	-0.00295	0.00156	3.739	-0.00336	0.00174
3.74	-0.00155	9.14E-04	3.74	-0.00205	0.00115	3.74	-0.00251	0.00137	3.74	-0.00295	0.00156	3.74	-0.00336	0.00174
3.741	-0.00155	9.14E-04	3.741	-0.00205	0.00115	3.741	-0.00251	0.00137	3.741	-0.00295	0.00156	3.741	-0.00336	0.00174
3.742	-0.00155	9.13E-04	3.742	-0.00204	0.00115	3.742	-0.00251	0.00136	3.742	-0.00294	0.00156	3.742	-0.00335	0.00174
3.743	-0.00155	9.12E-04	3.743	-0.00204	0.00115	3.743	-0.00251	0.00136	3.743	-0.00294	0.00156	3.743	-0.00335	0.00173
3.744	-0.00155	9.12E-04	3.744	-0.00204	0.00115	3.744	-0.0025	0.00136	3.744	-0.00294	0.00156	3.744	-0.00335	0.00173
3.745	-0.00155	9.11E-04	3.745	-0.00204	0.00115	3.745	-0.0025	0.00136	3.745	-0.00294	0.00155	3.745	-0.00334	0.00173
3.746	-0.00155	9.10E-04	3.746	-0.00204	0.00115	3.746	-0.0025	0.00136	3.746	-0.00293	0.00155	3.746	-0.00334	0.00173
3.747	-0.00155	9.10E-04	3.747	-0.00204	0.00115	3.747	-0.0025	0.00136	3.747	-0.00293	0.00155	3.747	-0.00334	0.00173
3.748	-0.00154	9.09E-04	3.748	-0.00203	0.00115	3.748	-0.0025	0.00136	3.748	-0.00293	0.00155	3.748	-0.00334	0.00173
3.749	-0.00154	9.09E-04	3.749	-0.00203	0.00114	3.749	-0.00249	0.00136	3.749	-0.00293	0.00155	3.749	-0.00333	0.00173
3.75	-0.00154	9.08E-04	3.75	-0.00203	0.00114	3.75	-0.00249	0.00136	3.75	-0.00293	0.00155	3.75	-0.00333	0.00173
3.751	-0.00154	9.07E-04	3.751	-0.00203	0.00114	3.751	-0.00249	0.00135	3.751	-0.00292	0.00155	3.751	-0.00333	0.00172
3.752	-0.00154	9.07E-04	3.752	-0.00203	0.00114	3.752	-0.00249	0.00135	3.752	-0.00292	0.00155	3.752	-0.00333	0.00172
3.753	-0.00154	9.06E-04	3.753	-0.00203	0.00114	3.753	-0.00249	0.00135	3.753	-0.00292	0.00155	3.753	-0.00332	0.00172
3.754	-0.00154	9.05E-04	3.754	-0.00203	0.00114	3.754	-0.00248	0.00135	3.754	-0.00292	0.00154	3.754	-0.00332	0.00172
3.755	-0.00154	9.05E-04	3.755	-0.00202	0.00114	3.755	-0.00248	0.00135	3.755	-0.00291	0.00154	3.755	-0.00332	0.00172
3.756	-0.00154	9.04E-04	3.756	-0.00202	0.00114	3.756	-0.00248	0.00135	3.756	-0.00291	0.00154	3.756	-0.00332	0.00172
3.757	-0.00153	9.03E-04	3.757	-0.00202	0.00114	3.757	-0.00248	0.00135	3.757	-0.00291	0.00154	3.757	-0.00331	0.00172
3.758	-0.00153	9.03E-04	3.758	-0.00202	0.00114	3.758	-0.00248	0.00135	3.758	-0.00291	0.00154	3.758	-0.00331	0.00171
3.759	-0.00153	9.02E-04	3.759	-0.00202	0.00114	3.759	-0.00248	0.00135	3.759	-0.0029	0.00154	3.759	-0.00331	0.00171
3.76	-0.00153	9.01E-04	3.76	-0.00202	0.00114	3.76	-0.00247	0.00135	3.76	-0.0029	0.00154	3.76	-0.00331	0.00171
3.761	-0.00153	9.01E-04	3.761	-0.00202	0.00113	3.761	-0.00247	0.00134	3.761	-0.0029	0.00154	3.761	-0.0033	0.00171
3.762	-0.00153	9.00E-04	3.762	-0.00201	0.00113	3.762	-0.00247	0.00134	3.762	-0.0029	0.00153	3.762	-0.0033	0.00171
3.763	-0.00153	8.99E-04	3.763	-0.00201	0.00113	3.763	-0.00247	0.00134	3.763	-0.0029	0.00153	3.763	-0.0033	0.00171
3.764	-0.00153	8.99E-04	3.764	-0.00201	0.00113	3.764	-0.00247	0.00134	3.764	-0.00289	0.00153	3.764	-0.0033	0.00171
3.765	-0.00153	8.98E-04	3.765	-0.00201	0.00113	3.765	-0.00246	0.00134	3.765	-0.00289	0.00153	3.765	-0.00329	0.00171
3.766	-0.00152	8.97E-04	3.766	-0.00201	0.00113	3.766	-0.00246	0.00134	3.766	-0.00289	0.00153	3.766	-0.00329	0.0017
3.767	-0.00152	8.97E-04	3.767	-0.00201	0.00113	3.767	-0.00246	0.00134	3.767	-0.00289	0.00153	3.767	-0.00329	0.0017
3.768	-0.00152	8.96E-04	3.768	-0.002	0.00113	3.768	-0.00246	0.00134	3.768	-0.00288	0.00153	3.768	-0.00329	0.0017
3.769	-0.00152	8.96E-04	3.769	-0.002	0.00113	3.769	-0.00246	0.00134	3.769	-0.00288	0.00153	3.769	-0.00328	0.0017
3.77	-0.00152	8.95E-04	3.77	-0.002	0.00113	3.77	-0.00246	0.00134	3.77	-0.00288	0.00153	3.77	-0.00328	0.0017
3.771	-0.00152	8.94E-04	3.771	-0.002	0.00113	3.771	-0.00245	0.00133	3.771	-0.00288	0.00152	3.771	-0.00328	0.0017
3.772	-0.00152	8.94E-04	3.772	-0.002	0.00113	3.772	-0.00245	0.00133	3.772	-0.00288	0.00152	3.772	-0.00327	0.0017
3.773	-0.00152	8.93E-04	3.773	-0.002	0.00112	3.773	-0.00245	0.00133	3.773	-0.00287	0.00152	3.773	-0.00327	0.00169
3.774	-0.00152	8.92E-04	3.774	-0.002	0.00112	3.774	-0.00245	0.00133	3.774	-0.00287	0.00152	3.774	-0.00327	0.00169
3.775	-0.00151	8.92E-04	3.775	-0.00199	0.00112	3.775	-0.00245	0.00133	3.775	-0.00287	0.00152	3.775	-0.00327	0.00169
3.776	-0.00151	8.91E-04	3.776	-0.00199	0.00112	3.776	-0.00244	0.00133	3.776	-0.00287	0.00152	3.776	-0.00326	0.00169
3.777	-0.00151	8.90E-04	3.777	-0.00199	0.00112	3.777	-0.00244	0.00133	3.777	-0.00287	0.00152	3.777	-0.00326	0.00169
3.778	-0.00151	8.90E-04	3.778	-0.00199	0.00112	3.778	-0.00244	0.00133	3.778	-0.00286	0.00152	3.778	-0.00326	0.00169
3.779	-0.00151	8.89E-04	3.779	-0.00199	0.00112	3.779	-0.00244	0.00133	3.779	-0.00286	0.00151	3.779	-0.00326	0.00169
3.78	-0.00151	8.89E-04	3.78	-0.00199	0.00112	3.78	-0.00244	0.00133	3.78	-0.00286	0.00151	3.78	-0.00325	0.00169
3.781	-0.00151	8.88E-04	3.781	-0.00199	0.00112	3.781	-0.00243	0.00132	3.781	-0.00286	0.00151	3.781	-0.00325	0.00168
3.782	-0.00151	8.87E-04	3.782	-0.00198	0.00112	3.782	-0.00243	0.00132	3.782	-0.00285	0.00151	3.782	-0.00325	0.00168
3.783	-0.00151	8.87E-04	3.783	-0.00198	0.00112	3.783	-0.00243	0.00132	3.783	-0.00285	0.00151	3.783	-0.00325	0.00168
3.784	-0.0015	8.86E-04	3.784	-0.00198	0.00112	3.784	-0.00243	0.00132	3.784	-0.00285	0.00151	3.784	-0.00324	0.00168
3.785	-0.0015	8.85E-04	3.785	-0.00198	0.00111	3.785	-0.00243	0.00132	3.785	-0.00285	0.00151	3.785	-0.00324	0.00168
3.786	-0.0015	8.85E-04	3.786	-0.00198	0.00111	3.786	-0.00243	0.00132	3.786	-0.00285	0.00151	3.786	-0.00324	0.00168
3.787	-0.0015	8.84E-04	3.787	-0.00198	0.00111	3.787	-0.00242	0.00132	3.787	-0.00284	0.00151	3.787	-0.00324	0.00168
3.788	-0.0015	8.83E-04	3.788	-0.00198	0.00111	3.788	-0.00242	0.00132	3.788	-0.00284	0.0015	3.788	-0.00323	0.00167
3.789	-0.0015	8.83E-04	3.789	-0.00197	0.00111	3.789	-0.00242	0.00132	3.789	-0.00284	0.0015	3.789	-0.00323	0.00167
3.79	-0.0015	8.82E-04	3.79	-0.00197	0.00111	3.79	-0.00242	0.00132	3.79	-0.00284	0.0015	3.79	-0.00323	0.00167
3.791	-0.0015	8.82E-04	3.791	-0.00197	0.00111	3.791	-0.00242	0.00131	3.791	-0.00283	0.0015	3.791	-0.00323	0.00167
3.792	-0.0015	8.81E-04	3.792	-0.00197	0.00111	3.792	-0.00241	0.00131	3.792	-0.00283	0.0015	3.792	-0.00322	0.00167
3.793	-0.0015	8.80E-04	3.793	-0.00197	0.00111	3.793	-0.00241	0.00131	3.793	-0.00283	0.0015	3.793	-0.00322	0.00167
3.794	-0.00149	8.80E-04	3.794	-0.00197	0.00111	3.794	-0.00241	0.00131	3.794	-0.00283	0.0015	3.794	-0.00322	0.00167
3.795	-0.00149	8.79E-04	3.795	-0.00197	0.00111	3.795	-0.00241	0.00131	3.795	-0.00283	0.0015	3.795	-0.00322	0.00167
3.796	-0.00149	8.78E-04	3.796	-0.00196	0.00111	3.796	-0.00241	0.00131	3.796	-0.00282	0.0015	3.796	-0.00321	0.00166
3.797	-0.00149	8.78E-04	3.797	-0.00196	0.0011	3.797	-0.00241	0.00131	3.797	-0.00282	0.00149	3.797	-0.00321	0.00166
3.798	-0.00149	8.77E-04	3.798	-0.00196	0.0011	3.798	-0.0024	0.00131	3.798	-0.00282	0.00149	3.798	-0.00321	0.00166
3.799	-0.00149	8.77E-04	3.799	-0.00196	0.0011	3.799	-0.0024	0.00131	3.799	-0.00282	0.00149	3.799	-0.00321	0.00166
3.8	-0.00149	8.76E-04	3.8	-0.00196	0.0011	3.8	-0.0024	0.00131	3.8	-0.00282	0.00149	3.8	-0.0032	0.00166
3.801	-0.00149	8.75E-04	3.801	-0.00196	0.0011	3.801	-0.0024	0.0013	3.801	-0.00281	0.00149	3.801	-0.0032	0.00166
3.802	-0.00149	8.75E-04	3.802	-0.00196	0.0011	3.802	-0.0024	0.0013	3.802	-0.00281	0.00149	3.802	-0.0032	0.00166
3.803	-0.00148	8.74E-04	3.803	-0.00195	0.0011	3.803	-0.0024	0.0013	3.803	-0.00281	0.00149	3.803	-0.0032	0.00166
3.804	-0.00148	8.74E-04	3.804	-0.00195	0.0011	3.804	-0.00239	0.0013	3.804	-0.00281	0.00149	3.804	-0.00319	0.00165
3.805	-0.00148	8.73E-04	3.805	-0.00195	0.0011	3.805	-0.00239	0.0013	3.805	-0.0028	0.00148	3.805	-0.00319	0.00165
3.806	-0.00148	8.72E-04	3.806	-0.00195	0.0011	3.806	-0.00239	0.0013	3.806	-0.0028	0.00148	3.806	-0.00319	0.00165
3.807	-0.00148	8.72E-04	3.807	-0.00195	0.0011	3.807	-0.00239	0.0013	3.807	-0.0028	0.00148	3.807	-0.00319	0.00165
3.808	-0.00148	8.71E-04	3.808	-0.00195	0.0011	3.808	-0.00239	0.0013	3.808	-0.0028	0.00148	3.808	-0.00319	0.00165
3.809	-0.00148	8.70E-04	3.809	-0.00195	0.0011	3.809	-0.00238	0.0013	3.809	-0.0028	0.00148	3.809	-0.00318	0.00165
3.81	-0.00148	8.70E-04	3.81	-0.00194	0.00109	3.81	-0.00238	0.0013	3.81	-0.00279	0.00148	3.81	-0.00318	0.00165
3.811	-0.00148	8.69E-04	3.811	-0.00194	0.00109	3.811	-0.00238	0.0013	3.811	-0.00279	0.00148	3.811	-0.00318	0.00165
3.812	-0.00148	8.69E-04	3.812	-0.00194	0.00109	3.812	-0.00238	0.00129	3.812	-0.00279	0.00148	3.812	-0.00318	0.00164
3.813	-0.00147	8.68E-04	3.813	-0.00194	0.00109	3.813	-0.00238	0.00129	3.813	-0.00279	0.00148	3.813	-0.00317	0.00164
3.814	-0.00147	8.67E-04	3.814	-0.00194	0.00109	3.814	-0.00238	0.00129	3.814	-0.00279	0.00147	3.814	-0.00317	0.00164
3.815	-0.00147	8.67E-04	3.815	-0.00194	0.00109	3.815	-0.00237	0.00129	3.815	-0.00278	0.00147	3.815	-0.00317	0.00164
3.816	-0.00147	8.66E-04	3.816	-0.00194	0.00109	3.816	-0.00237	0.00129	3.816	-0.00278	0.00147	3.816	-0.00317	0.00164
3.817	-0.00147	8.66E-04	3.817	-0.00193	0.00109	3.817	-0.00237	0.00129	3.817	-0.00278	0.00147	3.817	-0.00316	0.00164
3.818	-0.00147	8.65E-04	3.818	-0.00193	0.00109	3.818	-0.00237	0.00129	3.818	-0.00278	0.00147	3.818	-0.00316	0.00164
3.819	-0.00147	8.64E-04	3.819	-0.00193	0.00109	3.819	-0.00237	0.00129	3.819	-0.00278	0.00147	3.819	-0.00316	0.00164
3.82	-0.00147	8.64E-04	3.82	-0.00193	0.00109	3.82	-0.00236	0.00129	3.82	-0.00277	0.00147	3.82	-0.00316	0.00163
3.821	-0.00147	8.63E-04	3.821	-0.00193	0.00109	3.821	-0.00236	0.00129	3.821	-0.00277	0.00147	3.821	-0.00315	0.00163
3.822	-0.00146	8.62E-04	3.822	-0.00193	0.00109	3.822	-0.00236	0.00128	3.822	-0.00277	0.00147	3.822	-0.00315	0.00163
3.823	-0.00146	8.62E-04	3.823	-0.00193	0.00108	3.823	-0.00236	0.00128	3.823	-0.00277	0.00146	3.823	-0.00315	0.00163
3.824	-0.00146	8.61E-04	3.824	-0.00192	0.00108	3.824	-0.00236	0.00128	3.824	-0.00276	0.00146	3.824	-0.00315	0.00163
3.825	-0.00146	8.61E-04	3.825	-0.00192	0.00108	3.825	-0.00236	0.00128	3.825	-0.00276	0.00146	3.825	-0.00314	0.00163
3.826	-0.00146	8.60E-04	3.826	-0.00192	0.00108	3.826	-0.00235	0.00128	3.826	-0.00276	0.00146	3.826	-0.00314	0.00163
3.827	-0.00146	8.59E-04	3.827	-0.00192	0.00108	3.827	-0.00235	0.00128	3.827	-0.00276	0.00146	3.827	-0.00314	0.00163
3.828	-0.00146	8.59E-04	3.828	-0.00192	0.00108	3.828	-0.00235	0.00128	3.828	-0.00276	0.00146	3.828	-0.00314	0.00162
3.829	-0.00146	8.58E-04	3.829	-0.00192	0.00108	3.829	-0.00235	0.00128	3.829	-0.00275	0.00146	3.829	-0.00313	0.00162
3.83	-0.00146	8.58E-04	3.83	-0.00192	0.00108	3.83	-0.00235	0.00128	3.83	-0.00275	0.00146	3.83	-0.00313	0.00162
3.831	-0.00146	8.57E-04	3.831	-0.00191	0.00108	3.831	-0.00235	0.00128	3.831	-0.00275	0.00146	3.831	-0.00313	0.00162
3.832	-0.00145	8.56E-04	3.832	-0.00191	0.00108	3.832	-0.00234	0.00128	3.832	-0.00275	0.00145	3.832	-0.00313	0.00162
3.833	-0.00145	8.56E-04	3.833	-0.00191	0.00108	3.833	-0.00234	0.00127	3.833	-0.00275	0.00145	3.833	-0.00312	0.00162
3.834	-0.00145	8.55E-04	3.834	-0.00191	0.00108	3.834	-0.00234	0.00127	3.834	-0.00274	0.00145	3.834	-0.00312	0.00162
3.835	-0.00145	8.55E-04	3.835	-0.00191	0.00107	3.835	-0.00234	0.00127	3.835	-0.00274	0.00145	3.835	-0.00312	0.00162
3.836	-0.00145	8.54E-04	3.836	-0.00191	0.00107	3.836	-0.00234	0.00127	3.836	-0.00274	0.00145	3.836	-0.00312	0.00161
3.837	-0.00145	8.53E-04	3.837	-0.00191	0.00107	3.837	-0.00234	0.00127	3.837	-0.00274	0.00145	3.837	-0.00312	0.00161
3.838	-0.00145	8.53E-04	3.838	-0.0019	0.00107	3.838	-0.00233	0.00127	3.838	-0.00274	0.00145	3.838	-0.00311	0.00161
3.839	-0.00145	8.52E-04	3.839	-0.0019	0.00107	3.839	-0.00233	0.00127	3.839	-0.00273	0.00145	3.839	-0.00311	0.00161
3.84	-0.00145	8.52E-04	3.84	-0.0019	0.00107	3.84	-0.00233	0.00127	3.84	-0.00273	0.00145	3.84	-0.00311	0.00161
3.841	-0.00145	8.51E-04	3.841	-0.0019	0.00107	3.841	-0.00233	0.00127	3.841	-0.00273	0.00145	3.841	-0.00311	0.00161
3.842	-0.00144	8.50E-04	3.842	-0.0019	0.00107	3.842	-0.00233	0.00127	3.842	-0.00273	0.00144	3.842	-0.0031	0.00161
3.843	-0.00144	8.50E-04	3.843	-0.0019	0.00107	3.843	-0.00233	0.00126	3.843	-0.00273	0.00144	3.843	-0.0031	0.00161
3.844	-0.00144	8.49E-04	3.844	-0.0019	0.00107	3.844	-0.00232	0.00126	3.844	-0.00272	0.00144	3.844	-0.0031	0.0016
3.845	-0.00144	8.49E-04	3.845	-0.0019	0.00107	3.845	-0.00232	0.00126	3.845	-0.00272	0.00144	3.845	-0.0031	0.0016
3.846	-0.00144	8.48E-04	3.846	-0.00189	0.00107	3.846	-0.00232	0.00126	3.846	-0.00272	0.00144	3.846	-0.00309	0.0016
3.847	-0.00144	8.47E-04	3.847	-0.00189	0.00107	3.847	-0.00232	0.00126	3.847	-0.00272	0.00144	3.847	-0.00309	0.0016
3.848	-0.00144	8.47E-04	3.848	-0.00189	0.00106	3.848	-0.00232	0.00126	3.848	-0.00272	0.00144	3.848	-0.00309	0.0016
3.849	-0.00144	8.46E-04	3.849	-0.00189	0.00106	3.849	-0.00231	0.00126	3.849	-0.00271	0.00144	3.849	-0.00309	0.0016
3.85	-0.00144	8.46E-04	3.85	-0.00189	0.00106	3.85	-0.00231	0.00126	3.85	-0.00271	0.00144	3.85	-0.00308	0.0016
3.851	-0.00144	8.45E-04	3.851	-0.00189	0.00106	3.851	-0.00231	0.00126	3.851	-0.00271	0.00143	3.851	-0.00308	0.0016
3.852	-0.00143	8.44E-04	3.852	-0.00189	0.00106	3.852	-0.00231	0.00126	3.852	-0.00271	0.00143	3.852	-0.00308	0.00159
3.853	-0.00143	8.44E-04	3.853	-0.00188	0.00106	3.853	-0.00231	0.00126	3.853	-0.0027	0.00143	3.853	-0.00308	0.00159
3.854	-0.00143	8.43E-04	3.854	-0.00188	0.00106	3.854	-0.00231	0.00125	3.854	-0.0027	0.00143	3.854	-0.00307	0.00159
3.855	-0.00143	8.43E-04	3.855	-0.00188	0.00106	3.855	-0.0023	0.00125	3.855	-0.0027	0.00143	3.855	-0.00307	0.00159
3.856	-0.00143	8.42E-04	3.856	-0.00188	0.00106	3.856	-0.0023	0.00125	3.856	-0.0027	0.00143	3.856	-0.00307	0.00159
3.857	-0.00143	8.41E-04	3.857	-0.00188	0.00106	3.857	-0.0023	0.00125	3.857	-0.0027	0.00143	3.857	-0.00307	0.00159
3.858	-0.00143	8.41E-04	3.858	-0.00188	0.00106	3.858	-0.0023	0.00125	3.858	-0.00269	0.00143	3.858	-0.00307	0.00159
3.859	-0.00143	8.40E-04	3.859	-0.00188	0.00106	3.859	-0.0023	0.00125	3.859	-0.00269	0.00143	3.859	-0.00306	0.00159
3.86	-0.00143	8.40E-04	3.86	-0.00187	0.00106	3.86	-0.0023	0.00125	3.86	-0.00269	0.00142	3.86	-0.00306	0.00158
3.861	-0.00143	8.39E-04	3.861	-0.00187	0.00105	3.861	-0.00229	0.00125	3.861	-0.00269	0.00142	3.861	-0.00306	0.00158
3.862	-0.00142	8.39E-04	3.862	-0.00187	0.00105	3.862	-0.00229	0.00125	3.862	-0.00269	0.00142	3.862	-0.00306	0.00158
3.863	-0.00142	8.38E-04	3.863	-0.00187	0.00105	3.863	-0.00229	0.00125	3.863	-0.00268	0.00142	3.863	-0.00305	0.00158
3.864	-0.00142	8.37E-04	3.864	-0.00187	0.00105	3.864	-0.00229	0.00125	3.864	-0.00268	0.00142	3.864	-0.00305	0.00158
3.865	-0.00142	8.37E-04	3.865	-0.00187	0.00105	3.865	-0.00229	0.00124	3.865	-0.00268	0.00142	3.865	-0.00305	0.00158
3.866	-0.00142	8.36E-04	3.866	-0.00187	0.00105	3.866	-0.00229	0.00124	3.866	-0.00268	0.00142	3.866	-0.00305	0.00158
3.867	-0.00142	8.36E-04	3.867	-0.00187	0.00105	3.867	-0.00228	0.00124	3.867	-0.00268	0.00142	3.867	-0.00304	0.00158
3.868	-0.00142	8.35E-04	3.868	-0.00186	0.00105	3.868	-0.00228	0.00124	3.868	-0.00267	0.00142	3.868	-0.00304	0.00158
3.869	-0.00142	8.34E-04	3.869	-0.00186	0.00105	3.869	-0.00228	0.00124	3.869	-0.00267	0.00141	3.869	-0.00304	0.00157
3.87	-0.00142	8.34E-04	3.87	-0.00186	0.00105	3.87	-0.00228	0.00124	3.87	-0.00267	0.00141	3.87	-0.00304	0.00157
3.871	-0.00142	8.33E-04	3.871	-0.00186	0.00105	3.871	-0.00228	0.00124	3.871	-0.00267	0.00141	3.871	-0.00304	0.00157
3.872	-0.00141	8.33E-04	3.872	-0.00186	0.00105	3.872	-0.00228	0.00124	3.872	-0.00267	0.00141	3.872	-0.00303	0.00157
3.873	-0.00141	8.32E-04	3.873	-0.00186	0.00105	3.873	-0.00227	0.00124	3.873	-0.00266	0.00141	3.873	-0.00303	0.00157
3.874	-0.00141	8.32E-04	3.874	-0.00186	0.00105	3.874	-0.00227	0.00124	3.874	-0.00266	0.00141	3.874	-0.00303	0.00157
3.875	-0.00141	8.31E-04	3.875	-0.00185	0.00104	3.875	-0.00227	0.00124	3.875	-0.00266	0.00141	3.875	-0.00303	0.00157
3.876	-0.00141	8.30E-04	3.876	-0.00185	0.00104	3.876	-0.00227	0.00123	3.876	-0.00266	0.00141	3.876	-0.00302	0.00157
3.877	-0.00141	8.30E-04	3.877	-0.00185	0.00104	3.877	-0.00227	0.00123	3.877	-0.00266	0.00141	3.877	-0.00302	0.00156
3.878	-0.00141	8.29E-04	3.878	-0.00185	0.00104	3.878	-0.00227	0.00123	3.878	-0.00265	0.00141	3.878	-0.00302	0.00156
3.879	-0.00141	8.29E-04	3.879	-0.00185	0.00104	3.879	-0.00226	0.00123	3.879	-0.00265	0.0014	3.879	-0.00302	0.00156
3.88	-0.00141	8.28E-04	3.88	-0.00185	0.00104	3.88	-0.00226	0.00123	3.88	-0.00265	0.0014	3.88	-0.00301	0.00156
3.881	-0.00141	8.28E-04	3.881	-0.00185	0.00104	3.881	-0.00226	0.00123	3.881	-0.00265	0.0014	3.881	-0.00301	0.00156
3.882	-0.0014	8.27E-04	3.882	-0.00185	0.00104	3.882	-0.00226	0.00123	3.882	-0.00265	0.0014	3.882	-0.00301	0.00156
3.883	-0.0014	8.26E-04	3.883	-0.00184	0.00104	3.883	-0.00226	0.00123	3.883	-0.00265	0.0014	3.883	-0.00301	0.00156
3.884	-0.0014	8.26E-04	3.884	-0.00184	0.00104	3.884	-0.00226	0.00123	3.884	-0.00264	0.0014	3.884	-0.00301	0.00156
3.885	-0.0014	8.25E-04	3.885	-0.00184	0.00104	3.885	-0.00225	0.00123	3.885	-0.00264	0.0014	3.885	-0.003	0.00156
3.886	-0.0014	8.25E-04	3.886	-0.00184	0.00104	3.886	-0.00225	0.00123	3.886	-0.00264	0.0014	3.886	-0.003	0.00155
3.887	-0.0014	8.24E-04	3.887	-0.00184	0.00104	3.887	-0.00225	0.00122	3.887	-0.00264	0.0014	3.887	-0.003	0.00155
3.888	-0.0014	8.24E-04	3.888	-0.00184	0.00103	3.888	-0.00225	0.00122	3.888	-0.00264	0.0014	3.888	-0.003	0.00155
3.889	-0.0014	8.23E-04	3.889	-0.00184	0.00103	3.889	-0.00225	0.00122	3.889	-0.00263	0.00139	3.889	-0.00299	0.00155
3.89	-0.0014	8.22E-04	3.89	-0.00183	0.00103	3.89	-0.00225	0.00122	3.89	-0.00263	0.00139	3.89	-0.00299	0.00155
3.891	-0.0014	8.22E-04	3.891	-0.00183	0.00103	3.891	-0.00224	0.00122	3.891	-0.00263	0.00139	3.891	-0.00299	0.00155
3.892	-0.00139	8.21E-04	3.892	-0.00183	0.00103	3.892	-0.00224	0.00122	3.892	-0.00263	0.00139	3.892	-0.00299	0.00155
3.893	-0.00139	8.21E-04	3.893	-0.00183	0.00103	3.893	-0.00224	0.00122	3.893	-0.00263	0.00139	3.893	-0.00299	0.00155
3.894	-0.00139	8.20E-04	3.894	-0.00183	0.00103	3.894	-0.00224	0.00122	3.894	-0.00262	0.00139	3.894	-0.00298	0.00154
3.895	-0.00139	8.20E-04	3.895	-0.00183	0.00103	3.895	-0.00224	0.00122	3.895	-0.00262	0.00139	3.895	-0.00298	0.00154
3.896	-0.00139	8.19E-04	3.896	-0.00183	0.00103	3.896	-0.00224	0.00122	3.896	-0.00262	0.00139	3.896	-0.00298	0.00154
3.897	-0.00139	8.18E-04	3.897	-0.00183	0.00103	3.897	-0.00223	0.00122	3.897	-0.00262	0.00139	3.897	-0.00298	0.00154
3.898	-0.00139	8.18E-04	3.898	-0.00182	0.00103	3.898	-0.00223	0.00121	3.898	-0.00262	0.00138	3.898	-0.00297	0.00154
3.899	-0.00139	8.17E-04	3.899	-0.00182	0.00103	3.899	-0.00223	0.00121	3.899	-0.00261	0.00138	3.899	-0.00297	0.00154
3.9	-0.00139	8.17E-04	3.9	-0.00182	0.00103	3.9	-0.00223	0.00121	3.9	-0.00261	0.00138	3.9	-0.00297	0.00154
3.901	-0.00139	8.16E-04	3.901	-0.00182	0.00103	3.901	-0.00223	0.00121	3.901	-0.00261	0.00138	3.901	-0.00297	0.00154
3.902	-0.00139	8.16E-04	3.902	-0.00182	0.00102	3.902	-0.00223	0.00121	3.902	-0.00261	0.00138	3.902	-0.00297	0.00154
3.903	-0.00138	8.15E-04	3.903	-0.00182	0.00102	3.903	-0.00223	0.00121	3.903	-0.00261	0.00138	3.903	-0.00296	0.00153
3.904	-0.00138	8.14E-04	3.904	-0.00182	0.00102	3.904	-0.00222	0.00121	3.904	-0.0026	0.00138	3.904	-0.00296	0.00153
3.905	-0.00138	8.14E-04	3.905	-0.00182	0.00102	3.905	-0.00222	0.00121	3.905	-0.0026	0.00138	3.905	-0.00296	0.00153
3.906	-0.00138	8.13E-04	3.906	-0.00181	0.00102	3.906	-0.00222	0.00121	3.906	-0.0026	0.00138	3.906	-0.00296	0.00153
3.907	-0.00138	8.13E-04	3.907	-0.00181	0.00102	3.907	-0.00222	0.00121	3.907	-0.0026	0.00138	3.907	-0.00295	0.00153
3.908	-0.00138	8.12E-04	3.908	-0.00181	0.00102	3.908	-0.00222	0.00121	3.908	-0.0026	0.00137	3.908	-0.00295	0.00153
3.909	-0.00138	8.12E-04	3.909	-0.00181	0.00102	3.909	-0.00222	0.00121	3.909	-0.00259	0.00137	3.909	-0.00295	0.00153
3.91	-0.00138	8.11E-04	3.91	-0.00181	0.00102	3.91	-0.00221	0.0012	3.91	-0.00259	0.00137	3.91	-0.00295	0.00153
3.911	-0.00138	8.11E-04	3.911	-0.00181	0.00102	3.911	-0.00221	0.0012	3.911	-0.00259	0.00137	3.911	-0.00295	0.00153
3.912	-0.00138	8.10E-04	3.912	-0.00181	0.00102	3.912	-0.00221	0.0012	3.912	-0.00259	0.00137	3.912	-0.00294	0.00152
3.913	-0.00137	8.09E-04	3.913	-0.00181	0.00102	3.913	-0.00221	0.0012	3.913	-0.00259	0.00137	3.913	-0.00294	0.00152
3.914	-0.00137	8.09E-04	3.914	-0.0018	0.00102	3.914	-0.00221	0.0012	3.914	-0.00258	0.00137	3.914	-0.00294	0.00152
3.915	-0.00137	8.08E-04	3.915	-0.0018	0.00101	3.915	-0.00221	0.0012	3.915	-0.00258	0.00137	3.915	-0.00294	0.00152
3.916	-0.00137	8.08E-04	3.916	-0.0018	0.00101	3.916	-0.0022	0.0012	3.916	-0.00258	0.00137	3.916	-0.00293	0.00152
3.917	-0.00137	8.07E-04	3.917	-0.0018	0.00101	3.917	-0.0022	0.0012	3.917	-0.00258	0.00137	3.917	-0.00293	0.00152
3.918	-0.00137	8.07E-04	3.918	-0.0018	0.00101	3.918	-0.0022	0.0012	3.918	-0.00258	0.00136	3.918	-0.00293	0.00152
3.919	-0.00137	8.06E-04	3.919	-0.0018	0.00101	3.919	-0.0022	0.0012	3.919	-0.00258	0.00136	3.919	-0.00293	0.00152
3.92	-0.00137	8.05E-04	3.92	-0.0018	0.00101	3.92	-0.0022	0.0012	3.92	-0.00257	0.00136	3.92	-0.00293	0.00151
3.921	-0.00137	8.05E-04	3.921	-0.00179	0.00101	3.921	-0.0022	0.00119	3.921	-0.00257	0.00136	3.921	-0.00292	0.00151
3.922	-0.00137	8.04E-04	3.922	-0.00179	0.00101	3.922	-0.00219	0.00119	3.922	-0.00257	0.00136	3.922	-0.00292	0.00151

3.923	-0.00137	8.04E-04	3.923	-0.00179	0.00101	3.923	-0.00219	0.00119	3.923	-0.00257	0.00136	3.923	-0.00292	0.00151
3.924	-0.00136	8.03E-04	3.924	-0.00179	0.00101	3.924	-0.00219	0.00119	3.924	-0.00257	0.00136	3.924	-0.00292	0.00151
3.925	-0.00136	8.03E-04	3.925	-0.00179	0.00101	3.925	-0.00219	0.00119	3.925	-0.00256	0.00136	3.925	-0.00291	0.00151
3.926	-0.00136	8.02E-04	3.926	-0.00179	0.00101	3.926	-0.00219	0.00119	3.926	-0.00256	0.00136	3.926	-0.00291	0.00151
3.927	-0.00136	8.02E-04	3.927	-0.00179	0.00101	3.927	-0.00219	0.00119	3.927	-0.00256	0.00136	3.927	-0.00291	0.00151
3.928	-0.00136	8.01E-04	3.928	-0.00179	0.00101	3.928	-0.00219	0.00119	3.928	-0.00256	0.00135	3.928	-0.00291	0.00151
3.929	-0.00136	8.01E-04	3.929	-0.00178	0.001	3.929	-0.00218	0.00119	3.929	-0.00256	0.00135	3.929	-0.00291	0.0015
3.93	-0.00136	8.00E-04	3.93	-0.00178	0.001	3.93	-0.00218	0.00119	3.93	-0.00255	0.00135	3.93	-0.0029	0.0015
3.931	-0.00136	7.99E-04	3.931	-0.00178	0.001	3.931	-0.00218	0.00119	3.931	-0.00255	0.00135	3.931	-0.0029	0.0015
3.932	-0.00136	7.99E-04	3.932	-0.00178	0.001	3.932	-0.00218	0.00119	3.932	-0.00255	0.00135	3.932	-0.0029	0.0015
3.933	-0.00136	7.98E-04	3.933	-0.00178	0.001	3.933	-0.00218	0.00118	3.933	-0.00255	0.00135	3.933	-0.0029	0.0015
3.934	-0.00135	7.98E-04	3.934	-0.00178	0.001	3.934	-0.00218	0.00118	3.934	-0.00255	0.00135	3.934	-0.0029	0.0015
3.935	-0.00135	7.97E-04	3.935	-0.00178	0.001	3.935	-0.00217	0.00118	3.935	-0.00255	0.00135	3.935	-0.00289	0.0015
3.936	-0.00135	7.97E-04	3.936	-0.00178	0.001	3.936	-0.00217	0.00118	3.936	-0.00254	0.00135	3.936	-0.00289	0.0015
3.937	-0.00135	7.96E-04	3.937	-0.00177	9.99E-04	3.937	-0.00217	0.00118	3.937	-0.00254	0.00135	3.937	-0.00289	0.0015
3.938	-0.00135	7.96E-04	3.938	-0.00177	9.99E-04	3.938	-0.00217	0.00118	3.938	-0.00254	0.00134	3.938	-0.00289	0.00149
3.939	-0.00135	7.95E-04	3.939	-0.00177	9.98E-04	3.939	-0.00217	0.00118	3.939	-0.00254	0.00134	3.939	-0.00288	0.00149
3.94	-0.00135	7.95E-04	3.94	-0.00177	9.97E-04	3.94	-0.00217	0.00118	3.94	-0.00254	0.00134	3.94	-0.00288	0.00149
3.941	-0.00135	7.94E-04	3.941	-0.00177	9.96E-04	3.941	-0.00216	0.00118	3.941	-0.00253	0.00134	3.941	-0.00288	0.00149
3.942	-0.00135	7.93E-04	3.942	-0.00177	9.96E-04	3.942	-0.00216	0.00118	3.942	-0.00253	0.00134	3.942	-0.00288	0.00149
3.943	-0.00135	7.93E-04	3.943	-0.00177	9.95E-04	3.943	-0.00216	0.00118	3.943	-0.00253	0.00134	3.943	-0.00288	0.00149
3.944	-0.00135	7.92E-04	3.944	-0.00177	9.94E-04	3.944	-0.00216	0.00118	3.944	-0.00253	0.00134	3.944	-0.00287	0.00149
3.945	-0.00134	7.92E-04	3.945	-0.00176	9.94E-04	3.945	-0.00216	0.00117	3.945	-0.00253	0.00134	3.945	-0.00287	0.00149
3.946	-0.00134	7.91E-04	3.946	-0.00176	9.93E-04	3.946	-0.00216	0.00117	3.946	-0.00252	0.00134	3.946	-0.00287	0.00149
3.947	-0.00134	7.91E-04	3.947	-0.00176	9.92E-04	3.947	-0.00216	0.00117	3.947	-0.00252	0.00134	3.947	-0.00287	0.00148
3.948	-0.00134	7.90E-04	3.948	-0.00176	9.92E-04	3.948	-0.00215	0.00117	3.948	-0.00252	0.00133	3.948	-0.00287	0.00148
3.949	-0.00134	7.90E-04	3.949	-0.00176	9.91E-04	3.949	-0.00215	0.00117	3.949	-0.00252	0.00133	3.949	-0.00286	0.00148
3.95	-0.00134	7.89E-04	3.95	-0.00176	9.90E-04	3.95	-0.00215	0.00117	3.95	-0.00252	0.00133	3.95	-0.00286	0.00148
3.951	-0.00134	7.89E-04	3.951	-0.00176	9.90E-04	3.951	-0.00215	0.00117	3.951	-0.00252	0.00133	3.951	-0.00286	0.00148
3.952	-0.00134	7.88E-04	3.952	-0.00176	9.89E-04	3.952	-0.00215	0.00117	3.952	-0.00251	0.00133	3.952	-0.00286	0.00148
3.953	-0.00134	7.87E-04	3.953	-0.00175	9.88E-04	3.953	-0.00215	0.00117	3.953	-0.00251	0.00133	3.953	-0.00285	0.00148
3.954	-0.00134	7.87E-04	3.954	-0.00175	9.87E-04	3.954	-0.00214	0.00117	3.954	-0.00251	0.00133	3.954	-0.00285	0.00148
3.955	-0.00134	7.86E-04	3.955	-0.00175	9.87E-04	3.955	-0.00214	0.00117	3.955	-0.00251	0.00133	3.955	-0.00285	0.00148
3.956	-0.00133	7.86E-04	3.956	-0.00175	9.86E-04	3.956	-0.00214	0.00117	3.956	-0.00251	0.00133	3.956	-0.00285	0.00147
3.957	-0.00133	7.85E-04	3.957	-0.00175	9.85E-04	3.957	-0.00214	0.00116	3.957	-0.0025	0.00133	3.957	-0.00285	0.00147
3.958	-0.00133	7.85E-04	3.958	-0.00175	9.85E-04	3.958	-0.00214	0.00116	3.958	-0.0025	0.00133	3.958	-0.00284	0.00147
3.959	-0.00133	7.84E-04	3.959	-0.00175	9.84E-04	3.959	-0.00214	0.00116	3.959	-0.0025	0.00132	3.959	-0.00284	0.00147
3.96	-0.00133	7.84E-04	3.96	-0.00175	9.83E-04	3.96	-0.00214	0.00116	3.96	-0.0025	0.00132	3.96	-0.00284	0.00147
3.961	-0.00133	7.83E-04	3.961	-0.00175	9.83E-04	3.961	-0.00213	0.00116	3.961	-0.0025	0.00132	3.961	-0.00284	0.00147
3.962	-0.00133	7.83E-04	3.962	-0.00174	9.82E-04	3.962	-0.00213	0.00116	3.962	-0.0025	0.00132	3.962	-0.00284	0.00147
3.963	-0.00133	7.82E-04	3.963	-0.00174	9.81E-04	3.963	-0.00213	0.00116	3.963	-0.00249	0.00132	3.963	-0.00283	0.00147
3.964	-0.00133	7.82E-04	3.964	-0.00174	9.81E-04	3.964	-0.00213	0.00116	3.964	-0.00249	0.00132	3.964	-0.00283	0.00147
3.965	-0.00133	7.81E-04	3.965	-0.00174	9.80E-04	3.965	-0.00213	0.00116	3.965	-0.00249	0.00132	3.965	-0.00283	0.00147
3.966	-0.00133	7.81E-04	3.966	-0.00174	9.79E-04	3.966	-0.00213	0.00116	3.966	-0.00249	0.00132	3.966	-0.00283	0.00146
3.967	-0.00132	7.80E-04	3.967	-0.00174	9.78E-04	3.967	-0.00212	0.00116	3.967	-0.00249	0.00132	3.967	-0.00283	0.00146
3.968	-0.00132	7.79E-04	3.968	-0.00174	9.78E-04	3.968	-0.00212	0.00116	3.968	-0.00248	0.00132	3.968	-0.00282	0.00146
3.969	-0.00132	7.79E-04	3.969	-0.00174	9.77E-04	3.969	-0.00212	0.00115	3.969	-0.00248	0.00131	3.969	-0.00282	0.00146
3.97	-0.00132	7.78E-04	3.97	-0.00173	9.76E-04	3.97	-0.00212	0.00115	3.97	-0.00248	0.00131	3.97	-0.00282	0.00146
3.971	-0.00132	7.78E-04	3.971	-0.00173	9.76E-04	3.971	-0.00212	0.00115	3.971	-0.00248	0.00131	3.971	-0.00282	0.00146
3.972	-0.00132	7.77E-04	3.972	-0.00173	9.75E-04	3.972	-0.00212	0.00115	3.972	-0.00248	0.00131	3.972	-0.00281	0.00146
3.973	-0.00132	7.77E-04	3.973	-0.00173	9.74E-04	3.973	-0.00212	0.00115	3.973	-0.00248	0.00131	3.973	-0.00281	0.00146
3.974	-0.00132	7.76E-04	3.974	-0.00173	9.74E-04	3.974	-0.00211	0.00115	3.974	-0.00247	0.00131	3.974	-0.00281	0.00146
3.975	-0.00132	7.76E-04	3.975	-0.00173	9.73E-04	3.975	-0.00211	0.00115	3.975	-0.00247	0.00131	3.975	-0.00281	0.00145
3.976	-0.00132	7.75E-04	3.976	-0.00173	9.72E-04	3.976	-0.00211	0.00115	3.976	-0.00247	0.00131	3.976	-0.00281	0.00145
3.977	-0.00132	7.75E-04	3.977	-0.00173	9.72E-04	3.977	-0.00211	0.00115	3.977	-0.00247	0.00131	3.977	-0.0028	0.00145
3.978	-0.00131	7.74E-04	3.978	-0.00172	9.71E-04	3.978	-0.00211	0.00115	3.978	-0.00247	0.00131	3.978	-0.0028	0.00145
3.979	-0.00131	7.74E-04	3.979	-0.00172	9.70E-04	3.979	-0.00211	0.00115	3.979	-0.00246	0.0013	3.979	-0.0028	0.00145
3.98	-0.00131	7.73E-04	3.98	-0.00172	9.70E-04	3.98	-0.00211	0.00115	3.98	-0.00246	0.0013	3.98	-0.0028	0.00145
3.981	-0.00131	7.73E-04	3.981	-0.00172	9.69E-04	3.981	-0.0021	0.00114	3.981	-0.00246	0.0013	3.981	-0.0028	0.00145
3.982	-0.00131	7.72E-04	3.982	-0.00172	9.68E-04	3.982	-0.0021	0.00114	3.982	-0.00246	0.0013	3.982	-0.00279	0.00145
3.983	-0.00131	7.72E-04	3.983	-0.00172	9.68E-04	3.983	-0.0021	0.00114	3.983	-0.00246	0.0013	3.983	-0.00279	0.00145
3.984	-0.00131	7.71E-04	3.984	-0.00172	9.67E-04	3.984	-0.0021	0.00114	3.984	-0.00246	0.0013	3.984	-0.00279	0.00144
3.985	-0.00131	7.71E-04	3.985	-0.00172	9.66E-04	3.985	-0.0021	0.00114	3.985	-0.00245	0.0013	3.985	-0.00279	0.00144
3.986	-0.00131	7.70E-04	3.986	-0.00171	9.66E-04	3.986	-0.0021	0.00114	3.986	-0.00245	0.0013	3.986	-0.00279	0.00144
3.987	-0.00131	7.69E-04	3.987	-0.00171	9.65E-04	3.987	-0.00209	0.00114	3.987	-0.00245	0.0013	3.987	-0.00278	0.00144
3.988	-0.00131	7.69E-04	3.988	-0.00171	9.64E-04	3.988	-0.00209	0.00114	3.988	-0.00245	0.0013	3.988	-0.00278	0.00144
3.989	-0.00131	7.68E-04	3.989	-0.00171	9.64E-04	3.989	-0.00209	0.00114	3.989	-0.00245	0.0013	3.989	-0.00278	0.00144
3.99	-0.0013	7.68E-04	3.99	-0.00171	9.63E-04	3.99	-0.00209	0.00114	3.99	-0.00245	0.00129	3.99	-0.00278	0.00144
3.991	-0.0013	7.67E-04	3.991	-0.00171	9.62E-04	3.991	-0.00209	0.00114	3.991	-0.00244	0.00129	3.991	-0.00278	0.00144
3.992	-0.0013	7.67E-04	3.992	-0.00171	9.62E-04	3.992	-0.00209	0.00114	3.992	-0.00244	0.00129	3.992	-0.00277	0.00144
3.993	-0.0013	7.66E-04	3.993	-0.00171	9.61E-04	3.993	-0.00209	0.00113	3.993	-0.00244	0.00129	3.993	-0.00277	0.00144
3.994	-0.0013	7.66E-04	3.994	-0.00171	9.60E-04	3.994	-0.00208	0.00113	3.994	-0.00244	0.00129	3.994	-0.00277	0.00143
3.995	-0.0013	7.65E-04	3.995	-0.0017	9.60E-04	3.995	-0.00208	0.00113	3.995	-0.00244	0.00129	3.995	-0.00277	0.00143
3.996	-0.0013	7.65E-04	3.996	-0.0017	9.59E-04	3.996	-0.00208	0.00113	3.996	-0.00243	0.00129	3.996	-0.00277	0.00143
3.997	-0.0013	7.64E-04	3.997	-0.0017	9.58E-04	3.997	-0.00208	0.00113	3.997	-0.00243	0.00129	3.997	-0.00276	0.00143
3.998	-0.0013	7.64E-04	3.998	-0.0017	9.58E-04	3.998	-0.00208	0.00113	3.998	-0.00243	0.00129	3.998	-0.00276	0.00143
3.999	-0.0013	7.63E-04	3.999	-0.0017	9.57E-04	3.999	-0.00208	0.00113	3.999	-0.00243	0.00129	3.999	-0.00276	0.00143
4	-0.0013	7.63E-04	4	-0.0017	9.56E-04	4	-0.00208	0.00113	4	-0.00243	0.00129	4	-0.00276	0.00143
4.001	-0.00129	7.62E-04	4.001	-0.0017	9.56E-04	4.001	-0.00207	0.00113	4.001	-0.00243	0.00128	4.001	-0.00276	0.00143
4.002	-0.00129	7.62E-04	4.002	-0.0017	9.55E-04	4.002	-0.00207	0.00113	4.002	-0.00242	0.00128	4.002	-0.00275	0.00143
4.003	-0.00129	7.61E-04	4.003	-0.00169	9.54E-04	4.003	-0.00207	0.00113	4.003	-0.00242	0.00128	4.003	-0.00275	0.00142
4.004	-0.00129	7.61E-04	4.004	-0.00169	9.54E-04	4.004	-0.00207	0.00113	4.004	-0.00242	0.00128	4.004	-0.00275	0.00142
4.005	-0.00129	7.60E-04	4.005	-0.00169	9.53E-04	4.005	-0.00207	0.00113	4.005	-0.00242	0.00128	4.005	-0.00275	0.00142
4.006	-0.00129	7.60E-04	4.006	-0.00169	9.52E-04	4.006	-0.00207	0.00112	4.006	-0.00242	0.00128	4.006	-0.00275	0.00142
4.007	-0.00129	7.59E-04	4.007	-0.00169	9.52E-04	4.007	-0.00206	0.00112	4.007	-0.00242	0.00128	4.007	-0.00274	0.00142
4.008	-0.00129	7.59E-04	4.008	-0.00169	9.51E-04	4.008	-0.00206	0.00112	4.008	-0.00241	0.00128	4.008	-0.00274	0.00142
4.009	-0.00129	7.58E-04	4.009	-0.00169	9.50E-04	4.009	-0.00206	0.00112	4.009	-0.00241	0.00128	4.009	-0.00274	0.00142
4.01	-0.00129	7.58E-04	4.01	-0.00169	9.50E-04	4.01	-0.00206	0.00112	4.01	-0.00241	0.00128	4.01	-0.00274	0.00142
4.011	-0.00129	7.57E-04	4.011	-0.00169	9.49E-04	4.011	-0.00206	0.00112	4.011	-0.00241	0.00128	4.011	-0.00274	0.00142
4.012	-0.00129	7.57E-04	4.012	-0.00168	9.48E-04	4.012	-0.00206	0.00112	4.012	-0.00241	0.00127	4.012	-0.00273	0.00142
4.013	-0.00128	7.56E-04	4.013	-0.00168	9.48E-04	4.013	-0.00206	0.00112	4.013	-0.0024	0.00127	4.013	-0.00273	0.00141
4.014	-0.00128	7.56E-04	4.014	-0.00168	9.47E-04	4.014	-0.00205	0.00112	4.014	-0.0024	0.00127	4.014	-0.00273	0.00141
4.015	-0.00128	7.55E-04	4.015	-0.00168	9.46E-04	4.015	-0.00205	0.00112	4.015	-0.0024	0.00127	4.015	-0.00273	0.00141
4.016	-0.00128	7.55E-04	4.016	-0.00168	9.46E-04	4.016	-0.00205	0.00112	4.016	-0.0024	0.00127	4.016	-0.00273	0.00141
4.017	-0.00128	7.54E-04	4.017	-0.00168	9.45E-04	4.017	-0.00205	0.00112	4.017	-0.0024	0.00127	4.017	-0.00272	0.00141
4.018	-0.00128	7.54E-04	4.018	-0.00168	9.44E-04	4.018	-0.00205	0.00111	4.018	-0.0024	0.00127	4.018	-0.00272	0.00141
4.019	-0.00128	7.53E-04	4.019	-0.00168	9.44E-04	4.019	-0.00205	0.00111	4.019	-0.00239	0.00127	4.019	-0.00272	0.00141
4.02	-0.00128	7.53E-04	4.02	-0.00167	9.43E-04	4.02	-0.00205	0.00111	4.02	-0.00239	0.00127	4.02	-0.00272	0.00141
4.021	-0.00128	7.52E-04	4.021	-0.00167	9.42E-04	4.021	-0.00204	0.00111	4.021	-0.00239	0.00127	4.021	-0.00272	0.00141
4.022	-0.00128	7.52E-04	4.022	-0.00167	9.42E-04	4.022	-0.00204	0.00111	4.022	-0.00239	0.00127	4.022	-0.00271	0.00141
4.023	-0.00128	7.51E-04	4.023	-0.00167	9.41E-04	4.023	-0.00204	0.00111	4.023	-0.00239	0.00126	4.023	-0.00271	0.0014
4.024	-0.00127	7.51E-04	4.024	-0.00167	9.40E-04	4.024	-0.00204	0.00111	4.024	-0.00239	0.00126	4.024	-0.00271	0.0014
4.025	-0.00127	7.50E-04	4.025	-0.00167	9.40E-04	4.025	-0.00204	0.00111	4.025	-0.00238	0.00126	4.025	-0.00271	0.0014
4.026	-0.00127	7.50E-04	4.026	-0.00167	9.39E-04	4.026	-0.00204	0.00111	4.026	-0.00238	0.00126	4.026	-0.00271	0.0014
4.027	-0.00127	7.49E-04	4.027	-0.00167	9.39E-04	4.027	-0.00204	0.00111	4.027	-0.00238	0.00126	4.027	-0.0027	0.0014
4.028	-0.00127	7.49E-04	4.028	-0.00167	9.38E-04	4.028	-0.00203	0.00111	4.028	-0.00238	0.00126	4.028	-0.0027	0.0014
4.029	-0.00127	7.48E-04	4.029	-0.00166	9.37E-04	4.029	-0.00203	0.00111	4.029	-0.00238	0.00126	4.029	-0.0027	0.0014
4.03	-0.00127	7.48E-04	4.03	-0.00166	9.37E-04	4.03	-0.00203	0.00111	4.03	-0.00238	0.00126	4.03	-0.0027	0.0014
4.031	-0.00127	7.47E-04	4.031	-0.00166	9.36E-04	4.031	-0.00203	0.0011	4.031	-0.00237	0.00126	4.031	-0.0027	0.0014
4.032	-0.00127	7.47E-04	4.032	-0.00166	9.35E-04	4.032	-0.00203	0.0011	4.032	-0.00237	0.00126	4.032	-0.00269	0.00139
4.033	-0.00127	7.46E-04	4.033	-0.00166	9.35E-04	4.033	-0.00203	0.0011	4.033	-0.00237	0.00126	4.033	-0.00269	0.00139
4.034	-0.00127	7.46E-04	4.034	-0.00166	9.34E-04	4.034	-0.00203	0.0011	4.034	-0.00237	0.00125	4.034	-0.00269	0.00139
4.035	-0.00127	7.45E-04	4.035	-0.00166	9.33E-04	4.035	-0.00202	0.0011	4.035	-0.00237	0.00125	4.035	-0.00269	0.00139
4.036	-0.00126	7.45E-04	4.036	-0.00166	9.33E-04	4.036	-0.00202	0.0011	4.036	-0.00237	0.00125	4.036	-0.00269	0.00139
4.037	-0.00126	7.44E-04	4.037	-0.00166	9.32E-04	4.037	-0.00202	0.0011	4.037	-0.00236	0.00125	4.037	-0.00268	0.00139
4.038	-0.00126	7.44E-04	4.038	-0.00165	9.31E-04	4.038	-0.00202	0.0011	4.038	-0.00236	0.00125	4.038	-0.00268	0.00139
4.039	-0.00126	7.43E-04	4.039	-0.00165	9.31E-04	4.039	-0.00202	0.0011	4.039	-0.00236	0.00125	4.039	-0.00268	0.00139
4.04	-0.00126	7.43E-04	4.04	-0.00165	9.30E-04	4.04	-0.00202	0.0011	4.04	-0.00236	0.00125	4.04	-0.00268	0.00139
4.041	-0.00126	7.42E-04	4.041	-0.00165	9.30E-04	4.041	-0.00202	0.0011	4.041	-0.00236	0.00125	4.041	-0.00268	0.00139
4.042	-0.00126	7.42E-04	4.042	-0.00165	9.29E-04	4.042	-0.00201	0.0011	4.042	-0.00236	0.00125	4.042	-0.00267	0.00138
4.043	-0.00126	7.41E-04	4.043	-0.00165	9.28E-04	4.043	-0.00201	0.0011	4.043	-0.00235	0.00125	4.043	-0.00267	0.00138
4.044	-0.00126	7.41E-04	4.044	-0.00165	9.28E-04	4.044	-0.00201	0.00109	4.044	-0.00235	0.00125	4.044	-0.00267	0.00138
4.045	-0.00126	7.40E-04	4.045	-0.00165	9.27E-04	4.045	-0.00201	0.00109	4.045	-0.00235	0.00124	4.045	-0.00267	0.00138
4.046	-0.00126	7.40E-04	4.046	-0.00165	9.26E-04	4.046	-0.00201	0.00109	4.046	-0.00235	0.00124	4.046	-0.00267	0.00138
4.047	-0.00126	7.39E-04	4.047	-0.00164	9.26E-04	4.047	-0.00201	0.00109	4.047	-0.00235	0.00124	4.047	-0.00266	0.00138
4.048	-0.00125	7.39E-04	4.048	-0.00164	9.25E-04	4.048	-0.00201	0.00109	4.048	-0.00235	0.00124	4.048	-0.00266	0.00138
4.049	-0.00125	7.38E-04	4.049	-0.00164	9.24E-04	4.049	-0.002	0.00109	4.049	-0.00234	0.00124	4.049	-0.00266	0.00138
4.05	-0.00125	7.38E-04	4.05	-0.00164	9.24E-04	4.05	-0.002	0.00109	4.05	-0.00234	0.00124	4.05	-0.00266	0.00138
4.051	-0.00125	7.37E-04	4.051	-0.00164	9.23E-04	4.051	-0.002	0.00109	4.051	-0.00234	0.00124	4.051	-0.00266	0.00138
4.052	-0.00125	7.37E-04	4.052	-0.00164	9.23E-04	4.052	-0.002	0.00109	4.052	-0.00234	0.00124	4.052	-0.00265	0.00137
4.053	-0.00125	7.36E-04	4.053	-0.00164	9.22E-04	4.053	-0.002	0.00109	4.053	-0.00234	0.00124	4.053	-0.00265	0.00137
4.054	-0.00125	7.36E-04	4.054	-0.00164	9.21E-04	4.054	-0.002	0.00109	4.054	-0.00234	0.00124	4.054	-0.00265	0.00137
4.055	-0.00125	7.35E-04	4.055	-0.00164	9.21E-04	4.055	-0.002	0.00109	4.055	-0.00233	0.00124	4.055	-0.00265	0.00137
4.056	-0.00125	7.35E-04	4.056	-0.00163	9.20E-04	4.056	-0.00199	0.00109	4.056	-0.00233	0.00123	4.056	-0.00265	0.00137
4.057	-0.00125	7.34E-04	4.057	-0.00163	9.19E-04	4.057	-0.00199	0.00108	4.057	-0.00233	0.00123	4.057	-0.00265	0.00137
4.058	-0.00125	7.34E-04	4.058	-0.00163	9.19E-04	4.058	-0.00199	0.00108	4.058	-0.00233	0.00123	4.058	-0.00264	0.00137
4.059	-0.00125	7.33E-04	4.059	-0.00163	9.18E-04	4.059	-0.00199	0.00108	4.059	-0.00233	0.00123	4.059	-0.00264	0.00137
4.06	-0.00124	7.33E-04	4.06	-0.00163	9.18E-04	4.06	-0.00199	0.00108	4.06	-0.00233	0.00123	4.06	-0.00264	0.00137
4.061	-0.00124	7.32E-04	4.061	-0.00163	9.17E-04	4.061	-0.00199	0.00108	4.061	-0.00232	0.00123	4.061	-0.00264	0.00137
4.062	-0.00124	7.32E-04	4.062	-0.00163	9.16E-04	4.062	-0.00199	0.00108	4.062	-0.00232	0.00123	4.062	-0.00264	0.00136
4.063	-0.00124	7.31E-04	4.063	-0.00163	9.16E-04	4.063	-0.00199	0.00108	4.063	-0.00232	0.00123	4.063	-0.00263	0.00136
4.064	-0.00124	7.31E-04	4.064	-0.00163	9.15E-04	4.064	-0.00198	0.00108	4.064	-0.00232	0.00123	4.064	-0.00263	0.00136
4.065	-0.00124	7.30E-04	4.065	-0.00162	9.14E-04	4.065	-0.00198	0.00108	4.065	-0.00232	0.00123	4.065	-0.00263	0.00136
4.066	-0.00124	7.30E-04	4.066	-0.00162	9.14E-04	4.066	-0.00198	0.00108	4.066	-0.00232	0.00123	4.066	-0.00263	0.00136
4.067	-0.00124	7.29E-04	4.067	-0.00162	9.13E-04	4.067	-0.00198	0.00108	4.067	-0.00231	0.00123	4.067	-0.00263	0.00136
4.068	-0.00124	7.29E-04	4.068	-0.00162	9.13E-04	4.068	-0.00198	0.00108	4.068	-0.00231	0.00122	4.068	-0.00262	0.00136
4.069	-0.00124	7.28E-04	4.069	-0.00162	9.12E-04	4.069	-0.00198	0.00108	4.069	-0.00231	0.00122	4.069	-0.00262	0.00136
4.07	-0.00124	7.28E-04	4.07	-0.00162	9.11E-04	4.07	-0.00198	0.00107	4.07	-0.00231	0.00122	4.07	-0.00262	0.00136
4.071	-0.00124	7.27E-04	4.071	-0.00162	9.11E-04	4.071	-0.00197	0.00107	4.071	-0.00231	0.00122	4.071	-0.00262	0.00136
4.072	-0.00123	7.27E-04	4.072	-0.00162	9.10E-04	4.072	-0.00197	0.00107	4.072	-0.00231	0.00122	4.072	-0.00262	0.00136
4.073	-0.00123	7.26E-04	4.073	-0.00162	9.09E-04	4.073	-0.00197	0.00107	4.073	-0.0023	0.00122	4.073	-0.00262	0.00135
4.074	-0.00123	7.26E-04	4.074	-0.00161	9.09E-04	4.074	-0.00197	0.00107	4.074	-0.0023	0.00122	4.074	-0.00261	0.00135
4.075	-0.00123	7.25E-04	4.075	-0.00161	9.08E-04	4.075	-0.00197	0.00107	4.075	-0.0023	0.00122	4.075	-0.00261	0.00135
4.076	-0.00123	7.25E-04	4.076	-0.00161	9.08E-04	4.076	-0.00197	0.00107	4.076	-0.0023	0.00122	4.076	-0.00261	0.00135
4.077	-0.00123	7.25E-04	4.077	-0.00161	9.07E-04	4.077	-0.00197	0.00107	4.077	-0.0023	0.00122	4.077	-0.00261	0.00135
4.078	-0.00123	7.24E-04	4.078	-0.00161	9.06E-04	4.078	-0.00196	0.00107	4.078	-0.0023	0.00122	4.078	-0.00261	0.00135
4.079	-0.00123	7.24E-04	4.079	-0.00161	9.06E-04	4.079	-0.00196	0.00107	4.079	-0.00229	0.00121	4.079	-0.0026	0.00135
4.08	-0.00123	7.23E-04	4.08	-0.00161	9.05E-04	4.08	-0.00196	0.00107	4.08	-0.00229	0.00121	4.08	-0.0026	0.00135
4.081	-0.00123	7.23E-04	4.081	-0.00161	9.04E-04	4.081	-0.00196	0.00107	4.081	-0.00229	0.00121	4.081	-0.0026	0.00135
4.082	-0.00123	7.22E-04	4.082	-0.00161	9.04E-04	4.082	-0.00196	0.00107	4.082	-0.00229	0.00121	4.082	-0.0026	0.00135
4.083	-0.00123	7.22E-04	4.083	-0.0016	9.03E-04	4.083	-0.00196	0.00107	4.083	-0.00229	0.00121	4.083	-0.0026	0.00134
4.084	-0.00122	7.21E-04	4.084	-0.0016	9.03E-04	4.084	-0.00196	0.00106	4.084	-0.00229	0.00121	4.084	-0.00259	0.00134
4.085	-0.00122	7.21E-04	4.085	-0.0016	9.02E-04	4.085	-0.00196	0.00106	4.085	-0.00228	0.00121	4.085	-0.00259	0.00134
4.086	-0.00122	7.20E-04	4.086	-0.0016	9.01E-04	4.086	-0.00195	0.00106	4.086	-0.00228	0.00121	4.086	-0.00259	0.00134
4.087	-0.00122	7.20E-04	4.087	-0.0016	9.01E-04	4.087	-0.00195	0.00106	4.087	-0.00228	0.00121	4.087	-0.00259	0.00134
4.088	-0.00122	7.19E-04	4.088	-0.0016	9.00E-04	4.088	-0.00195	0.00106	4.088	-0.00228	0.00121	4.088	-0.00259	0.00134
4.089	-0.00122	7.19E-04	4.089	-0.0016	9.00E-04	4.089	-0.00195	0.00106	4.089	-0.00228	0.00121	4.089	-0.00259	0.00134
4.09	-0.00122	7.18E-04	4.09	-0.0016	8.99E-04	4.09	-0.00195	0.00106	4.09	-0.00228	0.00121	4.09	-0.00258	0.00134
4.091	-0.00122	7.18E-04	4.091	-0.0016	8.98E-04	4.091	-0.00195	0.00106	4.091	-0.00227	0.0012	4.091	-0.00258	0.00134
4.092	-0.00122	7.17E-04	4.092	-0.00159	8.98E-04	4.092	-0.00195	0.00106	4.092	-0.00227	0.0012	4.092	-0.00258	0.00134
4.093	-0.00122	7.17E-04	4.093	-0.00159	8.97E-04	4.093	-0.00194	0.00106	4.093	-0.00227	0.0012	4.093	-0.00258	0.00133
4.094	-0.00122	7.16E-04	4.094	-0.00159	8.97E-04	4.094	-0.00194	0.00106	4.094	-0.00227	0.0012	4.094	-0.00258	0.00133
4.095	-0.00122	7.16E-04	4.095	-0.00159	8.96E-04	4.095	-0.00194	0.00106	4.095	-0.00227	0.0012	4.095	-0.00257	0.00133
4.096	-0.00122	7.15E-04	4.096	-0.00159	8.95E-04	4.096	-0.00194	0.00106	4.096	-0.00227	0.0012	4.096	-0.00257	0.00133
4.097	-0.00121	7.15E-04	4.097	-0.00159	8.95E-04	4.097	-0.00194	0.00105	4.097	-0.00227	0.0012	4.097	-0.00257	0.00133
4.098	-0.00121	7.15E-04	4.098	-0.00159	8.94E-04	4.098	-0.00194	0.00105	4.098	-0.00226	0.0012	4.098	-0.00257	0.00133
4.099	-0.00121	7.14E-04	4.099	-0.00159	8.93E-04	4.099	-0.00194	0.00105	4.099	-0.00226	0.0012	4.099	-0.00257	0.00133
4.1	-0.00121	7.14E-04	4.1	-0.00159	8.93E-04	4.1	-0.00193	0.00105	4.1	-0.00226	0.0012	4.1	-0.00256	0.00133
4.101	-0.00121	7.13E-04	4.101	-0.00158	8.92E-04	4.101	-0.00193	0.00105	4.101	-0.00226	0.0012	4.101	-0.00256	0.00133
4.102	-0.00121	7.13E-04	4.102	-0.00158	8.92E-04	4.102	-0.00193	0.00105	4.102	-0.00226	0.0012	4.102	-0.00256	0.00133
4.103	-0.00121	7.12E-04	4.103	-0.00158	8.91E-04	4.103	-0.00193	0.00105	4.103	-0.00226	0.00119	4.103	-0.00256	0.00133
4.104	-0.00121	7.12E-04	4.104	-0.00158	8.90E-04	4.104	-0.00193	0.00105	4.104	-0.00225	0.00119	4.104	-0.00256	0.00132
4.105	-0.00121	7.11E-04	4.105	-0.00158	8.90E-04	4.105	-0.00193	0.00105	4.105	-0.00225	0.00119	4.105	-0.00256	0.00132
4.106	-0.00121	7.11E-04	4.106	-0.00158	8.89E-04	4.106	-0.00193	0.00105	4.106	-0.00225	0.00119	4.106	-0.00255	0.00132
4.107	-0.00121	7.10E-04	4.107	-0.00158	8.89E-04	4.107	-0.00193	0.00105	4.107	-0.00225	0.00119	4.107	-0.00255	0.00132

4.108	-0.00121	7.10E-04	4.108	-0.00158	8.88E-04	4.108	-0.00192	0.00105	4.108	-0.00225	0.00119	4.108	-0.00255	0.00132
4.109	-0.0012	7.09E-04	4.109	-0.00158	8.87E-04	4.109	-0.00192	0.00105	4.109	-0.00225	0.00119	4.109	-0.00255	0.00132
4.11	-0.0012	7.09E-04	4.11	-0.00158	8.87E-04	4.11	-0.00192	0.00105	4.11	-0.00224	0.00119	4.11	-0.00255	0.00132
4.111	-0.0012	7.08E-04	4.111	-0.00157	8.86E-04	4.111	-0.00192	0.00104	4.111	-0.00224	0.00119	4.111	-0.00254	0.00132
4.112	-0.0012	7.08E-04	4.112	-0.00157	8.86E-04	4.112	-0.00192	0.00104	4.112	-0.00224	0.00119	4.112	-0.00254	0.00132
4.113	-0.0012	7.08E-04	4.113	-0.00157	8.85E-04	4.113	-0.00192	0.00104	4.113	-0.00224	0.00119	4.113	-0.00254	0.00132
4.114	-0.0012	7.07E-04	4.114	-0.00157	8.84E-04	4.114	-0.00192	0.00104	4.114	-0.00224	0.00119	4.114	-0.00254	0.00131
4.115	-0.0012	7.07E-04	4.115	-0.00157	8.84E-04	4.115	-0.00191	0.00104	4.115	-0.00224	0.00118	4.115	-0.00254	0.00131
4.116	-0.0012	7.06E-04	4.116	-0.00157	8.83E-04	4.116	-0.00191	0.00104	4.116	-0.00224	0.00118	4.116	-0.00254	0.00131
4.117	-0.0012	7.06E-04	4.117	-0.00157	8.83E-04	4.117	-0.00191	0.00104	4.117	-0.00223	0.00118	4.117	-0.00253	0.00131
4.118	-0.0012	7.05E-04	4.118	-0.00157	8.82E-04	4.118	-0.00191	0.00104	4.118	-0.00223	0.00118	4.118	-0.00253	0.00131
4.119	-0.0012	7.05E-04	4.119	-0.00157	8.81E-04	4.119	-0.00191	0.00104	4.119	-0.00223	0.00118	4.119	-0.00253	0.00131
4.12	-0.0012	7.04E-04	4.12	-0.00156	8.81E-04	4.12	-0.00191	0.00104	4.12	-0.00223	0.00118	4.12	-0.00253	0.00131
4.121	-0.0012	7.04E-04	4.121	-0.00156	8.80E-04	4.121	-0.00191	0.00104	4.121	-0.00223	0.00118	4.121	-0.00253	0.00131
4.122	-0.00119	7.03E-04	4.122	-0.00156	8.80E-04	4.122	-0.00191	0.00104	4.122	-0.00223	0.00118	4.122	-0.00252	0.00131
4.123	-0.00119	7.03E-04	4.123	-0.00156	8.79E-04	4.123	-0.0019	0.00104	4.123	-0.00222	0.00118	4.123	-0.00252	0.00131
4.124	-0.00119	7.02E-04	4.124	-0.00156	8.79E-04	4.124	-0.0019	0.00104	4.124	-0.00222	0.00118	4.124	-0.00252	0.00131
4.125	-0.00119	7.02E-04	4.125	-0.00156	8.78E-04	4.125	-0.0019	0.00103	4.125	-0.00222	0.00118	4.125	-0.00252	0.0013
4.126	-0.00119	7.02E-04	4.126	-0.00156	8.77E-04	4.126	-0.0019	0.00103	4.126	-0.00222	0.00118	4.126	-0.00252	0.0013
4.127	-0.00119	7.01E-04	4.127	-0.00156	8.77E-04	4.127	-0.0019	0.00103	4.127	-0.00222	0.00117	4.127	-0.00252	0.0013
4.128	-0.00119	7.01E-04	4.128	-0.00156	8.76E-04	4.128	-0.0019	0.00103	4.128	-0.00222	0.00117	4.128	-0.00251	0.0013
4.129	-0.00119	7.00E-04	4.129	-0.00156	8.76E-04	4.129	-0.0019	0.00103	4.129	-0.00221	0.00117	4.129	-0.00251	0.0013
4.13	-0.00119	7.00E-04	4.13	-0.00155	8.75E-04	4.13	-0.0019	0.00103	4.13	-0.00221	0.00117	4.13	-0.00251	0.0013
4.131	-0.00119	6.99E-04	4.131	-0.00155	8.74E-04	4.131	-0.00189	0.00103	4.131	-0.00221	0.00117	4.131	-0.00251	0.0013
4.132	-0.00119	6.99E-04	4.132	-0.00155	8.74E-04	4.132	-0.00189	0.00103	4.132	-0.00221	0.00117	4.132	-0.00251	0.0013
4.133	-6.98E-04	6.98E-04	4.133	-0.00155	8.73E-04	4.133	-0.00189	0.00103	4.133	-0.00221	0.00117	4.133	-0.00251	0.0013
4.134	-6.98E-04	6.98E-04	4.134	-0.00155	8.73E-04	4.134	-0.00189	0.00103	4.134	-0.00221	0.00117	4.134	-0.0025	0.0013
4.135	-6.98E-04	6.98E-04	4.135	-0.00155	8.72E-04	4.135	-0.00189	0.00103	4.135	-0.00221	0.00117	4.135	-0.0025	0.0013
4.136	-6.97E-04	6.97E-04	4.136	-0.00155	8.71E-04	4.136	-0.00189	0.00103	4.136	-0.0022	0.00117	4.136	-0.0025	0.00129
4.137	-6.97E-04	6.97E-04	4.137	-0.00155	8.71E-04	4.137	-0.00189	0.00103	4.137	-0.0022	0.00117	4.137	-0.0025	0.00129
4.138	-6.97E-04	6.97E-04	4.138	-0.00155	8.70E-04	4.138	-0.00188	0.00103	4.138	-0.0022	0.00117	4.138	-0.0025	0.00129
4.139	-6.96E-04	6.96E-04	4.139	-0.00154	8.70E-04	4.139	-0.00188	0.00102	4.139	-0.0022	0.00116	4.139	-0.00249	0.00129
4.14	-6.96E-04	6.96E-04	4.14	-0.00154	8.69E-04	4.14	-0.00188	0.00102	4.14	-0.0022	0.00116	4.14	-0.00249	0.00129
4.141	-6.96E-04	6.96E-04	4.141	-0.00154	8.69E-04	4.141	-0.00188	0.00102	4.141	-0.0022	0.00116	4.141	-0.00249	0.00129
4.142	-6.95E-04	6.95E-04	4.142	-0.00154	8.68E-04	4.142	-0.00188	0.00102	4.142	-0.00219	0.00116	4.142	-0.00249	0.00129
4.143	-6.95E-04	6.95E-04	4.143	-0.00154	8.67E-04	4.143	-0.00188	0.00102	4.143	-0.00219	0.00116	4.143	-0.00249	0.00129
4.144	-6.95E-04	6.95E-04	4.144	-0.00154	8.67E-04	4.144	-0.00188	0.00102	4.144	-0.00219	0.00116	4.144	-0.00249	0.00129
4.145	-6.94E-04	6.94E-04	4.145	-0.00154	8.66E-04	4.145	-0.00188	0.00102	4.145	-0.00219	0.00116	4.145	-0.00248	0.00129
4.146	-6.94E-04	6.94E-04	4.146	-0.00154	8.66E-04	4.146	-0.00187	0.00102	4.146	-0.00219	0.00116	4.146	-0.00248	0.00129
4.147	-6.94E-04	6.94E-04	4.147	-0.00154	8.65E-04	4.147	-0.00187	0.00102	4.147	-0.00219	0.00116	4.147	-0.00248	0.00128
4.148	-6.93E-04	6.93E-04	4.148	-0.00154	8.64E-04	4.148	-0.00187	0.00102	4.148	-0.00219	0.00116	4.148	-0.00248	0.00128
4.149	-6.93E-04	6.93E-04	4.149	-0.00153	8.64E-04	4.149	-0.00187	0.00102	4.149	-0.00218	0.00116	4.149	-0.00248	0.00128
4.15	-6.93E-04	6.93E-04	4.15	-0.00153	8.63E-04	4.15	-0.00187	0.00102	4.15	-0.00218	0.00116	4.15	-0.00248	0.00128
4.151	-6.92E-04	6.92E-04	4.151	-0.00153	8.63E-04	4.151	-0.00187	0.00102	4.151	-0.00218	0.00115	4.151	-0.00247	0.00128
4.152	-6.92E-04	6.92E-04	4.152	-0.00153	8.62E-04	4.152	-0.00187	0.00102	4.152	-0.00218	0.00115	4.152	-0.00247	0.00128
4.153	-6.92E-04	6.92E-04	4.153	-0.00153	8.62E-04	4.153	-0.00187	0.00101	4.153	-0.00218	0.00115	4.153	-0.00247	0.00128
4.154	-6.91E-04	6.91E-04	4.154	-0.00153	8.61E-04	4.154	-0.00186	0.00101	4.154	-0.00218	0.00115	4.154	-0.00247	0.00128
4.155	-6.91E-04	6.91E-04	4.155	-0.00153	8.60E-04	4.155	-0.00186	0.00101	4.155	-0.00218	0.00115	4.155	-0.00247	0.00128
4.156	-6.91E-04	6.91E-04	4.156	-0.00153	8.60E-04	4.156	-0.00186	0.00101	4.156	-0.00217	0.00115	4.156	-0.00246	0.00128
4.157	-6.90E-04	6.90E-04	4.157	-0.00153	8.59E-04	4.157	-0.00186	0.00101	4.157	-0.00217	0.00115	4.157	-0.00246	0.00128
4.158	-6.90E-04	6.90E-04	4.158	-0.00153	8.59E-04	4.158	-0.00186	0.00101	4.158	-0.00217	0.00115	4.158	-0.00246	0.00127
4.159	-6.90E-04	6.90E-04	4.159	-0.00152	8.58E-04	4.159	-0.00186	0.00101	4.159	-0.00217	0.00115	4.159	-0.00246	0.00127
4.16	-6.89E-04	6.89E-04	4.16	-0.00152	8.58E-04	4.16	-0.00186	0.00101	4.16	-0.00217	0.00115	4.16	-0.00246	0.00127
4.161	-6.89E-04	6.89E-04	4.161	-0.00152	8.57E-04	4.161	-0.00186	0.00101	4.161	-0.00217	0.00115	4.161	-0.00246	0.00127
4.162	-6.89E-04	6.89E-04	4.162	-0.00152	8.56E-04	4.162	-0.00185	0.00101	4.162	-0.00216	0.00115	4.162	-0.00245	0.00127
4.163	-6.88E-04	6.88E-04	4.163	-0.00152	8.56E-04	4.163	-0.00185	0.00101	4.163	-0.00216	0.00115	4.163	-0.00245	0.00127
4.164	-6.88E-04	6.88E-04	4.164	-0.00152	8.55E-04	4.164	-0.00185	0.00101	4.164	-0.00216	0.00114	4.164	-0.00245	0.00127
4.165	-6.88E-04	6.88E-04	4.165	-0.00152	8.55E-04	4.165	-0.00185	0.00101	4.165	-0.00216	0.00114	4.165	-0.00245	0.00127
4.166	-6.87E-04	6.87E-04	4.166	-0.00152	8.54E-04	4.166	-0.00185	0.00101	4.166	-0.00216	0.00114	4.166	-0.00245	0.00127
4.167	-6.87E-04	6.87E-04	4.167	-0.00152	8.54E-04	4.167	-0.00185	0.00101	4.167	-0.00216	0.00114	4.167	-0.00245	0.00127
4.168	-6.87E-04	6.87E-04	4.168	-0.00151	8.53E-04	4.168	-0.00185	0.001	4.168	-0.00216	0.00114	4.168	-0.00244	0.00127
4.169	-6.86E-04	6.86E-04	4.169	-0.00151	8.52E-04	4.169	-0.00185	0.001	4.169	-0.00215	0.00114	4.169	-0.00244	0.00126
4.17	-6.86E-04	6.86E-04	4.17	-0.00151	8.52E-04	4.17	-0.00184	0.001	4.17	-0.00215	0.00114	4.17	-0.00244	0.00126
4.171	-6.86E-04	6.86E-04	4.171	-0.00151	8.51E-04	4.171	-0.00184	0.001	4.171	-0.00215	0.00114	4.171	-0.00244	0.00126
4.172	-6.85E-04	6.85E-04	4.172	-0.00151	8.51E-04	4.172	-0.00184	0.001	4.172	-0.00215	0.00114	4.172	-0.00244	0.00126
4.173	-6.85E-04	6.85E-04	4.173	-0.00151	8.50E-04	4.173	-0.00184	0.001	4.173	-0.00215	0.00114	4.173	-0.00244	0.00126
4.174	-6.85E-04	6.85E-04	4.174	-0.00151	8.50E-04	4.174	-0.00184	0.001	4.174	-0.00215	0.00114	4.174	-0.00243	0.00126
4.175	-6.84E-04	6.84E-04	4.175	-0.00151	8.49E-04	4.175	-0.00184	1.00E-03	4.175	-0.00215	0.00114	4.175	-0.00243	0.00126
4.176	-6.84E-04	6.84E-04	4.176	-0.00151	8.48E-04	4.176	-0.00184	9.99E-04	4.176	-0.00214	0.00113	4.176	-0.00243	0.00126
4.177	-6.84E-04	6.84E-04	4.177	-0.00151	8.48E-04	4.177	-0.00184	9.98E-04	4.177	-0.00214	0.00113	4.177	-0.00243	0.00126
4.178	-6.83E-04	6.83E-04	4.178	-0.0015	8.47E-04	4.178	-0.00183	9.98E-04	4.178	-0.00214	0.00113	4.178	-0.00243	0.00126
4.179	-6.83E-04	6.83E-04	4.179	-0.0015	8.47E-04	4.179	-0.00183	9.97E-04	4.179	-0.00214	0.00113	4.179	-0.00243	0.00126
4.18	-6.83E-04	6.83E-04	4.18	-0.0015	8.46E-04	4.18	-0.00183	9.96E-04	4.18	-0.00214	0.00113	4.18	-0.00242	0.00125
4.181	-6.82E-04	6.82E-04	4.181	-0.0015	8.46E-04	4.181	-0.00183	9.96E-04	4.181	-0.00214	0.00113	4.181	-0.00242	0.00125
4.182	-6.82E-04	6.82E-04	4.182	-0.0015	8.45E-04	4.182	-0.00183	9.95E-04	4.182	-0.00213	0.00113	4.182	-0.00242	0.00125
4.183	-6.82E-04	6.82E-04	4.183	-0.0015	8.45E-04	4.183	-0.00183	9.94E-04	4.183	-0.00213	0.00113	4.183	-0.00242	0.00125
4.184	-6.81E-04	6.81E-04	4.184	-0.0015	8.44E-04	4.184	-0.00183	9.94E-04	4.184	-0.00213	0.00113	4.184	-0.00242	0.00125
4.185	-6.81E-04	6.81E-04	4.185	-0.0015	8.43E-04	4.185	-0.00183	9.93E-04	4.185	-0.00213	0.00113	4.185	-0.00242	0.00125
4.186	-6.81E-04	6.81E-04	4.186	-0.0015	8.43E-04	4.186	-0.00182	9.92E-04	4.186	-0.00213	0.00113	4.186	-0.00241	0.00125
4.187	-6.80E-04	6.80E-04	4.187	-0.0015	8.42E-04	4.187	-0.00182	9.92E-04	4.187	-0.00213	0.00113	4.187	-0.00241	0.00125
4.188	-6.80E-04	6.80E-04	4.188	-0.00149	8.42E-04	4.188	-0.00182	9.91E-04	4.188	-0.00213	0.00113	4.188	-0.00241	0.00125
4.189	-6.80E-04	6.80E-04	4.189	-0.00149	8.41E-04	4.189	-0.00182	9.90E-04	4.189	-0.00212	0.00112	4.189	-0.00241	0.00125
4.19	-6.80E-04	6.80E-04	4.19	-0.00149	8.41E-04	4.19	-0.00182	9.90E-04	4.19	-0.00212	0.00112	4.19	-0.00241	0.00125
4.191	-6.79E-04	6.79E-04	4.191	-0.00149	8.40E-04	4.191	-0.00182	9.89E-04	4.191	-0.00212	0.00112	4.191	-0.00241	0.00125
4.192	-6.79E-04	6.79E-04	4.192	-0.00149	8.40E-04	4.192	-0.00182	9.88E-04	4.192	-0.00212	0.00112	4.192	-0.0024	0.00124
4.193	-6.79E-04	6.79E-04	4.193	-0.00149	8.39E-04	4.193	-0.00182	9.88E-04	4.193	-0.00212	0.00112	4.193	-0.0024	0.00124
4.194	-6.78E-04	6.78E-04	4.194	-0.00149	8.38E-04	4.194	-0.00181	9.87E-04	4.194	-0.00212	0.00112	4.194	-0.0024	0.00124
4.195	-6.78E-04	6.78E-04	4.195	-0.00149	8.38E-04	4.195	-0.00181	9.86E-04	4.195	-0.00212	0.00112	4.195	-0.0024	0.00124
4.196	-6.78E-04	6.78E-04	4.196	-0.00149	8.37E-04	4.196	-0.00181	9.86E-04	4.196	-0.00211	0.00112	4.196	-0.0024	0.00124
4.197	-6.77E-04	6.77E-04	4.197	-0.00149	8.37E-04	4.197	-0.00181	9.85E-04	4.197	-0.00211	0.00112	4.197	-0.00239	0.00124
4.198	-6.77E-04	6.77E-04	4.198	-0.00149	8.36E-04	4.198	-0.00181	9.84E-04	4.198	-0.00211	0.00112	4.198	-0.00239	0.00124
4.199	-6.77E-04	6.77E-04	4.199	-0.00148	8.36E-04	4.199	-0.00181	9.84E-04	4.199	-0.00211	0.00112	4.199	-0.00239	0.00124
4.2	-6.76E-04	6.76E-04	4.2	-0.00148	8.35E-04	4.2	-0.00181	9.83E-04	4.2	-0.00211	0.00112	4.2	-0.00239	0.00124
4.201	-6.76E-04	6.76E-04	4.201	-0.00148	8.35E-04	4.201	-0.00181	9.82E-04	4.201	-0.00211	0.00112	4.201	-0.00239	0.00124
4.202	-6.76E-04	6.76E-04	4.202	-0.00148	8.34E-04	4.202	-0.0018	9.82E-04	4.202	-0.00211	0.00111	4.202	-0.00239	0.00124
4.203	-6.75E-04	6.75E-04	4.203	-0.00148	8.33E-04	4.203	-0.0018	9.81E-04	4.203	-0.0021	0.00111	4.203	-0.00238	0.00123
4.204	-6.75E-04	6.75E-04	4.204	-0.00148	8.33E-04	4.204	-0.0018	9.80E-04	4.204	-0.0021	0.00111	4.204	-0.00238	0.00123
4.205	-6.75E-04	6.75E-04	4.205	-0.00148	8.32E-04	4.205	-0.0018	9.80E-04	4.205	-0.0021	0.00111	4.205	-0.00238	0.00123
4.206	-6.74E-04	6.74E-04	4.206	-0.00148	8.32E-04	4.206	-0.0018	9.79E-04	4.206	-0.0021	0.00111	4.206	-0.00238	0.00123
4.207	-6.74E-04	6.74E-04	4.207	-0.00148	8.31E-04	4.207	-0.0018	9.78E-04	4.207	-0.0021	0.00111	4.207	-0.00238	0.00123
4.208	-6.74E-04	6.74E-04	4.208	-0.00148	8.31E-04	4.208	-0.0018	9.78E-04	4.208	-0.0021	0.00111	4.208	-0.00238	0.00123
4.209	-6.73E-04	6.73E-04	4.209	-0.00147	8.30E-04	4.209	-0.0018	9.77E-04	4.209	-0.0021	0.00111	4.209	-0.00238	0.00123
4.21	-6.73E-04	6.73E-04	4.21	-0.00147	8.30E-04	4.21	-0.00179	9.76E-04	4.21	-0.00209	0.00111	4.21	-0.00237	0.00123
4.211	-6.73E-04	6.73E-04	4.211	-0.00147	8.29E-04	4.211	-0.00179	9.76E-04	4.211	-0.00209	0.00111	4.211	-0.00237	0.00123
4.212	-6.72E-04	6.72E-04	4.212	-0.00147	8.29E-04	4.212	-0.00179	9.75E-04	4.212	-0.00209	0.00111	4.212	-0.00237	0.00123
4.213	-6.72E-04	6.72E-04	4.213	-0.00147	8.28E-04	4.213	-0.00179	9.74E-04	4.213	-0.00209	0.00111	4.213	-0.00237	0.00123
4.214	-6.72E-04	6.72E-04	4.214	-0.00147	8.27E-04	4.214	-0.00179	9.74E-04	4.214	-0.00209	0.00111	4.214	-0.00237	0.00123
4.215	-6.71E-04	6.71E-04	4.215	-0.00147	8.27E-04	4.215	-0.00179	9.73E-04	4.215	-0.00209	0.0011	4.215	-0.00237	0.00122
4.216	-6.71E-04	6.71E-04	4.216	-0.00147	8.26E-04	4.216	-0.00179	9.72E-04	4.216	-0.00209	0.0011	4.216	-0.00236	0.00122
4.217	-6.71E-04	6.71E-04	4.217	-0.00147	8.26E-04	4.217	-0.00179	9.72E-04	4.217	-0.00208	0.0011	4.217	-0.00236	0.00122
4.218	-6.71E-04	6.71E-04	4.218	-0.00147	8.25E-04	4.218	-0.00179	9.71E-04	4.218	-0.00208	0.0011	4.218	-0.00236	0.00122
4.219	-6.70E-04	6.70E-04	4.219	-0.00146	8.25E-04	4.219	-0.00178	9.71E-04	4.219	-0.00208	0.0011	4.219	-0.00236	0.00122
4.22	-6.70E-04	6.70E-04	4.22	-0.00146	8.24E-04	4.22	-0.00178	9.70E-04	4.22	-0.00208	0.0011	4.22	-0.00236	0.00122
4.221	-6.70E-04	6.70E-04	4.221	-0.00146	8.24E-04	4.221	-0.00178	9.69E-04	4.221	-0.00208	0.0011	4.221	-0.00236	0.00122
4.222	-6.69E-04	6.69E-04	4.222	-0.00146	8.23E-04	4.222	-0.00178	9.69E-04	4.222	-0.00208	0.0011	4.222	-0.00235	0.00122
4.223	-6.69E-04	6.69E-04	4.223	-0.00146	8.23E-04	4.223	-0.00178	9.68E-04	4.223	-0.00208	0.0011	4.223	-0.00235	0.00122
4.224	-6.69E-04	6.69E-04	4.224	-0.00146	8.22E-04	4.224	-0.00178	9.67E-04	4.224	-0.00207	0.0011	4.224	-0.00235	0.00122
4.225	-6.68E-04	6.68E-04	4.225	-0.00146	8.21E-04	4.225	-0.00178	9.67E-04	4.225	-0.00207	0.0011	4.225	-0.00235	0.00122
4.226	-6.68E-04	6.68E-04	4.226	-0.00146	8.21E-04	4.226	-0.00178	9.66E-04	4.226	-0.00207	0.0011	4.226	-0.00235	0.00122
4.227	-6.68E-04	6.68E-04	4.227	-0.00146	8.20E-04	4.227	-0.00177	9.65E-04	4.227	-0.00207	0.0011	4.227	-0.00235	0.00121
4.228	-6.67E-04	6.67E-04	4.228	-0.00146	8.20E-04	4.228	-0.00177	9.65E-04	4.228	-0.00207	0.0011	4.228	-0.00234	0.00121
4.229	-6.67E-04	6.67E-04	4.229	-0.00146	8.19E-04	4.229	-0.00177	9.64E-04	4.229	-0.00207	0.00109	4.229	-0.00234	0.00121
4.23	-6.67E-04	6.67E-04	4.23	-0.00145	8.19E-04	4.23	-0.00177	9.63E-04	4.23	-0.00207	0.00109	4.23	-0.00234	0.00121
4.231	-6.66E-04	6.66E-04	4.231	-0.00145	8.18E-04	4.231	-0.00177	9.63E-04	4.231	-0.00206	0.00109	4.231	-0.00234	0.00121
4.232	-6.66E-04	6.66E-04	4.232	-0.00145	8.18E-04	4.232	-0.00177	9.62E-04	4.232	-0.00206	0.00109	4.232	-0.00234	0.00121
4.233	-6.66E-04	6.66E-04	4.233	-0.00145	8.17E-04	4.233	-0.00177	9.61E-04	4.233	-0.00206	0.00109	4.233	-0.00234	0.00121
4.234	-6.65E-04	6.65E-04	4.234	-0.00145	8.17E-04	4.234	-0.00177	9.61E-04	4.234	-0.00206	0.00109	4.234	-0.00233	0.00121
4.235	-6.65E-04	6.65E-04	4.235	-0.00145	8.16E-04	4.235	-0.00176	9.60E-04	4.235	-0.00206	0.00109	4.235	-0.00233	0.00121
4.236	-6.65E-04	6.65E-04	4.236	-0.00145	8.16E-04	4.236	-0.00176	9.60E-04	4.236	-0.00206	0.00109	4.236	-0.00233	0.00121
4.237	-6.65E-04	6.65E-04	4.237	-0.00145	8.15E-04	4.237	-0.00176	9.59E-04	4.237	-0.00206	0.00109	4.237	-0.00233	0.00121
4.238	-6.64E-04	6.64E-04	4.238	-0.00145	8.14E-04	4.238	-0.00176	9.58E-04	4.238	-0.00205	0.00109	4.238	-0.00233	0.00121
4.239	-6.64E-04	6.64E-04	4.239	-0.00145	8.14E-04	4.239	-0.00176	9.58E-04	4.239	-0.00205	0.00109	4.239	-0.00233	0.0012
4.24	-6.64E-04	6.64E-04	4.24	-0.00144	8.13E-04	4.24	-0.00176	9.57E-04	4.24	-0.00205	0.00109	4.24	-0.00232	0.0012
4.241	-6.63E-04	6.63E-04	4.241	-0.00144	8.13E-04	4.241	-0.00176	9.56E-04	4.241	-0.00205	0.00109	4.241	-0.00232	0.0012
4.242	-6.63E-04	6.63E-04	4.242	-0.00144	8.12E-04	4.242	-0.00176	9.56E-04	4.242	-0.00205	0.00108	4.242	-0.00232	0.0012
4.243	-6.63E-04	6.63E-04	4.243	-0.00144	8.12E-04	4.243	-0.00176	9.55E-04	4.243	-0.00205	0.00108	4.243	-0.00232	0.0012
4.244	-6.62E-04	6.62E-04	4.244	-0.00144	8.11E-04	4.244	-0.00175	9.54E-04	4.244	-0.00205	0.00108	4.244	-0.00232	0.0012
4.245	-6.62E-04	6.62E-04	4.245	-0.00144	8.11E-04	4.245	-0.00175	9.54E-04	4.245	-0.00204	0.00108	4.245	-0.00232	0.0012
4.246	-6.62E-04	6.62E-04	4.246	-0.00144	8.10E-04	4.246	-0.00175	9.53E-04	4.246	-0.00204	0.00108	4.246	-0.00231	0.0012
4.247	-6.61E-04	6.61E-04	4.247	-0.00144	8.10E-04	4.247	-0.00175	9.52E-04	4.247	-0.00204	0.00108	4.247	-0.00231	0.0012
4.248	-6.61E-04	6.61E-04	4.248	-0.00144	8.09E-04	4.248	-0.00175	9.52E-04	4.248	-0.00204	0.00108	4.248	-0.00231	0.0012
4.249	-6.61E-04	6.61E-04	4.249	-0.00144	8.09E-04	4.249	-0.00175	9.51E-04	4.249	-0.00204	0.00108	4.249	-0.00231	0.0012
4.25	-6.60E-04	6.60E-04	4.25	-0.00144	8.08E-04	4.25	-0.00175	9.51E-04	4.25	-0.00204	0.00108	4.25	-0.00231	0.0012
4.251	-6.60E-04	6.60E-04	4.251	-0.00143	8.08E-04	4.251	-0.00175	9.50E-04	4.251	-0.00204	0.00108	4.251	-0.00231	0.00119
4.252	-6.60E-04	6.60E-04	4.252	-0.00143	8.07E-04	4.252	-0.00174	9.49E-04	4.252	-0.00203	0.00108	4.252	-0.00231	0.00119
4.253	-6.60E-04	6.60E-04	4.253	-0.00143	8.07E-04	4.253	-0.00174	9.49E-04	4.253	-0.00203	0.00108	4.253	-0.0023	0.00119
4.254	-6.59E-04	6.59E-04	4.254	-0.00143	8.06E-04	4.254	-0.00174	9.48E-04	4.254	-0.00203	0.00108	4.254	-0.0023	0.00119
4.255	-6.59E-04	6.59E-04	4.255	-0.00143	8.05E-04	4.255	-0.00174	9.47E-04	4.255	-0.00203	0.00108	4.255	-0.0023	0.00119
4.256	-6.59E-04	6.59E-04	4.256	-0.00143	8.05E-04	4.256	-0.00174	9.47E-04	4.256	-0.00203	0.00107	4.256	-0.0023	0.00119
4.257	-6.58E-04	6.58E-04	4.257	-0.00143	8.04E-04	4.257	-0.00174	9.46E-04	4.257	-0.00203	0.00107	4.257	-0.0023	0.00119
4.258	-6.58E-04	6.58E-04	4.258	-0.00143	8.04E-04	4.258	-0.00174	9.46E-04	4.258	-0.00203	0.00107	4.258	-0.0023	0.00119
4.259	-6.58E-04	6.58E-04	4.259	-0.00143	8.03E-04	4.259	-0.00174	9.45E-04	4.259	-0.00203	0.00107	4.259	-0.00229	0.00119
4.26	-6.57E-04	6.57E-04	4.26	-0.00143	8.03E-04	4.26	-0.00174	9.44E-04	4.26	-0.00202	0.00107	4.26	-0.00229	0.00119
4.261	-6.57E-04	6.57E-04	4.261	-0.00142	8.02E-04	4.261	-0.00173	9.44E-04	4.261	-0.00202	0.00107	4.261	-0.00229	0.00119
4.262	-6.57E-04	6.57E-04	4.262	-0.00142	8.02E-04	4.262	-0.00173	9.43E-04	4.262	-0.00202	0.00107	4.262	-0.00229	0.00119
4.263	-6.56E-04	6.56E-04	4.263	-0.00142	8.01E-04	4.263	-0.00173	9.42E-04	4.263	-0.00202	0.00107	4.263	-0.00229	0.00118
4.264	-6.56E-04	6.56E-04	4.264	-0.00142	8.01E-04	4.264	-0.00173	9.42E-04	4.264	-0.00202	0.00107	4.264	-0.00229	0.00118
4.265	-6.56E-04	6.56E-04	4.265	-0.00142	8.00E-04	4.265	-0.00173	9.41E-04	4.265	-0.00202	0.00107	4.265	-0.00228	0.00118
4.266	-6.56E-04	6.56E-04	4.266	-0.00142	8.00E-04	4.266	-0.00173	9.40E-04	4.266	-0.00202	0.00107	4.266	-0.00228	0.00118
4.267	-6.55E-04	6.55E-04	4.267	-0.00142	7.99E-04	4.267	-0.00173	9.40E-04	4.267	-0.00201	0.00107	4.267	-0.00228	0.00118
4.268	-6.55E-04	6.55E-04	4.268	-0.00142	7.99E-04	4.268	-0.00173	9.39E-04	4.268	-0.00201	0.00107	4.268	-0.00228	0.00118
4.269	-6.55E-04	6.55E-04	4.269	-0.00142	7.98E-04	4.269	-0.00173	9.39E-04	4.269	-0.00201	0.00106	4.269	-0.00228	0.00118
4.27	-6.54E-04	6.54E-04	4.27	-0.00142	7.98E-04	4.27	-0.00172	9.38E-04	4.27	-0.00201	0.00106	4.27	-0.00228	0.00118
4.271	-6.54E-04	6.54E-04	4.271	-0.00142	7.97E-04	4.271	-0.00172	9.37E-04	4.271	-0.00201	0.00106	4.271	-0.00228	0.00118
4.272	-6.54E-04	6.54E-04	4.272	-0.00141	7.97E-04	4.272	-0.00172	9.37E-04	4.272	-0.00201	0.00106	4.272	-0.00227	0.00118
4.273	-6.53E-04	6.53E-04	4.273	-0.00141	7.96E-04	4.273	-0.00172	9.36E-04	4.273	-0.00201	0.00106	4.273	-0.00227	0.00118
4.274	-6.53E-04	6.53E-04	4.274	-0.00141	7.96E-04	4.274	-0.00172	9.36E-04	4.274	-0.002	0.00106	4.274	-0.00227	0.00118
4.275	-6.53E-04	6.53E-04	4.275	-0.00141	7.95E-04	4.275	-0.00172	9.35E-04	4.275	-0.002	0.00106	4.275	-0.00227	0.00117
4.276	-6.52E-04	6.52E-04	4.276	-0.00141	7.95E-04	4.276	-0.00172	9.34E-04	4.276	-0.002	0.00106	4.276	-0.00227	0.00117
4.277	-6.52E-04	6.52E-04	4.277	-0.00141	7.94E-04	4.277	-0.00172	9.34E-04	4.277	-0.002	0.00106	4.277	-0.00227	0.00117
4.278	-6.52E-04	6.52E-04	4.278	-0.00141	7.94E-04	4.278	-0.00171	9.33E-04	4.278	-0.002	0.00106	4.278	-0.00226	0.00117
4.279	-6.52E-04	6.52E-04	4.279	-0.00141	7.93E-04	4.279	-0.00171	9.32E-04	4.279	-0.002	0.00106	4.279	-0.00226	0.00117
4.28	-6.51E-04	6.51E-04	4.28	-0.00141	7.92E-04	4.28	-0.00171	9.32E-04	4.28	-0.002	0.00106	4.28	-0.00226	0.00117
4.281	-6.51E-04	6.51E-04	4.281	-0.00141	7.92E-04	4.281	-0.00171	9.31E-04	4.281	-0.002	0.00106	4.281	-0.00226	0.00117
4.282	-6.51E-04	6.51E-04	4.282	-0.00141	7.91E-04	4.282	-0.00171	9.31E-04	4.282	-0.00199	0.00106	4.282	-0.00226	0.00117
4.283	-6.50E-04	6.50E-04	4.283	-0.0014	7.91E-04	4.283	-0.00171	9.30E-04	4.283	-0.00199	0.00105	4.283	-0.00226	0.00117
4.284	-6.50E-04	6.50E-04	4.284	-0.0014	7.90E-04	4.284	-0.00171	9.29E-04	4.284	-0.00199	0.00105	4.284	-0.00226	0.00117
4.285	-6.50E-04	6.50E-04	4.285	-0.0014	7.90E-04	4.285	-0.00171	9.29E-04	4.285	-0.00199	0.00105	4.285	-0.00225	0.00117
4.286	-6.49E-04	6.49E-04	4.286	-0.0014	7.89E-04	4.286	-0.00171	9.28E-04	4.286	-0.00199	0.00105	4.286	-0.00225	0.00117
4.287	-6.49E-04	6.49E-04	4.287	-0.0014	7.89E-04	4.287	-0.0017	9.27E-04	4.287	-0.00199	0.00105	4.287	-0.00225	0.00117
4.288	-6.49E-04	6.49E-04	4.288	-0.0014	7.88E-04	4.288	-0.0017	9.27E-04	4.288	-0.00199	0.00105	4.288	-0.00225	0.00116
4.289	-6.49E-04	6.49E-04	4.289	-0.0014	7.88E-04	4.289	-0.0017	9.26E-04	4.289	-0.00198	0.00105	4.289	-0.00225	0.00116
4.29	-6.48E-04	6.48E-04	4.29	-0.0014	7.87E-04	4.29	-0.0017	9.26E-04	4.29	-0.00198	0.00105	4.29	-0.00225	0.00116
4.291	-6.48E-04	6.48E-04	4.291	-0.0014	7.87E-04	4.291	-0.0017	9.25E-04	4.291	-0.00198	0.00105	4.291	-0.00224	0.00116
4.292	-6.48E-04	6.48E-04	4.292	-0.0014	7.86E-04	4.292	-0.0017	9.24E-04	4.292	-0.00198	0.00105	4.292	-0.00224	0.00116

4.293	-6.47E-04	6.47E-04	4.293	-0.0014	7.86E-04	4.293	-0.0017	9.24E-04	4.293	-0.00198	0.00105	4.293	-0.00224	0.00116
4.294	-6.47E-04	6.47E-04	4.294	-0.00139	7.85E-04	4.294	-0.0017	9.23E-04	4.294	-0.00198	0.00105	4.294	-0.00224	0.00116
4.295	-6.47E-04	6.47E-04	4.295	-0.00139	7.85E-04	4.295	-0.0017	9.23E-04	4.295	-0.00198	0.00105	4.295	-0.00224	0.00116
4.296	-6.46E-04	6.46E-04	4.296	-0.00139	7.84E-04	4.296	-0.00169	9.22E-04	4.296	-0.00198	0.00105	4.296	-0.00224	0.00116
4.297	-6.46E-04	6.46E-04	4.297	-0.00139	7.84E-04	4.297	-0.00169	9.21E-04	4.297	-0.00197	0.00105	4.297	-0.00224	0.00116
4.298	-6.46E-04	6.46E-04	4.298	-0.00139	7.83E-04	4.298	-0.00169	9.21E-04	4.298	-0.00197	0.00104	4.298	-0.00223	0.00116
4.299	-6.45E-04	6.45E-04	4.299	-0.00139	7.83E-04	4.299	-0.00169	9.20E-04	4.299	-0.00197	0.00104	4.299	-0.00223	0.00116
4.3	-6.45E-04	6.45E-04	4.3	-0.00139	7.82E-04	4.3	-0.00169	9.20E-04	4.3	-0.00197	0.00104	4.3	-0.00223	0.00116
4.301	-6.45E-04	6.45E-04	4.301	-0.00139	7.82E-04	4.301	-0.00169	9.19E-04	4.301	-0.00197	0.00104	4.301	-0.00223	0.00115
4.302	-6.45E-04	6.45E-04	4.302	-0.00139	7.81E-04	4.302	-0.00169	9.18E-04	4.302	-0.00197	0.00104	4.302	-0.00223	0.00115
4.303	-6.44E-04	6.44E-04	4.303	-0.00139	7.81E-04	4.303	-0.00169	9.18E-04	4.303	-0.00197	0.00104	4.303	-0.00223	0.00115
4.304	-6.44E-04	6.44E-04	4.304	-0.00139	7.80E-04	4.304	-0.00169	9.17E-04	4.304	-0.00196	0.00104	4.304	-0.00222	0.00115
4.305	-6.44E-04	6.44E-04	4.305	-0.00138	7.80E-04	4.305	-0.00168	9.17E-04	4.305	-0.00196	0.00104	4.305	-0.00222	0.00115
4.306	-6.43E-04	6.43E-04	4.306	-0.00138	7.79E-04	4.306	-0.00168	9.16E-04	4.306	-0.00196	0.00104	4.306	-0.00222	0.00115
4.307	-6.43E-04	6.43E-04	4.307	-0.00138	7.79E-04	4.307	-0.00168	9.15E-04	4.307	-0.00196	0.00104	4.307	-0.00222	0.00115
4.308	-6.43E-04	6.43E-04	4.308	-0.00138	7.78E-04	4.308	-0.00168	9.15E-04	4.308	-0.00196	0.00104	4.308	-0.00222	0.00115
4.309	-6.43E-04	6.43E-04	4.309	-0.00138	7.78E-04	4.309	-0.00168	9.14E-04	4.309	-0.00196	0.00104	4.309	-0.00222	0.00115
4.31	-6.42E-04	6.42E-04	4.31	-0.00138	7.77E-04	4.31	-0.00168	9.14E-04	4.31	-0.00196	0.00104	4.31	-0.00222	0.00115
4.311	-6.42E-04	6.42E-04	4.311	-0.00138	7.77E-04	4.311	-0.00168	9.13E-04	4.311	-0.00196	0.00104	4.311	-0.00221	0.00115
4.312	-6.42E-04	6.42E-04	4.312	-0.00138	7.76E-04	4.312	-0.00168	9.12E-04	4.312	-0.00195	0.00103	4.312	-0.00221	0.00115
4.313	-6.41E-04	6.41E-04	4.313	-0.00138	7.76E-04	4.313	-0.00168	9.12E-04	4.313	-0.00195	0.00103	4.313	-0.00221	0.00114
4.314	-6.41E-04	6.41E-04	4.314	-0.00138	7.75E-04	4.314	-0.00167	9.11E-04	4.314	-0.00195	0.00103	4.314	-0.00221	0.00114
4.315	-6.41E-04	6.41E-04	4.315	-0.00138	7.75E-04	4.315	-0.00167	9.10E-04	4.315	-0.00195	0.00103	4.315	-0.00221	0.00114
4.316	-6.40E-04	6.40E-04	4.316	-0.00138	7.74E-04	4.316	-0.00167	9.10E-04	4.316	-0.00195	0.00103	4.316	-0.00221	0.00114
4.317	-6.40E-04	6.40E-04	4.317	-0.00137	7.74E-04	4.317	-0.00167	9.09E-04	4.317	-0.00195	0.00103	4.317	-0.00221	0.00114
4.318	-6.40E-04	6.40E-04	4.318	-0.00137	7.73E-04	4.318	-0.00167	9.09E-04	4.318	-0.00195	0.00103	4.318	-0.0022	0.00114
4.319	-6.40E-04	6.40E-04	4.319	-0.00137	7.73E-04	4.319	-0.00167	9.08E-04	4.319	-0.00194	0.00103	4.319	-0.0022	0.00114
4.32	-6.39E-04	6.39E-04	4.32	-0.00137	7.72E-04	4.32	-0.00167	9.08E-04	4.32	-0.00194	0.00103	4.32	-0.0022	0.00114
4.321	-6.39E-04	6.39E-04	4.321	-0.00137	7.72E-04	4.321	-0.00167	9.07E-04	4.321	-0.00194	0.00103	4.321	-0.0022	0.00114
4.322	-6.39E-04	6.39E-04	4.322	-0.00137	7.71E-04	4.322	-0.00167	9.06E-04	4.322	-0.00194	0.00103	4.322	-0.0022	0.00114
4.323	-6.38E-04	6.38E-04	4.323	-0.00137	7.71E-04	4.323	-0.00166	9.06E-04	4.323	-0.00194	0.00103	4.323	-0.0022	0.00114
4.324	-6.38E-04	6.38E-04	4.324	-0.00137	7.70E-04	4.324	-0.00166	9.05E-04	4.324	-0.00194	0.00103	4.324	-0.00219	0.00114
4.325	-6.38E-04	6.38E-04	4.325	-0.00137	7.70E-04	4.325	-0.00166	9.05E-04	4.325	-0.00194	0.00103	4.325	-0.00219	0.00114
4.326	-6.37E-04	6.37E-04	4.326	-0.00137	7.69E-04	4.326	-0.00166	9.04E-04	4.326	-0.00194	0.00102	4.326	-0.00219	0.00113
4.327	-6.37E-04	6.37E-04	4.327	-0.00137	7.69E-04	4.327	-0.00166	9.03E-04	4.327	-0.00193	0.00102	4.327	-0.00219	0.00113
4.328	-6.37E-04	6.37E-04	4.328	-0.00136	7.68E-04	4.328	-0.00166	9.03E-04	4.328	-0.00193	0.00102	4.328	-0.00219	0.00113
4.329	-6.37E-04	6.37E-04	4.329	-0.00136	7.68E-04	4.329	-0.00166	9.02E-04	4.329	-0.00193	0.00102	4.329	-0.00219	0.00113
4.33	-6.36E-04	6.36E-04	4.33	-0.00136	7.67E-04	4.33	-0.00166	9.02E-04	4.33	-0.00193	0.00102	4.33	-0.00219	0.00113
4.331	-6.36E-04	6.36E-04	4.331	-0.00136	7.67E-04	4.331	-0.00166	9.01E-04	4.331	-0.00193	0.00102	4.331	-0.00218	0.00113
4.332	-6.36E-04	6.36E-04	4.332	-0.00136	7.66E-04	4.332	-0.00165	9.00E-04	4.332	-0.00193	0.00102	4.332	-0.00218	0.00113
4.333	-6.35E-04	6.35E-04	4.333	-0.00136	7.66E-04	4.333	-0.00165	9.00E-04	4.333	-0.00193	0.00102	4.333	-0.00218	0.00113
4.334	-6.35E-04	6.35E-04	4.334	-0.00136	7.65E-04	4.334	-0.00165	8.99E-04	4.334	-0.00193	0.00102	4.334	-0.00218	0.00113
4.335	-6.35E-04	6.35E-04	4.335	-0.00136	7.65E-04	4.335	-0.00165	8.99E-04	4.335	-0.00192	0.00102	4.335	-0.00218	0.00113
4.336	-6.35E-04	6.35E-04	4.336	-0.00136	7.64E-04	4.336	-0.00165	8.98E-04	4.336	-0.00192	0.00102	4.336	-0.00218	0.00113
4.337	-6.34E-04	6.34E-04	4.337	-0.00136	7.64E-04	4.337	-0.00165	8.97E-04	4.337	-0.00192	0.00102	4.337	-0.00218	0.00113
4.338	-6.34E-04	6.34E-04	4.338	-0.00136	7.63E-04	4.338	-0.00165	8.97E-04	4.338	-0.00192	0.00102	4.338	-0.00217	0.00113
4.339	-6.34E-04	6.34E-04	4.339	-0.00135	7.63E-04	4.339	-0.00165	8.96E-04	4.339	-0.00192	0.00102	4.339	-0.00217	0.00112
4.34	-6.33E-04	6.33E-04	4.34	-0.00135	7.62E-04	4.34	-0.00165	8.96E-04	4.34	-0.00192	0.00102	4.34	-0.00217	0.00112
4.341	-6.33E-04	6.33E-04	4.341	-0.00135	7.62E-04	4.341	-0.00165	8.95E-04	4.341	-0.00192	0.00101	4.341	-0.00217	0.00112
4.342	-6.33E-04	6.33E-04	4.342	-0.00135	7.61E-04	4.342	-0.00164	8.95E-04	4.342	-0.00192	0.00101	4.342	-0.00217	0.00112
4.343	-6.32E-04	6.32E-04	4.343	-0.00135	7.61E-04	4.343	-0.00164	8.94E-04	4.343	-0.00191	0.00101	4.343	-0.00217	0.00112
4.344	-6.32E-04	6.32E-04	4.344	-0.00135	7.60E-04	4.344	-0.00164	8.93E-04	4.344	-0.00191	0.00101	4.344	-0.00217	0.00112
4.345	-6.32E-04	6.32E-04	4.345	-0.00135	7.60E-04	4.345	-0.00164	8.93E-04	4.345	-0.00191	0.00101	4.345	-0.00216	0.00112
4.346	-6.32E-04	6.32E-04	4.346	-0.00135	7.60E-04	4.346	-0.00164	8.92E-04	4.346	-0.00191	0.00101	4.346	-0.00216	0.00112
4.347	-6.31E-04	6.31E-04	4.347	-0.00135	7.59E-04	4.347	-0.00164	8.92E-04	4.347	-0.00191	0.00101	4.347	-0.00216	0.00112
4.348	-6.31E-04	6.31E-04	4.348	-0.00135	7.59E-04	4.348	-0.00164	8.91E-04	4.348	-0.00191	0.00101	4.348	-0.00216	0.00112
4.349	-6.31E-04	6.31E-04	4.349	-0.00135	7.58E-04	4.349	-0.00164	8.90E-04	4.349	-0.00191	0.00101	4.349	-0.00216	0.00112
4.35	-6.30E-04	6.30E-04	4.35	-0.00135	7.58E-04	4.35	-0.00164	8.90E-04	4.35	-0.00191	0.00101	4.35	-0.00216	0.00112
4.351	-6.30E-04	6.30E-04	4.351	-0.00134	7.57E-04	4.351	-0.00163	8.89E-04	4.351	-0.0019	0.00101	4.351	-0.00215	0.00112
4.352	-6.30E-04	6.30E-04	4.352	-0.00134	7.57E-04	4.352	-0.00163	8.89E-04	4.352	-0.0019	0.00101	4.352	-0.00215	0.00112
4.353	-6.30E-04	6.30E-04	4.353	-0.00134	7.56E-04	4.353	-0.00163	8.88E-04	4.353	-0.0019	0.00101	4.353	-0.00215	0.00111
4.354	-6.29E-04	6.29E-04	4.354	-0.00134	7.56E-04	4.354	-0.00163	8.88E-04	4.354	-0.0019	0.00101	4.354	-0.00215	0.00111
4.355	-6.29E-04	6.29E-04	4.355	-0.00134	7.55E-04	4.355	-0.00163	8.87E-04	4.355	-0.0019	0.00101	4.355	-0.00215	0.00111
4.356	-6.29E-04	6.29E-04	4.356	-0.00134	7.55E-04	4.356	-0.00163	8.86E-04	4.356	-0.0019	0.001	4.356	-0.00215	0.00111
4.357	-6.28E-04	6.28E-04	4.357	-0.00134	7.54E-04	4.357	-0.00163	8.86E-04	4.357	-0.0019	0.001	4.357	-0.00215	0.00111
4.358	-6.28E-04	6.28E-04	4.358	-0.00134	7.54E-04	4.358	-0.00163	8.85E-04	4.358	-0.0019	0.001	4.358	-0.00214	0.00111
4.359	-6.28E-04	6.28E-04	4.359	-0.00134	7.53E-04	4.359	-0.00163	8.85E-04	4.359	-0.00189	0.001	4.359	-0.00214	0.00111
4.36	-6.28E-04	6.28E-04	4.36	-0.00134	7.53E-04	4.36	-0.00163	8.84E-04	4.36	-0.00189	0.001	4.36	-0.00214	0.00111
4.361	-6.27E-04	6.27E-04	4.361	-0.00134	7.52E-04	4.361	-0.00162	8.84E-04	4.361	-0.00189	0.001	4.361	-0.00214	0.00111
4.362	-6.27E-04	6.27E-04	4.362	-0.00134	7.52E-04	4.362	-0.00162	8.83E-04	4.362	-0.00189	0.001	4.362	-0.00214	0.00111
4.363	-6.27E-04	6.27E-04	4.363	-0.00133	7.51E-04	4.363	-0.00162	8.82E-04	4.363	-0.00189	1.00E-03	4.363	-0.00214	0.00111
4.364	-6.26E-04	6.26E-04	4.364	-0.00133	7.51E-04	4.364	-0.00162	8.82E-04	4.364	-0.00189	9.99E-04	4.364	-0.00214	0.00111
4.365	-6.26E-04	6.26E-04	4.365	-0.00133	7.50E-04	4.365	-0.00162	8.81E-04	4.365	-0.00189	9.99E-04	4.365	-0.00213	0.00111
4.366	-6.26E-04	6.26E-04	4.366	-0.00133	7.50E-04	4.366	-0.00162	8.81E-04	4.366	-0.00189	9.98E-04	4.366	-0.00213	0.0011
4.367	-6.26E-04	6.26E-04	4.367	-0.00133	7.49E-04	4.367	-0.00162	8.80E-04	4.367	-0.00188	9.97E-04	4.367	-0.00213	0.0011
4.368	-6.25E-04	6.25E-04	4.368	-0.00133	7.49E-04	4.368	-0.00162	8.79E-04	4.368	-0.00188	9.97E-04	4.368	-0.00213	0.0011
4.369	-6.25E-04	6.25E-04	4.369	-0.00133	7.48E-04	4.369	-0.00162	8.79E-04	4.369	-0.00188	9.96E-04	4.369	-0.00213	0.0011
4.37	-6.25E-04	6.25E-04	4.37	-0.00133	7.48E-04	4.37	-0.00161	8.78E-04	4.37	-0.00188	9.95E-04	4.37	-0.00213	0.0011
4.371	-6.24E-04	6.24E-04	4.371	-0.00133	7.48E-04	4.371	-0.00161	8.78E-04	4.371	-0.00188	9.95E-04	4.371	-0.00213	0.0011
4.372	-6.24E-04	6.24E-04	4.372	-0.00133	7.47E-04	4.372	-0.00161	8.77E-04	4.372	-0.00188	9.94E-04	4.372	-0.00212	0.0011
4.373	-6.24E-04	6.24E-04	4.373	-0.00133	7.47E-04	4.373	-0.00161	8.77E-04	4.373	-0.00188	9.93E-04	4.373	-0.00212	0.0011
4.374	-6.24E-04	6.24E-04	4.374	-0.00133	7.46E-04	4.374	-0.00161	8.76E-04	4.374	-0.00188	9.93E-04	4.374	-0.00212	0.0011
4.375	-6.23E-04	6.23E-04	4.375	-0.00132	7.46E-04	4.375	-0.00161	8.76E-04	4.375	-0.00187	9.92E-04	4.375	-0.00212	0.0011
4.376	-6.23E-04	6.23E-04	4.376	-0.00132	7.45E-04	4.376	-0.00161	8.75E-04	4.376	-0.00187	9.91E-04	4.376	-0.00212	0.0011
4.377	-6.23E-04	6.23E-04	4.377	-0.00132	7.45E-04	4.377	-0.00161	8.74E-04	4.377	-0.00187	9.91E-04	4.377	-0.00212	0.0011
4.378	-6.22E-04	6.22E-04	4.378	-0.00132	7.44E-04	4.378	-0.00161	8.74E-04	4.378	-0.00187	9.90E-04	4.378	-0.00212	0.0011
4.379	-6.22E-04	6.22E-04	4.379	-0.00132	7.44E-04	4.379	-0.00161	8.73E-04	4.379	-0.00187	9.89E-04	4.379	-0.00211	0.0011
4.38	-6.22E-04	6.22E-04	4.38	-0.00132	7.43E-04	4.38	-0.0016	8.73E-04	4.38	-0.00187	9.89E-04	4.38	-0.00211	0.00109
4.381	-6.22E-04	6.22E-04	4.381	-0.00132	7.43E-04	4.381	-0.0016	8.72E-04	4.381	-0.00187	9.88E-04	4.381	-0.00211	0.00109
4.382	-6.21E-04	6.21E-04	4.382	-0.00132	7.42E-04	4.382	-0.0016	8.72E-04	4.382	-0.00187	9.88E-04	4.382	-0.00211	0.00109
4.383	-6.21E-04	6.21E-04	4.383	-0.00132	7.42E-04	4.383	-0.0016	8.71E-04	4.383	-0.00186	9.87E-04	4.383	-0.00211	0.00109
4.384	-6.21E-04	6.21E-04	4.384	-0.00132	7.41E-04	4.384	-0.0016	8.70E-04	4.384	-0.00186	9.86E-04	4.384	-0.00211	0.00109
4.385	-6.20E-04	6.20E-04	4.385	-0.00132	7.41E-04	4.385	-0.0016	8.70E-04	4.385	-0.00186	9.86E-04	4.385	-0.00211	0.00109
4.386	-6.20E-04	6.20E-04	4.386	-0.00132	7.40E-04	4.386	-0.0016	8.69E-04	4.386	-0.00186	9.85E-04	4.386	-0.0021	0.00109
4.387	-6.20E-04	6.20E-04	4.387	-0.00131	7.40E-04	4.387	-0.0016	8.69E-04	4.387	-0.00186	9.84E-04	4.387	-0.0021	0.00109
4.388	-6.20E-04	6.20E-04	4.388	-0.00131	7.40E-04	4.388	-0.0016	8.68E-04	4.388	-0.00186	9.84E-04	4.388	-0.0021	0.00109
4.389	-6.19E-04	6.19E-04	4.389	-0.00131	7.39E-04	4.389	-0.00159	8.68E-04	4.389	-0.00186	9.83E-04	4.389	-0.0021	0.00109
4.39	-6.19E-04	6.19E-04	4.39	-0.00131	7.39E-04	4.39	-0.00159	8.67E-04	4.39	-0.00186	9.82E-04	4.39	-0.0021	0.00109
4.391	-6.19E-04	6.19E-04	4.391	-0.00131	7.38E-04	4.391	-0.00159	8.66E-04	4.391	-0.00185	9.82E-04	4.391	-0.0021	0.00109
4.392	-6.18E-04	6.18E-04	4.392	-0.00131	7.38E-04	4.392	-0.00159	8.66E-04	4.392	-0.00185	9.81E-04	4.392	-0.0021	0.00109
4.393	-6.18E-04	6.18E-04	4.393	-0.00131	7.37E-04	4.393	-0.00159	8.65E-04	4.393	-0.00185	9.80E-04	4.393	-0.0021	0.00108
4.394	-6.18E-04	6.18E-04	4.394	-0.00131	7.37E-04	4.394	-0.00159	8.65E-04	4.394	-0.00185	9.80E-04	4.394	-0.00209	0.00108
4.395	-6.18E-04	6.18E-04	4.395	-0.00131	7.36E-04	4.395	-0.00159	8.64E-04	4.395	-0.00185	9.79E-04	4.395	-0.00209	0.00108
4.396	-6.17E-04	6.17E-04	4.396	-0.00131	7.36E-04	4.396	-0.00159	8.64E-04	4.396	-0.00185	9.78E-04	4.396	-0.00209	0.00108
4.397	-6.17E-04	6.17E-04	4.397	-7.35E-04	7.35E-04	4.397	-0.00159	8.63E-04	4.397	-0.00185	9.78E-04	4.397	-0.00209	0.00108
4.398	-6.17E-04	6.17E-04	4.398	-7.35E-04	7.35E-04	4.398	-0.00159	8.63E-04	4.398	-0.00185	9.77E-04	4.398	-0.00209	0.00108
4.399	-6.16E-04	6.16E-04	4.399	-7.35E-04	7.35E-04	4.399	-0.00158	8.62E-04	4.399	-0.00184	9.77E-04	4.399	-0.00209	0.00108
4.4	-6.16E-04	6.16E-04	4.4	-7.34E-04	7.34E-04	4.4	-0.00158	8.61E-04	4.4	-0.00184	9.76E-04	4.4	-0.00209	0.00108
4.401	-6.16E-04	6.16E-04	4.401	-7.34E-04	7.34E-04	4.401	-0.00158	8.61E-04	4.401	-0.00184	9.75E-04	4.401	-0.00208	0.00108
4.402	-6.16E-04	6.16E-04	4.402	-7.34E-04	7.34E-04	4.402	-0.00158	8.60E-04	4.402	-0.00184	9.75E-04	4.402	-0.00208	0.00108
4.403	-6.15E-04	6.15E-04	4.403	-7.33E-04	7.33E-04	4.403	-0.00158	8.60E-04	4.403	-0.00184	9.74E-04	4.403	-0.00208	0.00108
4.404	-6.15E-04	6.15E-04	4.404	-7.33E-04	7.33E-04	4.404	-0.00158	8.59E-04	4.404	-0.00184	9.73E-04	4.404	-0.00208	0.00108
4.405	-6.15E-04	6.15E-04	4.405	-7.33E-04	7.33E-04	4.405	-0.00158	8.59E-04	4.405	-0.00184	9.73E-04	4.405	-0.00208	0.00108
4.406	-6.15E-04	6.15E-04	4.406	-7.32E-04	7.32E-04	4.406	-0.00158	8.58E-04	4.406	-0.00184	9.72E-04	4.406	-0.00208	0.00108
4.407	-6.14E-04	6.14E-04	4.407	-7.32E-04	7.32E-04	4.407	-0.00158	8.58E-04	4.407	-0.00183	9.71E-04	4.407	-0.00208	0.00107
4.408	-6.14E-04	6.14E-04	4.408	-7.32E-04	7.32E-04	4.408	-0.00158	8.57E-04	4.408	-0.00183	9.71E-04	4.408	-0.00207	0.00107
4.409	-6.14E-04	6.14E-04	4.409	-7.31E-04	7.31E-04	4.409	-0.00157	8.56E-04	4.409	-0.00183	9.70E-04	4.409	-0.00207	0.00107
4.41	-6.13E-04	6.13E-04	4.41	-7.31E-04	7.31E-04	4.41	-0.00157	8.56E-04	4.41	-0.00183	9.70E-04	4.41	-0.00207	0.00107
4.411	-6.13E-04	6.13E-04	4.411	-7.31E-04	7.31E-04	4.411	-0.00157	8.55E-04	4.411	-0.00183	9.69E-04	4.411	-0.00207	0.00107
4.412	-6.13E-04	6.13E-04	4.412	-7.30E-04	7.30E-04	4.412	-0.00157	8.55E-04	4.412	-0.00183	9.68E-04	4.412	-0.00207	0.00107
4.413	-6.13E-04	6.13E-04	4.413	-7.30E-04	7.30E-04	4.413	-0.00157	8.54E-04	4.413	-0.00183	9.68E-04	4.413	-0.00207	0.00107
4.414	-6.12E-04	6.12E-04	4.414	-7.30E-04	7.30E-04	4.414	-0.00157	8.54E-04	4.414	-0.00183	9.67E-04	4.414	-0.00207	0.00107
4.415	-6.12E-04	6.12E-04	4.415	-7.29E-04	7.29E-04	4.415	-0.00157	8.53E-04	4.415	-0.00183	9.66E-04	4.415	-0.00206	0.00107
4.416	-6.12E-04	6.12E-04	4.416	-7.29E-04	7.29E-04	4.416	-0.00157	8.53E-04	4.416	-0.00182	9.66E-04	4.416	-0.00206	0.00107
4.417	-6.11E-04	6.11E-04	4.417	-7.29E-04	7.29E-04	4.417	-0.00157	8.52E-04	4.417	-0.00182	9.65E-04	4.417	-0.00206	0.00107
4.418	-6.11E-04	6.11E-04	4.418	-7.28E-04	7.28E-04	4.418	-0.00157	8.52E-04	4.418	-0.00182	9.64E-04	4.418	-0.00206	0.00107
4.419	-6.11E-04	6.11E-04	4.419	-7.28E-04	7.28E-04	4.419	-0.00156	8.51E-04	4.419	-0.00182	9.64E-04	4.419	-0.00206	0.00107
4.42	-6.11E-04	6.11E-04	4.42	-7.28E-04	7.28E-04	4.42	-0.00156	8.50E-04	4.42	-0.00182	9.63E-04	4.42	-0.00206	0.00107
4.421	-6.10E-04	6.10E-04	4.421	-7.27E-04	7.27E-04	4.421	-0.00156	8.50E-04	4.421	-0.00182	9.63E-04	4.421	-0.00206	0.00106
4.422	-6.10E-04	6.10E-04	4.422	-7.27E-04	7.27E-04	4.422	-0.00156	8.49E-04	4.422	-0.00182	9.62E-04	4.422	-0.00206	0.00106
4.423	-6.10E-04	6.10E-04	4.423	-7.27E-04	7.27E-04	4.423	-0.00156	8.49E-04	4.423	-0.00182	9.61E-04	4.423	-0.00205	0.00106
4.424	-6.10E-04	6.10E-04	4.424	-7.26E-04	7.26E-04	4.424	-0.00156	8.48E-04	4.424	-0.00181	9.61E-04	4.424	-0.00205	0.00106
4.425	-6.09E-04	6.09E-04	4.425	-7.26E-04	7.26E-04	4.425	-0.00156	8.48E-04	4.425	-0.00181	9.60E-04	4.425	-0.00205	0.00106
4.426	-6.09E-04	6.09E-04	4.426	-7.26E-04	7.26E-04	4.426	-0.00156	8.47E-04	4.426	-0.00181	9.59E-04	4.426	-0.00205	0.00106
4.427	-6.09E-04	6.09E-04	4.427	-7.25E-04	7.25E-04	4.427	-0.00156	8.47E-04	4.427	-0.00181	9.59E-04	4.427	-0.00205	0.00106
4.428	-6.08E-04	6.08E-04	4.428	-7.25E-04	7.25E-04	4.428	-0.00156	8.46E-04	4.428	-0.00181	9.58E-04	4.428	-0.00205	0.00106
4.429	-6.08E-04	6.08E-04	4.429	-7.25E-04	7.25E-04	4.429	-0.00155	8.46E-04	4.429	-0.00181	9.58E-04	4.429	-0.00205	0.00106
4.43	-6.08E-04	6.08E-04	4.43	-7.24E-04	7.24E-04	4.43	-0.00155	8.45E-04	4.43	-0.00181	9.57E-04	4.43	-0.00204	0.00106
4.431	-6.08E-04	6.08E-04	4.431	-7.24E-04	7.24E-04	4.431	-0.00155	8.44E-04	4.431	-0.00181	9.56E-04	4.431	-0.00204	0.00106
4.432	-6.07E-04	6.07E-04	4.432	-7.24E-04	7.24E-04	4.432	-0.00155	8.44E-04	4.432	-0.00181	9.56E-04	4.432	-0.00204	0.00106
4.433	-6.07E-04	6.07E-04	4.433	-7.23E-04	7.23E-04	4.433	-0.00155	8.43E-04	4.433	-0.0018	9.55E-04	4.433	-0.00204	0.00106
4.434	-6.07E-04	6.07E-04	4.434	-7.23E-04	7.23E-04	4.434	-0.00155	8.43E-04	4.434	-0.0018	9.54E-04	4.434	-0.00204	0.00106
4.435	-6.07E-04	6.07E-04	4.435	-7.23E-04	7.23E-04	4.435	-0.00155	8.42E-04	4.435	-0.0018	9.54E-04	4.435	-0.00204	0.00106
4.436	-6.06E-04	6.06E-04	4.436	-7.22E-04	7.22E-04	4.436	-0.00155	8.42E-04	4.436	-0.0018	9.53E-04	4.436	-0.00204	0.00105
4.437	-6.06E-04	6.06E-04	4.437	-7.22E-04	7.22E-04	4.437	-0.00155	8.41E-04	4.437	-0.0018	9.53E-04	4.437	-0.00203	0.00105
4.438	-6.06E-04	6.06E-04	4.438	-7.22E-04	7.22E-04	4.438	-0.00155	8.41E-04	4.438	-0.0018	9.52E-04	4.438	-0.00203	0.00105
4.439	-6.05E-04	6.05E-04	4.439	-7.21E-04	7.21E-04	4.439	-0.00154	8.40E-04	4.439	-0.0018	9.51E-04	4.439	-0.00203	0.00105
4.44	-6.05E-04	6.05E-04	4.44	-7.21E-04	7.21E-04	4.44	-0.00154	8.40E-04	4.44	-0.0018	9.51E-04	4.44	-0.00203	0.00105
4.441	-6.05E-04	6.05E-04	4.441	-7.21E-04	7.21E-04	4.441	-0.00154	8.39E-04	4.441	-0.00179	9.50E-04	4.441	-0.00203	0.00105
4.442	-6.05E-04	6.05E-04	4.442	-7.21E-04	7.21E-04	4.442	-0.00154	8.39E-04	4.442	-0.00179	9.49E-04	4.442	-0.00203	0.00105
4.443	-6.04E-04	6.04E-04	4.443	-7.20E-04	7.20E-04	4.443	-0.00154	8.38E-04	4.443	-0.00179	9.49E-04	4.443	-0.00203	0.00105
4.444	-6.04E-04	6.04E-04	4.444	-7.20E-04	7.20E-04	4.444	-0.00154	8.37E-04	4.444	-0.00179	9.48E-04	4.444	-0.00203	0.00105
4.445	-6.04E-04	6.04E-04	4.445	-7.20E-04	7.20E-04	4.445	-0.00154	8.37E-04	4.445	-0.00179	9.48E-04	4.445	-0.00202	0.00105
4.446	-6.04E-04	6.04E-04	4.446	-7.19E-04	7.19E-04	4.446	-0.00154	8.36E-04	4.446	-0.00179	9.47E-04	4.446	-0.00202	0.00105
4.447	-6.03E-04	6.03E-04	4.447	-7.19E-04	7.19E-04	4.447	-0.00154	8.36E-04	4.447	-0.00179	9.46E-04	4.447	-0.00202	0.00105
4.448	-6.03E-04	6.03E-04	4.448	-7.19E-04	7.19E-04	4.448	-0.00154	8.35E-04	4.448	-0.00179	9.46E-04	4.448	-0.00202	0.00105
4.449	-6.03E-04	6.03E-04	4.449	-7.18E-04	7.18E-04	4.449	-0.00153	8.35E-04	4.449	-0.00179	9.45E-04	4.449	-0.00202	0.00105
4.45	-6.02E-04	6.02E-04	4.45	-7.18E-04	7.18E-04	4.45	-0.00153	8.34E-04	4.45	-0.00178	9.45E-04	4.45	-0.00202	0.00104
4.451	-6.02E-04	6.02E-04	4.451	-7.18E-04	7.18E-04	4.451	-0.00153	8.34E-04	4.451	-0.00178	9.44E-04	4.451	-0.00202	0.00104
4.452	-6.02E-04	6.02E-04	4.452	-7.17E-04	7.17E-04	4.452	-0.00153	8.33E-04	4.452	-0.00178	9.43E-04	4.452	-0.00201	0.00104
4.453	-6.02E-04	6.02E-04	4.453	-7.17E-04	7.17E-04	4.453	-0.00153	8.33E-04	4.453	-0.00178	9.43E-04	4.453	-0.00201	0.00104
4.454	-6.01E-04	6.01E-04	4.454	-7.17E-04	7.17E-04	4.454	-0.00153	8.32E-04	4.454	-0.00178	9.42E-04	4.454	-0.00201	0.00104
4.455	-6.01E-04	6.01E-04	4.455	-7.16E-04	7.16E-04	4.455	-0.00153	8.32E-04	4.455	-0.00178	9.41E-04	4.455	-0.00201	0.00104
4.456	-6.01E-04	6.01E-04	4.456	-7.16E-04	7.16E-04	4.456	-0.00153	8.31E-04	4.456	-0.00178	9.41E-04	4.456	-0.00201	0.00104
4.457	-6.01E-04	6.01E-04	4.457	-7.16E-04	7.16E-04	4.457	-0.00153	8.31E-04	4.457	-0.00178	9.40E-04	4.457	-0.00201	0.00104
4.458	-6.00E-04	6.00E-04	4.458	-7.15E-04	7.15E-04	4.458	-0.00153	8.30E-04	4.458	-0.00177	9.40E-04	4.458	-0.00201	0.00104
4.459	-6.00E-04	6.00E-04	4.459	-7.15E-04	7.15E-04	4.459	-0.00152	8.30E-04	4.459	-0.00177	9.39E-04	4.459	-0.00201	0.00104
4.46	-6.00E-04	6.00E-04	4.46	-7.15E-04	7.15E-04	4.46	-0.00152	8.29E-04	4.46	-0.00177	9.38E-04	4.46	-0.002	0.00104
4.461	-5.99E-04	5.99E-04	4.461	-7.14E-04	7.14E-04	4.461	-0.00152	8.28E-04	4.461	-0.00177	9.38E-04	4.461	-0.002	0.00104
4.462	-5.99E-04	5.99E-04	4.462	-7.14E-04	7.14E-04	4.462	-0.00152	8.28E-04	4.462	-0.00177	9.37E-04	4.462	-0.002	0.00104
4.463	-5.99E-04	5.99E-04	4.463	-7.14E-04	7.14E-04	4.463	-0.00152	8.27E-04	4.463	-0.00177	9.37E-04	4.463	-0.002	0.00104
4.464	-5.99E-04	5.99E-04	4.464	-7.13E-04	7.13E-04	4.464	-0.00152	8.27E-04	4.464	-0.00177	9.36E-04	4.464	-0.002	0.00104
4.465	-5.98E-04	5.98E-04	4.465	-7.13E-04	7.13E-04	4.465	-0.00152	8.26E-04	4.465	-0.00177	9.35E-04	4.465	-0.002	0.00103
4.466	-5.98E-04	5.98E-04	4.466	-7.13E-04	7.13E-04	4.466	-0.00152	8.26E-04	4.466	-0.00177	9.35E-04	4.466	-0.002	0.00103
4.467	-5.98E-04	5.98E-04	4.467	-7.12E-04	7.12E-04	4.467	-0.00152	8.25E-04	4.467	-0.00176	9.34E-04	4.467	-0.00199	0.00103
4.468	-5.98E-04	5.98E-04	4.468	-7.12E-04	7.12E-04	4.468	-0.00152	8.25E-04	4.468	-0.00176	9.34E-04	4.468	-0.00199	0.00103
4.469	-5.97E-04	5.97E-04	4.469	-7.12E-04	7.12E-04	4.469	-0.00152	8.24E-04	4.469	-0.00176	9.33E-04	4.469	-0.00199	0.00103
4.47	-5.97E-04	5.97E-04	4.47	-7.12E-04	7.12E-04	4.47	-0.00151	8.24E-04	4.47	-0.00176	9.32E-04	4.47	-0.00199	0.00103
4.471	-5.97E-04	5.97E-04	4.471	-7.11E-04	7.11E-04	4.471	-0.00151	8.23E-04	4.471	-0.00176	9.32E-04	4.471	-0.00199	0.00103
4.472	-5.97E-04	5.97E-04	4.472	-7.11E-04	7.11E-04	4.472	-0.00151	8.23E-04	4.472	-0.00176	9.31E-04	4.472	-0.00199	0.00103
4.473	-5.96E-04	5.96E-04	4.473	-7.11E-04	7.11E-04	4.473	-0.00151	8.22E-04	4.473	-0.00176	9.31E-04	4.473	-0.00199	0.00103
4.474	-5.96E-04	5.96E-04	4.474	-7.10E-04	7.10E-04	4.474	-0.00151	8.22E-04	4.474	-0.00176	9.30E-04	4.474	-0.00199	0.00103
4.475	-5.96E-04	5.96E-04	4.475	-7.10E-04	7.10E-04	4.475	-0.00151	8.21E-04	4.475	-0.00176	9.29E-04	4.475	-0.00198	0.00103
4.476	-5.95E-04	5.95E-04	4.476	-7.10E-04	7.10E-04	4.476	-0.00151	8.21E-04	4.476	-0.00175	9.29E-04	4.476	-0.00198	0.00103
4.477	-5.95E-04	5.95E-04	4.477	-7.09E-04	7.09E-04	4.477	-0.00151	8.20E-04	4.477	-0.00175	9.28E-04	4.477	-0.00198	0.00103

4.478	-5.95E-04	5.95E-04	4.478	-7.09E-04	7.09E-04	4.478	-0.00151	8.20E-04	4.478	-0.00175	9.28E-04	4.478	-0.00198	0.00103
4.479	-5.95E-04	5.95E-04	4.479	-7.09E-04	7.09E-04	4.479	-0.00151	8.19E-04	4.479	-0.00175	9.27E-04	4.479	-0.00198	0.00102
4.48	-5.94E-04	5.94E-04	4.48	-7.08E-04	7.08E-04	4.48	-0.0015	8.19E-04	4.48	-0.00175	9.26E-04	4.48	-0.00198	0.00102
4.481	-5.94E-04	5.94E-04	4.481	-7.08E-04	7.08E-04	4.481	-0.0015	8.18E-04	4.481	-0.00175	9.26E-04	4.481	-0.00198	0.00102
4.482	-5.94E-04	5.94E-04	4.482	-7.08E-04	7.08E-04	4.482	-0.0015	8.17E-04	4.482	-0.00175	9.25E-04	4.482	-0.00198	0.00102
4.483	-5.94E-04	5.94E-04	4.483	-7.07E-04	7.07E-04	4.483	-0.0015	8.17E-04	4.483	-0.00175	9.25E-04	4.483	-0.00197	0.00102
4.484	-5.93E-04	5.93E-04	4.484	-7.07E-04	7.07E-04	4.484	-0.0015	8.16E-04	4.484	-0.00175	9.24E-04	4.484	-0.00197	0.00102
4.485	-5.93E-04	5.93E-04	4.485	-7.07E-04	7.07E-04	4.485	-0.0015	8.16E-04	4.485	-0.00174	9.23E-04	4.485	-0.00197	0.00102
4.486	-5.93E-04	5.93E-04	4.486	-7.06E-04	7.06E-04	4.486	-0.0015	8.15E-04	4.486	-0.00174	9.23E-04	4.486	-0.00197	0.00102
4.487	-5.93E-04	5.93E-04	4.487	-7.06E-04	7.06E-04	4.487	-0.0015	8.15E-04	4.487	-0.00174	9.22E-04	4.487	-0.00197	0.00102
4.488	-5.92E-04	5.92E-04	4.488	-7.06E-04	7.06E-04	4.488	-0.0015	8.14E-04	4.488	-0.00174	9.22E-04	4.488	-0.00197	0.00102
4.489	-5.92E-04	5.92E-04	4.489	-7.05E-04	7.05E-04	4.489	-0.0015	8.14E-04	4.489	-0.00174	9.21E-04	4.489	-0.00197	0.00102
4.49	-5.92E-04	5.92E-04	4.49	-7.05E-04	7.05E-04	4.49	-0.0015	8.13E-04	4.49	-0.00174	9.20E-04	4.49	-0.00197	0.00102
4.491	-5.91E-04	5.91E-04	4.491	-7.05E-04	7.05E-04	4.491	-0.00149	8.13E-04	4.491	-0.00174	9.20E-04	4.491	-0.00196	0.00102
4.492	-5.91E-04	5.91E-04	4.492	-7.05E-04	7.05E-04	4.492	-0.00149	8.12E-04	4.492	-0.00174	9.19E-04	4.492	-0.00196	0.00102
4.493	-5.91E-04	5.91E-04	4.493	-7.04E-04	7.04E-04	4.493	-0.00149	8.12E-04	4.493	-0.00174	9.19E-04	4.493	-0.00196	0.00102
4.494	-5.91E-04	5.91E-04	4.494	-7.04E-04	7.04E-04	4.494	-0.00149	8.11E-04	4.494	-0.00173	9.18E-04	4.494	-0.00196	0.00101
4.495	-5.90E-04	5.90E-04	4.495	-7.04E-04	7.04E-04	4.495	-0.00149	8.11E-04	4.495	-0.00173	9.17E-04	4.495	-0.00196	0.00101
4.496	-5.90E-04	5.90E-04	4.496	-7.03E-04	7.03E-04	4.496	-0.00149	8.10E-04	4.496	-0.00173	9.17E-04	4.496	-0.00196	0.00101
4.497	-5.90E-04	5.90E-04	4.497	-7.03E-04	7.03E-04	4.497	-0.00149	8.10E-04	4.497	-0.00173	9.16E-04	4.497	-0.00196	0.00101
4.498	-5.90E-04	5.90E-04	4.498	-7.03E-04	7.03E-04	4.498	-0.00149	8.09E-04	4.498	-0.00173	9.16E-04	4.498	-0.00195	0.00101
4.499	-5.89E-04	5.89E-04	4.499	-7.02E-04	7.02E-04	4.499	-0.00149	8.09E-04	4.499	-0.00173	9.15E-04	4.499	-0.00195	0.00101
4.5	-5.89E-04	5.89E-04	4.5	-7.02E-04	7.02E-04	4.5	-0.00149	8.08E-04	4.5	-0.00173	9.15E-04	4.5	-0.00195	0.00101
4.501	-5.89E-04	5.89E-04	4.501	-7.02E-04	7.02E-04	4.501	-0.00148	8.08E-04	4.501	-0.00173	9.14E-04	4.501	-0.00195	0.00101
4.502	-5.89E-04	5.89E-04	4.502	-7.01E-04	7.01E-04	4.502	-0.00148	8.07E-04	4.502	-0.00173	9.13E-04	4.502	-0.00195	0.00101
4.503	-5.88E-04	5.88E-04	4.503	-7.01E-04	7.01E-04	4.503	-0.00148	8.07E-04	4.503	-0.00172	9.13E-04	4.503	-0.00195	0.00101
4.504	-5.88E-04	5.88E-04	4.504	-7.01E-04	7.01E-04	4.504	-0.00148	8.06E-04	4.504	-0.00172	9.12E-04	4.504	-0.00195	0.00101
4.505	-5.88E-04	5.88E-04	4.505	-7.00E-04	7.00E-04	4.505	-0.00148	8.06E-04	4.505	-0.00172	9.12E-04	4.505	-0.00195	0.00101
4.506	-5.88E-04	5.88E-04	4.506	-7.00E-04	7.00E-04	4.506	-0.00148	8.05E-04	4.506	-0.00172	9.11E-04	4.506	-0.00194	0.00101
4.507	-5.87E-04	5.87E-04	4.507	-7.00E-04	7.00E-04	4.507	-0.00148	8.05E-04	4.507	-0.00172	9.10E-04	4.507	-0.00194	0.00101
4.508	-5.87E-04	5.87E-04	4.508	-7.00E-04	7.00E-04	4.508	-0.00148	8.04E-04	4.508	-0.00172	9.10E-04	4.508	-0.00194	0.00101
4.509	-5.87E-04	5.87E-04	4.509	-6.99E-04	6.99E-04	4.509	-0.00148	8.04E-04	4.509	-0.00172	9.09E-04	4.509	-0.00194	0.00101
4.51	-5.87E-04	5.87E-04	4.51	-6.99E-04	6.99E-04	4.51	-0.00148	8.03E-04	4.51	-0.00172	9.09E-04	4.51	-0.00194	0.001
4.511	-5.86E-04	5.86E-04	4.511	-6.99E-04	6.99E-04	4.511	-0.00148	8.03E-04	4.511	-0.00172	9.08E-04	4.511	-0.00194	0.001
4.512	-5.86E-04	5.86E-04	4.512	-6.98E-04	6.98E-04	4.512	-0.00147	8.02E-04	4.512	-0.00171	9.08E-04	4.512	-0.00194	0.001
4.513	-5.86E-04	5.86E-04	4.513	-6.98E-04	6.98E-04	4.513	-0.00147	8.02E-04	4.513	-0.00171	9.07E-04	4.513	-0.00194	0.001
4.514	-5.85E-04	5.85E-04	4.514	-6.98E-04	6.98E-04	4.514	-0.00147	8.01E-04	4.514	-0.00171	9.06E-04	4.514	-0.00193	0.001
4.515	-5.85E-04	5.85E-04	4.515	-6.97E-04	6.97E-04	4.515	-0.00147	8.01E-04	4.515	-0.00171	9.06E-04	4.515	-0.00193	0.001
4.516	-5.85E-04	5.85E-04	4.516	-6.97E-04	6.97E-04	4.516	-0.00147	8.00E-04	4.516	-0.00171	9.05E-04	4.516	-0.00193	0.001
4.517	-5.85E-04	5.85E-04	4.517	-6.97E-04	6.97E-04	4.517	-0.00147	8.00E-04	4.517	-0.00171	9.05E-04	4.517	-0.00193	1.00E-03
4.518	-5.84E-04	5.84E-04	4.518	-6.96E-04	6.96E-04	4.518	-0.00147	7.99E-04	4.518	-0.00171	9.04E-04	4.518	-0.00193	9.99E-04
4.519	-5.84E-04	5.84E-04	4.519	-6.96E-04	6.96E-04	4.519	-0.00147	7.99E-04	4.519	-0.00171	9.03E-04	4.519	-0.00193	9.99E-04
4.52	-5.84E-04	5.84E-04	4.52	-6.96E-04	6.96E-04	4.52	-0.00147	7.98E-04	4.52	-0.00171	9.03E-04	4.52	-0.00193	9.98E-04
4.521	-5.84E-04	5.84E-04	4.521	-6.96E-04	6.96E-04	4.521	-0.00147	7.98E-04	4.521	-0.0017	9.02E-04	4.521	-0.00193	9.97E-04
4.522	-5.83E-04	5.83E-04	4.522	-6.95E-04	6.95E-04	4.522	-0.00147	7.97E-04	4.522	-0.0017	9.02E-04	4.522	-0.00192	9.97E-04
4.523	-5.83E-04	5.83E-04	4.523	-6.95E-04	6.95E-04	4.523	-0.00146	7.97E-04	4.523	-0.0017	9.01E-04	4.523	-0.00192	9.96E-04
4.524	-5.83E-04	5.83E-04	4.524	-6.95E-04	6.95E-04	4.524	-0.00146	7.96E-04	4.524	-0.0017	9.01E-04	4.524	-0.00192	9.95E-04
4.525	-5.83E-04	5.83E-04	4.525	-6.94E-04	6.94E-04	4.525	-0.00146	7.96E-04	4.525	-0.0017	9.00E-04	4.525	-0.00192	9.95E-04
4.526	-5.82E-04	5.82E-04	4.526	-6.94E-04	6.94E-04	4.526	-0.00146	7.95E-04	4.526	-0.0017	8.99E-04	4.526	-0.00192	9.94E-04
4.527	-5.82E-04	5.82E-04	4.527	-6.94E-04	6.94E-04	4.527	-0.00146	7.95E-04	4.527	-0.0017	8.99E-04	4.527	-0.00192	9.93E-04
4.528	-5.82E-04	5.82E-04	4.528	-6.93E-04	6.93E-04	4.528	-0.00146	7.94E-04	4.528	-0.0017	8.98E-04	4.528	-0.00192	9.93E-04
4.529	-5.82E-04	5.82E-04	4.529	-6.93E-04	6.93E-04	4.529	-0.00146	7.94E-04	4.529	-0.0017	8.98E-04	4.529	-0.00192	9.92E-04
4.53	-5.81E-04	5.81E-04	4.53	-6.93E-04	6.93E-04	4.53	-0.00146	7.93E-04	4.53	-0.00169	8.97E-04	4.53	-0.00191	9.91E-04
4.531	-5.81E-04	5.81E-04	4.531	-6.92E-04	6.92E-04	4.531	-0.00146	7.93E-04	4.531	-0.00169	8.97E-04	4.531	-0.00191	9.91E-04
4.532	-5.81E-04	5.81E-04	4.532	-6.92E-04	6.92E-04	4.532	-0.00146	7.92E-04	4.532	-0.00169	8.96E-04	4.532	-0.00191	9.90E-04
4.533	-5.81E-04	5.81E-04	4.533	-6.92E-04	6.92E-04	4.533	-0.00146	7.92E-04	4.533	-0.00169	8.95E-04	4.533	-0.00191	9.90E-04
4.534	-5.80E-04	5.80E-04	4.534	-6.92E-04	6.92E-04	4.534	-0.00145	7.91E-04	4.534	-0.00169	8.95E-04	4.534	-0.00191	9.89E-04
4.535	-5.80E-04	5.80E-04	4.535	-6.91E-04	6.91E-04	4.535	-0.00145	7.91E-04	4.535	-0.00169	8.94E-04	4.535	-0.00191	9.88E-04
4.536	-5.80E-04	5.80E-04	4.536	-6.91E-04	6.91E-04	4.536	-0.00145	7.90E-04	4.536	-0.00169	8.94E-04	4.536	-0.00191	9.88E-04
4.537	-5.80E-04	5.80E-04	4.537	-6.91E-04	6.91E-04	4.537	-0.00145	7.90E-04	4.537	-0.00169	8.93E-04	4.537	-0.00191	9.87E-04
4.538	-5.79E-04	5.79E-04	4.538	-6.90E-04	6.90E-04	4.538	-0.00145	7.89E-04	4.538	-0.00169	8.93E-04	4.538	-0.0019	9.86E-04
4.539	-5.79E-04	5.79E-04	4.539	-6.90E-04	6.90E-04	4.539	-0.00145	7.89E-04	4.539	-0.00168	8.92E-04	4.539	-0.0019	9.86E-04
4.54	-5.79E-04	5.79E-04	4.54	-6.90E-04	6.90E-04	4.54	-0.00145	7.88E-04	4.54	-0.00168	8.91E-04	4.54	-0.0019	9.85E-04
4.541	-5.79E-04	5.79E-04	4.541	-6.89E-04	6.89E-04	4.541	-0.00145	7.88E-04	4.541	-0.00168	8.91E-04	4.541	-0.0019	9.84E-04
4.542	-5.78E-04	5.78E-04	4.542	-6.89E-04	6.89E-04	4.542	-0.00145	7.87E-04	4.542	-0.00168	8.90E-04	4.542	-0.0019	9.84E-04
4.543	-5.78E-04	5.78E-04	4.543	-6.89E-04	6.89E-04	4.543	-0.00145	7.87E-04	4.543	-0.00168	8.90E-04	4.543	-0.0019	9.83E-04
4.544	-5.78E-04	5.78E-04	4.544	-6.89E-04	6.89E-04	4.544	-0.00145	7.86E-04	4.544	-0.00168	8.89E-04	4.544	-0.0019	9.83E-04
4.545	-5.78E-04	5.78E-04	4.545	-6.88E-04	6.88E-04	4.545	-0.00144	7.86E-04	4.545	-0.00168	8.89E-04	4.545	-0.0019	9.82E-04
4.546	-5.77E-04	5.77E-04	4.546	-6.88E-04	6.88E-04	4.546	-0.00144	7.85E-04	4.546	-0.00168	8.88E-04	4.546	-0.0019	9.81E-04
4.547	-5.77E-04	5.77E-04	4.547	-6.88E-04	6.88E-04	4.547	-0.00144	7.85E-04	4.547	-0.00168	8.88E-04	4.547	-0.00189	9.81E-04
4.548	-5.77E-04	5.77E-04	4.548	-6.87E-04	6.87E-04	4.548	-0.00144	7.84E-04	4.548	-0.00168	8.87E-04	4.548	-0.00189	9.80E-04
4.549	-5.76E-04	5.76E-04	4.549	-6.87E-04	6.87E-04	4.549	-0.00144	7.84E-04	4.549	-0.00167	8.86E-04	4.549	-0.00189	9.79E-04
4.55	-5.76E-04	5.76E-04	4.55	-6.87E-04	6.87E-04	4.55	-0.00144	7.83E-04	4.55	-0.00167	8.86E-04	4.55	-0.00189	9.79E-04
4.551	-5.76E-04	5.76E-04	4.551	-6.86E-04	6.86E-04	4.551	-0.00144	7.83E-04	4.551	-0.00167	8.85E-04	4.551	-0.00189	9.78E-04
4.552	-5.76E-04	5.76E-04	4.552	-6.86E-04	6.86E-04	4.552	-0.00144	7.82E-04	4.552	-0.00167	8.85E-04	4.552	-0.00189	9.77E-04
4.553	-5.75E-04	5.75E-04	4.553	-6.86E-04	6.86E-04	4.553	-0.00144	7.82E-04	4.553	-0.00167	8.84E-04	4.553	-0.00189	9.77E-04
4.554	-5.75E-04	5.75E-04	4.554	-6.86E-04	6.86E-04	4.554	-0.00144	7.81E-04	4.554	-0.00167	8.84E-04	4.554	-0.00189	9.76E-04
4.555	-5.75E-04	5.75E-04	4.555	-6.85E-04	6.85E-04	4.555	-0.00144	7.81E-04	4.555	-0.00167	8.83E-04	4.555	-0.00188	9.76E-04
4.556	-5.75E-04	5.75E-04	4.556	-6.85E-04	6.85E-04	4.556	-0.00143	7.80E-04	4.556	-0.00167	8.82E-04	4.556	-0.00188	9.75E-04
4.557	-5.74E-04	5.74E-04	4.557	-6.85E-04	6.85E-04	4.557	-0.00143	7.80E-04	4.557	-0.00167	8.82E-04	4.557	-0.00188	9.74E-04
4.558	-5.74E-04	5.74E-04	4.558	-6.84E-04	6.84E-04	4.558	-0.00143	7.79E-04	4.558	-0.00166	8.81E-04	4.558	-0.00188	9.74E-04
4.559	-5.74E-04	5.74E-04	4.559	-6.84E-04	6.84E-04	4.559	-0.00143	7.79E-04	4.559	-0.00166	8.81E-04	4.559	-0.00188	9.73E-04
4.56	-5.74E-04	5.74E-04	4.56	-6.84E-04	6.84E-04	4.56	-0.00143	7.78E-04	4.56	-0.00166	8.80E-04	4.56	-0.00188	9.72E-04
4.561	-5.73E-04	5.73E-04	4.561	-6.83E-04	6.83E-04	4.561	-0.00143	7.78E-04	4.561	-0.00166	8.80E-04	4.561	-0.00188	9.72E-04
4.562	-5.73E-04	5.73E-04	4.562	-6.83E-04	6.83E-04	4.562	-0.00143	7.77E-04	4.562	-0.00166	8.79E-04	4.562	-0.00188	9.71E-04
4.563	-5.73E-04	5.73E-04	4.563	-6.83E-04	6.83E-04	4.563	-0.00143	7.77E-04	4.563	-0.00166	8.79E-04	4.563	-0.00187	9.71E-04
4.564	-5.73E-04	5.73E-04	4.564	-6.83E-04	6.83E-04	4.564	-0.00143	7.76E-04	4.564	-0.00166	8.78E-04	4.564	-0.00187	9.70E-04
4.565	-5.72E-04	5.72E-04	4.565	-6.82E-04	6.82E-04	4.565	-0.00143	7.76E-04	4.565	-0.00166	8.77E-04	4.565	-0.00187	9.69E-04
4.566	-5.72E-04	5.72E-04	4.566	-6.82E-04	6.82E-04	4.566	-0.00143	7.76E-04	4.566	-0.00166	8.77E-04	4.566	-0.00187	9.69E-04
4.567	-5.72E-04	5.72E-04	4.567	-6.82E-04	6.82E-04	4.567	-0.00142	7.75E-04	4.567	-0.00166	8.76E-04	4.567	-0.00187	9.68E-04
4.568	-5.72E-04	5.72E-04	4.568	-6.81E-04	6.81E-04	4.568	-0.00142	7.75E-04	4.568	-0.00165	8.76E-04	4.568	-0.00187	9.67E-04
4.569	-5.71E-04	5.71E-04	4.569	-6.81E-04	6.81E-04	4.569	-0.00142	7.74E-04	4.569	-0.00165	8.75E-04	4.569	-0.00187	9.67E-04
4.57	-5.71E-04	5.71E-04	4.57	-6.81E-04	6.81E-04	4.57	-0.00142	7.74E-04	4.57	-0.00165	8.75E-04	4.57	-0.00187	9.66E-04
4.571	-5.71E-04	5.71E-04	4.571	-6.80E-04	6.80E-04	4.571	-0.00142	7.73E-04	4.571	-0.00165	8.74E-04	4.571	-0.00186	9.66E-04
4.572	-5.71E-04	5.71E-04	4.572	-6.80E-04	6.80E-04	4.572	-0.00142	7.73E-04	4.572	-0.00165	8.74E-04	4.572	-0.00186	9.65E-04
4.573	-5.70E-04	5.70E-04	4.573	-6.80E-04	6.80E-04	4.573	-0.00142	7.72E-04	4.573	-0.00165	8.73E-04	4.573	-0.00186	9.64E-04
4.574	-5.70E-04	5.70E-04	4.574	-6.80E-04	6.80E-04	4.574	-0.00142	7.72E-04	4.574	-0.00165	8.72E-04	4.574	-0.00186	9.64E-04
4.575	-5.70E-04	5.70E-04	4.575	-6.79E-04	6.79E-04	4.575	-0.00142	7.71E-04	4.575	-0.00165	8.72E-04	4.575	-0.00186	9.63E-04
4.576	-5.70E-04	5.70E-04	4.576	-6.79E-04	6.79E-04	4.576	-0.00142	7.71E-04	4.576	-0.00165	8.71E-04	4.576	-0.00186	9.63E-04
4.577	-5.69E-04	5.69E-04	4.577	-6.79E-04	6.79E-04	4.577	-0.00142	7.70E-04	4.577	-0.00164	8.71E-04	4.577	-0.00186	9.62E-04
4.578	-5.69E-04	5.69E-04	4.578	-6.78E-04	6.78E-04	4.578	-0.00141	7.70E-04	4.578	-0.00164	8.70E-04	4.578	-0.00186	9.61E-04
4.579	-5.69E-04	5.69E-04	4.579	-6.78E-04	6.78E-04	4.579	-0.00141	7.69E-04	4.579	-0.00164	8.70E-04	4.579	-0.00186	9.61E-04
4.58	-5.69E-04	5.69E-04	4.58	-6.78E-04	6.78E-04	4.58	-0.00141	7.69E-04	4.58	-0.00164	8.69E-04	4.58	-0.00185	9.60E-04
4.581	-5.68E-04	5.68E-04	4.581	-6.77E-04	6.77E-04	4.581	-0.00141	7.68E-04	4.581	-0.00164	8.69E-04	4.581	-0.00185	9.59E-04
4.582	-5.68E-04	5.68E-04	4.582	-6.77E-04	6.77E-04	4.582	-0.00141	7.68E-04	4.582	-0.00164	8.68E-04	4.582	-0.00185	9.59E-04
4.583	-5.68E-04	5.68E-04	4.583	-6.77E-04	6.77E-04	4.583	-0.00141	7.67E-04	4.583	-0.00164	8.68E-04	4.583	-0.00185	9.58E-04
4.584	-5.68E-04	5.68E-04	4.584	-6.77E-04	6.77E-04	4.584	-0.00141	7.67E-04	4.584	-0.00164	8.67E-04	4.584	-0.00185	9.58E-04
4.585	-5.67E-04	5.67E-04	4.585	-6.76E-04	6.76E-04	4.585	-0.00141	7.66E-04	4.585	-0.00164	8.66E-04	4.585	-0.00185	9.57E-04
4.586	-5.67E-04	5.67E-04	4.586	-6.76E-04	6.76E-04	4.586	-0.00141	7.66E-04	4.586	-0.00164	8.66E-04	4.586	-0.00185	9.56E-04
4.587	-5.67E-04	5.67E-04	4.587	-6.76E-04	6.76E-04	4.587	-0.00141	7.65E-04	4.587	-0.00163	8.65E-04	4.587	-0.00185	9.56E-04
4.588	-5.67E-04	5.67E-04	4.588	-6.75E-04	6.75E-04	4.588	-0.00141	7.65E-04	4.588	-0.00163	8.65E-04	4.588	-0.00184	9.55E-04
4.589	-5.66E-04	5.66E-04	4.589	-6.75E-04	6.75E-04	4.589	-0.00141	7.65E-04	4.589	-0.00163	8.64E-04	4.589	-0.00184	9.55E-04
4.59	-5.66E-04	5.66E-04	4.59	-6.75E-04	6.75E-04	4.59	-0.0014	7.64E-04	4.59	-0.00163	8.64E-04	4.59	-0.00184	9.54E-04
4.591	-5.66E-04	5.66E-04	4.591	-6.74E-04	6.74E-04	4.591	-0.0014	7.64E-04	4.591	-0.00163	8.63E-04	4.591	-0.00184	9.53E-04
4.592	-5.66E-04	5.66E-04	4.592	-6.74E-04	6.74E-04	4.592	-0.0014	7.63E-04	4.592	-0.00163	8.63E-04	4.592	-0.00184	9.53E-04
4.593	-5.66E-04	5.66E-04	4.593	-6.74E-04	6.74E-04	4.593	-0.0014	7.63E-04	4.593	-0.00163	8.62E-04	4.593	-0.00184	9.52E-04
4.594	-5.65E-04	5.65E-04	4.594	-6.74E-04	6.74E-04	4.594	-0.0014	7.62E-04	4.594	-0.00163	8.62E-04	4.594	-0.00184	9.52E-04
4.595	-5.65E-04	5.65E-04	4.595	-6.73E-04	6.73E-04	4.595	-7.62E-04	7.62E-04	4.595	-0.00163	8.61E-04	4.595	-0.00184	9.51E-04
4.596	-5.65E-04	5.65E-04	4.596	-6.73E-04	6.73E-04	4.596	-7.61E-04	7.61E-04	4.596	-0.00163	8.60E-04	4.596	-0.00184	9.50E-04
4.597	-5.65E-04	5.65E-04	4.597	-6.73E-04	6.73E-04	4.597	-7.61E-04	7.61E-04	4.597	-0.00162	8.60E-04	4.597	-0.00183	9.50E-04
4.598	-5.64E-04	5.64E-04	4.598	-6.72E-04	6.72E-04	4.598	-7.61E-04	7.61E-04	4.598	-0.00162	8.59E-04	4.598	-0.00183	9.49E-04
4.599	-5.64E-04	5.64E-04	4.599	-6.72E-04	6.72E-04	4.599	-7.60E-04	7.60E-04	4.599	-0.00162	8.59E-04	4.599	-0.00183	9.48E-04
4.6	-5.64E-04	5.64E-04	4.6	-6.72E-04	6.72E-04	4.6	-7.60E-04	7.60E-04	4.6	-0.00162	8.58E-04	4.6	-0.00183	9.48E-04
4.601	-5.64E-04	5.64E-04	4.601	-6.72E-04	6.72E-04	4.601	-7.60E-04	7.60E-04	4.601	-0.00162	8.58E-04	4.601	-0.00183	9.47E-04
4.602	-5.63E-04	5.63E-04	4.602	-6.71E-04	6.71E-04	4.602	-7.60E-04	7.60E-04	4.602	-0.00162	8.57E-04	4.602	-0.00183	9.47E-04
4.603	-5.63E-04	5.63E-04	4.603	-6.71E-04	6.71E-04	4.603	-7.59E-04	7.59E-04	4.603	-0.00162	8.57E-04	4.603	-0.00183	9.46E-04
4.604	-5.63E-04	5.63E-04	4.604	-6.71E-04	6.71E-04	4.604	-7.59E-04	7.59E-04	4.604	-0.00162	8.56E-04	4.604	-0.00183	9.45E-04
4.605	-5.63E-04	5.63E-04	4.605	-6.70E-04	6.70E-04	4.605	-7.59E-04	7.59E-04	4.605	-0.00162	8.56E-04	4.605	-0.00182	9.45E-04
4.606	-5.62E-04	5.62E-04	4.606	-6.70E-04	6.70E-04	4.606	-7.58E-04	7.58E-04	4.606	-0.00162	8.55E-04	4.606	-0.00182	9.44E-04
4.607	-5.62E-04	5.62E-04	4.607	-6.70E-04	6.70E-04	4.607	-7.58E-04	7.58E-04	4.607	-0.00161	8.55E-04	4.607	-0.00182	9.44E-04
4.608	-5.62E-04	5.62E-04	4.608	-6.70E-04	6.70E-04	4.608	-7.58E-04	7.58E-04	4.608	-0.00161	8.54E-04	4.608	-0.00182	9.43E-04
4.609	-5.62E-04	5.62E-04	4.609	-6.69E-04	6.69E-04	4.609	-7.57E-04	7.57E-04	4.609	-0.00161	8.53E-04	4.609	-0.00182	9.42E-04
4.61	-5.61E-04	5.61E-04	4.61	-6.69E-04	6.69E-04	4.61	-7.57E-04	7.57E-04	4.61	-0.00161	8.53E-04	4.61	-0.00182	9.42E-04
4.611	-5.61E-04	5.61E-04	4.611	-6.69E-04	6.69E-04	4.611	-7.57E-04	7.57E-04	4.611	-0.00161	8.52E-04	4.611	-0.00182	9.41E-04
4.612	-5.61E-04	5.61E-04	4.612	-6.68E-04	6.68E-04	4.612	-7.56E-04	7.56E-04	4.612	-0.00161	8.52E-04	4.612	-0.00182	9.41E-04
4.613	-5.61E-04	5.61E-04	4.613	-6.68E-04	6.68E-04	4.613	-7.56E-04	7.56E-04	4.613	-0.00161	8.51E-04	4.613	-0.00182	9.40E-04
4.614	-5.60E-04	5.60E-04	4.614	-6.68E-04	6.68E-04	4.614	-7.56E-04	7.56E-04	4.614	-0.00161	8.51E-04	4.614	-0.00181	9.39E-04
4.615	-5.60E-04	5.60E-04	4.615	-6.67E-04	6.67E-04	4.615	-7.55E-04	7.55E-04	4.615	-0.00161	8.50E-04	4.615	-0.00181	9.39E-04
4.616	-5.60E-04	5.60E-04	4.616	-6.67E-04	6.67E-04	4.616	-7.55E-04	7.55E-04	4.616	-0.00161	8.50E-04	4.616	-0.00181	9.38E-04
4.617	-5.60E-04	5.60E-04	4.617	-6.67E-04	6.67E-04	4.617	-7.55E-04	7.55E-04	4.617	-0.0016	8.49E-04	4.617	-0.00181	9.38E-04
4.618	-5.59E-04	5.59E-04	4.618	-6.67E-04	6.67E-04	4.618	-7.54E-04	7.54E-04	4.618	-0.0016	8.49E-04	4.618	-0.00181	9.37E-04
4.619	-5.59E-04	5.59E-04	4.619	-6.66E-04	6.66E-04	4.619	-7.54E-04	7.54E-04	4.619	-0.0016	8.48E-04	4.619	-0.00181	9.37E-04
4.62	-5.59E-04	5.59E-04	4.62	-6.66E-04	6.66E-04	4.62	-7.54E-04	7.54E-04	4.62	-0.0016	8.48E-04	4.62	-0.00181	9.36E-04
4.621	-5.59E-04	5.59E-04	4.621	-6.66E-04	6.66E-04	4.621	-7.53E-04	7.53E-04	4.621	-0.0016	8.47E-04	4.621	-0.00181	9.35E-04
4.622	-5.58E-04	5.58E-04	4.622	-6.65E-04	6.65E-04	4.622	-7.53E-04	7.53E-04	4.622	-0.0016	8.47E-04	4.622	-0.00181	9.35E-04
4.623	-5.58E-04	5.58E-04	4.623	-6.65E-04	6.65E-04	4.623	-7.53E-04	7.53E-04	4.623	-0.0016	8.46E-04	4.623	-0.0018	9.34E-04
4.624	-5.58E-04	5.58E-04	4.624	-6.65E-04	6.65E-04	4.624	-7.52E-04	7.52E-04	4.624	-0.0016	8.45E-04	4.624	-0.0018	9.34E-04
4.625	-5.58E-04	5.58E-04	4.625	-6.65E-04	6.65E-04	4.625	-7.52E-04	7.52E-04	4.625	-0.0016	8.45E-04	4.625	-0.0018	9.33E-04
4.626	-5.57E-04	5.57E-04	4.626	-6.64E-04	6.64E-04	4.626	-7.52E-04	7.52E-04	4.626	-0.0016	8.44E-04	4.626	-0.0018	9.32E-04
4.627	-5.57E-04	5.57E-04	4.627	-6.64E-04	6.64E-04	4.627	-7.51E-04	7.51E-04	4.627	-0.00159	8.44E-04	4.627	-0.0018	9.32E-04
4.628	-5.57E-04	5.57E-04	4.628	-6.64E-04	6.64E-04	4.628	-7.51E-04	7.51E-04	4.628	-0.00159	8.43E-04	4.628	-0.0018	9.31E-04
4.629	-5.57E-04	5.57E-04	4.629	-6.63E-04	6.63E-04	4.629	-7.51E-04	7.51E-04	4.629	-0.00159	8.43E-04	4.629	-0.0018	9.31E-04
4.63	-5.57E-04	5.57E-04	4.63	-6.63E-04	6.63E-04	4.63	-7.50E-04	7.50E-04	4.63	-0.00159	8.42E-04	4.63	-0.0018	9.30E-04
4.631	-5.56E-04	5.56E-04	4.631	-6.63E-04	6.63E-04	4.631	-7.50E-04	7.50E-04	4.631	-0.00159	8.42E-04	4.631	-0.00179	9.29E-04
4.632	-5.56E-04	5.56E-04	4.632	-6.63E-04	6.63E-04	4.632	-7.50E-04	7.50E-04	4.632	-0.00159	8.41E-04	4.632	-0.00179	9.29E-04
4.633	-5.56E-04	5.56E-04	4.633	-6.62E-04	6.62E-04	4.633	-7.49E-04	7.49E-04	4.633	-0.00159	8.41E-04	4.633	-0.00179	9.28E-04
4.634	-5.56E-04	5.56E-04	4.634	-6.62E-04	6.62E-04	4.634	-7.49E-04	7.49E-04	4.634	-0.00159	8.40E-04	4.634	-0.00179	9.28E-04
4.635	-5.55E-04	5.55E-04	4.635	-6.62E-04	6.62E-04	4.635	-7.49E-04	7.49E-04	4.635	-0.00159	8.40E-04	4.635	-0.00179	9.27E-04
4.636	-5.55E-04	5.55E-04	4.636	-6.61E-04	6.61E-04	4.636	-7.48E-04	7.48E-04	4.636	-0.00159	8.39E-04	4.636	-0.00179	9.26E-04
4.637	-5.55E-04	5.55E-04	4.637	-6.61E-04	6.61E-04	4.637	-7.48E-04	7.48E-04	4.637	-0.00158	8.39E-04	4.637	-0.00179	9.26E-04
4.638	-5.55E-04	5.55E-04	4.638	-6.61E-04	6.61E-04	4.638	-7.48E-04	7.48E-04	4.638	-0.00158	8.38E-04	4.638	-0.00179	9.25E-04
4.639	-5.54E-04	5.54E-04	4.639	-6.61E-04	6.61E-04	4.639	-7.47E-04	7.47E-04	4.639	-0.00158	8.38E-04	4.639	-0.00179	9.25E-04
4.64	-5.54E-04	5.54E-04	4.64	-6.60E-04	6.60E-04	4.64	-7.47E-04	7.47E-04	4.64	-0.00158	8.37E-04	4.64	-0.00178	9.24E-04
4.641	-5.54E-04	5.54E-04	4.641	-6.60E-04	6.60E-04	4.641	-7.47E-04	7.47E-04	4.641	-0.00158	8.37E-04	4.641	-0.00178	9.24E-04
4.642	-5.54E-04	5.54E-04	4.642	-6.60E-04	6.60E-04	4.642	-7.46E-04	7.46E-04	4.642	-0.00158	8.36E-04	4.642	-0.00178	9.23E-04
4.643	-5.53E-04	5.53E-04	4.643	-6.59E-04	6.59E-04	4.643	-7.46E-04	7.46E-04	4.643	-0.00158	8.36E-04	4.643	-0.00178	9.22E-04
4.644	-5.53E-04	5.53E-04	4.644	-6.59E-04	6.59E-04	4.644	-7.46E-04	7.46E-04	4.644	-0.00158	8.35E-04	4.644	-0.00178	9.22E-04
4.645	-5.53E-04	5.53E-04	4.645	-6.59E-04	6.59E-04	4.645	-7.46E-04	7.46E-04	4.645	-0.00158	8.34E-04	4.645	-0.00178	9.21E-04
4.646	-5.53E-04	5.53E-04	4.646	-6.59E-04	6.59E-04	4.646	-7.45E-04	7.45E-04	4.646	-0.00158	8.34E-04	4.646	-0.00178	9.21E-04
4.647	-5.52E-04	5.52E-04	4.647	-6.58E-04	6.58E-04	4.647	-7.45E-04	7.45E-04	4.647	-0.00157	8.33E-04	4.647	-0.00178	9.20E-04
4.648	-5.52E-04	5.52E-04	4.648	-6.58E-04	6.58E-04	4.648	-7.45E-04	7.45E-04	4.648	-0.00157	8.33E-04	4.648	-0.00178	9.19E-04
4.649	-5.52E-04	5.52E-04	4.649	-6.58E-04	6.58E-04	4.649	-7.44E-04	7.44E-04	4.649	-0.00157	8.32E-04	4.649	-0.00177	9.19E-04
4.65	-5.52E-04	5.52E-04	4.65	-6.57E-04	6.57E-04	4.65	-7.44E-04	7.44E-04	4.65	-0.00157	8.32E-04	4.65	-0.00177	9.18E-04
4.651	-5.51E-04	5.51E-04	4.651	-6.57E-04	6.57E-04	4.651	-7.44E-04	7.44E-04	4.651	-0.00157	8.31E-04	4.651	-0.00177	9.18E-04
4.652	-5.51E-04	5.51E-04	4.652	-6.57E-04	6.57E-04	4.652	-7.43E-04	7.43E-04	4.652	-0.00157	8.31E-04	4.652	-0.00177	9.17E-04
4.653	-5.51E-04	5.51E-04	4.653	-6.57E-04	6.57E-04	4.653	-7.43E-04	7.43E-04	4.653	-0.00157	8.30E-04	4.653	-0.00177	9.17E-04
4.654	-5.51E-04	5.51E-04	4.654	-6.56E-04	6.56E-04	4.654	-7.43E-04	7.43E-04	4.654	-0.00157	8.30E-04	4.654	-0.00177	9.16E-04
4.655	-5.51E-04	5.51E-04	4.655	-6.56E-04	6.56E-04	4.655	-7.42E-04	7.42E-04	4.655	-0.00157	8.29E-04	4.655	-0.00177	9.15E-04
4.656	-5.50E-04	5.50E-04	4.656	-6.56E-04	6.56E-04	4.656	-7.42E-04	7.42E-04	4.656	-0.00157	8.29E-04	4.656	-0.00177	9.15E-04
4.657	-5.50E-04	5.50E-04	4.657	-6.56E-04	6.56E-04	4.657	-7.42E-04	7.42E-04	4.657	-0.00156	8.28E-04	4.657	-0.00177	9.14E-04
4.658	-5.50E-04	5.50E-04	4.658	-6.55E-04	6.55E-04	4.658	-7.41E-04	7.41E-04	4.658	-0.00156	8.28E-04	4.658	-0.00176	9.14E-04
4.659	-5.50E-04	5.50E-04	4.659	-6.55E-04	6.55E-04	4.659	-7.41E-04	7.41E-04	4.659	-0.00156	8.27E-04	4.659	-0.00176	9.13E-04
4.66	-5.49E-04	5.49E-04	4.66	-6.55E-04	6.55E-04	4.66	-7.41E-04	7.41E-04	4.66	-0.00156	8.27E-04	4.66	-0.00176	9.13E-04
4.661	-5.49E-04	5.49E-04	4.661	-6.54E-04	6.54E-04	4.661	-7.40E-04	7.40E-04	4.661	-0.00156	8.26E-04	4.661	-0.00176	9.12E-04
4.662	-5.49E-04	5.49E-04	4.662	-6.54E-04	6.54E-04	4.662	-7.40E-04	7.40E-04	4.662	-0.00156	8.26E-04	4.662	-0.00176	9.11E-04

4.663	-5.49E-04	5.49E-04	4.663	-6.54E-04	6.54E-04	4.663	-7.40E-04	7.40E-04	4.663	-0.00156	8.25E-04	4.663	-0.00176	9.11E-04
4.664	-5.48E-04	5.48E-04	4.664	-6.54E-04	6.54E-04	4.664	-7.39E-04	7.39E-04	4.664	-0.00156	8.25E-04	4.664	-0.00176	9.10E-04
4.665	-5.48E-04	5.48E-04	4.665	-6.53E-04	6.53E-04	4.665	-7.39E-04	7.39E-04	4.665	-0.00156	8.24E-04	4.665	-0.00176	9.10E-04
4.666	-5.48E-04	5.48E-04	4.666	-6.53E-04	6.53E-04	4.666	-7.39E-04	7.39E-04	4.666	-0.00156	8.24E-04	4.666	-0.00176	9.09E-04
4.667	-5.48E-04	5.48E-04	4.667	-6.53E-04	6.53E-04	4.667	-7.38E-04	7.38E-04	4.667	-0.00155	8.23E-04	4.667	-0.00175	9.09E-04
4.668	-5.47E-04	5.47E-04	4.668	-6.52E-04	6.52E-04	4.668	-7.38E-04	7.38E-04	4.668	-0.00155	8.23E-04	4.668	-0.00175	9.08E-04
4.669	-5.47E-04	5.47E-04	4.669	-6.52E-04	6.52E-04	4.669	-7.38E-04	7.38E-04	4.669	-0.00155	8.22E-04	4.669	-0.00175	9.07E-04
4.67	-5.47E-04	5.47E-04	4.67	-6.52E-04	6.52E-04	4.67	-7.38E-04	7.38E-04	4.67	-0.00155	8.22E-04	4.67	-0.00175	9.07E-04
4.671	-5.47E-04	5.47E-04	4.671	-6.52E-04	6.52E-04	4.671	-7.37E-04	7.37E-04	4.671	-0.00155	8.21E-04	4.671	-0.00175	9.06E-04
4.672	-5.47E-04	5.47E-04	4.672	-6.51E-04	6.51E-04	4.672	-7.37E-04	7.37E-04	4.672	-0.00155	8.21E-04	4.672	-0.00175	9.06E-04
4.673	-5.46E-04	5.46E-04	4.673	-6.51E-04	6.51E-04	4.673	-7.37E-04	7.37E-04	4.673	-0.00155	8.20E-04	4.673	-0.00175	9.05E-04
4.674	-5.46E-04	5.46E-04	4.674	-6.51E-04	6.51E-04	4.674	-7.36E-04	7.36E-04	4.674	-0.00155	8.20E-04	4.674	-0.00175	9.05E-04
4.675	-5.46E-04	5.46E-04	4.675	-6.50E-04	6.50E-04	4.675	-7.36E-04	7.36E-04	4.675	-0.00155	8.19E-04	4.675	-0.00175	9.04E-04
4.676	-5.46E-04	5.46E-04	4.676	-6.50E-04	6.50E-04	4.676	-7.36E-04	7.36E-04	4.676	-0.00155	8.19E-04	4.676	-0.00174	9.03E-04
4.677	-5.45E-04	5.45E-04	4.677	-6.50E-04	6.50E-04	4.677	-7.35E-04	7.35E-04	4.677	-0.00155	8.18E-04	4.677	-0.00174	9.03E-04
4.678	-5.45E-04	5.45E-04	4.678	-6.50E-04	6.50E-04	4.678	-7.35E-04	7.35E-04	4.678	-0.00154	8.18E-04	4.678	-0.00174	9.02E-04
4.679	-5.45E-04	5.45E-04	4.679	-6.49E-04	6.49E-04	4.679	-7.35E-04	7.35E-04	4.679	-0.00154	8.17E-04	4.679	-0.00174	9.02E-04
4.68	-5.45E-04	5.45E-04	4.68	-6.49E-04	6.49E-04	4.68	-7.34E-04	7.34E-04	4.68	-0.00154	8.17E-04	4.68	-0.00174	9.01E-04
4.681	-5.44E-04	5.44E-04	4.681	-6.49E-04	6.49E-04	4.681	-7.34E-04	7.34E-04	4.681	-0.00154	8.16E-04	4.681	-0.00174	9.01E-04
4.682	-5.44E-04	5.44E-04	4.682	-6.49E-04	6.49E-04	4.682	-7.34E-04	7.34E-04	4.682	-0.00154	8.16E-04	4.682	-0.00174	9.00E-04
4.683	-5.44E-04	5.44E-04	4.683	-6.48E-04	6.48E-04	4.683	-7.33E-04	7.33E-04	4.683	-0.00154	8.15E-04	4.683	-0.00174	8.99E-04
4.684	-5.44E-04	5.44E-04	4.684	-6.48E-04	6.48E-04	4.684	-7.33E-04	7.33E-04	4.684	-0.00154	8.15E-04	4.684	-0.00174	8.99E-04
4.685	-5.44E-04	5.44E-04	4.685	-6.48E-04	6.48E-04	4.685	-7.33E-04	7.33E-04	4.685	-0.00154	8.14E-04	4.685	-0.00173	8.98E-04
4.686	-5.43E-04	5.43E-04	4.686	-6.47E-04	6.47E-04	4.686	-7.33E-04	7.33E-04	4.686	-0.00154	8.14E-04	4.686	-0.00173	8.98E-04
4.687	-5.43E-04	5.43E-04	4.687	-6.47E-04	6.47E-04	4.687	-7.32E-04	7.32E-04	4.687	-0.00154	8.13E-04	4.687	-0.00173	8.97E-04
4.688	-5.43E-04	5.43E-04	4.688	-6.47E-04	6.47E-04	4.688	-7.32E-04	7.32E-04	4.688	-0.00153	8.13E-04	4.688	-0.00173	8.97E-04
4.689	-5.43E-04	5.43E-04	4.689	-6.47E-04	6.47E-04	4.689	-7.32E-04	7.32E-04	4.689	-0.00153	8.12E-04	4.689	-0.00173	8.96E-04
4.69	-5.42E-04	5.42E-04	4.69	-6.46E-04	6.46E-04	4.69	-7.31E-04	7.31E-04	4.69	-0.00153	8.12E-04	4.69	-0.00173	8.96E-04
4.691	-5.42E-04	5.42E-04	4.691	-6.46E-04	6.46E-04	4.691	-7.31E-04	7.31E-04	4.691	-0.00153	8.11E-04	4.691	-0.00173	8.95E-04
4.692	-5.42E-04	5.42E-04	4.692	-6.46E-04	6.46E-04	4.692	-7.31E-04	7.31E-04	4.692	-0.00153	8.11E-04	4.692	-0.00173	8.94E-04
4.693	-5.42E-04	5.42E-04	4.693	-6.45E-04	6.45E-04	4.693	-7.30E-04	7.30E-04	4.693	-0.00153	8.10E-04	4.693	-0.00173	8.94E-04
4.694	-5.41E-04	5.41E-04	4.694	-6.45E-04	6.45E-04	4.694	-7.30E-04	7.30E-04	4.694	-0.00153	8.10E-04	4.694	-0.00173	8.93E-04
4.695	-5.41E-04	5.41E-04	4.695	-6.45E-04	6.45E-04	4.695	-7.30E-04	7.30E-04	4.695	-0.00153	8.09E-04	4.695	-0.00172	8.93E-04
4.696	-5.41E-04	5.41E-04	4.696	-6.45E-04	6.45E-04	4.696	-7.29E-04	7.29E-04	4.696	-0.00153	8.09E-04	4.696	-0.00172	8.92E-04
4.697	-5.41E-04	5.41E-04	4.697	-6.44E-04	6.44E-04	4.697	-7.29E-04	7.29E-04	4.697	-0.00153	8.08E-04	4.697	-0.00172	8.92E-04
4.698	-5.41E-04	5.41E-04	4.698	-6.44E-04	6.44E-04	4.698	-7.29E-04	7.29E-04	4.698	-0.00153	8.08E-04	4.698	-0.00172	8.91E-04
4.699	-5.40E-04	5.40E-04	4.699	-6.44E-04	6.44E-04	4.699	-7.28E-04	7.28E-04	4.699	-0.00152	8.07E-04	4.699	-0.00172	8.91E-04
4.7	-5.40E-04	5.40E-04	4.7	-6.44E-04	6.44E-04	4.7	-7.28E-04	7.28E-04	4.7	-0.00152	8.07E-04	4.7	-0.00172	8.90E-04
4.701	-5.40E-04	5.40E-04	4.701	-6.43E-04	6.43E-04	4.701	-7.28E-04	7.28E-04	4.701	-0.00152	8.06E-04	4.701	-0.00172	8.89E-04
4.702	-5.40E-04	5.40E-04	4.702	-6.43E-04	6.43E-04	4.702	-7.28E-04	7.28E-04	4.702	-0.00152	8.06E-04	4.702	-0.00172	8.89E-04
4.703	-5.39E-04	5.39E-04	4.703	-6.43E-04	6.43E-04	4.703	-7.27E-04	7.27E-04	4.703	-0.00152	8.05E-04	4.703	-0.00172	8.88E-04
4.704	-5.39E-04	5.39E-04	4.704	-6.42E-04	6.42E-04	4.704	-7.27E-04	7.27E-04	4.704	-0.00152	8.05E-04	4.704	-0.00171	8.88E-04
4.705	-5.39E-04	5.39E-04	4.705	-6.42E-04	6.42E-04	4.705	-7.27E-04	7.27E-04	4.705	-0.00152	8.04E-04	4.705	-0.00171	8.87E-04
4.706	-5.39E-04	5.39E-04	4.706	-6.42E-04	6.42E-04	4.706	-7.26E-04	7.26E-04	4.706	-0.00152	8.04E-04	4.706	-0.00171	8.87E-04
4.707	-5.38E-04	5.38E-04	4.707	-6.42E-04	6.42E-04	4.707	-7.26E-04	7.26E-04	4.707	-0.00152	8.03E-04	4.707	-0.00171	8.86E-04
4.708	-5.38E-04	5.38E-04	4.708	-6.41E-04	6.41E-04	4.708	-7.26E-04	7.26E-04	4.708	-0.00152	8.03E-04	4.708	-0.00171	8.86E-04
4.709	-5.38E-04	5.38E-04	4.709	-6.41E-04	6.41E-04	4.709	-7.25E-04	7.25E-04	4.709	-0.00152	8.02E-04	4.709	-0.00171	8.85E-04
4.71	-5.38E-04	5.38E-04	4.71	-6.41E-04	6.41E-04	4.71	-7.25E-04	7.25E-04	4.71	-0.00151	8.02E-04	4.71	-0.00171	8.84E-04
4.711	-5.38E-04	5.38E-04	4.711	-6.41E-04	6.41E-04	4.711	-7.25E-04	7.25E-04	4.711	-0.00151	8.01E-04	4.711	-0.00171	8.84E-04
4.712	-5.37E-04	5.37E-04	4.712	-6.40E-04	6.40E-04	4.712	-7.24E-04	7.24E-04	4.712	-0.00151	8.01E-04	4.712	-0.00171	8.83E-04
4.713	-5.37E-04	5.37E-04	4.713	-6.40E-04	6.40E-04	4.713	-7.24E-04	7.24E-04	4.713	-0.00151	8.00E-04	4.713	-0.0017	8.83E-04
4.714	-5.37E-04	5.37E-04	4.714	-6.40E-04	6.40E-04	4.714	-7.24E-04	7.24E-04	4.714	-0.00151	8.00E-04	4.714	-0.0017	8.82E-04
4.715	-5.37E-04	5.37E-04	4.715	-6.39E-04	6.39E-04	4.715	-7.24E-04	7.24E-04	4.715	-0.00151	7.99E-04	4.715	-0.0017	8.82E-04
4.716	-5.36E-04	5.36E-04	4.716	-6.39E-04	6.39E-04	4.716	-7.23E-04	7.23E-04	4.716	-0.00151	7.99E-04	4.716	-0.0017	8.81E-04
4.717	-5.36E-04	5.36E-04	4.717	-6.39E-04	6.39E-04	4.717	-7.23E-04	7.23E-04	4.717	-0.00151	7.98E-04	4.717	-0.0017	8.81E-04
4.718	-5.36E-04	5.36E-04	4.718	-6.39E-04	6.39E-04	4.718	-7.23E-04	7.23E-04	4.718	-0.00151	7.98E-04	4.718	-0.0017	8.80E-04
4.719	-5.36E-04	5.36E-04	4.719	-6.38E-04	6.38E-04	4.719	-7.22E-04	7.22E-04	4.719	-0.00151	7.97E-04	4.719	-0.0017	8.80E-04
4.72	-5.35E-04	5.35E-04	4.72	-6.38E-04	6.38E-04	4.72	-7.22E-04	7.22E-04	4.72	-0.0015	7.97E-04	4.72	-0.0017	8.79E-04
4.721	-5.35E-04	5.35E-04	4.721	-6.38E-04	6.38E-04	4.721	-7.22E-04	7.22E-04	4.721	-0.0015	7.96E-04	4.721	-0.0017	8.78E-04
4.722	-5.35E-04	5.35E-04	4.722	-6.38E-04	6.38E-04	4.722	-7.21E-04	7.21E-04	4.722	-0.0015	7.96E-04	4.722	-0.0017	8.78E-04
4.723	-5.35E-04	5.35E-04	4.723	-6.37E-04	6.37E-04	4.723	-7.21E-04	7.21E-04	4.723	-0.0015	7.95E-04	4.723	-0.00169	8.77E-04
4.724	-5.35E-04	5.35E-04	4.724	-6.37E-04	6.37E-04	4.724	-7.21E-04	7.21E-04	4.724	-0.0015	7.95E-04	4.724	-0.00169	8.77E-04
4.725	-5.34E-04	5.34E-04	4.725	-6.37E-04	6.37E-04	4.725	-7.20E-04	7.20E-04	4.725	-0.0015	7.94E-04	4.725	-0.00169	8.76E-04
4.726	-5.34E-04	5.34E-04	4.726	-6.37E-04	6.37E-04	4.726	-7.20E-04	7.20E-04	4.726	-0.0015	7.94E-04	4.726	-0.00169	8.76E-04
4.727	-5.34E-04	5.34E-04	4.727	-6.36E-04	6.36E-04	4.727	-7.20E-04	7.20E-04	4.727	-0.0015	7.93E-04	4.727	-0.00169	8.75E-04
4.728	-5.34E-04	5.34E-04	4.728	-6.36E-04	6.36E-04	4.728	-7.20E-04	7.20E-04	4.728	-0.0015	7.93E-04	4.728	-0.00169	8.75E-04
4.729	-5.33E-04	5.33E-04	4.729	-6.36E-04	6.36E-04	4.729	-7.19E-04	7.19E-04	4.729	-0.0015	7.92E-04	4.729	-0.00169	8.74E-04
4.73	-5.33E-04	5.33E-04	4.73	-6.35E-04	6.35E-04	4.73	-7.19E-04	7.19E-04	4.73	-0.0015	7.92E-04	4.73	-0.00169	8.74E-04
4.731	-5.33E-04	5.33E-04	4.731	-6.35E-04	6.35E-04	4.731	-7.19E-04	7.19E-04	4.731	-0.00149	7.91E-04	4.731	-0.00169	8.73E-04
4.732	-5.33E-04	5.33E-04	4.732	-6.35E-04	6.35E-04	4.732	-7.18E-04	7.18E-04	4.732	-0.00149	7.91E-04	4.732	-0.00168	8.72E-04
4.733	-5.33E-04	5.33E-04	4.733	-6.35E-04	6.35E-04	4.733	-7.18E-04	7.18E-04	4.733	-0.00149	7.90E-04	4.733	-0.00168	8.72E-04
4.734	-5.32E-04	5.32E-04	4.734	-6.34E-04	6.34E-04	4.734	-7.18E-04	7.18E-04	4.734	-0.00149	7.90E-04	4.734	-0.00168	8.71E-04
4.735	-5.32E-04	5.32E-04	4.735	-6.34E-04	6.34E-04	4.735	-7.17E-04	7.17E-04	4.735	-0.00149	7.89E-04	4.735	-0.00168	8.71E-04
4.736	-5.32E-04	5.32E-04	4.736	-6.34E-04	6.34E-04	4.736	-7.17E-04	7.17E-04	4.736	-0.00149	7.89E-04	4.736	-0.00168	8.70E-04
4.737	-5.32E-04	5.32E-04	4.737	-6.34E-04	6.34E-04	4.737	-7.17E-04	7.17E-04	4.737	-0.00149	7.89E-04	4.737	-0.00168	8.70E-04
4.738	-5.31E-04	5.31E-04	4.738	-6.33E-04	6.33E-04	4.738	-7.17E-04	7.17E-04	4.738	-0.00149	7.88E-04	4.738	-0.00168	8.69E-04
4.739	-5.31E-04	5.31E-04	4.739	-6.33E-04	6.33E-04	4.739	-7.16E-04	7.16E-04	4.739	-0.00149	7.88E-04	4.739	-0.00168	8.69E-04
4.74	-5.31E-04	5.31E-04	4.74	-6.33E-04	6.33E-04	4.74	-7.16E-04	7.16E-04	4.74	-0.00149	7.87E-04	4.74	-0.00168	8.68E-04
4.741	-5.31E-04	5.31E-04	4.741	-6.32E-04	6.32E-04	4.741	-7.16E-04	7.16E-04	4.741	-0.00149	7.87E-04	4.741	-0.00168	8.68E-04
4.742	-5.31E-04	5.31E-04	4.742	-6.32E-04	6.32E-04	4.742	-7.15E-04	7.15E-04	4.742	-0.00148	7.86E-04	4.742	-0.00167	8.67E-04
4.743	-5.30E-04	5.30E-04	4.743	-6.32E-04	6.32E-04	4.743	-7.15E-04	7.15E-04	4.743	-0.00148	7.86E-04	4.743	-0.00167	8.67E-04
4.744	-5.30E-04	5.30E-04	4.744	-6.32E-04	6.32E-04	4.744	-7.15E-04	7.15E-04	4.744	-0.00148	7.85E-04	4.744	-0.00167	8.66E-04
4.745	-5.30E-04	5.30E-04	4.745	-6.31E-04	6.31E-04	4.745	-7.14E-04	7.14E-04	4.745	-0.00148	7.85E-04	4.745	-0.00167	8.65E-04
4.746	-5.30E-04	5.30E-04	4.746	-6.31E-04	6.31E-04	4.746	-7.14E-04	7.14E-04	4.746	-0.00148	7.84E-04	4.746	-0.00167	8.65E-04
4.747	-5.29E-04	5.29E-04	4.747	-6.31E-04	6.31E-04	4.747	-7.14E-04	7.14E-04	4.747	-0.00148	7.84E-04	4.747	-0.00167	8.64E-04
4.748	-5.29E-04	5.29E-04	4.748	-6.31E-04	6.31E-04	4.748	-7.14E-04	7.14E-04	4.748	-0.00148	7.83E-04	4.748	-0.00167	8.64E-04
4.749	-5.29E-04	5.29E-04	4.749	-6.30E-04	6.30E-04	4.749	-7.13E-04	7.13E-04	4.749	-0.00148	7.83E-04	4.749	-0.00167	8.63E-04
4.75	-5.29E-04	5.29E-04	4.75	-6.30E-04	6.30E-04	4.75	-7.13E-04	7.13E-04	4.75	-7.82E-04	7.82E-04	4.75	-0.00167	8.63E-04
4.751	-5.29E-04	5.29E-04	4.751	-6.30E-04	6.30E-04	4.751	-7.13E-04	7.13E-04	4.751	-7.82E-04	7.82E-04	4.751	-0.00167	8.62E-04
4.752	-5.28E-04	5.28E-04	4.752	-6.30E-04	6.30E-04	4.752	-7.12E-04	7.12E-04	4.752	-7.82E-04	7.82E-04	4.752	-0.00166	8.62E-04
4.753	-5.28E-04	5.28E-04	4.753	-6.29E-04	6.29E-04	4.753	-7.12E-04	7.12E-04	4.753	-7.81E-04	7.81E-04	4.753	-0.00166	8.61E-04
4.754	-5.28E-04	5.28E-04	4.754	-6.29E-04	6.29E-04	4.754	-7.12E-04	7.12E-04	4.754	-7.81E-04	7.81E-04	4.754	-0.00166	8.61E-04
4.755	-5.28E-04	5.28E-04	4.755	-6.29E-04	6.29E-04	4.755	-7.11E-04	7.11E-04	4.755	-7.81E-04	7.81E-04	4.755	-0.00166	8.60E-04
4.756	-5.27E-04	5.27E-04	4.756	-6.29E-04	6.29E-04	4.756	-7.11E-04	7.11E-04	4.756	-7.80E-04	7.80E-04	4.756	-0.00166	8.60E-04
4.757	-5.27E-04	5.27E-04	4.757	-6.28E-04	6.28E-04	4.757	-7.11E-04	7.11E-04	4.757	-7.80E-04	7.80E-04	4.757	-0.00166	8.59E-04
4.758	-5.27E-04	5.27E-04	4.758	-6.28E-04	6.28E-04	4.758	-7.11E-04	7.11E-04	4.758	-7.80E-04	7.80E-04	4.758	-0.00166	8.59E-04
4.759	-5.27E-04	5.27E-04	4.759	-6.28E-04	6.28E-04	4.759	-7.10E-04	7.10E-04	4.759	-7.79E-04	7.79E-04	4.759	-0.00166	8.58E-04
4.76	-5.27E-04	5.27E-04	4.76	-6.27E-04	6.27E-04	4.76	-7.10E-04	7.10E-04	4.76	-7.79E-04	7.79E-04	4.76	-0.00166	8.57E-04
4.761	-5.26E-04	5.26E-04	4.761	-6.27E-04	6.27E-04	4.761	-7.10E-04	7.10E-04	4.761	-7.79E-04	7.79E-04	4.761	-0.00165	8.57E-04
4.762	-5.26E-04	5.26E-04	4.762	-6.27E-04	6.27E-04	4.762	-7.09E-04	7.09E-04	4.762	-7.78E-04	7.78E-04	4.762	-0.00165	8.56E-04
4.763	-5.26E-04	5.26E-04	4.763	-6.27E-04	6.27E-04	4.763	-7.09E-04	7.09E-04	4.763	-7.78E-04	7.78E-04	4.763	-0.00165	8.56E-04
4.764	-5.26E-04	5.26E-04	4.764	-6.26E-04	6.26E-04	4.764	-7.09E-04	7.09E-04	4.764	-7.78E-04	7.78E-04	4.764	-0.00165	8.55E-04
4.765	-5.25E-04	5.25E-04	4.765	-6.26E-04	6.26E-04	4.765	-7.08E-04	7.08E-04	4.765	-7.77E-04	7.77E-04	4.765	-0.00165	8.55E-04
4.766	-5.25E-04	5.25E-04	4.766	-6.26E-04	6.26E-04	4.766	-7.08E-04	7.08E-04	4.766	-7.77E-04	7.77E-04	4.766	-0.00165	8.54E-04
4.767	-5.25E-04	5.25E-04	4.767	-6.26E-04	6.26E-04	4.767	-7.08E-04	7.08E-04	4.767	-7.77E-04	7.77E-04	4.767	-0.00165	8.54E-04
4.768	-5.25E-04	5.25E-04	4.768	-6.25E-04	6.25E-04	4.768	-7.08E-04	7.08E-04	4.768	-7.76E-04	7.76E-04	4.768	-0.00165	8.53E-04
4.769	-5.25E-04	5.25E-04	4.769	-6.25E-04	6.25E-04	4.769	-7.07E-04	7.07E-04	4.769	-7.76E-04	7.76E-04	4.769	-0.00165	8.53E-04
4.77	-5.24E-04	5.24E-04	4.77	-6.25E-04	6.25E-04	4.77	-7.07E-04	7.07E-04	4.77	-7.76E-04	7.76E-04	4.77	-0.00165	8.52E-04
4.771	-5.24E-04	5.24E-04	4.771	-6.25E-04	6.25E-04	4.771	-7.07E-04	7.07E-04	4.771	-7.75E-04	7.75E-04	4.771	-0.00164	8.52E-04
4.772	-5.24E-04	5.24E-04	4.772	-6.24E-04	6.24E-04	4.772	-7.06E-04	7.06E-04	4.772	-7.75E-04	7.75E-04	4.772	-0.00164	8.51E-04
4.773	-5.24E-04	5.24E-04	4.773	-6.24E-04	6.24E-04	4.773	-7.06E-04	7.06E-04	4.773	-7.75E-04	7.75E-04	4.773	-0.00164	8.51E-04
4.774	-5.23E-04	5.23E-04	4.774	-6.24E-04	6.24E-04	4.774	-7.06E-04	7.06E-04	4.774	-7.74E-04	7.74E-04	4.774	-0.00164	8.50E-04
4.775	-5.23E-04	5.23E-04	4.775	-6.24E-04	6.24E-04	4.775	-7.05E-04	7.05E-04	4.775	-7.74E-04	7.74E-04	4.775	-0.00164	8.50E-04
4.776	-5.23E-04	5.23E-04	4.776	-6.23E-04	6.23E-04	4.776	-7.05E-04	7.05E-04	4.776	-7.74E-04	7.74E-04	4.776	-0.00164	8.49E-04
4.777	-5.23E-04	5.23E-04	4.777	-6.23E-04	6.23E-04	4.777	-7.05E-04	7.05E-04	4.777	-7.74E-04	7.74E-04	4.777	-0.00164	8.49E-04
4.778	-5.23E-04	5.23E-04	4.778	-6.23E-04	6.23E-04	4.778	-7.05E-04	7.05E-04	4.778	-7.73E-04	7.73E-04	4.778	-0.00164	8.48E-04
4.779	-5.22E-04	5.22E-04	4.779	-6.22E-04	6.22E-04	4.779	-7.04E-04	7.04E-04	4.779	-7.73E-04	7.73E-04	4.779	-0.00164	8.48E-04
4.78	-5.22E-04	5.22E-04	4.78	-6.22E-04	6.22E-04	4.78	-7.04E-04	7.04E-04	4.78	-7.73E-04	7.73E-04	4.78	-0.00164	8.47E-04
4.781	-5.22E-04	5.22E-04	4.781	-6.22E-04	6.22E-04	4.781	-7.04E-04	7.04E-04	4.781	-7.72E-04	7.72E-04	4.781	-0.00163	8.46E-04
4.782	-5.22E-04	5.22E-04	4.782	-6.22E-04	6.22E-04	4.782	-7.03E-04	7.03E-04	4.782	-7.72E-04	7.72E-04	4.782	-0.00163	8.46E-04
4.783	-5.21E-04	5.21E-04	4.783	-6.21E-04	6.21E-04	4.783	-7.03E-04	7.03E-04	4.783	-7.72E-04	7.72E-04	4.783	-0.00163	8.45E-04
4.784	-5.21E-04	5.21E-04	4.784	-6.21E-04	6.21E-04	4.784	-7.03E-04	7.03E-04	4.784	-7.71E-04	7.71E-04	4.784	-0.00163	8.45E-04
4.785	-5.21E-04	5.21E-04	4.785	-6.21E-04	6.21E-04	4.785	-7.03E-04	7.03E-04	4.785	-7.71E-04	7.71E-04	4.785	-0.00163	8.44E-04
4.786	-5.21E-04	5.21E-04	4.786	-6.21E-04	6.21E-04	4.786	-7.02E-04	7.02E-04	4.786	-7.71E-04	7.71E-04	4.786	-0.00163	8.44E-04
4.787	-5.21E-04	5.21E-04	4.787	-6.20E-04	6.20E-04	4.787	-7.02E-04	7.02E-04	4.787	-7.70E-04	7.70E-04	4.787	-0.00163	8.43E-04
4.788	-5.20E-04	5.20E-04	4.788	-6.20E-04	6.20E-04	4.788	-7.02E-04	7.02E-04	4.788	-7.70E-04	7.70E-04	4.788	-0.00163	8.43E-04
4.789	-5.20E-04	5.20E-04	4.789	-6.20E-04	6.20E-04	4.789	-7.01E-04	7.01E-04	4.789	-7.70E-04	7.70E-04	4.789	-0.00163	8.42E-04
4.79	-5.20E-04	5.20E-04	4.79	-6.20E-04	6.20E-04	4.79	-7.01E-04	7.01E-04	4.79	-7.69E-04	7.69E-04	4.79	-0.00163	8.42E-04
4.791	-5.20E-04	5.20E-04	4.791	-6.19E-04	6.19E-04	4.791	-7.01E-04	7.01E-04	4.791	-7.69E-04	7.69E-04	4.791	-0.00162	8.41E-04
4.792	-5.20E-04	5.20E-04	4.792	-6.19E-04	6.19E-04	4.792	-7.00E-04	7.00E-04	4.792	-7.69E-04	7.69E-04	4.792	-0.00162	8.41E-04
4.793	-5.19E-04	5.19E-04	4.793	-6.19E-04	6.19E-04	4.793	-7.00E-04	7.00E-04	4.793	-7.68E-04	7.68E-04	4.793	-0.00162	8.40E-04
4.794	-5.19E-04	5.19E-04	4.794	-6.19E-04	6.19E-04	4.794	-7.00E-04	7.00E-04	4.794	-7.68E-04	7.68E-04	4.794	-0.00162	8.40E-04
4.795	-5.19E-04	5.19E-04	4.795	-6.18E-04	6.18E-04	4.795	-7.00E-04	7.00E-04	4.795	-7.68E-04	7.68E-04	4.795	-0.00162	8.39E-04
4.796	-5.19E-04	5.19E-04	4.796	-6.18E-04	6.18E-04	4.796	-6.99E-04	6.99E-04	4.796	-7.67E-04	7.67E-04	4.796	-0.00162	8.39E-04
4.797	-5.18E-04	5.18E-04	4.797	-6.18E-04	6.18E-04	4.797	-6.99E-04	6.99E-04	4.797	-7.67E-04	7.67E-04	4.797	-0.00162	8.38E-04
4.798	-5.18E-04	5.18E-04	4.798	-6.18E-04	6.18E-04	4.798	-6.99E-04	6.99E-04	4.798	-7.67E-04	7.67E-04	4.798	-0.00162	8.38E-04
4.799	-5.18E-04	5.18E-04	4.799	-6.17E-04	6.17E-04	4.799	-6.98E-04	6.98E-04	4.799	-7.66E-04	7.66E-04	4.799	-0.00162	8.37E-04
4.8	-5.18E-04	5.18E-04	4.8	-6.17E-04	6.17E-04	4.8	-6.98E-04	6.98E-04	4.8	-7.66E-04	7.66E-04	4.8	-0.00162	8.37E-04
4.801	-5.18E-04	5.18E-04	4.801	-6.17E-04	6.17E-04	4.801	-6.98E-04	6.98E-04	4.801	-7.66E-04	7.66E-04	4.801	-0.00161	8.36E-04
4.802	-5.17E-04	5.17E-04	4.802	-6.17E-04	6.17E-04	4.802	-6.98E-04	6.98E-04	4.802	-7.65E-04	7.65E-04	4.802	-0.00161	8.36E-04
4.803	-5.17E-04	5.17E-04	4.803	-6.16E-04	6.16E-04	4.803	-6.97E-04	6.97E-04	4.803	-7.65E-04	7.65E-04	4.803	-0.00161	8.35E-04
4.804	-5.17E-04	5.17E-04	4.804	-6.16E-04	6.16E-04	4.804	-6.97E-04	6.97E-04	4.804	-7.65E-04	7.65E-04	4.804	-0.00161	8.35E-04
4.805	-5.17E-04	5.17E-04	4.805	-6.16E-04	6.16E-04	4.805	-6.97E-04	6.97E-04	4.805	-7.65E-04	7.65E-04	4.805	-0.00161	8.34E-04
4.806	-5.16E-04	5.16E-04	4.806	-6.15E-04	6.15E-04	4.806	-6.96E-04	6.96E-04	4.806	-7.64E-04	7.64E-04	4.806	-0.00161	8.34E-04
4.807	-5.16E-04	5.16E-04	4.807	-6.15E-04	6.15E-04	4.807	-6.96E-04	6.96E-04	4.807	-7.64E-04	7.64E-04	4.807	-0.00161	8.33E-04
4.808	-5.16E-04	5.16E-04	4.808	-6.15E-04	6.15E-04	4.808	-6.96E-04	6.96E-04	4.808	-7.64E-04	7.64E-04	4.808	-0.00161	8.33E-04
4.809	-5.16E-04	5.16E-04	4.809	-6.15E-04	6.15E-04	4.809	-6.96E-04	6.96E-04	4.809	-7.63E-04	7.63E-04	4.809	-0.00161	8.32E-04
4.81	-5.16E-04	5.16E-04	4.81	-6.14E-04	6.14E-04	4.81	-6.95E-04	6.95E-04	4.81	-7.63E-04	7.63E-04	4.81	-0.00161	8.32E-04
4.811	-5.15E-04	5.15E-04	4.811	-6.14E-04	6.14E-04	4.811	-6.95E-04	6.95E-04	4.811	-7.63E-04	7.63E-04	4.811	-0.00161	8.31E-04
4.812	-5.15E-04	5.15E-04	4.812	-6.14E-04	6.14E-04	4.812	-6.95E-04	6.95E-04	4.812	-7.62E-04	7.62E-04	4.812	-0.0016	8.31E-04
4.813	-5.15E-04	5.15E-04	4.813	-6.14E-04	6.14E-04	4.813	-6.94E-04	6.94E-04	4.813	-7.62E-04	7.62E-04	4.813	-0.0016	8.30E-04
4.814	-5.15E-04	5.15E-04	4.814	-6.13E-04	6.13E-04	4.814	-6.94E-04	6.94E-04	4.814	-7.62E-04	7.62E-04	4.814	-0.0016	8.30E-04
4.815	-5.15E-04	5.15E-04	4.815	-6.13E-04	6.13E-04	4.815	-6.94E-04	6.94E-04	4.815	-7.61E-04	7.61E-04	4.815	-0.0016	8.29E-04
4.816	-5.14E-04	5.14E-04	4.816	-6.13E-04	6.13E-04	4.816	-6.94E-04	6.94E-04	4.816	-7.61E-04	7.61E-04	4.816	-0.0016	8.29E-04
4.817	-5.14E-04	5.14E-04	4.817	-6.13E-04	6.13E-04	4.817	-6.93E-04	6.93E-04	4.817	-7.61E-04	7.61E-04	4.817	-0.0016	8.28E-04
4.818	-5.14E-04	5.14E-04	4.818	-6.12E-04	6.12E-04	4.818	-6.93E-04	6.93E-04	4.818	-7.60E-04	7.60E-04	4.818	-0.0016	8.28E-04
4.819	-5.14E-04	5.14E-04	4.819	-6.12E-04	6.12E-04	4.819	-6.93E-04	6.93E-04	4.819	-7.60E-04	7.60E-04	4.819	-0.0016	8.27E-04
4.82	-5.13E-04	5.13E-04	4.82	-6.12E-04	6.12E-04	4.82	-6.92E-04	6.92E-04	4.82	-7.60E-04	7.60E-04	4.82	-0.0016	8.27E-04
4.821	-5.13E-04	5.13E-04	4.821	-6.12E-04	6.12E-04	4.821	-6.92E-04	6.92E-04	4.821	-7.59E-04	7.59E-04	4.821	-0.0016	8.26E-04
4.822	-5.13E-04	5.13E-04	4.822	-6.11E-04	6.11E-04	4.822	-6.92E-04	6.92E-04	4.822	-7.59E-04	7.59E-04	4.822	-0.00159	8.26E-04
4.823	-5.13E-04	5.13E-04	4.823	-6.11E-04	6.11E-04	4.823	-6.91E-04	6.91E-04	4.823	-7.59E-04	7.59E-04	4.823	-0.00159	8.25E-04
4.824	-5.13E-04	5.13E-04	4.824	-6.11E-04	6.11E-04	4.824	-6.91E-04	6.91E-04	4.824	-7.59E-04	7.59E-04	4.824	-0.00159	8.25E-04
4.825	-5.12E-04	5.12E-04	4.825	-6.11E-04	6.11E-04	4.825	-6.91E-04	6.91E-04	4.825	-7.58E-04	7.58E-04	4.825	-0.00159	8.24E-04
4.826	-5.12E-04	5.12E-04	4.826	-6.10E-04	6.10E-04	4.826	-6.91E-04	6.91E-04	4.826	-7.58E-04	7.58E-04	4.826	-0.00159	8.24E-04
4.827	-5.12E-04	5.12E-04	4.827	-6.10E-04	6.10E-04	4.827	-6.90E-04	6.90E-04	4.827	-7.58E-04	7.58E-04	4.827	-0.00159	8.23E-04
4.828	-5.12E-04	5.12E-04	4.828	-6.10E-04	6.10E-04	4.828	-6.90E-04	6.90E-04	4.828	-7.57E-04	7.57E-04	4.828	-0.00159	8.23E-04
4.829	-5.12E-04	5.12E-04	4.829	-6.10E-04	6.10E-04	4.829	-6.90E-04	6.90E-04	4.829	-7.57E-04	7.57E-04	4.829	-0.00159	8.22E-04
4.83	-5.11E-04	5.11E-04	4.83	-6.09E-04	6.09E-04	4.83	-6.89E-04	6.89E-04	4.83	-7.57E-04	7.57E-04	4.83	-0.00159	8.22E-04
4.831	-5.11E-04	5.11E-04	4.831	-6.09E-04	6.09E-04	4.831	-6.89E-04	6.89E-04	4.831	-7.56E-04	7.56E-04	4.831	-0.00159	8.21E-04
4.832	-5.11E-04	5.11E-04	4.832	-6.09E-04	6.09E-04	4.832	-6.89E-04	6.89E-04	4.832	-7.56E-04	7.56E-04	4.832	-0.00158	8.21E-04
4.833	-5.11E-04	5.11E-04	4.833	-6.09E-04	6.09E-04	4.833	-6.89E-04	6.89E-04	4.833	-7.56E-04	7.56E-04	4.833	-0.00158	8.20E-04
4.834	-5.11E-04	5.11E-04	4.834	-6.08E-04	6.08E-04	4.834	-6.88E-04	6.88E-04	4.834	-7.55E-04	7.55E-04	4.834	-0.00158	8.20E-04
4.835	-5.10E-04	5.10E-04	4.835	-6.08E-04	6.08E-04	4.835	-6.88E-04	6.88E-04	4.835	-7.55E-04	7.55E-04	4.835	-0.00158	8.19E-04
4.836	-5.10E-04	5.10E-04	4.836	-6.08E-04	6.08E-04	4.836	-6.88E-04	6.88E-04	4.836	-7.55E-04	7.55E-04	4.836	-0.00158	8.19E-04
4.837	-5.10E-04	5.10E-04	4.837	-6.08E-04	6.08E-04	4.837	-6.88E-04	6.88E-04	4.837	-7.54E-04	7.54E-04	4.837	-0.00158	8.18E-04
4.838	-5.10E-04	5.10E-04	4.838	-6.07E-04	6.07E-04	4.838	-6.87E-04	6.87E-04	4.838	-7.54E-04	7.54E-04	4.838	-0.00158	8.18E-04
4.839	-5.09E-04	5.09E-04	4.839	-6.07E-04	6.07E-04	4.839	-6.87E-04	6.87E-04	4.839	-7.54E-04	7.54E-04	4.839	-0.00158	8.17E-04
4.84	-5.09E-04	5.09E-04	4.84	-6.07E-04	6.07E-04	4.84	-6.87E-04	6.87E-04	4.84	-7.54E-04	7.54E-04	4.84	-0.00158	8.17E-04
4.841	-5.09E-04	5.09E-04	4.841	-6.07E-04	6.07E-04	4.841	-6.86E-04	6.86E-04	4.841	-7.53E-04	7.53E-04	4.841	-0.00158	8.16E-04
4.842	-5.09E-04	5.09E-04	4.842	-6.06E-04	6.06E-04	4.842	-6.86E-04	6.86E-04	4.842	-7.53E-04	7.53E-04	4.842	-0.00158	8.16E-04
4.843	-5.09E-04	5.09E-04	4.843	-6.06E-04	6.06E-04	4.843	-6.86E-04	6.86E-04	4.843	-7.53E-04	7.53E-04	4.843	-0.00157	8.15E-04
4.844	-5.08E-04	5.08E-04	4.844	-6.06E-04	6.06E-04	4.844	-6.86E-04	6.86E-04	4.844	-7.52E-04	7.52E-04	4.844	-0.00157	8.15E-04
4.845	-5.08E-04	5.08E-04	4.845	-6.06E-04	6.06E-04	4.845	-6.85E-04	6.85E-04	4.845	-7.52E-04	7.52E-04	4.845	-0.00157	8.14E-04
4.846	-5.08E-04	5.08E-04	4.846	-6.05E-04	6.05E-04	4.846	-6.85E-04	6.85E-04	4.846	-7.52E-04	7.52E-04	4.846	-0.00157	8.14E-04
4.847	-5.08E-04	5.08E-04	4.847	-6.05E-04	6.05E-04	4.847	-6.85E-04	6.85E-04	4.847	-7.51E-04	7.51E-04	4.847	-0.00157	8.13E-04

4.848	-5.08E-04	5.08E-04	4.848	-6.05E-04	6.05E-04	4.848	-6.84E-04	6.84E-04	4.848	-7.51E-04	7.51E-04	4.848	-0.00157	8.13E-04
4.849	-5.07E-04	5.07E-04	4.849	-6.05E-04	6.05E-04	4.849	-6.84E-04	6.84E-04	4.849	-7.51E-04	7.51E-04	4.849	-0.00157	8.12E-04
4.85	-5.07E-04	5.07E-04	4.85	-6.04E-04	6.04E-04	4.85	-6.84E-04	6.84E-04	4.85	-7.50E-04	7.50E-04	4.85	-0.00157	8.12E-04
4.851	-5.07E-04	5.07E-04	4.851	-6.04E-04	6.04E-04	4.851	-6.84E-04	6.84E-04	4.851	-7.50E-04	7.50E-04	4.851	-0.00157	8.11E-04
4.852	-5.07E-04	5.07E-04	4.852	-6.04E-04	6.04E-04	4.852	-6.83E-04	6.83E-04	4.852	-7.50E-04	7.50E-04	4.852	-0.00157	8.11E-04
4.853	-5.07E-04	5.07E-04	4.853	-6.04E-04	6.04E-04	4.853	-6.83E-04	6.83E-04	4.853	-7.49E-04	7.49E-04	4.853	-0.00156	8.10E-04
4.854	-5.06E-04	5.06E-04	4.854	-6.03E-04	6.03E-04	4.854	-6.83E-04	6.83E-04	4.854	-7.49E-04	7.49E-04	4.854	-0.00156	8.10E-04
4.855	-5.06E-04	5.06E-04	4.855	-6.03E-04	6.03E-04	4.855	-6.82E-04	6.82E-04	4.855	-7.49E-04	7.49E-04	4.855	-0.00156	8.09E-04
4.856	-5.06E-04	5.06E-04	4.856	-6.03E-04	6.03E-04	4.856	-6.82E-04	6.82E-04	4.856	-7.49E-04	7.49E-04	4.856	-0.00156	8.09E-04
4.857	-5.06E-04	5.06E-04	4.857	-6.03E-04	6.03E-04	4.857	-6.82E-04	6.82E-04	4.857	-7.48E-04	7.48E-04	4.857	-0.00156	8.08E-04
4.858	-5.05E-04	5.05E-04	4.858	-6.02E-04	6.02E-04	4.858	-6.82E-04	6.82E-04	4.858	-7.48E-04	7.48E-04	4.858	-0.00156	8.08E-04
4.859	-5.05E-04	5.05E-04	4.859	-6.02E-04	6.02E-04	4.859	-6.81E-04	6.81E-04	4.859	-7.48E-04	7.48E-04	4.859	-0.00156	8.07E-04
4.86	-5.05E-04	5.05E-04	4.86	-6.02E-04	6.02E-04	4.86	-6.81E-04	6.81E-04	4.86	-7.47E-04	7.47E-04	4.86	-0.00156	8.07E-04
4.861	-5.05E-04	5.05E-04	4.861	-6.02E-04	6.02E-04	4.861	-6.81E-04	6.81E-04	4.861	-7.47E-04	7.47E-04	4.861	-0.00156	8.06E-04
4.862	-5.05E-04	5.05E-04	4.862	-6.01E-04	6.01E-04	4.862	-6.80E-04	6.80E-04	4.862	-7.47E-04	7.47E-04	4.862	-0.00156	8.06E-04
4.863	-5.04E-04	5.04E-04	4.863	-6.01E-04	6.01E-04	4.863	-6.80E-04	6.80E-04	4.863	-7.46E-04	7.46E-04	4.863	-0.00156	8.05E-04
4.864	-5.04E-04	5.04E-04	4.864	-6.01E-04	6.01E-04	4.864	-6.80E-04	6.80E-04	4.864	-7.46E-04	7.46E-04	4.864	-0.00155	8.05E-04
4.865	-5.04E-04	5.04E-04	4.865	-6.01E-04	6.01E-04	4.865	-6.80E-04	6.80E-04	4.865	-7.46E-04	7.46E-04	4.865	-0.00155	8.04E-04
4.866	-5.04E-04	5.04E-04	4.866	-6.00E-04	6.00E-04	4.866	-6.79E-04	6.79E-04	4.866	-7.45E-04	7.45E-04	4.866	-0.00155	8.04E-04
4.867	-5.04E-04	5.04E-04	4.867	-6.00E-04	6.00E-04	4.867	-6.79E-04	6.79E-04	4.867	-7.45E-04	7.45E-04	4.867	-0.00155	8.03E-04
4.868	-5.03E-04	5.03E-04	4.868	-6.00E-04	6.00E-04	4.868	-6.79E-04	6.79E-04	4.868	-7.45E-04	7.45E-04	4.868	-0.00155	8.03E-04
4.869	-5.03E-04	5.03E-04	4.869	-6.00E-04	6.00E-04	4.869	-6.78E-04	6.78E-04	4.869	-7.45E-04	7.45E-04	4.869	-0.00155	8.02E-04
4.87	-5.03E-04	5.03E-04	4.87	-5.99E-04	5.99E-04	4.87	-6.78E-04	6.78E-04	4.87	-7.44E-04	7.44E-04	4.87	-0.00155	8.02E-04
4.871	-5.03E-04	5.03E-04	4.871	-5.99E-04	5.99E-04	4.871	-6.78E-04	6.78E-04	4.871	-7.44E-04	7.44E-04	4.871	-0.00155	8.01E-04
4.872	-5.03E-04	5.03E-04	4.872	-5.99E-04	5.99E-04	4.872	-6.78E-04	6.78E-04	4.872	-7.44E-04	7.44E-04	4.872	-0.00155	8.01E-04
4.873	-5.02E-04	5.02E-04	4.873	-5.99E-04	5.99E-04	4.873	-6.77E-04	6.77E-04	4.873	-7.43E-04	7.43E-04	4.873	-0.00155	8.01E-04
4.874	-5.02E-04	5.02E-04	4.874	-5.98E-04	5.98E-04	4.874	-6.77E-04	6.77E-04	4.874	-7.43E-04	7.43E-04	4.874	-0.00154	8.00E-04
4.875	-5.02E-04	5.02E-04	4.875	-5.98E-04	5.98E-04	4.875	-6.77E-04	6.77E-04	4.875	-7.43E-04	7.43E-04	4.875	-0.00154	8.00E-04
4.876	-5.02E-04	5.02E-04	4.876	-5.98E-04	5.98E-04	4.876	-6.77E-04	6.77E-04	4.876	-7.42E-04	7.42E-04	4.876	-0.00154	7.99E-04
4.877	-5.02E-04	5.02E-04	4.877	-5.98E-04	5.98E-04	4.877	-6.76E-04	6.76E-04	4.877	-7.42E-04	7.42E-04	4.877	-0.00154	7.99E-04
4.878	-5.01E-04	5.01E-04	4.878	-5.97E-04	5.97E-04	4.878	-6.76E-04	6.76E-04	4.878	-7.42E-04	7.42E-04	4.878	-7.98E-04	7.98E-04
4.879	-5.01E-04	5.01E-04	4.879	-5.97E-04	5.97E-04	4.879	-6.76E-04	6.76E-04	4.879	-7.42E-04	7.42E-04	4.879	-7.98E-04	7.98E-04
4.88	-5.01E-04	5.01E-04	4.88	-5.97E-04	5.97E-04	4.88	-6.75E-04	6.75E-04	4.88	-7.41E-04	7.41E-04	4.88	-7.98E-04	7.98E-04
4.881	-5.01E-04	5.01E-04	4.881	-5.97E-04	5.97E-04	4.881	-6.75E-04	6.75E-04	4.881	-7.41E-04	7.41E-04	4.881	-7.97E-04	7.97E-04
4.882	-5.01E-04	5.01E-04	4.882	-5.96E-04	5.96E-04	4.882	-6.75E-04	6.75E-04	4.882	-7.41E-04	7.41E-04	4.882	-7.97E-04	7.97E-04
4.883	-5.00E-04	5.00E-04	4.883	-5.96E-04	5.96E-04	4.883	-6.75E-04	6.75E-04	4.883	-7.40E-04	7.40E-04	4.883	-7.97E-04	7.97E-04
4.884	-5.00E-04	5.00E-04	4.884	-5.96E-04	5.96E-04	4.884	-6.74E-04	6.74E-04	4.884	-7.40E-04	7.40E-04	4.884	-7.96E-04	7.96E-04
4.885	-5.00E-04	5.00E-04	4.885	-5.96E-04	5.96E-04	4.885	-6.74E-04	6.74E-04	4.885	-7.40E-04	7.40E-04	4.885	-7.96E-04	7.96E-04
4.886	-5.00E-04	5.00E-04	4.886	-5.96E-04	5.96E-04	4.886	-6.74E-04	6.74E-04	4.886	-7.39E-04	7.39E-04	4.886	-7.96E-04	7.96E-04
4.887	-5.00E-04	5.00E-04	4.887	-5.95E-04	5.95E-04	4.887	-6.74E-04	6.74E-04	4.887	-7.39E-04	7.39E-04	4.887	-7.95E-04	7.95E-04
4.888	-4.99E-04	4.99E-04	4.888	-5.95E-04	5.95E-04	4.888	-6.73E-04	6.73E-04	4.888	-7.39E-04	7.39E-04	4.888	-7.95E-04	7.95E-04
4.889	-4.99E-04	4.99E-04	4.889	-5.95E-04	5.95E-04	4.889	-6.73E-04	6.73E-04	4.889	-7.38E-04	7.38E-04	4.889	-7.95E-04	7.95E-04
4.89	-4.99E-04	4.99E-04	4.89	-5.95E-04	5.95E-04	4.89	-6.73E-04	6.73E-04	4.89	-7.38E-04	7.38E-04	4.89	-7.94E-04	7.94E-04
4.891	-4.99E-04	4.99E-04	4.891	-5.94E-04	5.94E-04	4.891	-6.72E-04	6.72E-04	4.891	-7.38E-04	7.38E-04	4.891	-7.94E-04	7.94E-04
4.892	-4.98E-04	4.98E-04	4.892	-5.94E-04	5.94E-04	4.892	-6.72E-04	6.72E-04	4.892	-7.38E-04	7.38E-04	4.892	-7.94E-04	7.94E-04
4.893	-4.98E-04	4.98E-04	4.893	-5.94E-04	5.94E-04	4.893	-6.72E-04	6.72E-04	4.893	-7.37E-04	7.37E-04	4.893	-7.93E-04	7.93E-04
4.894	-4.98E-04	4.98E-04	4.894	-5.94E-04	5.94E-04	4.894	-6.72E-04	6.72E-04	4.894	-7.37E-04	7.37E-04	4.894	-7.93E-04	7.93E-04
4.895	-4.98E-04	4.98E-04	4.895	-5.93E-04	5.93E-04	4.895	-6.71E-04	6.71E-04	4.895	-7.37E-04	7.37E-04	4.895	-7.93E-04	7.93E-04
4.896	-4.98E-04	4.98E-04	4.896	-5.93E-04	5.93E-04	4.896	-6.71E-04	6.71E-04	4.896	-7.36E-04	7.36E-04	4.896	-7.92E-04	7.92E-04
4.897	-4.97E-04	4.97E-04	4.897	-5.93E-04	5.93E-04	4.897	-6.71E-04	6.71E-04	4.897	-7.36E-04	7.36E-04	4.897	-7.92E-04	7.92E-04
4.898	-4.97E-04	4.97E-04	4.898	-5.93E-04	5.93E-04	4.898	-6.70E-04	6.70E-04	4.898	-7.36E-04	7.36E-04	4.898	-7.92E-04	7.92E-04
4.899	-4.97E-04	4.97E-04	4.899	-5.92E-04	5.92E-04	4.899	-6.70E-04	6.70E-04	4.899	-7.35E-04	7.35E-04	4.899	-7.91E-04	7.91E-04
4.9	-4.97E-04	4.97E-04	4.9	-5.92E-04	5.92E-04	4.9	-6.70E-04	6.70E-04	4.9	-7.35E-04	7.35E-04	4.9	-7.91E-04	7.91E-04
4.901	-4.97E-04	4.97E-04	4.901	-5.92E-04	5.92E-04	4.901	-6.70E-04	6.70E-04	4.901	-7.35E-04	7.35E-04	4.901	-7.91E-04	7.91E-04
4.902	-4.96E-04	4.96E-04	4.902	-5.92E-04	5.92E-04	4.902	-6.69E-04	6.69E-04	4.902	-7.35E-04	7.35E-04	4.902	-7.90E-04	7.90E-04
4.903	-4.96E-04	4.96E-04	4.903	-5.91E-04	5.91E-04	4.903	-6.69E-04	6.69E-04	4.903	-7.34E-04	7.34E-04	4.903	-7.90E-04	7.90E-04
4.904	-4.96E-04	4.96E-04	4.904	-5.91E-04	5.91E-04	4.904	-6.69E-04	6.69E-04	4.904	-7.34E-04	7.34E-04	4.904	-7.90E-04	7.90E-04
4.905	-4.96E-04	4.96E-04	4.905	-5.91E-04	5.91E-04	4.905	-6.69E-04	6.69E-04	4.905	-7.34E-04	7.34E-04	4.905	-7.89E-04	7.89E-04
4.906	-4.96E-04	4.96E-04	4.906	-5.91E-04	5.91E-04	4.906	-6.68E-04	6.68E-04	4.906	-7.33E-04	7.33E-04	4.906	-7.89E-04	7.89E-04
4.907	-4.95E-04	4.95E-04	4.907	-5.90E-04	5.90E-04	4.907	-6.68E-04	6.68E-04	4.907	-7.33E-04	7.33E-04	4.907	-7.89E-04	7.89E-04
4.908	-4.95E-04	4.95E-04	4.908	-5.90E-04	5.90E-04	4.908	-6.68E-04	6.68E-04	4.908	-7.33E-04	7.33E-04	4.908	-7.88E-04	7.88E-04
4.909	-4.95E-04	4.95E-04	4.909	-5.90E-04	5.90E-04	4.909	-6.67E-04	6.67E-04	4.909	-7.32E-04	7.32E-04	4.909	-7.88E-04	7.88E-04
4.91	-4.95E-04	4.95E-04	4.91	-5.90E-04	5.90E-04	4.91	-6.67E-04	6.67E-04	4.91	-7.32E-04	7.32E-04	4.91	-7.88E-04	7.88E-04
4.911	-4.95E-04	4.95E-04	4.911	-5.89E-04	5.89E-04	4.911	-6.67E-04	6.67E-04	4.911	-7.32E-04	7.32E-04	4.911	-7.88E-04	7.88E-04
4.912	-4.94E-04	4.94E-04	4.912	-5.89E-04	5.89E-04	4.912	-6.67E-04	6.67E-04	4.912	-7.32E-04	7.32E-04	4.912	-7.87E-04	7.87E-04
4.913	-4.94E-04	4.94E-04	4.913	-5.89E-04	5.89E-04	4.913	-6.66E-04	6.66E-04	4.913	-7.31E-04	7.31E-04	4.913	-7.87E-04	7.87E-04
4.914	-4.94E-04	4.94E-04	4.914	-5.89E-04	5.89E-04	4.914	-6.66E-04	6.66E-04	4.914	-7.31E-04	7.31E-04	4.914	-7.87E-04	7.87E-04
4.915	-4.94E-04	4.94E-04	4.915	-5.89E-04	5.89E-04	4.915	-6.66E-04	6.66E-04	4.915	-7.31E-04	7.31E-04	4.915	-7.86E-04	7.86E-04
4.916	-4.94E-04	4.94E-04	4.916	-5.88E-04	5.88E-04	4.916	-6.66E-04	6.66E-04	4.916	-7.30E-04	7.30E-04	4.916	-7.86E-04	7.86E-04
4.917	-4.93E-04	4.93E-04	4.917	-5.88E-04	5.88E-04	4.917	-6.65E-04	6.65E-04	4.917	-7.30E-04	7.30E-04	4.917	-7.86E-04	7.86E-04
4.918	-4.93E-04	4.93E-04	4.918	-5.88E-04	5.88E-04	4.918	-6.65E-04	6.65E-04	4.918	-7.30E-04	7.30E-04	4.918	-7.85E-04	7.85E-04
4.919	-4.93E-04	4.93E-04	4.919	-5.88E-04	5.88E-04	4.919	-6.65E-04	6.65E-04	4.919	-7.30E-04	7.30E-04	4.919	-7.85E-04	7.85E-04
4.92	-4.93E-04	4.93E-04	4.92	-5.87E-04	5.87E-04	4.92	-6.64E-04	6.64E-04	4.92	-7.29E-04	7.29E-04	4.92	-7.85E-04	7.85E-04
4.921	-4.93E-04	4.93E-04	4.921	-5.87E-04	5.87E-04	4.921	-6.64E-04	6.64E-04	4.921	-7.29E-04	7.29E-04	4.921	-7.84E-04	7.84E-04
4.922	-4.92E-04	4.92E-04	4.922	-5.87E-04	5.87E-04	4.922	-6.64E-04	6.64E-04	4.922	-7.29E-04	7.29E-04	4.922	-7.84E-04	7.84E-04
4.923	-4.92E-04	4.92E-04	4.923	-5.87E-04	5.87E-04	4.923	-6.64E-04	6.64E-04	4.923	-7.28E-04	7.28E-04	4.923	-7.84E-04	7.84E-04
4.924	-4.92E-04	4.92E-04	4.924	-5.86E-04	5.86E-04	4.924	-6.63E-04	6.63E-04	4.924	-7.28E-04	7.28E-04	4.924	-7.83E-04	7.83E-04
4.925	-4.92E-04	4.92E-04	4.925	-5.86E-04	5.86E-04	4.925	-6.63E-04	6.63E-04	4.925	-7.28E-04	7.28E-04	4.925	-7.83E-04	7.83E-04
4.926	-4.92E-04	4.92E-04	4.926	-5.86E-04	5.86E-04	4.926	-6.63E-04	6.63E-04	4.926	-7.27E-04	7.27E-04	4.926	-7.83E-04	7.83E-04
4.927	-4.91E-04	4.91E-04	4.927	-5.86E-04	5.86E-04	4.927	-6.63E-04	6.63E-04	4.927	-7.27E-04	7.27E-04	4.927	-7.82E-04	7.82E-04
4.928	-4.91E-04	4.91E-04	4.928	-5.85E-04	5.85E-04	4.928	-6.62E-04	6.62E-04	4.928	-7.27E-04	7.27E-04	4.928	-7.82E-04	7.82E-04
4.929	-4.91E-04	4.91E-04	4.929	-5.85E-04	5.85E-04	4.929	-6.62E-04	6.62E-04	4.929	-7.27E-04	7.27E-04	4.929	-7.82E-04	7.82E-04
4.93	-4.91E-04	4.91E-04	4.93	-5.85E-04	5.85E-04	4.93	-6.62E-04	6.62E-04	4.93	-7.26E-04	7.26E-04	4.93	-7.81E-04	7.81E-04
4.931	-4.91E-04	4.91E-04	4.931	-5.85E-04	5.85E-04	4.931	-6.62E-04	6.62E-04	4.931	-7.26E-04	7.26E-04	4.931	-7.81E-04	7.81E-04
4.932	-4.90E-04	4.90E-04	4.932	-5.84E-04	5.84E-04	4.932	-6.61E-04	6.61E-04	4.932	-7.26E-04	7.26E-04	4.932	-7.81E-04	7.81E-04
4.933	-4.90E-04	4.90E-04	4.933	-5.84E-04	5.84E-04	4.933	-6.61E-04	6.61E-04	4.933	-7.25E-04	7.25E-04	4.933	-7.80E-04	7.80E-04
4.934	-4.90E-04	4.90E-04	4.934	-5.84E-04	5.84E-04	4.934	-6.61E-04	6.61E-04	4.934	-7.25E-04	7.25E-04	4.934	-7.80E-04	7.80E-04
4.935	-4.90E-04	4.90E-04	4.935	-5.84E-04	5.84E-04	4.935	-6.60E-04	6.60E-04	4.935	-7.25E-04	7.25E-04	4.935	-7.80E-04	7.80E-04
4.936	-4.90E-04	4.90E-04	4.936	-5.84E-04	5.84E-04	4.936	-6.60E-04	6.60E-04	4.936	-7.24E-04	7.24E-04	4.936	-7.80E-04	7.80E-04
4.937	-4.89E-04	4.89E-04	4.937	-5.83E-04	5.83E-04	4.937	-6.60E-04	6.60E-04	4.937	-7.24E-04	7.24E-04	4.937	-7.79E-04	7.79E-04
4.938	-4.89E-04	4.89E-04	4.938	-5.83E-04	5.83E-04	4.938	-6.60E-04	6.60E-04	4.938	-7.24E-04	7.24E-04	4.938	-7.79E-04	7.79E-04
4.939	-4.89E-04	4.89E-04	4.939	-5.83E-04	5.83E-04	4.939	-6.59E-04	6.59E-04	4.939	-7.24E-04	7.24E-04	4.939	-7.79E-04	7.79E-04
4.94	-4.89E-04	4.89E-04	4.94	-5.83E-04	5.83E-04	4.94	-6.59E-04	6.59E-04	4.94	-7.23E-04	7.23E-04	4.94	-7.78E-04	7.78E-04
4.941	-4.89E-04	4.89E-04	4.941	-5.82E-04	5.82E-04	4.941	-6.59E-04	6.59E-04	4.941	-7.23E-04	7.23E-04	4.941	-7.78E-04	7.78E-04
4.942	-4.88E-04	4.88E-04	4.942	-5.82E-04	5.82E-04	4.942	-6.59E-04	6.59E-04	4.942	-7.23E-04	7.23E-04	4.942	-7.78E-04	7.78E-04
4.943	-4.88E-04	4.88E-04	4.943	-5.82E-04	5.82E-04	4.943	-6.58E-04	6.58E-04	4.943	-7.22E-04	7.22E-04	4.943	-7.77E-04	7.77E-04
4.944	-4.88E-04	4.88E-04	4.944	-5.82E-04	5.82E-04	4.944	-6.58E-04	6.58E-04	4.944	-7.22E-04	7.22E-04	4.944	-7.77E-04	7.77E-04
4.945	-4.88E-04	4.88E-04	4.945	-5.81E-04	5.81E-04	4.945	-6.58E-04	6.58E-04	4.945	-7.22E-04	7.22E-04	4.945	-7.77E-04	7.77E-04
4.946	-4.88E-04	4.88E-04	4.946	-5.81E-04	5.81E-04	4.946	-6.58E-04	6.58E-04	4.946	-7.22E-04	7.22E-04	4.946	-7.76E-04	7.76E-04
4.947	-4.87E-04	4.87E-04	4.947	-5.81E-04	5.81E-04	4.947	-6.57E-04	6.57E-04	4.947	-7.21E-04	7.21E-04	4.947	-7.76E-04	7.76E-04
4.948	-4.87E-04	4.87E-04	4.948	-5.81E-04	5.81E-04	4.948	-6.57E-04	6.57E-04	4.948	-7.21E-04	7.21E-04	4.948	-7.76E-04	7.76E-04
4.949	-4.87E-04	4.87E-04	4.949	-5.80E-04	5.80E-04	4.949	-6.57E-04	6.57E-04	4.949	-7.21E-04	7.21E-04	4.949	-7.75E-04	7.75E-04
4.95	-4.87E-04	4.87E-04	4.95	-5.80E-04	5.80E-04	4.95	-6.56E-04	6.56E-04	4.95	-7.20E-04	7.20E-04	4.95	-7.75E-04	7.75E-04
4.951	-4.87E-04	4.87E-04	4.951	-5.80E-04	5.80E-04	4.951	-6.56E-04	6.56E-04	4.951	-7.20E-04	7.20E-04	4.951	-7.75E-04	7.75E-04
4.952	-4.86E-04	4.86E-04	4.952	-5.80E-04	5.80E-04	4.952	-6.56E-04	6.56E-04	4.952	-7.20E-04	7.20E-04	4.952	-7.75E-04	7.75E-04
4.953	-4.86E-04	4.86E-04	4.953	-5.80E-04	5.80E-04	4.953	-6.56E-04	6.56E-04	4.953	-7.20E-04	7.20E-04	4.953	-7.74E-04	7.74E-04
4.954	-4.86E-04	4.86E-04	4.954	-5.79E-04	5.79E-04	4.954	-6.55E-04	6.55E-04	4.954	-7.19E-04	7.19E-04	4.954	-7.74E-04	7.74E-04
4.955	-4.86E-04	4.86E-04	4.955	-5.79E-04	5.79E-04	4.955	-6.55E-04	6.55E-04	4.955	-7.19E-04	7.19E-04	4.955	-7.74E-04	7.74E-04
4.956	-4.86E-04	4.86E-04	4.956	-5.79E-04	5.79E-04	4.956	-6.55E-04	6.55E-04	4.956	-7.19E-04	7.19E-04	4.956	-7.73E-04	7.73E-04
4.957	-4.86E-04	4.86E-04	4.957	-5.79E-04	5.79E-04	4.957	-6.55E-04	6.55E-04	4.957	-7.18E-04	7.18E-04	4.957	-7.73E-04	7.73E-04
4.958	-4.85E-04	4.85E-04	4.958	-5.78E-04	5.78E-04	4.958	-6.54E-04	6.54E-04	4.958	-7.18E-04	7.18E-04	4.958	-7.73E-04	7.73E-04
4.959	-4.85E-04	4.85E-04	4.959	-5.78E-04	5.78E-04	4.959	-6.54E-04	6.54E-04	4.959	-7.18E-04	7.18E-04	4.959	-7.72E-04	7.72E-04
4.96	-4.85E-04	4.85E-04	4.96	-5.78E-04	5.78E-04	4.96	-6.54E-04	6.54E-04	4.96	-7.17E-04	7.17E-04	4.96	-7.72E-04	7.72E-04
4.961	-4.85E-04	4.85E-04	4.961	-5.78E-04	5.78E-04	4.961	-6.54E-04	6.54E-04	4.961	-7.17E-04	7.17E-04	4.961	-7.72E-04	7.72E-04
4.962	-4.85E-04	4.85E-04	4.962	-5.77E-04	5.77E-04	4.962	-6.53E-04	6.53E-04	4.962	-7.17E-04	7.17E-04	4.962	-7.71E-04	7.71E-04
4.963	-4.84E-04	4.84E-04	4.963	-5.77E-04	5.77E-04	4.963	-6.53E-04	6.53E-04	4.963	-7.17E-04	7.17E-04	4.963	-7.71E-04	7.71E-04
4.964	-4.84E-04	4.84E-04	4.964	-5.77E-04	5.77E-04	4.964	-6.53E-04	6.53E-04	4.964	-7.16E-04	7.16E-04	4.964	-7.71E-04	7.71E-04
4.965	-4.84E-04	4.84E-04	4.965	-5.77E-04	5.77E-04	4.965	-6.53E-04	6.53E-04	4.965	-7.16E-04	7.16E-04	4.965	-7.70E-04	7.70E-04
4.966	-4.84E-04	4.84E-04	4.966	-5.76E-04	5.76E-04	4.966	-6.52E-04	6.52E-04	4.966	-7.16E-04	7.16E-04	4.966	-7.70E-04	7.70E-04
4.967	-4.84E-04	4.84E-04	4.967	-5.76E-04	5.76E-04	4.967	-6.52E-04	6.52E-04	4.967	-7.15E-04	7.15E-04	4.967	-7.70E-04	7.70E-04
4.968	-4.83E-04	4.83E-04	4.968	-5.76E-04	5.76E-04	4.968	-6.52E-04	6.52E-04	4.968	-7.15E-04	7.15E-04	4.968	-7.70E-04	7.70E-04
4.969	-4.83E-04	4.83E-04	4.969	-5.76E-04	5.76E-04	4.969	-6.51E-04	6.51E-04	4.969	-7.15E-04	7.15E-04	4.969	-7.69E-04	7.69E-04
4.97	-4.83E-04	4.83E-04	4.97	-5.76E-04	5.76E-04	4.97	-6.51E-04	6.51E-04	4.97	-7.15E-04	7.15E-04	4.97	-7.69E-04	7.69E-04
4.971	-4.83E-04	4.83E-04	4.971	-5.75E-04	5.75E-04	4.971	-6.51E-04	6.51E-04	4.971	-7.14E-04	7.14E-04	4.971	-7.69E-04	7.69E-04
4.972	-4.83E-04	4.83E-04	4.972	-5.75E-04	5.75E-04	4.972	-6.51E-04	6.51E-04	4.972	-7.14E-04	7.14E-04	4.972	-7.68E-04	7.68E-04
4.973	-4.82E-04	4.82E-04	4.973	-5.75E-04	5.75E-04	4.973	-6.50E-04	6.50E-04	4.973	-7.14E-04	7.14E-04	4.973	-7.68E-04	7.68E-04
4.974	-4.82E-04	4.82E-04	4.974	-5.75E-04	5.75E-04	4.974	-6.50E-04	6.50E-04	4.974	-7.13E-04	7.13E-04	4.974	-7.68E-04	7.68E-04
4.975	-4.82E-04	4.82E-04	4.975	-5.74E-04	5.74E-04	4.975	-6.50E-04	6.50E-04	4.975	-7.13E-04	7.13E-04	4.975	-7.67E-04	7.67E-04
4.976	-4.82E-04	4.82E-04	4.976	-5.74E-04	5.74E-04	4.976	-6.50E-04	6.50E-04	4.976	-7.13E-04	7.13E-04	4.976	-7.67E-04	7.67E-04
4.977	-4.82E-04	4.82E-04	4.977	-5.74E-04	5.74E-04	4.977	-6.49E-04	6.49E-04	4.977	-7.13E-04	7.13E-04	4.977	-7.67E-04	7.67E-04
4.978	-4.81E-04	4.81E-04	4.978	-5.74E-04	5.74E-04	4.978	-6.49E-04	6.49E-04	4.978	-7.12E-04	7.12E-04	4.978	-7.66E-04	7.66E-04
4.979	-4.81E-04	4.81E-04	4.979	-5.73E-04	5.73E-04	4.979	-6.49E-04	6.49E-04	4.979	-7.12E-04	7.12E-04	4.979	-7.66E-04	7.66E-04
4.98	-4.81E-04	4.81E-04	4.98	-5.73E-04	5.73E-04	4.98	-6.49E-04	6.49E-04	4.98	-7.12E-04	7.12E-04	4.98	-7.66E-04	7.66E-04
4.981	-4.81E-04	4.81E-04	4.981	-5.73E-04	5.73E-04	4.981	-6.48E-04	6.48E-04	4.981	-7.11E-04	7.11E-04	4.981	-7.66E-04	7.66E-04
4.982	-4.81E-04	4.81E-04	4.982	-5.73E-04	5.73E-04	4.982	-6.48E-04	6.48E-04	4.982	-7.11E-04	7.11E-04	4.982	-7.65E-04	7.65E-04
4.983	-4.80E-04	4.80E-04	4.983	-5.73E-04	5.73E-04	4.983	-6.48E-04	6.48E-04	4.983	-7.11E-04	7.11E-04	4.983	-7.65E-04	7.65E-04
4.984	-4.80E-04	4.80E-04	4.984	-5.72E-04	5.72E-04	4.984	-6.48E-04	6.48E-04	4.984	-7.11E-04	7.11E-04	4.984	-7.65E-04	7.65E-04
4.985	-4.80E-04	4.80E-04	4.985	-5.72E-04	5.72E-04	4.985	-6.47E-04	6.47E-04	4.985	-7.10E-04	7.10E-04	4.985	-7.64E-04	7.64E-04
4.986	-4.80E-04	4.80E-04	4.986	-5.72E-04	5.72E-04	4.986	-6.47E-04	6.47E-04	4.986	-7.10E-04	7.10E-04	4.986	-7.64E-04	7.64E-04
4.987	-4.80E-04	4.80E-04	4.987	-5.72E-04	5.72E-04	4.987	-6.47E-04	6.47E-04	4.987	-7.10E-04	7.10E-04	4.987	-7.64E-04	7.64E-04
4.988	-4.79E-04	4.79E-04	4.988	-5.71E-04	5.71E-04	4.988	-6.47E-04	6.47E-04	4.988	-7.09E-04	7.09E-04	4.988	-7.63E-04	7.63E-04
4.989	-4.79E-04	4.79E-04	4.989	-5.71E-04	5.71E-04	4.989	-6.46E-04	6.46E-04	4.989	-7.09E-04	7.09E-04	4.989	-7.63E-04	7.63E-04
4.99	-4.79E-04	4.79E-04	4.99	-5.71E-04	5.71E-04	4.99	-6.46E-04	6.46E-04	4.99	-7.09E-04	7.09E-04	4.99	-7.63E-04	7.63E-04
4.991	-4.79E-04	4.79E-04	4.991	-5.71E-04	5.71E-04	4.991	-6.46E-04	6.46E-04	4.991	-7.09E-04	7.09E-04	4.991	-7.62E-04	7.62E-04
4.992	-4.79E-04	4.79E-04	4.992	-5.70E-04	5.70E-04	4.992	-6.45E-04	6.45E-04	4.992	-7.08E-04	7.08E-04	4.992	-7.62E-04	7.62E-04
4.993	-4.79E-04	4.79E-04	4.993	-5.70E-04	5.70E-04	4.993	-6.45E-04	6.45E-04	4.993	-7.08E-04	7.08E-04	4.993	-7.62E-04	7.62E-04
4.994	-4.78E-04	4.78E-04	4.994	-5.70E-04	5.70E-04	4.994	-6.45E-04	6.45E-04	4.994	-7.08E-04	7.08E-04	4.994	-7.62E-04	7.62E-04
4.995	-4.78E-04	4.78E-04	4.995	-5.70E-04	5.70E-04	4.995	-6.45E-04	6.45E-04	4.995	-7.07E-04	7.07E-04	4.995	-7.61E-04	7.61E-04
4.996	-4.78E-04	4.78E-04	4.996	-5.70E-04	5.70E-04	4.996	-6.44E-04	6.44E-04	4.996	-7.07E-04	7.07E-04	4.996	-7.61E-04	7.61E-04
4.997	-4.78E-04	4.78E-04	4.997	-5.69E-04	5.69E-04	4.997	-6.44E-04	6.44E-04	4.997	-7.07E-04	7.07E-04	4.997	-7.61E-04	7.61E-04
4.998	-4.78E-04	4.78E-04	4.998	-5.69E-04	5.69E-04	4.998	-6.44E-04	6.44E-04	4.998	-7.07E-04	7.07E-04	4.998	-7.60E-04	7.60E-04
4.999	-4.77E-04	4.77E-04	4.999	-5.69E-04	5.69E-04	4.999	-6.44E-04	6.44E-04	4.999	-7.06E-04	7.06E-04	4.999	-7.60E-04	7.60E-04
5	-4.77E-04	4.77E-04	5	-5.69E-04	5.69E-04	5	-6.43E-04	6.43E-04	5	-7.06E-04	7.06E-04	5	-7.60E-04	7.60E-04
5.001	-4.77E-04	4.77E-04	5.001	-5.68E-04	5.68E-04	5.001	-6.43E-04	6.43E-04	5.001	-7.06E-04	7.06E-04	5.001	-7.59E-04	7.59E-04
5.002	-4.77E-04	4.77E-04	5.002	-5.68E-04	5.68E-04	5.002	-6.43E-04	6.43E-04	5.002	-7.05E-04	7.05E-04	5.002	-7.59E-04	7.59E-04
5.003	-4.77E-04	4.77E-04	5.003	-5.68E-04	5.68E-04	5.003	-6.43E-04	6.43E-04	5.003	-7.05E-04	7.05E-04	5.003	-7.59E-04	7.59E-04
5.004	-4.76E-04	4.76E-04	5.004	-5.68E-04	5.68E-04	5.004	-6.42E-04	6.42E-04	5.004	-7.05E-04	7.05E-04	5.004	-7.59E-04	7.59E-04
5.005	-4.76E-04	4.76E-04	5.005	-5.68E-04	5.68E-04	5.005	-6.42E-04	6.42E-04	5.005	-7.05E-04	7.05E-04	5.005	-7.58E-04	7.58E-04
5.006	-4.76E-04	4.76E-04	5.006	-5.67E-04	5.67E-04	5.006	-6.42E-04	6.42E-04	5.006	-7.04E-04	7.04E-04	5.006	-7.58E-04	7.58E-04
5.007	-4.76E-04	4.76E-04	5.007	-5.67E-04	5.67E-04	5.007	-6.42E-04	6.42E-04	5.007	-7.04E-04	7.04E-04	5.007	-7.58E-04	7.58E-04
5.008	-4.76E-04	4.76E-04	5.008	-5.67E-04	5.67E-04	5.008	-6.41E-04	6.41E-04	5.008	-7.04E-04	7.04E-04	5.008	-7.57E-04	7.57E-04
5.009	-4.75E-04	4.75E-04	5.009	-5.67E-04	5.67E-04	5.009	-6.41E-04	6.41E-04	5.009	-7.04E-04	7.04E-04	5.009	-7.57E-04	7.57E-04
5.01	-4.75E-04	4.75E-04	5.01	-5.66E-04	5.66E-04	5.01	-6.41E-04	6.41E-04	5.01	-7.03E-04	7.03E-04	5.01	-7.57E-04	7.57E-04
5.011	-4.75E-04	4.75E-04	5.011	-5.66E-04	5.66E-04	5.011	-6.41E-04	6.41E-04	5.011	-7.03E-04	7.03E-04	5.011	-7.56E-04	7.56E-04
5.012	-4.75E-04	4.75E-04	5.012	-5.66E-04	5.66E-04	5.012	-6.40E-04	6.40E-04	5.012	-7.03E-04	7.03E-04	5.012	-7.56E-04	7.56E-04
5.013	-4.75E-04	4.75E-04	5.013	-5.66E-04	5.66E-04	5.013	-6.40E-04	6.40E-04	5.013	-7.02E-04	7.02E-04	5.013	-7.56E-04	7.56E-04
5.014	-4.75E-04	4.75E-04	5.014	-5.65E-04	5.65E-04	5.014	-6.40E-04	6.40E-04	5.014	-7.02E-04	7.02E-04	5.014	-7.55E-04	7.55E-04
5.015	-4.74E-04	4.74E-04	5.015	-5.65E-04	5.65E-04	5.015	-6.40E-04	6.40E-04	5.015	-7.02E-04	7.02E-04	5.015	-7.55E-04	7.55E-04
5.016	-4.74E-04	4.74E-04	5.016	-5.65E-04	5.65E-04	5.016	-6.39E-04	6.39E-04	5.016	-7.02E-04	7.02E-04	5.016	-7.55E-04	7.55E-04
5.017	-4.74E-04	4.74E-04	5.017	-5.65E-04	5.65E-04	5.017	-6.39E-04	6.39E-04	5.017	-7.01E-04	7.01E-04	5.017	-7.55E-04	7.55E-04
5.018	-4.74E-04	4.74E-04	5.018	-5.65E-04	5.65E-04	5.018	-6.39E-04	6.39E-04	5.018	-7.01E-04	7.01E-04	5.018	-7.54E-04	7.54E-04
5.019	-4.74E-04	4.74E-04	5.019	-5.64E-04	5.64E-04	5.019	-6.39E-04	6.39E-04	5.019	-7.01E-04	7.01E-04	5.019	-7.54E-04	7.54E-04
5.02	-4.73E-04	4.73E-04	5.02	-5.64E-04	5.64E-04	5.02	-6.38E-04	6.38E-04	5.02	-7.00E-04	7.00E-04	5.02	-7.54E-04	7.54E-04
5.021	-4.73E-04	4.73E-04	5.021	-5.64E-04	5.64E-04	5.021	-6.38E-04	6.38E-04	5.021	-7.00E-04	7.00E-04	5.021	-7.53E-04	7.53E-04
5.022	-4.73E-04	4.73E-04	5.022	-5.64E-04	5.64E-04	5.022	-6.38E-04	6.38E-04	5.022	-7.00E-04	7.00E-04	5.022	-7.53E-04	7.53E-04
5.023	-4.73E-04	4.73E-04	5.023	-5.63E-04	5.63E-04	5.023	-6.38E-04	6.38E-04	5.023	-7.00E-04	7.00E-04	5.023	-7.53E-04	7.53E-04
5.024	-4.73E-04	4.73E-04	5.024	-5.63E-04	5.63E-04	5.024	-6.37E-04	6.37E-04	5.024	-6.99E-04	6.99E-04	5.024	-7.52E-04	7.52E-04
5.025	-4.72E-04	4.72E-04	5.025	-5.63E-04	5.63E-04	5.025	-6.37E-04	6.37E-04	5.025	-6.99E-04	6.99E-04	5.025	-7.52E-04	7.52E-04
5.026	-4.72E-04	4.72E-04	5.026	-5.63E-04	5.63E-04	5.026	-6.37E-04	6.37E-04	5.026	-6.99E-04	6.99E-04	5.026	-7.52E-04	7.52E-04
5.027	-4.72E-04	4.72E-04	5.027	-5.63E-04	5.63E-04	5.027	-6.37E-04	6.37E-04	5.027	-6.98E-04	6.98E-04	5.027	-7.52E-04	7.52E-04
5.028	-4.72E-04	4.72E-04	5.028	-5.62E-04	5.62E-04	5.028	-6.36E-04	6.36E-04	5.028	-6.98E-04	6.98E-04	5.028	-7.51E-04	7.51E-04
5.029	-4.72E-04	4.72E-04	5.029	-5.62E-04	5.62E-04	5.029	-6.36E-04	6.36E-04	5.029	-6.98E-04	6.98E-04	5.029	-7.51E-04	7.51E-04
5.03	-4.72E-04	4.72E-04	5.03	-5.62E-04	5.62E-04	5.03	-6.36E-04	6.36E-04	5.03	-6.98E-04	6.98E-04	5.03	-7.51E-04	7.51E-04
5.031	-4.71E-04	4.71E-04	5.031	-5.62E-04	5.62E-04	5.031	-6.36E-04	6.36E-04	5.031	-6.97E-04	6.97E-04	5.031	-7.50E-04	7.50E-04
5.032	-4.71E-04	4.71E-04	5.032	-5.61E-04	5.61E-04	5.032	-6.35E-04	6.35E-04	5.032	-6.97E-04	6.97E-04	5.032	-7.50E-04	7.50E-04

5.033	-4.71E-04	4.71E-04	5.033	-5.61E-04	5.61E-04	5.033	-6.35E-04	6.35E-04	5.033	-6.97E-04	6.97E-04	5.033	-7.50E-04	7.50E-04
5.034	-4.71E-04	4.71E-04	5.034	-5.61E-04	5.61E-04	5.034	-6.35E-04	6.35E-04	5.034	-6.97E-04	6.97E-04	5.034	-7.49E-04	7.49E-04
5.035	-4.71E-04	4.71E-04	5.035	-5.61E-04	5.61E-04	5.035	-6.34E-04	6.34E-04	5.035	-6.96E-04	6.96E-04	5.035	-7.49E-04	7.49E-04
5.036	-4.70E-04	4.70E-04	5.036	-5.61E-04	5.61E-04	5.036	-6.34E-04	6.34E-04	5.036	-6.96E-04	6.96E-04	5.036	-7.49E-04	7.49E-04
5.037	-4.70E-04	4.70E-04	5.037	-5.60E-04	5.60E-04	5.037	-6.34E-04	6.34E-04	5.037	-6.96E-04	6.96E-04	5.037	-7.49E-04	7.49E-04
5.038	-4.70E-04	4.70E-04	5.038	-5.60E-04	5.60E-04	5.038	-6.34E-04	6.34E-04	5.038	-6.95E-04	6.95E-04	5.038	-7.48E-04	7.48E-04
5.039	-4.70E-04	4.70E-04	5.039	-5.60E-04	5.60E-04	5.039	-6.33E-04	6.33E-04	5.039	-6.95E-04	6.95E-04	5.039	-7.48E-04	7.48E-04
5.04	-4.70E-04	4.70E-04	5.04	-5.60E-04	5.60E-04	5.04	-6.33E-04	6.33E-04	5.04	-6.95E-04	6.95E-04	5.04	-7.48E-04	7.48E-04
5.041	-4.69E-04	4.69E-04	5.041	-5.59E-04	5.59E-04	5.041	-6.33E-04	6.33E-04	5.041	-6.95E-04	6.95E-04	5.041	-7.47E-04	7.47E-04
5.042	-4.69E-04	4.69E-04	5.042	-5.59E-04	5.59E-04	5.042	-6.33E-04	6.33E-04	5.042	-6.94E-04	6.94E-04	5.042	-7.47E-04	7.47E-04
5.043	-4.69E-04	4.69E-04	5.043	-5.59E-04	5.59E-04	5.043	-6.32E-04	6.32E-04	5.043	-6.94E-04	6.94E-04	5.043	-7.47E-04	7.47E-04
5.044	-4.69E-04	4.69E-04	5.044	-5.59E-04	5.59E-04	5.044	-6.32E-04	6.32E-04	5.044	-6.94E-04	6.94E-04	5.044	-7.47E-04	7.47E-04
5.045	-4.69E-04	4.69E-04	5.045	-5.59E-04	5.59E-04	5.045	-6.32E-04	6.32E-04	5.045	-6.94E-04	6.94E-04	5.045	-7.46E-04	7.46E-04
5.046	-4.69E-04	4.69E-04	5.046	-5.58E-04	5.58E-04	5.046	-6.32E-04	6.32E-04	5.046	-6.93E-04	6.93E-04	5.046	-7.46E-04	7.46E-04
5.047	-4.68E-04	4.68E-04	5.047	-5.58E-04	5.58E-04	5.047	-6.31E-04	6.31E-04	5.047	-6.93E-04	6.93E-04	5.047	-7.46E-04	7.46E-04
5.048	-4.68E-04	4.68E-04	5.048	-5.58E-04	5.58E-04	5.048	-6.31E-04	6.31E-04	5.048	-6.93E-04	6.93E-04	5.048	-7.45E-04	7.45E-04
5.049	-4.68E-04	4.68E-04	5.049	-5.58E-04	5.58E-04	5.049	-6.31E-04	6.31E-04	5.049	-6.92E-04	6.92E-04	5.049	-7.45E-04	7.45E-04
5.05	-4.68E-04	4.68E-04	5.05	-5.57E-04	5.57E-04	5.05	-6.31E-04	6.31E-04	5.05	-6.92E-04	6.92E-04	5.05	-7.45E-04	7.45E-04
5.051	-4.68E-04	4.68E-04	5.051	-5.57E-04	5.57E-04	5.051	-6.30E-04	6.30E-04	5.051	-6.92E-04	6.92E-04	5.051	-7.44E-04	7.44E-04
5.052	-4.67E-04	4.67E-04	5.052	-5.57E-04	5.57E-04	5.052	-6.30E-04	6.30E-04	5.052	-6.92E-04	6.92E-04	5.052	-7.44E-04	7.44E-04
5.053	-4.67E-04	4.67E-04	5.053	-5.57E-04	5.57E-04	5.053	-6.30E-04	6.30E-04	5.053	-6.91E-04	6.91E-04	5.053	-7.44E-04	7.44E-04
5.054	-4.67E-04	4.67E-04	5.054	-5.57E-04	5.57E-04	5.054	-6.30E-04	6.30E-04	5.054	-6.91E-04	6.91E-04	5.054	-7.44E-04	7.44E-04
5.055	-4.67E-04	4.67E-04	5.055	-5.56E-04	5.56E-04	5.055	-6.29E-04	6.29E-04	5.055	-6.91E-04	6.91E-04	5.055	-7.43E-04	7.43E-04
5.056	-4.67E-04	4.67E-04	5.056	-5.56E-04	5.56E-04	5.056	-6.29E-04	6.29E-04	5.056	-6.91E-04	6.91E-04	5.056	-7.43E-04	7.43E-04
5.057	-4.66E-04	4.66E-04	5.057	-5.56E-04	5.56E-04	5.057	-6.29E-04	6.29E-04	5.057	-6.90E-04	6.90E-04	5.057	-7.43E-04	7.43E-04
5.058	-4.66E-04	4.66E-04	5.058	-5.56E-04	5.56E-04	5.058	-6.29E-04	6.29E-04	5.058	-6.90E-04	6.90E-04	5.058	-7.42E-04	7.42E-04
5.059	-4.66E-04	4.66E-04	5.059	-5.55E-04	5.55E-04	5.059	-6.28E-04	6.28E-04	5.059	-6.90E-04	6.90E-04	5.059	-7.42E-04	7.42E-04
5.06	-4.66E-04	4.66E-04	5.06	-5.55E-04	5.55E-04	5.06	-6.28E-04	6.28E-04	5.06	-6.89E-04	6.89E-04	5.06	-7.42E-04	7.42E-04
5.061	-4.66E-04	4.66E-04	5.061	-5.55E-04	5.55E-04	5.061	-6.28E-04	6.28E-04	5.061	-6.89E-04	6.89E-04	5.061	-7.42E-04	7.42E-04
5.062	-4.66E-04	4.66E-04	5.062	-5.55E-04	5.55E-04	5.062	-6.28E-04	6.28E-04	5.062	-6.89E-04	6.89E-04	5.062	-7.41E-04	7.41E-04
5.063	-4.65E-04	4.65E-04	5.063	-5.55E-04	5.55E-04	5.063	-6.27E-04	6.27E-04	5.063	-6.89E-04	6.89E-04	5.063	-7.41E-04	7.41E-04
5.064	-4.65E-04	4.65E-04	5.064	-5.54E-04	5.54E-04	5.064	-6.27E-04	6.27E-04	5.064	-6.88E-04	6.88E-04	5.064	-7.41E-04	7.41E-04
5.065	-4.65E-04	4.65E-04	5.065	-5.54E-04	5.54E-04	5.065	-6.27E-04	6.27E-04	5.065	-6.88E-04	6.88E-04	5.065	-7.40E-04	7.40E-04
5.066	-4.65E-04	4.65E-04	5.066	-5.54E-04	5.54E-04	5.066	-6.27E-04	6.27E-04	5.066	-6.88E-04	6.88E-04	5.066	-7.40E-04	7.40E-04
5.067	-4.65E-04	4.65E-04	5.067	-5.54E-04	5.54E-04	5.067	-6.27E-04	6.27E-04	5.067	-6.88E-04	6.88E-04	5.067	-7.40E-04	7.40E-04
5.068	-4.64E-04	4.64E-04	5.068	-5.54E-04	5.54E-04	5.068	-6.26E-04	6.26E-04	5.068	-6.87E-04	6.87E-04	5.068	-7.39E-04	7.39E-04
5.069	-4.64E-04	4.64E-04	5.069	-5.53E-04	5.53E-04	5.069	-6.26E-04	6.26E-04	5.069	-6.87E-04	6.87E-04	5.069	-7.39E-04	7.39E-04
5.07	-4.64E-04	4.64E-04	5.07	-5.53E-04	5.53E-04	5.07	-6.26E-04	6.26E-04	5.07	-6.87E-04	6.87E-04	5.07	-7.39E-04	7.39E-04
5.071	-4.64E-04	4.64E-04	5.071	-5.53E-04	5.53E-04	5.071	-6.26E-04	6.26E-04	5.071	-6.86E-04	6.86E-04	5.071	-7.39E-04	7.39E-04
5.072	-4.64E-04	4.64E-04	5.072	-5.53E-04	5.53E-04	5.072	-6.25E-04	6.25E-04	5.072	-6.86E-04	6.86E-04	5.072	-7.38E-04	7.38E-04
5.073	-4.64E-04	4.64E-04	5.073	-5.52E-04	5.52E-04	5.073	-6.25E-04	6.25E-04	5.073	-6.86E-04	6.86E-04	5.073	-7.38E-04	7.38E-04
5.074	-4.63E-04	4.63E-04	5.074	-5.52E-04	5.52E-04	5.074	-6.25E-04	6.25E-04	5.074	-6.86E-04	6.86E-04	5.074	-7.38E-04	7.38E-04
5.075	-4.63E-04	4.63E-04	5.075	-5.52E-04	5.52E-04	5.075	-6.25E-04	6.25E-04	5.075	-6.85E-04	6.85E-04	5.075	-7.37E-04	7.37E-04
5.076	-4.63E-04	4.63E-04	5.076	-5.52E-04	5.52E-04	5.076	-6.24E-04	6.24E-04	5.076	-6.85E-04	6.85E-04	5.076	-7.37E-04	7.37E-04
5.077	-4.63E-04	4.63E-04	5.077	-5.52E-04	5.52E-04	5.077	-6.24E-04	6.24E-04	5.077	-6.85E-04	6.85E-04	5.077	-7.37E-04	7.37E-04
5.078	-4.63E-04	4.63E-04	5.078	-5.51E-04	5.51E-04	5.078	-6.24E-04	6.24E-04	5.078	-6.85E-04	6.85E-04	5.078	-7.37E-04	7.37E-04
5.079	-4.62E-04	4.62E-04	5.079	-5.51E-04	5.51E-04	5.079	-6.24E-04	6.24E-04	5.079	-6.84E-04	6.84E-04	5.079	-7.36E-04	7.36E-04
5.08	-4.62E-04	4.62E-04	5.08	-5.51E-04	5.51E-04	5.08	-6.23E-04	6.23E-04	5.08	-6.84E-04	6.84E-04	5.08	-7.36E-04	7.36E-04
5.081	-4.62E-04	4.62E-04	5.081	-5.51E-04	5.51E-04	5.081	-6.23E-04	6.23E-04	5.081	-6.84E-04	6.84E-04	5.081	-7.36E-04	7.36E-04
5.082	-4.62E-04	4.62E-04	5.082	-5.50E-04	5.50E-04	5.082	-6.23E-04	6.23E-04	5.082	-6.83E-04	6.83E-04	5.082	-7.35E-04	7.35E-04
5.083	-4.62E-04	4.62E-04	5.083	-5.50E-04	5.50E-04	5.083	-6.23E-04	6.23E-04	5.083	-6.83E-04	6.83E-04	5.083	-7.35E-04	7.35E-04
5.084	-4.62E-04	4.62E-04	5.084	-5.50E-04	5.50E-04	5.084	-6.22E-04	6.22E-04	5.084	-6.83E-04	6.83E-04	5.084	-7.35E-04	7.35E-04
5.085	-4.61E-04	4.61E-04	5.085	-5.50E-04	5.50E-04	5.085	-6.22E-04	6.22E-04	5.085	-6.83E-04	6.83E-04	5.085	-7.35E-04	7.35E-04
5.086	-4.61E-04	4.61E-04	5.086	-5.50E-04	5.50E-04	5.086	-6.22E-04	6.22E-04	5.086	-6.82E-04	6.82E-04	5.086	-7.34E-04	7.34E-04
5.087	-4.61E-04	4.61E-04	5.087	-5.49E-04	5.49E-04	5.087	-6.22E-04	6.22E-04	5.087	-6.82E-04	6.82E-04	5.087	-7.34E-04	7.34E-04
5.088	-4.61E-04	4.61E-04	5.088	-5.49E-04	5.49E-04	5.088	-6.21E-04	6.21E-04	5.088	-6.82E-04	6.82E-04	5.088	-7.34E-04	7.34E-04
5.089	-4.61E-04	4.61E-04	5.089	-5.49E-04	5.49E-04	5.089	-6.21E-04	6.21E-04	5.089	-6.82E-04	6.82E-04	5.089	-7.33E-04	7.33E-04
5.09	-4.60E-04	4.60E-04	5.09	-5.49E-04	5.49E-04	5.09	-6.21E-04	6.21E-04	5.09	-6.81E-04	6.81E-04	5.09	-7.33E-04	7.33E-04
5.091	-4.60E-04	4.60E-04	5.091	-5.49E-04	5.49E-04	5.091	-6.21E-04	6.21E-04	5.091	-6.81E-04	6.81E-04	5.091	-7.33E-04	7.33E-04
5.092	-4.60E-04	4.60E-04	5.092	-5.48E-04	5.48E-04	5.092	-6.20E-04	6.20E-04	5.092	-6.81E-04	6.81E-04	5.092	-7.33E-04	7.33E-04
5.093	-4.60E-04	4.60E-04	5.093	-5.48E-04	5.48E-04	5.093	-6.20E-04	6.20E-04	5.093	-6.81E-04	6.81E-04	5.093	-7.32E-04	7.32E-04
5.094	-4.60E-04	4.60E-04	5.094	-5.48E-04	5.48E-04	5.094	-6.20E-04	6.20E-04	5.094	-6.80E-04	6.80E-04	5.094	-7.32E-04	7.32E-04
5.095	-4.60E-04	4.60E-04	5.095	-5.48E-04	5.48E-04	5.095	-6.20E-04	6.20E-04	5.095	-6.80E-04	6.80E-04	5.095	-7.32E-04	7.32E-04
5.096	-4.59E-04	4.59E-04	5.096	-5.47E-04	5.47E-04	5.096	-6.19E-04	6.19E-04	5.096	-6.80E-04	6.80E-04	5.096	-7.31E-04	7.31E-04
5.097	-4.59E-04	4.59E-04	5.097	-5.47E-04	5.47E-04	5.097	-6.19E-04	6.19E-04	5.097	-6.79E-04	6.79E-04	5.097	-7.31E-04	7.31E-04
5.098	-4.59E-04	4.59E-04	5.098	-5.47E-04	5.47E-04	5.098	-6.19E-04	6.19E-04	5.098	-6.79E-04	6.79E-04	5.098	-7.31E-04	7.31E-04
5.099	-4.59E-04	4.59E-04	5.099	-5.47E-04	5.47E-04	5.099	-6.19E-04	6.19E-04	5.099	-6.79E-04	6.79E-04	5.099	-7.31E-04	7.31E-04
5.1	-4.59E-04	4.59E-04	5.1	-5.47E-04	5.47E-04	5.1	-6.18E-04	6.18E-04	5.1	-6.79E-04	6.79E-04	5.1	-7.30E-04	7.30E-04
5.101	-4.58E-04	4.58E-04	5.101	-5.46E-04	5.46E-04	5.101	-6.18E-04	6.18E-04	5.101	-6.78E-04	6.78E-04	5.101	-7.30E-04	7.30E-04
5.102	-4.58E-04	4.58E-04	5.102	-5.46E-04	5.46E-04	5.102	-6.18E-04	6.18E-04	5.102	-6.78E-04	6.78E-04	5.102	-7.30E-04	7.30E-04
5.103	-4.58E-04	4.58E-04	5.103	-5.46E-04	5.46E-04	5.103	-6.18E-04	6.18E-04	5.103	-6.78E-04	6.78E-04	5.103	-7.29E-04	7.29E-04
5.104	-4.58E-04	4.58E-04	5.104	-5.46E-04	5.46E-04	5.104	-6.17E-04	6.17E-04	5.104	-6.78E-04	6.78E-04	5.104	-7.29E-04	7.29E-04
5.105	-4.58E-04	4.58E-04	5.105	-5.46E-04	5.46E-04	5.105	-6.17E-04	6.17E-04	5.105	-6.77E-04	6.77E-04	5.105	-7.29E-04	7.29E-04
5.106	-4.58E-04	4.58E-04	5.106	-5.45E-04	5.45E-04	5.106	-6.17E-04	6.17E-04	5.106	-6.77E-04	6.77E-04	5.106	-7.29E-04	7.29E-04
5.107	-4.57E-04	4.57E-04	5.107	-5.45E-04	5.45E-04	5.107	-6.17E-04	6.17E-04	5.107	-6.77E-04	6.77E-04	5.107	-7.28E-04	7.28E-04
5.108	-4.57E-04	4.57E-04	5.108	-5.45E-04	5.45E-04	5.108	-6.16E-04	6.16E-04	5.108	-6.77E-04	6.77E-04	5.108	-7.28E-04	7.28E-04
5.109	-4.57E-04	4.57E-04	5.109	-5.45E-04	5.45E-04	5.109	-6.16E-04	6.16E-04	5.109	-6.76E-04	6.76E-04	5.109	-7.28E-04	7.28E-04
5.11	-4.57E-04	4.57E-04	5.11	-5.44E-04	5.44E-04	5.11	-6.16E-04	6.16E-04	5.11	-6.76E-04	6.76E-04	5.11	-7.27E-04	7.27E-04
5.111	-4.57E-04	4.57E-04	5.111	-5.44E-04	5.44E-04	5.111	-6.16E-04	6.16E-04	5.111	-6.76E-04	6.76E-04	5.111	-7.27E-04	7.27E-04
5.112	-4.57E-04	4.57E-04	5.112	-5.44E-04	5.44E-04	5.112	-6.16E-04	6.16E-04	5.112	-6.75E-04	6.75E-04	5.112	-7.27E-04	7.27E-04
5.113	-4.56E-04	4.56E-04	5.113	-5.44E-04	5.44E-04	5.113	-6.15E-04	6.15E-04	5.113	-6.75E-04	6.75E-04	5.113	-7.27E-04	7.27E-04
5.114	-4.56E-04	4.56E-04	5.114	-5.44E-04	5.44E-04	5.114	-6.15E-04	6.15E-04	5.114	-6.75E-04	6.75E-04	5.114	-7.26E-04	7.26E-04
5.115	-4.56E-04	4.56E-04	5.115	-5.43E-04	5.43E-04	5.115	-6.15E-04	6.15E-04	5.115	-6.75E-04	6.75E-04	5.115	-7.26E-04	7.26E-04
5.116	-4.56E-04	4.56E-04	5.116	-5.43E-04	5.43E-04	5.116	-6.15E-04	6.15E-04	5.116	-6.74E-04	6.74E-04	5.116	-7.26E-04	7.26E-04
5.117	-4.56E-04	4.56E-04	5.117	-5.43E-04	5.43E-04	5.117	-6.14E-04	6.14E-04	5.117	-6.74E-04	6.74E-04	5.117	-7.25E-04	7.25E-04
5.118	-4.55E-04	4.55E-04	5.118	-5.43E-04	5.43E-04	5.118	-6.14E-04	6.14E-04	5.118	-6.74E-04	6.74E-04	5.118	-7.25E-04	7.25E-04
5.119	-4.55E-04	4.55E-04	5.119	-5.43E-04	5.43E-04	5.119	-6.14E-04	6.14E-04	5.119	-6.74E-04	6.74E-04	5.119	-7.25E-04	7.25E-04
5.12	-4.55E-04	4.55E-04	5.12	-5.42E-04	5.42E-04	5.12	-6.14E-04	6.14E-04	5.12	-6.73E-04	6.73E-04	5.12	-7.25E-04	7.25E-04
5.121	-4.55E-04	4.55E-04	5.121	-5.42E-04	5.42E-04	5.121	-6.13E-04	6.13E-04	5.121	-6.73E-04	6.73E-04	5.121	-7.24E-04	7.24E-04
5.122	-4.55E-04	4.55E-04	5.122	-5.42E-04	5.42E-04	5.122	-6.13E-04	6.13E-04	5.122	-6.73E-04	6.73E-04	5.122	-7.24E-04	7.24E-04
5.123	-4.55E-04	4.55E-04	5.123	-5.42E-04	5.42E-04	5.123	-6.13E-04	6.13E-04	5.123	-6.73E-04	6.73E-04	5.123	-7.24E-04	7.24E-04
5.124	-4.54E-04	4.54E-04	5.124	-5.41E-04	5.41E-04	5.124	-6.13E-04	6.13E-04	5.124	-6.72E-04	6.72E-04	5.124	-7.23E-04	7.23E-04
5.125	-4.54E-04	4.54E-04	5.125	-5.41E-04	5.41E-04	5.125	-6.12E-04	6.12E-04	5.125	-6.72E-04	6.72E-04	5.125	-7.23E-04	7.23E-04
5.126	-4.54E-04	4.54E-04	5.126	-5.41E-04	5.41E-04	5.126	-6.12E-04	6.12E-04	5.126	-6.72E-04	6.72E-04	5.126	-7.23E-04	7.23E-04
5.127	-4.54E-04	4.54E-04	5.127	-5.41E-04	5.41E-04	5.127	-6.12E-04	6.12E-04	5.127	-6.72E-04	6.72E-04	5.127	-7.23E-04	7.23E-04
5.128	-4.54E-04	4.54E-04	5.128	-5.41E-04	5.41E-04	5.128	-6.12E-04	6.12E-04	5.128	-6.71E-04	6.71E-04	5.128	-7.22E-04	7.22E-04
5.129	-4.53E-04	4.53E-04	5.129	-5.40E-04	5.40E-04	5.129	-6.11E-04	6.11E-04	5.129	-6.71E-04	6.71E-04	5.129	-7.22E-04	7.22E-04
5.13	-4.53E-04	4.53E-04	5.13	-5.40E-04	5.40E-04	5.13	-6.11E-04	6.11E-04	5.13	-6.71E-04	6.71E-04	5.13	-7.22E-04	7.22E-04
5.131	-4.53E-04	4.53E-04	5.131	-5.40E-04	5.40E-04	5.131	-6.11E-04	6.11E-04	5.131	-6.70E-04	6.70E-04	5.131	-7.21E-04	7.21E-04
5.132	-4.53E-04	4.53E-04	5.132	-5.40E-04	5.40E-04	5.132	-6.11E-04	6.11E-04	5.132	-6.70E-04	6.70E-04	5.132	-7.21E-04	7.21E-04
5.133	-4.53E-04	4.53E-04	5.133	-5.40E-04	5.40E-04	5.133	-6.10E-04	6.10E-04	5.133	-6.70E-04	6.70E-04	5.133	-7.21E-04	7.21E-04
5.134	-4.53E-04	4.53E-04	5.134	-5.39E-04	5.39E-04	5.134	-6.10E-04	6.10E-04	5.134	-6.70E-04	6.70E-04	5.134	-7.21E-04	7.21E-04
5.135	-4.52E-04	4.52E-04	5.135	-5.39E-04	5.39E-04	5.135	-6.10E-04	6.10E-04	5.135	-6.69E-04	6.69E-04	5.135	-7.20E-04	7.20E-04
5.136	-4.52E-04	4.52E-04	5.136	-5.39E-04	5.39E-04	5.136	-6.10E-04	6.10E-04	5.136	-6.69E-04	6.69E-04	5.136	-7.20E-04	7.20E-04
5.137	-4.52E-04	4.52E-04	5.137	-5.39E-04	5.39E-04	5.137	-6.10E-04	6.10E-04	5.137	-6.69E-04	6.69E-04	5.137	-7.20E-04	7.20E-04
5.138	-4.52E-04	4.52E-04	5.138	-5.39E-04	5.39E-04	5.138	-6.09E-04	6.09E-04	5.138	-6.69E-04	6.69E-04	5.138	-7.19E-04	7.19E-04
5.139	-4.52E-04	4.52E-04	5.139	-5.38E-04	5.38E-04	5.139	-6.09E-04	6.09E-04	5.139	-6.68E-04	6.68E-04	5.139	-7.19E-04	7.19E-04
5.14	-4.52E-04	4.52E-04	5.14	-5.38E-04	5.38E-04	5.14	-6.09E-04	6.09E-04	5.14	-6.68E-04	6.68E-04	5.14	-7.19E-04	7.19E-04
5.141	-4.51E-04	4.51E-04	5.141	-5.38E-04	5.38E-04	5.141	-6.09E-04	6.09E-04	5.141	-6.68E-04	6.68E-04	5.141	-7.19E-04	7.19E-04
5.142	-4.51E-04	4.51E-04	5.142	-5.38E-04	5.38E-04	5.142	-6.08E-04	6.08E-04	5.142	-6.68E-04	6.68E-04	5.142	-7.18E-04	7.18E-04
5.143	-4.51E-04	4.51E-04	5.143	-5.37E-04	5.37E-04	5.143	-6.08E-04	6.08E-04	5.143	-6.67E-04	6.67E-04	5.143	-7.18E-04	7.18E-04
5.144	-4.51E-04	4.51E-04	5.144	-5.37E-04	5.37E-04	5.144	-6.08E-04	6.08E-04	5.144	-6.67E-04	6.67E-04	5.144	-7.18E-04	7.18E-04
5.145	-4.51E-04	4.51E-04	5.145	-5.37E-04	5.37E-04	5.145	-6.08E-04	6.08E-04	5.145	-6.67E-04	6.67E-04	5.145	-7.17E-04	7.17E-04
5.146	-4.50E-04	4.50E-04	5.146	-5.37E-04	5.37E-04	5.146	-6.07E-04	6.07E-04	5.146	-6.67E-04	6.67E-04	5.146	-7.17E-04	7.17E-04
5.147	-4.50E-04	4.50E-04	5.147	-5.37E-04	5.37E-04	5.147	-6.07E-04	6.07E-04	5.147	-6.66E-04	6.66E-04	5.147	-7.17E-04	7.17E-04
5.148	-4.50E-04	4.50E-04	5.148	-5.36E-04	5.36E-04	5.148	-6.07E-04	6.07E-04	5.148	-6.66E-04	6.66E-04	5.148	-7.17E-04	7.17E-04
5.149	-4.50E-04	4.50E-04	5.149	-5.36E-04	5.36E-04	5.149	-6.07E-04	6.07E-04	5.149	-6.66E-04	6.66E-04	5.149	-7.16E-04	7.16E-04
5.15	-4.50E-04	4.50E-04	5.15	-5.36E-04	5.36E-04	5.15	-6.06E-04	6.06E-04	5.15	-6.66E-04	6.66E-04	5.15	-7.16E-04	7.16E-04
5.151	-4.50E-04	4.50E-04	5.151	-5.36E-04	5.36E-04	5.151	-6.06E-04	6.06E-04	5.151	-6.65E-04	6.65E-04	5.151	-7.16E-04	7.16E-04
5.152	-4.49E-04	4.49E-04	5.152	-5.36E-04	5.36E-04	5.152	-6.06E-04	6.06E-04	5.152	-6.65E-04	6.65E-04	5.152	-7.16E-04	7.16E-04
5.153	-4.49E-04	4.49E-04	5.153	-5.35E-04	5.35E-04	5.153	-6.06E-04	6.06E-04	5.153	-6.65E-04	6.65E-04	5.153	-7.15E-04	7.15E-04
5.154	-4.49E-04	4.49E-04	5.154	-5.35E-04	5.35E-04	5.154	-6.06E-04	6.06E-04	5.154	-6.64E-04	6.64E-04	5.154	-7.15E-04	7.15E-04
5.155	-4.49E-04	4.49E-04	5.155	-5.35E-04	5.35E-04	5.155	-6.05E-04	6.05E-04	5.155	-6.64E-04	6.64E-04	5.155	-7.15E-04	7.15E-04
5.156	-4.49E-04	4.49E-04	5.156	-5.35E-04	5.35E-04	5.156	-6.05E-04	6.05E-04	5.156	-6.64E-04	6.64E-04	5.156	-7.14E-04	7.14E-04
5.157	-4.49E-04	4.49E-04	5.157	-5.35E-04	5.35E-04	5.157	-6.05E-04	6.05E-04	5.157	-6.64E-04	6.64E-04	5.157	-7.14E-04	7.14E-04
5.158	-4.48E-04	4.48E-04	5.158	-5.34E-04	5.34E-04	5.158	-6.05E-04	6.05E-04	5.158	-6.63E-04	6.63E-04	5.158	-7.14E-04	7.14E-04
5.159	-4.48E-04	4.48E-04	5.159	-5.34E-04	5.34E-04	5.159	-6.04E-04	6.04E-04	5.159	-6.63E-04	6.63E-04	5.159	-7.14E-04	7.14E-04
5.16	-4.48E-04	4.48E-04	5.16	-5.34E-04	5.34E-04	5.16	-6.04E-04	6.04E-04	5.16	-6.63E-04	6.63E-04	5.16	-7.13E-04	7.13E-04
5.161	-4.48E-04	4.48E-04	5.161	-5.34E-04	5.34E-04	5.161	-6.04E-04	6.04E-04	5.161	-6.63E-04	6.63E-04	5.161	-7.13E-04	7.13E-04
5.162	-4.48E-04	4.48E-04	5.162	-5.34E-04	5.34E-04	5.162	-6.04E-04	6.04E-04	5.162	-6.62E-04	6.62E-04	5.162	-7.13E-04	7.13E-04
5.163	-4.48E-04	4.48E-04	5.163	-5.33E-04	5.33E-04	5.163	-6.03E-04	6.03E-04	5.163	-6.62E-04	6.62E-04	5.163	-7.13E-04	7.13E-04
5.164	-4.47E-04	4.47E-04	5.164	-5.33E-04	5.33E-04	5.164	-6.03E-04	6.03E-04	5.164	-6.62E-04	6.62E-04	5.164	-7.12E-04	7.12E-04
5.165	-4.47E-04	4.47E-04	5.165	-5.33E-04	5.33E-04	5.165	-6.03E-04	6.03E-04	5.165	-6.62E-04	6.62E-04	5.165	-7.12E-04	7.12E-04
5.166	-4.47E-04	4.47E-04	5.166	-5.33E-04	5.33E-04	5.166	-6.03E-04	6.03E-04	5.166	-6.61E-04	6.61E-04	5.166	-7.12E-04	7.12E-04
5.167	-4.47E-04	4.47E-04	5.167	-5.32E-04	5.32E-04	5.167	-6.02E-04	6.02E-04	5.167	-6.61E-04	6.61E-04	5.167	-7.11E-04	7.11E-04
5.168	-4.47E-04	4.47E-04	5.168	-5.32E-04	5.32E-04	5.168	-6.02E-04	6.02E-04	5.168	-6.61E-04	6.61E-04	5.168	-7.11E-04	7.11E-04
5.169	-4.46E-04	4.46E-04	5.169	-5.32E-04	5.32E-04	5.169	-6.02E-04	6.02E-04	5.169	-6.61E-04	6.61E-04	5.169	-7.11E-04	7.11E-04
5.17	-4.46E-04	4.46E-04	5.17	-5.32E-04	5.32E-04	5.17	-6.02E-04	6.02E-04	5.17	-6.60E-04	6.60E-04	5.17	-7.11E-04	7.11E-04
5.171	-4.46E-04	4.46E-04	5.171	-5.32E-04	5.32E-04	5.171	-6.02E-04	6.02E-04	5.171	-6.60E-04	6.60E-04	5.171	-7.10E-04	7.10E-04
5.172	-4.46E-04	4.46E-04	5.172	-5.31E-04	5.31E-04	5.172	-6.01E-04	6.01E-04	5.172	-6.60E-04	6.60E-04	5.172	-7.10E-04	7.10E-04
5.173	-4.46E-04	4.46E-04	5.173	-5.31E-04	5.31E-04	5.173	-6.01E-04	6.01E-04	5.173	-6.60E-04	6.60E-04	5.173	-7.10E-04	7.10E-04
5.174	-4.46E-04	4.46E-04	5.174	-5.31E-04	5.31E-04	5.174	-6.01E-04	6.01E-04	5.174	-6.59E-04	6.59E-04	5.174	-7.09E-04	7.09E-04
5.175	-4.45E-04	4.45E-04	5.175	-5.31E-04	5.31E-04	5.175	-6.01E-04	6.01E-04	5.175	-6.59E-04	6.59E-04	5.175	-7.09E-04	7.09E-04
5.176	-4.45E-04	4.45E-04	5.176	-5.31E-04	5.31E-04	5.176	-6.00E-04	6.00E-04	5.176	-6.59E-04	6.59E-04	5.176	-7.09E-04	7.09E-04
5.177	-4.45E-04	4.45E-04	5.177	-5.30E-04	5.30E-04	5.177	-6.00E-04	6.00E-04	5.177	-6.59E-04	6.59E-04	5.177	-7.09E-04	7.09E-04
5.178	-4.45E-04	4.45E-04	5.178	-5.30E-04	5.30E-04	5.178	-6.00E-04	6.00E-04	5.178	-6.58E-04	6.58E-04	5.178	-7.08E-04	7.08E-04
5.179	-4.45E-04	4.45E-04	5.179	-5.30E-04	5.30E-04	5.179	-6.00E-04	6.00E-04	5.179	-6.58E-04	6.58E-04	5.179	-7.08E-04	7.08E-04
5.18	-4.45E-04	4.45E-04	5.18	-5.30E-04	5.30E-04	5.18	-5.99E-04	5.99E-04	5.18	-6.58E-04	6.58E-04	5.18	-7.08E-04	7.08E-04
5.181	-4.44E-04	4.44E-04	5.181	-5.30E-04	5.30E-04	5.181	-5.99E-04	5.99E-04	5.181	-6.58E-04	6.58E-04	5.181	-7.08E-04	7.08E-04
5.182	-4.44E-04	4.44E-04	5.182	-5.29E-04	5.29E-04	5.182	-5.99E-04	5.99E-04	5.182	-6.57E-04	6.57E-04	5.182	-7.07E-04	7.07E-04
5.183	-4.44E-04	4.44E-04	5.183	-5.29E-04	5.29E-04	5.183	-5.99E-04	5.99E-04	5.183	-6.57E-04	6.57E-04	5.183	-7.07E-04	7.07E-04
5.184	-4.44E-04	4.44E-04	5.184	-5.29E-04	5.29E-04	5.184	-5.99E-04	5.99E-04	5.184	-6.57E-04	6.57E-04	5.184	-7.07E-04	7.07E-04
5.185	-4.44E-04	4.44E-04	5.185	-5.29E-04	5.29E-04	5.185	-5.98E-04	5.98E-04	5.185	-6.57E-04	6.57E-04	5.185	-7.06E-04	7.06E-04
5.186	-4.44E-04	4.44E-04	5.186	-5.29E-04	5.29E-04	5.186	-5.98E-04	5.98E-04	5.186	-6.56E-04	6.56E-04	5.186	-7.06E-04	7.06E-04
5.187	-4.43E-04	4.43E-04	5.187	-5.28E-04	5.28E-04	5.187	-5.98E-04	5.98E-04	5.187	-6.56E-04	6.56E-04	5.187	-7.06E-04	7.06E-04
5.188	-4.43E-04	4.43E-04	5.188	-5.28E-04	5.28E-04	5.188	-5.98E-04	5.98E-04	5.188	-6.56E-04	6.56E-04	5.188	-7.06E-04	7.06E-04
5.189	-4.43E-04	4.43E-04	5.189	-5.28E-04	5.28E-04	5.189	-5.97E-04	5.97E-04	5.189	-6.56E-04	6.56E-04	5.189	-7.05E-04	7.05E-04
5.19	-4.43E-04	4.43E-04	5.19	-5.28E-04	5.28E-04	5.19	-5.97E-04	5.97E-04	5.19	-6.55E-04	6.55E-04	5.19	-7.05E-04	7.05E-04
5.191	-4.43E-04	4.43E-04	5.191	-5.28E-04	5.28E-04	5.191	-5.97E-04	5.97E-04	5.191	-6.55E-04	6.55E-04	5.191	-7.05E-04	7.05E-04
5.192	-4.43E-04	4.43E-04	5.192	-5.27E-04	5.27E-04	5.192	-5.97E-04	5.97E-04	5.192	-6.55E-04	6.55E-04	5.192	-7.05E-04	7.05E-04
5.193	-4.42E-04	4.42E-04	5.193	-5.27E-04	5.27E-04	5.193	-5.96E-04	5.96E-04	5.193	-6.55E-04	6.55E-04	5.193	-7.04E-04	7.04E-04
5.194	-4.42E-04	4.42E-04	5.194	-5.27E-04	5.27E-04	5.194	-5.96E-04	5.96E-04	5.194	-6.54E-04	6.54E-04	5.194	-7.04E-04	7.04E-04
5.195	-4.42E-04	4.42E-04	5.195	-5.27E-04	5.27E-04	5.195	-5.96E-04	5.96E-04	5.195	-6.54E-04	6.54E-04	5.195	-7.04E-04	7.04E-04
5.196	-4.42E-04	4.42E-04	5.196	-5.27E-04	5.27E-04	5.196	-5.96E-04	5.96E-04	5.196	-6.54E-04	6.54E-04	5.196	-7.03E-04	7.03E-04
5.197	-4.42E-04	4.42E-04	5.197	-5.26E-04	5.26E-04	5.197	-5.96E-04	5.96E-04	5.197	-6.54E-04	6.54E-04	5.197	-7.03E-04	7.03E-04
5.198	-4.42E-04	4.42E-04	5.198	-5.26E-04	5.26E-04	5.198	-5.95E-04	5.95E-04	5.198	-6.53E-04	6.53E-04	5.198	-7.03E-04	7.03E-04
5.199	-4.41E-04	4.41E-04	5.199	-5.26E-04	5.26E-04	5.199	-5.95E-04	5.95E-04	5.199	-6.53E-04	6.53E-04	5.199	-7.03E-04	7.03E-04
5.2	-4.41E-04	4.41E-04	5.2	-5.26E-04	5.26E-04	5.2	-5.95E-04	5.95E-04	5.2	-6.53E-04	6.53E-04	5.2	-7.02E-04	7.02E-04
5.201	-4.41E-04	4.41E-04	5.201	-5.26E-04	5.26E-04	5.201	-5.95E-04	5.95E-04	5.201	-6.53E-04	6.53E-04	5.201	-7.02E-04	7.02E-04
5.202	-4.41E-04	4.41E-04	5.202	-5.25E-04	5.25E-04	5.202	-5.94E-04	5.94E-04	5.202	-6.52E-04	6.52E-04	5.202	-7.02E-04	7.02E-04
5.203	-4.41E-04	4.41E-04	5.203	-5.25E-04	5.25E-04	5.203	-5.94E-04	5.94E-04	5.203	-6.52E-04	6.52E-04	5.203	-7.02E-04	7.02E-04
5.204	-4.41E-04	4.41E-04	5.204	-5.25E-04	5.25E-04	5.204	-5.94E-04	5.94E-04	5.204	-6.52E-04	6.52E-04	5.204	-7.01E-04	7.01E-04
5.205	-4.40E-04	4.40E-04	5.205	-5.25E-04	5.25E-04	5.205	-5.94E-04	5.94E-04	5.205	-6.52E-04	6.52E-04	5.205	-7.01E-04	7.01E-04
5.206	-4.40E-04	4.40E-04	5.206	-5.25E-04	5.25E-04	5.206	-5.93E-04	5.93E-04	5.206	-6.51E-04	6.51E-04	5.206	-7.01E-04	7.01E-04
5.207	-4.40E-04	4.40E-04	5.207	-5.24E-04	5.24E-04	5.207	-5.93E-04	5.93E-04	5.207	-6.51E-04	6.51E-04	5.207	-7.01E-04	7.01E-04
5.208	-4.40E-04	4.40E-04	5.208	-5.24E-04	5.24E-04	5.208	-5.93E-04	5.93E-04	5.208	-6.51E-04	6.51E-04	5.208	-7.00E-04	7.00E-04
5.209	-4.40E-04	4.40E-04	5.209	-5.24E-04	5.24E-04	5.209	-5.93E-04	5.93E-04	5.209	-6.51E-04	6.51E-04	5.209	-7.00E-04	7.00E-04
5.21	-4.39E-04	4.39E-04	5.21	-5.24E-04	5.24E-04	5.21	-5.93E-04	5.93E-04	5.21	-6.50E-04	6.50E-04	5.21	-7.00E-04	7.00E-04
5.211	-4.39E-04	4.39E-04	5.211	-5.24E-04	5.24E-04	5.211	-5.92E-04	5.92E-04	5.211	-6.50E-04	6.50E-04	5.211	-6.99E-04	6.99E-04
5.212	-4.39E-04	4.39E-04	5.212	-5.23E-04	5.23E-04	5.212	-5.92E-04	5.92E-04	5.212	-6.50E-04	6.50E-04	5.212	-6.99E-04	6.99E-04
5.213	-4.39E-04	4.39E-04	5.213	-5.23E-04	5.23E-04	5.213	-5.92E-04	5.92E-04	5.213	-6.50E-04	6.50E-04	5.213	-6.99E-04	6.99E-04
5.214	-4.39E-04	4.39E-04	5.214	-5.23E-04	5.23E-04	5.214	-5.92E-04	5.92E-04	5.214	-6.49E-04	6.49E-04	5.214	-6.99E-04	6.99E-04
5.215	-4.39E-04	4.39E-04	5.215	-5.23E-04	5.23E-04	5.215	-5.91E-04	5.91E-04	5.215	-6.49E-04	6.49E-04	5.215	-6.98E-04	6.98E-04
5.216	-4.38E-04	4.38E-04	5.216	-5.23E-04	5.23E-04	5.216	-5.91E-04	5.91E-04	5.216	-6.49E-04	6.49E-04	5.216	-6.98E-04	6.98E-04
5.217	-4.38E-04	4.38E-04	5.217	-5.22E-04	5.22E-04	5.217	-5.91E-04	5.91E-04	5.217	-6.49E-04	6.49E-04	5.217	-6.98E-04	6.98E-04

5.218	-4.38E-04	4.38E-04	5.218	-5.22E-04	5.22E-04	5.218	-5.91E-04	5.91E-04	5.218	-6.48E-04	6.48E-04	5.218	-6.98E-04	6.98E-04
5.219	-4.38E-04	4.38E-04	5.219	-5.22E-04	5.22E-04	5.219	-5.91E-04	5.91E-04	5.219	-6.48E-04	6.48E-04	5.219	-6.97E-04	6.97E-04
5.22	-4.38E-04	4.38E-04	5.22	-5.22E-04	5.22E-04	5.22	-5.90E-04	5.90E-04	5.22	-6.48E-04	6.48E-04	5.22	-6.97E-04	6.97E-04
5.221	-4.38E-04	4.38E-04	5.221	-5.22E-04	5.22E-04	5.221	-5.90E-04	5.90E-04	5.221	-6.48E-04	6.48E-04	5.221	-6.97E-04	6.97E-04
5.222	-4.37E-04	4.37E-04	5.222	-5.21E-04	5.21E-04	5.222	-5.90E-04	5.90E-04	5.222	-6.47E-04	6.47E-04	5.222	-6.96E-04	6.96E-04
5.223	-4.37E-04	4.37E-04	5.223	-5.21E-04	5.21E-04	5.223	-5.90E-04	5.90E-04	5.223	-6.47E-04	6.47E-04	5.223	-6.96E-04	6.96E-04
5.224	-4.37E-04	4.37E-04	5.224	-5.21E-04	5.21E-04	5.224	-5.89E-04	5.89E-04	5.224	-6.47E-04	6.47E-04	5.224	-6.96E-04	6.96E-04
5.225	-4.37E-04	4.37E-04	5.225	-5.21E-04	5.21E-04	5.225	-5.89E-04	5.89E-04	5.225	-6.47E-04	6.47E-04	5.225	-6.96E-04	6.96E-04
5.226	-4.37E-04	4.37E-04	5.226	-5.21E-04	5.21E-04	5.226	-5.89E-04	5.89E-04	5.226	-6.46E-04	6.46E-04	5.226	-6.95E-04	6.95E-04
5.227	-4.37E-04	4.37E-04	5.227	-5.20E-04	5.20E-04	5.227	-5.89E-04	5.89E-04	5.227	-6.46E-04	6.46E-04	5.227	-6.95E-04	6.95E-04
5.228	-4.36E-04	4.36E-04	5.228	-5.20E-04	5.20E-04	5.228	-5.89E-04	5.89E-04	5.228	-6.46E-04	6.46E-04	5.228	-6.95E-04	6.95E-04
5.229	-4.36E-04	4.36E-04	5.229	-5.20E-04	5.20E-04	5.229	-5.88E-04	5.88E-04	5.229	-6.46E-04	6.46E-04	5.229	-6.95E-04	6.95E-04
5.23	-4.36E-04	4.36E-04	5.23	-5.20E-04	5.20E-04	5.23	-5.88E-04	5.88E-04	5.23	-6.45E-04	6.45E-04	5.23	-6.94E-04	6.94E-04
5.231	-4.36E-04	4.36E-04	5.231	-5.20E-04	5.20E-04	5.231	-5.88E-04	5.88E-04	5.231	-6.45E-04	6.45E-04	5.231	-6.94E-04	6.94E-04
5.232	-4.36E-04	4.36E-04	5.232	-5.19E-04	5.19E-04	5.232	-5.88E-04	5.88E-04	5.232	-6.45E-04	6.45E-04	5.232	-6.94E-04	6.94E-04
5.233	-4.36E-04	4.36E-04	5.233	-5.19E-04	5.19E-04	5.233	-5.87E-04	5.87E-04	5.233	-6.45E-04	6.45E-04	5.233	-6.94E-04	6.94E-04
5.234	-4.35E-04	4.35E-04	5.234	-5.19E-04	5.19E-04	5.234	-5.87E-04	5.87E-04	5.234	-6.44E-04	6.44E-04	5.234	-6.93E-04	6.93E-04
5.235	-4.35E-04	4.35E-04	5.235	-5.19E-04	5.19E-04	5.235	-5.87E-04	5.87E-04	5.235	-6.44E-04	6.44E-04	5.235	-6.93E-04	6.93E-04
5.236	-4.35E-04	4.35E-04	5.236	-5.19E-04	5.19E-04	5.236	-5.87E-04	5.87E-04	5.236	-6.44E-04	6.44E-04	5.236	-6.93E-04	6.93E-04
5.237	-4.35E-04	4.35E-04	5.237	-5.18E-04	5.18E-04	5.237	-5.86E-04	5.86E-04	5.237	-6.44E-04	6.44E-04	5.237	-6.93E-04	6.93E-04
5.238	-4.35E-04	4.35E-04	5.238	-5.18E-04	5.18E-04	5.238	-5.86E-04	5.86E-04	5.238	-6.43E-04	6.43E-04	5.238	-6.92E-04	6.92E-04
5.239	-4.35E-04	4.35E-04	5.239	-5.18E-04	5.18E-04	5.239	-5.86E-04	5.86E-04	5.239	-6.43E-04	6.43E-04	5.239	-6.92E-04	6.92E-04
5.24	-4.34E-04	4.34E-04	5.24	-5.18E-04	5.18E-04	5.24	-5.86E-04	5.86E-04	5.24	-6.43E-04	6.43E-04	5.24	-6.92E-04	6.92E-04
5.241	-4.34E-04	4.34E-04	5.241	-5.18E-04	5.18E-04	5.241	-5.86E-04	5.86E-04	5.241	-6.43E-04	6.43E-04	5.241	-6.91E-04	6.91E-04
5.242	-4.34E-04	4.34E-04	5.242	-5.17E-04	5.17E-04	5.242	-5.85E-04	5.85E-04	5.242	-6.42E-04	6.42E-04	5.242	-6.91E-04	6.91E-04
5.243	-4.34E-04	4.34E-04	5.243	-5.17E-04	5.17E-04	5.243	-5.85E-04	5.85E-04	5.243	-6.42E-04	6.42E-04	5.243	-6.91E-04	6.91E-04
5.244	-4.34E-04	4.34E-04	5.244	-5.17E-04	5.17E-04	5.244	-5.85E-04	5.85E-04	5.244	-6.42E-04	6.42E-04	5.244	-6.91E-04	6.91E-04
5.245	-4.34E-04	4.34E-04	5.245	-5.17E-04	5.17E-04	5.245	-5.85E-04	5.85E-04	5.245	-6.42E-04	6.42E-04	5.245	-6.90E-04	6.90E-04
5.246	-4.33E-04	4.33E-04	5.246	-5.17E-04	5.17E-04	5.246	-5.84E-04	5.84E-04	5.246	-6.41E-04	6.41E-04	5.246	-6.90E-04	6.90E-04
5.247	-4.33E-04	4.33E-04	5.247	-5.16E-04	5.16E-04	5.247	-5.84E-04	5.84E-04	5.247	-6.41E-04	6.41E-04	5.247	-6.90E-04	6.90E-04
5.248	-4.33E-04	4.33E-04	5.248	-5.16E-04	5.16E-04	5.248	-5.84E-04	5.84E-04	5.248	-6.41E-04	6.41E-04	5.248	-6.90E-04	6.90E-04
5.249	-4.33E-04	4.33E-04	5.249	-5.16E-04	5.16E-04	5.249	-5.84E-04	5.84E-04	5.249	-6.41E-04	6.41E-04	5.249	-6.89E-04	6.89E-04
5.25	-4.33E-04	4.33E-04	5.25	-5.16E-04	5.16E-04	5.25	-5.84E-04	5.84E-04	5.25	-6.40E-04	6.40E-04	5.25	-6.89E-04	6.89E-04
5.251	-4.33E-04	4.33E-04	5.251	-5.16E-04	5.16E-04	5.251	-5.83E-04	5.83E-04	5.251	-6.40E-04	6.40E-04	5.251	-6.89E-04	6.89E-04
5.252	-4.32E-04	4.32E-04	5.252	-5.15E-04	5.15E-04	5.252	-5.83E-04	5.83E-04	5.252	-6.40E-04	6.40E-04	5.252	-6.89E-04	6.89E-04
5.253	-4.32E-04	4.32E-04	5.253	-5.15E-04	5.15E-04	5.253	-5.83E-04	5.83E-04	5.253	-6.40E-04	6.40E-04	5.253	-6.88E-04	6.88E-04
5.254	-4.32E-04	4.32E-04	5.254	-5.15E-04	5.15E-04	5.254	-5.83E-04	5.83E-04	5.254	-6.39E-04	6.39E-04	5.254	-6.88E-04	6.88E-04
5.255	-4.32E-04	4.32E-04	5.255	-5.15E-04	5.15E-04	5.255	-5.82E-04	5.82E-04	5.255	-6.39E-04	6.39E-04	5.255	-6.88E-04	6.88E-04
5.256	-4.32E-04	4.32E-04	5.256	-5.15E-04	5.15E-04	5.256	-5.82E-04	5.82E-04	5.256	-6.39E-04	6.39E-04	5.256	-6.88E-04	6.88E-04
5.257	-4.32E-04	4.32E-04	5.257	-5.14E-04	5.14E-04	5.257	-5.82E-04	5.82E-04	5.257	-6.39E-04	6.39E-04	5.257	-6.87E-04	6.87E-04
5.258	-4.32E-04	4.32E-04	5.258	-5.14E-04	5.14E-04	5.258	-5.82E-04	5.82E-04	5.258	-6.38E-04	6.38E-04	5.258	-6.87E-04	6.87E-04
5.259	-4.31E-04	4.31E-04	5.259	-5.14E-04	5.14E-04	5.259	-5.82E-04	5.82E-04	5.259	-6.38E-04	6.38E-04	5.259	-6.87E-04	6.87E-04
5.26	-4.31E-04	4.31E-04	5.26	-5.14E-04	5.14E-04	5.26	-5.81E-04	5.81E-04	5.26	-6.38E-04	6.38E-04	5.26	-6.86E-04	6.86E-04
5.261	-4.31E-04	4.31E-04	5.261	-5.14E-04	5.14E-04	5.261	-5.81E-04	5.81E-04	5.261	-6.38E-04	6.38E-04	5.261	-6.86E-04	6.86E-04
5.262	-4.31E-04	4.31E-04	5.262	-5.13E-04	5.13E-04	5.262	-5.81E-04	5.81E-04	5.262	-6.38E-04	6.38E-04	5.262	-6.86E-04	6.86E-04
5.263	-4.31E-04	4.31E-04	5.263	-5.13E-04	5.13E-04	5.263	-5.81E-04	5.81E-04	5.263	-6.37E-04	6.37E-04	5.263	-6.86E-04	6.86E-04
5.264	-4.31E-04	4.31E-04	5.264	-5.13E-04	5.13E-04	5.264	-5.80E-04	5.80E-04	5.264	-6.37E-04	6.37E-04	5.264	-6.85E-04	6.85E-04
5.265	-4.30E-04	4.30E-04	5.265	-5.13E-04	5.13E-04	5.265	-5.80E-04	5.80E-04	5.265	-6.37E-04	6.37E-04	5.265	-6.85E-04	6.85E-04
5.266	-4.30E-04	4.30E-04	5.266	-5.13E-04	5.13E-04	5.266	-5.80E-04	5.80E-04	5.266	-6.37E-04	6.37E-04	5.266	-6.85E-04	6.85E-04
5.267	-4.30E-04	4.30E-04	5.267	-5.12E-04	5.12E-04	5.267	-5.80E-04	5.80E-04	5.267	-6.36E-04	6.36E-04	5.267	-6.85E-04	6.85E-04
5.268	-4.30E-04	4.30E-04	5.268	-5.12E-04	5.12E-04	5.268	-5.80E-04	5.80E-04	5.268	-6.36E-04	6.36E-04	5.268	-6.84E-04	6.84E-04
5.269	-4.30E-04	4.30E-04	5.269	-5.12E-04	5.12E-04	5.269	-5.79E-04	5.79E-04	5.269	-6.36E-04	6.36E-04	5.269	-6.84E-04	6.84E-04
5.27	-4.30E-04	4.30E-04	5.27	-5.12E-04	5.12E-04	5.27	-5.79E-04	5.79E-04	5.27	-6.36E-04	6.36E-04	5.27	-6.84E-04	6.84E-04
5.271	-4.29E-04	4.29E-04	5.271	-5.12E-04	5.12E-04	5.271	-5.79E-04	5.79E-04	5.271	-6.35E-04	6.35E-04	5.271	-6.84E-04	6.84E-04
5.272	-4.29E-04	4.29E-04	5.272	-5.11E-04	5.11E-04	5.272	-5.79E-04	5.79E-04	5.272	-6.35E-04	6.35E-04	5.272	-6.83E-04	6.83E-04
5.273	-4.29E-04	4.29E-04	5.273	-5.11E-04	5.11E-04	5.273	-5.79E-04	5.79E-04	5.273	-6.35E-04	6.35E-04	5.273	-6.83E-04	6.83E-04
5.274	-4.29E-04	4.29E-04	5.274	-5.11E-04	5.11E-04	5.274	-5.78E-04	5.78E-04	5.274	-6.35E-04	6.35E-04	5.274	-6.83E-04	6.83E-04
5.275	-4.29E-04	4.29E-04	5.275	-5.11E-04	5.11E-04	5.275	-5.78E-04	5.78E-04	5.275	-6.34E-04	6.34E-04	5.275	-6.83E-04	6.83E-04
5.276	-4.29E-04	4.29E-04	5.276	-5.11E-04	5.11E-04	5.276	-5.78E-04	5.78E-04	5.276	-6.34E-04	6.34E-04	5.276	-6.82E-04	6.82E-04
5.277	-4.28E-04	4.28E-04	5.277	-5.11E-04	5.11E-04	5.277	-5.78E-04	5.78E-04	5.277	-6.34E-04	6.34E-04	5.277	-6.82E-04	6.82E-04
5.278	-4.28E-04	4.28E-04	5.278	-5.10E-04	5.10E-04	5.278	-5.77E-04	5.77E-04	5.278	-6.34E-04	6.34E-04	5.278	-6.82E-04	6.82E-04
5.279	-4.28E-04	4.28E-04	5.279	-5.10E-04	5.10E-04	5.279	-5.77E-04	5.77E-04	5.279	-6.33E-04	6.33E-04	5.279	-6.82E-04	6.82E-04
5.28	-4.28E-04	4.28E-04	5.28	-5.10E-04	5.10E-04	5.28	-5.77E-04	5.77E-04	5.28	-6.33E-04	6.33E-04	5.28	-6.81E-04	6.81E-04
5.281	-4.28E-04	4.28E-04	5.281	-5.10E-04	5.10E-04	5.281	-5.77E-04	5.77E-04	5.281	-6.33E-04	6.33E-04	5.281	-6.81E-04	6.81E-04
5.282	-4.28E-04	4.28E-04	5.282	-5.10E-04	5.10E-04	5.282	-5.77E-04	5.77E-04	5.282	-6.33E-04	6.33E-04	5.282	-6.81E-04	6.81E-04
5.283	-4.27E-04	4.27E-04	5.283	-5.09E-04	5.09E-04	5.283	-5.76E-04	5.76E-04	5.283	-6.32E-04	6.32E-04	5.283	-6.81E-04	6.81E-04
5.284	-4.27E-04	4.27E-04	5.284	-5.09E-04	5.09E-04	5.284	-5.76E-04	5.76E-04	5.284	-6.32E-04	6.32E-04	5.284	-6.80E-04	6.80E-04
5.285	-4.27E-04	4.27E-04	5.285	-5.09E-04	5.09E-04	5.285	-5.76E-04	5.76E-04	5.285	-6.32E-04	6.32E-04	5.285	-6.80E-04	6.80E-04
5.286	-4.27E-04	4.27E-04	5.286	-5.09E-04	5.09E-04	5.286	-5.76E-04	5.76E-04	5.286	-6.32E-04	6.32E-04	5.286	-6.80E-04	6.80E-04
5.287	-4.27E-04	4.27E-04	5.287	-5.09E-04	5.09E-04	5.287	-5.75E-04	5.75E-04	5.287	-6.31E-04	6.31E-04	5.287	-6.79E-04	6.79E-04
5.288	-4.27E-04	4.27E-04	5.288	-5.08E-04	5.08E-04	5.288	-5.75E-04	5.75E-04	5.288	-6.31E-04	6.31E-04	5.288	-6.79E-04	6.79E-04
5.289	-4.26E-04	4.26E-04	5.289	-5.08E-04	5.08E-04	5.289	-5.75E-04	5.75E-04	5.289	-6.31E-04	6.31E-04	5.289	-6.79E-04	6.79E-04
5.29	-4.26E-04	4.26E-04	5.29	-5.08E-04	5.08E-04	5.29	-5.75E-04	5.75E-04	5.29	-6.31E-04	6.31E-04	5.29	-6.79E-04	6.79E-04
5.291	-4.26E-04	4.26E-04	5.291	-5.08E-04	5.08E-04	5.291	-5.75E-04	5.75E-04	5.291	-6.31E-04	6.31E-04	5.291	-6.78E-04	6.78E-04
5.292	-4.26E-04	4.26E-04	5.292	-5.08E-04	5.08E-04	5.292	-5.74E-04	5.74E-04	5.292	-6.30E-04	6.30E-04	5.292	-6.78E-04	6.78E-04
5.293	-4.26E-04	4.26E-04	5.293	-5.07E-04	5.07E-04	5.293	-5.74E-04	5.74E-04	5.293	-6.30E-04	6.30E-04	5.293	-6.78E-04	6.78E-04
5.294	-4.26E-04	4.26E-04	5.294	-5.07E-04	5.07E-04	5.294	-5.74E-04	5.74E-04	5.294	-6.30E-04	6.30E-04	5.294	-6.78E-04	6.78E-04
5.295	-4.25E-04	4.25E-04	5.295	-5.07E-04	5.07E-04	5.295	-5.74E-04	5.74E-04	5.295	-6.30E-04	6.30E-04	5.295	-6.77E-04	6.77E-04
5.296	-4.25E-04	4.25E-04	5.296	-5.07E-04	5.07E-04	5.296	-5.73E-04	5.73E-04	5.296	-6.29E-04	6.29E-04	5.296	-6.77E-04	6.77E-04
5.297	-4.25E-04	4.25E-04	5.297	-5.07E-04	5.07E-04	5.297	-5.73E-04	5.73E-04	5.297	-6.29E-04	6.29E-04	5.297	-6.77E-04	6.77E-04
5.298	-4.25E-04	4.25E-04	5.298	-5.06E-04	5.06E-04	5.298	-5.73E-04	5.73E-04	5.298	-6.29E-04	6.29E-04	5.298	-6.77E-04	6.77E-04
5.299	-4.25E-04	4.25E-04	5.299	-5.06E-04	5.06E-04	5.299	-5.73E-04	5.73E-04	5.299	-6.29E-04	6.29E-04	5.299	-6.76E-04	6.76E-04
5.3	-4.25E-04	4.25E-04	5.3	-5.06E-04	5.06E-04	5.3	-5.73E-04	5.73E-04	5.3	-6.28E-04	6.28E-04	5.3	-6.76E-04	6.76E-04
5.301	-4.25E-04	4.25E-04	5.301	-5.06E-04	5.06E-04	5.301	-5.72E-04	5.72E-04	5.301	-6.28E-04	6.28E-04	5.301	-6.76E-04	6.76E-04
5.302	-4.24E-04	4.24E-04	5.302	-5.06E-04	5.06E-04	5.302	-5.72E-04	5.72E-04	5.302	-6.28E-04	6.28E-04	5.302	-6.76E-04	6.76E-04
5.303	-4.24E-04	4.24E-04	5.303	-5.06E-04	5.06E-04	5.303	-5.72E-04	5.72E-04	5.303	-6.28E-04	6.28E-04	5.303	-6.75E-04	6.75E-04
5.304	-4.24E-04	4.24E-04	5.304	-5.05E-04	5.05E-04	5.304	-5.72E-04	5.72E-04	5.304	-6.27E-04	6.27E-04	5.304	-6.75E-04	6.75E-04
5.305	-4.24E-04	4.24E-04	5.305	-5.05E-04	5.05E-04	5.305	-5.72E-04	5.72E-04	5.305	-6.27E-04	6.27E-04	5.305	-6.75E-04	6.75E-04
5.306	-4.24E-04	4.24E-04	5.306	-5.05E-04	5.05E-04	5.306	-5.71E-04	5.71E-04	5.306	-6.27E-04	6.27E-04	5.306	-6.75E-04	6.75E-04
5.307	-4.24E-04	4.24E-04	5.307	-5.05E-04	5.05E-04	5.307	-5.71E-04	5.71E-04	5.307	-6.27E-04	6.27E-04	5.307	-6.74E-04	6.74E-04
5.308	-4.23E-04	4.23E-04	5.308	-5.05E-04	5.05E-04	5.308	-5.71E-04	5.71E-04	5.308	-6.26E-04	6.26E-04	5.308	-6.74E-04	6.74E-04
5.309	-4.23E-04	4.23E-04	5.309	-5.04E-04	5.04E-04	5.309	-5.71E-04	5.71E-04	5.309	-6.26E-04	6.26E-04	5.309	-6.74E-04	6.74E-04
5.31	-4.23E-04	4.23E-04	5.31	-5.04E-04	5.04E-04	5.31	-5.70E-04	5.70E-04	5.31	-6.26E-04	6.26E-04	5.31	-6.74E-04	6.74E-04
5.311	-4.23E-04	4.23E-04	5.311	-5.04E-04	5.04E-04	5.311	-5.70E-04	5.70E-04	5.311	-6.26E-04	6.26E-04	5.311	-6.73E-04	6.73E-04
5.312	-4.23E-04	4.23E-04	5.312	-5.04E-04	5.04E-04	5.312	-5.70E-04	5.70E-04	5.312	-6.26E-04	6.26E-04	5.312	-6.73E-04	6.73E-04
5.313	-4.23E-04	4.23E-04	5.313	-5.04E-04	5.04E-04	5.313	-5.70E-04	5.70E-04	5.313	-6.25E-04	6.25E-04	5.313	-6.73E-04	6.73E-04
5.314	-4.22E-04	4.22E-04	5.314	-5.03E-04	5.03E-04	5.314	-5.70E-04	5.70E-04	5.314	-6.25E-04	6.25E-04	5.314	-6.73E-04	6.73E-04
5.315	-4.22E-04	4.22E-04	5.315	-5.03E-04	5.03E-04	5.315	-5.69E-04	5.69E-04	5.315	-6.25E-04	6.25E-04	5.315	-6.72E-04	6.72E-04
5.316	-4.22E-04	4.22E-04	5.316	-5.03E-04	5.03E-04	5.316	-5.69E-04	5.69E-04	5.316	-6.25E-04	6.25E-04	5.316	-6.72E-04	6.72E-04
5.317	-4.22E-04	4.22E-04	5.317	-5.03E-04	5.03E-04	5.317	-5.69E-04	5.69E-04	5.317	-6.24E-04	6.24E-04	5.317	-6.72E-04	6.72E-04
5.318	-4.22E-04	4.22E-04	5.318	-5.03E-04	5.03E-04	5.318	-5.69E-04	5.69E-04	5.318	-6.24E-04	6.24E-04	5.318	-6.72E-04	6.72E-04
5.319	-4.22E-04	4.22E-04	5.319	-5.02E-04	5.02E-04	5.319	-5.69E-04	5.69E-04	5.319	-6.24E-04	6.24E-04	5.319	-6.71E-04	6.71E-04
5.32	-4.22E-04	4.22E-04	5.32	-5.02E-04	5.02E-04	5.32	-5.68E-04	5.68E-04	5.32	-6.24E-04	6.24E-04	5.32	-6.71E-04	6.71E-04
5.321	-4.21E-04	4.21E-04	5.321	-5.02E-04	5.02E-04	5.321	-5.68E-04	5.68E-04	5.321	-6.23E-04	6.23E-04	5.321	-6.71E-04	6.71E-04
5.322	-4.21E-04	4.21E-04	5.322	-5.02E-04	5.02E-04	5.322	-5.68E-04	5.68E-04	5.322	-6.23E-04	6.23E-04	5.322	-6.71E-04	6.71E-04
5.323	-4.21E-04	4.21E-04	5.323	-5.02E-04	5.02E-04	5.323	-5.68E-04	5.68E-04	5.323	-6.23E-04	6.23E-04	5.323	-6.70E-04	6.70E-04
5.324	-4.21E-04	4.21E-04	5.324	-5.02E-04	5.02E-04	5.324	-5.67E-04	5.67E-04	5.324	-6.23E-04	6.23E-04	5.324	-6.70E-04	6.70E-04
5.325	-4.21E-04	4.21E-04	5.325	-5.01E-04	5.01E-04	5.325	-5.67E-04	5.67E-04	5.325	-6.23E-04	6.23E-04	5.325	-6.70E-04	6.70E-04
5.326	-4.21E-04	4.21E-04	5.326	-5.01E-04	5.01E-04	5.326	-5.67E-04	5.67E-04	5.326	-6.22E-04	6.22E-04	5.326	-6.70E-04	6.70E-04
5.327	-4.20E-04	4.20E-04	5.327	-5.01E-04	5.01E-04	5.327	-5.67E-04	5.67E-04	5.327	-6.22E-04	6.22E-04	5.327	-6.69E-04	6.69E-04
5.328	-4.20E-04	4.20E-04	5.328	-5.01E-04	5.01E-04	5.328	-5.67E-04	5.67E-04	5.328	-6.22E-04	6.22E-04	5.328	-6.69E-04	6.69E-04
5.329	-4.20E-04	4.20E-04	5.329	-5.01E-04	5.01E-04	5.329	-5.66E-04	5.66E-04	5.329	-6.22E-04	6.22E-04	5.329	-6.69E-04	6.69E-04
5.33	-4.20E-04	4.20E-04	5.33	-5.00E-04	5.00E-04	5.33	-5.66E-04	5.66E-04	5.33	-6.21E-04	6.21E-04	5.33	-6.69E-04	6.69E-04
5.331	-4.20E-04	4.20E-04	5.331	-5.00E-04	5.00E-04	5.331	-5.66E-04	5.66E-04	5.331	-6.21E-04	6.21E-04	5.331	-6.68E-04	6.68E-04
5.332	-4.20E-04	4.20E-04	5.332	-5.00E-04	5.00E-04	5.332	-5.66E-04	5.66E-04	5.332	-6.21E-04	6.21E-04	5.332	-6.68E-04	6.68E-04
5.333	-4.19E-04	4.19E-04	5.333	-5.00E-04	5.00E-04	5.333	-5.66E-04	5.66E-04	5.333	-6.21E-04	6.21E-04	5.333	-6.68E-04	6.68E-04
5.334	-4.19E-04	4.19E-04	5.334	-5.00E-04	5.00E-04	5.334	-5.65E-04	5.65E-04	5.334	-6.20E-04	6.20E-04	5.334	-6.68E-04	6.68E-04
5.335	-4.19E-04	4.19E-04	5.335	-4.99E-04	4.99E-04	5.335	-5.65E-04	5.65E-04	5.335	-6.20E-04	6.20E-04	5.335	-6.67E-04	6.67E-04
5.336	-4.19E-04	4.19E-04	5.336	-4.99E-04	4.99E-04	5.336	-5.65E-04	5.65E-04	5.336	-6.20E-04	6.20E-04	5.336	-6.67E-04	6.67E-04
5.337	-4.19E-04	4.19E-04	5.337	-4.99E-04	4.99E-04	5.337	-5.65E-04	5.65E-04	5.337	-6.20E-04	6.20E-04	5.337	-6.67E-04	6.67E-04
5.338	-4.19E-04	4.19E-04	5.338	-4.99E-04	4.99E-04	5.338	-5.65E-04	5.65E-04	5.338	-6.19E-04	6.19E-04	5.338	-6.67E-04	6.67E-04
5.339	-4.19E-04	4.19E-04	5.339	-4.99E-04	4.99E-04	5.339	-5.64E-04	5.64E-04	5.339	-6.19E-04	6.19E-04	5.339	-6.66E-04	6.66E-04
5.34	-4.18E-04	4.18E-04	5.34	-4.99E-04	4.99E-04	5.34	-5.64E-04	5.64E-04	5.34	-6.19E-04	6.19E-04	5.34	-6.66E-04	6.66E-04
5.341	-4.18E-04	4.18E-04	5.341	-4.98E-04	4.98E-04	5.341	-5.64E-04	5.64E-04	5.341	-6.19E-04	6.19E-04	5.341	-6.66E-04	6.66E-04
5.342	-4.18E-04	4.18E-04	5.342	-4.98E-04	4.98E-04	5.342	-5.64E-04	5.64E-04	5.342	-6.19E-04	6.19E-04	5.342	-6.66E-04	6.66E-04
5.343	-4.18E-04	4.18E-04	5.343	-4.98E-04	4.98E-04	5.343	-5.63E-04	5.63E-04	5.343	-6.18E-04	6.18E-04	5.343	-6.65E-04	6.65E-04
5.344	-4.18E-04	4.18E-04	5.344	-4.98E-04	4.98E-04	5.344	-5.63E-04	5.63E-04	5.344	-6.18E-04	6.18E-04	5.344	-6.65E-04	6.65E-04
5.345	-4.18E-04	4.18E-04	5.345	-4.98E-04	4.98E-04	5.345	-5.63E-04	5.63E-04	5.345	-6.18E-04	6.18E-04	5.345	-6.65E-04	6.65E-04
5.346	-4.17E-04	4.17E-04	5.346	-4.97E-04	4.97E-04	5.346	-5.63E-04	5.63E-04	5.346	-6.18E-04	6.18E-04	5.346	-6.65E-04	6.65E-04
5.347	-4.17E-04	4.17E-04	5.347	-4.97E-04	4.97E-04	5.347	-5.63E-04	5.63E-04	5.347	-6.17E-04	6.17E-04	5.347	-6.64E-04	6.64E-04
5.348	-4.17E-04	4.17E-04	5.348	-4.97E-04	4.97E-04	5.348	-5.62E-04	5.62E-04	5.348	-6.17E-04	6.17E-04	5.348	-6.64E-04	6.64E-04
5.349	-4.17E-04	4.17E-04	5.349	-4.97E-04	4.97E-04	5.349	-5.62E-04	5.62E-04	5.349	-6.17E-04	6.17E-04	5.349	-6.64E-04	6.64E-04
5.35	-4.17E-04	4.17E-04	5.35	-4.97E-04	4.97E-04	5.35	-5.62E-04	5.62E-04	5.35	-6.17E-04	6.17E-04	5.35	-6.64E-04	6.64E-04
5.351	-4.17E-04	4.17E-04	5.351	-4.97E-04	4.97E-04	5.351	-5.62E-04	5.62E-04	5.351	-6.16E-04	6.16E-04	5.351	-6.63E-04	6.63E-04
5.352	-4.16E-04	4.16E-04	5.352	-4.96E-04	4.96E-04	5.352	-5.62E-04	5.62E-04	5.352	-6.16E-04	6.16E-04	5.352	-6.63E-04	6.63E-04
5.353	-4.16E-04	4.16E-04	5.353	-4.96E-04	4.96E-04	5.353	-5.61E-04	5.61E-04	5.353	-6.16E-04	6.16E-04	5.353	-6.63E-04	6.63E-04
5.354	-4.16E-04	4.16E-04	5.354	-4.96E-04	4.96E-04	5.354	-5.61E-04	5.61E-04	5.354	-6.16E-04	6.16E-04	5.354	-6.63E-04	6.63E-04
5.355	-4.16E-04	4.16E-04	5.355	-4.96E-04	4.96E-04	5.355	-5.61E-04	5.61E-04	5.355	-6.16E-04	6.16E-04	5.355	-6.62E-04	6.62E-04
5.356	-4.16E-04	4.16E-04	5.356	-4.96E-04	4.96E-04	5.356	-5.61E-04	5.61E-04	5.356	-6.15E-04	6.15E-04	5.356	-6.62E-04	6.62E-04
5.357	-4.16E-04	4.16E-04	5.357	-4.95E-04	4.95E-04	5.357	-5.61E-04	5.61E-04	5.357	-6.15E-04	6.15E-04	5.357	-6.62E-04	6.62E-04
5.358	-4.16E-04	4.16E-04	5.358	-4.95E-04	4.95E-04	5.358	-5.60E-04	5.60E-04	5.358	-6.15E-04	6.15E-04	5.358	-6.62E-04	6.62E-04
5.359	-4.15E-04	4.15E-04	5.359	-4.95E-04	4.95E-04	5.359	-5.60E-04	5.60E-04	5.359	-6.15E-04	6.15E-04	5.359	-6.61E-04	6.61E-04
5.36	-4.15E-04	4.15E-04	5.36	-4.95E-04	4.95E-04	5.36	-5.60E-04	5.60E-04	5.36	-6.14E-04	6.14E-04	5.36	-6.61E-04	6.61E-04
5.361	-4.15E-04	4.15E-04	5.361	-4.95E-04	4.95E-04	5.361	-5.60E-04	5.60E-04	5.361	-6.14E-04	6.14E-04	5.361	-6.61E-04	6.61E-04
5.362	-4.15E-04	4.15E-04	5.362	-4.94E-04	4.94E-04	5.362	-5.59E-04	5.59E-04	5.362	-6.14E-04	6.14E-04	5.362	-6.61E-04	6.61E-04
5.363	-4.15E-04	4.15E-04	5.363	-4.94E-04	4.94E-04	5.363	-5.59E-04	5.59E-04	5.363	-6.14E-04	6.14E-04	5.363	-6.60E-04	6.60E-04
5.364	-4.15E-04	4.15E-04	5.364	-4.94E-04	4.94E-04	5.364	-5.59E-04	5.59E-04	5.364	-6.13E-04	6.13E-04	5.364	-6.60E-04	6.60E-04
5.365	-4.14E-04	4.14E-04	5.365	-4.94E-04	4.94E-04	5.365	-5.59E-04	5.59E-04	5.365	-6.13E-04	6.13E-04	5.365	-6.60E-04	6.60E-04
5.366	-4.14E-04	4.14E-04	5.366	-4.94E-04	4.94E-04	5.366	-5.59E-04	5.59E-04	5.366	-6.13E-04	6.13E-04	5.366	-6.60E-04	6.60E-04
5.367	-4.14E-04	4.14E-04	5.367	-4.94E-04	4.94E-04	5.367	-5.58E-04	5.58E-04	5.367	-6.13E-04	6.13E-04	5.367	-6.59E-04	6.59E-04
5.368	-4.14E-04	4.14E-04	5.368	-4.93E-04	4.93E-04	5.368	-5.58E-04	5.58E-04	5.368	-6.13E-04	6.13E-04	5.368	-6.59E-04	6.59E-04
5.369	-4.14E-04	4.14E-04	5.369	-4.93E-04	4.93E-04	5.369	-5.58E-04	5.58E-04	5.369	-6.12E-04	6.12E-04	5.369	-6.59E-04	6.59E-04
5.37	-4.14E-04	4.14E-04	5.37	-4.93E-04	4.93E-04	5.37	-5.58E-04	5.58E-04	5.37	-6.12E-04	6.12E-04	5.37	-6.59E-04	6.59E-04
5.371	-4.14E-04	4.14E-04	5.371	-4.93E-04	4.93E-04	5.371	-5.58E-04	5.58E-04	5.371	-6.12E-04	6.12E-04	5.371	-6.58E-04	6.58E-04
5.372	-4.13E-04	4.13E-04	5.372	-4.93E-04	4.93E-04	5.372	-5.57E-04	5.57E-04	5.372	-6.12E-04	6.12E-04	5.372	-6.58E-04	6.58E-04
5.373	-4.13E-04	4.13E-04	5.373	-4.92E-04	4.92E-04	5.373	-5.57E-04	5.57E-04	5.373	-6.11E-04	6.11E-04	5.373	-6.58E-04	6.58E-04
5.374	-4.13E-04	4.13E-04	5.374	-4.92E-04	4.92E-04	5.374	-5.57E-04	5.57E-04	5.374	-6.11E-04	6.11E-04	5.374	-6.58E-04	6.58E-04
5.375	-4.13E-04	4.13E-04	5.375	-4.92E-04	4.92E-04	5.375	-5.57E-04	5.57E-04	5.375	-6.11E-04	6.11E-04	5.375	-6.57E-04	6.57E-04
5.376	-4.13E-04	4.13E-04	5.376	-4.92E-04	4.92E-04	5.376	-5.57E-04	5.57E-04	5.376	-6.11E-04	6.11E-04	5.376	-6.57E-04	6.57E-04
5.377	-4.13E-04	4.13E-04	5.377	-4.92E-04	4.92E-04	5.377	-5.56E-04	5.56E-04	5.377	-6.11E-04	6.11E-04	5.377	-6.57E-04	6.57E-04
5.378	-4.12E-04	4.12E-04	5.378	-4.92E-04	4.92E-04	5.378	-5.56E-04	5.56E-04	5.378	-6.10E-04	6.10E-04	5.378	-6.57E-04	6.57E-04
5.379	-4.12E-04	4.12E-04	5.379	-4.91E-04	4.91E-04	5.379	-5.56E-04	5.56E-04	5.379	-6.10E-04	6.10E-04	5.379	-6.56E-04	6.56E-04
5.38	-4.12E-04	4.12E-04	5.38	-4.91E-04	4.91E-04	5.38	-5.56E-04	5.56E-04	5.38	-6.10E-04	6.10E-04	5.38	-6.56E-04	6.56E-04
5.381	-4.12E-04	4.12E-04	5.381	-4.91E-04	4.91E-04	5.381	-5.56E-04	5.56E-04	5.381	-6.10E-04	6.10E-04	5.381	-6.56E-04	6.56E-04
5.382	-4.12E-04	4.12E-04	5.382	-4.91E-04	4.91E-04	5.382	-5.55E-04	5.55E-04	5.382	-6.09E-04	6.09E-04	5.382	-6.56E-04	6.56E-04
5.383	-4.12E-04	4.12E-04	5.383	-4.91E-04	4.91E-04	5.383	-5.55E-04	5.55E-04	5.383	-6.09E-04	6.09E-04	5.383	-6.55E-04	6.55E-04
5.384	-4.12E-04	4.12E-04	5.384	-4.90E-04	4.90E-04	5.384	-5.55E-04	5.55E-04	5.384	-6.09E-04	6.09E-04	5.384	-6.55E-04	6.55E-04
5.385	-4.11E-04	4.11E-04	5.385	-4.90E-04	4.90E-04	5.385	-5.55E-04	5.55E-04	5.385	-6.09E-04	6.09E-04	5.385	-6.55E-04	6.55E-04
5.386	-4.11E-04	4.11E-04	5.386	-4.90E-04	4.90E-04	5.386	-5.54E-04	5.54E-04	5.386	-6.08E-04	6.08E-04	5.386	-6.55E-04	6.55E-04
5.387	-4.11E-04	4.11E-04	5.387	-4.90E-04	4.90E-04	5.387	-5.54E-04	5.54E-04	5.387	-6.08E-04	6.08E-04	5.387	-6.54E-04	6.54E-04
5.388	-4.11E-04	4.11E-04	5.388	-4.90E-04	4.90E-04	5.388	-5.54E-04	5.54E-04	5.388	-6.08E-04	6.08E-04	5.388	-6.54E-04	6.54E-04
5.389	-4.11E-04	4.11E-04	5.389	-4.90E-04	4.90E-04	5.389	-5.54E-04	5.54E-04	5.389	-6.08E-04	6.08E-04	5.389	-6.54E-04	6.54E-04
5.39	-4.11E-04	4.11E-04	5.39	-4.89E-04	4.89E-04	5.39	-5.54E-04	5.54E-04	5.39	-6.08E-04	6.08E-04	5.39	-6.54E-04	6.54E-04
5.391	-4.10E-04	4.10E-04	5.391	-4.89E-04	4.89E-04	5.391	-5.53E-04	5.53E-04	5.391	-6.07E-04	6.07E-04	5.391	-6.54E-04	6.54E-04
5.392	-4.10E-04	4.10E-04	5.392	-4.89E-04	4.89E-04	5.392	-5.53E-04	5.53E-04	5.392	-6.07E-04	6.07E-04	5.392	-6.53E-04	6.53E-04
5.393	-4.10E-04	4.10E-04	5.393	-4.89E-04	4.89E-04	5.393	-5.53E-04	5.53E-04	5.393	-6.07E-04	6.07E-04	5.393	-6.53E-04	6.53E-04
5.394	-4.10E-04	4.10E-04	5.394	-4.89E-04	4.89E-04	5.394	-5.53E-04	5.53E-04	5.394	-6.07E-04	6.07E-04	5.394	-6.53E-04	6.53E-04
5.395	-4.10E-04	4.10E-04	5.395	-4.88E-04	4.88E-04	5.395	-5.53E-04	5.53E-04	5.395	-6.06E-04	6.06E-04	5.395	-6.53E-04	6.53E-04
5.396	-4.10E-04	4.10E-04	5.396	-4.88E-04	4.88E-04	5.396	-5.52E-04	5.52E-04	5.396	-6.06E-04	6.06E-04	5.396	-6.52E-04	6.52E-04
5.397	-4.10E-04	4.10E-04	5.397	-4.88E-04	4.88E-04	5.397	-5.52E-04	5.52E-04	5.397	-6.06E-04	6.06E-04	5.397	-6.52E-04	6.52E-04
5.398	-4.09E-04	4.09E-04	5.398	-4.88E-04	4.88E-04	5.398	-5.52E-04	5.52E-04	5.398	-6.06E-04	6.06E-04	5.398	-6.52E-04	6.52E-04
5.399	-4.09E-04	4.09E-04	5.399	-4.88E-04	4.88E-04	5.399	-5.52E-04	5.52E-04	5.399	-6.06E-04	6.06E-04	5.399	-6.52E-04	6.52E-04
5.4	-4.09E-04	4.09E-04	5.4	-4.88E-04	4.88E-04	5.4	-5.52E-04	5.52E-04	5.4	-6.05E-04	6.05E-04	5.4	-6.51E-04	6.51E-04
5.401	-4.09E-04	4.09E-04	5.401	-4.87E-04	4.87E-04	5.401	-5.51E-04	5.51E-04	5.401	-6.05E-04	6.05E-04	5.401	-6.51E-04	6.51E-04
5.402	-4.09E-04	4.09E-04	5.402	-4.87E-04	4.87E-04	5.402	-5.51E-04	5.51E-04	5.402	-6.05E-04	6.05E-04	5.402	-6.51E-04	6.51E-04

5.403	-4.09E-04	4.09E-04	5.403	-4.87E-04	4.87E-04	5.403	-5.51E-04	5.51E-04	5.403	-6.05E-04	6.05E-04	5.403	-6.51E-04	6.51E-04
5.404	-4.09E-04	4.09E-04	5.404	-4.87E-04	4.87E-04	5.404	-5.51E-04	5.51E-04	5.404	-6.04E-04	6.04E-04	5.404	-6.50E-04	6.50E-04
5.405	-4.08E-04	4.08E-04	5.405	-4.87E-04	4.87E-04	5.405	-5.51E-04	5.51E-04	5.405	-6.04E-04	6.04E-04	5.405	-6.50E-04	6.50E-04
5.406	-4.08E-04	4.08E-04	5.406	-4.86E-04	4.86E-04	5.406	-5.50E-04	5.50E-04	5.406	-6.04E-04	6.04E-04	5.406	-6.50E-04	6.50E-04
5.407	-4.08E-04	4.08E-04	5.407	-4.86E-04	4.86E-04	5.407	-5.50E-04	5.50E-04	5.407	-6.04E-04	6.04E-04	5.407	-6.50E-04	6.50E-04
5.408	-4.08E-04	4.08E-04	5.408	-4.86E-04	4.86E-04	5.408	-5.50E-04	5.50E-04	5.408	-6.04E-04	6.04E-04	5.408	-6.49E-04	6.49E-04
5.409	-4.08E-04	4.08E-04	5.409	-4.86E-04	4.86E-04	5.409	-5.50E-04	5.50E-04	5.409	-6.03E-04	6.03E-04	5.409	-6.49E-04	6.49E-04
5.41	-4.08E-04	4.08E-04	5.41	-4.86E-04	4.86E-04	5.41	-5.50E-04	5.50E-04	5.41	-6.03E-04	6.03E-04	5.41	-6.49E-04	6.49E-04
5.411	-4.07E-04	4.07E-04	5.411	-4.86E-04	4.86E-04	5.411	-5.49E-04	5.49E-04	5.411	-6.03E-04	6.03E-04	5.411	-6.49E-04	6.49E-04
5.412	-4.07E-04	4.07E-04	5.412	-4.85E-04	4.85E-04	5.412	-5.49E-04	5.49E-04	5.412	-6.03E-04	6.03E-04	5.412	-6.48E-04	6.48E-04
5.413	-4.07E-04	4.07E-04	5.413	-4.85E-04	4.85E-04	5.413	-5.49E-04	5.49E-04	5.413	-6.02E-04	6.02E-04	5.413	-6.48E-04	6.48E-04
5.414	-4.07E-04	4.07E-04	5.414	-4.85E-04	4.85E-04	5.414	-5.49E-04	5.49E-04	5.414	-6.02E-04	6.02E-04	5.414	-6.48E-04	6.48E-04
5.415	-4.07E-04	4.07E-04	5.415	-4.85E-04	4.85E-04	5.415	-5.49E-04	5.49E-04	5.415	-6.02E-04	6.02E-04	5.415	-6.48E-04	6.48E-04
5.416	-4.07E-04	4.07E-04	5.416	-4.85E-04	4.85E-04	5.416	-5.48E-04	5.48E-04	5.416	-6.02E-04	6.02E-04	5.416	-6.47E-04	6.47E-04
5.417	-4.07E-04	4.07E-04	5.417	-4.84E-04	4.84E-04	5.417	-5.48E-04	5.48E-04	5.417	-6.02E-04	6.02E-04	5.417	-6.47E-04	6.47E-04
5.418	-4.06E-04	4.06E-04	5.418	-4.84E-04	4.84E-04	5.418	-5.48E-04	5.48E-04	5.418	-6.01E-04	6.01E-04	5.418	-6.47E-04	6.47E-04
5.419	-4.06E-04	4.06E-04	5.419	-4.84E-04	4.84E-04	5.419	-5.48E-04	5.48E-04	5.419	-6.01E-04	6.01E-04	5.419	-6.47E-04	6.47E-04
5.42	-4.06E-04	4.06E-04	5.42	-4.84E-04	4.84E-04	5.42	-5.48E-04	5.48E-04	5.42	-6.01E-04	6.01E-04	5.42	-6.47E-04	6.47E-04
5.421	-4.06E-04	4.06E-04	5.421	-4.84E-04	4.84E-04	5.421	-5.47E-04	5.47E-04	5.421	-6.01E-04	6.01E-04	5.421	-6.46E-04	6.46E-04
5.422	-4.06E-04	4.06E-04	5.422	-4.84E-04	4.84E-04	5.422	-5.47E-04	5.47E-04	5.422	-6.00E-04	6.00E-04	5.422	-6.46E-04	6.46E-04
5.423	-4.06E-04	4.06E-04	5.423	-4.83E-04	4.83E-04	5.423	-5.47E-04	5.47E-04	5.423	-6.00E-04	6.00E-04	5.423	-6.46E-04	6.46E-04
5.424	-4.06E-04	4.06E-04	5.424	-4.83E-04	4.83E-04	5.424	-5.47E-04	5.47E-04	5.424	-6.00E-04	6.00E-04	5.424	-6.46E-04	6.46E-04
5.425	-4.05E-04	4.05E-04	5.425	-4.83E-04	4.83E-04	5.425	-5.47E-04	5.47E-04	5.425	-6.00E-04	6.00E-04	5.425	-6.45E-04	6.45E-04
5.426	-4.05E-04	4.05E-04	5.426	-4.83E-04	4.83E-04	5.426	-5.46E-04	5.46E-04	5.426	-6.00E-04	6.00E-04	5.426	-6.45E-04	6.45E-04
5.427	-4.05E-04	4.05E-04	5.427	-4.83E-04	4.83E-04	5.427	-5.46E-04	5.46E-04	5.427	-5.99E-04	5.99E-04	5.427	-6.45E-04	6.45E-04
5.428	-4.05E-04	4.05E-04	5.428	-4.83E-04	4.83E-04	5.428	-5.46E-04	5.46E-04	5.428	-5.99E-04	5.99E-04	5.428	-6.45E-04	6.45E-04
5.429	-4.05E-04	4.05E-04	5.429	-4.82E-04	4.82E-04	5.429	-5.46E-04	5.46E-04	5.429	-5.99E-04	5.99E-04	5.429	-6.44E-04	6.44E-04
5.43	-4.05E-04	4.05E-04	5.43	-4.82E-04	4.82E-04	5.43	-5.46E-04	5.46E-04	5.43	-5.99E-04	5.99E-04	5.43	-6.44E-04	6.44E-04
5.431	-4.04E-04	4.04E-04	5.431	-4.82E-04	4.82E-04	5.431	-5.45E-04	5.45E-04	5.431	-5.98E-04	5.98E-04	5.431	-6.44E-04	6.44E-04
5.432	-4.04E-04	4.04E-04	5.432	-4.82E-04	4.82E-04	5.432	-5.45E-04	5.45E-04	5.432	-5.98E-04	5.98E-04	5.432	-6.44E-04	6.44E-04
5.433	-4.04E-04	4.04E-04	5.433	-4.82E-04	4.82E-04	5.433	-5.45E-04	5.45E-04	5.433	-5.98E-04	5.98E-04	5.433	-6.43E-04	6.43E-04
5.434	-4.04E-04	4.04E-04	5.434	-4.81E-04	4.81E-04	5.434	-5.45E-04	5.45E-04	5.434	-5.98E-04	5.98E-04	5.434	-6.43E-04	6.43E-04
5.435	-4.04E-04	4.04E-04	5.435	-4.81E-04	4.81E-04	5.435	-5.45E-04	5.45E-04	5.435	-5.98E-04	5.98E-04	5.435	-6.43E-04	6.43E-04
5.436	-4.04E-04	4.04E-04	5.436	-4.81E-04	4.81E-04	5.436	-5.44E-04	5.44E-04	5.436	-5.97E-04	5.97E-04	5.436	-6.43E-04	6.43E-04
5.437	-4.04E-04	4.04E-04	5.437	-4.81E-04	4.81E-04	5.437	-5.44E-04	5.44E-04	5.437	-5.97E-04	5.97E-04	5.437	-6.42E-04	6.42E-04
5.438	-4.03E-04	4.03E-04	5.438	-4.81E-04	4.81E-04	5.438	-5.44E-04	5.44E-04	5.438	-5.97E-04	5.97E-04	5.438	-6.42E-04	6.42E-04
5.439	-4.03E-04	4.03E-04	5.439	-4.81E-04	4.81E-04	5.439	-5.44E-04	5.44E-04	5.439	-5.97E-04	5.97E-04	5.439	-6.42E-04	6.42E-04
5.44	-4.03E-04	4.03E-04	5.44	-4.80E-04	4.80E-04	5.44	-5.44E-04	5.44E-04	5.44	-5.96E-04	5.96E-04	5.44	-6.42E-04	6.42E-04
5.441	-4.03E-04	4.03E-04	5.441	-4.80E-04	4.80E-04	5.441	-5.43E-04	5.43E-04	5.441	-5.96E-04	5.96E-04	5.441	-6.42E-04	6.42E-04
5.442	-4.03E-04	4.03E-04	5.442	-4.80E-04	4.80E-04	5.442	-5.43E-04	5.43E-04	5.442	-5.96E-04	5.96E-04	5.442	-6.41E-04	6.41E-04
5.443	-4.03E-04	4.03E-04	5.443	-4.80E-04	4.80E-04	5.443	-5.43E-04	5.43E-04	5.443	-5.96E-04	5.96E-04	5.443	-6.41E-04	6.41E-04
5.444	-4.03E-04	4.03E-04	5.444	-4.80E-04	4.80E-04	5.444	-5.43E-04	5.43E-04	5.444	-5.96E-04	5.96E-04	5.444	-6.41E-04	6.41E-04
5.445	-4.02E-04	4.02E-04	5.445	-4.80E-04	4.80E-04	5.445	-5.43E-04	5.43E-04	5.445	-5.95E-04	5.95E-04	5.445	-6.41E-04	6.41E-04
5.446	-4.02E-04	4.02E-04	5.446	-4.79E-04	4.79E-04	5.446	-5.42E-04	5.42E-04	5.446	-5.95E-04	5.95E-04	5.446	-6.40E-04	6.40E-04
5.447	-4.02E-04	4.02E-04	5.447	-4.79E-04	4.79E-04	5.447	-5.42E-04	5.42E-04	5.447	-5.95E-04	5.95E-04	5.447	-6.40E-04	6.40E-04
5.448	-4.02E-04	4.02E-04	5.448	-4.79E-04	4.79E-04	5.448	-5.42E-04	5.42E-04	5.448	-5.95E-04	5.95E-04	5.448	-6.40E-04	6.40E-04
5.449	-4.02E-04	4.02E-04	5.449	-4.79E-04	4.79E-04	5.449	-5.42E-04	5.42E-04	5.449	-5.94E-04	5.94E-04	5.449	-6.40E-04	6.40E-04
5.45	-4.02E-04	4.02E-04	5.45	-4.79E-04	4.79E-04	5.45	-5.42E-04	5.42E-04	5.45	-5.94E-04	5.94E-04	5.45	-6.39E-04	6.39E-04
5.451	-4.01E-04	4.01E-04	5.451	-4.78E-04	4.78E-04	5.451	-5.41E-04	5.41E-04	5.451	-5.94E-04	5.94E-04	5.451	-6.39E-04	6.39E-04
5.452	-4.01E-04	4.01E-04	5.452	-4.78E-04	4.78E-04	5.452	-5.41E-04	5.41E-04	5.452	-5.94E-04	5.94E-04	5.452	-6.39E-04	6.39E-04
5.453	-4.01E-04	4.01E-04	5.453	-4.78E-04	4.78E-04	5.453	-5.41E-04	5.41E-04	5.453	-5.94E-04	5.94E-04	5.453	-6.39E-04	6.39E-04
5.454	-4.01E-04	4.01E-04	5.454	-4.78E-04	4.78E-04	5.454	-5.41E-04	5.41E-04	5.454	-5.93E-04	5.93E-04	5.454	-6.39E-04	6.39E-04
5.455	-4.01E-04	4.01E-04	5.455	-4.78E-04	4.78E-04	5.455	-5.41E-04	5.41E-04	5.455	-5.93E-04	5.93E-04	5.455	-6.38E-04	6.38E-04
5.456	-4.01E-04	4.01E-04	5.456	-4.78E-04	4.78E-04	5.456	-5.40E-04	5.40E-04	5.456	-5.93E-04	5.93E-04	5.456	-6.38E-04	6.38E-04
5.457	-4.01E-04	4.01E-04	5.457	-4.77E-04	4.77E-04	5.457	-5.40E-04	5.40E-04	5.457	-5.93E-04	5.93E-04	5.457	-6.38E-04	6.38E-04
5.458	-4.00E-04	4.00E-04	5.458	-4.77E-04	4.77E-04	5.458	-5.40E-04	5.40E-04	5.458	-5.93E-04	5.93E-04	5.458	-6.38E-04	6.38E-04
5.459	-4.00E-04	4.00E-04	5.459	-4.77E-04	4.77E-04	5.459	-5.40E-04	5.40E-04	5.459	-5.92E-04	5.92E-04	5.459	-6.37E-04	6.37E-04
5.46	-4.00E-04	4.00E-04	5.46	-4.77E-04	4.77E-04	5.46	-5.40E-04	5.40E-04	5.46	-5.92E-04	5.92E-04	5.46	-6.37E-04	6.37E-04
5.461	-4.00E-04	4.00E-04	5.461	-4.77E-04	4.77E-04	5.461	-5.39E-04	5.39E-04	5.461	-5.92E-04	5.92E-04	5.461	-6.37E-04	6.37E-04
5.462	-4.00E-04	4.00E-04	5.462	-4.77E-04	4.77E-04	5.462	-5.39E-04	5.39E-04	5.462	-5.92E-04	5.92E-04	5.462	-6.37E-04	6.37E-04
5.463	-4.00E-04	4.00E-04	5.463	-4.76E-04	4.76E-04	5.463	-5.39E-04	5.39E-04	5.463	-5.91E-04	5.91E-04	5.463	-6.36E-04	6.36E-04
5.464	-4.00E-04	4.00E-04	5.464	-4.76E-04	4.76E-04	5.464	-5.39E-04	5.39E-04	5.464	-5.91E-04	5.91E-04	5.464	-6.36E-04	6.36E-04
5.465	-3.99E-04	3.99E-04	5.465	-4.76E-04	4.76E-04	5.465	-5.39E-04	5.39E-04	5.465	-5.91E-04	5.91E-04	5.465	-6.36E-04	6.36E-04
5.466	-3.99E-04	3.99E-04	5.466	-4.76E-04	4.76E-04	5.466	-5.38E-04	5.38E-04	5.466	-5.91E-04	5.91E-04	5.466	-6.36E-04	6.36E-04
5.467	-3.99E-04	3.99E-04	5.467	-4.76E-04	4.76E-04	5.467	-5.38E-04	5.38E-04	5.467	-5.91E-04	5.91E-04	5.467	-6.35E-04	6.35E-04
5.468	-3.99E-04	3.99E-04	5.468	-4.75E-04	4.75E-04	5.468	-5.38E-04	5.38E-04	5.468	-5.90E-04	5.90E-04	5.468	-6.35E-04	6.35E-04
5.469	-3.99E-04	3.99E-04	5.469	-4.75E-04	4.75E-04	5.469	-5.38E-04	5.38E-04	5.469	-5.90E-04	5.90E-04	5.469	-6.35E-04	6.35E-04
5.47	-3.99E-04	3.99E-04	5.47	-4.75E-04	4.75E-04	5.47	-5.38E-04	5.38E-04	5.47	-5.90E-04	5.90E-04	5.47	-6.35E-04	6.35E-04
5.471	-3.99E-04	3.99E-04	5.471	-4.75E-04	4.75E-04	5.471	-5.37E-04	5.37E-04	5.471	-5.90E-04	5.90E-04	5.471	-6.35E-04	6.35E-04
5.472	-3.98E-04	3.98E-04	5.472	-4.75E-04	4.75E-04	5.472	-5.37E-04	5.37E-04	5.472	-5.90E-04	5.90E-04	5.472	-6.34E-04	6.34E-04
5.473	-3.98E-04	3.98E-04	5.473	-4.75E-04	4.75E-04	5.473	-5.37E-04	5.37E-04	5.473	-5.89E-04	5.89E-04	5.473	-6.34E-04	6.34E-04
5.474	-3.98E-04	3.98E-04	5.474	-4.74E-04	4.74E-04	5.474	-5.37E-04	5.37E-04	5.474	-5.89E-04	5.89E-04	5.474	-6.34E-04	6.34E-04
5.475	-3.98E-04	3.98E-04	5.475	-4.74E-04	4.74E-04	5.475	-5.37E-04	5.37E-04	5.475	-5.89E-04	5.89E-04	5.475	-6.34E-04	6.34E-04
5.476	-3.98E-04	3.98E-04	5.476	-4.74E-04	4.74E-04	5.476	-5.36E-04	5.36E-04	5.476	-5.89E-04	5.89E-04	5.476	-6.33E-04	6.33E-04
5.477	-3.98E-04	3.98E-04	5.477	-4.74E-04	4.74E-04	5.477	-5.36E-04	5.36E-04	5.477	-5.88E-04	5.88E-04	5.477	-6.33E-04	6.33E-04
5.478	-3.98E-04	3.98E-04	5.478	-4.74E-04	4.74E-04	5.478	-5.36E-04	5.36E-04	5.478	-5.88E-04	5.88E-04	5.478	-6.33E-04	6.33E-04
5.479	-3.97E-04	3.97E-04	5.479	-4.74E-04	4.74E-04	5.479	-5.36E-04	5.36E-04	5.479	-5.88E-04	5.88E-04	5.479	-6.33E-04	6.33E-04
5.48	-3.97E-04	3.97E-04	5.48	-4.73E-04	4.73E-04	5.48	-5.36E-04	5.36E-04	5.48	-5.88E-04	5.88E-04	5.48	-6.32E-04	6.32E-04
5.481	-3.97E-04	3.97E-04	5.481	-4.73E-04	4.73E-04	5.481	-5.35E-04	5.35E-04	5.481	-5.88E-04	5.88E-04	5.481	-6.32E-04	6.32E-04
5.482	-3.97E-04	3.97E-04	5.482	-4.73E-04	4.73E-04	5.482	-5.35E-04	5.35E-04	5.482	-5.87E-04	5.87E-04	5.482	-6.32E-04	6.32E-04
5.483	-3.97E-04	3.97E-04	5.483	-4.73E-04	4.73E-04	5.483	-5.35E-04	5.35E-04	5.483	-5.87E-04	5.87E-04	5.483	-6.32E-04	6.32E-04
5.484	-3.97E-04	3.97E-04	5.484	-4.73E-04	4.73E-04	5.484	-5.35E-04	5.35E-04	5.484	-5.87E-04	5.87E-04	5.484	-6.32E-04	6.32E-04
5.485	-3.97E-04	3.97E-04	5.485	-4.73E-04	4.73E-04	5.485	-5.35E-04	5.35E-04	5.485	-5.87E-04	5.87E-04	5.485	-6.31E-04	6.31E-04
5.486	-3.96E-04	3.96E-04	5.486	-4.72E-04	4.72E-04	5.486	-5.34E-04	5.34E-04	5.486	-5.87E-04	5.87E-04	5.486	-6.31E-04	6.31E-04
5.487	-3.96E-04	3.96E-04	5.487	-4.72E-04	4.72E-04	5.487	-5.34E-04	5.34E-04	5.487	-5.86E-04	5.86E-04	5.487	-6.31E-04	6.31E-04
5.488	-3.96E-04	3.96E-04	5.488	-4.72E-04	4.72E-04	5.488	-5.34E-04	5.34E-04	5.488	-5.86E-04	5.86E-04	5.488	-6.31E-04	6.31E-04
5.489	-3.96E-04	3.96E-04	5.489	-4.72E-04	4.72E-04	5.489	-5.34E-04	5.34E-04	5.489	-5.86E-04	5.86E-04	5.489	-6.30E-04	6.30E-04
5.49	-3.96E-04	3.96E-04	5.49	-4.72E-04	4.72E-04	5.49	-5.34E-04	5.34E-04	5.49	-5.86E-04	5.86E-04	5.49	-6.30E-04	6.30E-04
5.491	-3.96E-04	3.96E-04	5.491	-4.72E-04	4.72E-04	5.491	-5.33E-04	5.33E-04	5.491	-5.85E-04	5.85E-04	5.491	-6.30E-04	6.30E-04
5.492	-3.96E-04	3.96E-04	5.492	-4.71E-04	4.71E-04	5.492	-5.33E-04	5.33E-04	5.492	-5.85E-04	5.85E-04	5.492	-6.30E-04	6.30E-04
5.493	-3.95E-04	3.95E-04	5.493	-4.71E-04	4.71E-04	5.493	-5.33E-04	5.33E-04	5.493	-5.85E-04	5.85E-04	5.493	-6.29E-04	6.29E-04
5.494	-3.95E-04	3.95E-04	5.494	-4.71E-04	4.71E-04	5.494	-5.33E-04	5.33E-04	5.494	-5.85E-04	5.85E-04	5.494	-6.29E-04	6.29E-04
5.495	-3.95E-04	3.95E-04	5.495	-4.71E-04	4.71E-04	5.495	-5.33E-04	5.33E-04	5.495	-5.85E-04	5.85E-04	5.495	-6.29E-04	6.29E-04
5.496	-3.95E-04	3.95E-04	5.496	-4.71E-04	4.71E-04	5.496	-5.33E-04	5.33E-04	5.496	-5.84E-04	5.84E-04	5.496	-6.29E-04	6.29E-04
5.497	-3.95E-04	3.95E-04	5.497	-4.70E-04	4.70E-04	5.497	-5.32E-04	5.32E-04	5.497	-5.84E-04	5.84E-04	5.497	-6.29E-04	6.29E-04
5.498	-3.95E-04	3.95E-04	5.498	-4.70E-04	4.70E-04	5.498	-5.32E-04	5.32E-04	5.498	-5.84E-04	5.84E-04	5.498	-6.28E-04	6.28E-04
5.499	-3.95E-04	3.95E-04	5.499	-4.70E-04	4.70E-04	5.499	-5.32E-04	5.32E-04	5.499	-5.84E-04	5.84E-04	5.499	-6.28E-04	6.28E-04
5.5	-3.94E-04	3.94E-04	5.5	-4.70E-04	4.70E-04	5.5	-5.32E-04	5.32E-04	5.5	-5.84E-04	5.84E-04	5.5	-6.28E-04	6.28E-04
5.501	-3.94E-04	3.94E-04	5.501	-4.70E-04	4.70E-04	5.501	-5.32E-04	5.32E-04	5.501	-5.83E-04	5.83E-04	5.501	-6.28E-04	6.28E-04
5.502	-3.94E-04	3.94E-04	5.502	-4.70E-04	4.70E-04	5.502	-5.31E-04	5.31E-04	5.502	-5.83E-04	5.83E-04	5.502	-6.27E-04	6.27E-04
5.503	-3.94E-04	3.94E-04	5.503	-4.69E-04	4.69E-04	5.503	-5.31E-04	5.31E-04	5.503	-5.83E-04	5.83E-04	5.503	-6.27E-04	6.27E-04
5.504	-3.94E-04	3.94E-04	5.504	-4.69E-04	4.69E-04	5.504	-5.31E-04	5.31E-04	5.504	-5.83E-04	5.83E-04	5.504	-6.27E-04	6.27E-04
5.505	-3.94E-04	3.94E-04	5.505	-4.69E-04	4.69E-04	5.505	-5.31E-04	5.31E-04	5.505	-5.82E-04	5.82E-04	5.505	-6.27E-04	6.27E-04
5.506	-3.94E-04	3.94E-04	5.506	-4.69E-04	4.69E-04	5.506	-5.31E-04	5.31E-04	5.506	-5.82E-04	5.82E-04	5.506	-6.26E-04	6.26E-04
5.507	-3.93E-04	3.93E-04	5.507	-4.69E-04	4.69E-04	5.507	-5.30E-04	5.30E-04	5.507	-5.82E-04	5.82E-04	5.507	-6.26E-04	6.26E-04
5.508	-3.93E-04	3.93E-04	5.508	-4.69E-04	4.69E-04	5.508	-5.30E-04	5.30E-04	5.508	-5.82E-04	5.82E-04	5.508	-6.26E-04	6.26E-04
5.509	-3.93E-04	3.93E-04	5.509	-4.68E-04	4.68E-04	5.509	-5.30E-04	5.30E-04	5.509	-5.82E-04	5.82E-04	5.509	-6.26E-04	6.26E-04
5.51	-3.93E-04	3.93E-04	5.51	-4.68E-04	4.68E-04	5.51	-5.30E-04	5.30E-04	5.51	-5.81E-04	5.81E-04	5.51	-6.26E-04	6.26E-04
5.511	-3.93E-04	3.93E-04	5.511	-4.68E-04	4.68E-04	5.511	-5.30E-04	5.30E-04	5.511	-5.81E-04	5.81E-04	5.511	-6.25E-04	6.25E-04
5.512	-3.93E-04	3.93E-04	5.512	-4.68E-04	4.68E-04	5.512	-5.29E-04	5.29E-04	5.512	-5.81E-04	5.81E-04	5.512	-6.25E-04	6.25E-04
5.513	-3.93E-04	3.93E-04	5.513	-4.68E-04	4.68E-04	5.513	-5.29E-04	5.29E-04	5.513	-5.81E-04	5.81E-04	5.513	-6.25E-04	6.25E-04
5.514	-3.92E-04	3.92E-04	5.514	-4.68E-04	4.68E-04	5.514	-5.29E-04	5.29E-04	5.514	-5.81E-04	5.81E-04	5.514	-6.25E-04	6.25E-04
5.515	-3.92E-04	3.92E-04	5.515	-4.67E-04	4.67E-04	5.515	-5.29E-04	5.29E-04	5.515	-5.80E-04	5.80E-04	5.515	-6.24E-04	6.24E-04
5.516	-3.92E-04	3.92E-04	5.516	-4.67E-04	4.67E-04	5.516	-5.29E-04	5.29E-04	5.516	-5.80E-04	5.80E-04	5.516	-6.24E-04	6.24E-04
5.517	-3.92E-04	3.92E-04	5.517	-4.67E-04	4.67E-04	5.517	-5.28E-04	5.28E-04	5.517	-5.80E-04	5.80E-04	5.517	-6.24E-04	6.24E-04
5.518	-3.92E-04	3.92E-04	5.518	-4.67E-04	4.67E-04	5.518	-5.28E-04	5.28E-04	5.518	-5.80E-04	5.80E-04	5.518	-6.24E-04	6.24E-04
5.519	-3.92E-04	3.92E-04	5.519	-4.67E-04	4.67E-04	5.519	-5.28E-04	5.28E-04	5.519	-5.80E-04	5.80E-04	5.519	-6.24E-04	6.24E-04
5.52	-3.92E-04	3.92E-04	5.52	-4.67E-04	4.67E-04	5.52	-5.28E-04	5.28E-04	5.52	-5.79E-04	5.79E-04	5.52	-6.23E-04	6.23E-04
5.521	-3.91E-04	3.91E-04	5.521	-4.66E-04	4.66E-04	5.521	-5.28E-04	5.28E-04	5.521	-5.79E-04	5.79E-04	5.521	-6.23E-04	6.23E-04
5.522	-3.91E-04	3.91E-04	5.522	-4.66E-04	4.66E-04	5.522	-5.28E-04	5.28E-04	5.522	-5.79E-04	5.79E-04	5.522	-6.23E-04	6.23E-04
5.523	-3.91E-04	3.91E-04	5.523	-4.66E-04	4.66E-04	5.523	-5.27E-04	5.27E-04	5.523	-5.79E-04	5.79E-04	5.523	-6.23E-04	6.23E-04
5.524	-3.91E-04	3.91E-04	5.524	-4.66E-04	4.66E-04	5.524	-5.27E-04	5.27E-04	5.524	-5.78E-04	5.78E-04	5.524	-6.22E-04	6.22E-04
5.525	-3.91E-04	3.91E-04	5.525	-4.66E-04	4.66E-04	5.525	-5.27E-04	5.27E-04	5.525	-5.78E-04	5.78E-04	5.525	-6.22E-04	6.22E-04
5.526	-3.91E-04	3.91E-04	5.526	-4.66E-04	4.66E-04	5.526	-5.27E-04	5.27E-04	5.526	-5.78E-04	5.78E-04	5.526	-6.22E-04	6.22E-04
5.527	-3.91E-04	3.91E-04	5.527	-4.65E-04	4.65E-04	5.527	-5.27E-04	5.27E-04	5.527	-5.78E-04	5.78E-04	5.527	-6.22E-04	6.22E-04
5.528	-3.90E-04	3.90E-04	5.528	-4.65E-04	4.65E-04	5.528	-5.26E-04	5.26E-04	5.528	-5.78E-04	5.78E-04	5.528	-6.22E-04	6.22E-04
5.529	-3.90E-04	3.90E-04	5.529	-4.65E-04	4.65E-04	5.529	-5.26E-04	5.26E-04	5.529	-5.77E-04	5.77E-04	5.529	-6.21E-04	6.21E-04
5.53	-3.90E-04	3.90E-04	5.53	-4.65E-04	4.65E-04	5.53	-5.26E-04	5.26E-04	5.53	-5.77E-04	5.77E-04	5.53	-6.21E-04	6.21E-04
5.531	-3.90E-04	3.90E-04	5.531	-4.65E-04	4.65E-04	5.531	-5.26E-04	5.26E-04	5.531	-5.77E-04	5.77E-04	5.531	-6.21E-04	6.21E-04
5.532	-3.90E-04	3.90E-04	5.532	-4.65E-04	4.65E-04	5.532	-5.26E-04	5.26E-04	5.532	-5.77E-04	5.77E-04	5.532	-6.21E-04	6.21E-04
5.533	-3.90E-04	3.90E-04	5.533	-4.64E-04	4.64E-04	5.533	-5.25E-04	5.25E-04	5.533	-5.77E-04	5.77E-04	5.533	-6.20E-04	6.20E-04
5.534	-3.90E-04	3.90E-04	5.534	-4.64E-04	4.64E-04	5.534	-5.25E-04	5.25E-04	5.534	-5.76E-04	5.76E-04	5.534	-6.20E-04	6.20E-04
5.535	-3.89E-04	3.89E-04	5.535	-4.64E-04	4.64E-04	5.535	-5.25E-04	5.25E-04	5.535	-5.76E-04	5.76E-04	5.535	-6.20E-04	6.20E-04
5.536	-3.89E-04	3.89E-04	5.536	-4.64E-04	4.64E-04	5.536	-5.25E-04	5.25E-04	5.536	-5.76E-04	5.76E-04	5.536	-6.20E-04	6.20E-04
5.537	-3.89E-04	3.89E-04	5.537	-4.64E-04	4.64E-04	5.537	-5.25E-04	5.25E-04	5.537	-5.76E-04	5.76E-04	5.537	-6.20E-04	6.20E-04
5.538	-3.89E-04	3.89E-04	5.538	-4.64E-04	4.64E-04	5.538	-5.24E-04	5.24E-04	5.538	-5.76E-04	5.76E-04	5.538	-6.19E-04	6.19E-04
5.539	-3.89E-04	3.89E-04	5.539	-4.63E-04	4.63E-04	5.539	-5.24E-04	5.24E-04	5.539	-5.75E-04	5.75E-04	5.539	-6.19E-04	6.19E-04
5.54	-3.89E-04	3.89E-04	5.54	-4.63E-04	4.63E-04	5.54	-5.24E-04	5.24E-04	5.54	-5.75E-04	5.75E-04	5.54	-6.19E-04	6.19E-04
5.541	-3.89E-04	3.89E-04	5.541	-4.63E-04	4.63E-04	5.541	-5.24E-04	5.24E-04	5.541	-5.75E-04	5.75E-04	5.541	-6.19E-04	6.19E-04
5.542	-3.88E-04	3.88E-04	5.542	-4.63E-04	4.63E-04	5.542	-5.24E-04	5.24E-04	5.542	-5.75E-04	5.75E-04	5.542	-6.18E-04	6.18E-04
5.543	-3.88E-04	3.88E-04	5.543	-4.63E-04	4.63E-04	5.543	-5.24E-04	5.24E-04	5.543	-5.75E-04	5.75E-04	5.543	-6.18E-04	6.18E-04
5.544	-3.88E-04	3.88E-04	5.544	-4.63E-04	4.63E-04	5.544	-5.23E-04	5.23E-04	5.544	-5.74E-04	5.74E-04	5.544	-6.18E-04	6.18E-04
5.545	-3.88E-04	3.88E-04	5.545	-4.62E-04	4.62E-04	5.545	-5.23E-04	5.23E-04	5.545	-5.74E-04	5.74E-04	5.545	-6.18E-04	6.18E-04
5.546	-3.88E-04	3.88E-04	5.546	-4.62E-04	4.62E-04	5.546	-5.23E-04	5.23E-04	5.546	-5.74E-04	5.74E-04	5.546	-6.17E-04	6.17E-04
5.547	-3.88E-04	3.88E-04	5.547	-4.62E-04	4.62E-04	5.547	-5.23E-04	5.23E-04	5.547	-5.74E-04	5.74E-04	5.547	-6.17E-04	6.17E-04
5.548	-3.88E-04	3.88E-04	5.548	-4.62E-04	4.62E-04	5.548	-5.23E-04	5.23E-04	5.548	-5.73E-04	5.73E-04	5.548	-6.17E-04	6.17E-04
5.549	-3.87E-04	3.87E-04	5.549	-4.62E-04	4.62E-04	5.549	-5.22E-04	5.22E-04	5.549	-5.73E-04	5.73E-04	5.549	-6.17E-04	6.17E-04
5.55	-3.87E-04	3.87E-04	5.55	-4.62E-04	4.62E-04	5.55	-5.22E-04	5.22E-04	5.55	-5.73E-04	5.73E-04	5.55	-6.17E-04	6.17E-04
5.551	-3.87E-04	3.87E-04	5.551	-4.61E-04	4.61E-04	5.551	-5.22E-04	5.22E-04	5.551	-5.73E-04	5.73E-04	5.551	-6.16E-04	6.16E-04
5.552	-3.87E-04	3.87E-04	5.552	-4.61E-04	4.61E-04	5.552	-5.22E-04	5.22E-04	5.552	-5.73E-04	5.73E-04	5.552	-6.16E-04	6.16E-04
5.553	-3.87E-04	3.87E-04	5.553	-4.61E-04	4.61E-04	5.553	-5.22E-04	5.22E-04	5.553	-5.72E-04	5.72E-04	5.553	-6.16E-04	6.16E-04
5.554	-3.87E-04	3.87E-04	5.554	-4.61E-04	4.61E-04	5.554	-5.21E-04	5.21E-04	5.554	-5.72E-04	5.72E-04	5.554	-6.16E-04	6.16E-04
5.555	-3.87E-04	3.87E-04	5.555	-4.61E-04	4.61E-04	5.555	-5.21E-04	5.21E-04	5.555	-5.72E-04	5.72E-04	5.555	-6.15E-04	6.15E-04
5.556	-3.86E-04	3.86E-04	5.556	-4.61E-04	4.61E-04	5.556	-5.21E-04	5.21E-04	5.556	-5.72E-04	5.72E-04	5.556	-6.15E-04	6.15E-04
5.557	-3.86E-04	3.86E-04	5.557	-4.60E-04	4.60E-04	5.557	-5.21E-04	5.21E-04	5.557	-5.72E-04	5.72E-04	5.557	-6.15E-04	6.15E-04
5.558	-3.86E-04	3.86E-04	5.558	-4.60E-04	4.60E-04	5.558	-5.21E-04	5.21E-04	5.558	-5.71E-04	5.71E-04	5.558	-6.15E-04	6.15E-04
5.559	-3.86E-04	3.86E-04	5.559	-4.60E-04	4.60E-04	5.559	-5.21E-04	5.21E-04	5.559	-5.71E-04	5.71E-04	5.559	-6.15E-04	6.15E-04
5.56	-3.86E-04	3.86E-04	5.56	-4.60E-04	4.60E-04	5.56	-5.20E-04	5.20E-04	5.56	-5.71E-04	5.71E-04	5.56	-6.14E-04	6.14E-04
5.561	-3.86E-04	3.86E-04	5.561	-4.60E-04	4.60E-04	5.561	-5.20E-04	5.20E-04	5.561	-5.71E-04	5.71E-04	5.561	-6.14E-04	6.14E-04
5.562	-3.86E-04	3.86E-04	5.562	-4.60E-04	4.60E-04	5.562	-5.20E-04	5.20E-04	5.562	-5.71E-04	5.71E-04	5.562	-6.14E-04	6.14E-04
5.563	-3.85E-04	3.85E-04	5.563	-4.59E-04	4.59E-04	5.563	-5.20E-04	5.20E-04	5.563	-5.70E-04	5.70E-04	5.563	-6.14E-04	6.14E-04
5.564	-3.85E-04	3.85E-04	5.564	-4.59E-04	4.59E-04	5.564	-5.20E-04	5.20E-04	5.564	-5.70E-04	5.70E-04	5.564	-6.14E-04	6.14E-04
5.565	-3.85E-04	3.85E-04	5.565	-4.59E-04	4.59E-04	5.565	-5.19E-04	5.19E-04	5.565	-5.70E-04	5.70E-04	5.565	-6.13E-04	6.13E-04
5.566	-3.85E-04	3.85E-04	5.566	-4.59E-04	4.59E-04	5.566	-5.19E-04	5.19E-04	5.566	-5.70E-04	5.70E-04	5.566	-6.13E-04	6.13E-04
5.567	-3.85E-04	3.85E-04	5.567	-4.59E-04	4.59E-04	5.567	-5.19E-04	5.19E-04	5.567	-5.70E-04	5.70E-04	5.567	-6.13E-04	6.13E-04
5.568	-3.85E-04	3.85E-04	5.568	-4.59E-04	4.59E-04	5.568	-5.19E-04	5.19E-04	5.568	-5.69E-04	5.69E-04	5.568	-6.13E-04	6.13E-04
5.569	-3.85E-04	3.85E-04	5.569	-4.58E-04	4.58E-04	5.569	-5.19E-04	5.19E-04	5.569	-5.69E-04	5.69E-04	5.569	-6.12E-04	6.12E-04
5.57	-3.85E-04	3.85E-04	5.57	-4.58E-04	4.58E-04	5.57	-5.18E-04	5.18E-04	5.57	-5.69E-04	5.69E-04	5.57	-6.12E-04	6.12E-04
5.571	-3.84E-04	3.84E-04	5.571	-4.58E-04	4.58E-04	5.571	-5.18E-04	5.18E-04	5.571	-5.69E-04	5.69E-04	5.571	-6.12E-04	6.12E-04
5.572	-3.84E-04	3.84E-04	5.572	-4.58E-04	4.58E-04	5.572	-5.18E-04	5.18E-04	5.572	-5.69E-04	5.69E-04	5.572	-6.12E-04	6.12E-04
5.573	-3.84E-04	3.84E-04	5.573	-4.58E-04	4.58E-04	5.573	-5.18E-04	5.18E-04	5.573	-5.68E-04	5.68E-04	5.573	-6.12E-04	6.12E-04
5.574	-3.84E-04	3.84E-04	5.574	-4.58E-04	4.58E-04	5.574	-5.18E-04	5.18E-04	5.574	-5.68E-04	5.68E-04	5.574	-6.11E-04	6.11E-04
5.575	-3.84E-04	3.84E-04	5.575	-4.57E-04	4.57E-04	5.575	-5.18E-04	5.18E-04	5.575	-5.68E-04	5.68E-04	5.575	-6.11E-04	6.11E-04
5.576	-3.84E-04	3.84E-04	5.576	-4.57E-04	4.57E-04	5.576	-5.17E-04	5.17E-04	5.576	-5.68E-04	5.68E-04	5.576	-6.11E-04	6.11E-04
5.577	-3.84E-04	3.84E-04	5.577	-4.57E-04	4.57E-04	5.577	-5.17E-04	5.17E-04	5.577	-5.68E-04	5.68E-04	5.577	-6.11E-04	6.11E-04
5.578	-3.83E-04	3.83E-04	5.578	-4.57E-04	4.57E-04	5.578	-5.17E-04	5.17E-04	5.578	-5.67E-04	5.67E-04	5.578	-6.10E-04	6.10E-04
5.579	-3.83E-04	3.83E-04	5.579	-4.57E-04	4.57E-04	5.579	-5.17E-04	5.17E-04	5.579	-5.67E-04	5.67E-04	5.579	-6.10E-04	6.10E-04
5.58	-3.83E-04	3.83E-04	5.58	-4.57E-04	4.57E-04	5.58	-5.17E-04	5.17E-04	5.58	-5.67E-04	5.67E-04	5.58	-6.10E-04	6.10E-04
5.581	-3.83E-04	3.83E-04	5.581	-4.56E-04	4.56E-04	5.581	-5.16E-04	5.16E-04	5.581	-5.67E-04	5.67E-04	5.581	-6.10E-04	6.10E-04
5.582	-3.83E-04	3.83E-04	5.582	-4.56E-04	4.56E-04	5.582	-5.16E-04	5.16E-04	5.582	-5.67E-04	5.67E-04	5.582	-6.10E-04	6.10E-04
5.583	-3.83E-04	3.83E-04	5.583	-4.56E-04	4.56E-04	5.583	-5.16E-04	5.16E-04	5.583	-5.66E-04	5.66E-04	5.583	-6.09E-04	6.09E-04
5.584	-3.83E-04	3.83E-04	5.584	-4.56E-04	4.56E-04	5.584	-5.16E-04	5.16E-04	5.584	-5.66E-04	5.66E-04	5.584	-6.09E-04	6.09E-04
5.585	-3.82E-04	3.82E-04	5.585	-4.56E-04	4.56E-04	5.585	-5.16E-04	5.16E-04	5.585	-5.66E-04	5.66E-04	5.585	-6.09E-04	6.09E-04
5.586	-3.82E-04	3.82E-04	5.586	-4.56E-04	4.56E-04	5.586	-5.15E-04	5.15E-04	5.586	-5.66E-04	5.66E-04	5.586	-6.09E-04	6.09E-04
5.587	-3.82E-04	3.82E-04	5.587	-4.55E-04	4.55E-04	5.587	-5.15E-04	5.15E-04	5.587	-5.65E-04	5.65E-04	5.587	-6.08E-04	6.08E-04

5.588	-3.82E-04	3.82E-04	5.588	-4.55E-04	4.55E-04	5.588	-5.15E-04	5.15E-04	5.588	-5.65E-04	5.65E-04	5.588	-6.08E-04	6.08E-04
5.589	-3.82E-04	3.82E-04	5.589	-4.55E-04	4.55E-04	5.589	-5.15E-04	5.15E-04	5.589	-5.65E-04	5.65E-04	5.589	-6.08E-04	6.08E-04
5.59	-3.82E-04	3.82E-04	5.59	-4.55E-04	4.55E-04	5.59	-5.15E-04	5.15E-04	5.59	-5.65E-04	5.65E-04	5.59	-6.08E-04	6.08E-04
5.591	-3.82E-04	3.82E-04	5.591	-4.55E-04	4.55E-04	5.591	-5.15E-04	5.15E-04	5.591	-5.65E-04	5.65E-04	5.591	-6.08E-04	6.08E-04
5.592	-3.82E-04	3.82E-04	5.592	-4.55E-04	4.55E-04	5.592	-5.14E-04	5.14E-04	5.592	-5.64E-04	5.64E-04	5.592	-6.07E-04	6.07E-04
5.593	-3.81E-04	3.81E-04	5.593	-4.54E-04	4.54E-04	5.593	-5.14E-04	5.14E-04	5.593	-5.64E-04	5.64E-04	5.593	-6.07E-04	6.07E-04
5.594	-3.81E-04	3.81E-04	5.594	-4.54E-04	4.54E-04	5.594	-5.14E-04	5.14E-04	5.594	-5.64E-04	5.64E-04	5.594	-6.07E-04	6.07E-04
5.595	-3.81E-04	3.81E-04	5.595	-4.54E-04	4.54E-04	5.595	-5.14E-04	5.14E-04	5.595	-5.64E-04	5.64E-04	5.595	-6.07E-04	6.07E-04
5.596	-3.81E-04	3.81E-04	5.596	-4.54E-04	4.54E-04	5.596	-5.14E-04	5.14E-04	5.596	-5.64E-04	5.64E-04	5.596	-6.07E-04	6.07E-04
5.597	-3.81E-04	3.81E-04	5.597	-4.54E-04	4.54E-04	5.597	-5.13E-04	5.13E-04	5.597	-5.63E-04	5.63E-04	5.597	-6.06E-04	6.06E-04
5.598	-3.81E-04	3.81E-04	5.598	-4.54E-04	4.54E-04	5.598	-5.13E-04	5.13E-04	5.598	-5.63E-04	5.63E-04	5.598	-6.06E-04	6.06E-04
5.599	-3.81E-04	3.81E-04	5.599	-4.53E-04	4.53E-04	5.599	-5.13E-04	5.13E-04	5.599	-5.63E-04	5.63E-04	5.599	-6.06E-04	6.06E-04
5.6	-3.80E-04	3.80E-04	5.6	-4.53E-04	4.53E-04	5.6	-5.13E-04	5.13E-04	5.6	-5.63E-04	5.63E-04	5.6	-6.06E-04	6.06E-04
5.601	-3.80E-04	3.80E-04	5.601	-4.53E-04	4.53E-04	5.601	-5.13E-04	5.13E-04	5.601	-5.63E-04	5.63E-04	5.601	-6.05E-04	6.05E-04
5.602	-3.80E-04	3.80E-04	5.602	-4.53E-04	4.53E-04	5.602	-5.13E-04	5.13E-04	5.602	-5.62E-04	5.62E-04	5.602	-6.05E-04	6.05E-04
5.603	-3.80E-04	3.80E-04	5.603	-4.53E-04	4.53E-04	5.603	-5.12E-04	5.12E-04	5.603	-5.62E-04	5.62E-04	5.603	-6.05E-04	6.05E-04
5.604	-3.80E-04	3.80E-04	5.604	-4.53E-04	4.53E-04	5.604	-5.12E-04	5.12E-04	5.604	-5.62E-04	5.62E-04	5.604	-6.05E-04	6.05E-04
5.605	-3.80E-04	3.80E-04	5.605	-4.53E-04	4.53E-04	5.605	-5.12E-04	5.12E-04	5.605	-5.62E-04	5.62E-04	5.605	-6.05E-04	6.05E-04
5.606	-3.80E-04	3.80E-04	5.606	-4.52E-04	4.52E-04	5.606	-5.12E-04	5.12E-04	5.606	-5.62E-04	5.62E-04	5.606	-6.04E-04	6.04E-04
5.607	-3.79E-04	3.79E-04	5.607	-4.52E-04	4.52E-04	5.607	-5.12E-04	5.12E-04	5.607	-5.61E-04	5.61E-04	5.607	-6.04E-04	6.04E-04
5.608	-3.79E-04	3.79E-04	5.608	-4.52E-04	4.52E-04	5.608	-5.11E-04	5.11E-04	5.608	-5.61E-04	5.61E-04	5.608	-6.04E-04	6.04E-04
5.609	-3.79E-04	3.79E-04	5.609	-4.52E-04	4.52E-04	5.609	-5.11E-04	5.11E-04	5.609	-5.61E-04	5.61E-04	5.609	-6.04E-04	6.04E-04
5.61	-3.79E-04	3.79E-04	5.61	-4.52E-04	4.52E-04	5.61	-5.11E-04	5.11E-04	5.61	-5.61E-04	5.61E-04	5.61	-6.03E-04	6.03E-04
5.611	-3.79E-04	3.79E-04	5.611	-4.52E-04	4.52E-04	5.611	-5.11E-04	5.11E-04	5.611	-5.61E-04	5.61E-04	5.611	-6.03E-04	6.03E-04
5.612	-3.79E-04	3.79E-04	5.612	-4.51E-04	4.51E-04	5.612	-5.11E-04	5.11E-04	5.612	-5.60E-04	5.60E-04	5.612	-6.03E-04	6.03E-04
5.613	-3.79E-04	3.79E-04	5.613	-4.51E-04	4.51E-04	5.613	-5.11E-04	5.11E-04	5.613	-5.60E-04	5.60E-04	5.613	-6.03E-04	6.03E-04
5.614	-3.79E-04	3.79E-04	5.614	-4.51E-04	4.51E-04	5.614	-5.10E-04	5.10E-04	5.614	-5.60E-04	5.60E-04	5.614	-6.03E-04	6.03E-04
5.615	-3.78E-04	3.78E-04	5.615	-4.51E-04	4.51E-04	5.615	-5.10E-04	5.10E-04	5.615	-5.60E-04	5.60E-04	5.615	-6.02E-04	6.02E-04
5.616	-3.78E-04	3.78E-04	5.616	-4.51E-04	4.51E-04	5.616	-5.10E-04	5.10E-04	5.616	-5.60E-04	5.60E-04	5.616	-6.02E-04	6.02E-04
5.617	-3.78E-04	3.78E-04	5.617	-4.51E-04	4.51E-04	5.617	-5.10E-04	5.10E-04	5.617	-5.59E-04	5.59E-04	5.617	-6.02E-04	6.02E-04
5.618	-3.78E-04	3.78E-04	5.618	-4.50E-04	4.50E-04	5.618	-5.10E-04	5.10E-04	5.618	-5.59E-04	5.59E-04	5.618	-6.02E-04	6.02E-04
5.619	-3.78E-04	3.78E-04	5.619	-4.50E-04	4.50E-04	5.619	-5.09E-04	5.09E-04	5.619	-5.59E-04	5.59E-04	5.619	-6.02E-04	6.02E-04
5.62	-3.78E-04	3.78E-04	5.62	-4.50E-04	4.50E-04	5.62	-5.09E-04	5.09E-04	5.62	-5.59E-04	5.59E-04	5.62	-6.01E-04	6.01E-04
5.621	-3.78E-04	3.78E-04	5.621	-4.50E-04	4.50E-04	5.621	-5.09E-04	5.09E-04	5.621	-5.59E-04	5.59E-04	5.621	-6.01E-04	6.01E-04
5.622	-3.77E-04	3.77E-04	5.622	-4.50E-04	4.50E-04	5.622	-5.09E-04	5.09E-04	5.622	-5.58E-04	5.58E-04	5.622	-6.01E-04	6.01E-04
5.623	-3.77E-04	3.77E-04	5.623	-4.50E-04	4.50E-04	5.623	-5.09E-04	5.09E-04	5.623	-5.58E-04	5.58E-04	5.623	-6.01E-04	6.01E-04
5.624	-3.77E-04	3.77E-04	5.624	-4.49E-04	4.49E-04	5.624	-5.09E-04	5.09E-04	5.624	-5.58E-04	5.58E-04	5.624	-6.00E-04	6.00E-04
5.625	-3.77E-04	3.77E-04	5.625	-4.49E-04	4.49E-04	5.625	-5.08E-04	5.08E-04	5.625	-5.58E-04	5.58E-04	5.625	-6.00E-04	6.00E-04
5.626	-3.77E-04	3.77E-04	5.626	-4.49E-04	4.49E-04	5.626	-5.08E-04	5.08E-04	5.626	-5.58E-04	5.58E-04	5.626	-6.00E-04	6.00E-04
5.627	-3.77E-04	3.77E-04	5.627	-4.49E-04	4.49E-04	5.627	-5.08E-04	5.08E-04	5.627	-5.57E-04	5.57E-04	5.627	-6.00E-04	6.00E-04
5.628	-3.77E-04	3.77E-04	5.628	-4.49E-04	4.49E-04	5.628	-5.08E-04	5.08E-04	5.628	-5.57E-04	5.57E-04	5.628	-6.00E-04	6.00E-04
5.629	-3.77E-04	3.77E-04	5.629	-4.49E-04	4.49E-04	5.629	-5.08E-04	5.08E-04	5.629	-5.57E-04	5.57E-04	5.629	-5.99E-04	5.99E-04
5.63	-3.76E-04	3.76E-04	5.63	-4.49E-04	4.49E-04	5.63	-5.07E-04	5.07E-04	5.63	-5.57E-04	5.57E-04	5.63	-5.99E-04	5.99E-04
5.631	-3.76E-04	3.76E-04	5.631	-4.48E-04	4.48E-04	5.631	-5.07E-04	5.07E-04	5.631	-5.57E-04	5.57E-04	5.631	-5.99E-04	5.99E-04
5.632	-3.76E-04	3.76E-04	5.632	-4.48E-04	4.48E-04	5.632	-5.07E-04	5.07E-04	5.632	-5.56E-04	5.56E-04	5.632	-5.99E-04	5.99E-04
5.633	-3.76E-04	3.76E-04	5.633	-4.48E-04	4.48E-04	5.633	-5.07E-04	5.07E-04	5.633	-5.56E-04	5.56E-04	5.633	-5.99E-04	5.99E-04
5.634	-3.76E-04	3.76E-04	5.634	-4.48E-04	4.48E-04	5.634	-5.07E-04	5.07E-04	5.634	-5.56E-04	5.56E-04	5.634	-5.98E-04	5.98E-04
5.635	-3.76E-04	3.76E-04	5.635	-4.48E-04	4.48E-04	5.635	-5.07E-04	5.07E-04	5.635	-5.56E-04	5.56E-04	5.635	-5.98E-04	5.98E-04
5.636	-3.76E-04	3.76E-04	5.636	-4.48E-04	4.48E-04	5.636	-5.06E-04	5.06E-04	5.636	-5.56E-04	5.56E-04	5.636	-5.98E-04	5.98E-04
5.637	-3.75E-04	3.75E-04	5.637	-4.47E-04	4.47E-04	5.637	-5.06E-04	5.06E-04	5.637	-5.56E-04	5.56E-04	5.637	-5.98E-04	5.98E-04
5.638	-3.75E-04	3.75E-04	5.638	-4.47E-04	4.47E-04	5.638	-5.06E-04	5.06E-04	5.638	-5.55E-04	5.55E-04	5.638	-5.98E-04	5.98E-04
5.639	-3.75E-04	3.75E-04	5.639	-4.47E-04	4.47E-04	5.639	-5.06E-04	5.06E-04	5.639	-5.55E-04	5.55E-04	5.639	-5.97E-04	5.97E-04
5.64	-3.75E-04	3.75E-04	5.64	-4.47E-04	4.47E-04	5.64	-5.06E-04	5.06E-04	5.64	-5.55E-04	5.55E-04	5.64	-5.97E-04	5.97E-04
5.641	-3.75E-04	3.75E-04	5.641	-4.47E-04	4.47E-04	5.641	-5.05E-04	5.05E-04	5.641	-5.55E-04	5.55E-04	5.641	-5.97E-04	5.97E-04
5.642	-3.75E-04	3.75E-04	5.642	-4.47E-04	4.47E-04	5.642	-5.05E-04	5.05E-04	5.642	-5.55E-04	5.55E-04	5.642	-5.97E-04	5.97E-04
5.643	-3.75E-04	3.75E-04	5.643	-4.46E-04	4.46E-04	5.643	-5.05E-04	5.05E-04	5.643	-5.54E-04	5.54E-04	5.643	-5.96E-04	5.96E-04
5.644	-3.75E-04	3.75E-04	5.644	-4.46E-04	4.46E-04	5.644	-5.05E-04	5.05E-04	5.644	-5.54E-04	5.54E-04	5.644	-5.96E-04	5.96E-04
5.645	-3.74E-04	3.74E-04	5.645	-4.46E-04	4.46E-04	5.645	-5.05E-04	5.05E-04	5.645	-5.54E-04	5.54E-04	5.645	-5.96E-04	5.96E-04
5.646	-3.74E-04	3.74E-04	5.646	-4.46E-04	4.46E-04	5.646	-5.05E-04	5.05E-04	5.646	-5.54E-04	5.54E-04	5.646	-5.96E-04	5.96E-04
5.647	-3.74E-04	3.74E-04	5.647	-4.46E-04	4.46E-04	5.647	-5.04E-04	5.04E-04	5.647	-5.54E-04	5.54E-04	5.647	-5.96E-04	5.96E-04
5.648	-3.74E-04	3.74E-04	5.648	-4.46E-04	4.46E-04	5.648	-5.04E-04	5.04E-04	5.648	-5.53E-04	5.53E-04	5.648	-5.95E-04	5.95E-04
5.649	-3.74E-04	3.74E-04	5.649	-4.46E-04	4.46E-04	5.649	-5.04E-04	5.04E-04	5.649	-5.53E-04	5.53E-04	5.649	-5.95E-04	5.95E-04
5.65	-3.74E-04	3.74E-04	5.65	-4.45E-04	4.45E-04	5.65	-5.04E-04	5.04E-04	5.65	-5.53E-04	5.53E-04	5.65	-5.95E-04	5.95E-04
5.651	-3.74E-04	3.74E-04	5.651	-4.45E-04	4.45E-04	5.651	-5.04E-04	5.04E-04	5.651	-5.53E-04	5.53E-04	5.651	-5.95E-04	5.95E-04
5.652	-3.73E-04	3.73E-04	5.652	-4.45E-04	4.45E-04	5.652	-5.04E-04	5.04E-04	5.652	-5.53E-04	5.53E-04	5.652	-5.95E-04	5.95E-04
5.653	-3.73E-04	3.73E-04	5.653	-4.45E-04	4.45E-04	5.653	-5.03E-04	5.03E-04	5.653	-5.52E-04	5.52E-04	5.653	-5.94E-04	5.94E-04
5.654	-3.73E-04	3.73E-04	5.654	-4.45E-04	4.45E-04	5.654	-5.03E-04	5.03E-04	5.654	-5.52E-04	5.52E-04	5.654	-5.94E-04	5.94E-04
5.655	-3.73E-04	3.73E-04	5.655	-4.45E-04	4.45E-04	5.655	-5.03E-04	5.03E-04	5.655	-5.52E-04	5.52E-04	5.655	-5.94E-04	5.94E-04
5.656	-3.73E-04	3.73E-04	5.656	-4.44E-04	4.44E-04	5.656	-5.03E-04	5.03E-04	5.656	-5.52E-04	5.52E-04	5.656	-5.94E-04	5.94E-04
5.657	-3.73E-04	3.73E-04	5.657	-4.44E-04	4.44E-04	5.657	-5.03E-04	5.03E-04	5.657	-5.52E-04	5.52E-04	5.657	-5.93E-04	5.93E-04
5.658	-3.73E-04	3.73E-04	5.658	-4.44E-04	4.44E-04	5.658	-5.02E-04	5.02E-04	5.658	-5.51E-04	5.51E-04	5.658	-5.93E-04	5.93E-04
5.659	-3.73E-04	3.73E-04	5.659	-4.44E-04	4.44E-04	5.659	-5.02E-04	5.02E-04	5.659	-5.51E-04	5.51E-04	5.659	-5.93E-04	5.93E-04
5.66	-3.72E-04	3.72E-04	5.66	-4.44E-04	4.44E-04	5.66	-5.02E-04	5.02E-04	5.66	-5.51E-04	5.51E-04	5.66	-5.93E-04	5.93E-04
5.661	-3.72E-04	3.72E-04	5.661	-4.44E-04	4.44E-04	5.661	-5.02E-04	5.02E-04	5.661	-5.51E-04	5.51E-04	5.661	-5.93E-04	5.93E-04
5.662	-3.72E-04	3.72E-04	5.662	-4.43E-04	4.43E-04	5.662	-5.02E-04	5.02E-04	5.662	-5.51E-04	5.51E-04	5.662	-5.92E-04	5.92E-04
5.663	-3.72E-04	3.72E-04	5.663	-4.43E-04	4.43E-04	5.663	-5.02E-04	5.02E-04	5.663	-5.50E-04	5.50E-04	5.663	-5.92E-04	5.92E-04
5.664	-3.72E-04	3.72E-04	5.664	-4.43E-04	4.43E-04	5.664	-5.01E-04	5.01E-04	5.664	-5.50E-04	5.50E-04	5.664	-5.92E-04	5.92E-04
5.665	-3.72E-04	3.72E-04	5.665	-4.43E-04	4.43E-04	5.665	-5.01E-04	5.01E-04	5.665	-5.50E-04	5.50E-04	5.665	-5.92E-04	5.92E-04
5.666	-3.72E-04	3.72E-04	5.666	-4.43E-04	4.43E-04	5.666	-5.01E-04	5.01E-04	5.666	-5.50E-04	5.50E-04	5.666	-5.92E-04	5.92E-04
5.667	-3.71E-04	3.71E-04	5.667	-4.43E-04	4.43E-04	5.667	-5.01E-04	5.01E-04	5.667	-5.50E-04	5.50E-04	5.667	-5.91E-04	5.91E-04
5.668	-3.71E-04	3.71E-04	5.668	-4.43E-04	4.43E-04	5.668	-5.01E-04	5.01E-04	5.668	-5.49E-04	5.49E-04	5.668	-5.91E-04	5.91E-04
5.669	-3.71E-04	3.71E-04	5.669	-4.42E-04	4.42E-04	5.669	-5.01E-04	5.01E-04	5.669	-5.49E-04	5.49E-04	5.669	-5.91E-04	5.91E-04
5.67	-3.71E-04	3.71E-04	5.67	-4.42E-04	4.42E-04	5.67	-5.00E-04	5.00E-04	5.67	-5.49E-04	5.49E-04	5.67	-5.91E-04	5.91E-04
5.671	-3.71E-04	3.71E-04	5.671	-4.42E-04	4.42E-04	5.671	-5.00E-04	5.00E-04	5.671	-5.49E-04	5.49E-04	5.671	-5.91E-04	5.91E-04
5.672	-3.71E-04	3.71E-04	5.672	-4.42E-04	4.42E-04	5.672	-5.00E-04	5.00E-04	5.672	-5.49E-04	5.49E-04	5.672	-5.90E-04	5.90E-04
5.673	-3.71E-04	3.71E-04	5.673	-4.42E-04	4.42E-04	5.673	-5.00E-04	5.00E-04	5.673	-5.48E-04	5.48E-04	5.673	-5.90E-04	5.90E-04
5.674	-3.71E-04	3.71E-04	5.674	-4.42E-04	4.42E-04	5.674	-5.00E-04	5.00E-04	5.674	-5.48E-04	5.48E-04	5.674	-5.90E-04	5.90E-04
5.675	-3.70E-04	3.70E-04	5.675	-4.41E-04	4.41E-04	5.675	-4.99E-04	4.99E-04	5.675	-5.48E-04	5.48E-04	5.675	-5.90E-04	5.90E-04
5.676	-3.70E-04	3.70E-04	5.676	-4.41E-04	4.41E-04	5.676	-4.99E-04	4.99E-04	5.676	-5.48E-04	5.48E-04	5.676	-5.90E-04	5.90E-04
5.677	-3.70E-04	3.70E-04	5.677	-4.41E-04	4.41E-04	5.677	-4.99E-04	4.99E-04	5.677	-5.48E-04	5.48E-04	5.677	-5.89E-04	5.89E-04
5.678	-3.70E-04	3.70E-04	5.678	-4.41E-04	4.41E-04	5.678	-4.99E-04	4.99E-04	5.678	-5.48E-04	5.48E-04	5.678	-5.89E-04	5.89E-04
5.679	-3.70E-04	3.70E-04	5.679	-4.41E-04	4.41E-04	5.679	-4.99E-04	4.99E-04	5.679	-5.47E-04	5.47E-04	5.679	-5.89E-04	5.89E-04
5.68	-3.70E-04	3.70E-04	5.68	-4.41E-04	4.41E-04	5.68	-4.99E-04	4.99E-04	5.68	-5.47E-04	5.47E-04	5.68	-5.89E-04	5.89E-04
5.681	-3.70E-04	3.70E-04	5.681	-4.40E-04	4.40E-04	5.681	-4.98E-04	4.98E-04	5.681	-5.47E-04	5.47E-04	5.681	-5.88E-04	5.88E-04
5.682	-3.70E-04	3.70E-04	5.682	-4.40E-04	4.40E-04	5.682	-4.98E-04	4.98E-04	5.682	-5.47E-04	5.47E-04	5.682	-5.88E-04	5.88E-04
5.683	-3.69E-04	3.69E-04	5.683	-4.40E-04	4.40E-04	5.683	-4.98E-04	4.98E-04	5.683	-5.47E-04	5.47E-04	5.683	-5.88E-04	5.88E-04
5.684	-3.69E-04	3.69E-04	5.684	-4.40E-04	4.40E-04	5.684	-4.98E-04	4.98E-04	5.684	-5.46E-04	5.46E-04	5.684	-5.88E-04	5.88E-04
5.685	-3.69E-04	3.69E-04	5.685	-4.40E-04	4.40E-04	5.685	-4.98E-04	4.98E-04	5.685	-5.46E-04	5.46E-04	5.685	-5.88E-04	5.88E-04
5.686	-3.69E-04	3.69E-04	5.686	-4.40E-04	4.40E-04	5.686	-4.98E-04	4.98E-04	5.686	-5.46E-04	5.46E-04	5.686	-5.87E-04	5.87E-04
5.687	-3.69E-04	3.69E-04	5.687	-4.40E-04	4.40E-04	5.687	-4.97E-04	4.97E-04	5.687	-5.46E-04	5.46E-04	5.687	-5.87E-04	5.87E-04
5.688	-3.69E-04	3.69E-04	5.688	-4.39E-04	4.39E-04	5.688	-4.97E-04	4.97E-04	5.688	-5.46E-04	5.46E-04	5.688	-5.87E-04	5.87E-04
5.689	-3.69E-04	3.69E-04	5.689	-4.39E-04	4.39E-04	5.689	-4.97E-04	4.97E-04	5.689	-5.45E-04	5.45E-04	5.689	-5.87E-04	5.87E-04
5.69	-3.68E-04	3.68E-04	5.69	-4.39E-04	4.39E-04	5.69	-4.97E-04	4.97E-04	5.69	-5.45E-04	5.45E-04	5.69	-5.87E-04	5.87E-04
5.691	-3.68E-04	3.68E-04	5.691	-4.39E-04	4.39E-04	5.691	-4.97E-04	4.97E-04	5.691	-5.45E-04	5.45E-04	5.691	-5.86E-04	5.86E-04
5.692	-3.68E-04	3.68E-04	5.692	-4.39E-04	4.39E-04	5.692	-4.96E-04	4.96E-04	5.692	-5.45E-04	5.45E-04	5.692	-5.86E-04	5.86E-04
5.693	-3.68E-04	3.68E-04	5.693	-4.39E-04	4.39E-04	5.693	-4.96E-04	4.96E-04	5.693	-5.45E-04	5.45E-04	5.693	-5.86E-04	5.86E-04
5.694	-3.68E-04	3.68E-04	5.694	-4.38E-04	4.38E-04	5.694	-4.96E-04	4.96E-04	5.694	-5.44E-04	5.44E-04	5.694	-5.86E-04	5.86E-04
5.695	-3.68E-04	3.68E-04	5.695	-4.38E-04	4.38E-04	5.695	-4.96E-04	4.96E-04	5.695	-5.44E-04	5.44E-04	5.695	-5.86E-04	5.86E-04
5.696	-3.68E-04	3.68E-04	5.696	-4.38E-04	4.38E-04	5.696	-4.96E-04	4.96E-04	5.696	-5.44E-04	5.44E-04	5.696	-5.85E-04	5.85E-04
5.697	-3.68E-04	3.68E-04	5.697	-4.38E-04	4.38E-04	5.697	-4.96E-04	4.96E-04	5.697	-5.44E-04	5.44E-04	5.697	-5.85E-04	5.85E-04
5.698	-3.67E-04	3.67E-04	5.698	-4.38E-04	4.38E-04	5.698	-4.95E-04	4.95E-04	5.698	-5.44E-04	5.44E-04	5.698	-5.85E-04	5.85E-04
5.699	-3.67E-04	3.67E-04	5.699	-4.38E-04	4.38E-04	5.699	-4.95E-04	4.95E-04	5.699	-5.43E-04	5.43E-04	5.699	-5.85E-04	5.85E-04
5.7	-3.67E-04	3.67E-04	5.7	-4.38E-04	4.38E-04	5.7	-4.95E-04	4.95E-04	5.7	-5.43E-04	5.43E-04	5.7	-5.85E-04	5.85E-04
5.701	-3.67E-04	3.67E-04	5.701	-4.37E-04	4.37E-04	5.701	-4.95E-04	4.95E-04	5.701	-5.43E-04	5.43E-04	5.701	-5.84E-04	5.84E-04
5.702	-3.67E-04	3.67E-04	5.702	-4.37E-04	4.37E-04	5.702	-4.95E-04	4.95E-04	5.702	-5.43E-04	5.43E-04	5.702	-5.84E-04	5.84E-04
5.703	-3.67E-04	3.67E-04	5.703	-4.37E-04	4.37E-04	5.703	-4.95E-04	4.95E-04	5.703	-5.43E-04	5.43E-04	5.703	-5.84E-04	5.84E-04
5.704	-3.67E-04	3.67E-04	5.704	-4.37E-04	4.37E-04	5.704	-4.94E-04	4.94E-04	5.704	-5.43E-04	5.43E-04	5.704	-5.84E-04	5.84E-04
5.705	-3.67E-04	3.67E-04	5.705	-4.37E-04	4.37E-04	5.705	-4.94E-04	4.94E-04	5.705	-5.42E-04	5.42E-04	5.705	-5.84E-04	5.84E-04
5.706	-3.66E-04	3.66E-04	5.706	-4.37E-04	4.37E-04	5.706	-4.94E-04	4.94E-04	5.706	-5.42E-04	5.42E-04	5.706	-5.83E-04	5.83E-04
5.707	-3.66E-04	3.66E-04	5.707	-4.36E-04	4.36E-04	5.707	-4.94E-04	4.94E-04	5.707	-5.42E-04	5.42E-04	5.707	-5.83E-04	5.83E-04
5.708	-3.66E-04	3.66E-04	5.708	-4.36E-04	4.36E-04	5.708	-4.94E-04	4.94E-04	5.708	-5.42E-04	5.42E-04	5.708	-5.83E-04	5.83E-04
5.709	-3.66E-04	3.66E-04	5.709	-4.36E-04	4.36E-04	5.709	-4.94E-04	4.94E-04	5.709	-5.42E-04	5.42E-04	5.709	-5.83E-04	5.83E-04
5.71	-3.66E-04	3.66E-04	5.71	-4.36E-04	4.36E-04	5.71	-4.93E-04	4.93E-04	5.71	-5.41E-04	5.41E-04	5.71	-5.83E-04	5.83E-04
5.711	-3.66E-04	3.66E-04	5.711	-4.36E-04	4.36E-04	5.711	-4.93E-04	4.93E-04	5.711	-5.41E-04	5.41E-04	5.711	-5.82E-04	5.82E-04
5.712	-3.66E-04	3.66E-04	5.712	-4.36E-04	4.36E-04	5.712	-4.93E-04	4.93E-04	5.712	-5.41E-04	5.41E-04	5.712	-5.82E-04	5.82E-04
5.713	-3.66E-04	3.66E-04	5.713	-4.36E-04	4.36E-04	5.713	-4.93E-04	4.93E-04	5.713	-5.41E-04	5.41E-04	5.713	-5.82E-04	5.82E-04
5.714	-3.65E-04	3.65E-04	5.714	-4.35E-04	4.35E-04	5.714	-4.93E-04	4.93E-04	5.714	-5.41E-04	5.41E-04	5.714	-5.82E-04	5.82E-04
5.715	-3.65E-04	3.65E-04	5.715	-4.35E-04	4.35E-04	5.715	-4.92E-04	4.92E-04	5.715	-5.40E-04	5.40E-04	5.715	-5.82E-04	5.82E-04
5.716	-3.65E-04	3.65E-04	5.716	-4.35E-04	4.35E-04	5.716	-4.92E-04	4.92E-04	5.716	-5.40E-04	5.40E-04	5.716	-5.81E-04	5.81E-04
5.717	-3.65E-04	3.65E-04	5.717	-4.35E-04	4.35E-04	5.717	-4.92E-04	4.92E-04	5.717	-5.40E-04	5.40E-04	5.717	-5.81E-04	5.81E-04
5.718	-3.65E-04	3.65E-04	5.718	-4.35E-04	4.35E-04	5.718	-4.92E-04	4.92E-04	5.718	-5.40E-04	5.40E-04	5.718	-5.81E-04	5.81E-04
5.719	-3.65E-04	3.65E-04	5.719	-4.35E-04	4.35E-04	5.719	-4.92E-04	4.92E-04	5.719	-5.40E-04	5.40E-04	5.719	-5.81E-04	5.81E-04
5.72	-3.65E-04	3.65E-04	5.72	-4.35E-04	4.35E-04	5.72	-4.92E-04	4.92E-04	5.72	-5.39E-04	5.39E-04	5.72	-5.80E-04	5.80E-04
5.721	-3.64E-04	3.64E-04	5.721	-4.34E-04	4.34E-04	5.721	-4.91E-04	4.91E-04	5.721	-5.39E-04	5.39E-04	5.721	-5.80E-04	5.80E-04
5.722	-3.64E-04	3.64E-04	5.722	-4.34E-04	4.34E-04	5.722	-4.91E-04	4.91E-04	5.722	-5.39E-04	5.39E-04	5.722	-5.80E-04	5.80E-04
5.723	-3.64E-04	3.64E-04	5.723	-4.34E-04	4.34E-04	5.723	-4.91E-04	4.91E-04	5.723	-5.39E-04	5.39E-04	5.723	-5.80E-04	5.80E-04
5.724	-3.64E-04	3.64E-04	5.724	-4.34E-04	4.34E-04	5.724	-4.91E-04	4.91E-04	5.724	-5.39E-04	5.39E-04	5.724	-5.80E-04	5.80E-04
5.725	-3.64E-04	3.64E-04	5.725	-4.34E-04	4.34E-04	5.725	-4.91E-04	4.91E-04	5.725	-5.39E-04	5.39E-04	5.725	-5.79E-04	5.79E-04
5.726	-3.64E-04	3.64E-04	5.726	-4.34E-04	4.34E-04	5.726	-4.91E-04	4.91E-04	5.726	-5.38E-04	5.38E-04	5.726	-5.79E-04	5.79E-04
5.727	-3.64E-04	3.64E-04	5.727	-4.33E-04	4.33E-04	5.727	-4.90E-04	4.90E-04	5.727	-5.38E-04	5.38E-04	5.727	-5.79E-04	5.79E-04
5.728	-3.64E-04	3.64E-04	5.728	-4.33E-04	4.33E-04	5.728	-4.90E-04	4.90E-04	5.728	-5.38E-04	5.38E-04	5.728	-5.79E-04	5.79E-04
5.729	-3.63E-04	3.63E-04	5.729	-4.33E-04	4.33E-04	5.729	-4.90E-04	4.90E-04	5.729	-5.38E-04	5.38E-04	5.729	-5.79E-04	5.79E-04
5.73	-3.63E-04	3.63E-04	5.73	-4.33E-04	4.33E-04	5.73	-4.90E-04	4.90E-04	5.73	-5.38E-04	5.38E-04	5.73	-5.78E-04	5.78E-04
5.731	-3.63E-04	3.63E-04	5.731	-4.33E-04	4.33E-04	5.731	-4.90E-04	4.90E-04	5.731	-5.37E-04	5.37E-04	5.731	-5.78E-04	5.78E-04
5.732	-3.63E-04	3.63E-04	5.732	-4.33E-04	4.33E-04	5.732	-4.90E-04	4.90E-04	5.732	-5.37E-04	5.37E-04	5.732	-5.78E-04	5.78E-04
5.733	-3.63E-04	3.63E-04	5.733	-4.33E-04	4.33E-04	5.733	-4.89E-04	4.89E-04	5.733	-5.37E-04	5.37E-04	5.733	-5.78E-04	5.78E-04
5.734	-3.63E-04	3.63E-04	5.734	-4.32E-04	4.32E-04	5.734	-4.89E-04	4.89E-04	5.734	-5.37E-04	5.37E-04	5.734	-5.78E-04	5.78E-04
5.735	-3.63E-04	3.63E-04	5.735	-4.32E-04	4.32E-04	5.735	-4.89E-04	4.89E-04	5.735	-5.37E-04	5.37E-04	5.735	-5.77E-04	5.77E-04
5.736	-3.63E-04	3.63E-04	5.736	-4.32E-04	4.32E-04	5.736	-4.89E-04	4.89E-04	5.736	-5.36E-04	5.36E-04	5.736	-5.77E-04	5.77E-04
5.737	-3.62E-04	3.62E-04	5.737	-4.32E-04	4.32E-04	5.737	-4.89E-04	4.89E-04	5.737	-5.36E-04	5.36E-04	5.737	-5.77E-04	5.77E-04
5.738	-3.62E-04	3.62E-04	5.738	-4.32E-04	4.32E-04	5.738	-4.89E-04	4.89E-04	5.738	-5.36E-04	5.36E-04	5.738	-5.77E-04	5.77E-04
5.739	-3.62E-04	3.62E-04	5.739	-4.32E-04	4.32E-04	5.739	-4.88E-04	4.88E-04	5.739	-5.36E-04	5.36E-04	5.739	-5.77E-04	5.77E-04
5.74	-3.62E-04	3.62E-04	5.74	-4.31E-04	4.31E-04	5.74	-4.88E-04	4.88E-04	5.74	-5.36E-04	5.36E-04	5.74	-5.76E-04	5.76E-04
5.741	-3.62E-04	3.62E-04	5.741	-4.31E-04	4.31E-04	5.741	-4.88E-04	4.88E-04	5.741	-5.36E-04	5.36E-04	5.741	-5.76E-04	5.76E-04
5.742	-3.62E-04	3.62E-04	5.742	-4.31E-04	4.31E-04	5.742	-4.88E-04	4.88E-04	5.742	-5.35E-04	5.35E-04	5.742	-5.76E-04	5.76E-04
5.743	-3.62E-04	3.62E-04	5.743	-4.31E-04	4.31E-04	5.743	-4.88E-04	4.88E-04	5.743	-5.35E-04	5.35E-04	5.743	-5.76E-04	5.76E-04
5.744	-3.62E-04	3.62E-04	5.744	-4.31E-04	4.31E-04	5.744	-4.88E-04	4.88E-04	5.744	-5.35E-04	5.35E-04	5.744	-5.76E-04	5.76E-04
5.745	-3.61E-04	3.61E-04	5.745	-4.31E-04	4.31E-04	5.745	-4.87E-04	4.87E-04	5.745	-5.35E-04	5.35E-04	5.745	-5.75E-04	5.75E-04
5.746	-3.61E-04	3.61E-04	5.746	-4.31E-04	4.31E-04	5.746	-4.87E-04	4.87E-04	5.746	-5.35E-04	5.35E-04	5.746	-5.75E-04	5.75E-04
5.747	-3.61E-04	3.61E-04	5.747	-4.30E-04	4.30E-04	5.747	-4.87E-04	4.87E-04	5.747	-5.34E-04	5.34E-04	5.747	-5.75E-04	5.75E-04
5.748	-3.61E-04	3.61E-04	5.748	-4.30E-04	4.30E-04	5.748	-4.87E-04	4.87E-04	5.748	-5.34E-04	5.34E-04	5.748	-5.75E-04	5.75E-04
5.749	-3.61E-04	3.61E-04	5.749	-4.30E-04	4.30E-04	5.749	-4.87E-04	4.87E-04	5.749	-5.34E-04	5.34E-04	5.749	-5.75E-04	5.75E-04
5.75	-3.61E-04	3.61E-04	5.75	-4.30E-04	4.30E-04	5.75	-4.87E-04	4.87E-04	5.75	-5.34E-04	5.34E-04	5.75	-5.74E-04	5.74E-04
5.751	-3.61E-04	3.61E-04	5.751	-4.30E-04	4.30E-04	5.751	-4.86E-04	4.86E-04	5.751	-5.34E-04	5.34E-04	5.751	-5.74E-04	5.74E-04
5.752	-3.61E-04	3.61E-04	5.752	-4.30E-04	4.30E-04	5.752	-4.86E-04	4.86E-04	5.752	-5.34E-04	5.34E-04	5.752	-5.74E-04	5.74E-04
5.753	-3.60E-04	3.60E-04	5.753	-4.30E-04	4.30E-04	5.753	-4.86E-04	4.86E-04	5.753	-5.33E-04	5.33E-04	5.753	-5.74E-04	5.74E-04
5.754	-3.60E-04	3.60E-04	5.754	-4.29E-04	4.29E-04	5.754	-4.86E-04	4.86E-04	5.754	-5.33E-04	5.33E-04	5.754	-5.74E-04	5.74E-04
5.755	-3.60E-04	3.60E-04	5.755	-4.29E-04	4.29E-04	5.755	-4.86E-04	4.86E-04	5.755	-5.33E-04	5.33E-04	5.755	-5.73E-04	5.73E-04
5.756	-3.60E-04	3.60E-04	5.756	-4.29E-04	4.29E-04	5.756	-4.85E-04	4.85E-04	5.756	-5.33E-04	5.33E-04	5.756	-5.73E-04	5.73E-04
5.757	-3.60E-04	3.60E-04	5.757	-4.29E-04	4.29E-04	5.757	-4.85E-04	4.85E-04	5.757	-5.33E-04	5.33E-04	5.757	-5.73E-04	5.73E-04
5.758	-3.60E-04	3.60E-04	5.758	-4.29E-04	4.29E-04	5.758	-4.85E-04	4.85E-04	5.758	-5.32E-04	5.32E-04	5.758	-5.73E-04	5.73E-04
5.759	-3.60E-04	3.60E-04	5.759	-4.29E-04	4.29E-04	5.759	-4.85E-04	4.85E-04	5.759	-5.32E-04	5.32E-04	5.759	-5.73E-04	5.73E-04
5.76	-3.60E-04	3.60E-04	5.76	-4.28E-04	4.28E-04	5.76	-4.85E-04	4.85E-04	5.76	-5.32E-04	5.32E-04	5.76	-5.72E-04	5.72E-04
5.761	-3.59E-04	3.59E-04	5.761	-4.28E-04	4.28E-04	5.761	-4.85E-04	4.85E-04	5.761	-5.32E-04	5.32E-04	5.761	-5.72E-04	5.72E-04
5.762	-3.59E-04	3.59E-04	5.762	-4.28E-04	4.28E-04	5.762	-4.84E-04	4.84E-04	5.762	-5.32E-04	5.32E-04	5.762	-5.72E-04	5.72E-04
5.763	-3.59E-04	3.59E-04	5.763	-4.28E-04	4.28E-04	5.763	-4.84E-04	4.84E-04	5.763	-5.31E-04	5.31E-04	5.763	-5.72E-04	5.72E-04
5.764	-3.59E-04	3.59E-04	5.764	-4.28E-04	4.28E-04	5.764	-4.84E-04	4.84E-04	5.764	-5.31E-04	5.31E-04	5.764	-5.72E-04	5.72E-04
5.765	-3.59E-04	3.59E-04	5.765	-4.28E-04	4.28E-04	5.765	-4.84E-04	4.84E-04	5.765	-5.31E-04	5.31E-04	5.765	-5.71E-04	5.71E-04
5.766	-3.59E-04	3.59E-04	5.766	-4.28E-04	4.28E-04	5.766	-4.84E-04	4.84E-04	5.766	-5.31E-04	5.31E-04	5.766	-5.71E-04	5.71E-04
5.767	-3.59E-04	3.59E-04	5.767	-4.27E-04	4.27E-04	5.767	-4.84E-04	4.84E-04	5.767	-5.31E-04	5.31E-04	5.767	-5.71E-04	5.71E-04
5.768	-3.59E-04	3.59E-04	5.768	-4.27E-04	4.27E-04	5.768	-4.83E-04	4.83E-04	5.768	-5.31E-04	5.31E-04	5.768	-5.71E-04	5.71E-04
5.769	-3.58E-04	3.58E-04	5.769	-4.27E-04	4.27E-04	5.769	-4.83E-04	4.83E-04	5.769	-5.30E-04	5.30E-04	5.769	-5.71E-04	5.71E-04
5.77	-3.58E-04	3.58E-04	5.77	-4.27E-04	4.27E-04	5.77	-4.83E-04	4.83E-04	5.77	-5.30E-04	5.30E-04	5.77	-5.70E-04	5.70E-04
5.771	-3.58E-04	3.58E-04	5.771	-4.27E-04	4.27E-04	5.771	-4.83E-04	4.83E-04	5.771	-5.30E-04	5.30E-04	5.771	-5.70E-04	5.70E-04
5.772	-3.58E-04	3.58E-04	5.772	-4.27E-04	4.27E-04	5.772	-4.83E-04	4.83E-04	5.772	-5.30E-04	5.30E-04	5.772	-5.70E-04	5.70E-04

5.773	-3.58E-04	3.58E-04	5.773	-4.27E-04	4.27E-04	5.773	-4.83E-04	4.83E-04	5.773	-5.30E-04	5.30E-04	5.773	-5.70E-04	5.70E-04
5.774	-3.58E-04	3.58E-04	5.774	-4.26E-04	4.26E-04	5.774	-4.82E-04	4.82E-04	5.774	-5.29E-04	5.29E-04	5.774	-5.70E-04	5.70E-04
5.775	-3.58E-04	3.58E-04	5.775	-4.26E-04	4.26E-04	5.775	-4.82E-04	4.82E-04	5.775	-5.29E-04	5.29E-04	5.775	-5.69E-04	5.69E-04
5.776	-3.58E-04	3.58E-04	5.776	-4.26E-04	4.26E-04	5.776	-4.82E-04	4.82E-04	5.776	-5.29E-04	5.29E-04	5.776	-5.69E-04	5.69E-04
5.777	-3.57E-04	3.57E-04	5.777	-4.26E-04	4.26E-04	5.777	-4.82E-04	4.82E-04	5.777	-5.29E-04	5.29E-04	5.777	-5.69E-04	5.69E-04
5.778	-3.57E-04	3.57E-04	5.778	-4.26E-04	4.26E-04	5.778	-4.82E-04	4.82E-04	5.778	-5.29E-04	5.29E-04	5.778	-5.69E-04	5.69E-04
5.779	-3.57E-04	3.57E-04	5.779	-4.26E-04	4.26E-04	5.779	-4.82E-04	4.82E-04	5.779	-5.29E-04	5.29E-04	5.779	-5.69E-04	5.69E-04
5.78	-3.57E-04	3.57E-04	5.78	-4.26E-04	4.26E-04	5.78	-4.81E-04	4.81E-04	5.78	-5.28E-04	5.28E-04	5.78	-5.69E-04	5.69E-04
5.781	-3.57E-04	3.57E-04	5.781	-4.25E-04	4.25E-04	5.781	-4.81E-04	4.81E-04	5.781	-5.28E-04	5.28E-04	5.781	-5.68E-04	5.68E-04
5.782	-3.57E-04	3.57E-04	5.782	-4.25E-04	4.25E-04	5.782	-4.81E-04	4.81E-04	5.782	-5.28E-04	5.28E-04	5.782	-5.68E-04	5.68E-04
5.783	-3.57E-04	3.57E-04	5.783	-4.25E-04	4.25E-04	5.783	-4.81E-04	4.81E-04	5.783	-5.28E-04	5.28E-04	5.783	-5.68E-04	5.68E-04
5.784	-3.57E-04	3.57E-04	5.784	-4.25E-04	4.25E-04	5.784	-4.81E-04	4.81E-04	5.784	-5.28E-04	5.28E-04	5.784	-5.68E-04	5.68E-04
5.785	-3.56E-04	3.56E-04	5.785	-4.25E-04	4.25E-04	5.785	-4.81E-04	4.81E-04	5.785	-5.27E-04	5.27E-04	5.785	-5.68E-04	5.68E-04
5.786	-3.56E-04	3.56E-04	5.786	-4.25E-04	4.25E-04	5.786	-4.80E-04	4.80E-04	5.786	-5.27E-04	5.27E-04	5.786	-5.67E-04	5.67E-04
5.787	-3.56E-04	3.56E-04	5.787	-4.25E-04	4.25E-04	5.787	-4.80E-04	4.80E-04	5.787	-5.27E-04	5.27E-04	5.787	-5.67E-04	5.67E-04
5.788	-3.56E-04	3.56E-04	5.788	-4.24E-04	4.24E-04	5.788	-4.80E-04	4.80E-04	5.788	-5.27E-04	5.27E-04	5.788	-5.67E-04	5.67E-04
5.789	-3.56E-04	3.56E-04	5.789	-4.24E-04	4.24E-04	5.789	-4.80E-04	4.80E-04	5.789	-5.27E-04	5.27E-04	5.789	-5.67E-04	5.67E-04
5.79	-3.56E-04	3.56E-04	5.79	-4.24E-04	4.24E-04	5.79	-4.80E-04	4.80E-04	5.79	-5.27E-04	5.27E-04	5.79	-5.67E-04	5.67E-04
5.791	-3.56E-04	3.56E-04	5.791	-4.24E-04	4.24E-04	5.791	-4.80E-04	4.80E-04	5.791	-5.26E-04	5.26E-04	5.791	-5.66E-04	5.66E-04
5.792	-3.56E-04	3.56E-04	5.792	-4.24E-04	4.24E-04	5.792	-4.79E-04	4.79E-04	5.792	-5.26E-04	5.26E-04	5.792	-5.66E-04	5.66E-04
5.793	-3.55E-04	3.55E-04	5.793	-4.24E-04	4.24E-04	5.793	-4.79E-04	4.79E-04	5.793	-5.26E-04	5.26E-04	5.793	-5.66E-04	5.66E-04
5.794	-3.55E-04	3.55E-04	5.794	-4.23E-04	4.23E-04	5.794	-4.79E-04	4.79E-04	5.794	-5.26E-04	5.26E-04	5.794	-5.66E-04	5.66E-04
5.795	-3.55E-04	3.55E-04	5.795	-4.23E-04	4.23E-04	5.795	-4.79E-04	4.79E-04	5.795	-5.26E-04	5.26E-04	5.795	-5.66E-04	5.66E-04
5.796	-3.55E-04	3.55E-04	5.796	-4.23E-04	4.23E-04	5.796	-4.79E-04	4.79E-04	5.796	-5.25E-04	5.25E-04	5.796	-5.65E-04	5.65E-04
5.797	-3.55E-04	3.55E-04	5.797	-4.23E-04	4.23E-04	5.797	-4.79E-04	4.79E-04	5.797	-5.25E-04	5.25E-04	5.797	-5.65E-04	5.65E-04
5.798	-3.55E-04	3.55E-04	5.798	-4.23E-04	4.23E-04	5.798	-4.78E-04	4.78E-04	5.798	-5.25E-04	5.25E-04	5.798	-5.65E-04	5.65E-04
5.799	-3.55E-04	3.55E-04	5.799	-4.23E-04	4.23E-04	5.799	-4.78E-04	4.78E-04	5.799	-5.25E-04	5.25E-04	5.799	-5.65E-04	5.65E-04
5.8	-3.55E-04	3.55E-04	5.8	-4.23E-04	4.23E-04	5.8	-4.78E-04	4.78E-04	5.8	-5.25E-04	5.25E-04	5.8	-5.65E-04	5.65E-04
5.801	-3.55E-04	3.55E-04	5.801	-4.22E-04	4.22E-04	5.801	-4.78E-04	4.78E-04	5.801	-5.25E-04	5.25E-04	5.801	-5.64E-04	5.64E-04
5.802	-3.54E-04	3.54E-04	5.802	-4.22E-04	4.22E-04	5.802	-4.78E-04	4.78E-04	5.802	-5.24E-04	5.24E-04	5.802	-5.64E-04	5.64E-04
5.803	-3.54E-04	3.54E-04	5.803	-4.22E-04	4.22E-04	5.803	-4.78E-04	4.78E-04	5.803	-5.24E-04	5.24E-04	5.803	-5.64E-04	5.64E-04
5.804	-3.54E-04	3.54E-04	5.804	-4.22E-04	4.22E-04	5.804	-4.77E-04	4.77E-04	5.804	-5.24E-04	5.24E-04	5.804	-5.64E-04	5.64E-04
5.805	-3.54E-04	3.54E-04	5.805	-4.22E-04	4.22E-04	5.805	-4.77E-04	4.77E-04	5.805	-5.24E-04	5.24E-04	5.805	-5.64E-04	5.64E-04
5.806	-3.54E-04	3.54E-04	5.806	-4.22E-04	4.22E-04	5.806	-4.77E-04	4.77E-04	5.806	-5.24E-04	5.24E-04	5.806	-5.63E-04	5.63E-04
5.807	-3.54E-04	3.54E-04	5.807	-4.22E-04	4.22E-04	5.807	-4.77E-04	4.77E-04	5.807	-5.23E-04	5.23E-04	5.807	-5.63E-04	5.63E-04
5.808	-3.54E-04	3.54E-04	5.808	-4.21E-04	4.21E-04	5.808	-4.77E-04	4.77E-04	5.808	-5.23E-04	5.23E-04	5.808	-5.63E-04	5.63E-04
5.809	-3.54E-04	3.54E-04	5.809	-4.21E-04	4.21E-04	5.809	-4.77E-04	4.77E-04	5.809	-5.23E-04	5.23E-04	5.809	-5.63E-04	5.63E-04
5.81	-3.53E-04	3.53E-04	5.81	-4.21E-04	4.21E-04	5.81	-4.77E-04	4.77E-04	5.81	-5.23E-04	5.23E-04	5.81	-5.63E-04	5.63E-04
5.811	-3.53E-04	3.53E-04	5.811	-4.21E-04	4.21E-04	5.811	-4.76E-04	4.76E-04	5.811	-5.23E-04	5.23E-04	5.811	-5.62E-04	5.62E-04
5.812	-3.53E-04	3.53E-04	5.812	-4.21E-04	4.21E-04	5.812	-4.76E-04	4.76E-04	5.812	-5.23E-04	5.23E-04	5.812	-5.62E-04	5.62E-04
5.813	-3.53E-04	3.53E-04	5.813	-4.21E-04	4.21E-04	5.813	-4.76E-04	4.76E-04	5.813	-5.22E-04	5.22E-04	5.813	-5.62E-04	5.62E-04
5.814	-3.53E-04	3.53E-04	5.814	-4.21E-04	4.21E-04	5.814	-4.76E-04	4.76E-04	5.814	-5.22E-04	5.22E-04	5.814	-5.62E-04	5.62E-04
5.815	-3.53E-04	3.53E-04	5.815	-4.20E-04	4.20E-04	5.815	-4.76E-04	4.76E-04	5.815	-5.22E-04	5.22E-04	5.815	-5.62E-04	5.62E-04
5.816	-3.53E-04	3.53E-04	5.816	-4.20E-04	4.20E-04	5.816	-4.76E-04	4.76E-04	5.816	-5.22E-04	5.22E-04	5.816	-5.61E-04	5.61E-04
5.817	-3.53E-04	3.53E-04	5.817	-4.20E-04	4.20E-04	5.817	-4.75E-04	4.75E-04	5.817	-5.22E-04	5.22E-04	5.817	-5.61E-04	5.61E-04
5.818	-3.52E-04	3.52E-04	5.818	-4.20E-04	4.20E-04	5.818	-4.75E-04	4.75E-04	5.818	-5.21E-04	5.21E-04	5.818	-5.61E-04	5.61E-04
5.819	-3.52E-04	3.52E-04	5.819	-4.20E-04	4.20E-04	5.819	-4.75E-04	4.75E-04	5.819	-5.21E-04	5.21E-04	5.819	-5.61E-04	5.61E-04
5.82	-3.52E-04	3.52E-04	5.82	-4.20E-04	4.20E-04	5.82	-4.75E-04	4.75E-04	5.82	-5.21E-04	5.21E-04	5.82	-5.61E-04	5.61E-04
5.821	-3.52E-04	3.52E-04	5.821	-4.20E-04	4.20E-04	5.821	-4.75E-04	4.75E-04	5.821	-5.21E-04	5.21E-04	5.821	-5.61E-04	5.61E-04
5.822	-3.52E-04	3.52E-04	5.822	-4.19E-04	4.19E-04	5.822	-4.75E-04	4.75E-04	5.822	-5.21E-04	5.21E-04	5.822	-5.60E-04	5.60E-04
5.823	-3.52E-04	3.52E-04	5.823	-4.19E-04	4.19E-04	5.823	-4.74E-04	4.74E-04	5.823	-5.21E-04	5.21E-04	5.823	-5.60E-04	5.60E-04
5.824	-3.52E-04	3.52E-04	5.824	-4.19E-04	4.19E-04	5.824	-4.74E-04	4.74E-04	5.824	-5.20E-04	5.20E-04	5.824	-5.60E-04	5.60E-04
5.825	-3.52E-04	3.52E-04	5.825	-4.19E-04	4.19E-04	5.825	-4.74E-04	4.74E-04	5.825	-5.20E-04	5.20E-04	5.825	-5.60E-04	5.60E-04
5.826	-3.51E-04	3.51E-04	5.826	-4.19E-04	4.19E-04	5.826	-4.74E-04	4.74E-04	5.826	-5.20E-04	5.20E-04	5.826	-5.60E-04	5.60E-04
5.827	-3.51E-04	3.51E-04	5.827	-4.19E-04	4.19E-04	5.827	-4.74E-04	4.74E-04	5.827	-5.20E-04	5.20E-04	5.827	-5.59E-04	5.59E-04
5.828	-3.51E-04	3.51E-04	5.828	-4.19E-04	4.19E-04	5.828	-4.74E-04	4.74E-04	5.828	-5.20E-04	5.20E-04	5.828	-5.59E-04	5.59E-04
5.829	-3.51E-04	3.51E-04	5.829	-4.18E-04	4.18E-04	5.829	-4.73E-04	4.73E-04	5.829	-5.20E-04	5.20E-04	5.829	-5.59E-04	5.59E-04
5.83	-3.51E-04	3.51E-04	5.83	-4.18E-04	4.18E-04	5.83	-4.73E-04	4.73E-04	5.83	-5.19E-04	5.19E-04	5.83	-5.59E-04	5.59E-04
5.831	-3.51E-04	3.51E-04	5.831	-4.18E-04	4.18E-04	5.831	-4.73E-04	4.73E-04	5.831	-5.19E-04	5.19E-04	5.831	-5.59E-04	5.59E-04
5.832	-3.51E-04	3.51E-04	5.832	-4.18E-04	4.18E-04	5.832	-4.73E-04	4.73E-04	5.832	-5.19E-04	5.19E-04	5.832	-5.58E-04	5.58E-04
5.833	-3.51E-04	3.51E-04	5.833	-4.18E-04	4.18E-04	5.833	-4.73E-04	4.73E-04	5.833	-5.19E-04	5.19E-04	5.833	-5.58E-04	5.58E-04
5.834	-3.51E-04	3.51E-04	5.834	-4.18E-04	4.18E-04	5.834	-4.73E-04	4.73E-04	5.834	-5.19E-04	5.19E-04	5.834	-5.58E-04	5.58E-04
5.835	-3.50E-04	3.50E-04	5.835	-4.18E-04	4.18E-04	5.835	-4.72E-04	4.72E-04	5.835	-5.18E-04	5.18E-04	5.835	-5.58E-04	5.58E-04
5.836	-3.50E-04	3.50E-04	5.836	-4.17E-04	4.17E-04	5.836	-4.72E-04	4.72E-04	5.836	-5.18E-04	5.18E-04	5.836	-5.58E-04	5.58E-04
5.837	-3.50E-04	3.50E-04	5.837	-4.17E-04	4.17E-04	5.837	-4.72E-04	4.72E-04	5.837	-5.18E-04	5.18E-04	5.837	-5.57E-04	5.57E-04
5.838	-3.50E-04	3.50E-04	5.838	-4.17E-04	4.17E-04	5.838	-4.72E-04	4.72E-04	5.838	-5.18E-04	5.18E-04	5.838	-5.57E-04	5.57E-04
5.839	-3.50E-04	3.50E-04	5.839	-4.17E-04	4.17E-04	5.839	-4.72E-04	4.72E-04	5.839	-5.18E-04	5.18E-04	5.839	-5.57E-04	5.57E-04
5.84	-3.50E-04	3.50E-04	5.84	-4.17E-04	4.17E-04	5.84	-4.72E-04	4.72E-04	5.84	-5.18E-04	5.18E-04	5.84	-5.57E-04	5.57E-04
5.841	-3.50E-04	3.50E-04	5.841	-4.17E-04	4.17E-04	5.841	-4.71E-04	4.71E-04	5.841	-5.17E-04	5.17E-04	5.841	-5.57E-04	5.57E-04
5.842	-3.50E-04	3.50E-04	5.842	-4.17E-04	4.17E-04	5.842	-4.71E-04	4.71E-04	5.842	-5.17E-04	5.17E-04	5.842	-5.57E-04	5.57E-04
5.843	-3.49E-04	3.49E-04	5.843	-4.16E-04	4.16E-04	5.843	-4.71E-04	4.71E-04	5.843	-5.17E-04	5.17E-04	5.843	-5.56E-04	5.56E-04
5.844	-3.49E-04	3.49E-04	5.844	-4.16E-04	4.16E-04	5.844	-4.71E-04	4.71E-04	5.844	-5.17E-04	5.17E-04	5.844	-5.56E-04	5.56E-04
5.845	-3.49E-04	3.49E-04	5.845	-4.16E-04	4.16E-04	5.845	-4.71E-04	4.71E-04	5.845	-5.17E-04	5.17E-04	5.845	-5.56E-04	5.56E-04
5.846	-3.49E-04	3.49E-04	5.846	-4.16E-04	4.16E-04	5.846	-4.71E-04	4.71E-04	5.846	-5.16E-04	5.16E-04	5.846	-5.56E-04	5.56E-04
5.847	-3.49E-04	3.49E-04	5.847	-4.16E-04	4.16E-04	5.847	-4.70E-04	4.70E-04	5.847	-5.16E-04	5.16E-04	5.847	-5.56E-04	5.56E-04
5.848	-3.49E-04	3.49E-04	5.848	-4.16E-04	4.16E-04	5.848	-4.70E-04	4.70E-04	5.848	-5.16E-04	5.16E-04	5.848	-5.55E-04	5.55E-04
5.849	-3.49E-04	3.49E-04	5.849	-4.16E-04	4.16E-04	5.849	-4.70E-04	4.70E-04	5.849	-5.16E-04	5.16E-04	5.849	-5.55E-04	5.55E-04
5.85	-3.49E-04	3.49E-04	5.85	-4.15E-04	4.15E-04	5.85	-4.70E-04	4.70E-04	5.85	-5.16E-04	5.16E-04	5.85	-5.55E-04	5.55E-04
5.851	-3.48E-04	3.48E-04	5.851	-4.15E-04	4.15E-04	5.851	-4.70E-04	4.70E-04	5.851	-5.16E-04	5.16E-04	5.851	-5.55E-04	5.55E-04
5.852	-3.48E-04	3.48E-04	5.852	-4.15E-04	4.15E-04	5.852	-4.70E-04	4.70E-04	5.852	-5.15E-04	5.15E-04	5.852	-5.55E-04	5.55E-04
5.853	-3.48E-04	3.48E-04	5.853	-4.15E-04	4.15E-04	5.853	-4.70E-04	4.70E-04	5.853	-5.15E-04	5.15E-04	5.853	-5.54E-04	5.54E-04
5.854	-3.48E-04	3.48E-04	5.854	-4.15E-04	4.15E-04	5.854	-4.69E-04	4.69E-04	5.854	-5.15E-04	5.15E-04	5.854	-5.54E-04	5.54E-04
5.855	-3.48E-04	3.48E-04	5.855	-4.15E-04	4.15E-04	5.855	-4.69E-04	4.69E-04	5.855	-5.15E-04	5.15E-04	5.855	-5.54E-04	5.54E-04
5.856	-3.48E-04	3.48E-04	5.856	-4.15E-04	4.15E-04	5.856	-4.69E-04	4.69E-04	5.856	-5.15E-04	5.15E-04	5.856	-5.54E-04	5.54E-04
5.857	-3.48E-04	3.48E-04	5.857	-4.14E-04	4.14E-04	5.857	-4.69E-04	4.69E-04	5.857	-5.15E-04	5.15E-04	5.857	-5.54E-04	5.54E-04
5.858	-3.48E-04	3.48E-04	5.858	-4.14E-04	4.14E-04	5.858	-4.69E-04	4.69E-04	5.858	-5.14E-04	5.14E-04	5.858	-5.53E-04	5.53E-04
5.859	-3.48E-04	3.48E-04	5.859	-4.14E-04	4.14E-04	5.859	-4.69E-04	4.69E-04	5.859	-5.14E-04	5.14E-04	5.859	-5.53E-04	5.53E-04
5.86	-3.47E-04	3.47E-04	5.86	-4.14E-04	4.14E-04	5.86	-4.68E-04	4.68E-04	5.86	-5.14E-04	5.14E-04	5.86	-5.53E-04	5.53E-04
5.861	-3.47E-04	3.47E-04	5.861	-4.14E-04	4.14E-04	5.861	-4.68E-04	4.68E-04	5.861	-5.14E-04	5.14E-04	5.861	-5.53E-04	5.53E-04
5.862	-3.47E-04	3.47E-04	5.862	-4.14E-04	4.14E-04	5.862	-4.68E-04	4.68E-04	5.862	-5.14E-04	5.14E-04	5.862	-5.53E-04	5.53E-04
5.863	-3.47E-04	3.47E-04	5.863	-4.14E-04	4.14E-04	5.863	-4.68E-04	4.68E-04	5.863	-5.14E-04	5.14E-04	5.863	-5.53E-04	5.53E-04
5.864	-3.47E-04	3.47E-04	5.864	-4.13E-04	4.13E-04	5.864	-4.68E-04	4.68E-04	5.864	-5.13E-04	5.13E-04	5.864	-5.52E-04	5.52E-04
5.865	-3.47E-04	3.47E-04	5.865	-4.13E-04	4.13E-04	5.865	-4.68E-04	4.68E-04	5.865	-5.13E-04	5.13E-04	5.865	-5.52E-04	5.52E-04
5.866	-3.47E-04	3.47E-04	5.866	-4.13E-04	4.13E-04	5.866	-4.67E-04	4.67E-04	5.866	-5.13E-04	5.13E-04	5.866	-5.52E-04	5.52E-04
5.867	-3.47E-04	3.47E-04	5.867	-4.13E-04	4.13E-04	5.867	-4.67E-04	4.67E-04	5.867	-5.13E-04	5.13E-04	5.867	-5.52E-04	5.52E-04
5.868	-3.46E-04	3.46E-04	5.868	-4.13E-04	4.13E-04	5.868	-4.67E-04	4.67E-04	5.868	-5.13E-04	5.13E-04	5.868	-5.52E-04	5.52E-04
5.869	-3.46E-04	3.46E-04	5.869	-4.13E-04	4.13E-04	5.869	-4.67E-04	4.67E-04	5.869	-5.12E-04	5.12E-04	5.869	-5.51E-04	5.51E-04
5.87	-3.46E-04	3.46E-04	5.87	-4.13E-04	4.13E-04	5.87	-4.67E-04	4.67E-04	5.87	-5.12E-04	5.12E-04	5.87	-5.51E-04	5.51E-04
5.871	-3.46E-04	3.46E-04	5.871	-4.12E-04	4.12E-04	5.871	-4.67E-04	4.67E-04	5.871	-5.12E-04	5.12E-04	5.871	-5.51E-04	5.51E-04
5.872	-3.46E-04	3.46E-04	5.872	-4.12E-04	4.12E-04	5.872	-4.67E-04	4.67E-04	5.872	-5.12E-04	5.12E-04	5.872	-5.51E-04	5.51E-04
5.873	-3.46E-04	3.46E-04	5.873	-4.12E-04	4.12E-04	5.873	-4.66E-04	4.66E-04	5.873	-5.12E-04	5.12E-04	5.873	-5.51E-04	5.51E-04
5.874	-3.46E-04	3.46E-04	5.874	-4.12E-04	4.12E-04	5.874	-4.66E-04	4.66E-04	5.874	-5.12E-04	5.12E-04	5.874	-5.50E-04	5.50E-04
5.875	-3.46E-04	3.46E-04	5.875	-4.12E-04	4.12E-04	5.875	-4.66E-04	4.66E-04	5.875	-5.11E-04	5.11E-04	5.875	-5.50E-04	5.50E-04
5.876	-3.46E-04	3.46E-04	5.876	-4.12E-04	4.12E-04	5.876	-4.66E-04	4.66E-04	5.876	-5.11E-04	5.11E-04	5.876	-5.50E-04	5.50E-04
5.877	-3.45E-04	3.45E-04	5.877	-4.12E-04	4.12E-04	5.877	-4.66E-04	4.66E-04	5.877	-5.11E-04	5.11E-04	5.877	-5.50E-04	5.50E-04
5.878	-3.45E-04	3.45E-04	5.878	-4.11E-04	4.11E-04	5.878	-4.66E-04	4.66E-04	5.878	-5.11E-04	5.11E-04	5.878	-5.50E-04	5.50E-04
5.879	-3.45E-04	3.45E-04	5.879	-4.11E-04	4.11E-04	5.879	-4.65E-04	4.65E-04	5.879	-5.11E-04	5.11E-04	5.879	-5.50E-04	5.50E-04
5.88	-3.45E-04	3.45E-04	5.88	-4.11E-04	4.11E-04	5.88	-4.65E-04	4.65E-04	5.88	-5.11E-04	5.11E-04	5.88	-5.49E-04	5.49E-04
5.881	-3.45E-04	3.45E-04	5.881	-4.11E-04	4.11E-04	5.881	-4.65E-04	4.65E-04	5.881	-5.10E-04	5.10E-04	5.881	-5.49E-04	5.49E-04
5.882	-3.45E-04	3.45E-04	5.882	-4.11E-04	4.11E-04	5.882	-4.65E-04	4.65E-04	5.882	-5.10E-04	5.10E-04	5.882	-5.49E-04	5.49E-04
5.883	-3.45E-04	3.45E-04	5.883	-4.11E-04	4.11E-04	5.883	-4.65E-04	4.65E-04	5.883	-5.10E-04	5.10E-04	5.883	-5.49E-04	5.49E-04
5.884	-3.45E-04	3.45E-04	5.884	-4.11E-04	4.11E-04	5.884	-4.65E-04	4.65E-04	5.884	-5.10E-04	5.10E-04	5.884	-5.49E-04	5.49E-04
5.885	-3.44E-04	3.44E-04	5.885	-4.10E-04	4.10E-04	5.885	-4.64E-04	4.64E-04	5.885	-5.10E-04	5.10E-04	5.885	-5.48E-04	5.48E-04
5.886	-3.44E-04	3.44E-04	5.886	-4.10E-04	4.10E-04	5.886	-4.64E-04	4.64E-04	5.886	-5.09E-04	5.09E-04	5.886	-5.48E-04	5.48E-04
5.887	-3.44E-04	3.44E-04	5.887	-4.10E-04	4.10E-04	5.887	-4.64E-04	4.64E-04	5.887	-5.09E-04	5.09E-04	5.887	-5.48E-04	5.48E-04
5.888	-3.44E-04	3.44E-04	5.888	-4.10E-04	4.10E-04	5.888	-4.64E-04	4.64E-04	5.888	-5.09E-04	5.09E-04	5.888	-5.48E-04	5.48E-04
5.889	-3.44E-04	3.44E-04	5.889	-4.10E-04	4.10E-04	5.889	-4.64E-04	4.64E-04	5.889	-5.09E-04	5.09E-04	5.889	-5.48E-04	5.48E-04
5.89	-3.44E-04	3.44E-04	5.89	-4.10E-04	4.10E-04	5.89	-4.64E-04	4.64E-04	5.89	-5.09E-04	5.09E-04	5.89	-5.47E-04	5.47E-04
5.891	-3.44E-04	3.44E-04	5.891	-4.10E-04	4.10E-04	5.891	-4.63E-04	4.63E-04	5.891	-5.09E-04	5.09E-04	5.891	-5.47E-04	5.47E-04
5.892	-3.44E-04	3.44E-04	5.892	-4.10E-04	4.10E-04	5.892	-4.63E-04	4.63E-04	5.892	-5.08E-04	5.08E-04	5.892	-5.47E-04	5.47E-04
5.893	-3.44E-04	3.44E-04	5.893	-4.09E-04	4.09E-04	5.893	-4.63E-04	4.63E-04	5.893	-5.08E-04	5.08E-04	5.893	-5.47E-04	5.47E-04
5.894	-3.43E-04	3.43E-04	5.894	-4.09E-04	4.09E-04	5.894	-4.63E-04	4.63E-04	5.894	-5.08E-04	5.08E-04	5.894	-5.47E-04	5.47E-04
5.895	-3.43E-04	3.43E-04	5.895	-4.09E-04	4.09E-04	5.895	-4.63E-04	4.63E-04	5.895	-5.08E-04	5.08E-04	5.895	-5.47E-04	5.47E-04
5.896	-3.43E-04	3.43E-04	5.896	-4.09E-04	4.09E-04	5.896	-4.63E-04	4.63E-04	5.896	-5.08E-04	5.08E-04	5.896	-5.46E-04	5.46E-04
5.897	-3.43E-04	3.43E-04	5.897	-4.09E-04	4.09E-04	5.897	-4.63E-04	4.63E-04	5.897	-5.08E-04	5.08E-04	5.897	-5.46E-04	5.46E-04
5.898	-3.43E-04	3.43E-04	5.898	-4.09E-04	4.09E-04	5.898	-4.62E-04	4.62E-04	5.898	-5.07E-04	5.07E-04	5.898	-5.46E-04	5.46E-04
5.899	-3.43E-04	3.43E-04	5.899	-4.09E-04	4.09E-04	5.899	-4.62E-04	4.62E-04	5.899	-5.07E-04	5.07E-04	5.899	-5.46E-04	5.46E-04
5.9	-3.43E-04	3.43E-04	5.9	-4.08E-04	4.08E-04	5.9	-4.62E-04	4.62E-04	5.9	-5.07E-04	5.07E-04	5.9	-5.46E-04	5.46E-04
5.901	-3.43E-04	3.43E-04	5.901	-4.08E-04	4.08E-04	5.901	-4.62E-04	4.62E-04	5.901	-5.07E-04	5.07E-04	5.901	-5.45E-04	5.45E-04
5.902	-3.42E-04	3.42E-04	5.902	-4.08E-04	4.08E-04	5.902	-4.62E-04	4.62E-04	5.902	-5.07E-04	5.07E-04	5.902	-5.45E-04	5.45E-04
5.903	-3.42E-04	3.42E-04	5.903	-4.08E-04	4.08E-04	5.903	-4.62E-04	4.62E-04	5.903	-5.07E-04	5.07E-04	5.903	-5.45E-04	5.45E-04
5.904	-3.42E-04	3.42E-04	5.904	-4.08E-04	4.08E-04	5.904	-4.61E-04	4.61E-04	5.904	-5.06E-04	5.06E-04	5.904	-5.45E-04	5.45E-04
5.905	-3.42E-04	3.42E-04	5.905	-4.08E-04	4.08E-04	5.905	-4.61E-04	4.61E-04	5.905	-5.06E-04	5.06E-04	5.905	-5.45E-04	5.45E-04
5.906	-3.42E-04	3.42E-04	5.906	-4.08E-04	4.08E-04	5.906	-4.61E-04	4.61E-04	5.906	-5.06E-04	5.06E-04	5.906	-5.45E-04	5.45E-04
5.907	-3.42E-04	3.42E-04	5.907	-4.07E-04	4.07E-04	5.907	-4.61E-04	4.61E-04	5.907	-5.06E-04	5.06E-04	5.907	-5.44E-04	5.44E-04
5.908	-3.42E-04	3.42E-04	5.908	-4.07E-04	4.07E-04	5.908	-4.61E-04	4.61E-04	5.908	-5.06E-04	5.06E-04	5.908	-5.44E-04	5.44E-04
5.909	-3.42E-04	3.42E-04	5.909	-4.07E-04	4.07E-04	5.909	-4.61E-04	4.61E-04	5.909	-5.06E-04	5.06E-04	5.909	-5.44E-04	5.44E-04
5.91	-3.42E-04	3.42E-04	5.91	-4.07E-04	4.07E-04	5.91	-4.61E-04	4.61E-04	5.91	-5.05E-04	5.05E-04	5.91	-5.44E-04	5.44E-04
5.911	-3.41E-04	3.41E-04	5.911	-4.07E-04	4.07E-04	5.911	-4.60E-04	4.60E-04	5.911	-5.05E-04	5.05E-04	5.911	-5.44E-04	5.44E-04
5.912	-3.41E-04	3.41E-04	5.912	-4.07E-04	4.07E-04	5.912	-4.60E-04	4.60E-04	5.912	-5.05E-04	5.05E-04	5.912	-5.43E-04	5.43E-04
5.913	-3.41E-04	3.41E-04	5.913	-4.07E-04	4.07E-04	5.913	-4.60E-04	4.60E-04	5.913	-5.05E-04	5.05E-04	5.913	-5.43E-04	5.43E-04
5.914	-3.41E-04	3.41E-04	5.914	-4.06E-04	4.06E-04	5.914	-4.60E-04	4.60E-04	5.914	-5.05E-04	5.05E-04	5.914	-5.43E-04	5.43E-04
5.915	-3.41E-04	3.41E-04	5.915	-4.06E-04	4.06E-04	5.915	-4.60E-04	4.60E-04	5.915	-5.05E-04	5.05E-04	5.915	-5.43E-04	5.43E-04
5.916	-3.41E-04	3.41E-04	5.916	-4.06E-04	4.06E-04	5.916	-4.60E-04	4.60E-04	5.916	-5.04E-04	5.04E-04	5.916	-5.43E-04	5.43E-04
5.917	-3.41E-04	3.41E-04	5.917	-4.06E-04	4.06E-04	5.917	-4.59E-04	4.59E-04	5.917	-5.04E-04	5.04E-04	5.917	-5.42E-04	5.42E-04
5.918	-3.41E-04	3.41E-04	5.918	-4.06E-04	4.06E-04	5.918	-4.59E-04	4.59E-04	5.918	-5.04E-04	5.04E-04	5.918	-5.42E-04	5.42E-04
5.919	-3.41E-04	3.41E-04	5.919	-4.06E-04	4.06E-04	5.919	-4.59E-04	4.59E-04	5.919	-5.04E-04	5.04E-04	5.919	-5.42E-04	5.42E-04
5.92	-3.40E-04	3.40E-04	5.92	-4.06E-04	4.06E-04	5.92	-4.59E-04	4.59E-04	5.92	-5.04E-04	5.04E-04	5.92	-5.42E-04	5.42E-04
5.921	-3.40E-04	3.40E-04	5.921	-4.06E-04	4.06E-04	5.921	-4.59E-04	4.59E-04	5.921	-5.03E-04	5.03E-04	5.921	-5.42E-04	5.42E-04
5.922	-3.40E-04	3.40E-04	5.922	-4.05E-04	4.05E-04	5.922	-4.59E-04	4.59E-04	5.922	-5.03E-04	5.03E-04	5.922	-5.42E-04	5.42E-04
5.923	-3.40E-04	3.40E-04	5.923	-4.05E-04	4.05E-04	5.923	-4.59E-04	4.59E-04	5.923	-5.03E-04	5.03E-04	5.923	-5.41E-04	5.41E-04
5.924	-3.40E-04	3.40E-04	5.924	-4.05E-04	4.05E-04	5.924	-4.58E-04	4.58E-04	5.924	-5.03E-04	5.03E-04	5.924	-5.41E-04	5.41E-04
5.925	-3.40E-04	3.40E-04	5.925	-4.05E-04	4.05E-04	5.925	-4.58E-04	4.58E-04	5.925	-5.03E-04	5.03E-04	5.925	-5.41E-04	5.41E-04
5.926	-3.40E-04	3.40E-04	5.926	-4.05E-04	4.05E-04	5.926	-4.58E-04	4.58E-04	5.926	-5.03E-04	5.03E-04	5.926	-5.41E-04	5.41E-04
5.927	-3.40E-04	3.40E-04	5.927	-4.05E-04	4.05E-04	5.927	-4.58E-04	4.58E-04	5.927	-5.02E-04	5.02E-04	5.927	-5.41E-04	5.41E-04
5.928	-3.39E-04	3.39E-04	5.928	-4.05E-04	4.05E-04	5.928	-4.58E-04	4.58E-04	5.928	-5.02E-04	5.02E-04	5.928	-5.40E-04	5.40E-04
5.929	-3.39E-04	3.39E-04	5.929	-4.04E-04	4.04E-04	5.929	-4.58E-04	4.58E-04	5.929	-5.02E-04	5.02E-04	5.929	-5.40E-04	5.40E-04
5.93	-3.39E-04	3.39E-04	5.93	-4.04E-04	4.04E-04	5.93	-4.57E-04	4.57E-04	5.93	-5.02E-04	5.02E-04	5.93	-5.40E-04	5.40E-04
5.931	-3.39E-04	3.39E-04	5.931	-4.04E-04	4.04E-04	5.931	-4.57E-04	4.57E-04	5.931	-5.02E-04	5.02E-04	5.931	-5.40E-04	5.40E-04
5.932	-3.39E-04	3.39E-04	5.932	-4.04E-04	4.04E-04	5.932	-4.57E-04	4.57E-04	5.932	-5.02E-04	5.02E-04	5.932	-5.40E-04	5.40E-04
5.933	-3.39E-04	3.39E-04	5.933	-4.04E-04	4.04E-04	5.933	-4.57E-04	4.57E-04	5.933	-5.01E-04	5.01E-04	5.933	-5.40E-04	5.40E-04
5.934	-3.39E-04	3.39E-04	5.934	-4.04E-04	4.04E-04	5.934	-4.57E-04	4.57E-04	5.934	-5.01E-04	5.01E-04	5.934	-5.39E-04	5.39E-04
5.935	-3.39E-04	3.39E-04	5.935	-4.04E-04	4.04E-04	5.935	-4.57E-04	4.57E-04	5.935	-5.01E-04	5.01E-04	5.935	-5.39E-04	5.39E-04
5.936	-3.39E-04	3.39E-04	5.936	-4.03E-04	4.03E-04	5.936	-4.56E-04	4.56E-04	5.936	-5.01E-04	5.01E-04	5.936	-5.39E-04	5.39E-04
5.937	-3.38E-04	3.38E-04	5.937	-4.03E-04	4.03E-04	5.937	-4.56E-04	4.56E-04	5.937	-5.01E-04	5.01E-04	5.937	-5.39E-04	5.39E-04
5.938	-3.38E-04	3.38E-04	5.938	-4.03E-04	4.03E-04	5.938	-4.56E-04	4.56E-04	5.938	-5.01E-04	5.01E-04	5.938	-5.39E-04	5.39E-04
5.939	-3.38E-04	3.38E-04	5.939	-4.03E-04	4.03E-04	5.939	-4.56E-04	4.56E-04	5.939	-5.00E-04	5.00E-04	5.939	-5.38E-04	5.38E-04
5.94	-3.38E-04	3.38E-04	5.94	-4.03E-04	4.03E-04	5.94	-4.56E-04	4.56E-04	5.94	-5.00E-04	5.00E-04	5.94	-5.38E-04	5.38E-04
5.941	-3.38E-04	3.38E-04	5.941	-4.03E-04	4.03E-04	5.941	-4.56E-04	4.56E-04	5.941	-5.00E-04	5.00E-04	5.941	-5.38E-04	5.38E-04
5.942	-3.38E-04	3.38E-04	5.942	-4.03E-04	4.03E-04	5.942	-4.56E-04	4.56E-04	5.942	-5.00E-04	5.00E-04	5.942	-5.38E-04	5.38E-04
5.943	-3.38E-04	3.38E-04	5.943	-4.03E-04	4.03E-04	5.943	-4.55E-04	4.55E-04	5.943	-5.00E-04	5.00E-04	5.943	-5.38E-04	5.38E-04
5.944	-3.38E-04	3.38E-04	5.944	-4.02E-04	4.02E-04	5.944	-4.55E-04	4.55E-04	5.944	-5.00E-04	5.00E-04	5.944	-5.38E-04	5.38E-04
5.945	-3.38E-04	3.38E-04	5.945	-4.02E-04	4.02E-04	5.945	-4.55E-04	4.55E-04	5.945	-4.99E-04	4.99E-04	5.945	-5.37E-04	5.37E-04
5.946	-3.37E-04	3.37E-04	5.946	-4.02E-04	4.02E-04	5.946	-4.55E-04	4.55E-04	5.946	-4.99E-04	4.99E-04	5.946	-5.37E-04	5.37E-04
5.947	-3.37E-04	3.37E-04	5.947	-4.02E-04	4.02E-04	5.947	-4.55E-04	4.55E-04	5.947	-4.99E-04	4.99E-04	5.947	-5.37E-04	5.37E-04
5.948	-3.37E-04	3.37E-04	5.948	-4.02E-04	4.02E-04	5.948	-4.55E-04	4.55E-04	5.948	-4.99E-04	4.99E-04	5.948	-5.37E-04	5.37E-04
5.949	-3.37E-04	3.37E-04	5.949	-4.02E-04	4.02E-04	5.949	-4.55E-04	4.55E-04	5.949	-4.99E-04	4.99E-04	5.949	-5.37E-04	5.37E-04
5.95	-3.37E-04	3.37E-04	5.95	-4.02E-04	4.02E-04	5.95	-4.54E-04	4.54E-04	5.95	-4.99E-04	4.99E-04	5.95	-5.36E-04	5.36E-04
5.951	-3.37E-04	3.37E-04	5.951	-4.01E-04	4.01E-04	5.951	-4.54E-04	4.54E-04	5.951	-4.98E-04	4.98E-04	5.951	-5.36E-04	5.36E-04
5.952	-3.37E-04	3.37E-04	5.952	-4.01E-04	4.01E-04	5.952	-4.54E-04	4.54E-04	5.952	-4.98E-04	4.98E-04	5.952	-5.36E-04	5.36E-04
5.953	-3.37E-04	3.37E-04	5.953	-4.01E-04	4.01E-04	5.953	-4.54E-04	4.54E-04	5.953	-4.98E-04	4.98E-04	5.953	-5.36E-04	5.36E-04
5.954	-3.37E-04	3.37E-04	5.954	-4.01E-04	4.01E-04	5.954	-4.54E-04	4.54E-04	5.954	-4.98E-04	4.98E-04	5.954	-5.36E-04	5.36E-04
5.955	-3.36E-04	3.36E-04	5.955	-4.01E-04	4.01E-04	5.955	-4.54E-04	4.54E-04	5.955	-4.98E-04	4.98E-04	5.955	-5.36E-04	5.36E-04
5.956	-3.36E-04	3.36E-04	5.956	-4.01E-04	4.01E-04	5.956	-4.53E-04	4.53E-04	5.956	-4.98E-04	4.98E-04	5.956	-5.35E-04	5.35E-04
5.957	-3.36E-04	3.36E-04	5.957	-4.01E-04	4.01E-04	5.957	-4.53E-04	4.53E-04	5.957	-4.97E-04	4.97E-04	5.957	-5.35E-04	5.35E-04

5.958	-3.36E-04	3.36E-04	5.958	-4.00E-04	4.00E-04	5.958	-4.53E-04	4.53E-04	5.958	-4.97E-04	4.97E-04	5.958	-5.35E-04	5.35E-04
5.959	-3.36E-04	3.36E-04	5.959	-4.00E-04	4.00E-04	5.959	-4.53E-04	4.53E-04	5.959	-4.97E-04	4.97E-04	5.959	-5.35E-04	5.35E-04
5.96	-3.36E-04	3.36E-04	5.96	-4.00E-04	4.00E-04	5.96	-4.53E-04	4.53E-04	5.96	-4.97E-04	4.97E-04	5.96	-5.35E-04	5.35E-04
5.961	-3.36E-04	3.36E-04	5.961	-4.00E-04	4.00E-04	5.961	-4.53E-04	4.53E-04	5.961	-4.97E-04	4.97E-04	5.961	-5.35E-04	5.35E-04
5.962	-3.36E-04	3.36E-04	5.962	-4.00E-04	4.00E-04	5.962	-4.53E-04	4.53E-04	5.962	-4.97E-04	4.97E-04	5.962	-5.34E-04	5.34E-04
5.963	-3.36E-04	3.36E-04	5.963	-4.00E-04	4.00E-04	5.963	-4.52E-04	4.52E-04	5.963	-4.96E-04	4.96E-04	5.963	-5.34E-04	5.34E-04
5.964	-3.35E-04	3.35E-04	5.964	-4.00E-04	4.00E-04	5.964	-4.52E-04	4.52E-04	5.964	-4.96E-04	4.96E-04	5.964	-5.34E-04	5.34E-04
5.965	-3.35E-04	3.35E-04	5.965	-4.00E-04	4.00E-04	5.965	-4.52E-04	4.52E-04	5.965	-4.96E-04	4.96E-04	5.965	-5.34E-04	5.34E-04
5.966	-3.35E-04	3.35E-04	5.966	-3.99E-04	3.99E-04	5.966	-4.52E-04	4.52E-04	5.966	-4.96E-04	4.96E-04	5.966	-5.34E-04	5.34E-04
5.967	-3.35E-04	3.35E-04	5.967	-3.99E-04	3.99E-04	5.967	-4.52E-04	4.52E-04	5.967	-4.96E-04	4.96E-04	5.967	-5.33E-04	5.33E-04
5.968	-3.35E-04	3.35E-04	5.968	-3.99E-04	3.99E-04	5.968	-4.52E-04	4.52E-04	5.968	-4.96E-04	4.96E-04	5.968	-5.33E-04	5.33E-04
5.969	-3.35E-04	3.35E-04	5.969	-3.99E-04	3.99E-04	5.969	-4.51E-04	4.51E-04	5.969	-4.95E-04	4.95E-04	5.969	-5.33E-04	5.33E-04
5.97	-3.35E-04	3.35E-04	5.97	-3.99E-04	3.99E-04	5.97	-4.51E-04	4.51E-04	5.97	-4.95E-04	4.95E-04	5.97	-5.33E-04	5.33E-04
5.971	-3.35E-04	3.35E-04	5.971	-3.99E-04	3.99E-04	5.971	-4.51E-04	4.51E-04	5.971	-4.95E-04	4.95E-04	5.971	-5.33E-04	5.33E-04
5.972	-3.34E-04	3.34E-04	5.972	-3.99E-04	3.99E-04	5.972	-4.51E-04	4.51E-04	5.972	-4.95E-04	4.95E-04	5.972	-5.33E-04	5.33E-04
5.973	-3.34E-04	3.34E-04	5.973	-3.98E-04	3.98E-04	5.973	-4.51E-04	4.51E-04	5.973	-4.95E-04	4.95E-04	5.973	-5.32E-04	5.32E-04
5.974	-3.34E-04	3.34E-04	5.974	-3.98E-04	3.98E-04	5.974	-4.51E-04	4.51E-04	5.974	-4.95E-04	4.95E-04	5.974	-5.32E-04	5.32E-04
5.975	-3.34E-04	3.34E-04	5.975	-3.98E-04	3.98E-04	5.975	-4.51E-04	4.51E-04	5.975	-4.94E-04	4.94E-04	5.975	-5.32E-04	5.32E-04
5.976	-3.34E-04	3.34E-04	5.976	-3.98E-04	3.98E-04	5.976	-4.50E-04	4.50E-04	5.976	-4.94E-04	4.94E-04	5.976	-5.32E-04	5.32E-04
5.977	-3.34E-04	3.34E-04	5.977	-3.98E-04	3.98E-04	5.977	-4.50E-04	4.50E-04	5.977	-4.94E-04	4.94E-04	5.977	-5.32E-04	5.32E-04
5.978	-3.34E-04	3.34E-04	5.978	-3.98E-04	3.98E-04	5.978	-4.50E-04	4.50E-04	5.978	-4.94E-04	4.94E-04	5.978	-5.31E-04	5.31E-04
5.979	-3.34E-04	3.34E-04	5.979	-3.98E-04	3.98E-04	5.979	-4.50E-04	4.50E-04	5.979	-4.94E-04	4.94E-04	5.979	-5.31E-04	5.31E-04
5.98	-3.34E-04	3.34E-04	5.98	-3.98E-04	3.98E-04	5.98	-4.50E-04	4.50E-04	5.98	-4.94E-04	4.94E-04	5.98	-5.31E-04	5.31E-04
5.981	-3.33E-04	3.33E-04	5.981	-3.97E-04	3.97E-04	5.981	-4.50E-04	4.50E-04	5.981	-4.93E-04	4.93E-04	5.981	-5.31E-04	5.31E-04
5.982	-3.33E-04	3.33E-04	5.982	-3.97E-04	3.97E-04	5.982	-4.50E-04	4.50E-04	5.982	-4.93E-04	4.93E-04	5.982	-5.31E-04	5.31E-04
5.983	-3.33E-04	3.33E-04	5.983	-3.97E-04	3.97E-04	5.983	-4.49E-04	4.49E-04	5.983	-4.93E-04	4.93E-04	5.983	-5.31E-04	5.31E-04
5.984	-3.33E-04	3.33E-04	5.984	-3.97E-04	3.97E-04	5.984	-4.49E-04	4.49E-04	5.984	-4.93E-04	4.93E-04	5.984	-5.30E-04	5.30E-04
5.985	-3.33E-04	3.33E-04	5.985	-3.97E-04	3.97E-04	5.985	-4.49E-04	4.49E-04	5.985	-4.93E-04	4.93E-04	5.985	-5.30E-04	5.30E-04
5.986	-3.33E-04	3.33E-04	5.986	-3.97E-04	3.97E-04	5.986	-4.49E-04	4.49E-04	5.986	-4.93E-04	4.93E-04	5.986	-5.30E-04	5.30E-04
5.987	-3.33E-04	3.33E-04	5.987	-3.97E-04	3.97E-04	5.987	-4.49E-04	4.49E-04	5.987	-4.92E-04	4.92E-04	5.987	-5.30E-04	5.30E-04
5.988	-3.33E-04	3.33E-04	5.988	-3.96E-04	3.96E-04	5.988	-4.49E-04	4.49E-04	5.988	-4.92E-04	4.92E-04	5.988	-5.30E-04	5.30E-04
5.989	-3.33E-04	3.33E-04	5.989	-3.96E-04	3.96E-04	5.989	-4.48E-04	4.48E-04	5.989	-4.92E-04	4.92E-04	5.989	-5.30E-04	5.30E-04
5.99	-3.32E-04	3.32E-04	5.99	-3.96E-04	3.96E-04	5.99	-4.48E-04	4.48E-04	5.99	-4.92E-04	4.92E-04	5.99	-5.29E-04	5.29E-04
5.991	-3.32E-04	3.32E-04	5.991	-3.96E-04	3.96E-04	5.991	-4.48E-04	4.48E-04	5.991	-4.92E-04	4.92E-04	5.991	-5.29E-04	5.29E-04
5.992	-3.32E-04	3.32E-04	5.992	-3.96E-04	3.96E-04	5.992	-4.48E-04	4.48E-04	5.992	-4.92E-04	4.92E-04	5.992	-5.29E-04	5.29E-04
5.993	-3.32E-04	3.32E-04	5.993	-3.96E-04	3.96E-04	5.993	-4.48E-04	4.48E-04	5.993	-4.91E-04	4.91E-04	5.993	-5.29E-04	5.29E-04
5.994	-3.32E-04	3.32E-04	5.994	-3.96E-04	3.96E-04	5.994	-4.48E-04	4.48E-04	5.994	-4.91E-04	4.91E-04	5.994	-5.29E-04	5.29E-04
5.995	-3.32E-04	3.32E-04	5.995	-3.96E-04	3.96E-04	5.995	-4.48E-04	4.48E-04	5.995	-4.91E-04	4.91E-04	5.995	-5.28E-04	5.28E-04
5.996	-3.32E-04	3.32E-04	5.996	-3.95E-04	3.95E-04	5.996	-4.47E-04	4.47E-04	5.996	-4.91E-04	4.91E-04	5.996	-5.28E-04	5.28E-04
5.997	-3.32E-04	3.32E-04	5.997	-3.95E-04	3.95E-04	5.997	-4.47E-04	4.47E-04	5.997	-4.91E-04	4.91E-04	5.997	-5.28E-04	5.28E-04
5.998	-3.32E-04	3.32E-04	5.998	-3.95E-04	3.95E-04	5.998	-4.47E-04	4.47E-04	5.998	-4.91E-04	4.91E-04	5.998	-5.28E-04	5.28E-04
5.999	-3.31E-04	3.31E-04	5.999	-3.95E-04	3.95E-04	5.999	-4.47E-04	4.47E-04	5.999	-4.90E-04	4.90E-04	5.999	-5.28E-04	5.28E-04
6	-3.31E-04	3.31E-04	6	-3.95E-04	3.95E-04	6	-4.47E-04	4.47E-04	6	-4.90E-04	4.90E-04	6	-5.28E-04	5.28E-04

b=1		b=0.75		b=0.5		b=0.25		b=0	
distance from roadway center/m	u_r	distance from roadway center/m	u_r	distance from roadway center/m	u_r	distance from roadway center/m	u_r	distance from roadway center/m	u_r
3	0.00497	3	0.00638	3	0.00766	3	0.00885	3	0.00995
3.001	0.00497	3.001	0.00637	3.001	0.00766	3.001	0.00885	3.001	0.00995
3.002	0.00497	3.002	0.00637	3.002	0.00765	3.002	0.00884	3.002	0.00994
3.003	0.00496	3.003	0.00636	3.003	0.00765	3.003	0.00884	3.003	0.00994
3.004	0.00496	3.004	0.00636	3.004	0.00764	3.004	0.00883	3.004	0.00993
3.005	0.00496	3.005	0.00636	3.005	0.00764	3.005	0.00882	3.005	0.00992
3.006	0.00496	3.006	0.00635	3.006	0.00764	3.006	0.00882	3.006	0.00992
3.007	0.00495	3.007	0.00635	3.007	0.00763	3.007	0.00881	3.007	0.00991
3.008	0.00495	3.008	0.00635	3.008	0.00763	3.008	0.00881	3.008	0.0099
3.009	0.00495	3.009	0.00634	3.009	0.00762	3.009	0.0088	3.009	0.0099
3.01	0.00495	3.01	0.00634	3.01	0.00762	3.01	0.0088	3.01	0.00989
3.011	0.00494	3.011	0.00633	3.011	0.00761	3.011	0.00879	3.011	0.00988
3.012	0.00494	3.012	0.00633	3.012	0.00761	3.012	0.00879	3.012	0.00988
3.013	0.00494	3.013	0.00633	3.013	0.0076	3.013	0.00878	3.013	0.00987
3.014	0.00493	3.014	0.00632	3.014	0.0076	3.014	0.00877	3.014	0.00987
3.015	0.00493	3.015	0.00632	3.015	0.00759	3.015	0.00877	3.015	0.00986
3.016	0.00493	3.016	0.00632	3.016	0.00759	3.016	0.00876	3.016	0.00985
3.017	0.00493	3.017	0.00631	3.017	0.00758	3.017	0.00876	3.017	0.00985
3.018	0.00492	3.018	0.00631	3.018	0.00758	3.018	0.00875	3.018	0.00984
3.019	0.00492	3.019	0.0063	3.019	0.00757	3.019	0.00875	3.019	0.00983
3.02	0.00492	3.02	0.0063	3.02	0.00757	3.02	0.00874	3.02	0.00983
3.021	0.00491	3.021	0.0063	3.021	0.00757	3.021	0.00874	3.021	0.00982
3.022	0.00491	3.022	0.00629	3.022	0.00756	3.022	0.00873	3.022	0.00981
3.023	0.00491	3.023	0.00629	3.023	0.00756	3.023	0.00873	3.023	0.00981
3.024	0.00491	3.024	0.00629	3.024	0.00755	3.024	0.00872	3.024	0.0098
3.025	0.0049	3.025	0.00628	3.025	0.00755	3.025	0.00871	3.025	0.0098
3.026	0.0049	3.026	0.00628	3.026	0.00754	3.026	0.00871	3.026	0.00979
3.027	0.0049	3.027	0.00627	3.027	0.00754	3.027	0.0087	3.027	0.00978
3.028	0.0049	3.028	0.00627	3.028	0.00753	3.028	0.0087	3.028	0.00978
3.029	0.00489	3.029	0.00627	3.029	0.00753	3.029	0.00869	3.029	0.00977
3.03	0.00489	3.03	0.00626	3.03	0.00752	3.03	0.00869	3.03	0.00976
3.031	0.00489	3.031	0.00626	3.031	0.00752	3.031	0.00868	3.031	0.00976
3.032	0.00488	3.032	0.00626	3.032	0.00752	3.032	0.00868	3.032	0.00975
3.033	0.00488	3.033	0.00625	3.033	0.00751	3.033	0.00867	3.033	0.00975
3.034	0.00488	3.034	0.00625	3.034	0.00751	3.034	0.00867	3.034	0.00974
3.035	0.00488	3.035	0.00625	3.035	0.0075	3.035	0.00866	3.035	0.00973
3.036	0.00487	3.036	0.00624	3.036	0.0075	3.036	0.00865	3.036	0.00973
3.037	0.00487	3.037	0.00624	3.037	0.00749	3.037	0.00865	3.037	0.00972
3.038	0.00487	3.038	0.00623	3.038	0.00749	3.038	0.00864	3.038	0.00972
3.039	0.00487	3.039	0.00623	3.039	0.00748	3.039	0.00864	3.039	0.00971
3.04	0.00486	3.04	0.00623	3.04	0.00748	3.04	0.00863	3.04	0.0097
3.041	0.00486	3.041	0.00622	3.041	0.00747	3.041	0.00863	3.041	0.0097
3.042	0.00486	3.042	0.00622	3.042	0.00747	3.042	0.00862	3.042	0.00969
3.043	0.00485	3.043	0.00622	3.043	0.00747	3.043	0.00862	3.043	0.00968
3.044	0.00485	3.044	0.00621	3.044	0.00746	3.044	0.00861	3.044	0.00968
3.045	0.00485	3.045	0.00621	3.045	0.00746	3.045	0.00861	3.045	0.00967
3.046	0.00485	3.046	0.00621	3.046	0.00745	3.046	0.0086	3.046	0.00967
3.047	0.00484	3.047	0.0062	3.047	0.00745	3.047	0.0086	3.047	0.00966
3.048	0.00484	3.048	0.0062	3.048	0.00744	3.048	0.00859	3.048	0.00965
3.049	0.00484	3.049	0.00619	3.049	0.00744	3.049	0.00859	3.049	0.00965
3.05	0.00484	3.05	0.00619	3.05	0.00743	3.05	0.00858	3.05	0.00964
3.051	0.00483	3.051	0.00619	3.051	0.00743	3.051	0.00857	3.051	0.00964
3.052	0.00483	3.052	0.00618	3.052	0.00743	3.052	0.00857	3.052	0.00963
3.053	0.00483	3.053	0.00618	3.053	0.00742	3.053	0.00856	3.053	0.00962
3.054	0.00482	3.054	0.00618	3.054	0.00742	3.054	0.00856	3.054	0.00962
3.055	0.00482	3.055	0.00617	3.055	0.00741	3.055	0.00855	3.055	0.00961
3.056	0.00482	3.056	0.00617	3.056	0.00741	3.056	0.00855	3.056	0.00961
3.057	0.00482	3.057	0.00617	3.057	0.0074	3.057	0.00854	3.057	0.0096
3.058	0.00481	3.058	0.00616	3.058	0.0074	3.058	0.00854	3.058	0.00959
3.059	0.00481	3.059	0.00616	3.059	0.00739	3.059	0.00853	3.059	0.00959
3.06	0.00481	3.06	0.00616	3.06	0.00739	3.06	0.00853	3.06	0.00958
3.061	0.00481	3.061	0.00615	3.061	0.00738	3.061	0.00852	3.061	0.00957
3.062	0.0048	3.062	0.00615	3.062	0.00738	3.062	0.00852	3.062	0.00957
3.063	0.0048	3.063	0.00614	3.063	0.00738	3.063	0.00851	3.063	0.00956
3.064	0.0048	3.064	0.00614	3.064	0.00737	3.064	0.00851	3.064	0.00956
3.065	0.0048	3.065	0.00614	3.065	0.00737	3.065	0.0085	3.065	0.00955
3.066	0.00479	3.066	0.00613	3.066	0.00736	3.066	0.0085	3.066	0.00954
3.067	0.00479	3.067	0.00613	3.067	0.00736	3.067	0.00849	3.067	0.00954
3.068	0.00479	3.068	0.00613	3.068	0.00735	3.068	0.00848	3.068	0.00953
3.069	0.00478	3.069	0.00612	3.069	0.00735	3.069	0.00848	3.069	0.00953
3.07	0.00478	3.07	0.00612	3.07	0.00735	3.07	0.00847	3.07	0.00952
3.071	0.00478	3.071	0.00612	3.071	0.00734	3.071	0.00847	3.071	0.00951
3.072	0.00478	3.072	0.00611	3.072	0.00734	3.072	0.00846	3.072	0.00951
3.073	0.00477	3.073	0.00611	3.073	0.00733	3.073	0.00846	3.073	0.0095
3.074	0.00477	3.074	0.00611	3.074	0.00733	3.074	0.00845	3.074	0.0095
3.075	0.00477	3.075	0.0061	3.075	0.00732	3.075	0.00845	3.075	0.00949
3.076	0.00477	3.076	0.0061	3.076	0.00732	3.076	0.00844	3.076	0.00948
3.077	0.00476	3.077	0.00609	3.077	0.00731	3.077	0.00844	3.077	0.00948
3.078	0.00476	3.078	0.00609	3.078	0.00731	3.078	0.00843	3.078	0.00947
3.079	0.00476	3.079	0.00609	3.079	0.00731	3.079	0.00843	3.079	0.00947
3.08	0.00476	3.08	0.00608	3.08	0.0073	3.08	0.00842	3.08	0.00946
3.081	0.00475	3.081	0.00608	3.081	0.0073	3.081	0.00842	3.081	0.00946
3.082	0.00475	3.082	0.00608	3.082	0.00729	3.082	0.00841	3.082	0.00945
3.083	0.00475	3.083	0.00607	3.083	0.00729	3.083	0.00841	3.083	0.00944
3.084	0.00475	3.084	0.00607	3.084	0.00728	3.084	0.0084	3.084	0.00944
3.085	0.00474	3.085	0.00607	3.085	0.00728	3.085	0.0084	3.085	0.00943
3.086	0.00474	3.086	0.00606	3.086	0.00728	3.086	0.00839	3.086	0.00943
3.087	0.00474	3.087	0.00606	3.087	0.00727	3.087	0.00839	3.087	0.00942
3.088	0.00473	3.088	0.00606	3.088	0.00727	3.088	0.00838	3.088	0.00941
3.089	0.00473	3.089	0.00605	3.089	0.00726	3.089	0.00838	3.089	0.00941
3.09	0.00473	3.09	0.00605	3.09	0.00726	3.09	0.00837	3.09	0.0094
3.091	0.00473	3.091	0.00605	3.091	0.00725	3.091	0.00837	3.091	0.0094
3.092	0.00472	3.092	0.00604	3.092	0.00725	3.092	0.00836	3.092	0.00939
3.093	0.00472	3.093	0.00604	3.093	0.00725	3.093	0.00836	3.093	0.00938
3.094	0.00472	3.094	0.00604	3.094	0.00724	3.094	0.00835	3.094	0.00938
3.095	0.00472	3.095	0.00603	3.095	0.00724	3.095	0.00835	3.095	0.00937
3.096	0.00471	3.096	0.00603	3.096	0.00723	3.096	0.00834	3.096	0.00937
3.097	0.00471	3.097	0.00603	3.097	0.00723	3.097	0.00834	3.097	0.00936
3.098	0.00471	3.098	0.00602	3.098	0.00722	3.098	0.00833	3.098	0.00936
3.099	0.00471	3.099	0.00602	3.099	0.00722	3.099	0.00833	3.099	0.00935
3.1	0.0047	3.1	0.00601	3.1	0.00722	3.1	0.00832	3.1	0.00934
3.101	0.0047	3.101	0.00601	3.101	0.00721	3.101	0.00832	3.101	0.00934
3.102	0.0047	3.102	0.00601	3.102	0.00721	3.102	0.00831	3.102	0.00933
3.103	0.0047	3.103	0.006	3.103	0.0072	3.103	0.00831	3.103	0.00933
3.104	0.00469	3.104	0.006	3.104	0.0072	3.104	0.0083	3.104	0.00932
3.105	0.00469	3.105	0.006	3.105	0.00719	3.105	0.00829	3.105	0.00931
3.106	0.00469	3.106	0.00599	3.106	0.00719	3.106	0.00829	3.106	0.00931
3.107	0.00469	3.107	0.00599	3.107	0.00719	3.107	0.00828	3.107	0.0093
3.108	0.00468	3.108	0.00599	3.108	0.00718	3.108	0.00828	3.108	0.0093
3.109	0.00468	3.109	0.00598	3.109	0.00718	3.109	0.00827	3.109	0.00929
3.11	0.00468	3.11	0.00598	3.11	0.00717	3.11	0.00827	3.11	0.00929
3.111	0.00468	3.111	0.00598	3.111	0.00717	3.111	0.00826	3.111	0.00928
3.112	0.00467	3.112	0.00597	3.112	0.00716	3.112	0.00826	3.112	0.00927
3.113	0.00467	3.113	0.00597	3.113	0.00716	3.113	0.00825	3.113	0.00927
3.114	0.00467	3.114	0.00597	3.114	0.00716	3.114	0.00825	3.114	0.00926
3.115	0.00467	3.115	0.00596	3.115	0.00715	3.115	0.00824	3.115	0.00926
3.116	0.00466	3.116	0.00596	3.116	0.00715	3.116	0.00824	3.116	0.00925
3.117	0.00466	3.117	0.00596	3.117	0.00714	3.117	0.00823	3.117	0.00925
3.118	0.00466	3.118	0.00595	3.118	0.00714	3.118	0.00823	3.118	0.00924
3.119	0.00466	3.119	0.00595	3.119	0.00713	3.119	0.00822	3.119	0.00923
3.12	0.00465	3.12	0.00595	3.12	0.00713	3.12	0.00822	3.12	0.00923
3.121	0.00465	3.121	0.00594	3.121	0.00713	3.121	0.00821	3.121	0.00922
3.122	0.00465	3.122	0.00594	3.122	0.00712	3.122	0.00821	3.122	0.00922
3.123	0.00465	3.123	0.00594	3.123	0.00712	3.123	0.0082	3.123	0.00921
3.124	0.00464	3.124	0.00593	3.124	0.00711	3.124	0.0082	3.124	0.00921
3.125	0.00464	3.125	0.00593	3.125	0.00711	3.125	0.00819	3.125	0.0092
3.126	0.00464	3.126	0.00593	3.126	0.00711	3.126	0.00819	3.126	0.00919
3.127	0.00464	3.127	0.00592	3.127	0.0071	3.127	0.00819	3.127	0.00919
3.128	0.00463	3.128	0.00592	3.128	0.0071	3.128	0.00818	3.128	0.00918
3.129	0.00463	3.129	0.00592	3.129	0.00709	3.129	0.00818	3.129	0.00918
3.13	0.00463	3.13	0.00591	3.13	0.00709	3.13	0.00817	3.13	0.00917
3.131	0.00463	3.131	0.00591	3.131	0.00708	3.131	0.00817	3.131	0.00917
3.132	0.00462	3.132	0.00591	3.132	0.00708	3.132	0.00816	3.132	0.00916

3.133	0.00462	3.133	0.0059	3.133	0.00708	3.133	0.00816	3.133	0.00915
3.134	0.00462	3.134	0.0059	3.134	0.00707	3.134	0.00815	3.134	0.00915
3.135	0.00462	3.135	0.0059	3.135	0.00707	3.135	0.00815	3.135	0.00914
3.136	0.00461	3.136	0.00589	3.136	0.00706	3.136	0.00814	3.136	0.00914
3.137	0.00461	3.137	0.00589	3.137	0.00706	3.137	0.00814	3.137	0.00913
3.138	0.00461	3.138	0.00589	3.138	0.00706	3.138	0.00813	3.138	0.00913
3.139	0.00461	3.139	0.00588	3.139	0.00705	3.139	0.00813	3.139	0.00912
3.14	0.0046	3.14	0.00588	3.14	0.00705	3.14	0.00812	3.14	0.00912
3.141	0.0046	3.141	0.00588	3.141	0.00704	3.141	0.00812	3.141	0.00911
3.142	0.0046	3.142	0.00587	3.142	0.00704	3.142	0.00811	3.142	0.0091
3.143	0.0046	3.143	0.00587	3.143	0.00703	3.143	0.00811	3.143	0.0091
3.144	0.00459	3.144	0.00587	3.144	0.00703	3.144	0.0081	3.144	0.00909
3.145	0.00459	3.145	0.00586	3.145	0.00703	3.145	0.0081	3.145	0.00909
3.146	0.00459	3.146	0.00586	3.146	0.00702	3.146	0.00809	3.146	0.00908
3.147	0.00459	3.147	0.00586	3.147	0.00702	3.147	0.00809	3.147	0.00908
3.148	0.00458	3.148	0.00585	3.148	0.00701	3.148	0.00808	3.148	0.00907
3.149	0.00458	3.149	0.00585	3.149	0.00701	3.149	0.00808	3.149	0.00906
3.15	0.00458	3.15	0.00585	3.15	0.00701	3.15	0.00807	3.15	0.00906
3.151	0.00458	3.151	0.00584	3.151	0.007	3.151	0.00807	3.151	0.00905
3.152	0.00457	3.152	0.00584	3.152	0.007	3.152	0.00806	3.152	0.00905
3.153	0.00457	3.153	0.00584	3.153	0.00699	3.153	0.00806	3.153	0.00904
3.154	0.00457	3.154	0.00583	3.154	0.00699	3.154	0.00805	3.154	0.00904
3.155	0.00457	3.155	0.00583	3.155	0.00699	3.155	0.00805	3.155	0.00903
3.156	0.00456	3.156	0.00583	3.156	0.00698	3.156	0.00804	3.156	0.00903
3.157	0.00456	3.157	0.00582	3.157	0.00698	3.157	0.00804	3.157	0.00902
3.158	0.00456	3.158	0.00582	3.158	0.00697	3.158	0.00803	3.158	0.00901
3.159	0.00456	3.159	0.00582	3.159	0.00697	3.159	0.00803	3.159	0.00901
3.16	0.00455	3.16	0.00581	3.16	0.00697	3.16	0.00802	3.16	0.009
3.161	0.00455	3.161	0.00581	3.161	0.00696	3.161	0.00802	3.161	0.009
3.162	0.00455	3.162	0.00581	3.162	0.00696	3.162	0.00801	3.162	0.00899
3.163	0.00455	3.163	0.0058	3.163	0.00695	3.163	0.00801	3.163	0.00899
3.164	0.00454	3.164	0.0058	3.164	0.00695	3.164	0.00801	3.164	0.00898
3.165	0.00454	3.165	0.0058	3.165	0.00695	3.165	0.008	3.165	0.00898
3.166	0.00454	3.166	0.00579	3.166	0.00694	3.166	0.008	3.166	0.00897
3.167	0.00454	3.167	0.00579	3.167	0.00694	3.167	0.00799	3.167	0.00897
3.168	0.00453	3.168	0.00579	3.168	0.00693	3.168	0.00799	3.168	0.00896
3.169	0.00453	3.169	0.00578	3.169	0.00693	3.169	0.00798	3.169	0.00895
3.17	0.00453	3.17	0.00578	3.17	0.00692	3.17	0.00798	3.17	0.00895
3.171	0.00453	3.171	0.00578	3.171	0.00692	3.171	0.00797	3.171	0.00894
3.172	0.00452	3.172	0.00577	3.172	0.00692	3.172	0.00797	3.172	0.00894
3.173	0.00452	3.173	0.00577	3.173	0.00691	3.173	0.00796	3.173	0.00893
3.174	0.00452	3.174	0.00577	3.174	0.00691	3.174	0.00796	3.174	0.00893
3.175	0.00452	3.175	0.00576	3.175	0.0069	3.175	0.00795	3.175	0.00892
3.176	0.00451	3.176	0.00576	3.176	0.0069	3.176	0.00795	3.176	0.00892
3.177	0.00451	3.177	0.00576	3.177	0.0069	3.177	0.00794	3.177	0.00891
3.178	0.00451	3.178	0.00576	3.178	0.00689	3.178	0.00794	3.178	0.00891
3.179	0.00451	3.179	0.00575	3.179	0.00689	3.179	0.00793	3.179	0.0089
3.18	0.0045	3.18	0.00575	3.18	0.00689	3.18	0.00793	3.18	0.00889
3.181	0.0045	3.181	0.00575	3.181	0.00688	3.181	0.00792	3.181	0.00889
3.182	0.0045	3.182	0.00574	3.182	0.00688	3.182	0.00792	3.182	0.00888
3.183	0.0045	3.183	0.00574	3.183	0.00687	3.183	0.00792	3.183	0.00888
3.184	0.0045	3.184	0.00574	3.184	0.00687	3.184	0.00791	3.184	0.00887
3.185	0.00449	3.185	0.00573	3.185	0.00687	3.185	0.00791	3.185	0.00887
3.186	0.00449	3.186	0.00573	3.186	0.00686	3.186	0.0079	3.186	0.00886
3.187	0.00449	3.187	0.00573	3.187	0.00686	3.187	0.0079	3.187	0.00886
3.188	0.00449	3.188	0.00572	3.188	0.00685	3.188	0.00789	3.188	0.00885
3.189	0.00448	3.189	0.00572	3.189	0.00685	3.189	0.00789	3.189	0.00885
3.19	0.00448	3.19	0.00572	3.19	0.00685	3.19	0.00788	3.19	0.00884
3.191	0.00448	3.191	0.00571	3.191	0.00684	3.191	0.00788	3.191	0.00884
3.192	0.00448	3.192	0.00571	3.192	0.00684	3.192	0.00787	3.192	0.00883
3.193	0.00447	3.193	0.00571	3.193	0.00683	3.193	0.00787	3.193	0.00883
3.194	0.00447	3.194	0.0057	3.194	0.00683	3.194	0.00786	3.194	0.00882
3.195	0.00447	3.195	0.0057	3.195	0.00683	3.195	0.00786	3.195	0.00881
3.196	0.00447	3.196	0.0057	3.196	0.00682	3.196	0.00785	3.196	0.00881
3.197	0.00446	3.197	0.00569	3.197	0.00682	3.197	0.00785	3.197	0.0088
3.198	0.00446	3.198	0.00569	3.198	0.00681	3.198	0.00785	3.198	0.0088
3.199	0.00446	3.199	0.00569	3.199	0.00681	3.199	0.00784	3.199	0.00879
3.2	0.00446	3.2	0.00568	3.2	0.00681	3.2	0.00784	3.2	0.00879
3.201	0.00445	3.201	0.00568	3.201	0.0068	3.201	0.00783	3.201	0.00878
3.202	0.00445	3.202	0.00568	3.202	0.0068	3.202	0.00783	3.202	0.00878
3.203	0.00445	3.203	0.00568	3.203	0.00679	3.203	0.00782	3.203	0.00877
3.204	0.00445	3.204	0.00567	3.204	0.00679	3.204	0.00782	3.204	0.00877
3.205	0.00445	3.205	0.00567	3.205	0.00679	3.205	0.00781	3.205	0.00876
3.206	0.00444	3.206	0.00567	3.206	0.00678	3.206	0.00781	3.206	0.00876
3.207	0.00444	3.207	0.00566	3.207	0.00678	3.207	0.0078	3.207	0.00875
3.208	0.00444	3.208	0.00566	3.208	0.00677	3.208	0.0078	3.208	0.00875
3.209	0.00444	3.209	0.00566	3.209	0.00677	3.209	0.00779	3.209	0.00874
3.21	0.00443	3.21	0.00565	3.21	0.00677	3.21	0.00779	3.21	0.00874
3.211	0.00443	3.211	0.00565	3.211	0.00676	3.211	0.00779	3.211	0.00873
3.212	0.00443	3.212	0.00565	3.212	0.00676	3.212	0.00778	3.212	0.00872
3.213	0.00443	3.213	0.00564	3.213	0.00676	3.213	0.00778	3.213	0.00872
3.214	0.00442	3.214	0.00564	3.214	0.00675	3.214	0.00777	3.214	0.00871
3.215	0.00442	3.215	0.00564	3.215	0.00675	3.215	0.00777	3.215	0.00871
3.216	0.00442	3.216	0.00563	3.216	0.00674	3.216	0.00776	3.216	0.0087
3.217	0.00442	3.217	0.00563	3.217	0.00674	3.217	0.00776	3.217	0.0087
3.218	0.00441	3.218	0.00563	3.218	0.00674	3.218	0.00775	3.218	0.00869
3.219	0.00441	3.219	0.00563	3.219	0.00673	3.219	0.00775	3.219	0.00869
3.22	0.00441	3.22	0.00562	3.22	0.00673	3.22	0.00774	3.22	0.00868
3.221	0.00441	3.221	0.00562	3.221	0.00672	3.221	0.00774	3.221	0.00868
3.222	0.00441	3.222	0.00562	3.222	0.00672	3.222	0.00774	3.222	0.00867
3.223	0.0044	3.223	0.00561	3.223	0.00672	3.223	0.00773	3.223	0.00867
3.224	0.0044	3.224	0.00561	3.224	0.00671	3.224	0.00773	3.224	0.00866
3.225	0.0044	3.225	0.00561	3.225	0.00671	3.225	0.00772	3.225	0.00866
3.226	0.0044	3.226	0.0056	3.226	0.00671	3.226	0.00772	3.226	0.00865
3.227	0.00439	3.227	0.0056	3.227	0.0067	3.227	0.00771	3.227	0.00865
3.228	0.00439	3.228	0.0056	3.228	0.0067	3.228	0.00771	3.228	0.00864
3.229	0.00439	3.229	0.00559	3.229	0.00669	3.229	0.0077	3.229	0.00864
3.23	0.00439	3.23	0.00559	3.23	0.00669	3.23	0.0077	3.23	0.00863
3.231	0.00438	3.231	0.00559	3.231	0.00669	3.231	0.00769	3.231	0.00863
3.232	0.00438	3.232	0.00559	3.232	0.00668	3.232	0.00769	3.232	0.00862
3.233	0.00438	3.233	0.00558	3.233	0.00668	3.233	0.00769	3.233	0.00862
3.234	0.00438	3.234	0.00558	3.234	0.00668	3.234	0.00768	3.234	0.00861
3.235	0.00438	3.235	0.00558	3.235	0.00667	3.235	0.00768	3.235	0.00861
3.236	0.00437	3.236	0.00557	3.236	0.00667	3.236	0.00767	3.236	0.0086
3.237	0.00437	3.237	0.00557	3.237	0.00666	3.237	0.00767	3.237	0.00859
3.238	0.00437	3.238	0.00557	3.238	0.00666	3.238	0.00766	3.238	0.00859
3.239	0.00437	3.239	0.00556	3.239	0.00666	3.239	0.00766	3.239	0.00858
3.24	0.00436	3.24	0.00556	3.24	0.00665	3.24	0.00765	3.24	0.00858
3.241	0.00436	3.241	0.00556	3.241	0.00665	3.241	0.00765	3.241	0.00857
3.242	0.00436	3.242	0.00555	3.242	0.00664	3.242	0.00765	3.242	0.00857
3.243	0.00436	3.243	0.00555	3.243	0.00664	3.243	0.00764	3.243	0.00856
3.244	0.00435	3.244	0.00555	3.244	0.00664	3.244	0.00764	3.244	0.00856
3.245	0.00435	3.245	0.00555	3.245	0.00663	3.245	0.00763	3.245	0.00855
3.246	0.00435	3.246	0.00554	3.246	0.00663	3.246	0.00763	3.246	0.00855
3.247	0.00435	3.247	0.00554	3.247	0.00663	3.247	0.00762	3.247	0.00854
3.248	0.00435	3.248	0.00554	3.248	0.00662	3.248	0.00762	3.248	0.00854
3.249	0.00434	3.249	0.00553	3.249	0.00662	3.249	0.00761	3.249	0.00853
3.25	0.00434	3.25	0.00553	3.25	0.00661	3.25	0.00761	3.25	0.00853
3.251	0.00434	3.251	0.00553	3.251	0.00661	3.251	0.00761	3.251	0.00852
3.252	0.00434	3.252	0.00552	3.252	0.00661	3.252	0.0076	3.252	0.00852
3.253	0.00433	3.253	0.00552	3.253	0.0066	3.253	0.0076	3.253	0.00851
3.254	0.00433	3.254	0.00552	3.254	0.0066	3.254	0.00759	3.254	0.00851
3.255	0.00433	3.255	0.00552	3.255	0.0066	3.255	0.00759	3.255	0.0085
3.256	0.00433	3.256	0.00551	3.256	0.00659	3.256	0.00758	3.256	0.0085
3.257	0.00433	3.257	0.00551	3.257	0.00659	3.257	0.00758	3.257	0.00849
3.258	0.00432	3.258	0.00551	3.258	0.00659	3.258	0.00757	3.258	0.00849
3.259	0.00432	3.259	0.0055	3.259	0.00658	3.259	0.00757	3.259	0.00848
3.26	0.00432	3.26	0.0055	3.26	0.00658	3.26	0.00757	3.26	0.00848
3.261	0.00432	3.261	0.0055	3.261	0.00657	3.261	0.00756	3.261	0.00847
3.262	0.00431	3.262	0.00549	3.262	0.00657	3.262	0.00756	3.262	0.00847
3.263	0.00431	3.263	0.00549	3.263	0.00657	3.263	0.00755	3.263	0.00846
3.264	0.00431	3.264	0.00549	3.264	0.00656	3.264	0.00755	3.264	0.00846
3.265	0.00431	3.265	0.00549	3.265	0.00656	3.265	0.00754	3.265	0.00845
3.266	0.00431	3.266	0.00548	3.266	0.00656	3.266	0.00754	3.266	0.00845
3.267	0.0043	3.267	0.00548	3.267	0.00655	3.267	0.00754	3.267	0.00844

3.268	0.0043	3.268	0.00548	3.268	0.00655	3.268	0.00753	3.268	0.00844
3.269	0.0043	3.269	0.00547	3.269	0.00654	3.269	0.00753	3.269	0.00843
3.27	0.0043	3.27	0.00547	3.27	0.00654	3.27	0.00752	3.27	0.00843
3.271	0.00429	3.271	0.00547	3.271	0.00654	3.271	0.00752	3.271	0.00842
3.272	0.00429	3.272	0.00546	3.272	0.00653	3.272	0.00751	3.272	0.00842
3.273	0.00429	3.273	0.00546	3.273	0.00653	3.273	0.00751	3.273	0.00841
3.274	0.00429	3.274	0.00546	3.274	0.00653	3.274	0.0075	3.274	0.00841
3.275	0.00429	3.275	0.00546	3.275	0.00652	3.275	0.0075	3.275	0.0084
3.276	0.00428	3.276	0.00545	3.276	0.00652	3.276	0.0075	3.276	0.0084
3.277	0.00428	3.277	0.00545	3.277	0.00652	3.277	0.00749	3.277	0.00839
3.278	0.00428	3.278	0.00545	3.278	0.00651	3.278	0.00749	3.278	0.00839
3.279	0.00428	3.279	0.00544	3.279	0.00651	3.279	0.00748	3.279	0.00838
3.28	0.00427	3.28	0.00544	3.28	0.0065	3.28	0.00748	3.28	0.00838
3.281	0.00427	3.281	0.00544	3.281	0.0065	3.281	0.00747	3.281	0.00837
3.282	0.00427	3.282	0.00544	3.282	0.0065	3.282	0.00747	3.282	0.00837
3.283	0.00427	3.283	0.00543	3.283	0.00649	3.283	0.00747	3.283	0.00836
3.284	0.00427	3.284	0.00543	3.284	0.00649	3.284	0.00746	3.284	0.00836
3.285	0.00426	3.285	0.00543	3.285	0.00649	3.285	0.00746	3.285	0.00835
3.286	0.00426	3.286	0.00542	3.286	0.00648	3.286	0.00745	3.286	0.00835
3.287	0.00426	3.287	0.00542	3.287	0.00648	3.287	0.00745	3.287	0.00834
3.288	0.00426	3.288	0.00542	3.288	0.00648	3.288	0.00744	3.288	0.00834
3.289	0.00425	3.289	0.00541	3.289	0.00647	3.289	0.00744	3.289	0.00833
3.29	0.00425	3.29	0.00541	3.29	0.00647	3.29	0.00744	3.29	0.00833
3.291	0.00425	3.291	0.00541	3.291	0.00646	3.291	0.00743	3.291	0.00832
3.292	0.00425	3.292	0.00541	3.292	0.00646	3.292	0.00743	3.292	0.00832
3.293	0.00425	3.293	0.0054	3.293	0.00646	3.293	0.00742	3.293	0.00831
3.294	0.00424	3.294	0.0054	3.294	0.00645	3.294	0.00742	3.294	0.00831
3.295	0.00424	3.295	0.0054	3.295	0.00645	3.295	0.00741	3.295	0.00831
3.296	0.00424	3.296	0.00539	3.296	0.00645	3.296	0.00741	3.296	0.0083
3.297	0.00424	3.297	0.00539	3.297	0.00644	3.297	0.00741	3.297	0.0083
3.298	0.00423	3.298	0.00539	3.298	0.00644	3.298	0.0074	3.298	0.00829
3.299	0.00423	3.299	0.00539	3.299	0.00644	3.299	0.0074	3.299	0.00829
3.3	0.00423	3.3	0.00538	3.3	0.00643	3.3	0.00739	3.3	0.00828
3.301	0.00423	3.301	0.00538	3.301	0.00643	3.301	0.00739	3.301	0.00828
3.302	0.00423	3.302	0.00538	3.302	0.00642	3.302	0.00738	3.302	0.00827
3.303	0.00422	3.303	0.00537	3.303	0.00642	3.303	0.00738	3.303	0.00827
3.304	0.00422	3.304	0.00537	3.304	0.00642	3.304	0.00738	3.304	0.00826
3.305	0.00422	3.305	0.00537	3.305	0.00641	3.305	0.00737	3.305	0.00826
3.306	0.00422	3.306	0.00537	3.306	0.00641	3.306	0.00737	3.306	0.00825
3.307	0.00421	3.307	0.00536	3.307	0.00641	3.307	0.00736	3.307	0.00825
3.308	0.00421	3.308	0.00536	3.308	0.0064	3.308	0.00736	3.308	0.00824
3.309	0.00421	3.309	0.00536	3.309	0.0064	3.309	0.00736	3.309	0.00824
3.31	0.00421	3.31	0.00535	3.31	0.0064	3.31	0.00735	3.31	0.00823
3.311	0.00421	3.311	0.00535	3.311	0.00639	3.311	0.00735	3.311	0.00823
3.312	0.0042	3.312	0.00535	3.312	0.00639	3.312	0.00734	3.312	0.00822
3.313	0.0042	3.313	0.00535	3.313	0.00639	3.313	0.00734	3.313	0.00822
3.314	0.0042	3.314	0.00534	3.314	0.00638	3.314	0.00733	3.314	0.00821
3.315	0.0042	3.315	0.00534	3.315	0.00638	3.315	0.00733	3.315	0.00821
3.316	0.0042	3.316	0.00534	3.316	0.00637	3.316	0.00733	3.316	0.0082
3.317	0.00419	3.317	0.00533	3.317	0.00637	3.317	0.00732	3.317	0.0082
3.318	0.00419	3.318	0.00533	3.318	0.00637	3.318	0.00732	3.318	0.00819
3.319	0.00419	3.319	0.00533	3.319	0.00636	3.319	0.00731	3.319	0.00819
3.32	0.00419	3.32	0.00533	3.32	0.00636	3.32	0.00731	3.32	0.00818
3.321	0.00418	3.321	0.00532	3.321	0.00636	3.321	0.00731	3.321	0.00818
3.322	0.00418	3.322	0.00532	3.322	0.00635	3.322	0.0073	3.322	0.00818
3.323	0.00418	3.323	0.00532	3.323	0.00635	3.323	0.0073	3.323	0.00817
3.324	0.00418	3.324	0.00531	3.324	0.00635	3.324	0.00729	3.324	0.00817
3.325	0.00418	3.325	0.00531	3.325	0.00634	3.325	0.00729	3.325	0.00816
3.326	0.00417	3.326	0.00531	3.326	0.00634	3.326	0.00728	3.326	0.00816
3.327	0.00417	3.327	0.00531	3.327	0.00634	3.327	0.00728	3.327	0.00815
3.328	0.00417	3.328	0.0053	3.328	0.00633	3.328	0.00728	3.328	0.00815
3.329	0.00417	3.329	0.0053	3.329	0.00633	3.329	0.00727	3.329	0.00814
3.33	0.00417	3.33	0.0053	3.33	0.00633	3.33	0.00727	3.33	0.00814
3.331	0.00416	3.331	0.00529	3.331	0.00632	3.331	0.00726	3.331	0.00813
3.332	0.00416	3.332	0.00529	3.332	0.00632	3.332	0.00726	3.332	0.00813
3.333	0.00416	3.333	0.00529	3.333	0.00632	3.333	0.00726	3.333	0.00812
3.334	0.00416	3.334	0.00529	3.334	0.00631	3.334	0.00725	3.334	0.00812
3.335	0.00415	3.335	0.00528	3.335	0.00631	3.335	0.00725	3.335	0.00811
3.336	0.00415	3.336	0.00528	3.336	0.0063	3.336	0.00724	3.336	0.00811
3.337	0.00415	3.337	0.00528	3.337	0.0063	3.337	0.00724	3.337	0.0081
3.338	0.00415	3.338	0.00527	3.338	0.0063	3.338	0.00724	3.338	0.0081
3.339	0.00415	3.339	0.00527	3.339	0.00629	3.339	0.00723	3.339	0.0081
3.34	0.00414	3.34	0.00527	3.34	0.00629	3.34	0.00723	3.34	0.00809
3.341	0.00414	3.341	0.00527	3.341	0.00629	3.341	0.00722	3.341	0.00809
3.342	0.00414	3.342	0.00526	3.342	0.00628	3.342	0.00722	3.342	0.00808
3.343	0.00414	3.343	0.00526	3.343	0.00628	3.343	0.00721	3.343	0.00808
3.344	0.00414	3.344	0.00526	3.344	0.00628	3.344	0.00721	3.344	0.00807
3.345	0.00413	3.345	0.00525	3.345	0.00627	3.345	0.00721	3.345	0.00807
3.346	0.00413	3.346	0.00525	3.346	0.00627	3.346	0.0072	3.346	0.00806
3.347	0.00413	3.347	0.00525	3.347	0.00627	3.347	0.0072	3.347	0.00806
3.348	0.00413	3.348	0.00525	3.348	0.00626	3.348	0.00719	3.348	0.00805
3.349	0.00413	3.349	0.00524	3.349	0.00626	3.349	0.00719	3.349	0.00805
3.35	0.00412	3.35	0.00524	3.35	0.00626	3.35	0.00719	3.35	0.00804
3.351	0.00412	3.351	0.00524	3.351	0.00625	3.351	0.00718	3.351	0.00804
3.352	0.00412	3.352	0.00524	3.352	0.00625	3.352	0.00718	3.352	0.00803
3.353	0.00412	3.353	0.00523	3.353	0.00625	3.353	0.00717	3.353	0.00803
3.354	0.00412	3.354	0.00523	3.354	0.00624	3.354	0.00717	3.354	0.00803
3.355	0.00411	3.355	0.00523	3.355	0.00624	3.355	0.00717	3.355	0.00802
3.356	0.00411	3.356	0.00522	3.356	0.00624	3.356	0.00716	3.356	0.00802
3.357	0.00411	3.357	0.00522	3.357	0.00623	3.357	0.00716	3.357	0.00801
3.358	0.00411	3.358	0.00522	3.358	0.00623	3.358	0.00715	3.358	0.00801
3.359	0.0041	3.359	0.00522	3.359	0.00623	3.359	0.00715	3.359	0.008
3.36	0.0041	3.36	0.00521	3.36	0.00622	3.36	0.00715	3.36	0.008
3.361	0.0041	3.361	0.00521	3.361	0.00622	3.361	0.00714	3.361	0.00799
3.362	0.0041	3.362	0.00521	3.362	0.00622	3.362	0.00714	3.362	0.00799
3.363	0.0041	3.363	0.0052	3.363	0.00621	3.363	0.00713	3.363	0.00798
3.364	0.00409	3.364	0.0052	3.364	0.00621	3.364	0.00713	3.364	0.00798
3.365	0.00409	3.365	0.0052	3.365	0.00621	3.365	0.00713	3.365	0.00797
3.366	0.00409	3.366	0.0052	3.366	0.0062	3.366	0.00712	3.366	0.00797
3.367	0.00409	3.367	0.00519	3.367	0.0062	3.367	0.00712	3.367	0.00797
3.368	0.00409	3.368	0.00519	3.368	0.0062	3.368	0.00711	3.368	0.00796
3.369	0.00408	3.369	0.00519	3.369	0.00619	3.369	0.00711	3.369	0.00796
3.37	0.00408	3.37	0.00519	3.37	0.00619	3.37	0.00711	3.37	0.00795
3.371	0.00408	3.371	0.00518	3.371	0.00619	3.371	0.0071	3.371	0.00795
3.372	0.00408	3.372	0.00518	3.372	0.00618	3.372	0.0071	3.372	0.00794
3.373	0.00408	3.373	0.00518	3.373	0.00618	3.373	0.00709	3.373	0.00794
3.374	0.00407	3.374	0.00517	3.374	0.00617	3.374	0.00709	3.374	0.00793
3.375	0.00407	3.375	0.00517	3.375	0.00617	3.375	0.00709	3.375	0.00793
3.376	0.00407	3.376	0.00517	3.376	0.00617	3.376	0.00708	3.376	0.00792
3.377	0.00407	3.377	0.00517	3.377	0.00616	3.377	0.00708	3.377	0.00792
3.378	0.00407	3.378	0.00516	3.378	0.00616	3.378	0.00707	3.378	0.00792
3.379	0.00406	3.379	0.00516	3.379	0.00616	3.379	0.00707	3.379	0.00791
3.38	0.00406	3.38	0.00516	3.38	0.00615	3.38	0.00707	3.38	0.00791
3.381	0.00406	3.381	0.00516	3.381	0.00615	3.381	0.00706	3.381	0.0079
3.382	0.00406	3.382	0.00515	3.382	0.00615	3.382	0.00706	3.382	0.0079
3.383	0.00406	3.383	0.00515	3.383	0.00614	3.383	0.00705	3.383	0.00789
3.384	0.00405	3.384	0.00515	3.384	0.00614	3.384	0.00705	3.384	0.00789
3.385	0.00405	3.385	0.00514	3.385	0.00614	3.385	0.00705	3.385	0.00788
3.386	0.00405	3.386	0.00514	3.386	0.00613	3.386	0.00704	3.386	0.00788
3.387	0.00405	3.387	0.00514	3.387	0.00613	3.387	0.00704	3.387	0.00788
3.388	0.00405	3.388	0.00514	3.388	0.00613	3.388	0.00703	3.388	0.00787
3.389	0.00404	3.389	0.00513	3.389	0.00612	3.389	0.00703	3.389	0.00787
3.39	0.00404	3.39	0.00513	3.39	0.00612	3.39	0.00703	3.39	0.00786
3.391	0.00404	3.391	0.00513	3.391	0.00612	3.391	0.00702	3.391	0.00786
3.392	0.00404	3.392	0.00513	3.392	0.00611	3.392	0.00702	3.392	0.00785
3.393	0.00403	3.393	0.00512	3.393	0.00611	3.393	0.00702	3.393	0.00785
3.394	0.00403	3.394	0.00512	3.394	0.00611	3.394	0.00701	3.394	0.00784
3.395	0.00403	3.395	0.00512	3.395	0.0061	3.395	0.00701	3.395	0.00784
3.396	0.00403	3.396	0.00512	3.396	0.0061	3.396	0.007	3.396	0.00783
3.397	0.00403	3.397	0.00511	3.397	0.0061	3.397	0.007	3.397	0.00783
3.398	0.00402	3.398	0.00511	3.398	0.0061	3.398	0.007	3.398	0.00783
3.399	0.00402	3.399	0.00511	3.399	0.00609	3.399	0.00699	3.399	0.00782
3.4	0.00402	3.4	0.0051	3.4	0.00609	3.4	0.00699	3.4	0.00782
3.401	0.00402	3.401	0.0051	3.401	0.00609	3.401	0.00698	3.401	0.00781
3.402	0.00402	3.402	0.0051	3.402	0.00608	3.402	0.00698	3.402	0.00781

3.403	0.00401	3.403	0.0051	3.403	0.00608	3.403	0.00698	3.403	0.0078
3.404	0.00401	3.404	0.00509	3.404	0.00608	3.404	0.00697	3.404	0.0078
3.405	0.00401	3.405	0.00509	3.405	0.00607	3.405	0.00697	3.405	0.00779
3.406	0.00401	3.406	0.00509	3.406	0.00607	3.406	0.00696	3.406	0.00779
3.407	0.00401	3.407	0.00509	3.407	0.00607	3.407	0.00696	3.407	0.00779
3.408	0.004	3.408	0.00508	3.408	0.00606	3.408	0.00696	3.408	0.00778
3.409	0.004	3.409	0.00508	3.409	0.00606	3.409	0.00695	3.409	0.00778
3.41	0.004	3.41	0.00508	3.41	0.00606	3.41	0.00695	3.41	0.00777
3.411	0.004	3.411	0.00508	3.411	0.00605	3.411	0.00695	3.411	0.00777
3.412	0.004	3.412	0.00507	3.412	0.00605	3.412	0.00694	3.412	0.00776
3.413	0.00399	3.413	0.00507	3.413	0.00605	3.413	0.00694	3.413	0.00776
3.414	0.00399	3.414	0.00507	3.414	0.00604	3.414	0.00693	3.414	0.00776
3.415	0.00399	3.415	0.00506	3.415	0.00604	3.415	0.00693	3.415	0.00775
3.416	0.00399	3.416	0.00506	3.416	0.00604	3.416	0.00693	3.416	0.00775
3.417	0.00399	3.417	0.00506	3.417	0.00603	3.417	0.00692	3.417	0.00774
3.418	0.00398	3.418	0.00506	3.418	0.00603	3.418	0.00692	3.418	0.00774
3.419	0.00398	3.419	0.00505	3.419	0.00603	3.419	0.00691	3.419	0.00773
3.42	0.00398	3.42	0.00505	3.42	0.00602	3.42	0.00691	3.42	0.00773
3.421	0.00398	3.421	0.00505	3.421	0.00602	3.421	0.00691	3.421	0.00772
3.422	0.00398	3.422	0.00505	3.422	0.00602	3.422	0.0069	3.422	0.00772
3.423	0.00398	3.423	0.00504	3.423	0.00601	3.423	0.0069	3.423	0.00772
3.424	0.00397	3.424	0.00504	3.424	0.00601	3.424	0.0069	3.424	0.00771
3.425	0.00397	3.425	0.00504	3.425	0.00601	3.425	0.00689	3.425	0.00771
3.426	0.00397	3.426	0.00504	3.426	0.006	3.426	0.00689	3.426	0.0077
3.427	0.00397	3.427	0.00503	3.427	0.006	3.427	0.00688	3.427	0.0077
3.428	0.00397	3.428	0.00503	3.428	0.006	3.428	0.00688	3.428	0.00769
3.429	0.00396	3.429	0.00503	3.429	0.00599	3.429	0.00688	3.429	0.00769
3.43	0.00396	3.43	0.00503	3.43	0.00599	3.43	0.00687	3.43	0.00769
3.431	0.00396	3.431	0.00502	3.431	0.00599	3.431	0.00687	3.431	0.00768
3.432	0.00396	3.432	0.00502	3.432	0.00598	3.432	0.00687	3.432	0.00768
3.433	0.00396	3.433	0.00502	3.433	0.00598	3.433	0.00686	3.433	0.00767
3.434	0.00395	3.434	0.00502	3.434	0.00598	3.434	0.00686	3.434	0.00767
3.435	0.00395	3.435	0.00501	3.435	0.00597	3.435	0.00685	3.435	0.00766
3.436	0.00395	3.436	0.00501	3.436	0.00597	3.436	0.00685	3.436	0.00766
3.437	0.00395	3.437	0.00501	3.437	0.00597	3.437	0.00685	3.437	0.00766
3.438	0.00395	3.438	0.005	3.438	0.00597	3.438	0.00684	3.438	0.00765
3.439	0.00394	3.439	0.005	3.439	0.00596	3.439	0.00684	3.439	0.00765
3.44	0.00394	3.44	0.005	3.44	0.00596	3.44	0.00684	3.44	0.00764
3.441	0.00394	3.441	0.005	3.441	0.00596	3.441	0.00683	3.441	0.00764
3.442	0.00394	3.442	0.00499	3.442	0.00595	3.442	0.00683	3.442	0.00763
3.443	0.00394	3.443	0.00499	3.443	0.00595	3.443	0.00682	3.443	0.00763
3.444	0.00393	3.444	0.00499	3.444	0.00595	3.444	0.00682	3.444	0.00763
3.445	0.00393	3.445	0.00499	3.445	0.00594	3.445	0.00682	3.445	0.00762
3.446	0.00393	3.446	0.00498	3.446	0.00594	3.446	0.00681	3.446	0.00762
3.447	0.00393	3.447	0.00498	3.447	0.00594	3.447	0.00681	3.447	0.00761
3.448	0.00393	3.448	0.00498	3.448	0.00593	3.448	0.00681	3.448	0.00761
3.449	0.00392	3.449	0.00498	3.449	0.00593	3.449	0.0068	3.449	0.0076
3.45	0.00392	3.45	0.00497	3.45	0.00593	3.45	0.0068	3.45	0.0076
3.451	0.00392	3.451	0.00497	3.451	0.00592	3.451	0.00679	3.451	0.0076
3.452	0.00392	3.452	0.00497	3.452	0.00592	3.452	0.00679	3.452	0.00759
3.453	0.00392	3.453	0.00497	3.453	0.00592	3.453	0.00679	3.453	0.00759
3.454	0.00391	3.454	0.00496	3.454	0.00591	3.454	0.00678	3.454	0.00758
3.455	0.00391	3.455	0.00496	3.455	0.00591	3.455	0.00678	3.455	0.00758
3.456	0.00391	3.456	0.00496	3.456	0.00591	3.456	0.00678	3.456	0.00757
3.457	0.00391	3.457	0.00496	3.457	0.00591	3.457	0.00677	3.457	0.00757
3.458	0.00391	3.458	0.00495	3.458	0.0059	3.458	0.00677	3.458	0.00757
3.459	0.00391	3.459	0.00495	3.459	0.0059	3.459	0.00676	3.459	0.00756
3.46	0.0039	3.46	0.00495	3.46	0.0059	3.46	0.00676	3.46	0.00756
3.461	0.0039	3.461	0.00495	3.461	0.00589	3.461	0.00676	3.461	0.00755
3.462	0.0039	3.462	0.00494	3.462	0.00589	3.462	0.00675	3.462	0.00755
3.463	0.0039	3.463	0.00494	3.463	0.00589	3.463	0.00675	3.463	0.00754
3.464	0.0039	3.464	0.00494	3.464	0.00588	3.464	0.00675	3.464	0.00754
3.465	0.00389	3.465	0.00494	3.465	0.00588	3.465	0.00674	3.465	0.00754
3.466	0.00389	3.466	0.00493	3.466	0.00588	3.466	0.00674	3.466	0.00753
3.467	0.00389	3.467	0.00493	3.467	0.00587	3.467	0.00674	3.467	0.00753
3.468	0.00389	3.468	0.00493	3.468	0.00587	3.468	0.00673	3.468	0.00752
3.469	0.00389	3.469	0.00493	3.469	0.00587	3.469	0.00673	3.469	0.00752
3.47	0.00388	3.47	0.00492	3.47	0.00586	3.47	0.00672	3.47	0.00752
3.471	0.00388	3.471	0.00492	3.471	0.00586	3.471	0.00672	3.471	0.00751
3.472	0.00388	3.472	0.00492	3.472	0.00586	3.472	0.00672	3.472	0.00751
3.473	0.00388	3.473	0.00492	3.473	0.00586	3.473	0.00671	3.473	0.0075
3.474	0.00388	3.474	0.00491	3.474	0.00585	3.474	0.00671	3.474	0.0075
3.475	0.00387	3.475	0.00491	3.475	0.00585	3.475	0.00671	3.475	0.00749
3.476	0.00387	3.476	0.00491	3.476	0.00585	3.476	0.0067	3.476	0.00749
3.477	0.00387	3.477	0.00491	3.477	0.00584	3.477	0.0067	3.477	0.00749
3.478	0.00387	3.478	0.0049	3.478	0.00584	3.478	0.0067	3.478	0.00748
3.479	0.00387	3.479	0.0049	3.479	0.00584	3.479	0.00669	3.479	0.00748
3.48	0.00387	3.48	0.0049	3.48	0.00583	3.48	0.00669	3.48	0.00747
3.481	0.00386	3.481	0.0049	3.481	0.00583	3.481	0.00668	3.481	0.00747
3.482	0.00386	3.482	0.00489	3.482	0.00583	3.482	0.00668	3.482	0.00747
3.483	0.00386	3.483	0.00489	3.483	0.00582	3.483	0.00668	3.483	0.00746
3.484	0.00386	3.484	0.00489	3.484	0.00582	3.484	0.00667	3.484	0.00746
3.485	0.00386	3.485	0.00489	3.485	0.00582	3.485	0.00667	3.485	0.00745
3.486	0.00385	3.486	0.00488	3.486	0.00582	3.486	0.00667	3.486	0.00745
3.487	0.00385	3.487	0.00488	3.487	0.00581	3.487	0.00666	3.487	0.00744
3.488	0.00385	3.488	0.00488	3.488	0.00581	3.488	0.00666	3.488	0.00744
3.489	0.00385	3.489	0.00488	3.489	0.00581	3.489	0.00666	3.489	0.00744
3.49	0.00385	3.49	0.00487	3.49	0.0058	3.49	0.00665	3.49	0.00743
3.491	0.00384	3.491	0.00487	3.491	0.0058	3.491	0.00665	3.491	0.00743
3.492	0.00384	3.492	0.00487	3.492	0.0058	3.492	0.00664	3.492	0.00742
3.493	0.00384	3.493	0.00487	3.493	0.00579	3.493	0.00664	3.493	0.00742
3.494	0.00384	3.494	0.00486	3.494	0.00579	3.494	0.00664	3.494	0.00742
3.495	0.00384	3.495	0.00486	3.495	0.00579	3.495	0.00663	3.495	0.00741
3.496	0.00384	3.496	0.00486	3.496	0.00578	3.496	0.00663	3.496	0.00741
3.497	0.00383	3.497	0.00486	3.497	0.00578	3.497	0.00663	3.497	0.0074
3.498	0.00383	3.498	0.00485	3.498	0.00578	3.498	0.00662	3.498	0.0074
3.499	0.00383	3.499	0.00485	3.499	0.00578	3.499	0.00662	3.499	0.0074
3.5	0.00383	3.5	0.00485	3.5	0.00577	3.5	0.00662	3.5	0.00739
3.501	0.00383	3.501	0.00485	3.501	0.00577	3.501	0.00661	3.501	0.00739
3.502	0.00382	3.502	0.00484	3.502	0.00577	3.502	0.00661	3.502	0.00738
3.503	0.00382	3.503	0.00484	3.503	0.00576	3.503	0.00661	3.503	0.00738
3.504	0.00382	3.504	0.00484	3.504	0.00576	3.504	0.0066	3.504	0.00738
3.505	0.00382	3.505	0.00484	3.505	0.00576	3.505	0.0066	3.505	0.00737
3.506	0.00382	3.506	0.00483	3.506	0.00575	3.506	0.00659	3.506	0.00737
3.507	0.00381	3.507	0.00483	3.507	0.00575	3.507	0.00659	3.507	0.00736
3.508	0.00381	3.508	0.00483	3.508	0.00575	3.508	0.00659	3.508	0.00736
3.509	0.00381	3.509	0.00483	3.509	0.00575	3.509	0.00658	3.509	0.00735
3.51	0.00381	3.51	0.00482	3.51	0.00574	3.51	0.00658	3.51	0.00735
3.511	0.00381	3.511	0.00482	3.511	0.00574	3.511	0.00658	3.511	0.00735
3.512	0.00381	3.512	0.00482	3.512	0.00574	3.512	0.00657	3.512	0.00734
3.513	0.0038	3.513	0.00482	3.513	0.00573	3.513	0.00657	3.513	0.00734
3.514	0.0038	3.514	0.00481	3.514	0.00573	3.514	0.00657	3.514	0.00733
3.515	0.0038	3.515	0.00481	3.515	0.00573	3.515	0.00656	3.515	0.00733
3.516	0.0038	3.516	0.00481	3.516	0.00572	3.516	0.00656	3.516	0.00733
3.517	0.0038	3.517	0.00481	3.517	0.00572	3.517	0.00656	3.517	0.00732
3.518	0.00379	3.518	0.0048	3.518	0.00572	3.518	0.00655	3.518	0.00732
3.519	0.00379	3.519	0.0048	3.519	0.00572	3.519	0.00655	3.519	0.00731
3.52	0.00379	3.52	0.0048	3.52	0.00571	3.52	0.00654	3.52	0.00731
3.521	0.00379	3.521	0.0048	3.521	0.00571	3.521	0.00654	3.521	0.00731
3.522	0.00379	3.522	0.00479	3.522	0.00571	3.522	0.00654	3.522	0.0073
3.523	0.00379	3.523	0.00479	3.523	0.0057	3.523	0.00653	3.523	0.0073
3.524	0.00378	3.524	0.00479	3.524	0.0057	3.524	0.00653	3.524	0.00729
3.525	0.00378	3.525	0.00479	3.525	0.0057	3.525	0.00653	3.525	0.00729
3.526	0.00378	3.526	0.00479	3.526	0.00569	3.526	0.00652	3.526	0.00729
3.527	0.00378	3.527	0.00478	3.527	0.00569	3.527	0.00652	3.527	0.00728
3.528	0.00378	3.528	0.00478	3.528	0.00569	3.528	0.00652	3.528	0.00728
3.529	0.00377	3.529	0.00478	3.529	0.00569	3.529	0.00651	3.529	0.00727
3.53	0.00377	3.53	0.00478	3.53	0.00568	3.53	0.00651	3.53	0.00727
3.531	0.00377	3.531	0.00477	3.531	0.00568	3.531	0.00651	3.531	0.00727
3.532	0.00377	3.532	0.00477	3.532	0.00568	3.532	0.0065	3.532	0.00726
3.533	0.00377	3.533	0.00477	3.533	0.00567	3.533	0.0065	3.533	0.00726
3.534	0.00377	3.534	0.00477	3.534	0.00567	3.534	0.0065	3.534	0.00725
3.535	0.00376	3.535	0.00476	3.535	0.00567	3.535	0.00649	3.535	0.00725
3.536	0.00376	3.536	0.00476	3.536	0.00566	3.536	0.00649	3.536	0.00725
3.537	0.00376	3.537	0.00476	3.537	0.00566	3.537	0.00649	3.537	0.00724

3.538	0.00376	3.538	0.00476	3.538	0.00566	3.538	0.00648	3.538	0.00724
3.539	0.00376	3.539	0.00475	3.539	0.00566	3.539	0.00648	3.539	0.00723
3.54	0.00375	3.54	0.00475	3.54	0.00565	3.54	0.00648	3.54	0.00723
3.541	0.00375	3.541	0.00475	3.541	0.00565	3.541	0.00647	3.541	0.00723
3.542	0.00375	3.542	0.00475	3.542	0.00565	3.542	0.00647	3.542	0.00722
3.543	0.00375	3.543	0.00474	3.543	0.00564	3.543	0.00646	3.543	0.00722
3.544	0.00375	3.544	0.00474	3.544	0.00564	3.544	0.00646	3.544	0.00722
3.545	0.00375	3.545	0.00474	3.545	0.00564	3.545	0.00646	3.545	0.00721
3.546	0.00374	3.546	0.00474	3.546	0.00564	3.546	0.00645	3.546	0.00721
3.547	0.00374	3.547	0.00474	3.547	0.00563	3.547	0.00645	3.547	0.0072
3.548	0.00374	3.548	0.00473	3.548	0.00563	3.548	0.00645	3.548	0.0072
3.549	0.00374	3.549	0.00473	3.549	0.00563	3.549	0.00644	3.549	0.0072
3.55	0.00374	3.55	0.00473	3.55	0.00562	3.55	0.00644	3.55	0.00719
3.551	0.00373	3.551	0.00473	3.551	0.00562	3.551	0.00644	3.551	0.00719
3.552	0.00373	3.552	0.00472	3.552	0.00562	3.552	0.00643	3.552	0.00718
3.553	0.00373	3.553	0.00472	3.553	0.00562	3.553	0.00643	3.553	0.00718
3.554	0.00373	3.554	0.00472	3.554	0.00561	3.554	0.00643	3.554	0.00718
3.555	0.00373	3.555	0.00472	3.555	0.00561	3.555	0.00642	3.555	0.00717
3.556	0.00373	3.556	0.00471	3.556	0.00561	3.556	0.00642	3.556	0.00717
3.557	0.00372	3.557	0.00471	3.557	0.0056	3.557	0.00642	3.557	0.00716
3.558	0.00372	3.558	0.00471	3.558	0.0056	3.558	0.00641	3.558	0.00716
3.559	0.00372	3.559	0.00471	3.559	0.0056	3.559	0.00641	3.559	0.00716
3.56	0.00372	3.56	0.0047	3.56	0.00559	3.56	0.00641	3.56	0.00715
3.561	0.00372	3.561	0.0047	3.561	0.00559	3.561	0.0064	3.561	0.00715
3.562	0.00372	3.562	0.0047	3.562	0.00559	3.562	0.0064	3.562	0.00714
3.563	0.00371	3.563	0.0047	3.563	0.00559	3.563	0.0064	3.563	0.00714
3.564	0.00371	3.564	0.00469	3.564	0.00558	3.564	0.00639	3.564	0.00714
3.565	0.00371	3.565	0.00469	3.565	0.00558	3.565	0.00639	3.565	0.00713
3.566	0.00371	3.566	0.00469	3.566	0.00558	3.566	0.00639	3.566	0.00713
3.567	0.00371	3.567	0.00469	3.567	0.00557	3.567	0.00638	3.567	0.00713
3.568	0.0037	3.568	0.00469	3.568	0.00557	3.568	0.00638	3.568	0.00712
3.569	0.0037	3.569	0.00468	3.569	0.00557	3.569	0.00638	3.569	0.00712
3.57	0.0037	3.57	0.00468	3.57	0.00557	3.57	0.00637	3.57	0.00711
3.571	0.0037	3.571	0.00468	3.571	0.00556	3.571	0.00637	3.571	0.00711
3.572	0.0037	3.572	0.00468	3.572	0.00556	3.572	0.00637	3.572	0.00711
3.573	0.0037	3.573	0.00467	3.573	0.00556	3.573	0.00636	3.573	0.0071
3.574	0.00369	3.574	0.00467	3.574	0.00555	3.574	0.00636	3.574	0.0071
3.575	0.00369	3.575	0.00467	3.575	0.00555	3.575	0.00636	3.575	0.00709
3.576	0.00369	3.576	0.00467	3.576	0.00555	3.576	0.00635	3.576	0.00709
3.577	0.00369	3.577	0.00466	3.577	0.00555	3.577	0.00635	3.577	0.00709
3.578	0.00369	3.578	0.00466	3.578	0.00554	3.578	0.00635	3.578	0.00708
3.579	0.00369	3.579	0.00466	3.579	0.00554	3.579	0.00634	3.579	0.00708
3.58	0.00368	3.58	0.00466	3.58	0.00554	3.58	0.00634	3.58	0.00708
3.581	0.00368	3.581	0.00466	3.581	0.00553	3.581	0.00634	3.581	0.00707
3.582	0.00368	3.582	0.00465	3.582	0.00553	3.582	0.00633	3.582	0.00707
3.583	0.00368	3.583	0.00465	3.583	0.00553	3.583	0.00633	3.583	0.00706
3.584	0.00368	3.584	0.00465	3.584	0.00553	3.584	0.00633	3.584	0.00706
3.585	0.00367	3.585	0.00465	3.585	0.00552	3.585	0.00632	3.585	0.00706
3.586	0.00367	3.586	0.00464	3.586	0.00552	3.586	0.00632	3.586	0.00705
3.587	0.00367	3.587	0.00464	3.587	0.00552	3.587	0.00632	3.587	0.00705
3.588	0.00367	3.588	0.00464	3.588	0.00551	3.588	0.00631	3.588	0.00705
3.589	0.00367	3.589	0.00464	3.589	0.00551	3.589	0.00631	3.589	0.00704
3.59	0.00367	3.59	0.00463	3.59	0.00551	3.59	0.00631	3.59	0.00704
3.591	0.00366	3.591	0.00463	3.591	0.00551	3.591	0.0063	3.591	0.00703
3.592	0.00366	3.592	0.00463	3.592	0.0055	3.592	0.0063	3.592	0.00703
3.593	0.00366	3.593	0.00463	3.593	0.0055	3.593	0.0063	3.593	0.00703
3.594	0.00366	3.594	0.00463	3.594	0.0055	3.594	0.00629	3.594	0.00702
3.595	0.00366	3.595	0.00462	3.595	0.0055	3.595	0.00629	3.595	0.00702
3.596	0.00366	3.596	0.00462	3.596	0.00549	3.596	0.00629	3.596	0.00702
3.597	0.00365	3.597	0.00462	3.597	0.00549	3.597	0.00628	3.597	0.00701
3.598	0.00365	3.598	0.00462	3.598	0.00549	3.598	0.00628	3.598	0.00701
3.599	0.00365	3.599	0.00461	3.599	0.00548	3.599	0.00628	3.599	0.007
3.6	0.00365	3.6	0.00461	3.6	0.00548	3.6	0.00627	3.6	0.007
3.601	0.00365	3.601	0.00461	3.601	0.00548	3.601	0.00627	3.601	0.007
3.602	0.00365	3.602	0.00461	3.602	0.00548	3.602	0.00627	3.602	0.00699
3.603	0.00364	3.603	0.00461	3.603	0.00547	3.603	0.00626	3.603	0.00699
3.604	0.00364	3.604	0.0046	3.604	0.00547	3.604	0.00626	3.604	0.00699
3.605	0.00364	3.605	0.0046	3.605	0.00547	3.605	0.00626	3.605	0.00698
3.606	0.00364	3.606	0.0046	3.606	0.00546	3.606	0.00625	3.606	0.00698
3.607	0.00364	3.607	0.0046	3.607	0.00546	3.607	0.00625	3.607	0.00697
3.608	0.00364	3.608	0.00459	3.608	0.00546	3.608	0.00625	3.608	0.00697
3.609	0.00363	3.609	0.00459	3.609	0.00546	3.609	0.00624	3.609	0.00697
3.61	0.00363	3.61	0.00459	3.61	0.00545	3.61	0.00624	3.61	0.00696
3.611	0.00363	3.611	0.00459	3.611	0.00545	3.611	0.00624	3.611	0.00696
3.612	0.00363	3.612	0.00458	3.612	0.00545	3.612	0.00623	3.612	0.00696
3.613	0.00363	3.613	0.00458	3.613	0.00545	3.613	0.00623	3.613	0.00695
3.614	0.00362	3.614	0.00458	3.614	0.00544	3.614	0.00623	3.614	0.00695
3.615	0.00362	3.615	0.00458	3.615	0.00544	3.615	0.00622	3.615	0.00694
3.616	0.00362	3.616	0.00458	3.616	0.00544	3.616	0.00622	3.616	0.00694
3.617	0.00362	3.617	0.00457	3.617	0.00543	3.617	0.00622	3.617	0.00694
3.618	0.00362	3.618	0.00457	3.618	0.00543	3.618	0.00621	3.618	0.00693
3.619	0.00362	3.619	0.00457	3.619	0.00543	3.619	0.00621	3.619	0.00693
3.62	0.00361	3.62	0.00457	3.62	0.00543	3.62	0.00621	3.62	0.00693
3.621	0.00361	3.621	0.00456	3.621	0.00542	3.621	0.0062	3.621	0.00692
3.622	0.00361	3.622	0.00456	3.622	0.00542	3.622	0.0062	3.622	0.00692
3.623	0.00361	3.623	0.00456	3.623	0.00542	3.623	0.0062	3.623	0.00691
3.624	0.00361	3.624	0.00456	3.624	0.00541	3.624	0.00619	3.624	0.00691
3.625	0.00361	3.625	0.00456	3.625	0.00541	3.625	0.00619	3.625	0.00691
3.626	0.0036	3.626	0.00455	3.626	0.00541	3.626	0.00619	3.626	0.0069
3.627	0.0036	3.627	0.00455	3.627	0.00541	3.627	0.00619	3.627	0.0069
3.628	0.0036	3.628	0.00455	3.628	0.0054	3.628	0.00618	3.628	0.0069
3.629	0.0036	3.629	0.00455	3.629	0.0054	3.629	0.00618	3.629	0.00689
3.63	0.0036	3.63	0.00454	3.63	0.0054	3.63	0.00618	3.63	0.00689
3.631	0.0036	3.631	0.00454	3.631	0.0054	3.631	0.00617	3.631	0.00689
3.632	0.00359	3.632	0.00454	3.632	0.00539	3.632	0.00617	3.632	0.00688
3.633	0.00359	3.633	0.00454	3.633	0.00539	3.633	0.00617	3.633	0.00688
3.634	0.00359	3.634	0.00454	3.634	0.00539	3.634	0.00616	3.634	0.00687
3.635	0.00359	3.635	0.00453	3.635	0.00538	3.635	0.00616	3.635	0.00687
3.636	0.00359	3.636	0.00453	3.636	0.00538	3.636	0.00616	3.636	0.00687
3.637	0.00359	3.637	0.00453	3.637	0.00538	3.637	0.00615	3.637	0.00686
3.638	0.00358	3.638	0.00453	3.638	0.00538	3.638	0.00615	3.638	0.00686
3.639	0.00358	3.639	0.00452	3.639	0.00537	3.639	0.00615	3.639	0.00686
3.64	0.00358	3.64	0.00452	3.64	0.00537	3.64	0.00614	3.64	0.00685
3.641	0.00358	3.641	0.00452	3.641	0.00537	3.641	0.00614	3.641	0.00685
3.642	0.00358	3.642	0.00452	3.642	0.00537	3.642	0.00614	3.642	0.00684
3.643	0.00358	3.643	0.00452	3.643	0.00536	3.643	0.00613	3.643	0.00684
3.644	0.00357	3.644	0.00451	3.644	0.00536	3.644	0.00613	3.644	0.00684
3.645	0.00357	3.645	0.00451	3.645	0.00536	3.645	0.00613	3.645	0.00683
3.646	0.00357	3.646	0.00451	3.646	0.00535	3.646	0.00612	3.646	0.00683
3.647	0.00357	3.647	0.00451	3.647	0.00535	3.647	0.00612	3.647	0.00683
3.648	0.00357	3.648	0.0045	3.648	0.00535	3.648	0.00612	3.648	0.00682
3.649	0.00357	3.649	0.0045	3.649	0.00535	3.649	0.00611	3.649	0.00682
3.65	0.00356	3.65	0.0045	3.65	0.00534	3.65	0.00611	3.65	0.00682
3.651	0.00356	3.651	0.0045	3.651	0.00534	3.651	0.00611	3.651	0.00681
3.652	0.00356	3.652	0.0045	3.652	0.00534	3.652	0.00611	3.652	0.00681
3.653	0.00356	3.653	0.00449	3.653	0.00534	3.653	0.0061	3.653	0.00681
3.654	0.00356	3.654	0.00449	3.654	0.00533	3.654	0.0061	3.654	0.0068
3.655	0.00356	3.655	0.00449	3.655	0.00533	3.655	0.0061	3.655	0.0068
3.656	0.00355	3.656	0.00449	3.656	0.00533	3.656	0.00609	3.656	0.00679
3.657	0.00355	3.657	0.00449	3.657	0.00533	3.657	0.00609	3.657	0.00679
3.658	0.00355	3.658	0.00448	3.658	0.00532	3.658	0.00609	3.658	0.00679
3.659	0.00355	3.659	0.00448	3.659	0.00532	3.659	0.00608	3.659	0.00678
3.66	0.00355	3.66	0.00448	3.66	0.00532	3.66	0.00608	3.66	0.00678
3.661	0.00355	3.661	0.00448	3.661	0.00531	3.661	0.00608	3.661	0.00678
3.662	0.00354	3.662	0.00447	3.662	0.00531	3.662	0.00607	3.662	0.00677
3.663	0.00354	3.663	0.00447	3.663	0.00531	3.663	0.00607	3.663	0.00677
3.664	0.00354	3.664	0.00447	3.664	0.00531	3.664	0.00607	3.664	0.00677
3.665	0.00354	3.665	0.00447	3.665	0.0053	3.665	0.00606	3.665	0.00676
3.666	0.00354	3.666	0.00447	3.666	0.0053	3.666	0.00606	3.666	0.00676
3.667	0.00354	3.667	0.00446	3.667	0.0053	3.667	0.00606	3.667	0.00676
3.668	0.00353	3.668	0.00446	3.668	0.0053	3.668	0.00606	3.668	0.00675
3.669	0.00353	3.669	0.00446	3.669	0.00529	3.669	0.00605	3.669	0.00675
3.67	0.00353	3.67	0.00446	3.67	0.00529	3.67	0.00605	3.67	0.00674
3.671	0.00353	3.671	0.00445	3.671	0.00529	3.671	0.00605	3.671	0.00674
3.672	0.00353	3.672	0.00445	3.672	0.00529	3.672	0.00604	3.672	0.00674

3.673	0.00353	3.673	0.00445	3.673	0.00528	3.673	0.00604	3.673	0.00673
3.674	0.00352	3.674	0.00445	3.674	0.00528	3.674	0.00604	3.674	0.00673
3.675	0.00352	3.675	0.00445	3.675	0.00528	3.675	0.00603	3.675	0.00673
3.676	0.00352	3.676	0.00444	3.676	0.00527	3.676	0.00603	3.676	0.00672
3.677	0.00352	3.677	0.00444	3.677	0.00527	3.677	0.00603	3.677	0.00672
3.678	0.00352	3.678	0.00444	3.678	0.00527	3.678	0.00602	3.678	0.00672
3.679	0.00352	3.679	0.00444	3.679	0.00527	3.679	0.00602	3.679	0.00671
3.68	0.00352	3.68	0.00444	3.68	0.00526	3.68	0.00602	3.68	0.00671
3.681	0.00351	3.681	0.00443	3.681	0.00526	3.681	0.00601	3.681	0.00671
3.682	0.00351	3.682	0.00443	3.682	0.00526	3.682	0.00601	3.682	0.0067
3.683	0.00351	3.683	0.00443	3.683	0.00526	3.683	0.00601	3.683	0.0067
3.684	0.00351	3.684	0.00443	3.684	0.00525	3.684	0.00601	3.684	0.0067
3.685	0.00351	3.685	0.00442	3.685	0.00525	3.685	0.006	3.685	0.00669
3.686	0.00351	3.686	0.00442	3.686	0.00525	3.686	0.006	3.686	0.00669
3.687	0.0035	3.687	0.00442	3.687	0.00525	3.687	0.006	3.687	0.00668
3.688	0.0035	3.688	0.00442	3.688	0.00524	3.688	0.00599	3.688	0.00668
3.689	0.0035	3.689	0.00442	3.689	0.00524	3.689	0.00599	3.689	0.00668
3.69	0.0035	3.69	0.00441	3.69	0.00524	3.69	0.00599	3.69	0.00667
3.691	0.0035	3.691	0.00441	3.691	0.00524	3.691	0.00598	3.691	0.00667
3.692	0.0035	3.692	0.00441	3.692	0.00523	3.692	0.00598	3.692	0.00667
3.693	0.00349	3.693	0.00441	3.693	0.00523	3.693	0.00598	3.693	0.00666
3.694	0.00349	3.694	0.00441	3.694	0.00523	3.694	0.00597	3.694	0.00666
3.695	0.00349	3.695	0.0044	3.695	0.00522	3.695	0.00597	3.695	0.00666
3.696	0.00349	3.696	0.0044	3.696	0.00522	3.696	0.00597	3.696	0.00665
3.697	0.00349	3.697	0.0044	3.697	0.00522	3.697	0.00597	3.697	0.00665
3.698	0.00349	3.698	0.0044	3.698	0.00522	3.698	0.00596	3.698	0.00665
3.699	0.00348	3.699	0.00439	3.699	0.00521	3.699	0.00596	3.699	0.00664
3.7	0.00348	3.7	0.00439	3.7	0.00521	3.7	0.00596	3.7	0.00664
3.701	0.00348	3.701	0.00439	3.701	0.00521	3.701	0.00595	3.701	0.00664
3.702	0.00348	3.702	0.00439	3.702	0.00521	3.702	0.00595	3.702	0.00663
3.703	0.00348	3.703	0.00439	3.703	0.0052	3.703	0.00595	3.703	0.00663
3.704	0.00348	3.704	0.00438	3.704	0.0052	3.704	0.00594	3.704	0.00663
3.705	0.00348	3.705	0.00438	3.705	0.0052	3.705	0.00594	3.705	0.00662
3.706	0.00347	3.706	0.00438	3.706	0.0052	3.706	0.00594	3.706	0.00662
3.707	0.00347	3.707	0.00438	3.707	0.00519	3.707	0.00594	3.707	0.00662
3.708	0.00347	3.708	0.00438	3.708	0.00519	3.708	0.00593	3.708	0.00661
3.709	0.00347	3.709	0.00437	3.709	0.00519	3.709	0.00593	3.709	0.00661
3.71	0.00347	3.71	0.00437	3.71	0.00519	3.71	0.00593	3.71	0.0066
3.711	0.00347	3.711	0.00437	3.711	0.00518	3.711	0.00592	3.711	0.0066
3.712	0.00346	3.712	0.00437	3.712	0.00518	3.712	0.00592	3.712	0.0066
3.713	0.00346	3.713	0.00437	3.713	0.00518	3.713	0.00592	3.713	0.00659
3.714	0.00346	3.714	0.00436	3.714	0.00518	3.714	0.00591	3.714	0.00659
3.715	0.00346	3.715	0.00436	3.715	0.00517	3.715	0.00591	3.715	0.00659
3.716	0.00346	3.716	0.00436	3.716	0.00517	3.716	0.00591	3.716	0.00658
3.717	0.00346	3.717	0.00436	3.717	0.00517	3.717	0.00591	3.717	0.00658
3.718	0.00345	3.718	0.00436	3.718	0.00517	3.718	0.0059	3.718	0.00658
3.719	0.00345	3.719	0.00435	3.719	0.00516	3.719	0.0059	3.719	0.00657
3.72	0.00345	3.72	0.00435	3.72	0.00516	3.72	0.0059	3.72	0.00657
3.721	0.00345	3.721	0.00435	3.721	0.00516	3.721	0.00589	3.721	0.00657
3.722	0.00345	3.722	0.00435	3.722	0.00516	3.722	0.00589	3.722	0.00656
3.723	0.00345	3.723	0.00434	3.723	0.00515	3.723	0.00589	3.723	0.00656
3.724	0.00344	3.724	0.00434	3.724	0.00515	3.724	0.00588	3.724	0.00656
3.725	0.00344	3.725	0.00434	3.725	0.00515	3.725	0.00588	3.725	0.00655
3.726	0.00344	3.726	0.00434	3.726	0.00515	3.726	0.00588	3.726	0.00655
3.727	0.00344	3.727	0.00434	3.727	0.00514	3.727	0.00588	3.727	0.00655
3.728	0.00344	3.728	0.00433	3.728	0.00514	3.728	0.00587	3.728	0.00654
3.729	0.00344	3.729	0.00433	3.729	0.00514	3.729	0.00587	3.729	0.00654
3.73	0.00344	3.73	0.00433	3.73	0.00514	3.73	0.00587	3.73	0.00654
3.731	0.00343	3.731	0.00433	3.731	0.00513	3.731	0.00586	3.731	0.00653
3.732	0.00343	3.732	0.00433	3.732	0.00513	3.732	0.00586	3.732	0.00653
3.733	0.00343	3.733	0.00432	3.733	0.00513	3.733	0.00586	3.733	0.00653
3.734	0.00343	3.734	0.00432	3.734	0.00513	3.734	0.00585	3.734	0.00652
3.735	0.00343	3.735	0.00432	3.735	0.00512	3.735	0.00585	3.735	0.00652
3.736	0.00343	3.736	0.00432	3.736	0.00512	3.736	0.00585	3.736	0.00652
3.737	0.00342	3.737	0.00432	3.737	0.00512	3.737	0.00585	3.737	0.00651
3.738	0.00342	3.738	0.00431	3.738	0.00512	3.738	0.00584	3.738	0.00651
3.739	0.00342	3.739	0.00431	3.739	0.00511	3.739	0.00584	3.739	0.00651
3.74	0.00342	3.74	0.00431	3.74	0.00511	3.74	0.00584	3.74	0.0065
3.741	0.00342	3.741	0.00431	3.741	0.00511	3.741	0.00583	3.741	0.0065
3.742	0.00342	3.742	0.00431	3.742	0.0051	3.742	0.00583	3.742	0.0065
3.743	0.00342	3.743	0.0043	3.743	0.0051	3.743	0.00583	3.743	0.00649
3.744	0.00341	3.744	0.0043	3.744	0.0051	3.744	0.00582	3.744	0.00649
3.745	0.00341	3.745	0.0043	3.745	0.0051	3.745	0.00582	3.745	0.00649
3.746	0.00341	3.746	0.0043	3.746	0.00509	3.746	0.00582	3.746	0.00648
3.747	0.00341	3.747	0.0043	3.747	0.00509	3.747	0.00582	3.747	0.00648
3.748	0.00341	3.748	0.00429	3.748	0.00509	3.748	0.00581	3.748	0.00648
3.749	0.00341	3.749	0.00429	3.749	0.00509	3.749	0.00581	3.749	0.00647
3.75	0.0034	3.75	0.00429	3.75	0.00508	3.75	0.00581	3.75	0.00647
3.751	0.0034	3.751	0.00429	3.751	0.00508	3.751	0.0058	3.751	0.00647
3.752	0.0034	3.752	0.00429	3.752	0.00508	3.752	0.0058	3.752	0.00646
3.753	0.0034	3.753	0.00428	3.753	0.00508	3.753	0.0058	3.753	0.00646
3.754	0.0034	3.754	0.00428	3.754	0.00508	3.754	0.0058	3.754	0.00646
3.755	0.0034	3.755	0.00428	3.755	0.00507	3.755	0.00579	3.755	0.00645
3.756	0.0034	3.756	0.00428	3.756	0.00507	3.756	0.00579	3.756	0.00645
3.757	0.00339	3.757	0.00428	3.757	0.00507	3.757	0.00579	3.757	0.00645
3.758	0.00339	3.758	0.00427	3.758	0.00507	3.758	0.00578	3.758	0.00644
3.759	0.00339	3.759	0.00427	3.759	0.00506	3.759	0.00578	3.759	0.00644
3.76	0.00339	3.76	0.00427	3.76	0.00506	3.76	0.00578	3.76	0.00644
3.761	0.00339	3.761	0.00427	3.761	0.00506	3.761	0.00578	3.761	0.00643
3.762	0.00339	3.762	0.00427	3.762	0.00506	3.762	0.00577	3.762	0.00643
3.763	0.00338	3.763	0.00426	3.763	0.00505	3.763	0.00577	3.763	0.00643
3.764	0.00338	3.764	0.00426	3.764	0.00505	3.764	0.00577	3.764	0.00642
3.765	0.00338	3.765	0.00426	3.765	0.00505	3.765	0.00576	3.765	0.00642
3.766	0.00338	3.766	0.00426	3.766	0.00505	3.766	0.00576	3.766	0.00642
3.767	0.00338	3.767	0.00426	3.767	0.00504	3.767	0.00576	3.767	0.00641
3.768	0.00338	3.768	0.00425	3.768	0.00504	3.768	0.00576	3.768	0.00641
3.769	0.00338	3.769	0.00425	3.769	0.00504	3.769	0.00575	3.769	0.00641
3.77	0.00337	3.77	0.00425	3.77	0.00504	3.77	0.00575	3.77	0.0064
3.771	0.00337	3.771	0.00425	3.771	0.00503	3.771	0.00575	3.771	0.0064
3.772	0.00337	3.772	0.00425	3.772	0.00503	3.772	0.00574	3.772	0.0064
3.773	0.00337	3.773	0.00424	3.773	0.00503	3.773	0.00574	3.773	0.00639
3.774	0.00337	3.774	0.00424	3.774	0.00503	3.774	0.00574	3.774	0.00639
3.775	0.00337	3.775	0.00424	3.775	0.00502	3.775	0.00573	3.775	0.00639
3.776	0.00336	3.776	0.00424	3.776	0.00502	3.776	0.00573	3.776	0.00638
3.777	0.00336	3.777	0.00424	3.777	0.00502	3.777	0.00573	3.777	0.00638
3.778	0.00336	3.778	0.00423	3.778	0.00502	3.778	0.00573	3.778	0.00638
3.779	0.00336	3.779	0.00423	3.779	0.00501	3.779	0.00572	3.779	0.00637
3.78	0.00336	3.78	0.00423	3.78	0.00501	3.78	0.00572	3.78	0.00637
3.781	0.00336	3.781	0.00423	3.781	0.00501	3.781	0.00572	3.781	0.00637
3.782	0.00336	3.782	0.00423	3.782	0.00501	3.782	0.00571	3.782	0.00636
3.783	0.00335	3.783	0.00422	3.783	0.005	3.783	0.00571	3.783	0.00636
3.784	0.00335	3.784	0.00422	3.784	0.005	3.784	0.00571	3.784	0.00636
3.785	0.00335	3.785	0.00422	3.785	0.005	3.785	0.00571	3.785	0.00635
3.786	0.00335	3.786	0.00422	3.786	0.005	3.786	0.0057	3.786	0.00635
3.787	0.00335	3.787	0.00422	3.787	0.00499	3.787	0.0057	3.787	0.00635
3.788	0.00335	3.788	0.00421	3.788	0.00499	3.788	0.0057	3.788	0.00634
3.789	0.00335	3.789	0.00421	3.789	0.00499	3.789	0.0057	3.789	0.00634
3.79	0.00334	3.79	0.00421	3.79	0.00499	3.79	0.00569	3.79	0.00634
3.791	0.00334	3.791	0.00421	3.791	0.00498	3.791	0.00569	3.791	0.00633
3.792	0.00334	3.792	0.00421	3.792	0.00498	3.792	0.00569	3.792	0.00633
3.793	0.00334	3.793	0.0042	3.793	0.00498	3.793	0.00568	3.793	0.00633
3.794	0.00334	3.794	0.0042	3.794	0.00498	3.794	0.00568	3.794	0.00633
3.795	0.00334	3.795	0.0042	3.795	0.00497	3.795	0.00568	3.795	0.00632
3.796	0.00333	3.796	0.0042	3.796	0.00497	3.796	0.00568	3.796	0.00632
3.797	0.00333	3.797	0.0042	3.797	0.00497	3.797	0.00567	3.797	0.00632
3.798	0.00333	3.798	0.00419	3.798	0.00497	3.798	0.00567	3.798	0.00631
3.799	0.00333	3.799	0.00419	3.799	0.00497	3.799	0.00567	3.799	0.00631
3.8	0.00333	3.8	0.00419	3.8	0.00496	3.8	0.00566	3.8	0.00631
3.801	0.00333	3.801	0.00419	3.801	0.00496	3.801	0.00566	3.801	0.0063
3.802	0.00333	3.802	0.00419	3.802	0.00496	3.802	0.00566	3.802	0.0063
3.803	0.00332	3.803	0.00418	3.803	0.00496	3.803	0.00566	3.803	0.0063
3.804	0.00332	3.804	0.00418	3.804	0.00495	3.804	0.00565	3.804	0.00629
3.805	0.00332	3.805	0.00418	3.805	0.00495	3.805	0.00565	3.805	0.00629
3.806	0.00332	3.806	0.00418	3.806	0.00495	3.806	0.00565	3.806	0.00629
3.807	0.00332	3.807	0.00418	3.807	0.00495	3.807	0.00564	3.807	0.00628

3.808	0.00332	3.808	0.00417	3.808	0.00494	3.808	0.00564	3.808	0.00628
3.809	0.00332	3.809	0.00417	3.809	0.00494	3.809	0.00564	3.809	0.00628
3.81	0.00331	3.81	0.00417	3.81	0.00494	3.81	0.00564	3.81	0.00627
3.811	0.00331	3.811	0.00417	3.811	0.00494	3.811	0.00563	3.811	0.00627
3.812	0.00331	3.812	0.00417	3.812	0.00493	3.812	0.00563	3.812	0.00627
3.813	0.00331	3.813	0.00416	3.813	0.00493	3.813	0.00563	3.813	0.00626
3.814	0.00331	3.814	0.00416	3.814	0.00493	3.814	0.00562	3.814	0.00626
3.815	0.00331	3.815	0.00416	3.815	0.00493	3.815	0.00562	3.815	0.00626
3.816	0.00331	3.816	0.00416	3.816	0.00492	3.816	0.00562	3.816	0.00626
3.817	0.0033	3.817	0.00416	3.817	0.00492	3.817	0.00562	3.817	0.00625
3.818	0.0033	3.818	0.00415	3.818	0.00492	3.818	0.00561	3.818	0.00625
3.819	0.0033	3.819	0.00415	3.819	0.00492	3.819	0.00561	3.819	0.00625
3.82	0.0033	3.82	0.00415	3.82	0.00492	3.82	0.00561	3.82	0.00624
3.821	0.0033	3.821	0.00415	3.821	0.00491	3.821	0.00561	3.821	0.00624
3.822	0.0033	3.822	0.00415	3.822	0.00491	3.822	0.0056	3.822	0.00624
3.823	0.00329	3.823	0.00415	3.823	0.00491	3.823	0.0056	3.823	0.00623
3.824	0.00329	3.824	0.00414	3.824	0.00491	3.824	0.0056	3.824	0.00623
3.825	0.00329	3.825	0.00414	3.825	0.0049	3.825	0.00559	3.825	0.00623
3.826	0.00329	3.826	0.00414	3.826	0.0049	3.826	0.00559	3.826	0.00622
3.827	0.00329	3.827	0.00414	3.827	0.0049	3.827	0.00559	3.827	0.00622
3.828	0.00329	3.828	0.00414	3.828	0.0049	3.828	0.00559	3.828	0.00622
3.829	0.00329	3.829	0.00413	3.829	0.00489	3.829	0.00558	3.829	0.00621
3.83	0.00328	3.83	0.00413	3.83	0.00489	3.83	0.00558	3.83	0.00621
3.831	0.00328	3.831	0.00413	3.831	0.00489	3.831	0.00558	3.831	0.00621
3.832	0.00328	3.832	0.00413	3.832	0.00489	3.832	0.00557	3.832	0.0062
3.833	0.00328	3.833	0.00413	3.833	0.00488	3.833	0.00557	3.833	0.0062
3.834	0.00328	3.834	0.00412	3.834	0.00488	3.834	0.00557	3.834	0.0062
3.835	0.00328	3.835	0.00412	3.835	0.00488	3.835	0.00557	3.835	0.0062
3.836	0.00328	3.836	0.00412	3.836	0.00488	3.836	0.00556	3.836	0.00619
3.837	0.00327	3.837	0.00412	3.837	0.00488	3.837	0.00556	3.837	0.00619
3.838	0.00327	3.838	0.00412	3.838	0.00487	3.838	0.00556	3.838	0.00619
3.839	0.00327	3.839	0.00411	3.839	0.00487	3.839	0.00556	3.839	0.00618
3.84	0.00327	3.84	0.00411	3.84	0.00487	3.84	0.00555	3.84	0.00618
3.841	0.00327	3.841	0.00411	3.841	0.00487	3.841	0.00555	3.841	0.00618
3.842	0.00327	3.842	0.00411	3.842	0.00486	3.842	0.00555	3.842	0.00617
3.843	0.00327	3.843	0.00411	3.843	0.00486	3.843	0.00554	3.843	0.00617
3.844	0.00326	3.844	0.0041	3.844	0.00486	3.844	0.00554	3.844	0.00617
3.845	0.00326	3.845	0.0041	3.845	0.00486	3.845	0.00554	3.845	0.00616
3.846	0.00326	3.846	0.0041	3.846	0.00485	3.846	0.00554	3.846	0.00616
3.847	0.00326	3.847	0.0041	3.847	0.00485	3.847	0.00553	3.847	0.00616
3.848	0.00326	3.848	0.0041	3.848	0.00485	3.848	0.00553	3.848	0.00615
3.849	0.00326	3.849	0.0041	3.849	0.00485	3.849	0.00553	3.849	0.00615
3.85	0.00326	3.85	0.00409	3.85	0.00484	3.85	0.00553	3.85	0.00615
3.851	0.00325	3.851	0.00409	3.851	0.00484	3.851	0.00552	3.851	0.00615
3.852	0.00325	3.852	0.00409	3.852	0.00484	3.852	0.00552	3.852	0.00614
3.853	0.00325	3.853	0.00409	3.853	0.00484	3.853	0.00552	3.853	0.00614
3.854	0.00325	3.854	0.00409	3.854	0.00484	3.854	0.00551	3.854	0.00614
3.855	0.00325	3.855	0.00408	3.855	0.00483	3.855	0.00551	3.855	0.00613
3.856	0.00325	3.856	0.00408	3.856	0.00483	3.856	0.00551	3.856	0.00613
3.857	0.00325	3.857	0.00408	3.857	0.00483	3.857	0.00551	3.857	0.00613
3.858	0.00324	3.858	0.00408	3.858	0.00483	3.858	0.0055	3.858	0.00612
3.859	0.00324	3.859	0.00408	3.859	0.00482	3.859	0.0055	3.859	0.00612
3.86	0.00324	3.86	0.00407	3.86	0.00482	3.86	0.0055	3.86	0.00612
3.861	0.00324	3.861	0.00407	3.861	0.00482	3.861	0.0055	3.861	0.00611
3.862	0.00324	3.862	0.00407	3.862	0.00482	3.862	0.00549	3.862	0.00611
3.863	0.00324	3.863	0.00407	3.863	0.00481	3.863	0.00549	3.863	0.00611
3.864	0.00324	3.864	0.00407	3.864	0.00481	3.864	0.00549	3.864	0.00611
3.865	0.00323	3.865	0.00407	3.865	0.00481	3.865	0.00549	3.865	0.0061
3.866	0.00323	3.866	0.00406	3.866	0.00481	3.866	0.00548	3.866	0.0061
3.867	0.00323	3.867	0.00406	3.867	0.00481	3.867	0.00548	3.867	0.0061
3.868	0.00323	3.868	0.00406	3.868	0.0048	3.868	0.00548	3.868	0.00609
3.869	0.00323	3.869	0.00406	3.869	0.0048	3.869	0.00547	3.869	0.00609
3.87	0.00323	3.87	0.00406	3.87	0.0048	3.87	0.00547	3.87	0.00609
3.871	0.00323	3.871	0.00405	3.871	0.0048	3.871	0.00547	3.871	0.00608
3.872	0.00322	3.872	0.00405	3.872	0.00479	3.872	0.00547	3.872	0.00608
3.873	0.00322	3.873	0.00405	3.873	0.00479	3.873	0.00546	3.873	0.00608
3.874	0.00322	3.874	0.00405	3.874	0.00479	3.874	0.00546	3.874	0.00608
3.875	0.00322	3.875	0.00405	3.875	0.00479	3.875	0.00546	3.875	0.00607
3.876	0.00322	3.876	0.00404	3.876	0.00479	3.876	0.00546	3.876	0.00607
3.877	0.00322	3.877	0.00404	3.877	0.00478	3.877	0.00545	3.877	0.00607
3.878	0.00322	3.878	0.00404	3.878	0.00478	3.878	0.00545	3.878	0.00606
3.879	0.00321	3.879	0.00404	3.879	0.00478	3.879	0.00545	3.879	0.00606
3.88	0.00321	3.88	0.00404	3.88	0.00478	3.88	0.00545	3.88	0.00606
3.881	0.00321	3.881	0.00404	3.881	0.00477	3.881	0.00544	3.881	0.00605
3.882	0.00321	3.882	0.00403	3.882	0.00477	3.882	0.00544	3.882	0.00605
3.883	0.00321	3.883	0.00403	3.883	0.00477	3.883	0.00544	3.883	0.00605
3.884	0.00321	3.884	0.00403	3.884	0.00477	3.884	0.00543	3.884	0.00605
3.885	0.00321	3.885	0.00403	3.885	0.00476	3.885	0.00543	3.885	0.00604
3.886	0.0032	3.886	0.00403	3.886	0.00476	3.886	0.00543	3.886	0.00604
3.887	0.0032	3.887	0.00402	3.887	0.00476	3.887	0.00543	3.887	0.00604
3.888	0.0032	3.888	0.00402	3.888	0.00476	3.888	0.00542	3.888	0.00603
3.889	0.0032	3.889	0.00402	3.889	0.00476	3.889	0.00542	3.889	0.00603
3.89	0.0032	3.89	0.00402	3.89	0.00475	3.89	0.00542	3.89	0.00603
3.891	0.0032	3.891	0.00402	3.891	0.00475	3.891	0.00542	3.891	0.00602
3.892	0.0032	3.892	0.00402	3.892	0.00475	3.892	0.00541	3.892	0.00602
3.893	0.00319	3.893	0.00401	3.893	0.00475	3.893	0.00541	3.893	0.00602
3.894	0.00319	3.894	0.00401	3.894	0.00474	3.894	0.00541	3.894	0.00602
3.895	0.00319	3.895	0.00401	3.895	0.00474	3.895	0.00541	3.895	0.00601
3.896	0.00319	3.896	0.00401	3.896	0.00474	3.896	0.0054	3.896	0.00601
3.897	0.00319	3.897	0.00401	3.897	0.00474	3.897	0.0054	3.897	0.00601
3.898	0.00319	3.898	0.004	3.898	0.00474	3.898	0.0054	3.898	0.006
3.899	0.00319	3.899	0.004	3.899	0.00473	3.899	0.0054	3.899	0.006
3.9	0.00319	3.9	0.004	3.9	0.00473	3.9	0.00539	3.9	0.006
3.901	0.00318	3.901	0.004	3.901	0.00473	3.901	0.00539	3.901	0.00599
3.902	0.00318	3.902	0.004	3.902	0.00473	3.902	0.00539	3.902	0.00599
3.903	0.00318	3.903	0.004	3.903	0.00472	3.903	0.00538	3.903	0.00599
3.904	0.00318	3.904	0.00399	3.904	0.00472	3.904	0.00538	3.904	0.00599
3.905	0.00318	3.905	0.00399	3.905	0.00472	3.905	0.00538	3.905	0.00598
3.906	0.00318	3.906	0.00399	3.906	0.00472	3.906	0.00538	3.906	0.00598
3.907	0.00318	3.907	0.00399	3.907	0.00472	3.907	0.00537	3.907	0.00598
3.908	0.00317	3.908	0.00399	3.908	0.00471	3.908	0.00537	3.908	0.00597
3.909	0.00317	3.909	0.00398	3.909	0.00471	3.909	0.00537	3.909	0.00597
3.91	0.00317	3.91	0.00398	3.91	0.00471	3.91	0.00537	3.91	0.00597
3.911	0.00317	3.911	0.00398	3.911	0.00471	3.911	0.00536	3.911	0.00596
3.912	0.00317	3.912	0.00398	3.912	0.0047	3.912	0.00536	3.912	0.00596
3.913	0.00317	3.913	0.00398	3.913	0.0047	3.913	0.00536	3.913	0.00596
3.914	0.00317	3.914	0.00398	3.914	0.0047	3.914	0.00536	3.914	0.00596
3.915	0.00316	3.915	0.00397	3.915	0.0047	3.915	0.00535	3.915	0.00595
3.916	0.00316	3.916	0.00397	3.916	0.0047	3.916	0.00535	3.916	0.00595
3.917	0.00316	3.917	0.00397	3.917	0.00469	3.917	0.00535	3.917	0.00595
3.918	0.00316	3.918	0.00397	3.918	0.00469	3.918	0.00535	3.918	0.00594
3.919	0.00316	3.919	0.00397	3.919	0.00469	3.919	0.00534	3.919	0.00594
3.92	0.00316	3.92	0.00396	3.92	0.00469	3.92	0.00534	3.92	0.00594
3.921	0.00316	3.921	0.00396	3.921	0.00468	3.921	0.00534	3.921	0.00594
3.922	0.00315	3.922	0.00396	3.922	0.00468	3.922	0.00534	3.922	0.00593
3.923	0.00315	3.923	0.00396	3.923	0.00468	3.923	0.00533	3.923	0.00593
3.924	0.00315	3.924	0.00396	3.924	0.00468	3.924	0.00533	3.924	0.00593
3.925	0.00315	3.925	0.00396	3.925	0.00468	3.925	0.00533	3.925	0.00592
3.926	0.00315	3.926	0.00395	3.926	0.00467	3.926	0.00533	3.926	0.00592
3.927	0.00315	3.927	0.00395	3.927	0.00467	3.927	0.00532	3.927	0.00592
3.928	0.00315	3.928	0.00395	3.928	0.00467	3.928	0.00532	3.928	0.00592
3.929	0.00315	3.929	0.00395	3.929	0.00467	3.929	0.00532	3.929	0.00591
3.93	0.00314	3.93	0.00395	3.93	0.00467	3.93	0.00532	3.93	0.00591
3.931	0.00314	3.931	0.00394	3.931	0.00466	3.931	0.00531	3.931	0.00591
3.932	0.00314	3.932	0.00394	3.932	0.00466	3.932	0.00531	3.932	0.0059
3.933	0.00314	3.933	0.00394	3.933	0.00466	3.933	0.00531	3.933	0.0059
3.934	0.00314	3.934	0.00394	3.934	0.00466	3.934	0.00531	3.934	0.0059
3.935	0.00314	3.935	0.00394	3.935	0.00465	3.935	0.0053	3.935	0.00589
3.936	0.00314	3.936	0.00394	3.936	0.00465	3.936	0.0053	3.936	0.00589
3.937	0.00313	3.937	0.00393	3.937	0.00465	3.937	0.0053	3.937	0.00589
3.938	0.00313	3.938	0.00393	3.938	0.00465	3.938	0.00529	3.938	0.00589
3.939	0.00313	3.939	0.00393	3.939	0.00465	3.939	0.00529	3.939	0.00588
3.94	0.00313	3.94	0.00393	3.94	0.00464	3.94	0.00529	3.94	0.00588
3.941	0.00313	3.941	0.00393	3.941	0.00464	3.941	0.00529	3.941	0.00588
3.942	0.00313	3.942	0.00393	3.942	0.00464	3.942	0.00528	3.942	0.00587

3.943	0.00313	3.943	0.00392	3.943	0.00464	3.943	0.00528	3.943	0.00587
3.944	0.00312	3.944	0.00392	3.944	0.00463	3.944	0.00528	3.944	0.00587
3.945	0.00312	3.945	0.00392	3.945	0.00463	3.945	0.00528	3.945	0.00587
3.946	0.00312	3.946	0.00392	3.946	0.00463	3.946	0.00527	3.946	0.00586
3.947	0.00312	3.947	0.00392	3.947	0.00463	3.947	0.00527	3.947	0.00586
3.948	0.00312	3.948	0.00391	3.948	0.00463	3.948	0.00527	3.948	0.00586
3.949	0.00312	3.949	0.00391	3.949	0.00462	3.949	0.00527	3.949	0.00585
3.95	0.00312	3.95	0.00391	3.95	0.00462	3.95	0.00526	3.95	0.00585
3.951	0.00312	3.951	0.00391	3.951	0.00462	3.951	0.00526	3.951	0.00585
3.952	0.00311	3.952	0.00391	3.952	0.00462	3.952	0.00526	3.952	0.00585
3.953	0.00311	3.953	0.00391	3.953	0.00462	3.953	0.00526	3.953	0.00584
3.954	0.00311	3.954	0.0039	3.954	0.00461	3.954	0.00525	3.954	0.00584
3.955	0.00311	3.955	0.0039	3.955	0.00461	3.955	0.00525	3.955	0.00584
3.956	0.00311	3.956	0.0039	3.956	0.00461	3.956	0.00525	3.956	0.00583
3.957	0.00311	3.957	0.0039	3.957	0.00461	3.957	0.00525	3.957	0.00583
3.958	0.00311	3.958	0.0039	3.958	0.0046	3.958	0.00524	3.958	0.00583
3.959	0.0031	3.959	0.0039	3.959	0.0046	3.959	0.00524	3.959	0.00583
3.96	0.0031	3.96	0.00389	3.96	0.0046	3.96	0.00524	3.96	0.00582
3.961	0.0031	3.961	0.00389	3.961	0.0046	3.961	0.00524	3.961	0.00582
3.962	0.0031	3.962	0.00389	3.962	0.0046	3.962	0.00523	3.962	0.00582
3.963	0.0031	3.963	0.00389	3.963	0.00459	3.963	0.00523	3.963	0.00581
3.964	0.0031	3.964	0.00389	3.964	0.00459	3.964	0.00523	3.964	0.00581
3.965	0.0031	3.965	0.00389	3.965	0.00459	3.965	0.00523	3.965	0.00581
3.966	0.0031	3.966	0.00388	3.966	0.00459	3.966	0.00522	3.966	0.00581
3.967	0.00309	3.967	0.00388	3.967	0.00459	3.967	0.00522	3.967	0.0058
3.968	0.00309	3.968	0.00388	3.968	0.00458	3.968	0.00522	3.968	0.0058
3.969	0.00309	3.969	0.00388	3.969	0.00458	3.969	0.00522	3.969	0.0058
3.97	0.00309	3.97	0.00388	3.97	0.00458	3.97	0.00521	3.97	0.00579
3.971	0.00309	3.971	0.00387	3.971	0.00458	3.971	0.00521	3.971	0.00579
3.972	0.00309	3.972	0.00387	3.972	0.00457	3.972	0.00521	3.972	0.00579
3.973	0.00309	3.973	0.00387	3.973	0.00457	3.973	0.00521	3.973	0.00579
3.974	0.00309	3.974	0.00387	3.974	0.00457	3.974	0.0052	3.974	0.00578
3.975	0.00308	3.975	0.00387	3.975	0.00457	3.975	0.0052	3.975	0.00578
3.976	0.00308	3.976	0.00387	3.976	0.00457	3.976	0.0052	3.976	0.00578
3.977	0.00308	3.977	0.00386	3.977	0.00456	3.977	0.0052	3.977	0.00578
3.978	0.00308	3.978	0.00386	3.978	0.00456	3.978	0.00519	3.978	0.00577
3.979	0.00308	3.979	0.00386	3.979	0.00456	3.979	0.00519	3.979	0.00577
3.98	0.00308	3.98	0.00386	3.98	0.00456	3.98	0.00519	3.98	0.00577
3.981	0.00308	3.981	0.00386	3.981	0.00456	3.981	0.00519	3.981	0.00576
3.982	0.00307	3.982	0.00386	3.982	0.00455	3.982	0.00518	3.982	0.00576
3.983	0.00307	3.983	0.00385	3.983	0.00455	3.983	0.00518	3.983	0.00576
3.984	0.00307	3.984	0.00385	3.984	0.00455	3.984	0.00518	3.984	0.00576
3.985	0.00307	3.985	0.00385	3.985	0.00455	3.985	0.00518	3.985	0.00575
3.986	0.00307	3.986	0.00385	3.986	0.00455	3.986	0.00518	3.986	0.00575
3.987	0.00307	3.987	0.00385	3.987	0.00454	3.987	0.00517	3.987	0.00575
3.988	0.00307	3.988	0.00385	3.988	0.00454	3.988	0.00517	3.988	0.00574
3.989	0.00307	3.989	0.00384	3.989	0.00454	3.989	0.00517	3.989	0.00574
3.99	0.00306	3.99	0.00384	3.99	0.00454	3.99	0.00517	3.99	0.00574
3.991	0.00306	3.991	0.00384	3.991	0.00453	3.991	0.00516	3.991	0.00574
3.992	0.00306	3.992	0.00384	3.992	0.00453	3.992	0.00516	3.992	0.00573
3.993	0.00306	3.993	0.00384	3.993	0.00453	3.993	0.00516	3.993	0.00573
3.994	0.00306	3.994	0.00384	3.994	0.00453	3.994	0.00516	3.994	0.00573
3.995	0.00306	3.995	0.00383	3.995	0.00453	3.995	0.00515	3.995	0.00573
3.996	0.00306	3.996	0.00383	3.996	0.00452	3.996	0.00515	3.996	0.00572
3.997	0.00305	3.997	0.00383	3.997	0.00452	3.997	0.00515	3.997	0.00572
3.998	0.00305	3.998	0.00383	3.998	0.00452	3.998	0.00515	3.998	0.00572
3.999	0.00305	3.999	0.00383	3.999	0.00452	3.999	0.00514	3.999	0.00571
4	0.00305	4	0.00382	4	0.00452	4	0.00514	4	0.00571
4.001	0.00305	4.001	0.00382	4.001	0.00451	4.001	0.00514	4.001	0.00571
4.002	0.00305	4.002	0.00382	4.002	0.00451	4.002	0.00514	4.002	0.00571
4.003	0.00305	4.003	0.00382	4.003	0.00451	4.003	0.00513	4.003	0.0057
4.004	0.00305	4.004	0.00382	4.004	0.00451	4.004	0.00513	4.004	0.0057
4.005	0.00304	4.005	0.00382	4.005	0.00451	4.005	0.00513	4.005	0.0057
4.006	0.00304	4.006	0.00381	4.006	0.0045	4.006	0.00513	4.006	0.00569
4.007	0.00304	4.007	0.00381	4.007	0.0045	4.007	0.00512	4.007	0.00569
4.008	0.00304	4.008	0.00381	4.008	0.0045	4.008	0.00512	4.008	0.00569
4.009	0.00304	4.009	0.00381	4.009	0.0045	4.009	0.00512	4.009	0.00569
4.01	0.00304	4.01	0.00381	4.01	0.0045	4.01	0.00512	4.01	0.00568
4.011	0.00304	4.011	0.00381	4.011	0.00449	4.011	0.00511	4.011	0.00568
4.012	0.00304	4.012	0.0038	4.012	0.00449	4.012	0.00511	4.012	0.00568
4.013	0.00303	4.013	0.0038	4.013	0.00449	4.013	0.00511	4.013	0.00568
4.014	0.00303	4.014	0.0038	4.014	0.00449	4.014	0.00511	4.014	0.00567
4.015	0.00303	4.015	0.0038	4.015	0.00449	4.015	0.0051	4.015	0.00567
4.016	0.00303	4.016	0.0038	4.016	0.00448	4.016	0.0051	4.016	0.00567
4.017	0.00303	4.017	0.0038	4.017	0.00448	4.017	0.0051	4.017	0.00566
4.018	0.00303	4.018	0.00379	4.018	0.00448	4.018	0.0051	4.018	0.00566
4.019	0.00303	4.019	0.00379	4.019	0.00448	4.019	0.0051	4.019	0.00566
4.02	0.00303	4.02	0.00379	4.02	0.00447	4.02	0.00509	4.02	0.00566
4.021	0.00302	4.021	0.00379	4.021	0.00447	4.021	0.00509	4.021	0.00565
4.022	0.00302	4.022	0.00379	4.022	0.00447	4.022	0.00509	4.022	0.00565
4.023	0.00302	4.023	0.00379	4.023	0.00447	4.023	0.00509	4.023	0.00565
4.024	0.00302	4.024	0.00378	4.024	0.00447	4.024	0.00508	4.024	0.00565
4.025	0.00302	4.025	0.00378	4.025	0.00446	4.025	0.00508	4.025	0.00564
4.026	0.00302	4.026	0.00378	4.026	0.00446	4.026	0.00508	4.026	0.00564
4.027	0.00302	4.027	0.00378	4.027	0.00446	4.027	0.00508	4.027	0.00564
4.028	0.00302	4.028	0.00378	4.028	0.00446	4.028	0.00507	4.028	0.00563
4.029	0.00301	4.029	0.00378	4.029	0.00446	4.029	0.00507	4.029	0.00563
4.03	0.00301	4.03	0.00377	4.03	0.00445	4.03	0.00507	4.03	0.00563
4.031	0.00301	4.031	0.00377	4.031	0.00445	4.031	0.00507	4.031	0.00563
4.032	0.00301	4.032	0.00377	4.032	0.00445	4.032	0.00506	4.032	0.00562
4.033	0.00301	4.033	0.00377	4.033	0.00445	4.033	0.00506	4.033	0.00562
4.034	0.00301	4.034	0.00377	4.034	0.00445	4.034	0.00506	4.034	0.00562
4.035	0.00301	4.035	0.00377	4.035	0.00444	4.035	0.00506	4.035	0.00562
4.036	0.003	4.036	0.00376	4.036	0.00444	4.036	0.00505	4.036	0.00561
4.037	0.003	4.037	0.00376	4.037	0.00444	4.037	0.00505	4.037	0.00561
4.038	0.003	4.038	0.00376	4.038	0.00444	4.038	0.00505	4.038	0.00561
4.039	0.003	4.039	0.00376	4.039	0.00444	4.039	0.00505	4.039	0.00561
4.04	0.003	4.04	0.00376	4.04	0.00443	4.04	0.00505	4.04	0.0056
4.041	0.003	4.041	0.00376	4.041	0.00443	4.041	0.00504	4.041	0.0056
4.042	0.003	4.042	0.00375	4.042	0.00443	4.042	0.00504	4.042	0.0056
4.043	0.003	4.043	0.00375	4.043	0.00443	4.043	0.00504	4.043	0.00559
4.044	0.00299	4.044	0.00375	4.044	0.00443	4.044	0.00504	4.044	0.00559
4.045	0.00299	4.045	0.00375	4.045	0.00442	4.045	0.00503	4.045	0.00559
4.046	0.00299	4.046	0.00375	4.046	0.00442	4.046	0.00503	4.046	0.00559
4.047	0.00299	4.047	0.00375	4.047	0.00442	4.047	0.00503	4.047	0.00558
4.048	0.00299	4.048	0.00374	4.048	0.00442	4.048	0.00503	4.048	0.00558
4.049	0.00299	4.049	0.00374	4.049	0.00442	4.049	0.00502	4.049	0.00558
4.05	0.00299	4.05	0.00374	4.05	0.00441	4.05	0.00502	4.05	0.00558
4.051	0.00299	4.051	0.00374	4.051	0.00441	4.051	0.00502	4.051	0.00557
4.052	0.00298	4.052	0.00374	4.052	0.00441	4.052	0.00502	4.052	0.00557
4.053	0.00298	4.053	0.00374	4.053	0.00441	4.053	0.00501	4.053	0.00557
4.054	0.00298	4.054	0.00373	4.054	0.00441	4.054	0.00501	4.054	0.00557
4.055	0.00298	4.055	0.00373	4.055	0.0044	4.055	0.00501	4.055	0.00556
4.056	0.00298	4.056	0.00373	4.056	0.0044	4.056	0.00501	4.056	0.00556
4.057	0.00298	4.057	0.00373	4.057	0.0044	4.057	0.00501	4.057	0.00556
4.058	0.00298	4.058	0.00373	4.058	0.0044	4.058	0.005	4.058	0.00555
4.059	0.00298	4.059	0.00373	4.059	0.0044	4.059	0.005	4.059	0.00555
4.06	0.00297	4.06	0.00373	4.06	0.00439	4.06	0.005	4.06	0.00555
4.061	0.00297	4.061	0.00372	4.061	0.00439	4.061	0.005	4.061	0.00555
4.062	0.00297	4.062	0.00372	4.062	0.00439	4.062	0.00499	4.062	0.00554
4.063	0.00297	4.063	0.00372	4.063	0.00439	4.063	0.00499	4.063	0.00554
4.064	0.00297	4.064	0.00372	4.064	0.00439	4.064	0.00499	4.064	0.00554
4.065	0.00297	4.065	0.00372	4.065	0.00438	4.065	0.00499	4.065	0.00554
4.066	0.00297	4.066	0.00372	4.066	0.00438	4.066	0.00498	4.066	0.00553
4.067	0.00297	4.067	0.00371	4.067	0.00438	4.067	0.00498	4.067	0.00553
4.068	0.00296	4.068	0.00371	4.068	0.00438	4.068	0.00498	4.068	0.00553
4.069	0.00296	4.069	0.00371	4.069	0.00438	4.069	0.00498	4.069	0.00553
4.07	0.00296	4.07	0.00371	4.07	0.00437	4.07	0.00498	4.07	0.00552
4.071	0.00296	4.071	0.00371	4.071	0.00437	4.071	0.00497	4.071	0.00552
4.072	0.00296	4.072	0.00371	4.072	0.00437	4.072	0.00497	4.072	0.00552
4.073	0.00296	4.073	0.0037	4.073	0.00437	4.073	0.00497	4.073	0.00552
4.074	0.00296	4.074	0.0037	4.074	0.00437	4.074	0.00497	4.074	0.00551
4.075	0.00296	4.075	0.0037	4.075	0.00436	4.075	0.00496	4.075	0.00551
4.076	0.00296	4.076	0.0037	4.076	0.00436	4.076	0.00496	4.076	0.00551
4.077	0.00295	4.077	0.0037	4.077	0.00436	4.077	0.00496	4.077	0.0055

4.078	0.00295	4.078	0.0037	4.078	0.00436	4.078	0.00496	4.078	0.0055
4.079	0.00295	4.079	0.00369	4.079	0.00436	4.079	0.00495	4.079	0.0055
4.08	0.00295	4.08	0.00369	4.08	0.00435	4.08	0.00495	4.08	0.0055
4.081	0.00295	4.081	0.00369	4.081	0.00435	4.081	0.00495	4.081	0.00549
4.082	0.00295	4.082	0.00369	4.082	0.00435	4.082	0.00495	4.082	0.00549
4.083	0.00295	4.083	0.00369	4.083	0.00435	4.083	0.00495	4.083	0.00549
4.084	0.00295	4.084	0.00369	4.084	0.00435	4.084	0.00494	4.084	0.00549
4.085	0.00294	4.085	0.00368	4.085	0.00434	4.085	0.00494	4.085	0.00548
4.086	0.00294	4.086	0.00368	4.086	0.00434	4.086	0.00494	4.086	0.00548
4.087	0.00294	4.087	0.00368	4.087	0.00434	4.087	0.00494	4.087	0.00548
4.088	0.00294	4.088	0.00368	4.088	0.00434	4.088	0.00493	4.088	0.00548
4.089	0.00294	4.089	0.00368	4.089	0.00434	4.089	0.00493	4.089	0.00547
4.09	0.00294	4.09	0.00368	4.09	0.00434	4.09	0.00493	4.09	0.00547
4.091	0.00294	4.091	0.00368	4.091	0.00433	4.091	0.00493	4.091	0.00547
4.092	0.00294	4.092	0.00367	4.092	0.00433	4.092	0.00492	4.092	0.00547
4.093	0.00293	4.093	0.00367	4.093	0.00433	4.093	0.00492	4.093	0.00546
4.094	0.00293	4.094	0.00367	4.094	0.00433	4.094	0.00492	4.094	0.00546
4.095	0.00293	4.095	0.00367	4.095	0.00433	4.095	0.00492	4.095	0.00546
4.096	0.00293	4.096	0.00367	4.096	0.00432	4.096	0.00492	4.096	0.00546
4.097	0.00293	4.097	0.00367	4.097	0.00432	4.097	0.00491	4.097	0.00545
4.098	0.00293	4.098	0.00366	4.098	0.00432	4.098	0.00491	4.098	0.00545
4.099	0.00293	4.099	0.00366	4.099	0.00432	4.099	0.00491	4.099	0.00545
4.1	0.00293	4.1	0.00366	4.1	0.00432	4.1	0.00491	4.1	0.00545
4.101	0.00292	4.101	0.00366	4.101	0.00431	4.101	0.0049	4.101	0.00544
4.102	0.00292	4.102	0.00366	4.102	0.00431	4.102	0.0049	4.102	0.00544
4.103	0.00292	4.103	0.00366	4.103	0.00431	4.103	0.0049	4.103	0.00544
4.104	0.00292	4.104	0.00365	4.104	0.00431	4.104	0.0049	4.104	0.00544
4.105	0.00292	4.105	0.00365	4.105	0.00431	4.105	0.0049	4.105	0.00543
4.106	0.00292	4.106	0.00365	4.106	0.0043	4.106	0.00489	4.106	0.00543
4.107	0.00292	4.107	0.00365	4.107	0.0043	4.107	0.00489	4.107	0.00543
4.108	0.00292	4.108	0.00365	4.108	0.0043	4.108	0.00489	4.108	0.00542
4.109	0.00291	4.109	0.00365	4.109	0.0043	4.109	0.00489	4.109	0.00542
4.11	0.00291	4.11	0.00364	4.11	0.0043	4.11	0.00488	4.11	0.00542
4.111	0.00291	4.111	0.00364	4.111	0.00429	4.111	0.00488	4.111	0.00542
4.112	0.00291	4.112	0.00364	4.112	0.00429	4.112	0.00488	4.112	0.00541
4.113	0.00291	4.113	0.00364	4.113	0.00429	4.113	0.00488	4.113	0.00541
4.114	0.00291	4.114	0.00364	4.114	0.00429	4.114	0.00488	4.114	0.00541
4.115	0.00291	4.115	0.00364	4.115	0.00429	4.115	0.00487	4.115	0.00541
4.116	0.00291	4.116	0.00364	4.116	0.00429	4.116	0.00487	4.116	0.0054
4.117	0.00291	4.117	0.00363	4.117	0.00428	4.117	0.00487	4.117	0.0054
4.118	0.0029	4.118	0.00363	4.118	0.00428	4.118	0.00487	4.118	0.0054
4.119	0.0029	4.119	0.00363	4.119	0.00428	4.119	0.00486	4.119	0.0054
4.12	0.0029	4.12	0.00363	4.12	0.00428	4.12	0.00486	4.12	0.00539
4.121	0.0029	4.121	0.00363	4.121	0.00428	4.121	0.00486	4.121	0.00539
4.122	0.0029	4.122	0.00363	4.122	0.00427	4.122	0.00486	4.122	0.00539
4.123	0.0029	4.123	0.00362	4.123	0.00427	4.123	0.00486	4.123	0.00539
4.124	0.0029	4.124	0.00362	4.124	0.00427	4.124	0.00485	4.124	0.00538
4.125	0.0029	4.125	0.00362	4.125	0.00427	4.125	0.00485	4.125	0.00538
4.126	0.00289	4.126	0.00362	4.126	0.00427	4.126	0.00485	4.126	0.00538
4.127	0.00289	4.127	0.00362	4.127	0.00426	4.127	0.00485	4.127	0.00538
4.128	0.00289	4.128	0.00362	4.128	0.00426	4.128	0.00484	4.128	0.00537
4.129	0.00289	4.129	0.00362	4.129	0.00426	4.129	0.00484	4.129	0.00537
4.13	0.00289	4.13	0.00361	4.13	0.00426	4.13	0.00484	4.13	0.00537
4.131	0.00289	4.131	0.00361	4.131	0.00426	4.131	0.00484	4.131	0.00537
4.132	0.00289	4.132	0.00361	4.132	0.00425	4.132	0.00484	4.132	0.00536
4.133	0.00289	4.133	0.00361	4.133	0.00425	4.133	0.00483	4.133	0.00536
4.134	0.00289	4.134	0.00361	4.134	0.00425	4.134	0.00483	4.134	0.00536
4.135	0.00289	4.135	0.00361	4.135	0.00425	4.135	0.00483	4.135	0.00536
4.136	0.00288	4.136	0.0036	4.136	0.00425	4.136	0.00483	4.136	0.00535
4.137	0.00288	4.137	0.0036	4.137	0.00425	4.137	0.00482	4.137	0.00535
4.138	0.00288	4.138	0.0036	4.138	0.00424	4.138	0.00482	4.138	0.00535
4.139	0.00288	4.139	0.0036	4.139	0.00424	4.139	0.00482	4.139	0.00535
4.14	0.00288	4.14	0.0036	4.14	0.00424	4.14	0.00482	4.14	0.00534
4.141	0.00288	4.141	0.0036	4.141	0.00424	4.141	0.00482	4.141	0.00534
4.142	0.00288	4.142	0.0036	4.142	0.00424	4.142	0.00481	4.142	0.00534
4.143	0.00288	4.143	0.00359	4.143	0.00423	4.143	0.00481	4.143	0.00534
4.144	0.00288	4.144	0.00359	4.144	0.00423	4.144	0.00481	4.144	0.00533
4.145	0.00288	4.145	0.00359	4.145	0.00423	4.145	0.00481	4.145	0.00533
4.146	0.00288	4.146	0.00359	4.146	0.00423	4.146	0.0048	4.146	0.00533
4.147	0.00288	4.147	0.00359	4.147	0.00423	4.147	0.0048	4.147	0.00533
4.148	0.00288	4.148	0.00359	4.148	0.00422	4.148	0.0048	4.148	0.00532
4.149	0.00288	4.149	0.00358	4.149	0.00422	4.149	0.0048	4.149	0.00532
4.15	0.00287	4.15	0.00358	4.15	0.00422	4.15	0.0048	4.15	0.00532
4.151	0.00287	4.151	0.00358	4.151	0.00422	4.151	0.00479	4.151	0.00532
4.152	0.00287	4.152	0.00358	4.152	0.00422	4.152	0.00479	4.152	0.00531
4.153	0.00287	4.153	0.00358	4.153	0.00422	4.153	0.00479	4.153	0.00531
4.154	0.00287	4.154	0.00358	4.154	0.00421	4.154	0.00479	4.154	0.00531
4.155	0.00287	4.155	0.00358	4.155	0.00421	4.155	0.00478	4.155	0.00531
4.156	0.00287	4.156	0.00357	4.156	0.00421	4.156	0.00478	4.156	0.0053
4.157	0.00287	4.157	0.00357	4.157	0.00421	4.157	0.00478	4.157	0.0053
4.158	0.00287	4.158	0.00357	4.158	0.00421	4.158	0.00478	4.158	0.0053
4.159	0.00287	4.159	0.00357	4.159	0.0042	4.159	0.00478	4.159	0.0053
4.16	0.00287	4.16	0.00357	4.16	0.0042	4.16	0.00477	4.16	0.00529
4.161	0.00287	4.161	0.00357	4.161	0.0042	4.161	0.00477	4.161	0.00529
4.162	0.00287	4.162	0.00356	4.162	0.0042	4.162	0.00477	4.162	0.00529
4.163	0.00287	4.163	0.00356	4.163	0.0042	4.163	0.00477	4.163	0.00529
4.164	0.00286	4.164	0.00356	4.164	0.00419	4.164	0.00477	4.164	0.00528
4.165	0.00286	4.165	0.00356	4.165	0.00419	4.165	0.00476	4.165	0.00528
4.166	0.00286	4.166	0.00356	4.166	0.00419	4.166	0.00476	4.166	0.00528
4.167	0.00286	4.167	0.00356	4.167	0.00419	4.167	0.00476	4.167	0.00528
4.168	0.00286	4.168	0.00356	4.168	0.00419	4.168	0.00476	4.168	0.00528
4.169	0.00286	4.169	0.00355	4.169	0.00419	4.169	0.00475	4.169	0.00527
4.17	0.00286	4.17	0.00355	4.17	0.00418	4.17	0.00475	4.17	0.00527
4.171	0.00286	4.171	0.00355	4.171	0.00418	4.171	0.00475	4.171	0.00527
4.172	0.00286	4.172	0.00355	4.172	0.00418	4.172	0.00475	4.172	0.00527
4.173	0.00286	4.173	0.00355	4.173	0.00418	4.173	0.00475	4.173	0.00526
4.174	0.00286	4.174	0.00355	4.174	0.00418	4.174	0.00474	4.174	0.00526
4.175	0.00286	4.175	0.00354	4.175	0.00417	4.175	0.00474	4.175	0.00526
4.176	0.00286	4.176	0.00354	4.176	0.00417	4.176	0.00474	4.176	0.00526
4.177	0.00286	4.177	0.00354	4.177	0.00417	4.177	0.00474	4.177	0.00525
4.178	0.00286	4.178	0.00354	4.178	0.00417	4.178	0.00474	4.178	0.00525
4.179	0.00285	4.179	0.00354	4.179	0.00417	4.179	0.00473	4.179	0.00525
4.18	0.00285	4.18	0.00354	4.18	0.00417	4.18	0.00473	4.18	0.00525
4.181	0.00285	4.181	0.00354	4.181	0.00416	4.181	0.00473	4.181	0.00524
4.182	0.00285	4.182	0.00353	4.182	0.00416	4.182	0.00473	4.182	0.00524
4.183	0.00285	4.183	0.00353	4.183	0.00416	4.183	0.00472	4.183	0.00524
4.184	0.00285	4.184	0.00353	4.184	0.00416	4.184	0.00472	4.184	0.00524
4.185	0.00285	4.185	0.00353	4.185	0.00416	4.185	0.00472	4.185	0.00523
4.186	0.00285	4.186	0.00353	4.186	0.00415	4.186	0.00472	4.186	0.00523
4.187	0.00285	4.187	0.00353	4.187	0.00415	4.187	0.00472	4.187	0.00523
4.188	0.00285	4.188	0.00353	4.188	0.00415	4.188	0.00471	4.188	0.00523
4.189	0.00285	4.189	0.00352	4.189	0.00415	4.189	0.00471	4.189	0.00522
4.19	0.00285	4.19	0.00352	4.19	0.00415	4.19	0.00471	4.19	0.00522
4.191	0.00285	4.191	0.00352	4.191	0.00415	4.191	0.00471	4.191	0.00522
4.192	0.00285	4.192	0.00352	4.192	0.00414	4.192	0.00471	4.192	0.00522
4.193	0.00285	4.193	0.00352	4.193	0.00414	4.193	0.0047	4.193	0.00521
4.194	0.00284	4.194	0.00352	4.194	0.00414	4.194	0.0047	4.194	0.00521
4.195	0.00284	4.195	0.00351	4.195	0.00414	4.195	0.0047	4.195	0.00521
4.196	0.00284	4.196	0.00351	4.196	0.00414	4.196	0.0047	4.196	0.00521
4.197	0.00284	4.197	0.00351	4.197	0.00413	4.197	0.00469	4.197	0.0052
4.198	0.00284	4.198	0.00351	4.198	0.00413	4.198	0.00469	4.198	0.0052
4.199	0.00284	4.199	0.00351	4.199	0.00413	4.199	0.00469	4.199	0.0052
4.2	0.00284	4.2	0.00351	4.2	0.00413	4.2	0.00469	4.2	0.0052
4.201	0.00284	4.201	0.00351	4.201	0.00413	4.201	0.00469	4.201	0.0052
4.202	0.00284	4.202	0.0035	4.202	0.00413	4.202	0.00468	4.202	0.00519
4.203	0.00284	4.203	0.0035	4.203	0.00412	4.203	0.00468	4.203	0.00519
4.204	0.00284	4.204	0.0035	4.204	0.00412	4.204	0.00468	4.204	0.00519
4.205	0.00284	4.205	0.0035	4.205	0.00412	4.205	0.00468	4.205	0.00519
4.206	0.00284	4.206	0.0035	4.206	0.00412	4.206	0.00468	4.206	0.00518
4.207	0.00284	4.207	0.0035	4.207	0.00412	4.207	0.00467	4.207	0.00518
4.208	0.00284	4.208	0.0035	4.208	0.00411	4.208	0.00467	4.208	0.00518
4.209	0.00283	4.209	0.00349	4.209	0.00411	4.209	0.00467	4.209	0.00518
4.21	0.00283	4.21	0.00349	4.21	0.00411	4.21	0.00467	4.21	0.00517
4.211	0.00283	4.211	0.00349	4.211	0.00411	4.211	0.00467	4.211	0.00517
4.212	0.00283	4.212	0.00349	4.212	0.00411	4.212	0.00466	4.212	0.00517

4.213	0.00283	4.213	0.00349	4.213	0.00411	4.213	0.00466	4.213	0.00517
4.214	0.00283	4.214	0.00349	4.214	0.0041	4.214	0.00466	4.214	0.00516
4.215	0.00283	4.215	0.00349	4.215	0.0041	4.215	0.00466	4.215	0.00516
4.216	0.00283	4.216	0.00348	4.216	0.0041	4.216	0.00465	4.216	0.00516
4.217	0.00283	4.217	0.00348	4.217	0.0041	4.217	0.00465	4.217	0.00516
4.218	0.00283	4.218	0.00348	4.218	0.0041	4.218	0.00465	4.218	0.00515
4.219	0.00283	4.219	0.00348	4.219	0.00409	4.219	0.00465	4.219	0.00515
4.22	0.00283	4.22	0.00348	4.22	0.00409	4.22	0.00465	4.22	0.00515
4.221	0.00283	4.221	0.00348	4.221	0.00409	4.221	0.00464	4.221	0.00515
4.222	0.00283	4.222	0.00348	4.222	0.00409	4.222	0.00464	4.222	0.00515
4.223	0.00282	4.223	0.00347	4.223	0.00409	4.223	0.00464	4.223	0.00514
4.224	0.00282	4.224	0.00347	4.224	0.00409	4.224	0.00464	4.224	0.00514
4.225	0.00282	4.225	0.00347	4.225	0.00408	4.225	0.00464	4.225	0.00514
4.226	0.00282	4.226	0.00347	4.226	0.00408	4.226	0.00463	4.226	0.00514
4.227	0.00282	4.227	0.00347	4.227	0.00408	4.227	0.00463	4.227	0.00513
4.228	0.00282	4.228	0.00347	4.228	0.00408	4.228	0.00463	4.228	0.00513
4.229	0.00282	4.229	0.00346	4.229	0.00408	4.229	0.00463	4.229	0.00513
4.23	0.00282	4.23	0.00346	4.23	0.00408	4.23	0.00463	4.23	0.00513
4.231	0.00282	4.231	0.00346	4.231	0.00407	4.231	0.00462	4.231	0.00512
4.232	0.00282	4.232	0.00346	4.232	0.00407	4.232	0.00462	4.232	0.00512
4.233	0.00282	4.233	0.00346	4.233	0.00407	4.233	0.00462	4.233	0.00512
4.234	0.00282	4.234	0.00346	4.234	0.00407	4.234	0.00462	4.234	0.00512
4.235	0.00282	4.235	0.00346	4.235	0.00407	4.235	0.00462	4.235	0.00512
4.236	0.00282	4.236	0.00345	4.236	0.00406	4.236	0.00461	4.236	0.00511
4.237	0.00282	4.237	0.00345	4.237	0.00406	4.237	0.00461	4.237	0.00511
4.238	0.00281	4.238	0.00345	4.238	0.00406	4.238	0.00461	4.238	0.00511
4.239	0.00281	4.239	0.00345	4.239	0.00406	4.239	0.00461	4.239	0.00511
4.24	0.00281	4.24	0.00345	4.24	0.00406	4.24	0.00461	4.24	0.0051
4.241	0.00281	4.241	0.00345	4.241	0.00406	4.241	0.0046	4.241	0.0051
4.242	0.00281	4.242	0.00345	4.242	0.00405	4.242	0.0046	4.242	0.0051
4.243	0.00281	4.243	0.00344	4.243	0.00405	4.243	0.0046	4.243	0.0051
4.244	0.00281	4.244	0.00344	4.244	0.00405	4.244	0.0046	4.244	0.00509
4.245	0.00281	4.245	0.00344	4.245	0.00405	4.245	0.00459	4.245	0.00509
4.246	0.00281	4.246	0.00344	4.246	0.00405	4.246	0.00459	4.246	0.00509
4.247	0.00281	4.247	0.00344	4.247	0.00405	4.247	0.00459	4.247	0.00509
4.248	0.00281	4.248	0.00344	4.248	0.00404	4.248	0.00459	4.248	0.00508
4.249	0.00281	4.249	0.00344	4.249	0.00404	4.249	0.00459	4.249	0.00508
4.25	0.00281	4.25	0.00343	4.25	0.00404	4.25	0.00458	4.25	0.00508
4.251	0.00281	4.251	0.00343	4.251	0.00404	4.251	0.00458	4.251	0.00508
4.252	0.00281	4.252	0.00343	4.252	0.00404	4.252	0.00458	4.252	0.00508
4.253	0.00281	4.253	0.00343	4.253	0.00403	4.253	0.00458	4.253	0.00507
4.254	0.0028	4.254	0.00343	4.254	0.00403	4.254	0.00458	4.254	0.00507
4.255	0.0028	4.255	0.00343	4.255	0.00403	4.255	0.00457	4.255	0.00507
4.256	0.0028	4.256	0.00343	4.256	0.00403	4.256	0.00457	4.256	0.00507
4.257	0.0028	4.257	0.00342	4.257	0.00403	4.257	0.00457	4.257	0.00506
4.258	0.0028	4.258	0.00342	4.258	0.00403	4.258	0.00457	4.258	0.00506
4.259	0.0028	4.259	0.00342	4.259	0.00402	4.259	0.00457	4.259	0.00506
4.26	0.0028	4.26	0.00342	4.26	0.00402	4.26	0.00456	4.26	0.00506
4.261	0.0028	4.261	0.00342	4.261	0.00402	4.261	0.00456	4.261	0.00505
4.262	0.0028	4.262	0.00342	4.262	0.00402	4.262	0.00456	4.262	0.00505
4.263	0.0028	4.263	0.00342	4.263	0.00402	4.263	0.00456	4.263	0.00505
4.264	0.0028	4.264	0.00341	4.264	0.00402	4.264	0.00456	4.264	0.00505
4.265	0.0028	4.265	0.00341	4.265	0.00401	4.265	0.00455	4.265	0.00505
4.266	0.0028	4.266	0.00341	4.266	0.00401	4.266	0.00455	4.266	0.00504
4.267	0.0028	4.267	0.00341	4.267	0.00401	4.267	0.00455	4.267	0.00504
4.268	0.0028	4.268	0.00341	4.268	0.00401	4.268	0.00455	4.268	0.00504
4.269	0.00279	4.269	0.00341	4.269	0.00401	4.269	0.00455	4.269	0.00504
4.27	0.00279	4.27	0.00341	4.27	0.00401	4.27	0.00454	4.27	0.00503
4.271	0.00279	4.271	0.0034	4.271	0.004	4.271	0.00454	4.271	0.00503
4.272	0.00279	4.272	0.0034	4.272	0.004	4.272	0.00454	4.272	0.00503
4.273	0.00279	4.273	0.0034	4.273	0.004	4.273	0.00454	4.273	0.00503
4.274	0.00279	4.274	0.0034	4.274	0.004	4.274	0.00454	4.274	0.00503
4.275	0.00279	4.275	0.0034	4.275	0.004	4.275	0.00453	4.275	0.00502
4.276	0.00279	4.276	0.0034	4.276	0.00399	4.276	0.00453	4.276	0.00502
4.277	0.00279	4.277	0.0034	4.277	0.00399	4.277	0.00453	4.277	0.00502
4.278	0.00279	4.278	0.00339	4.278	0.00399	4.278	0.00453	4.278	0.00502
4.279	0.00279	4.279	0.00339	4.279	0.00399	4.279	0.00453	4.279	0.00501
4.28	0.00279	4.28	0.00339	4.28	0.00399	4.28	0.00452	4.28	0.00501
4.281	0.00279	4.281	0.00339	4.281	0.00399	4.281	0.00452	4.281	0.00501
4.282	0.00279	4.282	0.00339	4.282	0.00398	4.282	0.00452	4.282	0.00501
4.283	0.00279	4.283	0.00339	4.283	0.00398	4.283	0.00452	4.283	0.005
4.284	0.00278	4.284	0.00339	4.284	0.00398	4.284	0.00452	4.284	0.005
4.285	0.00278	4.285	0.00338	4.285	0.00398	4.285	0.00451	4.285	0.005
4.286	0.00278	4.286	0.00338	4.286	0.00398	4.286	0.00451	4.286	0.005
4.287	0.00278	4.287	0.00338	4.287	0.00398	4.287	0.00451	4.287	0.005
4.288	0.00278	4.288	0.00338	4.288	0.00397	4.288	0.00451	4.288	0.00499
4.289	0.00278	4.289	0.00338	4.289	0.00397	4.289	0.00451	4.289	0.00499
4.29	0.00278	4.29	0.00338	4.29	0.00397	4.29	0.0045	4.29	0.00499
4.291	0.00278	4.291	0.00338	4.291	0.00397	4.291	0.0045	4.291	0.00499
4.292	0.00278	4.292	0.00337	4.292	0.00397	4.292	0.0045	4.292	0.00498
4.293	0.00278	4.293	0.00337	4.293	0.00397	4.293	0.0045	4.293	0.00498
4.294	0.00278	4.294	0.00337	4.294	0.00396	4.294	0.0045	4.294	0.00498
4.295	0.00278	4.295	0.00337	4.295	0.00396	4.295	0.00449	4.295	0.00498
4.296	0.00278	4.296	0.00337	4.296	0.00396	4.296	0.00449	4.296	0.00498
4.297	0.00278	4.297	0.00337	4.297	0.00396	4.297	0.00449	4.297	0.00497
4.298	0.00278	4.298	0.00337	4.298	0.00396	4.298	0.00449	4.298	0.00497
4.299	0.00278	4.299	0.00337	4.299	0.00396	4.299	0.00449	4.299	0.00497
4.3	0.00277	4.3	0.00336	4.3	0.00395	4.3	0.00448	4.3	0.00497
4.301	0.00277	4.301	0.00336	4.301	0.00395	4.301	0.00448	4.301	0.00496
4.302	0.00277	4.302	0.00336	4.302	0.00395	4.302	0.00448	4.302	0.00496
4.303	0.00277	4.303	0.00336	4.303	0.00395	4.303	0.00448	4.303	0.00496
4.304	0.00277	4.304	0.00336	4.304	0.00395	4.304	0.00448	4.304	0.00496
4.305	0.00277	4.305	0.00336	4.305	0.00395	4.305	0.00447	4.305	0.00496
4.306	0.00277	4.306	0.00336	4.306	0.00394	4.306	0.00447	4.306	0.00495
4.307	0.00277	4.307	0.00335	4.307	0.00394	4.307	0.00447	4.307	0.00495
4.308	0.00277	4.308	0.00335	4.308	0.00394	4.308	0.00447	4.308	0.00495
4.309	0.00277	4.309	0.00335	4.309	0.00394	4.309	0.00447	4.309	0.00495
4.31	0.00277	4.31	0.00335	4.31	0.00394	4.31	0.00446	4.31	0.00494
4.311	0.00277	4.311	0.00335	4.311	0.00394	4.311	0.00446	4.311	0.00494
4.312	0.00277	4.312	0.00335	4.312	0.00393	4.312	0.00446	4.312	0.00494
4.313	0.00277	4.313	0.00335	4.313	0.00393	4.313	0.00446	4.313	0.00494
4.314	0.00277	4.314	0.00334	4.314	0.00393	4.314	0.00446	4.314	0.00494
4.315	0.00276	4.315	0.00334	4.315	0.00393	4.315	0.00446	4.315	0.00493
4.316	0.00276	4.316	0.00334	4.316	0.00393	4.316	0.00445	4.316	0.00493
4.317	0.00276	4.317	0.00334	4.317	0.00393	4.317	0.00445	4.317	0.00493
4.318	0.00276	4.318	0.00334	4.318	0.00392	4.318	0.00445	4.318	0.00493
4.319	0.00276	4.319	0.00334	4.319	0.00392	4.319	0.00445	4.319	0.00492
4.32	0.00276	4.32	0.00334	4.32	0.00392	4.32	0.00445	4.32	0.00492
4.321	0.00276	4.321	0.00333	4.321	0.00392	4.321	0.00444	4.321	0.00492
4.322	0.00276	4.322	0.00333	4.322	0.00392	4.322	0.00444	4.322	0.00492
4.323	0.00276	4.323	0.00333	4.323	0.00392	4.323	0.00444	4.323	0.00492
4.324	0.00276	4.324	0.00333	4.324	0.00391	4.324	0.00444	4.324	0.00491
4.325	0.00276	4.325	0.00333	4.325	0.00391	4.325	0.00444	4.325	0.00491
4.326	0.00276	4.326	0.00333	4.326	0.00391	4.326	0.00443	4.326	0.00491
4.327	0.00276	4.327	0.00333	4.327	0.00391	4.327	0.00443	4.327	0.00491
4.328	0.00276	4.328	0.00333	4.328	0.00391	4.328	0.00443	4.328	0.0049
4.329	0.00276	4.329	0.00332	4.329	0.00391	4.329	0.00443	4.329	0.0049
4.33	0.00276	4.33	0.00332	4.33	0.0039	4.33	0.00443	4.33	0.0049
4.331	0.00275	4.331	0.00332	4.331	0.0039	4.331	0.00442	4.331	0.0049
4.332	0.00275	4.332	0.00332	4.332	0.0039	4.332	0.00442	4.332	0.0049
4.333	0.00275	4.333	0.00332	4.333	0.0039	4.333	0.00442	4.333	0.00489
4.334	0.00275	4.334	0.00332	4.334	0.0039	4.334	0.00442	4.334	0.00489
4.335	0.00275	4.335	0.00332	4.335	0.0039	4.335	0.00442	4.335	0.00489
4.336	0.00275	4.336	0.00331	4.336	0.00389	4.336	0.00441	4.336	0.00489
4.337	0.00275	4.337	0.00331	4.337	0.00389	4.337	0.00441	4.337	0.00489
4.338	0.00275	4.338	0.00331	4.338	0.00389	4.338	0.00441	4.338	0.00488
4.339	0.00275	4.339	0.00331	4.339	0.00389	4.339	0.00441	4.339	0.00488
4.34	0.00275	4.34	0.00331	4.34	0.00389	4.34	0.00441	4.34	0.00488
4.341	0.00275	4.341	0.00331	4.341	0.00389	4.341	0.0044	4.341	0.00488
4.342	0.00275	4.342	0.00331	4.342	0.00388	4.342	0.0044	4.342	0.00487
4.343	0.00275	4.343	0.0033	4.343	0.00388	4.343	0.0044	4.343	0.00487
4.344	0.00275	4.344	0.0033	4.344	0.00388	4.344	0.0044	4.344	0.00487
4.345	0.00275	4.345	0.0033	4.345	0.00388	4.345	0.0044	4.345	0.00487
4.346	0.00274	4.346	0.0033	4.346	0.00388	4.346	0.0044	4.346	0.00487
4.347	0.00274	4.347	0.0033	4.347	0.00388	4.347	0.00439	4.347	0.00486

4.348	0.00274	4.348	0.0033	4.348	0.00387	4.348	0.00439	4.348	0.00486
4.349	0.00274	4.349	0.0033	4.349	0.00387	4.349	0.00439	4.349	0.00486
4.35	0.00274	4.35	0.0033	4.35	0.00387	4.35	0.00439	4.35	0.00486
4.351	0.00274	4.351	0.00329	4.351	0.00387	4.351	0.00439	4.351	0.00485
4.352	0.00274	4.352	0.00329	4.352	0.00387	4.352	0.00438	4.352	0.00485
4.353	0.00274	4.353	0.00329	4.353	0.00387	4.353	0.00438	4.353	0.00485
4.354	0.00274	4.354	0.00329	4.354	0.00386	4.354	0.00438	4.354	0.00485
4.355	0.00274	4.355	0.00329	4.355	0.00386	4.355	0.00438	4.355	0.00485
4.356	0.00274	4.356	0.00329	4.356	0.00386	4.356	0.00438	4.356	0.00484
4.357	0.00274	4.357	0.00329	4.357	0.00386	4.357	0.00437	4.357	0.00484
4.358	0.00274	4.358	0.00328	4.358	0.00386	4.358	0.00437	4.358	0.00484
4.359	0.00274	4.359	0.00328	4.359	0.00386	4.359	0.00437	4.359	0.00484
4.36	0.00274	4.36	0.00328	4.36	0.00385	4.36	0.00437	4.36	0.00484
4.361	0.00274	4.361	0.00328	4.361	0.00385	4.361	0.00437	4.361	0.00483
4.362	0.00273	4.362	0.00328	4.362	0.00385	4.362	0.00436	4.362	0.00483
4.363	0.00273	4.363	0.00328	4.363	0.00385	4.363	0.00436	4.363	0.00483
4.364	0.00273	4.364	0.00328	4.364	0.00385	4.364	0.00436	4.364	0.00483
4.365	0.00273	4.365	0.00328	4.365	0.00385	4.365	0.00436	4.365	0.00482
4.366	0.00273	4.366	0.00327	4.366	0.00384	4.366	0.00436	4.366	0.00482
4.367	0.00273	4.367	0.00327	4.367	0.00384	4.367	0.00436	4.367	0.00482
4.368	0.00273	4.368	0.00327	4.368	0.00384	4.368	0.00435	4.368	0.00482
4.369	0.00273	4.369	0.00327	4.369	0.00384	4.369	0.00435	4.369	0.00482
4.37	0.00273	4.37	0.00327	4.37	0.00384	4.37	0.00435	4.37	0.00481
4.371	0.00273	4.371	0.00327	4.371	0.00384	4.371	0.00435	4.371	0.00481
4.372	0.00273	4.372	0.00327	4.372	0.00384	4.372	0.00435	4.372	0.00481
4.373	0.00273	4.373	0.00326	4.373	0.00383	4.373	0.00434	4.373	0.00481
4.374	0.00273	4.374	0.00326	4.374	0.00383	4.374	0.00434	4.374	0.00481
4.375	0.00273	4.375	0.00326	4.375	0.00383	4.375	0.00434	4.375	0.0048
4.376	0.00273	4.376	0.00326	4.376	0.00383	4.376	0.00434	4.376	0.0048
4.377	0.00273	4.377	0.00326	4.377	0.00383	4.377	0.00434	4.377	0.0048
4.378	0.00272	4.378	0.00326	4.378	0.00383	4.378	0.00433	4.378	0.0048
4.379	0.00272	4.379	0.00326	4.379	0.00382	4.379	0.00433	4.379	0.0048
4.38	0.00272	4.38	0.00326	4.38	0.00382	4.38	0.00433	4.38	0.00479
4.381	0.00272	4.381	0.00325	4.381	0.00382	4.381	0.00433	4.381	0.00479
4.382	0.00272	4.382	0.00325	4.382	0.00382	4.382	0.00433	4.382	0.00479
4.383	0.00272	4.383	0.00325	4.383	0.00382	4.383	0.00433	4.383	0.00479
4.384	0.00272	4.384	0.00325	4.384	0.00382	4.384	0.00432	4.384	0.00478
4.385	0.00272	4.385	0.00325	4.385	0.00381	4.385	0.00432	4.385	0.00478
4.386	0.00272	4.386	0.00325	4.386	0.00381	4.386	0.00432	4.386	0.00478
4.387	0.00272	4.387	0.00325	4.387	0.00381	4.387	0.00432	4.387	0.00478
4.388	0.00272	4.388	0.00324	4.388	0.00381	4.388	0.00432	4.388	0.00478
4.389	0.00272	4.389	0.00324	4.389	0.00381	4.389	0.00431	4.389	0.00477
4.39	0.00272	4.39	0.00324	4.39	0.00381	4.39	0.00431	4.39	0.00477
4.391	0.00272	4.391	0.00324	4.391	0.0038	4.391	0.00431	4.391	0.00477
4.392	0.00272	4.392	0.00324	4.392	0.0038	4.392	0.00431	4.392	0.00477
4.393	0.00272	4.393	0.00324	4.393	0.0038	4.393	0.00431	4.393	0.00477
4.394	0.00272	4.394	0.00324	4.394	0.0038	4.394	0.00431	4.394	0.00476
4.395	0.00271	4.395	0.00324	4.395	0.0038	4.395	0.0043	4.395	0.00476
4.396	0.00271	4.396	0.00323	4.396	0.0038	4.396	0.0043	4.396	0.00476
4.397	0.00271	4.397	0.00323	4.397	0.0038	4.397	0.0043	4.397	0.00476
4.398	0.00271	4.398	0.00323	4.398	0.00379	4.398	0.0043	4.398	0.00476
4.399	0.00271	4.399	0.00323	4.399	0.00379	4.399	0.0043	4.399	0.00475
4.4	0.00271	4.4	0.00323	4.4	0.00379	4.4	0.00429	4.4	0.00475
4.401	0.00271	4.401	0.00323	4.401	0.00379	4.401	0.00429	4.401	0.00475
4.402	0.00271	4.402	0.00323	4.402	0.00379	4.402	0.00429	4.402	0.00475
4.403	0.00271	4.403	0.00323	4.403	0.00379	4.403	0.00429	4.403	0.00474
4.404	0.00271	4.404	0.00323	4.404	0.00378	4.404	0.00429	4.404	0.00474
4.405	0.00271	4.405	0.00323	4.405	0.00378	4.405	0.00428	4.405	0.00474
4.406	0.00271	4.406	0.00323	4.406	0.00378	4.406	0.00428	4.406	0.00474
4.407	0.00271	4.407	0.00323	4.407	0.00378	4.407	0.00428	4.407	0.00474
4.408	0.00271	4.408	0.00323	4.408	0.00378	4.408	0.00428	4.408	0.00473
4.409	0.00271	4.409	0.00322	4.409	0.00378	4.409	0.00428	4.409	0.00473
4.41	0.00271	4.41	0.00322	4.41	0.00377	4.41	0.00428	4.41	0.00473
4.411	0.0027	4.411	0.00322	4.411	0.00377	4.411	0.00427	4.411	0.00473
4.412	0.0027	4.412	0.00322	4.412	0.00377	4.412	0.00427	4.412	0.00473
4.413	0.0027	4.413	0.00322	4.413	0.00377	4.413	0.00427	4.413	0.00472
4.414	0.0027	4.414	0.00322	4.414	0.00377	4.414	0.00427	4.414	0.00472
4.415	0.0027	4.415	0.00322	4.415	0.00377	4.415	0.00427	4.415	0.00472
4.416	0.0027	4.416	0.00322	4.416	0.00377	4.416	0.00426	4.416	0.00472
4.417	0.0027	4.417	0.00322	4.417	0.00376	4.417	0.00426	4.417	0.00472
4.418	0.0027	4.418	0.00322	4.418	0.00376	4.418	0.00426	4.418	0.00471
4.419	0.0027	4.419	0.00322	4.419	0.00376	4.419	0.00426	4.419	0.00471
4.42	0.0027	4.42	0.00322	4.42	0.00376	4.42	0.00426	4.42	0.00471
4.421	0.0027	4.421	0.00322	4.421	0.00376	4.421	0.00426	4.421	0.00471
4.422	0.0027	4.422	0.00321	4.422	0.00376	4.422	0.00425	4.422	0.00471
4.423	0.0027	4.423	0.00321	4.423	0.00375	4.423	0.00425	4.423	0.0047
4.424	0.0027	4.424	0.00321	4.424	0.00375	4.424	0.00425	4.424	0.0047
4.425	0.0027	4.425	0.00321	4.425	0.00375	4.425	0.00425	4.425	0.0047
4.426	0.0027	4.426	0.00321	4.426	0.00375	4.426	0.00425	4.426	0.0047
4.427	0.00269	4.427	0.00321	4.427	0.00375	4.427	0.00424	4.427	0.0047
4.428	0.00269	4.428	0.00321	4.428	0.00375	4.428	0.00424	4.428	0.00469
4.429	0.00269	4.429	0.00321	4.429	0.00374	4.429	0.00424	4.429	0.00469
4.43	0.00269	4.43	0.00321	4.43	0.00374	4.43	0.00424	4.43	0.00469
4.431	0.00269	4.431	0.00321	4.431	0.00374	4.431	0.00424	4.431	0.00469
4.432	0.00269	4.432	0.00321	4.432	0.00374	4.432	0.00424	4.432	0.00469
4.433	0.00269	4.433	0.00321	4.433	0.00374	4.433	0.00423	4.433	0.00468
4.434	0.00269	4.434	0.00321	4.434	0.00374	4.434	0.00423	4.434	0.00468
4.435	0.00269	4.435	0.00321	4.435	0.00374	4.435	0.00423	4.435	0.00468
4.436	0.00269	4.436	0.0032	4.436	0.00373	4.436	0.00423	4.436	0.00468
4.437	0.00269	4.437	0.0032	4.437	0.00373	4.437	0.00423	4.437	0.00467
4.438	0.00269	4.438	0.0032	4.438	0.00373	4.438	0.00422	4.438	0.00467
4.439	0.00269	4.439	0.0032	4.439	0.00373	4.439	0.00422	4.439	0.00467
4.44	0.00269	4.44	0.0032	4.44	0.00373	4.44	0.00422	4.44	0.00467
4.441	0.00269	4.441	0.0032	4.441	0.00373	4.441	0.00422	4.441	0.00467
4.442	0.00269	4.442	0.0032	4.442	0.00372	4.442	0.00422	4.442	0.00466
4.443	0.00269	4.443	0.0032	4.443	0.00372	4.443	0.00422	4.443	0.00466
4.444	0.00268	4.444	0.0032	4.444	0.00372	4.444	0.00421	4.444	0.00466
4.445	0.00268	4.445	0.0032	4.445	0.00372	4.445	0.00421	4.445	0.00466
4.446	0.00268	4.446	0.0032	4.446	0.00372	4.446	0.00421	4.446	0.00466
4.447	0.00268	4.447	0.0032	4.447	0.00372	4.447	0.00421	4.447	0.00465
4.448	0.00268	4.448	0.0032	4.448	0.00372	4.448	0.00421	4.448	0.00465
4.449	0.00268	4.449	0.0032	4.449	0.00371	4.449	0.00421	4.449	0.00465
4.45	0.00268	4.45	0.00319	4.45	0.00371	4.45	0.0042	4.45	0.00465
4.451	0.00268	4.451	0.00319	4.451	0.00371	4.451	0.0042	4.451	0.00465
4.452	0.00268	4.452	0.00319	4.452	0.00371	4.452	0.0042	4.452	0.00464
4.453	0.00268	4.453	0.00319	4.453	0.00371	4.453	0.0042	4.453	0.00464
4.454	0.00268	4.454	0.00319	4.454	0.00371	4.454	0.0042	4.454	0.00464
4.455	0.00268	4.455	0.00319	4.455	0.0037	4.455	0.00419	4.455	0.00464
4.456	0.00268	4.456	0.00319	4.456	0.0037	4.456	0.00419	4.456	0.00464
4.457	0.00268	4.457	0.00319	4.457	0.0037	4.457	0.00419	4.457	0.00463
4.458	0.00268	4.458	0.00319	4.458	0.0037	4.458	0.00419	4.458	0.00463
4.459	0.00268	4.459	0.00319	4.459	0.0037	4.459	0.00419	4.459	0.00463
4.46	0.00267	4.46	0.00319	4.46	0.0037	4.46	0.00419	4.46	0.00463
4.461	0.00267	4.461	0.00319	4.461	0.0037	4.461	0.00418	4.461	0.00463
4.462	0.00267	4.462	0.00319	4.462	0.00369	4.462	0.00418	4.462	0.00462
4.463	0.00267	4.463	0.00319	4.463	0.00369	4.463	0.00418	4.463	0.00462
4.464	0.00267	4.464	0.00318	4.464	0.00369	4.464	0.00418	4.464	0.00462
4.465	0.00267	4.465	0.00318	4.465	0.00369	4.465	0.00418	4.465	0.00462
4.466	0.00267	4.466	0.00318	4.466	0.00369	4.466	0.00417	4.466	0.00462
4.467	0.00267	4.467	0.00318	4.467	0.00369	4.467	0.00417	4.467	0.00461
4.468	0.00267	4.468	0.00318	4.468	0.00369	4.468	0.00417	4.468	0.00461
4.469	0.00267	4.469	0.00318	4.469	0.00368	4.469	0.00417	4.469	0.00461
4.47	0.00267	4.47	0.00318	4.47	0.00368	4.47	0.00417	4.47	0.00461
4.471	0.00267	4.471	0.00318	4.471	0.00368	4.471	0.00417	4.471	0.00461
4.472	0.00267	4.472	0.00318	4.472	0.00368	4.472	0.00416	4.472	0.0046
4.473	0.00267	4.473	0.00318	4.473	0.00368	4.473	0.00416	4.473	0.0046
4.474	0.00267	4.474	0.00318	4.474	0.00368	4.474	0.00416	4.474	0.0046
4.475	0.00267	4.475	0.00318	4.475	0.00367	4.475	0.00416	4.475	0.0046
4.476	0.00267	4.476	0.00318	4.476	0.00367	4.476	0.00416	4.476	0.0046
4.477	0.00266	4.477	0.00318	4.477	0.00367	4.477	0.00416	4.477	0.00459
4.478	0.00266	4.478	0.00317	4.478	0.00367	4.478	0.00415	4.478	0.00459
4.479	0.00266	4.479	0.00317	4.479	0.00367	4.479	0.00415	4.479	0.00459
4.48	0.00266	4.48	0.00317	4.48	0.00367	4.48	0.00415	4.48	0.00459
4.481	0.00266	4.481	0.00317	4.481	0.00367	4.481	0.00415	4.481	0.00459
4.482	0.00266	4.482	0.00317	4.482	0.00366	4.482	0.00415	4.482	0.00458

4.483	0.00266	4.483	0.00317	4.483	0.00366	4.483	0.00414	4.483	0.00458
4.484	0.00266	4.484	0.00317	4.484	0.00366	4.484	0.00414	4.484	0.00458
4.485	0.00266	4.485	0.00317	4.485	0.00366	4.485	0.00414	4.485	0.00458
4.486	0.00266	4.486	0.00317	4.486	0.00366	4.486	0.00414	4.486	0.00458
4.487	0.00266	4.487	0.00317	4.487	0.00366	4.487	0.00414	4.487	0.00457
4.488	0.00266	4.488	0.00317	4.488	0.00365	4.488	0.00414	4.488	0.00457
4.489	0.00266	4.489	0.00317	4.489	0.00365	4.489	0.00413	4.489	0.00457
4.49	0.00266	4.49	0.00317	4.49	0.00365	4.49	0.00413	4.49	0.00457
4.491	0.00266	4.491	0.00317	4.491	0.00365	4.491	0.00413	4.491	0.00457
4.492	0.00266	4.492	0.00316	4.492	0.00365	4.492	0.00413	4.492	0.00456
4.493	0.00266	4.493	0.00316	4.493	0.00365	4.493	0.00413	4.493	0.00456
4.494	0.00265	4.494	0.00316	4.494	0.00365	4.494	0.00413	4.494	0.00456
4.495	0.00265	4.495	0.00316	4.495	0.00364	4.495	0.00412	4.495	0.00456
4.496	0.00265	4.496	0.00316	4.496	0.00364	4.496	0.00412	4.496	0.00456
4.497	0.00265	4.497	0.00316	4.497	0.00364	4.497	0.00412	4.497	0.00456
4.498	0.00265	4.498	0.00316	4.498	0.00364	4.498	0.00412	4.498	0.00455
4.499	0.00265	4.499	0.00316	4.499	0.00364	4.499	0.00412	4.499	0.00455
4.5	0.00265	4.5	0.00316	4.5	0.00364	4.5	0.00412	4.5	0.00455
4.501	0.00265	4.501	0.00316	4.501	0.00364	4.501	0.00411	4.501	0.00455
4.502	0.00265	4.502	0.00316	4.502	0.00363	4.502	0.00411	4.502	0.00455
4.503	0.00265	4.503	0.00316	4.503	0.00363	4.503	0.00411	4.503	0.00454
4.504	0.00265	4.504	0.00316	4.504	0.00363	4.504	0.00411	4.504	0.00454
4.505	0.00265	4.505	0.00316	4.505	0.00363	4.505	0.00411	4.505	0.00454
4.506	0.00265	4.506	0.00316	4.506	0.00363	4.506	0.00411	4.506	0.00454
4.507	0.00265	4.507	0.00315	4.507	0.00363	4.507	0.0041	4.507	0.00454
4.508	0.00265	4.508	0.00315	4.508	0.00363	4.508	0.0041	4.508	0.00453
4.509	0.00265	4.509	0.00315	4.509	0.00362	4.509	0.0041	4.509	0.00453
4.51	0.00265	4.51	0.00315	4.51	0.00362	4.51	0.0041	4.51	0.00453
4.511	0.00264	4.511	0.00315	4.511	0.00362	4.511	0.0041	4.511	0.00453
4.512	0.00264	4.512	0.00315	4.512	0.00362	4.512	0.00409	4.512	0.00453
4.513	0.00264	4.513	0.00315	4.513	0.00362	4.513	0.00409	4.513	0.00452
4.514	0.00264	4.514	0.00315	4.514	0.00362	4.514	0.00409	4.514	0.00452
4.515	0.00264	4.515	0.00315	4.515	0.00361	4.515	0.00409	4.515	0.00452
4.516	0.00264	4.516	0.00315	4.516	0.00361	4.516	0.00409	4.516	0.00452
4.517	0.00264	4.517	0.00315	4.517	0.00361	4.517	0.00409	4.517	0.00452
4.518	0.00264	4.518	0.00315	4.518	0.00361	4.518	0.00408	4.518	0.00451
4.519	0.00264	4.519	0.00315	4.519	0.00361	4.519	0.00408	4.519	0.00451
4.52	0.00264	4.52	0.00315	4.52	0.00361	4.52	0.00408	4.52	0.00451
4.521	0.00264	4.521	0.00314	4.521	0.00361	4.521	0.00408	4.521	0.00451
4.522	0.00264	4.522	0.00314	4.522	0.0036	4.522	0.00408	4.522	0.00451
4.523	0.00264	4.523	0.00314	4.523	0.0036	4.523	0.00408	4.523	0.0045
4.524	0.00264	4.524	0.00314	4.524	0.0036	4.524	0.00407	4.524	0.0045
4.525	0.00264	4.525	0.00314	4.525	0.0036	4.525	0.00407	4.525	0.0045
4.526	0.00264	4.526	0.00314	4.526	0.0036	4.526	0.00407	4.526	0.0045
4.527	0.00264	4.527	0.00314	4.527	0.0036	4.527	0.00407	4.527	0.0045
4.528	0.00263	4.528	0.00314	4.528	0.0036	4.528	0.00407	4.528	0.0045
4.529	0.00263	4.529	0.00314	4.529	0.00359	4.529	0.00407	4.529	0.00449
4.53	0.00263	4.53	0.00314	4.53	0.00359	4.53	0.00406	4.53	0.00449
4.531	0.00263	4.531	0.00314	4.531	0.00359	4.531	0.00406	4.531	0.00449
4.532	0.00263	4.532	0.00314	4.532	0.00359	4.532	0.00406	4.532	0.00449
4.533	0.00263	4.533	0.00314	4.533	0.00359	4.533	0.00406	4.533	0.00449
4.534	0.00263	4.534	0.00314	4.534	0.00359	4.534	0.00406	4.534	0.00448
4.535	0.00263	4.535	0.00313	4.535	0.00359	4.535	0.00406	4.535	0.00448
4.536	0.00263	4.536	0.00313	4.536	0.00358	4.536	0.00405	4.536	0.00448
4.537	0.00263	4.537	0.00313	4.537	0.00358	4.537	0.00405	4.537	0.00448
4.538	0.00263	4.538	0.00313	4.538	0.00358	4.538	0.00405	4.538	0.00448
4.539	0.00263	4.539	0.00313	4.539	0.00358	4.539	0.00405	4.539	0.00447
4.54	0.00263	4.54	0.00313	4.54	0.00358	4.54	0.00405	4.54	0.00447
4.541	0.00263	4.541	0.00313	4.541	0.00358	4.541	0.00405	4.541	0.00447
4.542	0.00263	4.542	0.00313	4.542	0.00358	4.542	0.00404	4.542	0.00447
4.543	0.00263	4.543	0.00313	4.543	0.00357	4.543	0.00404	4.543	0.00447
4.544	0.00263	4.544	0.00313	4.544	0.00357	4.544	0.00404	4.544	0.00446
4.545	0.00262	4.545	0.00313	4.545	0.00357	4.545	0.00404	4.545	0.00446
4.546	0.00262	4.546	0.00313	4.546	0.00357	4.546	0.00404	4.546	0.00446
4.547	0.00262	4.547	0.00313	4.547	0.00357	4.547	0.00404	4.547	0.00446
4.548	0.00262	4.548	0.00313	4.548	0.00357	4.548	0.00403	4.548	0.00446
4.549	0.00262	4.549	0.00313	4.549	0.00357	4.549	0.00403	4.549	0.00446
4.55	0.00262	4.55	0.00312	4.55	0.00356	4.55	0.00403	4.55	0.00445
4.551	0.00262	4.551	0.00312	4.551	0.00356	4.551	0.00403	4.551	0.00445
4.552	0.00262	4.552	0.00312	4.552	0.00356	4.552	0.00403	4.552	0.00445
4.553	0.00262	4.553	0.00312	4.553	0.00356	4.553	0.00403	4.553	0.00445
4.554	0.00262	4.554	0.00312	4.554	0.00356	4.554	0.00402	4.554	0.00445
4.555	0.00262	4.555	0.00312	4.555	0.00356	4.555	0.00402	4.555	0.00444
4.556	0.00262	4.556	0.00312	4.556	0.00356	4.556	0.00402	4.556	0.00444
4.557	0.00262	4.557	0.00312	4.557	0.00355	4.557	0.00402	4.557	0.00444
4.558	0.00262	4.558	0.00312	4.558	0.00355	4.558	0.00402	4.558	0.00444
4.559	0.00262	4.559	0.00312	4.559	0.00355	4.559	0.00402	4.559	0.00444
4.56	0.00262	4.56	0.00312	4.56	0.00355	4.56	0.00401	4.56	0.00443
4.561	0.00262	4.561	0.00312	4.561	0.00355	4.561	0.00401	4.561	0.00443
4.562	0.00262	4.562	0.00312	4.562	0.00355	4.562	0.00401	4.562	0.00443
4.563	0.00261	4.563	0.00312	4.563	0.00355	4.563	0.00401	4.563	0.00443
4.564	0.00261	4.564	0.00311	4.564	0.00354	4.564	0.00401	4.564	0.00443
4.565	0.00261	4.565	0.00311	4.565	0.00354	4.565	0.00401	4.565	0.00443
4.566	0.00261	4.566	0.00311	4.566	0.00354	4.566	0.004	4.566	0.00442
4.567	0.00261	4.567	0.00311	4.567	0.00354	4.567	0.004	4.567	0.00442
4.568	0.00261	4.568	0.00311	4.568	0.00354	4.568	0.004	4.568	0.00442
4.569	0.00261	4.569	0.00311	4.569	0.00354	4.569	0.004	4.569	0.00442
4.57	0.00261	4.57	0.00311	4.57	0.00354	4.57	0.004	4.57	0.00442
4.571	0.00261	4.571	0.00311	4.571	0.00353	4.571	0.004	4.571	0.00441
4.572	0.00261	4.572	0.00311	4.572	0.00353	4.572	0.00399	4.572	0.00441
4.573	0.00261	4.573	0.00311	4.573	0.00353	4.573	0.00399	4.573	0.00441
4.574	0.00261	4.574	0.00311	4.574	0.00353	4.574	0.00399	4.574	0.00441
4.575	0.00261	4.575	0.00311	4.575	0.00353	4.575	0.00399	4.575	0.00441
4.576	0.00261	4.576	0.00311	4.576	0.00353	4.576	0.00399	4.576	0.0044
4.577	0.00261	4.577	0.00311	4.577	0.00353	4.577	0.00399	4.577	0.0044
4.578	0.00261	4.578	0.00311	4.578	0.00352	4.578	0.00398	4.578	0.0044
4.579	0.00261	4.579	0.0031	4.579	0.00352	4.579	0.00398	4.579	0.0044
4.58	0.0026	4.58	0.0031	4.58	0.00352	4.58	0.00398	4.58	0.0044
4.581	0.0026	4.581	0.0031	4.581	0.00352	4.581	0.00398	4.581	0.0044
4.582	0.0026	4.582	0.0031	4.582	0.00352	4.582	0.00398	4.582	0.00439
4.583	0.0026	4.583	0.0031	4.583	0.00352	4.583	0.00398	4.583	0.00439
4.584	0.0026	4.584	0.0031	4.584	0.00352	4.584	0.00397	4.584	0.00439
4.585	0.0026	4.585	0.0031	4.585	0.00351	4.585	0.00397	4.585	0.00439
4.586	0.0026	4.586	0.0031	4.586	0.00351	4.586	0.00397	4.586	0.00439
4.587	0.0026	4.587	0.0031	4.587	0.00351	4.587	0.00397	4.587	0.00438
4.588	0.0026	4.588	0.0031	4.588	0.00351	4.588	0.00397	4.588	0.00438
4.589	0.0026	4.589	0.0031	4.589	0.00351	4.589	0.00397	4.589	0.00438
4.59	0.0026	4.59	0.0031	4.59	0.00351	4.59	0.00396	4.59	0.00438
4.591	0.0026	4.591	0.0031	4.591	0.00351	4.591	0.00396	4.591	0.00438
4.592	0.0026	4.592	0.0031	4.592	0.0035	4.592	0.00396	4.592	0.00437
4.593	0.0026	4.593	0.0031	4.593	0.0035	4.593	0.00396	4.593	0.00437
4.594	0.0026	4.594	0.00309	4.594	0.0035	4.594	0.00396	4.594	0.00437
4.595	0.0026	4.595	0.00309	4.595	0.0035	4.595	0.00396	4.595	0.00437
4.596	0.0026	4.596	0.00309	4.596	0.0035	4.596	0.00395	4.596	0.00437
4.597	0.0026	4.597	0.00309	4.597	0.0035	4.597	0.00395	4.597	0.00437
4.598	0.00259	4.598	0.00309	4.598	0.0035	4.598	0.00395	4.598	0.00436
4.599	0.00259	4.599	0.00309	4.599	0.0035	4.599	0.00395	4.599	0.00436
4.6	0.00259	4.6	0.00309	4.6	0.0035	4.6	0.00395	4.6	0.00436
4.601	0.00259	4.601	0.00309	4.601	0.0035	4.601	0.00395	4.601	0.00436
4.602	0.00259	4.602	0.00309	4.602	0.0035	4.602	0.00394	4.602	0.00436
4.603	0.00259	4.603	0.00309	4.603	0.00349	4.603	0.00394	4.603	0.00435
4.604	0.00259	4.604	0.00309	4.604	0.00349	4.604	0.00394	4.604	0.00435
4.605	0.00259	4.605	0.00309	4.605	0.00349	4.605	0.00394	4.605	0.00435
4.606	0.00259	4.606	0.00309	4.606	0.00349	4.606	0.00394	4.606	0.00435
4.607	0.00259	4.607	0.00309	4.607	0.00349	4.607	0.00394	4.607	0.00435
4.608	0.00259	4.608	0.00309	4.608	0.00349	4.608	0.00394	4.608	0.00435
4.609	0.00259	4.609	0.00308	4.609	0.00349	4.609	0.00393	4.609	0.00434
4.61	0.00259	4.61	0.00308	4.61	0.00349	4.61	0.00393	4.61	0.00434
4.611	0.00259	4.611	0.00308	4.611	0.00349	4.611	0.00393	4.611	0.00434
4.612	0.00259	4.612	0.00308	4.612	0.00349	4.612	0.00393	4.612	0.00434
4.613	0.00259	4.613	0.00308	4.613	0.00349	4.613	0.00393	4.613	0.00434
4.614	0.00259	4.614	0.00308	4.614	0.00349	4.614	0.00393	4.614	0.00433
4.615	0.00258	4.615	0.00308	4.615	0.00349	4.615	0.00392	4.615	0.00433
4.616	0.00258	4.616	0.00308	4.616	0.00348	4.616	0.00392	4.616	0.00433
4.617	0.00258	4.617	0.00308	4.617	0.00348	4.617	0.00392	4.617	0.00433

4.618	0.00258	4.618	0.00308	4.618	0.00348	4.618	0.00392	4.618	0.00433
4.619	0.00258	4.619	0.00308	4.619	0.00348	4.619	0.00392	4.619	0.00433
4.62	0.00258	4.62	0.00308	4.62	0.00348	4.62	0.00392	4.62	0.00432
4.621	0.00258	4.621	0.00308	4.621	0.00348	4.621	0.00391	4.621	0.00432
4.622	0.00258	4.622	0.00308	4.622	0.00348	4.622	0.00391	4.622	0.00432
4.623	0.00258	4.623	0.00308	4.623	0.00348	4.623	0.00391	4.623	0.00432
4.624	0.00258	4.624	0.00307	4.624	0.00348	4.624	0.00391	4.624	0.00432
4.625	0.00258	4.625	0.00307	4.625	0.00348	4.625	0.00391	4.625	0.00431
4.626	0.00258	4.626	0.00307	4.626	0.00348	4.626	0.00391	4.626	0.00431
4.627	0.00258	4.627	0.00307	4.627	0.00348	4.627	0.0039	4.627	0.00431
4.628	0.00258	4.628	0.00307	4.628	0.00348	4.628	0.0039	4.628	0.00431
4.629	0.00258	4.629	0.00307	4.629	0.00347	4.629	0.0039	4.629	0.00431
4.63	0.00258	4.63	0.00307	4.63	0.00347	4.63	0.0039	4.63	0.00431
4.631	0.00258	4.631	0.00307	4.631	0.00347	4.631	0.0039	4.631	0.0043
4.632	0.00258	4.632	0.00307	4.632	0.00347	4.632	0.0039	4.632	0.0043
4.633	0.00257	4.633	0.00307	4.633	0.00347	4.633	0.0039	4.633	0.0043
4.634	0.00257	4.634	0.00307	4.634	0.00347	4.634	0.00389	4.634	0.0043
4.635	0.00257	4.635	0.00307	4.635	0.00347	4.635	0.00389	4.635	0.0043
4.636	0.00257	4.636	0.00307	4.636	0.00347	4.636	0.00389	4.636	0.0043
4.637	0.00257	4.637	0.00307	4.637	0.00347	4.637	0.00389	4.637	0.00429
4.638	0.00257	4.638	0.00307	4.638	0.00347	4.638	0.00389	4.638	0.00429
4.639	0.00257	4.639	0.00306	4.639	0.00347	4.639	0.00389	4.639	0.00429
4.64	0.00257	4.64	0.00306	4.64	0.00347	4.64	0.00388	4.64	0.00429
4.641	0.00257	4.641	0.00306	4.641	0.00347	4.641	0.00388	4.641	0.00429
4.642	0.00257	4.642	0.00306	4.642	0.00347	4.642	0.00388	4.642	0.00428
4.643	0.00257	4.643	0.00306	4.643	0.00346	4.643	0.00388	4.643	0.00428
4.644	0.00257	4.644	0.00306	4.644	0.00346	4.644	0.00388	4.644	0.00428
4.645	0.00257	4.645	0.00306	4.645	0.00346	4.645	0.00388	4.645	0.00428
4.646	0.00257	4.646	0.00306	4.646	0.00346	4.646	0.00387	4.646	0.00428
4.647	0.00257	4.647	0.00306	4.647	0.00346	4.647	0.00387	4.647	0.00428
4.648	0.00257	4.648	0.00306	4.648	0.00346	4.648	0.00387	4.648	0.00427
4.649	0.00257	4.649	0.00306	4.649	0.00346	4.649	0.00387	4.649	0.00427
4.65	0.00257	4.65	0.00306	4.65	0.00346	4.65	0.00387	4.65	0.00427
4.651	0.00256	4.651	0.00306	4.651	0.00346	4.651	0.00387	4.651	0.00427
4.652	0.00256	4.652	0.00306	4.652	0.00346	4.652	0.00387	4.652	0.00427
4.653	0.00256	4.653	0.00306	4.653	0.00346	4.653	0.00386	4.653	0.00426
4.654	0.00256	4.654	0.00305	4.654	0.00346	4.654	0.00386	4.654	0.00426
4.655	0.00256	4.655	0.00305	4.655	0.00346	4.655	0.00386	4.655	0.00426
4.656	0.00256	4.656	0.00305	4.656	0.00345	4.656	0.00386	4.656	0.00426
4.657	0.00256	4.657	0.00305	4.657	0.00345	4.657	0.00386	4.657	0.00426
4.658	0.00256	4.658	0.00305	4.658	0.00345	4.658	0.00386	4.658	0.00426
4.659	0.00256	4.659	0.00305	4.659	0.00345	4.659	0.00385	4.659	0.00425
4.66	0.00256	4.66	0.00305	4.66	0.00345	4.66	0.00385	4.66	0.00425
4.661	0.00256	4.661	0.00305	4.661	0.00345	4.661	0.00385	4.661	0.00425
4.662	0.00256	4.662	0.00305	4.662	0.00345	4.662	0.00385	4.662	0.00425
4.663	0.00256	4.663	0.00305	4.663	0.00345	4.663	0.00385	4.663	0.00425
4.664	0.00256	4.664	0.00305	4.664	0.00345	4.664	0.00385	4.664	0.00425
4.665	0.00256	4.665	0.00305	4.665	0.00345	4.665	0.00384	4.665	0.00424
4.666	0.00256	4.666	0.00305	4.666	0.00345	4.666	0.00384	4.666	0.00424
4.667	0.00256	4.667	0.00305	4.667	0.00345	4.667	0.00384	4.667	0.00424
4.668	0.00256	4.668	0.00305	4.668	0.00345	4.668	0.00384	4.668	0.00424
4.669	0.00256	4.669	0.00304	4.669	0.00345	4.669	0.00384	4.669	0.00424
4.67	0.00255	4.67	0.00304	4.67	0.00344	4.67	0.00384	4.67	0.00423
4.671	0.00255	4.671	0.00304	4.671	0.00344	4.671	0.00384	4.671	0.00423
4.672	0.00255	4.672	0.00304	4.672	0.00344	4.672	0.00383	4.672	0.00423
4.673	0.00255	4.673	0.00304	4.673	0.00344	4.673	0.00383	4.673	0.00423
4.674	0.00255	4.674	0.00304	4.674	0.00344	4.674	0.00383	4.674	0.00423
4.675	0.00255	4.675	0.00304	4.675	0.00344	4.675	0.00383	4.675	0.00423
4.676	0.00255	4.676	0.00304	4.676	0.00344	4.676	0.00383	4.676	0.00422
4.677	0.00255	4.677	0.00304	4.677	0.00344	4.677	0.00383	4.677	0.00422
4.678	0.00255	4.678	0.00304	4.678	0.00344	4.678	0.00382	4.678	0.00422
4.679	0.00255	4.679	0.00304	4.679	0.00344	4.679	0.00382	4.679	0.00422
4.68	0.00255	4.68	0.00304	4.68	0.00344	4.68	0.00382	4.68	0.00422
4.681	0.00255	4.681	0.00304	4.681	0.00344	4.681	0.00382	4.681	0.00422
4.682	0.00255	4.682	0.00304	4.682	0.00344	4.682	0.00382	4.682	0.00421
4.683	0.00255	4.683	0.00304	4.683	0.00343	4.683	0.00382	4.683	0.00421
4.684	0.00255	4.684	0.00304	4.684	0.00343	4.684	0.00382	4.684	0.00421
4.685	0.00255	4.685	0.00303	4.685	0.00343	4.685	0.00381	4.685	0.00421
4.686	0.00255	4.686	0.00303	4.686	0.00343	4.686	0.00381	4.686	0.00421
4.687	0.00255	4.687	0.00303	4.687	0.00343	4.687	0.00381	4.687	0.00421
4.688	0.00254	4.688	0.00303	4.688	0.00343	4.688	0.00381	4.688	0.0042
4.689	0.00254	4.689	0.00303	4.689	0.00343	4.689	0.00381	4.689	0.0042
4.69	0.00254	4.69	0.00303	4.69	0.00343	4.69	0.00381	4.69	0.0042
4.691	0.00254	4.691	0.00303	4.691	0.00343	4.691	0.0038	4.691	0.0042
4.692	0.00254	4.692	0.00303	4.692	0.00343	4.692	0.0038	4.692	0.0042
4.693	0.00254	4.693	0.00303	4.693	0.00343	4.693	0.0038	4.693	0.00419
4.694	0.00254	4.694	0.00303	4.694	0.00343	4.694	0.0038	4.694	0.00419
4.695	0.00254	4.695	0.00303	4.695	0.00343	4.695	0.0038	4.695	0.00419
4.696	0.00254	4.696	0.00303	4.696	0.00343	4.696	0.0038	4.696	0.00419
4.697	0.00254	4.697	0.00303	4.697	0.00342	4.697	0.0038	4.697	0.00419
4.698	0.00254	4.698	0.00303	4.698	0.00342	4.698	0.00379	4.698	0.00419
4.699	0.00254	4.699	0.00303	4.699	0.00342	4.699	0.00379	4.699	0.00418
4.7	0.00254	4.7	0.00302	4.7	0.00342	4.7	0.00379	4.7	0.00418
4.701	0.00254	4.701	0.00302	4.701	0.00342	4.701	0.00379	4.701	0.00418
4.702	0.00254	4.702	0.00302	4.702	0.00342	4.702	0.00379	4.702	0.00418
4.703	0.00254	4.703	0.00302	4.703	0.00342	4.703	0.00379	4.703	0.00418
4.704	0.00254	4.704	0.00302	4.704	0.00342	4.704	0.00378	4.704	0.00418
4.705	0.00254	4.705	0.00302	4.705	0.00342	4.705	0.00378	4.705	0.00417
4.706	0.00254	4.706	0.00302	4.706	0.00342	4.706	0.00378	4.706	0.00417
4.707	0.00253	4.707	0.00302	4.707	0.00342	4.707	0.00378	4.707	0.00417
4.708	0.00253	4.708	0.00302	4.708	0.00342	4.708	0.00378	4.708	0.00417
4.709	0.00253	4.709	0.00302	4.709	0.00342	4.709	0.00378	4.709	0.00417
4.71	0.00253	4.71	0.00302	4.71	0.00342	4.71	0.00378	4.71	0.00417
4.711	0.00253	4.711	0.00302	4.711	0.00341	4.711	0.00377	4.711	0.00416
4.712	0.00253	4.712	0.00302	4.712	0.00341	4.712	0.00377	4.712	0.00416
4.713	0.00253	4.713	0.00302	4.713	0.00341	4.713	0.00377	4.713	0.00416
4.714	0.00253	4.714	0.00302	4.714	0.00341	4.714	0.00377	4.714	0.00416
4.715	0.00253	4.715	0.00302	4.715	0.00341	4.715	0.00377	4.715	0.00416
4.716	0.00253	4.716	0.00301	4.716	0.00341	4.716	0.00377	4.716	0.00416
4.717	0.00253	4.717	0.00301	4.717	0.00341	4.717	0.00377	4.717	0.00415
4.718	0.00253	4.718	0.00301	4.718	0.00341	4.718	0.00376	4.718	0.00415
4.719	0.00253	4.719	0.00301	4.719	0.00341	4.719	0.00376	4.719	0.00415
4.72	0.00253	4.72	0.00301	4.72	0.00341	4.72	0.00376	4.72	0.00415
4.721	0.00253	4.721	0.00301	4.721	0.00341	4.721	0.00376	4.721	0.00415
4.722	0.00253	4.722	0.00301	4.722	0.00341	4.722	0.00376	4.722	0.00415
4.723	0.00253	4.723	0.00301	4.723	0.00341	4.723	0.00376	4.723	0.00414
4.724	0.00253	4.724	0.00301	4.724	0.0034	4.724	0.00375	4.724	0.00414
4.725	0.00252	4.725	0.00301	4.725	0.0034	4.725	0.00375	4.725	0.00414
4.726	0.00252	4.726	0.00301	4.726	0.0034	4.726	0.00375	4.726	0.00414
4.727	0.00252	4.727	0.00301	4.727	0.0034	4.727	0.00375	4.727	0.00414
4.728	0.00252	4.728	0.00301	4.728	0.0034	4.728	0.00375	4.728	0.00414
4.729	0.00252	4.729	0.00301	4.729	0.0034	4.729	0.00375	4.729	0.00413
4.73	0.00252	4.73	0.00301	4.73	0.0034	4.73	0.00375	4.73	0.00413
4.731	0.00252	4.731	0.003	4.731	0.0034	4.731	0.00374	4.731	0.00413
4.732	0.00252	4.732	0.003	4.732	0.0034	4.732	0.00374	4.732	0.00413
4.733	0.00252	4.733	0.003	4.733	0.0034	4.733	0.00374	4.733	0.00413
4.734	0.00252	4.734	0.003	4.734	0.0034	4.734	0.00374	4.734	0.00413
4.735	0.00252	4.735	0.003	4.735	0.0034	4.735	0.00374	4.735	0.00412
4.736	0.00252	4.736	0.003	4.736	0.0034	4.736	0.00374	4.736	0.00412
4.737	0.00252	4.737	0.003	4.737	0.0034	4.737	0.00374	4.737	0.00412
4.738	0.00252	4.738	0.003	4.738	0.00339	4.738	0.00373	4.738	0.00412
4.739	0.00252	4.739	0.003	4.739	0.00339	4.739	0.00373	4.739	0.00412
4.74	0.00252	4.74	0.003	4.74	0.00339	4.74	0.00373	4.74	0.00411
4.741	0.00252	4.741	0.003	4.741	0.00339	4.741	0.00373	4.741	0.00411
4.742	0.00252	4.742	0.003	4.742	0.00339	4.742	0.00373	4.742	0.00411
4.743	0.00252	4.743	0.003	4.743	0.00339	4.743	0.00373	4.743	0.00411
4.744	0.00251	4.744	0.003	4.744	0.00339	4.744	0.00372	4.744	0.00411
4.745	0.00251	4.745	0.003	4.745	0.00339	4.745	0.00372	4.745	0.00411
4.746	0.00251	4.746	0.003	4.746	0.00339	4.746	0.00372	4.746	0.0041
4.747	0.00251	4.747	0.00299	4.747	0.00339	4.747	0.00372	4.747	0.0041
4.748	0.00251	4.748	0.00299	4.748	0.00339	4.748	0.00372	4.748	0.0041
4.749	0.00251	4.749	0.00299	4.749	0.00339	4.749	0.00372	4.749	0.0041
4.75	0.00251	4.75	0.00299	4.75	0.00339	4.75	0.00372	4.75	0.0041
4.751	0.00251	4.751	0.00299	4.751	0.00339	4.751	0.00372	4.751	0.0041
4.752	0.00251	4.752	0.00299	4.752	0.00338	4.752	0.00371	4.752	0.00409

4.753	0.00251	4.753	0.00299	4.753	0.00338	4.753	0.00371	4.753	0.00409
4.754	0.00251	4.754	0.00299	4.754	0.00338	4.754	0.00371	4.754	0.00409
4.755	0.00251	4.755	0.00299	4.755	0.00338	4.755	0.00371	4.755	0.00409
4.756	0.00251	4.756	0.00299	4.756	0.00338	4.756	0.00371	4.756	0.00409
4.757	0.00251	4.757	0.00299	4.757	0.00338	4.757	0.00371	4.757	0.00409
4.758	0.00251	4.758	0.00299	4.758	0.00338	4.758	0.00371	4.758	0.00408
4.759	0.00251	4.759	0.00299	4.759	0.00338	4.759	0.00371	4.759	0.00408
4.76	0.00251	4.76	0.00299	4.76	0.00338	4.76	0.00371	4.76	0.00408
4.761	0.00251	4.761	0.00299	4.761	0.00338	4.761	0.00371	4.761	0.00408
4.762	0.00251	4.762	0.00299	4.762	0.00338	4.762	0.00371	4.762	0.00408
4.763	0.0025	4.763	0.00298	4.763	0.00338	4.763	0.00371	4.763	0.00408
4.764	0.0025	4.764	0.00298	4.764	0.00338	4.764	0.00371	4.764	0.00408
4.765	0.0025	4.765	0.00298	4.765	0.00338	4.765	0.0037	4.765	0.00407
4.766	0.0025	4.766	0.00298	4.766	0.00337	4.766	0.0037	4.766	0.00407
4.767	0.0025	4.767	0.00298	4.767	0.00337	4.767	0.0037	4.767	0.00407
4.768	0.0025	4.768	0.00298	4.768	0.00337	4.768	0.0037	4.768	0.00407
4.769	0.0025	4.769	0.00298	4.769	0.00337	4.769	0.0037	4.769	0.00407
4.77	0.0025	4.77	0.00298	4.77	0.00337	4.77	0.0037	4.77	0.00407
4.771	0.0025	4.771	0.00298	4.771	0.00337	4.771	0.0037	4.771	0.00406
4.772	0.0025	4.772	0.00298	4.772	0.00337	4.772	0.0037	4.772	0.00406
4.773	0.0025	4.773	0.00298	4.773	0.00337	4.773	0.0037	4.773	0.00406
4.774	0.0025	4.774	0.00298	4.774	0.00337	4.774	0.0037	4.774	0.00406
4.775	0.0025	4.775	0.00298	4.775	0.00337	4.775	0.0037	4.775	0.00406
4.776	0.0025	4.776	0.00298	4.776	0.00337	4.776	0.0037	4.776	0.00406
4.777	0.0025	4.777	0.00298	4.777	0.00337	4.777	0.0037	4.777	0.00405
4.778	0.0025	4.778	0.00298	4.778	0.00337	4.778	0.00369	4.778	0.00405
4.779	0.0025	4.779	0.00297	4.779	0.00337	4.779	0.00369	4.779	0.00405
4.78	0.0025	4.78	0.00297	4.78	0.00337	4.78	0.00369	4.78	0.00405
4.781	0.0025	4.781	0.00297	4.781	0.00336	4.781	0.00369	4.781	0.00405
4.782	0.00249	4.782	0.00297	4.782	0.00336	4.782	0.00369	4.782	0.00405
4.783	0.00249	4.783	0.00297	4.783	0.00336	4.783	0.00369	4.783	0.00404
4.784	0.00249	4.784	0.00297	4.784	0.00336	4.784	0.00369	4.784	0.00404
4.785	0.00249	4.785	0.00297	4.785	0.00336	4.785	0.00369	4.785	0.00404
4.786	0.00249	4.786	0.00297	4.786	0.00336	4.786	0.00369	4.786	0.00404
4.787	0.00249	4.787	0.00297	4.787	0.00336	4.787	0.00369	4.787	0.00404
4.788	0.00249	4.788	0.00297	4.788	0.00336	4.788	0.00369	4.788	0.00404
4.789	0.00249	4.789	0.00297	4.789	0.00336	4.789	0.00369	4.789	0.00403
4.79	0.00249	4.79	0.00297	4.79	0.00336	4.79	0.00369	4.79	0.00403
4.791	0.00249	4.791	0.00297	4.791	0.00336	4.791	0.00368	4.791	0.00403
4.792	0.00249	4.792	0.00297	4.792	0.00336	4.792	0.00368	4.792	0.00403
4.793	0.00249	4.793	0.00297	4.793	0.00336	4.793	0.00368	4.793	0.00403
4.794	0.00249	4.794	0.00297	4.794	0.00336	4.794	0.00368	4.794	0.00403
4.795	0.00249	4.795	0.00296	4.795	0.00335	4.795	0.00368	4.795	0.00402
4.796	0.00249	4.796	0.00296	4.796	0.00335	4.796	0.00368	4.796	0.00402
4.797	0.00249	4.797	0.00296	4.797	0.00335	4.797	0.00368	4.797	0.00402
4.798	0.00249	4.798	0.00296	4.798	0.00335	4.798	0.00368	4.798	0.00402
4.799	0.00249	4.799	0.00296	4.799	0.00335	4.799	0.00368	4.799	0.00402
4.8	0.00249	4.8	0.00296	4.8	0.00335	4.8	0.00368	4.8	0.00402
4.801	0.00248	4.801	0.00296	4.801	0.00335	4.801	0.00368	4.801	0.00401
4.802	0.00248	4.802	0.00296	4.802	0.00335	4.802	0.00368	4.802	0.00401
4.803	0.00248	4.803	0.00296	4.803	0.00335	4.803	0.00368	4.803	0.00401
4.804	0.00248	4.804	0.00296	4.804	0.00335	4.804	0.00367	4.804	0.00401
4.805	0.00248	4.805	0.00296	4.805	0.00335	4.805	0.00367	4.805	0.00401
4.806	0.00248	4.806	0.00296	4.806	0.00335	4.806	0.00367	4.806	0.00401
4.807	0.00248	4.807	0.00296	4.807	0.00335	4.807	0.00367	4.807	0.004
4.808	0.00248	4.808	0.00296	4.808	0.00335	4.808	0.00367	4.808	0.004
4.809	0.00248	4.809	0.00296	4.809	0.00334	4.809	0.00367	4.809	0.004
4.81	0.00248	4.81	0.00296	4.81	0.00334	4.81	0.00367	4.81	0.004
4.811	0.00248	4.811	0.00296	4.811	0.00334	4.811	0.00367	4.811	0.004
4.812	0.00248	4.812	0.00295	4.812	0.00334	4.812	0.00367	4.812	0.004
4.813	0.00248	4.813	0.00295	4.813	0.00334	4.813	0.00367	4.813	0.004
4.814	0.00248	4.814	0.00295	4.814	0.00334	4.814	0.00367	4.814	0.00399
4.815	0.00248	4.815	0.00295	4.815	0.00334	4.815	0.00367	4.815	0.00399
4.816	0.00248	4.816	0.00295	4.816	0.00334	4.816	0.00367	4.816	0.00399
4.817	0.00248	4.817	0.00295	4.817	0.00334	4.817	0.00366	4.817	0.00399
4.818	0.00248	4.818	0.00295	4.818	0.00334	4.818	0.00366	4.818	0.00399
4.819	0.00248	4.819	0.00295	4.819	0.00334	4.819	0.00366	4.819	0.00399
4.82	0.00248	4.82	0.00295	4.82	0.00334	4.82	0.00366	4.82	0.00398
4.821	0.00247	4.821	0.00295	4.821	0.00334	4.821	0.00366	4.821	0.00398
4.822	0.00247	4.822	0.00295	4.822	0.00334	4.822	0.00366	4.822	0.00398
4.823	0.00247	4.823	0.00295	4.823	0.00334	4.823	0.00366	4.823	0.00398
4.824	0.00247	4.824	0.00295	4.824	0.00333	4.824	0.00366	4.824	0.00398
4.825	0.00247	4.825	0.00295	4.825	0.00333	4.825	0.00366	4.825	0.00398
4.826	0.00247	4.826	0.00295	4.826	0.00333	4.826	0.00366	4.826	0.00397
4.827	0.00247	4.827	0.00295	4.827	0.00333	4.827	0.00366	4.827	0.00397
4.828	0.00247	4.828	0.00294	4.828	0.00333	4.828	0.00366	4.828	0.00397
4.829	0.00247	4.829	0.00294	4.829	0.00333	4.829	0.00366	4.829	0.00397
4.83	0.00247	4.83	0.00294	4.83	0.00333	4.83	0.00365	4.83	0.00397
4.831	0.00247	4.831	0.00294	4.831	0.00333	4.831	0.00365	4.831	0.00397
4.832	0.00247	4.832	0.00294	4.832	0.00333	4.832	0.00365	4.832	0.00397
4.833	0.00247	4.833	0.00294	4.833	0.00333	4.833	0.00365	4.833	0.00396
4.834	0.00247	4.834	0.00294	4.834	0.00333	4.834	0.00365	4.834	0.00396
4.835	0.00247	4.835	0.00294	4.835	0.00333	4.835	0.00365	4.835	0.00396
4.836	0.00247	4.836	0.00294	4.836	0.00333	4.836	0.00365	4.836	0.00396
4.837	0.00247	4.837	0.00294	4.837	0.00333	4.837	0.00365	4.837	0.00396
4.838	0.00247	4.838	0.00294	4.838	0.00332	4.838	0.00365	4.838	0.00396
4.839	0.00247	4.839	0.00294	4.839	0.00332	4.839	0.00365	4.839	0.00395
4.84	0.00246	4.84	0.00294	4.84	0.00332	4.84	0.00365	4.84	0.00395
4.841	0.00246	4.841	0.00294	4.841	0.00332	4.841	0.00365	4.841	0.00395
4.842	0.00246	4.842	0.00294	4.842	0.00332	4.842	0.00365	4.842	0.00395
4.843	0.00246	4.843	0.00294	4.843	0.00332	4.843	0.00364	4.843	0.00395
4.844	0.00246	4.844	0.00293	4.844	0.00332	4.844	0.00364	4.844	0.00395
4.845	0.00246	4.845	0.00293	4.845	0.00332	4.845	0.00364	4.845	0.00394
4.846	0.00246	4.846	0.00293	4.846	0.00332	4.846	0.00364	4.846	0.00394
4.847	0.00246	4.847	0.00293	4.847	0.00332	4.847	0.00364	4.847	0.00394
4.848	0.00246	4.848	0.00293	4.848	0.00332	4.848	0.00364	4.848	0.00394
4.849	0.00246	4.849	0.00293	4.849	0.00332	4.849	0.00364	4.849	0.00394
4.85	0.00246	4.85	0.00293	4.85	0.00332	4.85	0.00364	4.85	0.00394
4.851	0.00246	4.851	0.00293	4.851	0.00332	4.851	0.00364	4.851	0.00394
4.852	0.00246	4.852	0.00293	4.852	0.00332	4.852	0.00364	4.852	0.00393
4.853	0.00246	4.853	0.00293	4.853	0.00331	4.853	0.00364	4.853	0.00393
4.854	0.00246	4.854	0.00293	4.854	0.00331	4.854	0.00364	4.854	0.00393
4.855	0.00246	4.855	0.00293	4.855	0.00331	4.855	0.00364	4.855	0.00393
4.856	0.00246	4.856	0.00293	4.856	0.00331	4.856	0.00363	4.856	0.00393
4.857	0.00246	4.857	0.00293	4.857	0.00331	4.857	0.00363	4.857	0.00393
4.858	0.00246	4.858	0.00293	4.858	0.00331	4.858	0.00363	4.858	0.00392
4.859	0.00246	4.859	0.00293	4.859	0.00331	4.859	0.00363	4.859	0.00392
4.86	0.00245	4.86	0.00293	4.86	0.00331	4.86	0.00363	4.86	0.00392
4.861	0.00245	4.861	0.00292	4.861	0.00331	4.861	0.00363	4.861	0.00392
4.862	0.00245	4.862	0.00292	4.862	0.00331	4.862	0.00363	4.862	0.00392
4.863	0.00245	4.863	0.00292	4.863	0.00331	4.863	0.00363	4.863	0.00392
4.864	0.00245	4.864	0.00292	4.864	0.00331	4.864	0.00363	4.864	0.00391
4.865	0.00245	4.865	0.00292	4.865	0.00331	4.865	0.00363	4.865	0.00391
4.866	0.00245	4.866	0.00292	4.866	0.00331	4.866	0.00363	4.866	0.00391
4.867	0.00245	4.867	0.00292	4.867	0.0033	4.867	0.00363	4.867	0.00391
4.868	0.00245	4.868	0.00292	4.868	0.0033	4.868	0.00363	4.868	0.00391
4.869	0.00245	4.869	0.00292	4.869	0.0033	4.869	0.00363	4.869	0.00391
4.87	0.00245	4.87	0.00292	4.87	0.0033	4.87	0.00362	4.87	0.00391
4.871	0.00245	4.871	0.00292	4.871	0.0033	4.871	0.00362	4.871	0.0039
4.872	0.00245	4.872	0.00292	4.872	0.0033	4.872	0.00362	4.872	0.0039
4.873	0.00245	4.873	0.00292	4.873	0.0033	4.873	0.00362	4.873	0.0039
4.874	0.00245	4.874	0.00292	4.874	0.0033	4.874	0.00362	4.874	0.0039
4.875	0.00245	4.875	0.00292	4.875	0.0033	4.875	0.00362	4.875	0.0039
4.876	0.00245	4.876	0.00292	4.876	0.0033	4.876	0.00362	4.876	0.0039
4.877	0.00245	4.877	0.00292	4.877	0.0033	4.877	0.00362	4.877	0.00389
4.878	0.00245	4.878	0.00291	4.878	0.0033	4.878	0.00362	4.878	0.00389
4.879	0.00245	4.879	0.00291	4.879	0.0033	4.879	0.00362	4.879	0.00389
4.88	0.00244	4.88	0.00291	4.88	0.0033	4.88	0.00362	4.88	0.00389
4.881	0.00244	4.881	0.00291	4.881	0.0033	4.881	0.00362	4.881	0.00389
4.882	0.00244	4.882	0.00291	4.882	0.00329	4.882	0.00362	4.882	0.00389
4.883	0.00244	4.883	0.00291	4.883	0.00329	4.883	0.00361	4.883	0.00389
4.884	0.00244	4.884	0.00291	4.884	0.00329	4.884	0.00361	4.884	0.00389
4.885	0.00244	4.885	0.00291	4.885	0.00329	4.885	0.00361	4.885	0.00389
4.886	0.00244	4.886	0.00291	4.886	0.00329	4.886	0.00361	4.886	0.00389
4.887	0.00244	4.887	0.00291	4.887	0.00329	4.887	0.00361	4.887	0.00389

4.888	0.00244	4.888	0.00291	4.888	0.00329	4.888	0.00361	4.888	0.00389
4.889	0.00244	4.889	0.00291	4.889	0.00329	4.889	0.00361	4.889	0.00388
4.89	0.00244	4.89	0.00291	4.89	0.00329	4.89	0.00361	4.89	0.00388
4.891	0.00244	4.891	0.00291	4.891	0.00329	4.891	0.00361	4.891	0.00388
4.892	0.00244	4.892	0.00291	4.892	0.00329	4.892	0.00361	4.892	0.00388
4.893	0.00244	4.893	0.00291	4.893	0.00329	4.893	0.00361	4.893	0.00388
4.894	0.00244	4.894	0.0029	4.894	0.00329	4.894	0.00361	4.894	0.00388
4.895	0.00244	4.895	0.0029	4.895	0.00329	4.895	0.00361	4.895	0.00388
4.896	0.00244	4.896	0.0029	4.896	0.00329	4.896	0.00361	4.896	0.00388
4.897	0.00244	4.897	0.0029	4.897	0.00328	4.897	0.0036	4.897	0.00388
4.898	0.00244	4.898	0.0029	4.898	0.00328	4.898	0.0036	4.898	0.00388
4.899	0.00244	4.899	0.0029	4.899	0.00328	4.899	0.0036	4.899	0.00388
4.9	0.00243	4.9	0.0029	4.9	0.00328	4.9	0.0036	4.9	0.00388
4.901	0.00243	4.901	0.0029	4.901	0.00328	4.901	0.0036	4.901	0.00388
4.902	0.00243	4.902	0.0029	4.902	0.00328	4.902	0.0036	4.902	0.00387
4.903	0.00243	4.903	0.0029	4.903	0.00328	4.903	0.0036	4.903	0.00387
4.904	0.00243	4.904	0.0029	4.904	0.00328	4.904	0.0036	4.904	0.00387
4.905	0.00243	4.905	0.0029	4.905	0.00328	4.905	0.0036	4.905	0.00387
4.906	0.00243	4.906	0.0029	4.906	0.00328	4.906	0.0036	4.906	0.00387
4.907	0.00243	4.907	0.0029	4.907	0.00328	4.907	0.0036	4.907	0.00387
4.908	0.00243	4.908	0.0029	4.908	0.00328	4.908	0.0036	4.908	0.00387
4.909	0.00243	4.909	0.0029	4.909	0.00328	4.909	0.0036	4.909	0.00387
4.91	0.00243	4.91	0.0029	4.91	0.00328	4.91	0.0036	4.91	0.00387
4.911	0.00243	4.911	0.00289	4.911	0.00328	4.911	0.00359	4.911	0.00387
4.912	0.00243	4.912	0.00289	4.912	0.00327	4.912	0.00359	4.912	0.00387
4.913	0.00243	4.913	0.00289	4.913	0.00327	4.913	0.00359	4.913	0.00387
4.914	0.00243	4.914	0.00289	4.914	0.00327	4.914	0.00359	4.914	0.00387
4.915	0.00243	4.915	0.00289	4.915	0.00327	4.915	0.00359	4.915	0.00386
4.916	0.00243	4.916	0.00289	4.916	0.00327	4.916	0.00359	4.916	0.00386
4.917	0.00243	4.917	0.00289	4.917	0.00327	4.917	0.00359	4.917	0.00386
4.918	0.00243	4.918	0.00289	4.918	0.00327	4.918	0.00359	4.918	0.00386
4.919	0.00243	4.919	0.00289	4.919	0.00327	4.919	0.00359	4.919	0.00386
4.92	0.00242	4.92	0.00289	4.92	0.00327	4.92	0.00359	4.92	0.00386
4.921	0.00242	4.921	0.00289	4.921	0.00327	4.921	0.00359	4.921	0.00386
4.922	0.00242	4.922	0.00289	4.922	0.00327	4.922	0.00359	4.922	0.00386
4.923	0.00242	4.923	0.00289	4.923	0.00327	4.923	0.00359	4.923	0.00386
4.924	0.00242	4.924	0.00289	4.924	0.00327	4.924	0.00358	4.924	0.00386
4.925	0.00242	4.925	0.00289	4.925	0.00327	4.925	0.00358	4.925	0.00386
4.926	0.00242	4.926	0.00289	4.926	0.00327	4.926	0.00358	4.926	0.00386
4.927	0.00242	4.927	0.00289	4.927	0.00326	4.927	0.00358	4.927	0.00385
4.928	0.00242	4.928	0.00288	4.928	0.00326	4.928	0.00358	4.928	0.00385
4.929	0.00242	4.929	0.00288	4.929	0.00326	4.929	0.00358	4.929	0.00385
4.93	0.00242	4.93	0.00288	4.93	0.00326	4.93	0.00358	4.93	0.00385
4.931	0.00242	4.931	0.00288	4.931	0.00326	4.931	0.00358	4.931	0.00385
4.932	0.00242	4.932	0.00288	4.932	0.00326	4.932	0.00358	4.932	0.00385
4.933	0.00242	4.933	0.00288	4.933	0.00326	4.933	0.00358	4.933	0.00385
4.934	0.00242	4.934	0.00288	4.934	0.00326	4.934	0.00358	4.934	0.00385
4.935	0.00242	4.935	0.00288	4.935	0.00326	4.935	0.00358	4.935	0.00385
4.936	0.00242	4.936	0.00288	4.936	0.00326	4.936	0.00358	4.936	0.00385
4.937	0.00242	4.937	0.00288	4.937	0.00326	4.937	0.00358	4.937	0.00385
4.938	0.00242	4.938	0.00288	4.938	0.00326	4.938	0.00357	4.938	0.00385
4.939	0.00242	4.939	0.00288	4.939	0.00326	4.939	0.00357	4.939	0.00385
4.94	0.00241	4.94	0.00288	4.94	0.00326	4.94	0.00357	4.94	0.00384
4.941	0.00241	4.941	0.00288	4.941	0.00326	4.941	0.00357	4.941	0.00384
4.942	0.00241	4.942	0.00288	4.942	0.00325	4.942	0.00357	4.942	0.00384
4.943	0.00241	4.943	0.00288	4.943	0.00325	4.943	0.00357	4.943	0.00384
4.944	0.00241	4.944	0.00288	4.944	0.00325	4.944	0.00357	4.944	0.00384
4.945	0.00241	4.945	0.00287	4.945	0.00325	4.945	0.00357	4.945	0.00384
4.946	0.00241	4.946	0.00287	4.946	0.00325	4.946	0.00357	4.946	0.00384
4.947	0.00241	4.947	0.00287	4.947	0.00325	4.947	0.00357	4.947	0.00384
4.948	0.00241	4.948	0.00287	4.948	0.00325	4.948	0.00357	4.948	0.00384
4.949	0.00241	4.949	0.00287	4.949	0.00325	4.949	0.00357	4.949	0.00384
4.95	0.00241	4.95	0.00287	4.95	0.00325	4.95	0.00357	4.95	0.00384
4.951	0.00241	4.951	0.00287	4.951	0.00325	4.951	0.00357	4.951	0.00384
4.952	0.00241	4.952	0.00287	4.952	0.00325	4.952	0.00356	4.952	0.00384
4.953	0.00241	4.953	0.00287	4.953	0.00325	4.953	0.00356	4.953	0.00383
4.954	0.00241	4.954	0.00287	4.954	0.00325	4.954	0.00356	4.954	0.00383
4.955	0.00241	4.955	0.00287	4.955	0.00325	4.955	0.00356	4.955	0.00383
4.956	0.00241	4.956	0.00287	4.956	0.00325	4.956	0.00356	4.956	0.00383
4.957	0.00241	4.957	0.00287	4.957	0.00324	4.957	0.00356	4.957	0.00383
4.958	0.00241	4.958	0.00287	4.958	0.00324	4.958	0.00356	4.958	0.00383
4.959	0.00241	4.959	0.00287	4.959	0.00324	4.959	0.00356	4.959	0.00383
4.96	0.00241	4.96	0.00287	4.96	0.00324	4.96	0.00356	4.96	0.00383
4.961	0.0024	4.961	0.00287	4.961	0.00324	4.961	0.00356	4.961	0.00383
4.962	0.0024	4.962	0.00287	4.962	0.00324	4.962	0.00356	4.962	0.00383
4.963	0.0024	4.963	0.00286	4.963	0.00324	4.963	0.00356	4.963	0.00383
4.964	0.0024	4.964	0.00286	4.964	0.00324	4.964	0.00356	4.964	0.00383
4.965	0.0024	4.965	0.00286	4.965	0.00324	4.965	0.00356	4.965	0.00383
4.966	0.0024	4.966	0.00286	4.966	0.00324	4.966	0.00355	4.966	0.00382
4.967	0.0024	4.967	0.00286	4.967	0.00324	4.967	0.00355	4.967	0.00382
4.968	0.0024	4.968	0.00286	4.968	0.00324	4.968	0.00355	4.968	0.00382
4.969	0.0024	4.969	0.00286	4.969	0.00324	4.969	0.00355	4.969	0.00382
4.97	0.0024	4.97	0.00286	4.97	0.00324	4.97	0.00355	4.97	0.00382
4.971	0.0024	4.971	0.00286	4.971	0.00324	4.971	0.00355	4.971	0.00382
4.972	0.0024	4.972	0.00286	4.972	0.00324	4.972	0.00355	4.972	0.00382
4.973	0.0024	4.973	0.00286	4.973	0.00323	4.973	0.00355	4.973	0.00382
4.974	0.0024	4.974	0.00286	4.974	0.00323	4.974	0.00355	4.974	0.00382
4.975	0.0024	4.975	0.00286	4.975	0.00323	4.975	0.00355	4.975	0.00382
4.976	0.0024	4.976	0.00286	4.976	0.00323	4.976	0.00355	4.976	0.00382
4.977	0.0024	4.977	0.00286	4.977	0.00323	4.977	0.00355	4.977	0.00382
4.978	0.0024	4.978	0.00286	4.978	0.00323	4.978	0.00355	4.978	0.00382
4.979	0.0024	4.979	0.00286	4.979	0.00323	4.979	0.00355	4.979	0.00381
4.98	0.0024	4.98	0.00285	4.98	0.00323	4.98	0.00354	4.98	0.00381
4.981	0.0024	4.981	0.00285	4.981	0.00323	4.981	0.00354	4.981	0.00381
4.982	0.00239	4.982	0.00285	4.982	0.00323	4.982	0.00354	4.982	0.00381
4.983	0.00239	4.983	0.00285	4.983	0.00323	4.983	0.00354	4.983	0.00381
4.984	0.00239	4.984	0.00285	4.984	0.00323	4.984	0.00354	4.984	0.00381
4.985	0.00239	4.985	0.00285	4.985	0.00323	4.985	0.00354	4.985	0.00381
4.986	0.00239	4.986	0.00285	4.986	0.00323	4.986	0.00354	4.986	0.00381
4.987	0.00239	4.987	0.00285	4.987	0.00323	4.987	0.00354	4.987	0.00381
4.988	0.00239	4.988	0.00285	4.988	0.00322	4.988	0.00354	4.988	0.00381
4.989	0.00239	4.989	0.00285	4.989	0.00322	4.989	0.00354	4.989	0.00381
4.99	0.00239	4.99	0.00285	4.99	0.00322	4.99	0.00354	4.99	0.00381
4.991	0.00239	4.991	0.00285	4.991	0.00322	4.991	0.00354	4.991	0.00381
4.992	0.00239	4.992	0.00285	4.992	0.00322	4.992	0.00354	4.992	0.0038
4.993	0.00239	4.993	0.00285	4.993	0.00322	4.993	0.00354	4.993	0.0038
4.994	0.00239	4.994	0.00285	4.994	0.00322	4.994	0.00353	4.994	0.0038
4.995	0.00239	4.995	0.00285	4.995	0.00322	4.995	0.00353	4.995	0.0038
4.996	0.00239	4.996	0.00285	4.996	0.00322	4.996	0.00353	4.996	0.0038
4.997	0.00239	4.997	0.00285	4.997	0.00322	4.997	0.00353	4.997	0.0038
4.998	0.00239	4.998	0.00284	4.998	0.00322	4.998	0.00353	4.998	0.0038
4.999	0.00239	4.999	0.00284	4.999	0.00322	4.999	0.00353	4.999	0.0038
5	0.00239	5	0.00284	5	0.00322	5	0.00353	5	0.0038
5.001	0.00239	5.001	0.00284	5.001	0.00322	5.001	0.00353	5.001	0.0038
5.002	0.00238	5.002	0.00284	5.002	0.00322	5.002	0.00353	5.002	0.0038
5.003	0.00238	5.003	0.00284	5.003	0.00322	5.003	0.00353	5.003	0.0038
5.004	0.00238	5.004	0.00284	5.004	0.00321	5.004	0.00353	5.004	0.0038
5.005	0.00238	5.005	0.00284	5.005	0.00321	5.005	0.00353	5.005	0.00379
5.006	0.00238	5.006	0.00284	5.006	0.00321	5.006	0.00353	5.006	0.00379
5.007	0.00238	5.007	0.00284	5.007	0.00321	5.007	0.00353	5.007	0.00379
5.008	0.00238	5.008	0.00284	5.008	0.00321	5.008	0.00352	5.008	0.00379
5.009	0.00238	5.009	0.00284	5.009	0.00321	5.009	0.00352	5.009	0.00379
5.01	0.00238	5.01	0.00284	5.01	0.00321	5.01	0.00352	5.01	0.00379
5.011	0.00238	5.011	0.00284	5.011	0.00321	5.011	0.00352	5.011	0.00379
5.012	0.00238	5.012	0.00284	5.012	0.00321	5.012	0.00352	5.012	0.00379
5.013	0.00238	5.013	0.00284	5.013	0.00321	5.013	0.00352	5.013	0.00379
5.014	0.00238	5.014	0.00284	5.014	0.00321	5.014	0.00352	5.014	0.00379
5.015	0.00238	5.015	0.00283	5.015	0.00321	5.015	0.00352	5.015	0.00379
5.016	0.00238	5.016	0.00283	5.016	0.00321	5.016	0.00352	5.016	0.00379
5.017	0.00238	5.017	0.00283	5.017	0.00321	5.017	0.00352	5.017	0.00379
5.018	0.00238	5.018	0.00283	5.018	0.00321	5.018	0.00352	5.018	0.00378
5.019	0.00238	5.019	0.00283	5.019	0.0032	5.019	0.00352	5.019	0.00378
5.02	0.00238	5.02	0.00283	5.02	0.0032	5.02	0.00352	5.02	0.00378
5.021	0.00238	5.021	0.00283	5.021	0.0032	5.021	0.00352	5.021	0.00378
5.022	0.00238	5.022	0.00283	5.022	0.0032	5.022	0.00351	5.022	0.00378

5.023	0.00238	5.023	0.00283	5.023	0.0032	5.023	0.00351	5.023	0.00378
5.024	0.00237	5.024	0.00283	5.024	0.0032	5.024	0.00351	5.024	0.00378
5.025	0.00237	5.025	0.00283	5.025	0.0032	5.025	0.00351	5.025	0.00378
5.026	0.00237	5.026	0.00283	5.026	0.0032	5.026	0.00351	5.026	0.00378
5.027	0.00237	5.027	0.00283	5.027	0.0032	5.027	0.00351	5.027	0.00378
5.028	0.00237	5.028	0.00283	5.028	0.0032	5.028	0.00351	5.028	0.00378
5.029	0.00237	5.029	0.00283	5.029	0.0032	5.029	0.00351	5.029	0.00378
5.03	0.00237	5.03	0.00283	5.03	0.0032	5.03	0.00351	5.03	0.00378
5.031	0.00237	5.031	0.00283	5.031	0.0032	5.031	0.00351	5.031	0.00378
5.032	0.00237	5.032	0.00283	5.032	0.0032	5.032	0.00351	5.032	0.00377
5.033	0.00237	5.033	0.00282	5.033	0.0032	5.033	0.00351	5.033	0.00377
5.034	0.00237	5.034	0.00282	5.034	0.0032	5.034	0.00351	5.034	0.00377
5.035	0.00237	5.035	0.00282	5.035	0.00319	5.035	0.00351	5.035	0.00377
5.036	0.00237	5.036	0.00282	5.036	0.00319	5.036	0.00351	5.036	0.00377
5.037	0.00237	5.037	0.00282	5.037	0.00319	5.037	0.0035	5.037	0.00377
5.038	0.00237	5.038	0.00282	5.038	0.00319	5.038	0.0035	5.038	0.00377
5.039	0.00237	5.039	0.00282	5.039	0.00319	5.039	0.0035	5.039	0.00377
5.04	0.00237	5.04	0.00282	5.04	0.00319	5.04	0.0035	5.04	0.00377
5.041	0.00237	5.041	0.00282	5.041	0.00319	5.041	0.0035	5.041	0.00377
5.042	0.00237	5.042	0.00282	5.042	0.00319	5.042	0.0035	5.042	0.00377
5.043	0.00237	5.043	0.00282	5.043	0.00319	5.043	0.0035	5.043	0.00377
5.044	0.00237	5.044	0.00282	5.044	0.00319	5.044	0.0035	5.044	0.00377
5.045	0.00236	5.045	0.00282	5.045	0.00319	5.045	0.0035	5.045	0.00376
5.046	0.00236	5.046	0.00282	5.046	0.00319	5.046	0.0035	5.046	0.00376
5.047	0.00236	5.047	0.00282	5.047	0.00319	5.047	0.0035	5.047	0.00376
5.048	0.00236	5.048	0.00282	5.048	0.00319	5.048	0.0035	5.048	0.00376
5.049	0.00236	5.049	0.00282	5.049	0.00319	5.049	0.0035	5.049	0.00376
5.05	0.00236	5.05	0.00282	5.05	0.00319	5.05	0.0035	5.05	0.00376
5.051	0.00236	5.051	0.00281	5.051	0.00318	5.051	0.00349	5.051	0.00376
5.052	0.00236	5.052	0.00281	5.052	0.00318	5.052	0.00349	5.052	0.00376
5.053	0.00236	5.053	0.00281	5.053	0.00318	5.053	0.00349	5.053	0.00376
5.054	0.00236	5.054	0.00281	5.054	0.00318	5.054	0.00349	5.054	0.00376
5.055	0.00236	5.055	0.00281	5.055	0.00318	5.055	0.00349	5.055	0.00376
5.056	0.00236	5.056	0.00281	5.056	0.00318	5.056	0.00349	5.056	0.00376
5.057	0.00236	5.057	0.00281	5.057	0.00318	5.057	0.00349	5.057	0.00376
5.058	0.00236	5.058	0.00281	5.058	0.00318	5.058	0.00349	5.058	0.00376
5.059	0.00236	5.059	0.00281	5.059	0.00318	5.059	0.00349	5.059	0.00375
5.06	0.00236	5.06	0.00281	5.06	0.00318	5.06	0.00349	5.06	0.00375
5.061	0.00236	5.061	0.00281	5.061	0.00318	5.061	0.00349	5.061	0.00375
5.062	0.00236	5.062	0.00281	5.062	0.00318	5.062	0.00349	5.062	0.00375
5.063	0.00236	5.063	0.00281	5.063	0.00318	5.063	0.00349	5.063	0.00375
5.064	0.00236	5.064	0.00281	5.064	0.00318	5.064	0.00349	5.064	0.00375
5.065	0.00236	5.065	0.00281	5.065	0.00318	5.065	0.00348	5.065	0.00375
5.066	0.00235	5.066	0.00281	5.066	0.00318	5.066	0.00348	5.066	0.00375
5.067	0.00235	5.067	0.00281	5.067	0.00317	5.067	0.00348	5.067	0.00375
5.068	0.00235	5.068	0.00281	5.068	0.00317	5.068	0.00348	5.068	0.00375
5.069	0.00235	5.069	0.0028	5.069	0.00317	5.069	0.00348	5.069	0.00375
5.07	0.00235	5.07	0.0028	5.07	0.00317	5.07	0.00348	5.07	0.00375
5.071	0.00235	5.071	0.0028	5.071	0.00317	5.071	0.00348	5.071	0.00375
5.072	0.00235	5.072	0.0028	5.072	0.00317	5.072	0.00348	5.072	0.00374
5.073	0.00235	5.073	0.0028	5.073	0.00317	5.073	0.00348	5.073	0.00374
5.074	0.00235	5.074	0.0028	5.074	0.00317	5.074	0.00348	5.074	0.00374
5.075	0.00235	5.075	0.0028	5.075	0.00317	5.075	0.00348	5.075	0.00374
5.076	0.00235	5.076	0.0028	5.076	0.00317	5.076	0.00348	5.076	0.00374
5.077	0.00235	5.077	0.0028	5.077	0.00317	5.077	0.00348	5.077	0.00374
5.078	0.00235	5.078	0.0028	5.078	0.00317	5.078	0.00348	5.078	0.00374
5.079	0.00235	5.079	0.0028	5.079	0.00317	5.079	0.00348	5.079	0.00374
5.08	0.00235	5.08	0.0028	5.08	0.00317	5.08	0.00347	5.08	0.00374
5.081	0.00235	5.081	0.0028	5.081	0.00317	5.081	0.00347	5.081	0.00374
5.082	0.00235	5.082	0.0028	5.082	0.00317	5.082	0.00347	5.082	0.00374
5.083	0.00235	5.083	0.0028	5.083	0.00316	5.083	0.00347	5.083	0.00374
5.084	0.00235	5.084	0.0028	5.084	0.00316	5.084	0.00347	5.084	0.00374
5.085	0.00235	5.085	0.0028	5.085	0.00316	5.085	0.00347	5.085	0.00374
5.086	0.00235	5.086	0.0028	5.086	0.00316	5.086	0.00347	5.086	0.00373
5.087	0.00235	5.087	0.00279	5.087	0.00316	5.087	0.00347	5.087	0.00373
5.088	0.00234	5.088	0.00279	5.088	0.00316	5.088	0.00347	5.088	0.00373
5.089	0.00234	5.089	0.00279	5.089	0.00316	5.089	0.00347	5.089	0.00373
5.09	0.00234	5.09	0.00279	5.09	0.00316	5.09	0.00347	5.09	0.00373
5.091	0.00234	5.091	0.00279	5.091	0.00316	5.091	0.00347	5.091	0.00373
5.092	0.00234	5.092	0.00279	5.092	0.00316	5.092	0.00347	5.092	0.00373
5.093	0.00234	5.093	0.00279	5.093	0.00316	5.093	0.00347	5.093	0.00373
5.094	0.00234	5.094	0.00279	5.094	0.00316	5.094	0.00347	5.094	0.00373
5.095	0.00234	5.095	0.00279	5.095	0.00316	5.095	0.00346	5.095	0.00373
5.096	0.00234	5.096	0.00279	5.096	0.00316	5.096	0.00346	5.096	0.00373
5.097	0.00234	5.097	0.00279	5.097	0.00316	5.097	0.00346	5.097	0.00373
5.098	0.00234	5.098	0.00279	5.098	0.00316	5.098	0.00346	5.098	0.00373
5.099	0.00234	5.099	0.00279	5.099	0.00315	5.099	0.00346	5.099	0.00372
5.1	0.00234	5.1	0.00279	5.1	0.00315	5.1	0.00346	5.1	0.00372
5.101	0.00234	5.101	0.00279	5.101	0.00315	5.101	0.00346	5.101	0.00372
5.102	0.00234	5.102	0.00279	5.102	0.00315	5.102	0.00346	5.102	0.00372
5.103	0.00234	5.103	0.00279	5.103	0.00315	5.103	0.00346	5.103	0.00372
5.104	0.00234	5.104	0.00279	5.104	0.00315	5.104	0.00346	5.104	0.00372
5.105	0.00234	5.105	0.00278	5.105	0.00315	5.105	0.00346	5.105	0.00372
5.106	0.00234	5.106	0.00278	5.106	0.00315	5.106	0.00346	5.106	0.00372
5.107	0.00234	5.107	0.00278	5.107	0.00315	5.107	0.00346	5.107	0.00372
5.108	0.00234	5.108	0.00278	5.108	0.00315	5.108	0.00346	5.108	0.00372
5.109	0.00234	5.109	0.00278	5.109	0.00315	5.109	0.00345	5.109	0.00372
5.11	0.00233	5.11	0.00278	5.11	0.00315	5.11	0.00345	5.11	0.00372
5.111	0.00233	5.111	0.00278	5.111	0.00315	5.111	0.00345	5.111	0.00372
5.112	0.00233	5.112	0.00278	5.112	0.00315	5.112	0.00345	5.112	0.00372
5.113	0.00233	5.113	0.00278	5.113	0.00315	5.113	0.00345	5.113	0.00371
5.114	0.00233	5.114	0.00278	5.114	0.00315	5.114	0.00345	5.114	0.00371
5.115	0.00233	5.115	0.00278	5.115	0.00314	5.115	0.00345	5.115	0.00371
5.116	0.00233	5.116	0.00278	5.116	0.00314	5.116	0.00345	5.116	0.00371
5.117	0.00233	5.117	0.00278	5.117	0.00314	5.117	0.00345	5.117	0.00371
5.118	0.00233	5.118	0.00278	5.118	0.00314	5.118	0.00345	5.118	0.00371
5.119	0.00233	5.119	0.00278	5.119	0.00314	5.119	0.00345	5.119	0.00371
5.12	0.00233	5.12	0.00278	5.12	0.00314	5.12	0.00345	5.12	0.00371
5.121	0.00233	5.121	0.00278	5.121	0.00314	5.121	0.00345	5.121	0.00371
5.122	0.00233	5.122	0.00278	5.122	0.00314	5.122	0.00345	5.122	0.00371
5.123	0.00233	5.123	0.00278	5.123	0.00314	5.123	0.00345	5.123	0.00371
5.124	0.00233	5.124	0.00277	5.124	0.00314	5.124	0.00344	5.124	0.00371
5.125	0.00233	5.125	0.00277	5.125	0.00314	5.125	0.00344	5.125	0.00371
5.126	0.00233	5.126	0.00277	5.126	0.00314	5.126	0.00344	5.126	0.00371
5.127	0.00233	5.127	0.00277	5.127	0.00314	5.127	0.00344	5.127	0.0037
5.128	0.00233	5.128	0.00277	5.128	0.00314	5.128	0.00344	5.128	0.0037
5.129	0.00233	5.129	0.00277	5.129	0.00314	5.129	0.00344	5.129	0.0037
5.13	0.00233	5.13	0.00277	5.13	0.00314	5.13	0.00344	5.13	0.0037
5.131	0.00233	5.131	0.00277	5.131	0.00313	5.131	0.00344	5.131	0.0037
5.132	0.00232	5.132	0.00277	5.132	0.00313	5.132	0.00344	5.132	0.0037
5.133	0.00232	5.133	0.00277	5.133	0.00313	5.133	0.00344	5.133	0.0037
5.134	0.00232	5.134	0.00277	5.134	0.00313	5.134	0.00344	5.134	0.0037
5.135	0.00232	5.135	0.00277	5.135	0.00313	5.135	0.00344	5.135	0.0037
5.136	0.00232	5.136	0.00277	5.136	0.00313	5.136	0.00344	5.136	0.0037
5.137	0.00232	5.137	0.00277	5.137	0.00313	5.137	0.00344	5.137	0.0037
5.138	0.00232	5.138	0.00277	5.138	0.00313	5.138	0.00344	5.138	0.0037
5.139	0.00232	5.139	0.00277	5.139	0.00313	5.139	0.00343	5.139	0.0037
5.14	0.00232	5.14	0.00277	5.14	0.00313	5.14	0.00343	5.14	0.0037
5.141	0.00232	5.141	0.00277	5.141	0.00313	5.141	0.00343	5.141	0.00369
5.142	0.00232	5.142	0.00276	5.142	0.00313	5.142	0.00343	5.142	0.00369
5.143	0.00232	5.143	0.00276	5.143	0.00313	5.143	0.00343	5.143	0.00369
5.144	0.00232	5.144	0.00276	5.144	0.00313	5.144	0.00343	5.144	0.00369
5.145	0.00232	5.145	0.00276	5.145	0.00313	5.145	0.00343	5.145	0.00369
5.146	0.00232	5.146	0.00276	5.146	0.00313	5.146	0.00343	5.146	0.00369
5.147	0.00232	5.147	0.00276	5.147	0.00313	5.147	0.00343	5.147	0.00369
5.148	0.00232	5.148	0.00276	5.148	0.00312	5.148	0.00343	5.148	0.00369
5.149	0.00232	5.149	0.00276	5.149	0.00312	5.149	0.00343	5.149	0.00369
5.15	0.00232	5.15	0.00276	5.15	0.00312	5.15	0.00343	5.15	0.00369
5.151	0.00232	5.151	0.00276	5.151	0.00312	5.151	0.00343	5.151	0.00369
5.152	0.00232	5.152	0.00276	5.152	0.00312	5.152	0.00343	5.152	0.00369
5.153	0.00232	5.153	0.00276	5.153	0.00312	5.153	0.00343	5.153	0.00369
5.154	0.00231	5.154	0.00276	5.154	0.00312	5.154	0.00342	5.154	0.00369
5.155	0.00231	5.155	0.00276	5.155	0.00312	5.155	0.00342	5.155	0.00368
5.156	0.00231	5.156	0.00276	5.156	0.00312	5.156	0.00342	5.156	0.00368
5.157	0.00231	5.157	0.00276	5.157	0.00312	5.157	0.00342	5.157	0.00368

5.158	0.00231	5.158	0.00276	5.158	0.00312	5.158	0.00342	5.158	0.00368
5.159	0.00231	5.159	0.00276	5.159	0.00312	5.159	0.00342	5.159	0.00368
5.16	0.00231	5.16	0.00276	5.16	0.00312	5.16	0.00342	5.16	0.00368
5.161	0.00231	5.161	0.00275	5.161	0.00312	5.161	0.00342	5.161	0.00368
5.162	0.00231	5.162	0.00275	5.162	0.00312	5.162	0.00342	5.162	0.00368
5.163	0.00231	5.163	0.00275	5.163	0.00312	5.163	0.00342	5.163	0.00368
5.164	0.00231	5.164	0.00275	5.164	0.00311	5.164	0.00342	5.164	0.00368
5.165	0.00231	5.165	0.00275	5.165	0.00311	5.165	0.00342	5.165	0.00368
5.166	0.00231	5.166	0.00275	5.166	0.00311	5.166	0.00342	5.166	0.00368
5.167	0.00231	5.167	0.00275	5.167	0.00311	5.167	0.00342	5.167	0.00368
5.168	0.00231	5.168	0.00275	5.168	0.00311	5.168	0.00342	5.168	0.00368
5.169	0.00231	5.169	0.00275	5.169	0.00311	5.169	0.00341	5.169	0.00367
5.17	0.00231	5.17	0.00275	5.17	0.00311	5.17	0.00341	5.17	0.00367
5.171	0.00231	5.171	0.00275	5.171	0.00311	5.171	0.00341	5.171	0.00367
5.172	0.00231	5.172	0.00275	5.172	0.00311	5.172	0.00341	5.172	0.00367
5.173	0.00231	5.173	0.00275	5.173	0.00311	5.173	0.00341	5.173	0.00367
5.174	0.00231	5.174	0.00275	5.174	0.00311	5.174	0.00341	5.174	0.00367
5.175	0.00231	5.175	0.00275	5.175	0.00311	5.175	0.00341	5.175	0.00367
5.176	0.0023	5.176	0.00275	5.176	0.00311	5.176	0.00341	5.176	0.00367
5.177	0.0023	5.177	0.00275	5.177	0.00311	5.177	0.00341	5.177	0.00367
5.178	0.0023	5.178	0.00275	5.178	0.00311	5.178	0.00341	5.178	0.00367
5.179	0.0023	5.179	0.00275	5.179	0.00311	5.179	0.00341	5.179	0.00367
5.18	0.0023	5.18	0.00274	5.18	0.00311	5.18	0.00341	5.18	0.00367
5.181	0.0023	5.181	0.00274	5.181	0.0031	5.181	0.00341	5.181	0.00367
5.182	0.0023	5.182	0.00274	5.182	0.0031	5.182	0.00341	5.182	0.00367
5.183	0.0023	5.183	0.00274	5.183	0.0031	5.183	0.00341	5.183	0.00366
5.184	0.0023	5.184	0.00274	5.184	0.0031	5.184	0.0034	5.184	0.00366
5.185	0.0023	5.185	0.00274	5.185	0.0031	5.185	0.0034	5.185	0.00366
5.186	0.0023	5.186	0.00274	5.186	0.0031	5.186	0.0034	5.186	0.00366
5.187	0.0023	5.187	0.00274	5.187	0.0031	5.187	0.0034	5.187	0.00366
5.188	0.0023	5.188	0.00274	5.188	0.0031	5.188	0.0034	5.188	0.00366
5.189	0.0023	5.189	0.00274	5.189	0.0031	5.189	0.0034	5.189	0.00366
5.19	0.0023	5.19	0.00274	5.19	0.0031	5.19	0.0034	5.19	0.00366
5.191	0.0023	5.191	0.00274	5.191	0.0031	5.191	0.0034	5.191	0.00366
5.192	0.0023	5.192	0.00274	5.192	0.0031	5.192	0.0034	5.192	0.00366
5.193	0.0023	5.193	0.00274	5.193	0.0031	5.193	0.0034	5.193	0.00366
5.194	0.0023	5.194	0.00274	5.194	0.0031	5.194	0.0034	5.194	0.00366
5.195	0.0023	5.195	0.00274	5.195	0.0031	5.195	0.0034	5.195	0.00366
5.196	0.0023	5.196	0.00274	5.196	0.0031	5.196	0.0034	5.196	0.00366
5.197	0.0023	5.197	0.00274	5.197	0.0031	5.197	0.0034	5.197	0.00365
5.198	0.0023	5.198	0.00274	5.198	0.00309	5.198	0.0034	5.198	0.00365
5.199	0.00229	5.199	0.00273	5.199	0.00309	5.199	0.0034	5.199	0.00365
5.2	0.00229	5.2	0.00273	5.2	0.00309	5.2	0.00339	5.2	0.00365
5.201	0.00229	5.201	0.00273	5.201	0.00309	5.201	0.00339	5.201	0.00365
5.202	0.00229	5.202	0.00273	5.202	0.00309	5.202	0.00339	5.202	0.00365
5.203	0.00229	5.203	0.00273	5.203	0.00309	5.203	0.00339	5.203	0.00365
5.204	0.00229	5.204	0.00273	5.204	0.00309	5.204	0.00339	5.204	0.00365
5.205	0.00229	5.205	0.00273	5.205	0.00309	5.205	0.00339	5.205	0.00365
5.206	0.00229	5.206	0.00273	5.206	0.00309	5.206	0.00339	5.206	0.00365
5.207	0.00229	5.207	0.00273	5.207	0.00309	5.207	0.00339	5.207	0.00365
5.208	0.00229	5.208	0.00273	5.208	0.00309	5.208	0.00339	5.208	0.00365
5.209	0.00229	5.209	0.00273	5.209	0.00309	5.209	0.00339	5.209	0.00365
5.21	0.00229	5.21	0.00273	5.21	0.00309	5.21	0.00339	5.21	0.00365
5.211	0.00229	5.211	0.00273	5.211	0.00309	5.211	0.00339	5.211	0.00364
5.212	0.00229	5.212	0.00273	5.212	0.00309	5.212	0.00339	5.212	0.00364
5.213	0.00229	5.213	0.00273	5.213	0.00309	5.213	0.00339	5.213	0.00364
5.214	0.00229	5.214	0.00273	5.214	0.00308	5.214	0.00339	5.214	0.00364
5.215	0.00229	5.215	0.00273	5.215	0.00308	5.215	0.00338	5.215	0.00364
5.216	0.00229	5.216	0.00273	5.216	0.00308	5.216	0.00338	5.216	0.00364
5.217	0.00229	5.217	0.00273	5.217	0.00308	5.217	0.00338	5.217	0.00364
5.218	0.00229	5.218	0.00272	5.218	0.00308	5.218	0.00338	5.218	0.00364
5.219	0.00229	5.219	0.00272	5.219	0.00308	5.219	0.00338	5.219	0.00364
5.22	0.00229	5.22	0.00272	5.22	0.00308	5.22	0.00338	5.22	0.00364
5.221	0.00228	5.221	0.00272	5.221	0.00308	5.221	0.00338	5.221	0.00364
5.222	0.00228	5.222	0.00272	5.222	0.00308	5.222	0.00338	5.222	0.00364
5.223	0.00228	5.223	0.00272	5.223	0.00308	5.223	0.00338	5.223	0.00364
5.224	0.00228	5.224	0.00272	5.224	0.00308	5.224	0.00338	5.224	0.00364
5.225	0.00228	5.225	0.00272	5.225	0.00308	5.225	0.00338	5.225	0.00364
5.226	0.00228	5.226	0.00272	5.226	0.00308	5.226	0.00338	5.226	0.00363
5.227	0.00228	5.227	0.00272	5.227	0.00308	5.227	0.00338	5.227	0.00363
5.228	0.00228	5.228	0.00272	5.228	0.00308	5.228	0.00338	5.228	0.00363
5.229	0.00228	5.229	0.00272	5.229	0.00308	5.229	0.00338	5.229	0.00363
5.23	0.00228	5.23	0.00272	5.23	0.00308	5.23	0.00338	5.23	0.00363
5.231	0.00228	5.231	0.00272	5.231	0.00307	5.231	0.00337	5.231	0.00363
5.232	0.00228	5.232	0.00272	5.232	0.00307	5.232	0.00337	5.232	0.00363
5.233	0.00228	5.233	0.00272	5.233	0.00307	5.233	0.00337	5.233	0.00363
5.234	0.00228	5.234	0.00272	5.234	0.00307	5.234	0.00337	5.234	0.00363
5.235	0.00228	5.235	0.00272	5.235	0.00307	5.235	0.00337	5.235	0.00363
5.236	0.00228	5.236	0.00272	5.236	0.00307	5.236	0.00337	5.236	0.00363
5.237	0.00228	5.237	0.00271	5.237	0.00307	5.237	0.00337	5.237	0.00363
5.238	0.00228	5.238	0.00271	5.238	0.00307	5.238	0.00337	5.238	0.00363
5.239	0.00228	5.239	0.00271	5.239	0.00307	5.239	0.00337	5.239	0.00363
5.24	0.00228	5.24	0.00271	5.24	0.00307	5.24	0.00337	5.24	0.00362
5.241	0.00228	5.241	0.00271	5.241	0.00307	5.241	0.00337	5.241	0.00362
5.242	0.00228	5.242	0.00271	5.242	0.00307	5.242	0.00337	5.242	0.00362
5.243	0.00228	5.243	0.00271	5.243	0.00307	5.243	0.00337	5.243	0.00362
5.244	0.00227	5.244	0.00271	5.244	0.00307	5.244	0.00337	5.244	0.00362
5.245	0.00227	5.245	0.00271	5.245	0.00307	5.245	0.00337	5.245	0.00362
5.246	0.00227	5.246	0.00271	5.246	0.00307	5.246	0.00336	5.246	0.00362
5.247	0.00227	5.247	0.00271	5.247	0.00307	5.247	0.00336	5.247	0.00362
5.248	0.00227	5.248	0.00271	5.248	0.00307	5.248	0.00336	5.248	0.00362
5.249	0.00227	5.249	0.00271	5.249	0.00306	5.249	0.00336	5.249	0.00362
5.25	0.00227	5.25	0.00271	5.25	0.00306	5.25	0.00336	5.25	0.00362
5.251	0.00227	5.251	0.00271	5.251	0.00306	5.251	0.00336	5.251	0.00362
5.252	0.00227	5.252	0.00271	5.252	0.00306	5.252	0.00336	5.252	0.00362
5.253	0.00227	5.253	0.00271	5.253	0.00306	5.253	0.00336	5.253	0.00362
5.254	0.00227	5.254	0.00271	5.254	0.00306	5.254	0.00336	5.254	0.00361
5.255	0.00227	5.255	0.00271	5.255	0.00306	5.255	0.00336	5.255	0.00361
5.256	0.00227	5.256	0.0027	5.256	0.00306	5.256	0.00336	5.256	0.00361
5.257	0.00227	5.257	0.0027	5.257	0.00306	5.257	0.00336	5.257	0.00361
5.258	0.00227	5.258	0.0027	5.258	0.00306	5.258	0.00336	5.258	0.00361
5.259	0.00227	5.259	0.0027	5.259	0.00306	5.259	0.00336	5.259	0.00361
5.26	0.00227	5.26	0.0027	5.26	0.00306	5.26	0.00336	5.26	0.00361
5.261	0.00227	5.261	0.0027	5.261	0.00306	5.261	0.00336	5.261	0.00361
5.262	0.00227	5.262	0.0027	5.262	0.00306	5.262	0.00335	5.262	0.00361
5.263	0.00227	5.263	0.0027	5.263	0.00306	5.263	0.00335	5.263	0.00361
5.264	0.00227	5.264	0.0027	5.264	0.00306	5.264	0.00335	5.264	0.00361
5.265	0.00227	5.265	0.0027	5.265	0.00306	5.265	0.00335	5.265	0.00361
5.266	0.00227	5.266	0.0027	5.266	0.00305	5.266	0.00335	5.266	0.00361
5.267	0.00226	5.267	0.0027	5.267	0.00305	5.267	0.00335	5.267	0.00361
5.268	0.00226	5.268	0.0027	5.268	0.00305	5.268	0.00335	5.268	0.00361
5.269	0.00226	5.269	0.0027	5.269	0.00305	5.269	0.00335	5.269	0.0036
5.27	0.00226	5.27	0.0027	5.27	0.00305	5.27	0.00335	5.27	0.0036
5.271	0.00226	5.271	0.0027	5.271	0.00305	5.271	0.00335	5.271	0.0036
5.272	0.00226	5.272	0.0027	5.272	0.00305	5.272	0.00335	5.272	0.0036
5.273	0.00226	5.273	0.0027	5.273	0.00305	5.273	0.00335	5.273	0.0036
5.274	0.00226	5.274	0.0027	5.274	0.00305	5.274	0.00335	5.274	0.0036
5.275	0.00226	5.275	0.0027	5.275	0.00305	5.275	0.00335	5.275	0.0036
5.276	0.00226	5.276	0.00269	5.276	0.00305	5.276	0.00335	5.276	0.0036
5.277	0.00226	5.277	0.00269	5.277	0.00305	5.277	0.00334	5.277	0.0036
5.278	0.00226	5.278	0.00269	5.278	0.00305	5.278	0.00334	5.278	0.0036
5.279	0.00226	5.279	0.00269	5.279	0.00305	5.279	0.00334	5.279	0.0036
5.28	0.00226	5.28	0.00269	5.28	0.00305	5.28	0.00334	5.28	0.0036
5.281	0.00226	5.281	0.00269	5.281	0.00305	5.281	0.00334	5.281	0.0036
5.282	0.00226	5.282	0.00269	5.282	0.00305	5.282	0.00334	5.282	0.0036
5.283	0.00226	5.283	0.00269	5.283	0.00304	5.283	0.00334	5.283	0.0036
5.284	0.00226	5.284	0.00269	5.284	0.00304	5.284	0.00334	5.284	0.00359
5.285	0.00226	5.285	0.00269	5.285	0.00304	5.285	0.00334	5.285	0.00359
5.286	0.00226	5.286	0.00269	5.286	0.00304	5.286	0.00334	5.286	0.00359
5.287	0.00226	5.287	0.00269	5.287	0.00304	5.287	0.00334	5.287	0.00359
5.288	0.00226	5.288	0.00269	5.288	0.00304	5.288	0.00334	5.288	0.00359
5.289	0.00226	5.289	0.00269	5.289	0.00304	5.289	0.00334	5.289	0.00359
5.29	0.00226	5.29	0.00269	5.29	0.00304	5.29	0.00334	5.29	0.00359
5.291	0.00225	5.291	0.00269	5.291	0.00304	5.291	0.00334	5.291	0.00359
5.292	0.00225	5.292	0.00269	5.292	0.00304	5.292	0.00334	5.292	0.00359

5.293	0.00225	5.293	0.00269	5.293	0.00304	5.293	0.00333	5.293	0.00359
5.294	0.00225	5.294	0.00269	5.294	0.00304	5.294	0.00333	5.294	0.00359
5.295	0.00225	5.295	0.00268	5.295	0.00304	5.295	0.00333	5.295	0.00359
5.296	0.00225	5.296	0.00268	5.296	0.00304	5.296	0.00333	5.296	0.00359
5.297	0.00225	5.297	0.00268	5.297	0.00304	5.297	0.00333	5.297	0.00359
5.298	0.00225	5.298	0.00268	5.298	0.00304	5.298	0.00333	5.298	0.00358
5.299	0.00225	5.299	0.00268	5.299	0.00304	5.299	0.00333	5.299	0.00358
5.3	0.00225	5.3	0.00268	5.3	0.00303	5.3	0.00333	5.3	0.00358
5.301	0.00225	5.301	0.00268	5.301	0.00303	5.301	0.00333	5.301	0.00358
5.302	0.00225	5.302	0.00268	5.302	0.00303	5.302	0.00333	5.302	0.00358
5.303	0.00225	5.303	0.00268	5.303	0.00303	5.303	0.00333	5.303	0.00358
5.304	0.00225	5.304	0.00268	5.304	0.00303	5.304	0.00333	5.304	0.00358
5.305	0.00225	5.305	0.00268	5.305	0.00303	5.305	0.00333	5.305	0.00358
5.306	0.00225	5.306	0.00268	5.306	0.00303	5.306	0.00333	5.306	0.00358
5.307	0.00225	5.307	0.00268	5.307	0.00303	5.307	0.00333	5.307	0.00358
5.308	0.00225	5.308	0.00268	5.308	0.00303	5.308	0.00333	5.308	0.00358
5.309	0.00225	5.309	0.00268	5.309	0.00303	5.309	0.00332	5.309	0.00358
5.31	0.00225	5.31	0.00268	5.31	0.00303	5.31	0.00332	5.31	0.00358
5.311	0.00225	5.311	0.00268	5.311	0.00303	5.311	0.00332	5.311	0.00358
5.312	0.00225	5.312	0.00268	5.312	0.00303	5.312	0.00332	5.312	0.00358
5.313	0.00225	5.313	0.00268	5.313	0.00303	5.313	0.00332	5.313	0.00357
5.314	0.00224	5.314	0.00268	5.314	0.00303	5.314	0.00332	5.314	0.00357
5.315	0.00224	5.315	0.00267	5.315	0.00303	5.315	0.00332	5.315	0.00357
5.316	0.00224	5.316	0.00267	5.316	0.00303	5.316	0.00332	5.316	0.00357
5.317	0.00224	5.317	0.00267	5.317	0.00303	5.317	0.00332	5.317	0.00357
5.318	0.00224	5.318	0.00267	5.318	0.00302	5.318	0.00332	5.318	0.00357
5.319	0.00224	5.319	0.00267	5.319	0.00302	5.319	0.00332	5.319	0.00357
5.32	0.00224	5.32	0.00267	5.32	0.00302	5.32	0.00332	5.32	0.00357
5.321	0.00224	5.321	0.00267	5.321	0.00302	5.321	0.00332	5.321	0.00357
5.322	0.00224	5.322	0.00267	5.322	0.00302	5.322	0.00332	5.322	0.00357
5.323	0.00224	5.323	0.00267	5.323	0.00302	5.323	0.00332	5.323	0.00357
5.324	0.00224	5.324	0.00267	5.324	0.00302	5.324	0.00332	5.324	0.00357
5.325	0.00224	5.325	0.00267	5.325	0.00302	5.325	0.00331	5.325	0.00357
5.326	0.00224	5.326	0.00267	5.326	0.00302	5.326	0.00331	5.326	0.00357
5.327	0.00224	5.327	0.00267	5.327	0.00302	5.327	0.00331	5.327	0.00357
5.328	0.00224	5.328	0.00267	5.328	0.00302	5.328	0.00331	5.328	0.00356
5.329	0.00224	5.329	0.00267	5.329	0.00302	5.329	0.00331	5.329	0.00356
5.33	0.00224	5.33	0.00267	5.33	0.00302	5.33	0.00331	5.33	0.00356
5.331	0.00224	5.331	0.00267	5.331	0.00302	5.331	0.00331	5.331	0.00356
5.332	0.00224	5.332	0.00267	5.332	0.00302	5.332	0.00331	5.332	0.00356
5.333	0.00224	5.333	0.00267	5.333	0.00302	5.333	0.00331	5.333	0.00356
5.334	0.00224	5.334	0.00267	5.334	0.00302	5.334	0.00331	5.334	0.00356
5.335	0.00224	5.335	0.00266	5.335	0.00302	5.335	0.00331	5.335	0.00356
5.336	0.00224	5.336	0.00266	5.336	0.00301	5.336	0.00331	5.336	0.00356
5.337	0.00224	5.337	0.00266	5.337	0.00301	5.337	0.00331	5.337	0.00356
5.338	0.00223	5.338	0.00266	5.338	0.00301	5.338	0.00331	5.338	0.00356
5.339	0.00223	5.339	0.00266	5.339	0.00301	5.339	0.00331	5.339	0.00356
5.34	0.00223	5.34	0.00266	5.34	0.00301	5.34	0.00331	5.34	0.00356
5.341	0.00223	5.341	0.00266	5.341	0.00301	5.341	0.0033	5.341	0.00356
5.342	0.00223	5.342	0.00266	5.342	0.00301	5.342	0.0033	5.342	0.00356
5.343	0.00223	5.343	0.00266	5.343	0.00301	5.343	0.0033	5.343	0.00355
5.344	0.00223	5.344	0.00266	5.344	0.00301	5.344	0.0033	5.344	0.00355
5.345	0.00223	5.345	0.00266	5.345	0.00301	5.345	0.0033	5.345	0.00355
5.346	0.00223	5.346	0.00266	5.346	0.00301	5.346	0.0033	5.346	0.00355
5.347	0.00223	5.347	0.00266	5.347	0.00301	5.347	0.0033	5.347	0.00355
5.348	0.00223	5.348	0.00266	5.348	0.00301	5.348	0.0033	5.348	0.00355
5.349	0.00223	5.349	0.00266	5.349	0.00301	5.349	0.0033	5.349	0.00355
5.35	0.00223	5.35	0.00266	5.35	0.00301	5.35	0.0033	5.35	0.00355
5.351	0.00223	5.351	0.00266	5.351	0.00301	5.351	0.0033	5.351	0.00355
5.352	0.00223	5.352	0.00266	5.352	0.00301	5.352	0.0033	5.352	0.00355
5.353	0.00223	5.353	0.00266	5.353	0.003	5.353	0.0033	5.353	0.00355
5.354	0.00223	5.354	0.00266	5.354	0.003	5.354	0.0033	5.354	0.00355
5.355	0.00223	5.355	0.00265	5.355	0.003	5.355	0.0033	5.355	0.00355
5.356	0.00223	5.356	0.00265	5.356	0.003	5.356	0.0033	5.356	0.00355
5.357	0.00223	5.357	0.00265	5.357	0.003	5.357	0.0033	5.357	0.00355
5.358	0.00223	5.358	0.00265	5.358	0.003	5.358	0.00329	5.358	0.00354
5.359	0.00223	5.359	0.00265	5.359	0.003	5.359	0.00329	5.359	0.00354
5.36	0.00223	5.36	0.00265	5.36	0.003	5.36	0.00329	5.36	0.00354
5.361	0.00223	5.361	0.00265	5.361	0.003	5.361	0.00329	5.361	0.00354
5.362	0.00222	5.362	0.00265	5.362	0.003	5.362	0.00329	5.362	0.00354
5.363	0.00222	5.363	0.00265	5.363	0.003	5.363	0.00329	5.363	0.00354
5.364	0.00222	5.364	0.00265	5.364	0.003	5.364	0.00329	5.364	0.00354
5.365	0.00222	5.365	0.00265	5.365	0.003	5.365	0.00329	5.365	0.00354
5.366	0.00222	5.366	0.00265	5.366	0.003	5.366	0.00329	5.366	0.00354
5.367	0.00222	5.367	0.00265	5.367	0.003	5.367	0.00329	5.367	0.00354
5.368	0.00222	5.368	0.00265	5.368	0.003	5.368	0.00329	5.368	0.00354
5.369	0.00222	5.369	0.00265	5.369	0.003	5.369	0.00329	5.369	0.00354
5.37	0.00222	5.37	0.00265	5.37	0.003	5.37	0.00329	5.37	0.00354
5.371	0.00222	5.371	0.00265	5.371	0.00299	5.371	0.00329	5.371	0.00354
5.372	0.00222	5.372	0.00265	5.372	0.00299	5.372	0.00329	5.372	0.00354
5.373	0.00222	5.373	0.00265	5.373	0.00299	5.373	0.00329	5.373	0.00353
5.374	0.00222	5.374	0.00265	5.374	0.00299	5.374	0.00328	5.374	0.00353
5.375	0.00222	5.375	0.00264	5.375	0.00299	5.375	0.00328	5.375	0.00353
5.376	0.00222	5.376	0.00264	5.376	0.00299	5.376	0.00328	5.376	0.00353
5.377	0.00222	5.377	0.00264	5.377	0.00299	5.377	0.00328	5.377	0.00353
5.378	0.00222	5.378	0.00264	5.378	0.00299	5.378	0.00328	5.378	0.00353
5.379	0.00222	5.379	0.00264	5.379	0.00299	5.379	0.00328	5.379	0.00353
5.38	0.00222	5.38	0.00264	5.38	0.00299	5.38	0.00328	5.38	0.00353
5.381	0.00222	5.381	0.00264	5.381	0.00299	5.381	0.00328	5.381	0.00353
5.382	0.00222	5.382	0.00264	5.382	0.00299	5.382	0.00328	5.382	0.00353
5.383	0.00222	5.383	0.00264	5.383	0.00299	5.383	0.00328	5.383	0.00353
5.384	0.00222	5.384	0.00264	5.384	0.00299	5.384	0.00328	5.384	0.00353
5.385	0.00222	5.385	0.00264	5.385	0.00299	5.385	0.00328	5.385	0.00353
5.386	0.00221	5.386	0.00264	5.386	0.00299	5.386	0.00328	5.386	0.00353
5.387	0.00221	5.387	0.00264	5.387	0.00299	5.387	0.00328	5.387	0.00353
5.388	0.00221	5.388	0.00264	5.388	0.00299	5.388	0.00328	5.388	0.00353
5.389	0.00221	5.389	0.00264	5.389	0.00298	5.389	0.00328	5.389	0.00352
5.39	0.00221	5.39	0.00264	5.39	0.00298	5.39	0.00327	5.39	0.00352
5.391	0.00221	5.391	0.00264	5.391	0.00298	5.391	0.00327	5.391	0.00352
5.392	0.00221	5.392	0.00264	5.392	0.00298	5.392	0.00327	5.392	0.00352
5.393	0.00221	5.393	0.00264	5.393	0.00298	5.393	0.00327	5.393	0.00352
5.394	0.00221	5.394	0.00264	5.394	0.00298	5.394	0.00327	5.394	0.00352
5.395	0.00221	5.395	0.00264	5.395	0.00298	5.395	0.00327	5.395	0.00352
5.396	0.00221	5.396	0.00263	5.396	0.00298	5.396	0.00327	5.396	0.00352
5.397	0.00221	5.397	0.00263	5.397	0.00298	5.397	0.00327	5.397	0.00352
5.398	0.00221	5.398	0.00263	5.398	0.00298	5.398	0.00327	5.398	0.00352
5.399	0.00221	5.399	0.00263	5.399	0.00298	5.399	0.00327	5.399	0.00352
5.4	0.00221	5.4	0.00263	5.4	0.00298	5.4	0.00327	5.4	0.00352
5.401	0.00221	5.401	0.00263	5.401	0.00298	5.401	0.00327	5.401	0.00352
5.402	0.00221	5.402	0.00263	5.402	0.00298	5.402	0.00327	5.402	0.00352
5.403	0.00221	5.403	0.00263	5.403	0.00298	5.403	0.00327	5.403	0.00352
5.404	0.00221	5.404	0.00263	5.404	0.00298	5.404	0.00327	5.404	0.00351
5.405	0.00221	5.405	0.00263	5.405	0.00298	5.405	0.00327	5.405	0.00351
5.406	0.00221	5.406	0.00263	5.406	0.00298	5.406	0.00327	5.406	0.00351
5.407	0.00221	5.407	0.00263	5.407	0.00297	5.407	0.00326	5.407	0.00351
5.408	0.00221	5.408	0.00263	5.408	0.00297	5.408	0.00326	5.408	0.00351
5.409	0.00221	5.409	0.00263	5.409	0.00297	5.409	0.00326	5.409	0.00351
5.41	0.00221	5.41	0.00263	5.41	0.00297	5.41	0.00326	5.41	0.00351
5.411	0.0022	5.411	0.00263	5.411	0.00297	5.411	0.00326	5.411	0.00351
5.412	0.0022	5.412	0.00263	5.412	0.00297	5.412	0.00326	5.412	0.00351
5.413	0.0022	5.413	0.00263	5.413	0.00297	5.413	0.00326	5.413	0.00351
5.414	0.0022	5.414	0.00263	5.414	0.00297	5.414	0.00326	5.414	0.00351
5.415	0.0022	5.415	0.00263	5.415	0.00297	5.415	0.00326	5.415	0.00351
5.416	0.0022	5.416	0.00262	5.416	0.00297	5.416	0.00326	5.416	0.00351
5.417	0.0022	5.417	0.00262	5.417	0.00297	5.417	0.00326	5.417	0.00351
5.418	0.0022	5.418	0.00262	5.418	0.00297	5.418	0.00326	5.418	0.00351
5.419	0.0022	5.419	0.00262	5.419	0.00297	5.419	0.00326	5.419	0.0035
5.42	0.0022	5.42	0.00262	5.42	0.00297	5.42	0.00326	5.42	0.0035
5.421	0.0022	5.421	0.00262	5.421	0.00297	5.421	0.00326	5.421	0.0035
5.422	0.0022	5.422	0.00262	5.422	0.00297	5.422	0.00326	5.422	0.0035
5.423	0.0022	5.423	0.00262	5.423	0.00297	5.423	0.00325	5.423	0.0035
5.424	0.0022	5.424	0.00262	5.424	0.00297	5.424	0.00325	5.424	0.0035
5.425	0.0022	5.425	0.00262	5.425	0.00297	5.425	0.00325	5.425	0.0035
5.426	0.0022	5.426	0.00262	5.426	0.00296	5.426	0.00325	5.426	0.0035
5.427	0.0022	5.427	0.00262	5.427	0.00296	5.427	0.00325	5.427	0.0035

5.428	0.0022	5.428	0.00262	5.428	0.00296	5.428	0.00325	5.428	0.0035
5.429	0.0022	5.429	0.00262	5.429	0.00296	5.429	0.00325	5.429	0.0035
5.43	0.0022	5.43	0.00262	5.43	0.00296	5.43	0.00325	5.43	0.0035
5.431	0.0022	5.431	0.00262	5.431	0.00296	5.431	0.00325	5.431	0.0035
5.432	0.0022	5.432	0.00262	5.432	0.00296	5.432	0.00325	5.432	0.0035
5.433	0.0022	5.433	0.00262	5.433	0.00296	5.433	0.00325	5.433	0.0035
5.434	0.0022	5.434	0.00262	5.434	0.00296	5.434	0.00325	5.434	0.0035
5.435	0.00219	5.435	0.00262	5.435	0.00296	5.435	0.00325	5.435	0.00349
5.436	0.00219	5.436	0.00262	5.436	0.00296	5.436	0.00325	5.436	0.00349
5.437	0.00219	5.437	0.00261	5.437	0.00296	5.437	0.00325	5.437	0.00349
5.438	0.00219	5.438	0.00261	5.438	0.00296	5.438	0.00325	5.438	0.00349
5.439	0.00219	5.439	0.00261	5.439	0.00296	5.439	0.00325	5.439	0.00349
5.44	0.00219	5.44	0.00261	5.44	0.00296	5.44	0.00324	5.44	0.00349
5.441	0.00219	5.441	0.00261	5.441	0.00296	5.441	0.00324	5.441	0.00349
5.442	0.00219	5.442	0.00261	5.442	0.00296	5.442	0.00324	5.442	0.00349
5.443	0.00219	5.443	0.00261	5.443	0.00296	5.443	0.00324	5.443	0.00349
5.444	0.00219	5.444	0.00261	5.444	0.00295	5.444	0.00324	5.444	0.00349
5.445	0.00219	5.445	0.00261	5.445	0.00295	5.445	0.00324	5.445	0.00349
5.446	0.00219	5.446	0.00261	5.446	0.00295	5.446	0.00324	5.446	0.00349
5.447	0.00219	5.447	0.00261	5.447	0.00295	5.447	0.00324	5.447	0.00349
5.448	0.00219	5.448	0.00261	5.448	0.00295	5.448	0.00324	5.448	0.00349
5.449	0.00219	5.449	0.00261	5.449	0.00295	5.449	0.00324	5.449	0.00349
5.45	0.00219	5.45	0.00261	5.45	0.00295	5.45	0.00324	5.45	0.00348
5.451	0.00219	5.451	0.00261	5.451	0.00295	5.451	0.00324	5.451	0.00348
5.452	0.00219	5.452	0.00261	5.452	0.00295	5.452	0.00324	5.452	0.00348
5.453	0.00219	5.453	0.00261	5.453	0.00295	5.453	0.00324	5.453	0.00348
5.454	0.00219	5.454	0.00261	5.454	0.00295	5.454	0.00324	5.454	0.00348
5.455	0.00219	5.455	0.00261	5.455	0.00295	5.455	0.00324	5.455	0.00348
5.456	0.00219	5.456	0.00261	5.456	0.00295	5.456	0.00324	5.456	0.00348
5.457	0.00219	5.457	0.00261	5.457	0.00295	5.457	0.00323	5.457	0.00348
5.458	0.00219	5.458	0.0026	5.458	0.00295	5.458	0.00323	5.458	0.00348
5.459	0.00219	5.459	0.0026	5.459	0.00295	5.459	0.00323	5.459	0.00348
5.46	0.00218	5.46	0.0026	5.46	0.00295	5.46	0.00323	5.46	0.00348
5.461	0.00218	5.461	0.0026	5.461	0.00295	5.461	0.00323	5.461	0.00348
5.462	0.00218	5.462	0.0026	5.462	0.00294	5.462	0.00323	5.462	0.00348
5.463	0.00218	5.463	0.0026	5.463	0.00294	5.463	0.00323	5.463	0.00348
5.464	0.00218	5.464	0.0026	5.464	0.00294	5.464	0.00323	5.464	0.00348
5.465	0.00218	5.465	0.0026	5.465	0.00294	5.465	0.00323	5.465	0.00348
5.466	0.00218	5.466	0.0026	5.466	0.00294	5.466	0.00323	5.466	0.00347
5.467	0.00218	5.467	0.0026	5.467	0.00294	5.467	0.00323	5.467	0.00347
5.468	0.00218	5.468	0.0026	5.468	0.00294	5.468	0.00323	5.468	0.00347
5.469	0.00218	5.469	0.0026	5.469	0.00294	5.469	0.00323	5.469	0.00347
5.47	0.00218	5.47	0.0026	5.47	0.00294	5.47	0.00323	5.47	0.00347
5.471	0.00218	5.471	0.0026	5.471	0.00294	5.471	0.00323	5.471	0.00347
5.472	0.00218	5.472	0.0026	5.472	0.00294	5.472	0.00323	5.472	0.00347
5.473	0.00218	5.473	0.0026	5.473	0.00294	5.473	0.00323	5.473	0.00347
5.474	0.00218	5.474	0.0026	5.474	0.00294	5.474	0.00322	5.474	0.00347
5.475	0.00218	5.475	0.0026	5.475	0.00294	5.475	0.00322	5.475	0.00347
5.476	0.00218	5.476	0.0026	5.476	0.00294	5.476	0.00322	5.476	0.00347
5.477	0.00218	5.477	0.0026	5.477	0.00294	5.477	0.00322	5.477	0.00347
5.478	0.00218	5.478	0.0026	5.478	0.00294	5.478	0.00322	5.478	0.00347
5.479	0.00218	5.479	0.00259	5.479	0.00294	5.479	0.00322	5.479	0.00347
5.48	0.00218	5.48	0.00259	5.48	0.00294	5.48	0.00322	5.48	0.00347
5.481	0.00218	5.481	0.00259	5.481	0.00293	5.481	0.00322	5.481	0.00347
5.482	0.00218	5.482	0.00259	5.482	0.00293	5.482	0.00322	5.482	0.00346
5.483	0.00218	5.483	0.00259	5.483	0.00293	5.483	0.00322	5.483	0.00346
5.484	0.00218	5.484	0.00259	5.484	0.00293	5.484	0.00322	5.484	0.00346
5.485	0.00217	5.485	0.00259	5.485	0.00293	5.485	0.00322	5.485	0.00346
5.486	0.00217	5.486	0.00259	5.486	0.00293	5.486	0.00322	5.486	0.00346
5.487	0.00217	5.487	0.00259	5.487	0.00293	5.487	0.00322	5.487	0.00346
5.488	0.00217	5.488	0.00259	5.488	0.00293	5.488	0.00322	5.488	0.00346
5.489	0.00217	5.489	0.00259	5.489	0.00293	5.489	0.00322	5.489	0.00346
5.49	0.00217	5.49	0.00259	5.49	0.00293	5.49	0.00322	5.49	0.00346
5.491	0.00217	5.491	0.00259	5.491	0.00293	5.491	0.00321	5.491	0.00346
5.492	0.00217	5.492	0.00259	5.492	0.00293	5.492	0.00321	5.492	0.00346
5.493	0.00217	5.493	0.00259	5.493	0.00293	5.493	0.00321	5.493	0.00346
5.494	0.00217	5.494	0.00259	5.494	0.00293	5.494	0.00321	5.494	0.00346
5.495	0.00217	5.495	0.00259	5.495	0.00293	5.495	0.00321	5.495	0.00346
5.496	0.00217	5.496	0.00259	5.496	0.00293	5.496	0.00321	5.496	0.00346
5.497	0.00217	5.497	0.00259	5.497	0.00293	5.497	0.00321	5.497	0.00346
5.498	0.00217	5.498	0.00259	5.498	0.00293	5.498	0.00321	5.498	0.00345
5.499	0.00217	5.499	0.00259	5.499	0.00293	5.499	0.00321	5.499	0.00345
5.5	0.00217	5.5	0.00258	5.5	0.00292	5.5	0.00321	5.5	0.00345
5.501	0.00217	5.501	0.00258	5.501	0.00292	5.501	0.00321	5.501	0.00345
5.502	0.00217	5.502	0.00258	5.502	0.00292	5.502	0.00321	5.502	0.00345
5.503	0.00217	5.503	0.00258	5.503	0.00292	5.503	0.00321	5.503	0.00345
5.504	0.00217	5.504	0.00258	5.504	0.00292	5.504	0.00321	5.504	0.00345
5.505	0.00217	5.505	0.00258	5.505	0.00292	5.505	0.00321	5.505	0.00345
5.506	0.00217	5.506	0.00258	5.506	0.00292	5.506	0.00321	5.506	0.00345
5.507	0.00217	5.507	0.00258	5.507	0.00292	5.507	0.00321	5.507	0.00345
5.508	0.00217	5.508	0.00258	5.508	0.00292	5.508	0.0032	5.508	0.00345
5.509	0.00217	5.509	0.00258	5.509	0.00292	5.509	0.0032	5.509	0.00345
5.51	0.00217	5.51	0.00258	5.51	0.00292	5.51	0.0032	5.51	0.00345
5.511	0.00216	5.511	0.00258	5.511	0.00292	5.511	0.0032	5.511	0.00345
5.512	0.00216	5.512	0.00258	5.512	0.00292	5.512	0.0032	5.512	0.00345
5.513	0.00216	5.513	0.00258	5.513	0.00292	5.513	0.0032	5.513	0.00345
5.514	0.00216	5.514	0.00258	5.514	0.00292	5.514	0.0032	5.514	0.00344
5.515	0.00216	5.515	0.00258	5.515	0.00292	5.515	0.0032	5.515	0.00344
5.516	0.00216	5.516	0.00258	5.516	0.00292	5.516	0.0032	5.516	0.00344
5.517	0.00216	5.517	0.00258	5.517	0.00292	5.517	0.0032	5.517	0.00344
5.518	0.00216	5.518	0.00258	5.518	0.00292	5.518	0.0032	5.518	0.00344
5.519	0.00216	5.519	0.00258	5.519	0.00291	5.519	0.0032	5.519	0.00344
5.52	0.00216	5.52	0.00258	5.52	0.00291	5.52	0.0032	5.52	0.00344
5.521	0.00216	5.521	0.00257	5.521	0.00291	5.521	0.0032	5.521	0.00344
5.522	0.00216	5.522	0.00257	5.522	0.00291	5.522	0.0032	5.522	0.00344
5.523	0.00216	5.523	0.00257	5.523	0.00291	5.523	0.0032	5.523	0.00344
5.524	0.00216	5.524	0.00257	5.524	0.00291	5.524	0.0032	5.524	0.00344
5.525	0.00216	5.525	0.00257	5.525	0.00291	5.525	0.00319	5.525	0.00344
5.526	0.00216	5.526	0.00257	5.526	0.00291	5.526	0.00319	5.526	0.00344
5.527	0.00216	5.527	0.00257	5.527	0.00291	5.527	0.00319	5.527	0.00344
5.528	0.00216	5.528	0.00257	5.528	0.00291	5.528	0.00319	5.528	0.00344
5.529	0.00216	5.529	0.00257	5.529	0.00291	5.529	0.00319	5.529	0.00344
5.53	0.00216	5.53	0.00257	5.53	0.00291	5.53	0.00319	5.53	0.00343
5.531	0.00216	5.531	0.00257	5.531	0.00291	5.531	0.00319	5.531	0.00343
5.532	0.00216	5.532	0.00257	5.532	0.00291	5.532	0.00319	5.532	0.00343
5.533	0.00216	5.533	0.00257	5.533	0.00291	5.533	0.00319	5.533	0.00343
5.534	0.00216	5.534	0.00257	5.534	0.00291	5.534	0.00319	5.534	0.00343
5.535	0.00216	5.535	0.00257	5.535	0.00291	5.535	0.00319	5.535	0.00343
5.536	0.00215	5.536	0.00257	5.536	0.00291	5.536	0.00319	5.536	0.00343
5.537	0.00215	5.537	0.00257	5.537	0.00291	5.537	0.00319	5.537	0.00343
5.538	0.00215	5.538	0.00257	5.538	0.0029	5.538	0.00319	5.538	0.00343
5.539	0.00215	5.539	0.00257	5.539	0.0029	5.539	0.00319	5.539	0.00343
5.54	0.00215	5.54	0.00257	5.54	0.0029	5.54	0.00319	5.54	0.00343
5.541	0.00215	5.541	0.00257	5.541	0.0029	5.541	0.00319	5.541	0.00343
5.542	0.00215	5.542	0.00257	5.542	0.0029	5.542	0.00319	5.542	0.00343
5.543	0.00215	5.543	0.00256	5.543	0.0029	5.543	0.00318	5.543	0.00343
5.544	0.00215	5.544	0.00256	5.544	0.0029	5.544	0.00318	5.544	0.00343
5.545	0.00215	5.545	0.00256	5.545	0.0029	5.545	0.00318	5.545	0.00343
5.546	0.00215	5.546	0.00256	5.546	0.0029	5.546	0.00318	5.546	0.00342
5.547	0.00215	5.547	0.00256	5.547	0.0029	5.547	0.00318	5.547	0.00342
5.548	0.00215	5.548	0.00256	5.548	0.0029	5.548	0.00318	5.548	0.00342
5.549	0.00215	5.549	0.00256	5.549	0.0029	5.549	0.00318	5.549	0.00342
5.55	0.00215	5.55	0.00256	5.55	0.0029	5.55	0.00318	5.55	0.00342
5.551	0.00215	5.551	0.00256	5.551	0.0029	5.551	0.00318	5.551	0.00342
5.552	0.00215	5.552	0.00256	5.552	0.0029	5.552	0.00318	5.552	0.00342
5.553	0.00215	5.553	0.00256	5.553	0.0029	5.553	0.00318	5.553	0.00342
5.554	0.00215	5.554	0.00256	5.554	0.0029	5.554	0.00318	5.554	0.00342
5.555	0.00215	5.555	0.00256	5.555	0.0029	5.555	0.00318	5.555	0.00342
5.556	0.00215	5.556	0.00256	5.556	0.0029	5.556	0.00318	5.556	0.00342
5.557	0.00215	5.557	0.00256	5.557	0.00289	5.557	0.00318	5.557	0.00342
5.558	0.00215	5.558	0.00256	5.558	0.00289	5.558	0.00318	5.558	0.00342
5.559	0.00215	5.559	0.00256	5.559	0.00289	5.559	0.00318	5.559	0.00342
5.56	0.00215	5.56	0.00256	5.56	0.00289	5.56	0.00317	5.56	0.00342
5.561	0.00215	5.561	0.00256	5.561	0.00289	5.561	0.00317	5.561	0.00342
5.562	0.00214	5.562	0.00256	5.562	0.00289	5.562	0.00317	5.562	0.00341

5.563	0.00214	5.563	0.00256	5.563	0.00289	5.563	0.00317	5.563	0.00341
5.564	0.00214	5.564	0.00256	5.564	0.00289	5.564	0.00317	5.564	0.00341
5.565	0.00214	5.565	0.00255	5.565	0.00289	5.565	0.00317	5.565	0.00341
5.566	0.00214	5.566	0.00255	5.566	0.00289	5.566	0.00317	5.566	0.00341
5.567	0.00214	5.567	0.00255	5.567	0.00289	5.567	0.00317	5.567	0.00341
5.568	0.00214	5.568	0.00255	5.568	0.00289	5.568	0.00317	5.568	0.00341
5.569	0.00214	5.569	0.00255	5.569	0.00289	5.569	0.00317	5.569	0.00341
5.57	0.00214	5.57	0.00255	5.57	0.00289	5.57	0.00317	5.57	0.00341
5.571	0.00214	5.571	0.00255	5.571	0.00289	5.571	0.00317	5.571	0.00341
5.572	0.00214	5.572	0.00255	5.572	0.00289	5.572	0.00317	5.572	0.00341
5.573	0.00214	5.573	0.00255	5.573	0.00289	5.573	0.00317	5.573	0.00341
5.574	0.00214	5.574	0.00255	5.574	0.00289	5.574	0.00317	5.574	0.00341
5.575	0.00214	5.575	0.00255	5.575	0.00289	5.575	0.00317	5.575	0.00341
5.576	0.00214	5.576	0.00255	5.576	0.00288	5.576	0.00317	5.576	0.00341
5.577	0.00214	5.577	0.00255	5.577	0.00288	5.577	0.00317	5.577	0.00341
5.578	0.00214	5.578	0.00255	5.578	0.00288	5.578	0.00316	5.578	0.0034
5.579	0.00214	5.579	0.00255	5.579	0.00288	5.579	0.00316	5.579	0.0034
5.58	0.00214	5.58	0.00255	5.58	0.00288	5.58	0.00316	5.58	0.0034
5.581	0.00214	5.581	0.00255	5.581	0.00288	5.581	0.00316	5.581	0.0034
5.582	0.00214	5.582	0.00255	5.582	0.00288	5.582	0.00316	5.582	0.0034
5.583	0.00214	5.583	0.00255	5.583	0.00288	5.583	0.00316	5.583	0.0034
5.584	0.00214	5.584	0.00255	5.584	0.00288	5.584	0.00316	5.584	0.0034
5.585	0.00214	5.585	0.00255	5.585	0.00288	5.585	0.00316	5.585	0.0034
5.586	0.00214	5.586	0.00255	5.586	0.00288	5.586	0.00316	5.586	0.0034
5.587	0.00214	5.587	0.00254	5.587	0.00288	5.587	0.00316	5.587	0.0034
5.588	0.00213	5.588	0.00254	5.588	0.00288	5.588	0.00316	5.588	0.0034
5.589	0.00213	5.589	0.00254	5.589	0.00288	5.589	0.00316	5.589	0.0034
5.59	0.00213	5.59	0.00254	5.59	0.00288	5.59	0.00316	5.59	0.0034
5.591	0.00213	5.591	0.00254	5.591	0.00288	5.591	0.00316	5.591	0.0034
5.592	0.00213	5.592	0.00254	5.592	0.00288	5.592	0.00316	5.592	0.0034
5.593	0.00213	5.593	0.00254	5.593	0.00288	5.593	0.00316	5.593	0.0034
5.594	0.00213	5.594	0.00254	5.594	0.00288	5.594	0.00316	5.594	0.0034
5.595	0.00213	5.595	0.00254	5.595	0.00287	5.595	0.00315	5.595	0.00339
5.596	0.00213	5.596	0.00254	5.596	0.00287	5.596	0.00315	5.596	0.00339
5.597	0.00213	5.597	0.00254	5.597	0.00287	5.597	0.00315	5.597	0.00339
5.598	0.00213	5.598	0.00254	5.598	0.00287	5.598	0.00315	5.598	0.00339
5.599	0.00213	5.599	0.00254	5.599	0.00287	5.599	0.00315	5.599	0.00339
5.6	0.00213	5.6	0.00254	5.6	0.00287	5.6	0.00315	5.6	0.00339
5.601	0.00213	5.601	0.00254	5.601	0.00287	5.601	0.00315	5.601	0.00339
5.602	0.00213	5.602	0.00254	5.602	0.00287	5.602	0.00315	5.602	0.00339
5.603	0.00213	5.603	0.00254	5.603	0.00287	5.603	0.00315	5.603	0.00339
5.604	0.00213	5.604	0.00254	5.604	0.00287	5.604	0.00315	5.604	0.00339
5.605	0.00213	5.605	0.00254	5.605	0.00287	5.605	0.00315	5.605	0.00339
5.606	0.00213	5.606	0.00254	5.606	0.00287	5.606	0.00315	5.606	0.00339
5.607	0.00213	5.607	0.00254	5.607	0.00287	5.607	0.00315	5.607	0.00339
5.608	0.00213	5.608	0.00254	5.608	0.00287	5.608	0.00315	5.608	0.00339
5.609	0.00213	5.609	0.00253	5.609	0.00287	5.609	0.00315	5.609	0.00339
5.61	0.00213	5.61	0.00253	5.61	0.00287	5.61	0.00315	5.61	0.00339
5.611	0.00213	5.611	0.00253	5.611	0.00287	5.611	0.00315	5.611	0.00338
5.612	0.00213	5.612	0.00253	5.612	0.00287	5.612	0.00315	5.612	0.00338
5.613	0.00213	5.613	0.00253	5.613	0.00287	5.613	0.00314	5.613	0.00338
5.614	0.00212	5.614	0.00253	5.614	0.00287	5.614	0.00314	5.614	0.00338
5.615	0.00212	5.615	0.00253	5.615	0.00286	5.615	0.00314	5.615	0.00338
5.616	0.00212	5.616	0.00253	5.616	0.00286	5.616	0.00314	5.616	0.00338
5.617	0.00212	5.617	0.00253	5.617	0.00286	5.617	0.00314	5.617	0.00338
5.618	0.00212	5.618	0.00253	5.618	0.00286	5.618	0.00314	5.618	0.00338
5.619	0.00212	5.619	0.00253	5.619	0.00286	5.619	0.00314	5.619	0.00338
5.62	0.00212	5.62	0.00253	5.62	0.00286	5.62	0.00314	5.62	0.00338
5.621	0.00212	5.621	0.00253	5.621	0.00286	5.621	0.00314	5.621	0.00338
5.622	0.00212	5.622	0.00253	5.622	0.00286	5.622	0.00314	5.622	0.00338
5.623	0.00212	5.623	0.00253	5.623	0.00286	5.623	0.00314	5.623	0.00338
5.624	0.00212	5.624	0.00253	5.624	0.00286	5.624	0.00314	5.624	0.00338
5.625	0.00212	5.625	0.00253	5.625	0.00286	5.625	0.00314	5.625	0.00338
5.626	0.00212	5.626	0.00253	5.626	0.00286	5.626	0.00314	5.626	0.00338
5.627	0.00212	5.627	0.00253	5.627	0.00286	5.627	0.00314	5.627	0.00338
5.628	0.00212	5.628	0.00253	5.628	0.00286	5.628	0.00314	5.628	0.00337
5.629	0.00212	5.629	0.00253	5.629	0.00286	5.629	0.00314	5.629	0.00337
5.63	0.00212	5.63	0.00253	5.63	0.00286	5.63	0.00314	5.63	0.00337
5.631	0.00212	5.631	0.00252	5.631	0.00286	5.631	0.00313	5.631	0.00337
5.632	0.00212	5.632	0.00252	5.632	0.00286	5.632	0.00313	5.632	0.00337
5.633	0.00212	5.633	0.00252	5.633	0.00286	5.633	0.00313	5.633	0.00337
5.634	0.00212	5.634	0.00252	5.634	0.00286	5.634	0.00313	5.634	0.00337
5.635	0.00212	5.635	0.00252	5.635	0.00285	5.635	0.00313	5.635	0.00337
5.636	0.00212	5.636	0.00252	5.636	0.00285	5.636	0.00313	5.636	0.00337
5.637	0.00212	5.637	0.00252	5.637	0.00285	5.637	0.00313	5.637	0.00337
5.638	0.00212	5.638	0.00252	5.638	0.00285	5.638	0.00313	5.638	0.00337
5.639	0.00212	5.639	0.00252	5.639	0.00285	5.639	0.00313	5.639	0.00337
5.64	0.00212	5.64	0.00252	5.64	0.00285	5.64	0.00313	5.64	0.00337
5.641	0.00211	5.641	0.00252	5.641	0.00285	5.641	0.00313	5.641	0.00337
5.642	0.00211	5.642	0.00252	5.642	0.00285	5.642	0.00313	5.642	0.00337
5.643	0.00211	5.643	0.00252	5.643	0.00285	5.643	0.00313	5.643	0.00337
5.644	0.00211	5.644	0.00252	5.644	0.00285	5.644	0.00313	5.644	0.00337
5.645	0.00211	5.645	0.00252	5.645	0.00285	5.645	0.00313	5.645	0.00336
5.646	0.00211	5.646	0.00252	5.646	0.00285	5.646	0.00313	5.646	0.00336
5.647	0.00211	5.647	0.00252	5.647	0.00285	5.647	0.00313	5.647	0.00336
5.648	0.00211	5.648	0.00252	5.648	0.00285	5.648	0.00313	5.648	0.00336
5.649	0.00211	5.649	0.00252	5.649	0.00285	5.649	0.00312	5.649	0.00336
5.65	0.00211	5.65	0.00252	5.65	0.00285	5.65	0.00312	5.65	0.00336
5.651	0.00211	5.651	0.00252	5.651	0.00285	5.651	0.00312	5.651	0.00336
5.652	0.00211	5.652	0.00252	5.652	0.00285	5.652	0.00312	5.652	0.00336
5.653	0.00211	5.653	0.00251	5.653	0.00285	5.653	0.00312	5.653	0.00336
5.654	0.00211	5.654	0.00251	5.654	0.00284	5.654	0.00312	5.654	0.00336
5.655	0.00211	5.655	0.00251	5.655	0.00284	5.655	0.00312	5.655	0.00336
5.656	0.00211	5.656	0.00251	5.656	0.00284	5.656	0.00312	5.656	0.00336
5.657	0.00211	5.657	0.00251	5.657	0.00284	5.657	0.00312	5.657	0.00336
5.658	0.00211	5.658	0.00251	5.658	0.00284	5.658	0.00312	5.658	0.00336
5.659	0.00211	5.659	0.00251	5.659	0.00284	5.659	0.00312	5.659	0.00336
5.66	0.00211	5.66	0.00251	5.66	0.00284	5.66	0.00312	5.66	0.00336
5.661	0.00211	5.661	0.00251	5.661	0.00284	5.661	0.00312	5.661	0.00336
5.662	0.00211	5.662	0.00251	5.662	0.00284	5.662	0.00312	5.662	0.00335
5.663	0.00211	5.663	0.00251	5.663	0.00284	5.663	0.00312	5.663	0.00335
5.664	0.00211	5.664	0.00251	5.664	0.00284	5.664	0.00312	5.664	0.00335
5.665	0.00211	5.665	0.00251	5.665	0.00284	5.665	0.00312	5.665	0.00335
5.666	0.00211	5.666	0.00251	5.666	0.00284	5.666	0.00312	5.666	0.00335
5.667	0.00211	5.667	0.00251	5.667	0.00284	5.667	0.00311	5.667	0.00335
5.668	0.0021	5.668	0.00251	5.668	0.00284	5.668	0.00311	5.668	0.00335
5.669	0.0021	5.669	0.00251	5.669	0.00284	5.669	0.00311	5.669	0.00335
5.67	0.0021	5.67	0.00251	5.67	0.00284	5.67	0.00311	5.67	0.00335
5.671	0.0021	5.671	0.00251	5.671	0.00284	5.671	0.00311	5.671	0.00335
5.672	0.0021	5.672	0.00251	5.672	0.00284	5.672	0.00311	5.672	0.00335
5.673	0.0021	5.673	0.00251	5.673	0.00284	5.673	0.00311	5.673	0.00335
5.674	0.0021	5.674	0.00251	5.674	0.00283	5.674	0.00311	5.674	0.00335
5.675	0.0021	5.675	0.00251	5.675	0.00283	5.675	0.00311	5.675	0.00335
5.676	0.0021	5.676	0.0025	5.676	0.00283	5.676	0.00311	5.676	0.00335
5.677	0.0021	5.677	0.0025	5.677	0.00283	5.677	0.00311	5.677	0.00335
5.678	0.0021	5.678	0.0025	5.678	0.00283	5.678	0.00311	5.678	0.00334
5.679	0.0021	5.679	0.0025	5.679	0.00283	5.679	0.00311	5.679	0.00334
5.68	0.0021	5.68	0.0025	5.68	0.00283	5.68	0.00311	5.68	0.00334
5.681	0.0021	5.681	0.0025	5.681	0.00283	5.681	0.00311	5.681	0.00334
5.682	0.0021	5.682	0.0025	5.682	0.00283	5.682	0.00311	5.682	0.00334
5.683	0.0021	5.683	0.0025	5.683	0.00283	5.683	0.00311	5.683	0.00334
5.684	0.0021	5.684	0.0025	5.684	0.00283	5.684	0.00311	5.684	0.00334
5.685	0.0021	5.685	0.0025	5.685	0.00283	5.685	0.0031	5.685	0.00334
5.686	0.0021	5.686	0.0025	5.686	0.00283	5.686	0.0031	5.686	0.00334
5.687	0.0021	5.687	0.0025	5.687	0.00283	5.687	0.0031	5.687	0.00334
5.688	0.0021	5.688	0.0025	5.688	0.00283	5.688	0.0031	5.688	0.00334
5.689	0.0021	5.689	0.0025	5.689	0.00283	5.689	0.0031	5.689	0.00334
5.69	0.0021	5.69	0.0025	5.69	0.00283	5.69	0.0031	5.69	0.00334
5.691	0.0021	5.691	0.0025	5.691	0.00283	5.691	0.0031	5.691	0.00334
5.692	0.0021	5.692	0.0025	5.692	0.00283	5.692	0.0031	5.692	0.00334
5.693	0.0021	5.693	0.0025	5.693	0.00283	5.693	0.0031	5.693	0.00334
5.694	0.0021	5.694	0.0025	5.694	0.00282	5.694	0.0031	5.694	0.00334
5.695	0.00209	5.695	0.0025	5.695	0.00282	5.695	0.0031	5.695	0.00334
5.696	0.00209	5.696	0.0025	5.696	0.00282	5.696	0.0031	5.696	0.00333
5.697	0.00209	5.697	0.0025	5.697	0.00282	5.697	0.0031	5.697	0.00333

5.698	0.00209	5.698	0.0025	5.698	0.00282	5.698	0.0031	5.698	0.00333
5.699	0.00209	5.699	0.00249	5.699	0.00282	5.699	0.0031	5.699	0.00333
5.7	0.00209	5.7	0.00249	5.7	0.00282	5.7	0.0031	5.7	0.00333
5.701	0.00209	5.701	0.00249	5.701	0.00282	5.701	0.0031	5.701	0.00333
5.702	0.00209	5.702	0.00249	5.702	0.00282	5.702	0.0031	5.702	0.00333
5.703	0.00209	5.703	0.00249	5.703	0.00282	5.703	0.0031	5.703	0.00333
5.704	0.00209	5.704	0.00249	5.704	0.00282	5.704	0.00309	5.704	0.00333
5.705	0.00209	5.705	0.00249	5.705	0.00282	5.705	0.00309	5.705	0.00333
5.706	0.00209	5.706	0.00249	5.706	0.00282	5.706	0.00309	5.706	0.00333
5.707	0.00209	5.707	0.00249	5.707	0.00282	5.707	0.00309	5.707	0.00333
5.708	0.00209	5.708	0.00249	5.708	0.00282	5.708	0.00309	5.708	0.00333
5.709	0.00209	5.709	0.00249	5.709	0.00282	5.709	0.00309	5.709	0.00333
5.71	0.00209	5.71	0.00249	5.71	0.00282	5.71	0.00309	5.71	0.00333
5.711	0.00209	5.711	0.00249	5.711	0.00282	5.711	0.00309	5.711	0.00333
5.712	0.00209	5.712	0.00249	5.712	0.00282	5.712	0.00309	5.712	0.00333
5.713	0.00209	5.713	0.00249	5.713	0.00282	5.713	0.00309	5.713	0.00332
5.714	0.00209	5.714	0.00249	5.714	0.00282	5.714	0.00309	5.714	0.00332
5.715	0.00209	5.715	0.00249	5.715	0.00281	5.715	0.00309	5.715	0.00332
5.716	0.00209	5.716	0.00249	5.716	0.00281	5.716	0.00309	5.716	0.00332
5.717	0.00209	5.717	0.00249	5.717	0.00281	5.717	0.00309	5.717	0.00332
5.718	0.00209	5.718	0.00249	5.718	0.00281	5.718	0.00309	5.718	0.00332
5.719	0.00209	5.719	0.00249	5.719	0.00281	5.719	0.00309	5.719	0.00332
5.72	0.00209	5.72	0.00249	5.72	0.00281	5.72	0.00309	5.72	0.00332
5.721	0.00209	5.721	0.00248	5.721	0.00281	5.721	0.00309	5.721	0.00332
5.722	0.00208	5.722	0.00248	5.722	0.00281	5.722	0.00308	5.722	0.00332
5.723	0.00208	5.723	0.00248	5.723	0.00281	5.723	0.00308	5.723	0.00332
5.724	0.00208	5.724	0.00248	5.724	0.00281	5.724	0.00308	5.724	0.00332
5.725	0.00208	5.725	0.00248	5.725	0.00281	5.725	0.00308	5.725	0.00332
5.726	0.00208	5.726	0.00248	5.726	0.00281	5.726	0.00308	5.726	0.00332
5.727	0.00208	5.727	0.00248	5.727	0.00281	5.727	0.00308	5.727	0.00332
5.728	0.00208	5.728	0.00248	5.728	0.00281	5.728	0.00308	5.728	0.00332
5.729	0.00208	5.729	0.00248	5.729	0.00281	5.729	0.00308	5.729	0.00332
5.73	0.00208	5.73	0.00248	5.73	0.00281	5.73	0.00308	5.73	0.00331
5.731	0.00208	5.731	0.00248	5.731	0.00281	5.731	0.00308	5.731	0.00331
5.732	0.00208	5.732	0.00248	5.732	0.00281	5.732	0.00308	5.732	0.00331
5.733	0.00208	5.733	0.00248	5.733	0.00281	5.733	0.00308	5.733	0.00331
5.734	0.00208	5.734	0.00248	5.734	0.00281	5.734	0.00308	5.734	0.00331
5.735	0.00208	5.735	0.00248	5.735	0.0028	5.735	0.00308	5.735	0.00331
5.736	0.00208	5.736	0.00248	5.736	0.0028	5.736	0.00308	5.736	0.00331
5.737	0.00208	5.737	0.00248	5.737	0.0028	5.737	0.00308	5.737	0.00331
5.738	0.00208	5.738	0.00248	5.738	0.0028	5.738	0.00308	5.738	0.00331
5.739	0.00208	5.739	0.00248	5.739	0.0028	5.739	0.00308	5.739	0.00331
5.74	0.00208	5.74	0.00248	5.74	0.0028	5.74	0.00308	5.74	0.00331
5.741	0.00208	5.741	0.00248	5.741	0.0028	5.741	0.00307	5.741	0.00331
5.742	0.00208	5.742	0.00248	5.742	0.0028	5.742	0.00307	5.742	0.00331
5.743	0.00208	5.743	0.00248	5.743	0.0028	5.743	0.00307	5.743	0.00331
5.744	0.00208	5.744	0.00248	5.744	0.0028	5.744	0.00307	5.744	0.00331
5.745	0.00208	5.745	0.00247	5.745	0.0028	5.745	0.00307	5.745	0.00331
5.746	0.00208	5.746	0.00247	5.746	0.0028	5.746	0.00307	5.746	0.00331
5.747	0.00208	5.747	0.00247	5.747	0.0028	5.747	0.00307	5.747	0.0033
5.748	0.00208	5.748	0.00247	5.748	0.0028	5.748	0.00307	5.748	0.0033
5.749	0.00208	5.749	0.00247	5.749	0.0028	5.749	0.00307	5.749	0.0033
5.75	0.00207	5.75	0.00247	5.75	0.0028	5.75	0.00307	5.75	0.0033
5.751	0.00207	5.751	0.00247	5.751	0.0028	5.751	0.00307	5.751	0.0033
5.752	0.00207	5.752	0.00247	5.752	0.0028	5.752	0.00307	5.752	0.0033
5.753	0.00207	5.753	0.00247	5.753	0.0028	5.753	0.00307	5.753	0.0033
5.754	0.00207	5.754	0.00247	5.754	0.0028	5.754	0.00307	5.754	0.0033
5.755	0.00207	5.755	0.00247	5.755	0.00279	5.755	0.00307	5.755	0.0033
5.756	0.00207	5.756	0.00247	5.756	0.00279	5.756	0.00307	5.756	0.0033
5.757	0.00207	5.757	0.00247	5.757	0.00279	5.757	0.00307	5.757	0.0033
5.758	0.00207	5.758	0.00247	5.758	0.00279	5.758	0.00307	5.758	0.0033
5.759	0.00207	5.759	0.00247	5.759	0.00279	5.759	0.00307	5.759	0.0033
5.76	0.00207	5.76	0.00247	5.76	0.00279	5.76	0.00306	5.76	0.0033
5.761	0.00207	5.761	0.00247	5.761	0.00279	5.761	0.00306	5.761	0.0033
5.762	0.00207	5.762	0.00247	5.762	0.00279	5.762	0.00306	5.762	0.0033
5.763	0.00207	5.763	0.00247	5.763	0.00279	5.763	0.00306	5.763	0.0033
5.764	0.00207	5.764	0.00247	5.764	0.00279	5.764	0.00306	5.764	0.0033
5.765	0.00207	5.765	0.00247	5.765	0.00279	5.765	0.00306	5.765	0.00329
5.766	0.00207	5.766	0.00247	5.766	0.00279	5.766	0.00306	5.766	0.00329
5.767	0.00207	5.767	0.00247	5.767	0.00279	5.767	0.00306	5.767	0.00329
5.768	0.00207	5.768	0.00246	5.768	0.00279	5.768	0.00306	5.768	0.00329
5.769	0.00207	5.769	0.00246	5.769	0.00279	5.769	0.00306	5.769	0.00329
5.77	0.00207	5.77	0.00246	5.77	0.00279	5.77	0.00306	5.77	0.00329
5.771	0.00207	5.771	0.00246	5.771	0.00279	5.771	0.00306	5.771	0.00329
5.772	0.00207	5.772	0.00246	5.772	0.00279	5.772	0.00306	5.772	0.00329
5.773	0.00207	5.773	0.00246	5.773	0.00279	5.773	0.00306	5.773	0.00329
5.774	0.00207	5.774	0.00246	5.774	0.00279	5.774	0.00306	5.774	0.00329
5.775	0.00207	5.775	0.00246	5.775	0.00279	5.775	0.00306	5.775	0.00329
5.776	0.00207	5.776	0.00246	5.776	0.00278	5.776	0.00306	5.776	0.00329
5.777	0.00207	5.777	0.00246	5.777	0.00278	5.777	0.00306	5.777	0.00329
5.778	0.00206	5.778	0.00246	5.778	0.00278	5.778	0.00305	5.778	0.00329
5.779	0.00206	5.779	0.00246	5.779	0.00278	5.779	0.00305	5.779	0.00329
5.78	0.00206	5.78	0.00246	5.78	0.00278	5.78	0.00305	5.78	0.00329
5.781	0.00206	5.781	0.00246	5.781	0.00278	5.781	0.00305	5.781	0.00329
5.782	0.00206	5.782	0.00246	5.782	0.00278	5.782	0.00305	5.782	0.00328
5.783	0.00206	5.783	0.00246	5.783	0.00278	5.783	0.00305	5.783	0.00328
5.784	0.00206	5.784	0.00246	5.784	0.00278	5.784	0.00305	5.784	0.00328
5.785	0.00206	5.785	0.00246	5.785	0.00278	5.785	0.00305	5.785	0.00328
5.786	0.00206	5.786	0.00246	5.786	0.00278	5.786	0.00305	5.786	0.00328
5.787	0.00206	5.787	0.00246	5.787	0.00278	5.787	0.00305	5.787	0.00328
5.788	0.00206	5.788	0.00246	5.788	0.00278	5.788	0.00305	5.788	0.00328
5.789	0.00206	5.789	0.00246	5.789	0.00278	5.789	0.00305	5.789	0.00328
5.79	0.00206	5.79	0.00246	5.79	0.00278	5.79	0.00305	5.79	0.00328
5.791	0.00206	5.791	0.00245	5.791	0.00278	5.791	0.00305	5.791	0.00328
5.792	0.00206	5.792	0.00245	5.792	0.00278	5.792	0.00305	5.792	0.00328
5.793	0.00206	5.793	0.00245	5.793	0.00278	5.793	0.00305	5.793	0.00328
5.794	0.00206	5.794	0.00245	5.794	0.00278	5.794	0.00305	5.794	0.00328
5.795	0.00206	5.795	0.00245	5.795	0.00278	5.795	0.00305	5.795	0.00328
5.796	0.00206	5.796	0.00245	5.796	0.00278	5.796	0.00305	5.796	0.00328
5.797	0.00206	5.797	0.00245	5.797	0.00277	5.797	0.00304	5.797	0.00328
5.798	0.00206	5.798	0.00245	5.798	0.00277	5.798	0.00304	5.798	0.00328
5.799	0.00206	5.799	0.00245	5.799	0.00277	5.799	0.00304	5.799	0.00328
5.8	0.00206	5.8	0.00245	5.8	0.00277	5.8	0.00304	5.8	0.00327
5.801	0.00206	5.801	0.00245	5.801	0.00277	5.801	0.00304	5.801	0.00327
5.802	0.00206	5.802	0.00245	5.802	0.00277	5.802	0.00304	5.802	0.00327
5.803	0.00206	5.803	0.00245	5.803	0.00277	5.803	0.00304	5.803	0.00327
5.804	0.00206	5.804	0.00245	5.804	0.00277	5.804	0.00304	5.804	0.00327
5.805	0.00206	5.805	0.00245	5.805	0.00277	5.805	0.00304	5.805	0.00327
5.806	0.00205	5.806	0.00245	5.806	0.00277	5.806	0.00304	5.806	0.00327
5.807	0.00205	5.807	0.00245	5.807	0.00277	5.807	0.00304	5.807	0.00327
5.808	0.00205	5.808	0.00245	5.808	0.00277	5.808	0.00304	5.808	0.00327
5.809	0.00205	5.809	0.00245	5.809	0.00277	5.809	0.00304	5.809	0.00327
5.81	0.00205	5.81	0.00245	5.81	0.00277	5.81	0.00304	5.81	0.00327
5.811	0.00205	5.811	0.00245	5.811	0.00277	5.811	0.00304	5.811	0.00327
5.812	0.00205	5.812	0.00245	5.812	0.00277	5.812	0.00304	5.812	0.00327
5.813	0.00205	5.813	0.00245	5.813	0.00277	5.813	0.00304	5.813	0.00327
5.814	0.00205	5.814	0.00245	5.814	0.00277	5.814	0.00304	5.814	0.00327
5.815	0.00205	5.815	0.00244	5.815	0.00277	5.815	0.00304	5.815	0.00327
5.816	0.00205	5.816	0.00244	5.816	0.00277	5.816	0.00303	5.816	0.00327
5.817	0.00205	5.817	0.00244	5.817	0.00277	5.817	0.00303	5.817	0.00327
5.818	0.00205	5.818	0.00244	5.818	0.00276	5.818	0.00303	5.818	0.00326
5.819	0.00205	5.819	0.00244	5.819	0.00276	5.819	0.00303	5.819	0.00326
5.82	0.00205	5.82	0.00244	5.82	0.00276	5.82	0.00303	5.82	0.00326
5.821	0.00205	5.821	0.00244	5.821	0.00276	5.821	0.00303	5.821	0.00326
5.822	0.00205	5.822	0.00244	5.822	0.00276	5.822	0.00303	5.822	0.00326
5.823	0.00205	5.823	0.00244	5.823	0.00276	5.823	0.00303	5.823	0.00326
5.824	0.00205	5.824	0.00244	5.824	0.00276	5.824	0.00303	5.824	0.00326
5.825	0.00205	5.825	0.00244	5.825	0.00276	5.825	0.00303	5.825	0.00326
5.826	0.00205	5.826	0.00244	5.826	0.00276	5.826	0.00303	5.826	0.00326
5.827	0.00205	5.827	0.00244	5.827	0.00276	5.827	0.00303	5.827	0.00326
5.828	0.00205	5.828	0.00244	5.828	0.00276	5.828	0.00303	5.828	0.00326
5.829	0.00205	5.829	0.00244	5.829	0.00276	5.829	0.00303	5.829	0.00326
5.83	0.00205	5.83	0.00244	5.83	0.00276	5.83	0.00303	5.83	0.00326
5.831	0.00205	5.831	0.00244	5.831	0.00276	5.831	0.00303	5.831	0.00326
5.832	0.00205	5.832	0.00244	5.832	0.00276	5.832	0.00303	5.832	0.00326

5.833	0.00205	5.833	0.00244	5.833	0.00276	5.833	0.00303	5.833	0.00326
5.834	0.00204	5.834	0.00244	5.834	0.00276	5.834	0.00303	5.834	0.00326
5.835	0.00204	5.835	0.00244	5.835	0.00276	5.835	0.00303	5.835	0.00325
5.836	0.00204	5.836	0.00244	5.836	0.00276	5.836	0.00302	5.836	0.00325
5.837	0.00204	5.837	0.00244	5.837	0.00276	5.837	0.00302	5.837	0.00325
5.838	0.00204	5.838	0.00244	5.838	0.00276	5.838	0.00302	5.838	0.00325
5.839	0.00204	5.839	0.00243	5.839	0.00275	5.839	0.00302	5.839	0.00325
5.84	0.00204	5.84	0.00243	5.84	0.00275	5.84	0.00302	5.84	0.00325
5.841	0.00204	5.841	0.00243	5.841	0.00275	5.841	0.00302	5.841	0.00325
5.842	0.00204	5.842	0.00243	5.842	0.00275	5.842	0.00302	5.842	0.00325
5.843	0.00204	5.843	0.00243	5.843	0.00275	5.843	0.00302	5.843	0.00325
5.844	0.00204	5.844	0.00243	5.844	0.00275	5.844	0.00302	5.844	0.00325
5.845	0.00204	5.845	0.00243	5.845	0.00275	5.845	0.00302	5.845	0.00325
5.846	0.00204	5.846	0.00243	5.846	0.00275	5.846	0.00302	5.846	0.00325
5.847	0.00204	5.847	0.00243	5.847	0.00275	5.847	0.00302	5.847	0.00325
5.848	0.00204	5.848	0.00243	5.848	0.00275	5.848	0.00302	5.848	0.00325
5.849	0.00204	5.849	0.00243	5.849	0.00275	5.849	0.00302	5.849	0.00325
5.85	0.00204	5.85	0.00243	5.85	0.00275	5.85	0.00302	5.85	0.00325
5.851	0.00204	5.851	0.00243	5.851	0.00275	5.851	0.00302	5.851	0.00325
5.852	0.00204	5.852	0.00243	5.852	0.00275	5.852	0.00302	5.852	0.00325
5.853	0.00204	5.853	0.00243	5.853	0.00275	5.853	0.00302	5.853	0.00324
5.854	0.00204	5.854	0.00243	5.854	0.00275	5.854	0.00302	5.854	0.00324
5.855	0.00204	5.855	0.00243	5.855	0.00275	5.855	0.00301	5.855	0.00324
5.856	0.00204	5.856	0.00243	5.856	0.00275	5.856	0.00301	5.856	0.00324
5.857	0.00204	5.857	0.00243	5.857	0.00275	5.857	0.00301	5.857	0.00324
5.858	0.00204	5.858	0.00243	5.858	0.00275	5.858	0.00301	5.858	0.00324
5.859	0.00204	5.859	0.00243	5.859	0.00275	5.859	0.00301	5.859	0.00324
5.86	0.00204	5.86	0.00243	5.86	0.00274	5.86	0.00301	5.86	0.00324
5.861	0.00204	5.861	0.00243	5.861	0.00274	5.861	0.00301	5.861	0.00324
5.862	0.00204	5.862	0.00243	5.862	0.00274	5.862	0.00301	5.862	0.00324
5.863	0.00203	5.863	0.00242	5.863	0.00274	5.863	0.00301	5.863	0.00324
5.864	0.00203	5.864	0.00242	5.864	0.00274	5.864	0.00301	5.864	0.00324
5.865	0.00203	5.865	0.00242	5.865	0.00274	5.865	0.00301	5.865	0.00324
5.866	0.00203	5.866	0.00242	5.866	0.00274	5.866	0.00301	5.866	0.00324
5.867	0.00203	5.867	0.00242	5.867	0.00274	5.867	0.00301	5.867	0.00324
5.868	0.00203	5.868	0.00242	5.868	0.00274	5.868	0.00301	5.868	0.00324
5.869	0.00203	5.869	0.00242	5.869	0.00274	5.869	0.00301	5.869	0.00324
5.87	0.00203	5.87	0.00242	5.87	0.00274	5.87	0.00301	5.87	0.00324
5.871	0.00203	5.871	0.00242	5.871	0.00274	5.871	0.00301	5.871	0.00324
5.872	0.00203	5.872	0.00242	5.872	0.00274	5.872	0.00301	5.872	0.00323
5.873	0.00203	5.873	0.00242	5.873	0.00274	5.873	0.00301	5.873	0.00323
5.874	0.00203	5.874	0.00242	5.874	0.00274	5.874	0.00301	5.874	0.00323
5.875	0.00203	5.875	0.00242	5.875	0.00274	5.875	0.003	5.875	0.00323
5.876	0.00203	5.876	0.00242	5.876	0.00274	5.876	0.003	5.876	0.00323
5.877	0.00203	5.877	0.00242	5.877	0.00274	5.877	0.003	5.877	0.00323
5.878	0.00203	5.878	0.00242	5.878	0.00274	5.878	0.003	5.878	0.00323
5.879	0.00203	5.879	0.00242	5.879	0.00274	5.879	0.003	5.879	0.00323
5.88	0.00203	5.88	0.00242	5.88	0.00274	5.88	0.003	5.88	0.00323
5.881	0.00203	5.881	0.00242	5.881	0.00274	5.881	0.003	5.881	0.00323
5.882	0.00203	5.882	0.00242	5.882	0.00273	5.882	0.003	5.882	0.00323
5.883	0.00203	5.883	0.00242	5.883	0.00273	5.883	0.003	5.883	0.00323
5.884	0.00203	5.884	0.00242	5.884	0.00273	5.884	0.003	5.884	0.00323
5.885	0.00203	5.885	0.00242	5.885	0.00273	5.885	0.003	5.885	0.00323
5.886	0.00203	5.886	0.00242	5.886	0.00273	5.886	0.003	5.886	0.00323
5.887	0.00203	5.887	0.00241	5.887	0.00273	5.887	0.003	5.887	0.00323
5.888	0.00203	5.888	0.00241	5.888	0.00273	5.888	0.003	5.888	0.00323
5.889	0.00203	5.889	0.00241	5.889	0.00273	5.889	0.003	5.889	0.00323
5.89	0.00203	5.89	0.00241	5.89	0.00273	5.89	0.003	5.89	0.00322
5.891	0.00203	5.891	0.00241	5.891	0.00273	5.891	0.003	5.891	0.00322
5.892	0.00202	5.892	0.00241	5.892	0.00273	5.892	0.003	5.892	0.00322
5.893	0.00202	5.893	0.00241	5.893	0.00273	5.893	0.003	5.893	0.00322
5.894	0.00202	5.894	0.00241	5.894	0.00273	5.894	0.00299	5.894	0.00322
5.895	0.00202	5.895	0.00241	5.895	0.00273	5.895	0.00299	5.895	0.00322
5.896	0.00202	5.896	0.00241	5.896	0.00273	5.896	0.00299	5.896	0.00322
5.897	0.00202	5.897	0.00241	5.897	0.00273	5.897	0.00299	5.897	0.00322
5.898	0.00202	5.898	0.00241	5.898	0.00273	5.898	0.00299	5.898	0.00322
5.899	0.00202	5.899	0.00241	5.899	0.00273	5.899	0.00299	5.899	0.00322
5.9	0.00202	5.9	0.00241	5.9	0.00273	5.9	0.00299	5.9	0.00322
5.901	0.00202	5.901	0.00241	5.901	0.00273	5.901	0.00299	5.901	0.00322
5.902	0.00202	5.902	0.00241	5.902	0.00273	5.902	0.00299	5.902	0.00322
5.903	0.00202	5.903	0.00241	5.903	0.00272	5.903	0.00299	5.903	0.00322
5.904	0.00202	5.904	0.00241	5.904	0.00272	5.904	0.00299	5.904	0.00322
5.905	0.00202	5.905	0.00241	5.905	0.00272	5.905	0.00299	5.905	0.00322
5.906	0.00202	5.906	0.00241	5.906	0.00272	5.906	0.00299	5.906	0.00322
5.907	0.00202	5.907	0.00241	5.907	0.00272	5.907	0.00299	5.907	0.00322
5.908	0.00202	5.908	0.00241	5.908	0.00272	5.908	0.00299	5.908	0.00321
5.909	0.00202	5.909	0.00241	5.909	0.00272	5.909	0.00299	5.909	0.00321
5.91	0.00202	5.91	0.00241	5.91	0.00272	5.91	0.00299	5.91	0.00321
5.911	0.00202	5.911	0.00241	5.911	0.00272	5.911	0.00299	5.911	0.00321
5.912	0.00202	5.912	0.0024	5.912	0.00272	5.912	0.00299	5.912	0.00321
5.913	0.00202	5.913	0.0024	5.913	0.00272	5.913	0.00299	5.913	0.00321
5.914	0.00202	5.914	0.0024	5.914	0.00272	5.914	0.00298	5.914	0.00321
5.915	0.00202	5.915	0.0024	5.915	0.00272	5.915	0.00298	5.915	0.00321
5.916	0.00202	5.916	0.0024	5.916	0.00272	5.916	0.00298	5.916	0.00321
5.917	0.00202	5.917	0.0024	5.917	0.00272	5.917	0.00298	5.917	0.00321
5.918	0.00202	5.918	0.0024	5.918	0.00272	5.918	0.00298	5.918	0.00321
5.919	0.00202	5.919	0.0024	5.919	0.00272	5.919	0.00298	5.919	0.00321
5.92	0.00202	5.92	0.0024	5.92	0.00272	5.92	0.00298	5.92	0.00321
5.921	0.00201	5.921	0.0024	5.921	0.00272	5.921	0.00298	5.921	0.00321
5.922	0.00201	5.922	0.0024	5.922	0.00272	5.922	0.00298	5.922	0.00321
5.923	0.00201	5.923	0.0024	5.923	0.00272	5.923	0.00298	5.923	0.00321
5.924	0.00201	5.924	0.0024	5.924	0.00272	5.924	0.00298	5.924	0.00321
5.925	0.00201	5.925	0.0024	5.925	0.00271	5.925	0.00298	5.925	0.00321
5.926	0.00201	5.926	0.0024	5.926	0.00271	5.926	0.00298	5.926	0.00321
5.927	0.00201	5.927	0.0024	5.927	0.00271	5.927	0.00298	5.927	0.0032
5.928	0.00201	5.928	0.0024	5.928	0.00271	5.928	0.00298	5.928	0.0032
5.929	0.00201	5.929	0.0024	5.929	0.00271	5.929	0.00298	5.929	0.0032
5.93	0.00201	5.93	0.0024	5.93	0.00271	5.93	0.00298	5.93	0.0032
5.931	0.00201	5.931	0.0024	5.931	0.00271	5.931	0.00298	5.931	0.0032
5.932	0.00201	5.932	0.0024	5.932	0.00271	5.932	0.00298	5.932	0.0032
5.933	0.00201	5.933	0.0024	5.933	0.00271	5.933	0.00298	5.933	0.0032
5.934	0.00201	5.934	0.0024	5.934	0.00271	5.934	0.00297	5.934	0.0032
5.935	0.00201	5.935	0.0024	5.935	0.00271	5.935	0.00297	5.935	0.0032
5.936	0.00201	5.936	0.00239	5.936	0.00271	5.936	0.00297	5.936	0.0032
5.937	0.00201	5.937	0.00239	5.937	0.00271	5.937	0.00297	5.937	0.0032
5.938	0.00201	5.938	0.00239	5.938	0.00271	5.938	0.00297	5.938	0.0032
5.939	0.00201	5.939	0.00239	5.939	0.00271	5.939	0.00297	5.939	0.0032
5.94	0.00201	5.94	0.00239	5.94	0.00271	5.94	0.00297	5.94	0.0032
5.941	0.00201	5.941	0.00239	5.941	0.00271	5.941	0.00297	5.941	0.0032
5.942	0.00201	5.942	0.00239	5.942	0.00271	5.942	0.00297	5.942	0.0032
5.943	0.00201	5.943	0.00239	5.943	0.00271	5.943	0.00297	5.943	0.0032
5.944	0.00201	5.944	0.00239	5.944	0.00271	5.944	0.00297	5.944	0.0032
5.945	0.00201	5.945	0.00239	5.945	0.00271	5.945	0.00297	5.945	0.00319
5.946	0.00201	5.946	0.00239	5.946	0.00271	5.946	0.00297	5.946	0.00319
5.947	0.00201	5.947	0.00239	5.947	0.0027	5.947	0.00297	5.947	0.00319
5.948	0.00201	5.948	0.00239	5.948	0.0027	5.948	0.00297	5.948	0.00319
5.949	0.00201	5.949	0.00239	5.949	0.0027	5.949	0.00297	5.949	0.00319
5.95	0.002	5.95	0.00239	5.95	0.0027	5.95	0.00297	5.95	0.00319
5.951	0.002	5.951	0.00239	5.951	0.0027	5.951	0.00297	5.951	0.00319
5.952	0.002	5.952	0.00239	5.952	0.0027	5.952	0.00297	5.952	0.00319
5.953	0.002	5.953	0.00239	5.953	0.0027	5.953	0.00297	5.953	0.00319
5.954	0.002	5.954	0.00239	5.954	0.0027	5.954	0.00296	5.954	0.00319
5.955	0.002	5.955	0.00239	5.955	0.0027	5.955	0.00296	5.955	0.00319
5.956	0.002	5.956	0.00239	5.956	0.0027	5.956	0.00296	5.956	0.00319
5.957	0.002	5.957	0.00239	5.957	0.0027	5.957	0.00296	5.957	0.00319
5.958	0.002	5.958	0.00239	5.958	0.0027	5.958	0.00296	5.958	0.00319
5.959	0.002	5.959	0.00239	5.959	0.0027	5.959	0.00296	5.959	0.00319
5.96	0.002	5.96	0.00239	5.96	0.0027	5.96	0.00296	5.96	0.00319
5.961	0.002	5.961	0.00238	5.961	0.0027	5.961	0.00296	5.961	0.00319
5.962	0.002	5.962	0.00238	5.962	0.0027	5.962	0.00296	5.962	0.00319
5.963	0.002	5.963	0.00238	5.963	0.0027	5.963	0.00296	5.963	0.00319
5.964	0.002	5.964	0.00238	5.964	0.0027	5.964	0.00296	5.964	0.00318
5.965	0.002	5.965	0.00238	5.965	0.0027	5.965	0.00296	5.965	0.00318
5.966	0.002	5.966	0.00238	5.966	0.0027	5.966	0.00296	5.966	0.00318
5.967	0.002	5.967	0.00238	5.967	0.0027	5.967	0.00296	5.967	0.00318

5.968	0.002	5.968	0.00238	5.968	0.0027	5.968	0.00296	5.968	0.00318
5.969	0.002	5.969	0.00238	5.969	0.00269	5.969	0.00296	5.969	0.00318
5.97	0.002	5.97	0.00238	5.97	0.00269	5.97	0.00296	5.97	0.00318
5.971	0.002	5.971	0.00238	5.971	0.00269	5.971	0.00296	5.971	0.00318
5.972	0.002	5.972	0.00238	5.972	0.00269	5.972	0.00296	5.972	0.00318
5.973	0.002	5.973	0.00238	5.973	0.00269	5.973	0.00296	5.973	0.00318
5.974	0.002	5.974	0.00238	5.974	0.00269	5.974	0.00295	5.974	0.00318
5.975	0.002	5.975	0.00238	5.975	0.00269	5.975	0.00295	5.975	0.00318
5.976	0.002	5.976	0.00238	5.976	0.00269	5.976	0.00295	5.976	0.00318
5.977	0.002	5.977	0.00238	5.977	0.00269	5.977	0.00295	5.977	0.00318
5.978	0.002	5.978	0.00238	5.978	0.00269	5.978	0.00295	5.978	0.00318
5.979	0.002	5.979	0.00238	5.979	0.00269	5.979	0.00295	5.979	0.00318
5.98	0.00199	5.98	0.00238	5.98	0.00269	5.98	0.00295	5.98	0.00318
5.981	0.00199	5.981	0.00238	5.981	0.00269	5.981	0.00295	5.981	0.00318
5.982	0.00199	5.982	0.00238	5.982	0.00269	5.982	0.00295	5.982	0.00318
5.983	0.00199	5.983	0.00238	5.983	0.00269	5.983	0.00295	5.983	0.00317
5.984	0.00199	5.984	0.00238	5.984	0.00269	5.984	0.00295	5.984	0.00317
5.985	0.00199	5.985	0.00238	5.985	0.00269	5.985	0.00295	5.985	0.00317
5.986	0.00199	5.986	0.00237	5.986	0.00269	5.986	0.00295	5.986	0.00317
5.987	0.00199	5.987	0.00237	5.987	0.00269	5.987	0.00295	5.987	0.00317
5.988	0.00199	5.988	0.00237	5.988	0.00269	5.988	0.00295	5.988	0.00317
5.989	0.00199	5.989	0.00237	5.989	0.00269	5.989	0.00295	5.989	0.00317
5.99	0.00199	5.99	0.00237	5.99	0.00269	5.99	0.00295	5.99	0.00317
5.991	0.00199	5.991	0.00237	5.991	0.00268	5.991	0.00295	5.991	0.00317
5.992	0.00199	5.992	0.00237	5.992	0.00268	5.992	0.00295	5.992	0.00317
5.993	0.00199	5.993	0.00237	5.993	0.00268	5.993	0.00295	5.993	0.00317
5.994	0.00199	5.994	0.00237	5.994	0.00268	5.994	0.00294	5.994	0.00317
5.995	0.00199	5.995	0.00237	5.995	0.00268	5.995	0.00294	5.995	0.00317
5.996	0.00199	5.996	0.00237	5.996	0.00268	5.996	0.00294	5.996	0.00317
5.997	0.00199	5.997	0.00237	5.997	0.00268	5.997	0.00294	5.997	0.00317
5.998	0.00199	5.998	0.00237	5.998	0.00268	5.998	0.00294	5.998	0.00317
5.999	0.00199	5.999	0.00237	5.999	0.00268	5.999	0.00294	5.999	0.00317
6	0.00199	6	0.00237	6	0.00268	6	0.00294	6	0.00317

intensity parameter b	R_h/R_0	R_s/R_0	R_f/R_0
0	1.6258	1.60275	1.37427
0.1	1.60969	1.58646	1.36091
0.2	1.59239	1.56896	1.34655
0.3	1.57373	1.55006	1.33106
0.4	1.55348	1.52957	1.31425
0.5	1.53139	1.50719	1.29589
0.6	1.50711	1.4826	1.2757
0.7	1.48022	1.45537	1.25333
0.8	1.45018	1.42495	1.2283
0.9	1.41625	1.39059	1.2
1	1.37747	1.35133	1.16759

b	ψ_i	η_i
0	0	1
0	5	1.191
0	10	1.42
0	15	1.698
0	20	2.04
0.25	0	1
0.25	5	1.212
0.25	10	1.467
0.25	15	1.776
0.25	20	2.155
0.5	0	1
0.5	5	1.229
0.5	10	1.504
0.5	15	1.838
0.5	20	2.248
0.75	0	1
0.75	5	1.243
0.75	10	1.535
0.75	15	1.889
0.75	20	2.323
1	0	1
1	5	1.255
1	10	1.56
1	15	1.931
1	20	2.386
